# Stereoselective gold(I)-catalyzed approach to the synthesis of complex α-glycosyl phosphosaccharides

**DOI:** 10.1038/s41467-022-28025-0

**Published:** 2022-01-20

**Authors:** Xiaojuan Zhang, Yutong Yang, Jiahao Ding, Yun Zhao, Hongbin Zhang, Yugen Zhu

**Affiliations:** Key Laboratory of Medicinal Chemistry for Natural Resource, Ministry of Education; Yunnan Provincial Center for Research & Development of Natural Products; School of Chemical Science and Technology, Yunnan University, Kunming, Yunnan 650091 People’s Republic of China

**Keywords:** Carbohydrate chemistry, Stereochemistry, Synthetic chemistry methodology, Sugar phosphates

## Abstract

Glycosyl phosphosaccharides represent a large and important family of complex glycans. Due to the distinct nature of these complex molecules, efficient approaches to access glycosyl phosphosaccharides are still in great demand. Here, we disclose a highly efficient and stereoselective approach to the synthesis of biologically important and complex *α*-glycosyl phosphosaccharides, employing direct gold(I)-catalyzed glycosylation of the weakly nucleophilic phosphoric acid acceptors. In this work, the broad substrate scope is demonstrated with more than 45 examples, including glucose, xylose, glucuronate, galactose, mannose, rhamnose, fucose, 2-N_3_-2-deoxymannose, 2-N_3_-2-deoxyglucose, 2-N_3_-2-deoxygalactose and unnatural carbohydrates. Here, we show the glycosyl phosphotriester prepared herein was successfully applied to the one-pot synthesis of a phosphosaccharide from *Leishmania donovani*, and an effective preparation of a trisaccharide diphosphate of phosphosaccharide fragments from *Hansenula capsulate* via iterative elongation strategy is realized.

## Abstract (web summary)

Glycosyl phosphosaccharides represent a large and important family of complex glycans, but are difficult to synthesize efficiently. Here, the authors disclose a stereoselective methodology to make α-glycosyl phosphosaccharides, via gold(I)-catalyzed glycosylation of phosphoric acid acceptors.

## Introduction

**Fig. 1:**
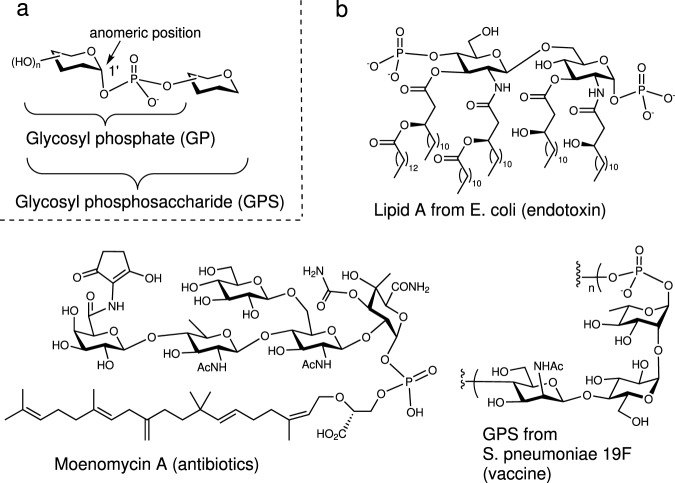
Introduction of glycosyl phosphate and glycosyl phosphosaccharide. **a** Nomenclatures for glycosyl phosphate and phosphosaccharide. **b** Representative bioactive phosphosaccharides.

Glycosyl phosphosaccharides (GPSs) represent a large and important family of complex glycans, which are ubiquitously distributed in bacteria, yeasts, protozoan parasites and animals, and exhibit numerous bio-functions including bacterial infections, cell adhesive, immunoresponse, and antimicrobial (Fig. 1b)^1–5^. The GPSs consist of anomeric glycosyl phosphates in which the anomeric position of one constituent glycan was linked to another one mainly by α-type phosphodiester linkage (Fig. 1a). In the process of carbohydrate metabolism, the constituent glycosyl phosphates (GPs) are significant intermediates^6^. Synthetically, protected GPs have been utilized as effective glycosyl donor reagents^7^, and under the catalysis of bis-thiourea, the armed GPSs proceed an S_*N*_-2 glycosylation pathway^8^. Despite the significance of GPSs, efficient approaches to access GPSs are rather limited: isolation from cell culture hardly affords homogeneous sample and chemical synthesis remains challenging due to the character of complex structure. Accordingly, efficient methods of constructing homogeneous GPSs and GPs are still in great demand.

**Fig. 2:**
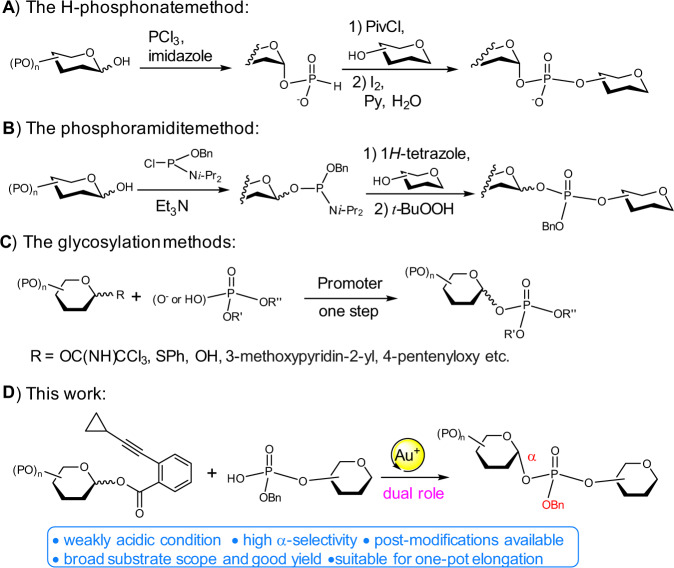
Profile of approaches to glycosyl phosphosaccharides and our method. In red are the α-configuration of the formed glycosidic bond and the benzyl (Bn) group. In yellow is the gold(I) catalysis. In pink is the indication of the role of gold(I). In blue are the features of the present approach.

In addition to intrinsically labile character, the anomeric stereocontrol of forming α-GPSs, most of which assume 1,2-*cis* configuration, is regarded as a challenging task^9–17^. The synthetic method of H-phosphonate chemistry has found extensive applications in the synthesis of α-GPSs, while two-step transformations of nucleophilic displacement and oxidation are inevitable (Fig. 2A)^2,18,19^. Nevertheless, the resulted phosphate anions render product incompatible with follow-up or late-stage chemical modifications to increase molecular complexity and diversity^20^. The alternative approach employing phosphoramidite displays great success in installation of phosphoester, yet is rarely applied to synthesis of anomeric GPS probably due to issues of diastereo-selectivity and undesired oxidative cleavage reaction (Fig. 2B)^2,21–23^.

One-step and direct glycosylation of phosphate acceptors with glycosyl donors represents a convergent and concise method for the synthesis of α-GPSs, which does not require oxidation transformation (Fig. 2C). This glycosylation strategy to access α-GPSs was previous realized by utilizing glycosyl trichloroacetimidate as donor and the weak nucleophile of phosphoric acid as acceptor under the action of strong acid^24^, and later, by a panel of donors with different leaving groups (e.g., SPh, 3-methoxypyridin-2-yl, pentenyloxy)^25–31^. The efficiency of these glycosylation reactions with phosphates as acceptors remains unmet: (1) the yield was deteriorated when strong acid was used to realize α-stereoselectivity^24^, (2) stoichiometric base was applied to preserve the entity of GPs, but leading to poor 1,2-*cis* stereoselectivity^28^, (3) only a handful of complex disaccharide GPSs were accessed by using the direct glycosylation strategy with phosphate anion as acceptor^25^.

Catalytic glycosylation methods have emerged as an appealing approach to the synthesis of carbohydrates, which feature less promoter and waste, and high efficiency^32^. Among those, alkynylphilic gold(I) catalysis has been extensively applied in the syntheses of numerous complex glycans and glycoconjugates^33^, along with other natural products^34^, by exploiting the compatibility with oxygen-containing functionalities^35^. Especially, glycosyl donor with *ortho*-alkynylbenzoate as leaving group first introduced by Yu and coworkers can glycosylate a variety of acceptors^36,37^. Nevertheless, the glycosylation of exceedingly poor nucleophile of phosphoric acid remains elusive, which entails mild conditions free from competitive nucleophilic species.

In this work, we disclose a stereoselective and general approach to the synthesis of α-GPs and α-GPSs via a gold(I)-catalyzed glycosylation method with glycosyl *ortho*-alkynylbenzoate as donor and weakly nucleophilic phosphoric acid as acceptor. While the alkynylphilicity of gold(I) catalysis has been widely investigated and applied, the Lewis acid property when oxygen-containing functionalities is activated by gold(I) remains much less explored. Herein, both the alkynylphilicity and weak acidity of gold(I) catalyst are capitalized on to initiate the glycosylation reaction and promote epimerization to α-anomer, respectively (Fig. 2D). Moreover, the anomeric effect, which is conferred by the weak nucleophile of phosphoric acid in the glycosylation reaction, is also exploited to direct the α-selectivity under the present weakly acidic condition^38,39^. The present gold(I)-catalyzed glycosylation method facilitates the efficient synthesis of more than 45 complex glycosyl phosphates, one-pot synthesis of GPS from *Leishmania donovani*, and iterative elongation of a trisaccharide diphosphate of GPS fragments from *Hansenula capsulate*. Mechanistic studies are investigated to indicate the dual role of Ph_3_PAuNTf_2_ in the glycosylation-epimerization process, in which Ph_3_PAuNTf_2_ not only triggers the glycosylation reaction, but also subsequently promotes the epimerization of the α/β products to enrich the α one.

## Results

### Reaction optimization

To test the validity of this proposal, tetrabenzyl glucoside **1a** was examined with a structurally simple acceptor of phosphoric acid dibenzyl ester **2a** (Table 1). Initial experiment gave promising results with a good yield of α/β mixture (88%) and α-selectivity (α/β = 2.8/1, entry 1). Thus, detailed optimizations were subsequently conducted by tuning reaction temperature, solvent and additive. As depicted in Table 1, lowering temperature was not effective (83%, α/β = 2/1, entry 2); replacing the anion of Ph_3_PAuNTf_2_ with ^−^OTf diminished α-selectivity (entry 3). The ether solvent and additive of Ph_3_P = O, which direct α-selectivity when alcohols are used as acceptors, did not lead to satisfactory results in the present reaction (entries 4-6)^11^. Gratifyingly, the diastereoselective ratio was raised to 10/1 by an added HOTf (0.1 equiv.), which is supposed to thermodynamically equilibrated β-anomer to the α-one, but compromise the overall yield (62%, entry 7). The strong and heterogenous acidic H^+^ resin did not result in an improvement in diastereoselectivity as that of HOTf (entry 8). Then, the homogeneous and weak Lewis acid of gold(I) catalysis under an elevated temperature was anticipated to trigger the epimerization to enrich α-anomer and maintain a high yield. Indeed, after complete glycosylation of **2a** at 0 °C for 0.5 h, keeping the mixture at 60 °C for 2 h in non-coordinating ClCH_2_CH_2_Cl (DCE) for anomerization produced the α-anomer in good selectivity (α/β = 9/1) without deteriorating yield (87%, entry 9). Further elevation of temperature (75 °C and 95 °C) resulted in a drop of yield or decomposition of product (entry 10, 11). Fortunately, increasing the equivalence of donor **1a** to 1.5 relative to acceptor **2a** (1.0 equiv.) reached an exceptional ratio of 16/1 in 62% yield which was calculated based on donor (entry 12). Interestingly, decreasing the equivalence of donor to 1.0 relative to acceptor (1.0 equiv.) significantly lowered the yield and α-selectivity (entry 13). While prolonging the reaction time for epimerization from 2 h to 3 h gave higher α-selectivity of 20/1, the yield dropped to 57% (entry 14). Notably, the yields of α/β mixtures were herein reported owing to that the α/β mixture was not separable by utilizing flash silica gel column chromatography, and the yields were calculated based on the donor which was used in excess (1.5 equiv.).

**Table 1:** Optimizations of the reaction conditions.


Entry	Temperature	Solvent	Additive	Yield, α/β ^*a, b*^
1	20 °C	CH_2_Cl_2_	—	88%, 2.8/1
2	−78 to 0 °C	CH_2_Cl_2_	—	83%, 2/1
3^*c*^	20 °C	CH_2_Cl_2_	—	86%, 1.5/1
4	20 °C	Et_2_O	—	88%, 3.2/1
5	20 °C	1,4-dioxane/toluene	—	60%, 1.2/1
6	20 °C	CH_2_Cl_2_	Ph_3_P = O	80%, 1/1
7	20 °C	Et_2_O	HOTf	62%, 10/1
8	20 °C	Et_2_O	H^+^ resin	78%, 2.9/1
9	0 °C then 60 °C	ClCH_2_CH_2_Cl	—	87%, 9/1
10	0 °C then 75 °C	ClCH_2_CH_2_Cl	—	75%, 9/1
11	0 °C then 95 °C	ClCH_2_CH_2_Cl	—	0
12^*d*^	0 °C then 60 °C	ClCH_2_CH_2_Cl	—	62%, 16/1
13^*d, e*^	0 °C then 60 °C	ClCH_2_CH_2_Cl	—	85%, 9.7/1
14^*d, f*^	0 °C then 60 °C	ClCH_2_CH_2_Cl	—	57%, 20/1

^*a*^Reaction condition: **1a** (0.05 mmol), **2a** (0.075 mmol), solvent (1.0 mL), Ph_3_PAuNTf_2_ (10 mol%), 5 Å MS, 2 h. ^*b*^Yields of α/β mixture (α/β mixture not separable), and ratios determined by HPLC. ^*c*^Ph_3_PAuOTf was used instead. ^*d*^Optimized condition: **1a** (0.075 mmol) and **2a** (0.05 mmol), Ph_3_PAuNTf_2_ (10 mol%), DCE (1 mL), 0 °C, 30 min, then 60 °C, 2 h. ^*e*^**1a** (0.05 mmol) and **2a** (0.05 mmol) were used instead. ^*f*^Reaction time was prolonged from 2 h to 3 h. Tf: trifluoromethanesulfonyl.

### Reaction scope of donors

**Fig. 3:**
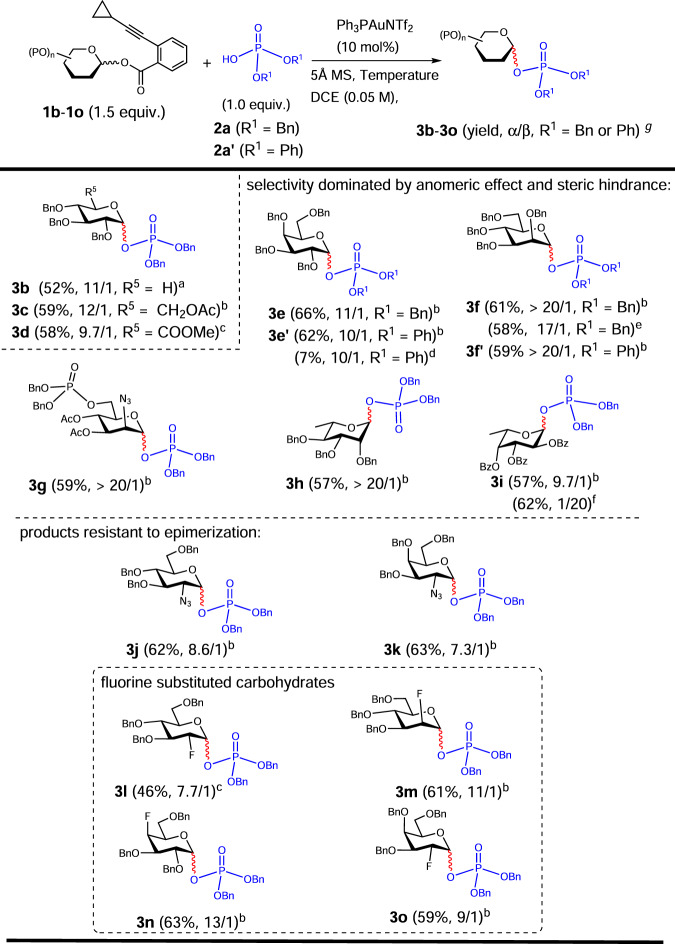
The reaction scope of various glycosyl donors. ^a^Temperature: 0 °C, 30 min then 60 °C, 2 h. ^b^Temperature: 0 °C, 30 min, then 20 °C, 2 h. ^c^Temperature: 0 °C, 30 min, then 100 °C, 2 h. ^d^galactosyl trichloroacetimidate was used as donor in the presence of TMSOTf (0.1 equiv.) at 0 °C in 30 min. ^e^On 1.2 g scale with 0.5 mol% Ph_3_PAuNTf_2_. ^f^0.2 equiv. of *i-*Pr_2_NEt was used as additive. ^g^Yields of α/β mixtures (α/β mixtures were not separable, except **3e** and **3i**), and ratios were determined by crude ^31^P NMR. Bn: benzyl. Bz: benzoyl. Ph: phenyl. Ac: acetyl. Tf: trifluoromethanesulfonyl. In red are the formed glycosidic bonds. In blue are the acceptors of phosphoric acids and the phosphate moieties in the products.

Next, we wondered whether this gold (I)-catalyzed glycosylation strategy was amenable to various glycosyl donors outfitted with different protecting groups or configurations (Fig. 3). First, xylosyl donor **1b**, of which the BnOCH_2_ moiety is omitted compared to **1a**, delivered the expected xylosyl phosphate **3b** in a good diastereoselectivity (11/1) and 55% yield of α/β mixture calculated based on the donor (1.5 equiv.) by utilizing the aforementioned protocol. The electron-withdrawing groups such as AcOCH_2_ of 6-*O*-acetyl-glucosyl donor **1c** and COOMe of glucuronic acid methyl ester donor **1d** may greatly reduce the reactivity of donor, thereby causing difficulty in epimerization. Luckily, the 6-*O*-Ac product (**3c**) could be reached in a diastereoselective ratio of 12/1 merely at room temperature, of which α-selectivity is presumably dominated by anomeric effect^38,39^. For the other one equipped with COOMe, the product (**3d**) can be epimerized to enrich α-anomer (9.7/1) under a higher temperature of 100 °C with a yield of 58%.

Consequently, this strategy was extended to a variety of donors including Gal, Man, ManN_3_, Rha, Fuc, GlcN_3_, GalN_3_ and unnatural carbohydrates (Fig. 3). For the production of those with highly anomeric effect and dominated by steric hindrance, such as galactosyl phosphate (**3e** and **3e’**), mannosyl phosphate (**3** **f** and **3** **f’**), disarmed 2-N_3_-2-dexoy-mannosyl phosphate (**3** **g**), rhamnosyl phosphate (**3** **h**) and fucosyl phosphate (**3i**), an operationally simple procedure at room temperature was effective to access desired products in highly diastereoselective manners. However, switching to phenyl tetrabenzyl-1-thio-mannoside which was preactivated with *p*-TolSCl and AgOTf, followed by addition of **2a**, gave only byproduct **3p** derived from intramolecular cyclization between 2-*O* Bn and anomeric position (please see SI)^40^. Among those, **3** **g** represents constituent unit derived from capsule polysaccharide of *Neisseria meningitidis* A^41^. The straightforward syntheses of extremely unstable diphenyl phosphate **3e’**^8^ and **3** **f’** demonstrate the utility of this strategy for directly glycosylating weakly nucleophilic phosphoric acid. In comparison, only 7% yield was obtained when galactosyl trichloroacetimidate was used as donor in the presence of TMSOTf (0.1 equiv.) at 0 °C. Moreover, **3** **f** can be obtained on a 1.2 g scale with low catalytic loading of Ph_3_PAuNTf_2_ (0.5 mol%) albeit in slightly decreased diastereoselectivity (17/1) and yield (58%)^42^. The deprotected form of **3** **f** might serve as replacement therapy for the disease of congenital disorder of glycosylation type Ia which is under clinical trial^43^. Interestingly, 1,2-*cis* α-fucosyl phosphate **3i** was obtained even with neighboring-participating Bz situated at 2-*O*, demonstrating the strong anomeric effect in the case of phosphoric acid as acceptor. However, the diastereoselectivity can be reversed by adding extra base of iPr_2_NEt (0.2 equiv.) to form phosphate anion as acceptor.

Because of the distinct nature of N_3_ substituent in comparison with OBn, formation of 1,2-*cis* α-D-glycosamine glycosidic bond remains elusive in the case of alcohols as acceptors^14–17^. Although the azido substituted glycosyl phosphates are resistant to epimerization at high temperature, donors of tribenzyl GlcN_3_ and GalN_3_ underwent smoothly coupling reactions with good diastereoselective ratios of 8.6/1 (**3j**) and 7.3/1 (**3k**) which might find utility in the syntheses of Lipid A or other phosphosaccharides^2,44^

Derivatization and mimicking of natural glycosyl phosphates emerge as attractive tools to elucidate molecular mechanism of glycosyltransferases and discover novel therapeutic reagents^45,46^. Herein, a panel of fluorine-substituted α-GPs (**3l**-**3o**) were readily assembled via gold(I)-catalyzed glycosylation approach. Notably, the highly α-selective outcomes are in stark contrast to the reported results of fluorine-directed glycosylation with alcohol acceptors^47^.

### Reaction scope of acceptors

**Fig. 4:**
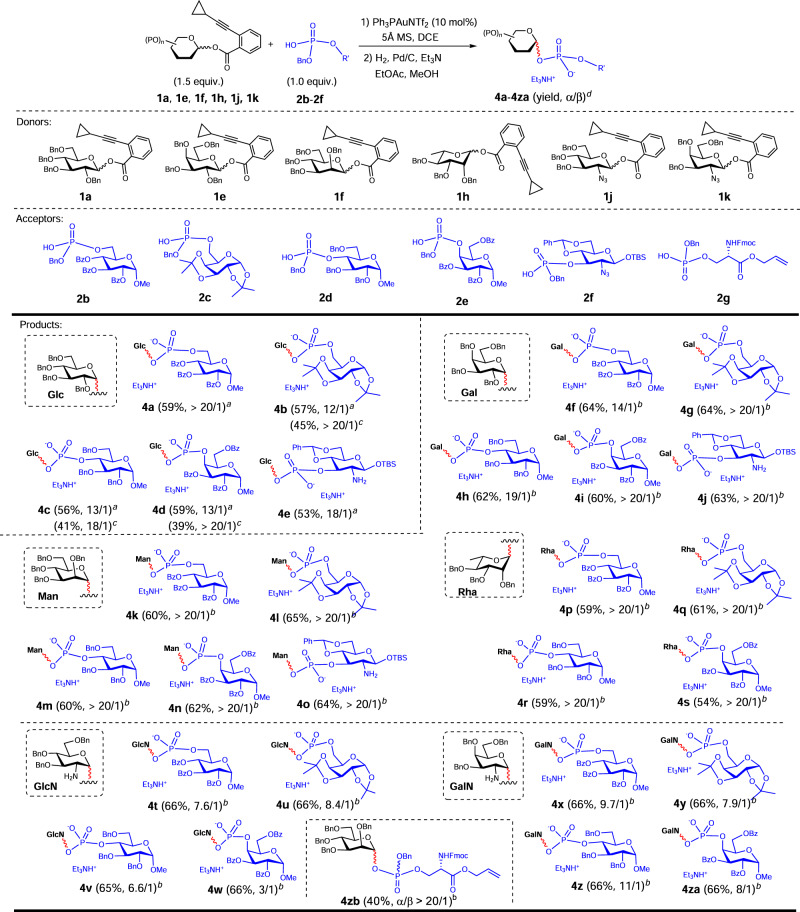
The reaction scope of various acceptors of phosphoric acids. ^a^Condition: 0 °C, 30 min then 60 °C, 2 h. ^b^Condition: 0 °C, 30 min, then 20 °C, 2 h. ^c^Condition: 0 °C, 30 min then 60 °C, 3 h. ^d^Yields of α/β mixtures (α/β mixtures not separable), and ratios were determined by ^31^P NMR. TBS: *tert*-butyldimethylsilyl. Bn: benzyl. Bz: benzoyl. Tf: trifluoromethanesulfonyl. Fmoc: 9-fluorenylmethyloxycarbonyl. In red are the formed glycosidic bonds. In blue are the acceptors of phosphoric acid and the phosphate moieties in the products.

After determining the generality of various glycosyl donors which glycosylated with phosphoric acid **2a**, we explored the possibility of extension to more complex phosphate nucleophiles. Thus, a set of structurally diverse acceptors of phosphoric acid glycosyl esters were readily prepared through a straightforward route of phosphorylation of alcohol and subsequent debenzylation (see SI), including 6-*O*-benzyloxyphosphoryl glucoside **2b** and **2c**, sterically hindered 4-*O*-benzyloxyphosphoryl glucoside **2d** and galactoside **2e**, and 3-*O*-benzyloxyphosphoryl glucoside **2** **f** outfitted with labile groups of TBS and benzylidene, and serinyl phosphate **2** **g** (Fig. 4). The carbohydrates widely distributed in natural GPSs were selected as glycosyl donors (glucose (Glc, **1a**), galactose (Gal, **1e**), mannose (Man, **1** **f**), rhamnose (Rha, **1** **h**), 2-N_3_-2-deoxyglucose (GlcN_3_, **1j**), 2-N_3_-2-deoxygalactose (GalN_3_, **1k**)), which led to twenty-seven bis-glycosyl benzylphosphotriesters. For convenience of characterization, the phosphorus chirality was eliminated by hydrogenolysis of benzyl phosphates (**4a-4za**), which simultaneously resulted in reduction of N_3_ (**4t-4z**, **4za**, **4e**, **4j**, **4o**). In detail, by using the protocol of glycosylation and subsequent anomerization, condensation of Glc donor (**1a**) and all the five acceptors (**2b**-**2f**) delivered the corresponding GPSs (**4a**-**4e**) in highly diastereoselective manners. The donors of Gal (**1e**), Man (**1** **f**) and Rha (**1** **h**) with highly anomeric effect produced GPSs (**4f**-**4s**) in invariably high α-selectivities. Although azido substituted substrates of GlcN_3_ and GalN_3_ are resistant to epimerization and display weaker α-configured bias, good results were attained with consistent stereoselectivities (**4t**-**4v**, **4x**-**4z**, **4za**), except **4w** which was formed in low stereoselectivity (3/1). Furthermore, one of phosphoglycoserines (**4zb**), which are found in parasites, was concisely assembled via glycosylation of serinyl phosphate **2** **g** in a stereospecific manner, albeit in a low yield of 40% because of limited solubility of **2** **g** in DCE^48^ (Fig. 4).

### Reaction scope of large donors

**Fig. 5:**
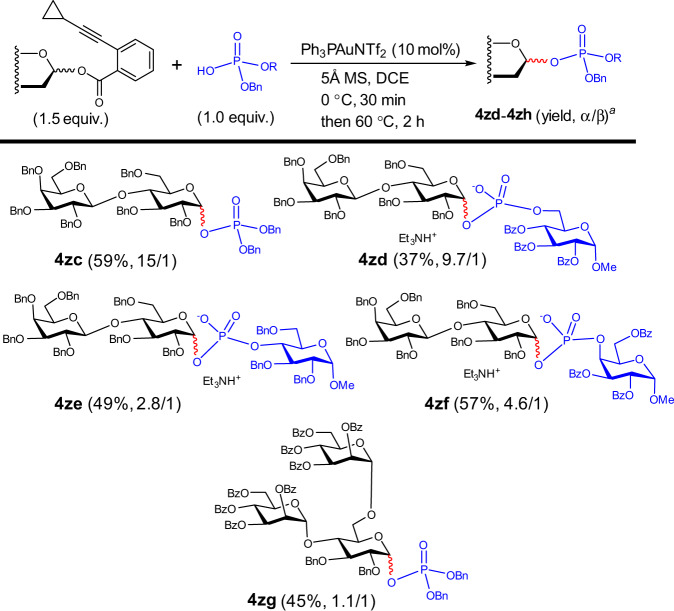
The epimerization-glycosylation reactions with large glycosyl donors. ^a^Yields of α/β mixtures (α/β mixtures were not separable), and α/β ratios were determined by ^31^P NMR. Bn: benzyl. Bz: benzoyl. In red are the formed glycosidic bonds. In blue are the acceptors of phosphoric acids and the phosphate moieties in the products.

The successful glycosylation-epimerization process with mono glucosyl donor encouraged us to evaluate large glucosyl donors (one disaccharide donor and one trisaccharide donor) with a non-participating group at 2-*O* position (Fig. 5). As shown in Fig. 5, while the glycosyl phosphoesters (**4zc**, **4zd**) with less hindered environment around the motif of glucosyl phosphate possessed good selectivity, higher steric hindrance had a detrimental effect on the α/β selectivity (**4ze** (2.8/1), **4zf** (4.6/1), **4zg** (1.1/1)).

### Global deprotection reactions

**Fig. 6:**
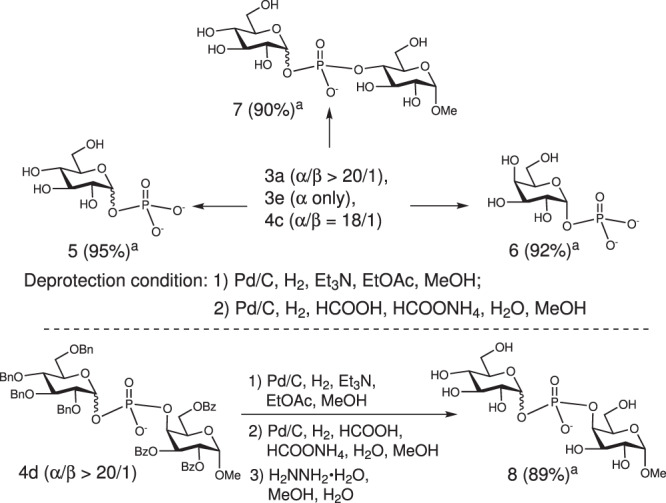
Global deprotection of armed glycosyl phosphoesters. ^a^Yields of isolated products. Bn: benzyl. Bz: benzoyl.

Although the prepared glycosyl phosphotriesters are rather labile toward acid, the corresponding anions readily prepared through hydrogenolysis exhibit remarkable inertness toward acid. Thus, the deprotection sequence for these glycosyl phosphotriesters could be carried out through a two-step protocol: removal of benzyl group of benzyl phosphate and global deprotection of other protecting groups (Fig. 6). The phosphotriester **3a** and **3e** were hydrogenated in the presence of Et_3_N to produce quantitatively intermediate compounds of glycosyl phosphomonoester, which were then subjected to global deprotection of all other benzyl groups even in the presence of acidic HCOOH to afford cleanly the product **5** and **6**. Similarly, the glycosyl phosphodiester **4c** were directly deprotected under the acidic hydrogenation condition to furnish **7** in excellent yield (90%). For the benzoyl protected **4d**, one additional step of deacetylation by using hydrazine after hydrogenation reaction was required to release the bis-glycosyl phosphoester **8** in high yield (89%).

### One-pot glycosylation

**Fig. 7:**
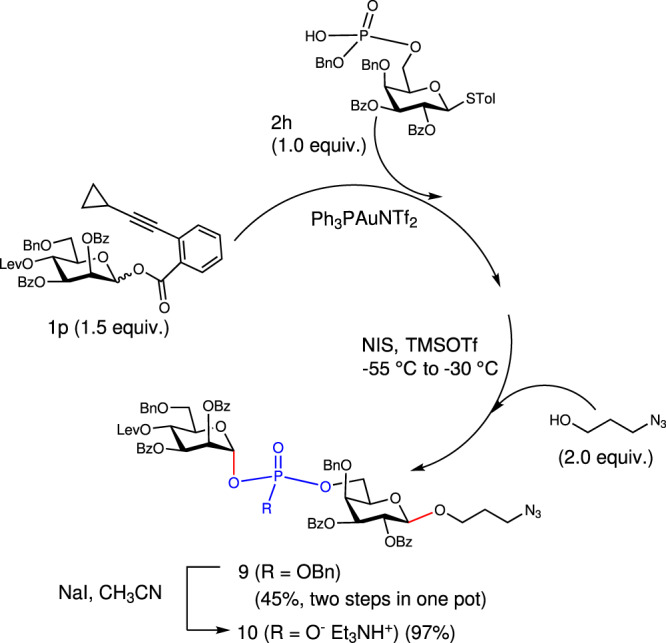
One-pot synthesis of GPS 9. NIS: *N*-iodosuccinimide. Tol: tolyl. Lev: levulinoyl. Bn: benzyl. Bz: benzoyl. TMSOTf: trimethylsilyl trifluoromethanesulfonate. In red are the formed glycosidic bonds. In blue is the phosphate moiety in product.

While one-pot glycosylation protocol emerges as versatile strategy to synthesize complex oligo/polysaccharides of which the units are tethered by acetal linkages^49–51^, this strategy is not applicable to the assembly of conventionally synthesized bis-glycosyl phosphodiester which incorporate reactive functionality of phosphate anion^20^. Gratifyingly, bis-glycosyl benzyl phosphotriesters readily prepared in our system could serve as attractive substrates for one-pot glycosylation, and described in Fig. 7 is an example, in which linker-tethered **9** was assembled in one pot via gold(I)-catalyzed glycosylation reaction and a follow-up orthogonal coupling reaction promoted by NIS and TMSOTf. The chirality of phosphorus atom can be eliminated by converting OBn to O^−^, generating a single stereomer (**10**, 97%) derived from phosphosaccharide of *Leishmania donovani*^1^.

### Iterative elongation

**Fig. 8:**
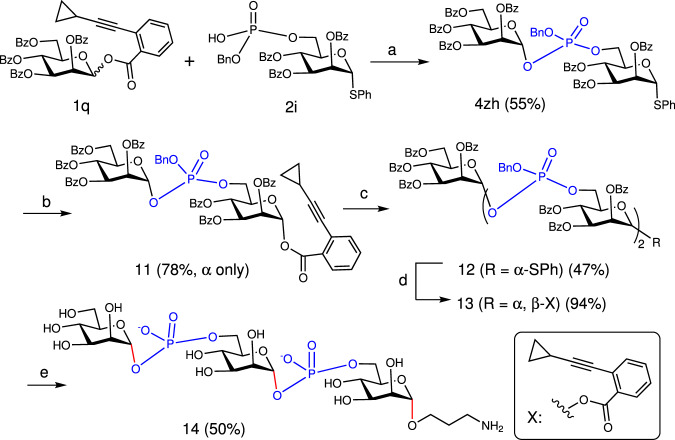
The synthetic route to GPS 14 via iterative elongation. **a** Ph_3_PAuNTf_2_ (10 mol%), 5 Å MS, DCE. **b** 1) NBS, acetone, H_2_O; 2) HX, EDCl, DMAP, CH_2_Cl_2_. **c** **2i**, Ph_3_PAuNTf_2_, DCE. **d** 1) NBS, acetone, H_2_O; 2) HX, EDCl, DMAP, CH_2_Cl_2._ **e** 1) 3-azidopropanol, Ph_3_PAuNTf_2_ (10 mol%); 2) H_2_, Pd/C, HCOOH, HCOONH_4_, THF, *t*-BuOH; 3) NH_2_NH_2_•H_2_O, MeOH. NBS: *N*-bromosuccinimide. EDCl: 1-(3-dimethylaminopropyl)-3-ethylcarbodiimide hydrochloride. DMAP: 4-(dimethylamino)pyridine. Bz: benzoyl. In red are the formed glycosidic bonds. In blue are the phosphate moieties in the products.

Finally, the utility of this gold(I)-catalyzed one-step approach to synthesis of GPSs was further illustrated by iterative elongation of phosphomannosyl fragments from *Hansenula capsulate* (Fig. 8)^52^. First, condensation of donor **1q** and acceptor **2i** furnished the desired phosphotriester **4zh**. Because **4zh** was resistant to preactivation by using *p*-TolSCl/AgOTf or BSP/Tf_2_O which led to only 30% yield and armed donor with benzyl groups was prone to give cyclized byproduct (e.g., **3p**)^40,53^, SPh was converted to *ortho*-alkynylbenzoate as leaving group via two steps. Next, condensation of **11** and **2i** generated a trisaccharide **12** consisting of two phosphotriester funcitonalities in 47% yield based on donor **11** (1.5 equiv.), which was subsequently converted to trisaccharide donor **13** in a procedure similar to that for **11**. As a late-stage chemical modification on this trisaccharide, a third glycosylation reaction between donor **13** and 3-azidopropanol was performed to install a linker with the two present phosphotriesters intact. Finally, the resulting trisaccharide was globally deprotected under mild conditions to afford an amino-linker tethered trisaccharide diphosphate **14**.

### Mechanistic studies

**Fig. 9:**
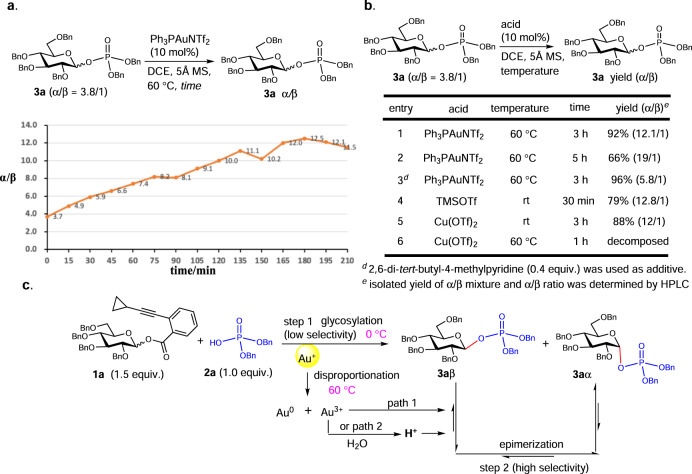
Mechanistic studies. **a** The plot of α/β ratio versus reaction time. **b** The epimerization reaction with various acids. **c** The proposed mechanism. In red are the formed glycosidic bonds. In blue are the phosphoric acid and the phosphate moiety in product. In pink are the reaction temperatures. In yellow is the gold(I) catalysis. In orange is the curve of α/β ratio versus reaction time.

To probe the role of Ph_3_PAuNTf_2_ in the epimerization reaction, the epimerization process to enrich α-anomer was monitored at elevated temperature (60 °C) by HPLC. As depicted in Fig. 9, the ratio of α/β increased along with the time under the catalysis of Ph_3_PAuNTf_2_ to give the α/β ratio of 12/1 in approximately 3 h (Fig. 9a). Further prolonging the epimerization time can improve the α/β ratio to 18/1 (entry 2, Fig. 9b). Interestingly, in the presence of sterically hindered base (2,6-di-*tert*-butyl-4-methylpyridine) which is supposed to not coordinate the gold(I) catalyst, the epimerization process was substantially impeded to give a ratio of 5.8/1 (entry 3, Fig. 9b). Other Lewis acid promoted epimerization afforded the same α/β selectivity as that when Ph_3_PAuNTf_2_ was utilized (Fig. 9b and Supplementary Fig. 1). On the basis of these results, we speculated the acid was the real specie to trigger the epimerization process, and the gold(I) catalyst probably acted as an acid reservoir which can slowly release acid through possible disproportionation reaction to generate strong acidic Au^3+^ at elevated temperature (Supplementary Fig. 2)^54^. The strong Lewis acid Au^3+^ itself, or the H^+^ which could be generated by reaction of Au^3+^ with trace of H_2_O in the reaction system could promote the epimerization process.

In conclusion, we have developed a highly efficient and stereoselective approach to the synthesis of GPSs by employing gold(I)-catalyzed glycosylation of phosphoric acid acceptors. The efficiency of this protocol was demonstrated by its universal application in preparing more than 45 complex GPSs, one-pot synthesis of linker-tethered GPS from *Leishmania donovani*, and an effective preparation of trisaccharide diphosphate from *Hansenula capsulate* via iterative elongation. Because of its exceptionally broad substrate scope, high α-selectivity and inertness of phosphotriester toward chemical manipulations in comparison to phosphodiester, this strategy will offer new opportunities to create complex phosphosaccharides and install diverse phosphosaccharides on bioactive molecules.

## Supplementary information

supplementary information

## Data Availability

The authors declare that all data supporting the findings of this study are available within the paper and its supplementary information file, including experimental details, characterization data, and NMR spectra of new compounds.
