# Supplementary material for: Stereoselective gold(I)-catalyzed approach to the synthesis of complex α-glycosyl phosphosaccharides
Source: Nat Commun. 2022 Jan 20;13:421. doi: 10.1038/s41467-022-28025-0 (PMC8776814; doi:10.1038/s41467-022-28025-0)

Supplementary Information

Stereoselective Gold(I)-Catalyzed Approach to the Synthesis of Complex α-Glycosyl Phosphosaccharides

Xiaojuan Zhang[a], Yutong Yang[a], Jiahao Ding[a], Yun Zhao[a], Hongbin Zhang[a] and Yugen Zhu*[a]

Key Laboratory of Medicinal Chemistry for Natural Resource, Ministry of Education; Yunnan Provincial Center for Research & Development of Natural Products; School of Chemical Science and Technology, Yunnan University, Kunming, Yunnan 650091, P.R. China. Email: zhuyg86@163.com

Table of Contents

1. **Supplementary Methods**

1.1 General methods--S2

1.2 Mechanistic studies---S3

1.3 Synthesis and characterization

1.3.1 Glycosylation procedure (A) and compounds **3a-3o** and **3p** ------------S4-S14

1.3.2 Hydrogenolysis procedure (B) and **4a-4zg**-------------------------------S14-S36

1.3.3 Procedure (C) for donor synthesis and characterization ----------------S36-S47

1.3.4 Procedure (D) (E) for acceptor synthesis and characterization---------S47-S53

1.3.5 Global deprotection and compounds **5-8**---------------------------------S53-S55

1.3.6 One-pot synthesis and characterization ---------------------------------S56-S61

1.3.7 Synthesis of trisaccharide and characterization --------------------S61-S66

1.3.8 Supplementary Figures of NMR spectra ----------------------------S67-S218

2. **Supplementary References** ---S219-S220

1. Supplementary Methods

1.1 General Methods.

All reactions were carried out at room temperature (RT) under argon atmosphere in glassware with magnetic stirring unless otherwise stated. Unless otherwise stated, all reagents were commercially available and used as received without further purification. Proton nuclear magnetic resonance (^{1}H NMR), carbon nuclear magnetic resonance (^{13}C NMR) and phosphorus nuclear magnetic resonance (^{31}P NMR) spectra were recorded on Brüker DRX 400 spectrometer at 400, 101 and 162 MHz, respectively. Chemical shifts are reported in parts per million (ppm) in $CDCl_3$, D_2O. NMR data are presented as follows: Chemical shift, multiplicity (s = singlet, d = doublet, t = triplet, dd = doublet of doublet, m = multiplet and/or multiple resonances); coupling constant are reported in Hertz (Hz). NMR signals were assigned on the basis of ^{1}H NMR, ^{13}C NMR, COSY and HSQC experiments. High resolution mass spectra were recorded on AB QSTAR Pulsar mass spectrometer. Column chromatography was performed on silica gel G60 (Silicycle, 60-200 µm, 60 Å). TLC analysis was conducted on Silica gel 60 F254 (EMD Chemicals Inc.) with detection by UV light (254 nm) where applicable, and by charring with 10% sulfuric acid in ethanol followed by heating. Molecular sieves were flame-dried under vacuum immediately prior to use.

1.2 Mechanistic studies.

Procedure for the epimerization monitoring by HPLC. To a solution of **3a** (α/β = 3.8/1, 40 mg, 0.05 mmol) and 5Å MS in DCE (1 mL) was added $Ph_3PAuNTf_2$ (3.7 mg, 0.005 mmol) at room temperature. Then the reaction mixture was stirred at 60 °C, and the epimerization started (t = 0). At intervals, small portions of the reaction mixture (~20 µL) were withdrawn via a pipettor and injected into a mixed solvent of CH_3CN (0.7 mL) and Et_3N (~20 µL). After filtration, the solution was then analyzed by HPLC to determine the α/β ratio.

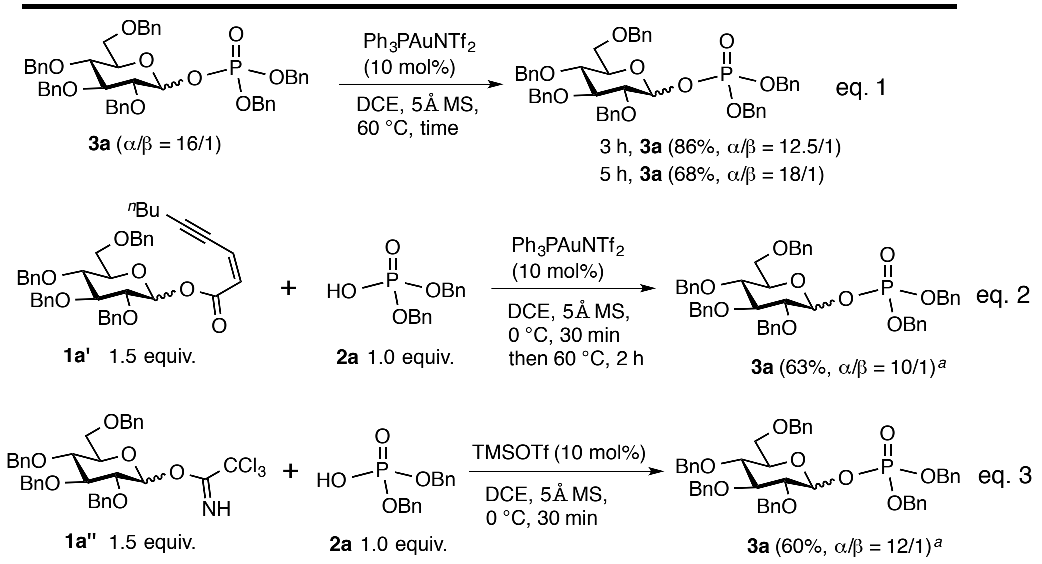

[a]: Yields of α/β mixture were calculated based on donor (1.5 equiv.)

Supplementary Figure 1. The epimerization reaction with 3a (α/β = 16/1) (eq. 1), the glycosylation reaction with other donors (eq. 2 and eq. 3)

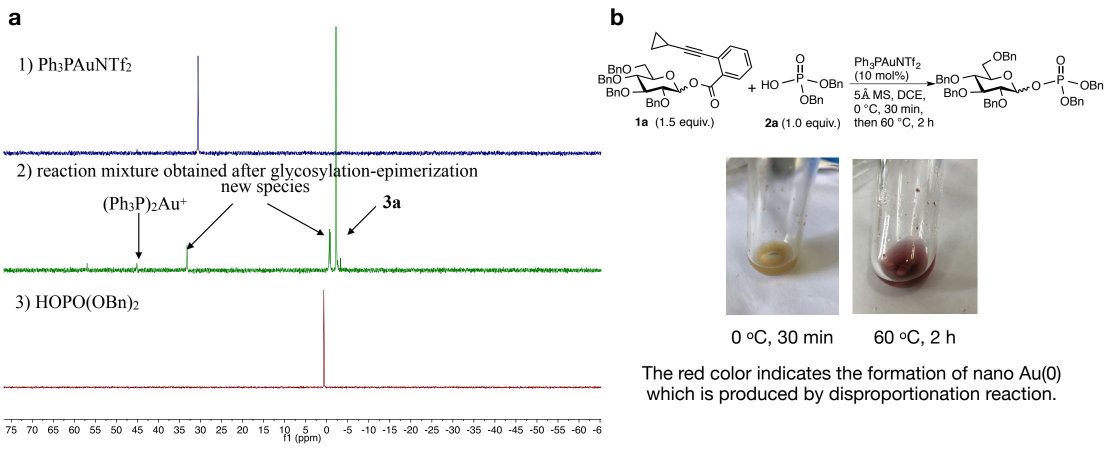

Supplementary Figure 2. Analysis of reaction mixture. a The ^{31}P NMR analysis. **b** The color change of the reaction mixture.

1.3 Synthesis and characterization

1.3.1 Glycosylation procedure (A) and glycosyl phosphates 3a-3o and 3p

General procedure (A) for the gold(I)-catalyzed glycosylation reaction of glycosyl *ortho*-alkynylbenzoate donors and phosphoric acid acceptors. To a solution of donor (0.075 mmol, 1.5 equiv.), acceptor (0.05 mmol, 1.0 equiv.) and 5Å MS in $ClCH_2CH_2Cl$ (1.0 mL) was added $Ph_3PAuNTf_2$ (3.7 mg, 0.005 mmol, 0.1 equiv.) at 0 °C under argon. After the mixture was stirred for 0.5 h at 0 °C, the reaction temperature was elevated to 60 °C when donor **1a, S1 and S3** was used, 100 °C when donor **1d** or **1l** was used, and 25 °C when the other donors were used. After stirring at elevated temperature for 2.0 h, the mixture was cooled down and quenched with Et_3N. Then the mixture was filtrated, and the filtrate was concentrated to give a residue, which was loaded to a silica gel column which was neutralized prior to use by using Hexanes/Et_3N (100/1), then purified to give the α/β mixture of glycosyl phosphosaccharide. Notably, because most of α/β mixture of glycosyl phosphates were not separable (except **3e** and **3i**), the yields of α/β mixture were herein reported. The yields of all the glycosyl phosphates (**3a**-**3o**, **4a**-**4zh**, **9** and **12**) obtained from the present glycosylation approach were calculated based on the donor which was used in excess (1.5 equiv.).

dibenzyl 2,3,4,6-tetra-*O*-benzyl-*α*-D-glucopyranosyl phosphate (3a)

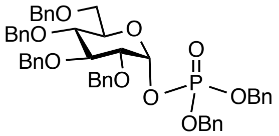

3a, prepared via general procedure (A), eluting with Hexanes/EtOAc/Et_3N (5/1/0.01 to 2/1/0.01), R_f= 0.2 (Hexanes/EtOAc, 2/1), colorless oil, 37.2 mg, 62% yield, α/β = 16/1. $[\alpha]_D^{25}$ = 40.2 (*c* 3.5, $CHCl_3$); ^1H NMR (400 MHz, $CDCl_3$) δ 7.34-7.12 (m, 30H, Ar*H*), 5.96 (dd, *J* = 6.8, 3.2 Hz, 1H, H-1), 5.04 (dd, *J* = 11.9, 7.7 Hz, 4H, CH_2Ph x 2), 4.91 (d, *J* = 10.9 Hz, 1H, C*H*Ph), 4.82 (d, *J* = 10.8 Hz, 1H, C*H*Ph), 4.78 (d, *J* = 11.3 Hz, 2H, C*H*Ph x 2), 4.67 (d, *J* = 11.4 Hz, 1H, C*H*Ph), 4.54 (d, *J* = 12.1 Hz, 1H, C*H*Ph), 4.48 (d, *J* = 10.8 Hz, 1H, C*H*Ph), 4.41 (d, *J* = 12.1 Hz, 1H, C*H*Ph), 3.92 (t, *J* = 9.3 Hz, 2H, H-4, H-5), 3.71 (t, *J* = 9.6 Hz, 1H, H-3), 3.65-3.63 (m, 2H, H-2, H-6), 3.47 (dd, *J* = 10.9, 1.9 Hz, 1H, H-6'); ^{13}C NMR (101 MHz, $CDCl_3$) δ 138.7, 138.2, 137.9, 137.7, 136.0, 135.9, 128.63, 128.58, 128.52, 128.45, 128.3, 128.06, 128.03, 128.00, 127.94, 127.88, 127.85, 127.79, 95.8 (d, *J* = 6.1 Hz), 81.3, 79.4 (d, *J* = 7.4 Hz), 75.8, 75.3, 73.6, 73.1,

72.6, 69.4 (d, *J* = 5.4 Hz), 69.2 (d, *J* = 5.4 Hz), 68.0; ^{31}P NMR (162 MHz, $CDCl_3$) δ -2.2; HRMS(ESI) (m/z): $[M+H^+]^+$calcd. for $C_{48}H_{50}O_9P^+$, 801.3187; found, 801.3188.

dibenzyl 2,3,4-tri-*O*-benzyl-*α*-D-xylopyranosyl phosphate (3b)

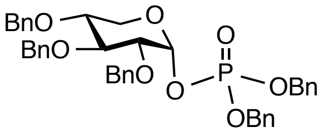

3b, prepared via general procedure (A), eluting with Hexanes/EtOAc/Et_3N (5/1/0.01 to 2/1/0.01), R_f= 0.2 (Hexanes/EtOAc, 2/1), colorless oil, 26.5 mg, 52% yield, α/β = 11/1. $[\alpha]_D^{25}$ = 53.3 (*c* 2.3 $CHCl_3$); ^{1}H NMR (400 MHz, $CDCl_3$) δ 7.33-7.24 (m, 25H, Ar*H*), 5.85 (dd, *J* = 6.8, 3.3 Hz, 1H, H-1), 5.04 (ddd, *J* = 17.7, 7.4, 2.8 Hz, 4H, 4 x C*H*Ph), 4.90-4.81 (m, 2H, 2 x C*H*Ph), 4.72 (dt, *J* = 22.1, 11.2 Hz, 3H, 3 x C*H*Ph), 4.60 (d, *J* = 11.6 Hz, 1H, C*H*Ph), 3.83 (t, *J* = 8.9 Hz, 1H, H-3), 3.72-3.57 (m, 3H, H-4, H-5, H-5'), 3.53 (dt, *J* = 9.5, 3.1 Hz, 1H, H-2); ^{13}C NMR (101 MHz, $CDCl_3$) δ 138.6, 138.1, 137.7, 135.9, 135.84, 135.80, 135.77, 134.3, 134.1, 128.55, 128.49, 128.47, 128.44, 128.41, 128.38, 128.35, 128.1, 128.0, 127.90, 127.87, 127.8, 127.7, 95.8 (d, *J* = 6.1 Hz), 80.5, 79.1 (d, *J* = 7.2 Hz), 77.2, 75.7, 73.6, 73.3, 69.4 (d, *J* = 5.4 Hz), 69.2 (d, *J* = 5.4 Hz), 61.8; ^{31}P NMR (162 MHz, $CDCl_3$) δ -2.2; HRMS(ESI) (m/z): $[M+Na^+]^+$ calcd. for $C_{40}H_{41}NaO_8P^+$, 703.2431; found, 703.2428.

dibenzyl 6-*O*-acetyl-2,3,4-tri-*O*-benzyl-*α*-D-glucopyranosyl phosphate (3c)

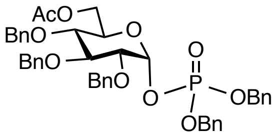

3c, prepared via general procedure (A), eluting with Hexanes/EtOAc/Et_3N (5/1/0.01 to 2/1/0.01), R_f= 0.2 (Hexanes/EtOAc, 5/3), colorless oil, 33.1 mg, 59% yield, α/β = 12/1. $[\alpha]_D^{25}$ = 55.2 (*c* 1.2 $CHCl_3$); ^{1}H NMR (400 MHz, $CDCl_3$) δ 7.33-7.25 (m, 25H, Ar*H*), 5.94 (dd, *J* = 7.0, 3.3 Hz, 1H, H-1), 5.06 (ddd, *J* = 13.7, 7.4, 2.0 Hz, 4H, 2 x CH_2Ph), 4.94 (d, *J* = 10.8 Hz, 1H, C*H*Ph), 4.87 (d, *J* = 10.8 Hz, 1H, C*H*Ph), 4.82-4.75 (m, 2H, 2 x C*H*Ph), 4.67 (d, *J* = 11.3 Hz, 1H, C*H*Ph), 4.55 (d, *J* = 10.7 Hz, 1H, C*H*Ph), 4.19 (dd, *J* = 12.1, 4.4 Hz, 1H, H-6), 4.11 (dd, *J* = 12.2, 2.2 Hz, 1H, H-6'), 4.01-3.86 (m, 2H, H-3, H-5), 3.62 (dt, *J* = 9.6, 3.1 Hz, 1H, H-2), 3.52 (t, *J* = 9.5 Hz, 1H, H-4), 1.92 (s, 3H, CH_3); ^{13}C NMR (101 MHz, $CDCl_3$) δ 170.7, 138.4, 137.8, 137.6, 135.8, 128.73, 128.67, 128.62, 128.58, 128.52, 128.3, 128.2, 128.13, 128.10, 128.07, 128.0, 127.92, 127.86, 81.2, 79.4 (d, J = 7.3 Hz), 76.6, 75.8, 75.3, 73.2, 70.9, 69.5 (d, *J* = 5.4 Hz), 69.3 (d, *J* = 5.4 Hz), 62.7, 20.8; ^{31}P NMR (162 MHz, $CDCl_3$) δ -2.2; HRMS(ESI) (m/z): $[M+Na^+]^+$ calcd. for $C_{43}H_{45}NaO_{10}P^+$, 775.2643; found, 775.2641.

dibenzyl methyl-2,3,4-tri-*O*-benzyl-*α*-D-glucopyranuronatosyl phosphate (3d)

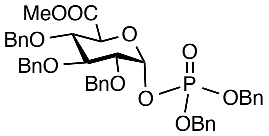

3d, prepared via general procedure (A), eluting with Hexanes/EtOAc/Et_3N (5/1/0.01 to 2/1/0.01), R_f= 0.2 (Hexanes/EtOAc, 5/3), colorless oil, 32.1 mg, 58% yield, α/β = 9.7/1. $[\alpha]_D^{25}$ = 40 (*c* 3.2 $CHCl_3$); ^1H NMR (400 MHz, $CDCl_3$) δ 7.26-7.13 (m, 25H, Ar*H*), 5.86 (dd, *J* = 7.1, 3.2 Hz, 1H, H-1), 5.05-4.91 (m, 4H, 2 x CH_2Ph), 4.82 (d, *J* = 11.0 Hz, 1H, C*H*Ph), 4.76-4.66 (m, 3H, 3 x C*H*Ph), 4.59 (d, *J* = 11.4 Hz, 1H, C*H*Ph), 4.49 (d, *J* = 10.8 Hz, 1H, C*H*Ph), 4.31 (d, *J* = 10.0 Hz, 1H, H-5), 3.84 (t, *J* = 9.3 Hz, 1H, H-3), 3.71 (t, *J* = 9.6 Hz, 1H, H-4), 3.61-3.57 (m, 4H, H-2, OMe); ^{13}C NMR (101 MHz, $CDCl_3$) δ 169.2, 138.4, 137.8, 137.4, 135.83, 135.77, 135.70, 128.65, 128.59, 128.54, 128.52, 128.3, 128.21, 128.05, 128.01, 128.00, 127.93, 127.89, 127.8, 95.5 (d, *J* = 6.1 Hz), 80.6, 78.9, 78.8 (d, *J* = 7.2 Hz), 75.9, 75.4, 73.3, 72.0, 69.6 (d, *J* = 5.5 Hz), 69.5 (d, *J* = 5.5 Hz), 52.7; ^{31}P NMR (162 MHz, $CDCl_3$) δ -2.4; HRMS(ESI) (m/z): $[M+H^+]^+$ calcd. for $C_{42}H_{44}O_{10}P^+$, 739.2667; found, 739.2664.

dibenzyl 2,3,4,6-tetra-*O*-benzyl-*α*-D-galactopyranosyl phosphate (3eα)

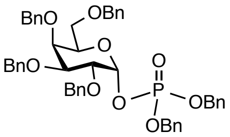

3eα, prepared via general procedure (A), eluting with Hexanes/EtOAc/Et_3N (5/1/0.01 to 2/1/0.01), colorless oil, 38.9 mg, 66% yield, α/β = 11/1. α anomer: R_f = 0.2 (Hexanes/EtOAc, 2/1), $[\alpha]_D^{25}$ = 37 (*c* 0.44, $CHCl_3$); ^1H NMR (400 MHz, $CDCl_3$) δ 7.36-7.20 (m, 30H, Ar*H*), 5.96 (dd, *J* = 6.6, 3.4 Hz, 1H, H-1), 5.07-4.91 (m, 5H, C*H*Ph x 5), 4.81-4.65 (m, 4H, C*H*Ph x 4), 4.56 (d, *J* = 11.3 Hz, 1H, C*H*Ph), 4.35 (q, *J* = 11.7 Hz, 2H, C*H*Ph x 2), 4.16 – 4.06 (m, 2H, H-2, H-3), 3.99 (d, *J* = 2.8 Hz, 1H, H-4), 3.87 (dd, *J* = 10.1, 2.8 Hz, 1H, H-5), 3.52 (dd, *J* = 9.3, 7.1 Hz, 1H, H-6), 3.43 (dd, *J* = 9.3, 5.9 Hz, 1H, H-6'); ^{13}C NMR (101 MHz, $CDCl_3$) δ 138.61, 138.56, 138.2, 137.9, 136.14, 136.06, 136.02, 128.6, 128.5, 128.43, 128.40, 128.35, 128.3, 128.2, 127.95, 127.87, 127.85, 127.8, 127.7, 127.63, 127.57, 96.7 (d, *J* = 6.3 Hz), 78.2, 75.9 (d, *J* =7.0 Hz), 75.0, 74.7, 73.6, 73.4, 73.1, 71.6, 69.3 (d, *J* = 5.3 Hz), 69.1 (d, *J* = 5.2 Hz), 68.6; ^{31}P NMR (162 MHz, $CDCl_3$) δ -2.1. HRMS (m/z): $[M+H^+]^+$calcd. for $C_{48}H_{50}O_9P^+$, 801.3187; found, 801.3188. β anomer: R_f= 0.15 (Hexanes/EtOAc, 2/1), $[\alpha]_D^{25}$ = 27 (*c* 0.44, $CHCl_3$); ^1H NMR (400 MHz, $CDCl_3$) δ 7.31-7.21 (m, 30H, Ar*H*), 5.20 (t, *J* = 7.3 Hz, 1H, H-1, C*H*Ph), 5.09-4.91 (m, 5H, 5 x C*H*Ph), 4.80 (s, 2H, 2 x C*H*Ph), 4.71 (s,

2H, 2 x C*H*Ph), 4.60 (d, *J* = 11.5 Hz, 1H, C*H*Ph), 4.44-4.29 (m, 2H, 2 x C*H*Ph), 4.02-3.88 (m, 2H, H-2, H-4), 3.72-3.64 (m, 1H, H-5), 3.61-3.49 (m, 3H, H-3, H-6, H-6'); ^{13}C NMR (101 MHz, $CDCl_3$) δ 138.7, 138.4, 138.3, 137.8, 128.6, 128.6, 128.4, 128.4, 128.4, 128.3, 128.0, 128.0, 128.0, 127.8, 127.8, 127.7, 127.7, 127.6, 99.51 (d, *J* = 6.4 Hz), 82.1, 79.24 (d, *J* = 9.0 Hz), 75.3, 74.9, 74.3, 73.6, 73.4, 73.2, 69.37 (d, *J* = 4.6 Hz), 69.29, 68.3; ^{31}P NMR (162 MHz, CDCl3) δ -2.3. HRMS (m/z): $[M+H^+]^+$ calcd. for $C_{48}H_{50}O_9P^+$, 801.3187; found, 801.3189.

diphenyl 2,3,4,6-tetra-*O*-benzyl-*α*-D-galactopyranosyl phosphate (3e')

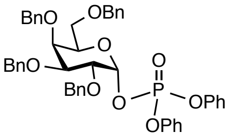

3e', prepared via general procedure (A), eluting with Hexanes/EtOAc/Et_3N (5/1/0.01 to 3/1/0.01), R_f = 0.2 (Hexanes/EtOAc, 3/1), colorless oil, 35.9 mg, 62% yield, α/β = 10/1. $[\alpha]_D^{25}$ = 24.6 (*c* 1.6 $CHCl_3$); ^{1}H NMR (400 MHz, $CDCl_3$) δ 7.34-7.09 (m, 30H, Ar*H*), 6.07 (dd, *J* = 6.2, 3.3 Hz, 1H, H-1), 4.92 (d, *J* = 11.4 Hz, 1H, C*H*Ph), 4.78-4.65 (m, 4H, 4 x C*H*Ph), 4.55 (d, *J* = 11.4 Hz, 1H, C*H*Ph), 4.36 (d, *J* = 1.4 Hz, 2H), 4.13 (dt, *J* = 10.0, 3.3 Hz, 1H, H-2), 4.04-3.95 (m, 2H, H-4, H-5), 3.83 (dd, *J* = 10.0, 2.7 Hz, 1H, H-3), 3.52 (t, *J* = 8.6 Hz, 1H, H-6), 3.29 (dd, *J* = 9.0, 5.2 Hz, 1H, H-6'); ^{13}C NMR (101 MHz, $CDCl_3$) δ 150.73 (d, *J* = 3.6 Hz), 150.65 (d, *J* = 3.6 Hz), 138.6, 138.5, 138.0, 137.8, 129.8, 129.6, 128.5, 128.4, 128.35, 128.26, 128.20, 128.17, 128.00, 127.7, 127.88, 127.85, 127.81, 127.73, 127.67, 127.62, 127.5, 125.32, 125.28, 120.53, 120.48, 120.3, 120.2, 98.1, 78.1, 75.7, 75.0, 74.5, 73.5, 73.4, 73.1, 71.7, 68.0; ^{31}P NMR (162 MHz, $CDCl_3$) δ -13.2; HRMS (m/z): $[M+H^+]^+$ calcd. for $C_{46}H_{46}O_9P^+$, 773.2874; found, 773.2875.

dibenzyl 2,3,4,6-tetra-*O*-benzyl-*α*-D-mannopyranosyl phosphate (3f)

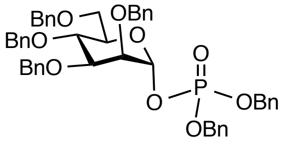

3f, prepared via general procedure (A), eluting with Hexanes/EtOAc/Et_3N (5/1/0.01 to 2/1/0.01), R_f = 0.2 (Hexanes/EtOAc, 2/1), colorless oil, 36.8 mg, 61% yield, α/β > 20/1. $[\alpha]_D^{25}$ = 11.3 (*c* 0.8 $CHCl_3$); ^{1}H NMR (400 MHz, $CDCl_3$) δ 7.34-7.15 (m, 30H, Ar*H*), 5.78 (dd, *J* = 6.2, 2.0 Hz, 1H, H-1), 5.02 (d, *J* = 8.2 Hz, 2H, CH_2Ph), 4.96 (d, *J* = 8.5 Hz, 2H, CH_2Ph), 4.86 (d, *J* = 10.8 Hz, 1H, C*H*Ph), 4.66 (s, 2H, CH_2Ph), 4.60 (d, *J* =

12.1 Hz, 1H, C*H*Ph), 4.55-4.40 (m, 4H, C*H*Ph x 4), 4.04 (t, *J* = 9.7 Hz, 1H, H-4), 3.89 (ddd, *J* = 10.0, 4.6, 1.7 Hz, 1H, H-5), 3.81 (dd, *J* = 9.4, 3.1 Hz, 1H, H-3), 3.73-3.69 (m, 2H, H-2, H-6), 3.56 (dd, *J* = 11.0, 1.8 Hz, 1H, H-6'); ^{13}C NMR (101 MHz, $CDCl_3$) δ 138.5, 138.33, 138.31, 137.9, 135.85, 135.78, 135.71, 128.7, 128.6, 128.5, 128.44, 128.40, 128.1, 128.08, 128.03, 127.99, 127.92, 127.8, 127.6, 96.5 (d, *J* = 6.2 Hz), 79.0, 75.3, 74.5 (d, *J* = 9.5 Hz), 74.2, 74.0, 73.5, 72.9, 72.3, 69.6 (d, *J* = 5.3 Hz), 69.5 (d, *J* = 5.4 Hz), 68.8; ^{31}P NMR (162 MHz, $CDCl_3$) δ -2.7; HRMS (m/z): $[M+Na^+]^+$ calcd. for $C_{48}H_{49}NaO_9P^+$, 823.3006; found, 823.3000.

diphenyl 2,3,4,6-tetra-*O*-benzyl-*α*-D-mannopyranosyl phosphate (3e')

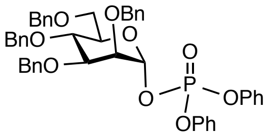

3f', prepared via general procedure (A), eluting with Hexanes/EtOAc/Et_3N (5/1/0.01 to 3/1/0.01), R_f = 0.2 (Hexanes/EtOAc, 3/1), colorless oil, 34.4 mg, 59% yield, α/β > 20/1. $[\alpha]_D^{25}$ = 25.7 (*c* 1.8 $CHCl_3$); ^{1}H NMR (400 MHz, $CDCl_3$) δ 7.33-71.3 (m, 30H, Ar*H*), 6.00 (dd, *J* = 6.0, 2.7 Hz, 1H, H-1), 4.91-4.81 (m, 1H, C*H*Ph), 4.69 (s, 2H, CH_2Ph), 4.62 (d, *J* = 11.8 Hz, 1H, C*H*Ph), 4.55-4.37 (m, 4H, 4 x C*H*Ph), 4.09 (td, *J* = 9.7, 4.9 Hz, 1H, H-4), 3.93-3.79 (m, 2H, H-3, H-5), 3.78-3.67 (m, 2H, H-2, H-6), 3.53 (dd, *J* = 11.4, 2.3 Hz, 1H, H-6'); ^{13}C NMR (101 MHz, $CDCl_3$) δ 150.4, 138.35, 138.30, 138.2, 137.7, 129.91, 129.88, 128.5, 128.42, 128.38, 128.1, 127.92, 127.89, 127.84, 127.78, 127.71, 127.6, 125.6, 125.5, 120.22, 120.17, 97.8, 78.9, 75.3, 74.4, 74.3, 74.2, 74.0, 73.5, 72.9, 72.3, 68.5; ^{31}P NMR (162 MHz, $CDCl_3$) δ -13.9; HRMS (m/z): $[M+H^+]^+$ calcd. for $C_{46}H_{46}O_9P^+$, 773.2874; found, 773.2876.

dibenzyl 3,4-di-*O*-acetyl-2-azido-6-*O*-dibenzyloxyphosphoryl-2-deoxy-*α*-D-mannopyranosyl phosphate (3g)

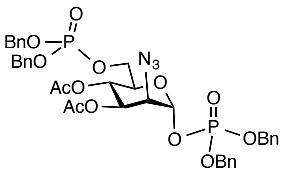

3g, prepared via general procedure (A), eluting with Hexanes/EtOAc/Et_3N (5/1/0.01 to 3/2/0.01), R_f = 0.2 (Hexanes/EtOAc, 3/2), colorless oil, 36.0 mg, 59% yield, α/β > 20/1. $[\alpha]_D^{25}$ = 24.6 (*c* 2.2 $CHCl_3$); ^{1}H NMR (400 MHz, $CDCl_3$) δ 7.35-7.31 (m, 30H, Ar*H*), 5.51 (dd, *J* = 6.6, 1.8 Hz, 1H, H-1), 5.33 (t, *J* = 9.7 Hz, 1H, H-4), 5.27 (dd, *J* = 9.9, 3.6 Hz, 1H, H-3), 5.13-4.97 (m, 8H, 4 x CH_2Ph), 4.00-3.85 (m, 3H, H-5, H-6, H-6'), 3.67 (t, *J* = 2.5 Hz, 1H, H-2), 2.09 (s, 3H, CH_3), 1.98 (s, 3H, CH_3); ^{13}C NMR (101 MHz,

$CDCl_3$) δ 169.8, 169.4, 135.9, 135.8, 135.43, 135.37, 129.0, 128.9, 128.7, 128.6, 128.4, 128.3, 128.1, 128.0, 95.6 (d, J = 5.0 Hz), 70.9 (d, J = 8.4 Hz), 70.21, 70.15 (d, 2^{13}C, J = 6.5 Hz), 69.6 (d, 2^{13}C, J = 5.3 Hz), 65.1, 65.0 (d, J = 4.8 Hz), 61.4 (d, J = 9.9 Hz), 20.7, 20.6; ^{31}P NMR (162 MHz, $CDCl_3$) δ -1.4, -3.2; HRMS (m/z): $[M+H^+]^+$ calcd. for $C_{38}H_{42}N_3O_{13}P_2^+$, 810.2187; found, 810.2183.

dibenzyl 2,3,4-tri-*O*-benzyl-*α*-L-rhamnopyranosyl phosphate (3h)

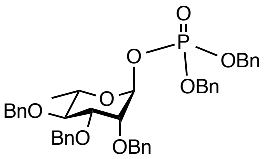

3h, prepared via general procedure (A), eluting with Hexanes/EtOAc/Et_3N (5/1/0.01 to 3/1/0.01), R_f= 0.2 (Hexanes/EtOAc, 3/1), colorless oil, 29.5 mg, 57% yield, α/β > 20/1. $[\alpha]_D^{25}$ = -39.5 (c 1.4 $CHCl_3$); ^{1}H NMR (400 MHz, $CDCl_3$) δ 7.33-7.25 (m, 25H, ArH), 5.66 (dd, J = 6.0, 2.0 Hz, 1H, H-1), 5.01 (d, J = 8.1 Hz, 2H, CH_2Ph), 4.96 (d, J = 8.4 Hz, 2H, CH_2Ph), 4.92 (d, J = 10.9 Hz, 1H CHPh), 4.65 (s, 2H, CH_2Ph), 4.61 (d, J = 10.9 Hz, 1H, CHPh), 4.56-4.43 (m, 2H, CH_2Ph), 3.88-3.79 (m, 1H, H-5), 3.76 (dd, J = 9.4, 3.1 Hz, 1H, H-3), 3.70 (t, J = 2.6 Hz, 1H, H-2), 3.61 (t, J = 9.5 Hz, 1H, H-4), 1.25 (d, J = 6.3 Hz, 3H, CH_3); ^{13}C NMR (101 MHz, $CDCl_3$) δ = 138.5, 138.4, 137.9, 135.81, 135.77, 135.73, 128.7, 128.54, 128.50, 128.1, 128.08, 128.03, 127.82, 127.77, 96.5 (d, J = 6.0 Hz), 79.8, 78.9, 75.5, 74.7 (d, J = 9 Hz), 73.0, 72.3, 70.3, 69.5 (d, 2C, J = 5.4 Hz); ^{31}P NMR (162 MHz, $CDCl_3$) δ = -2.7; HRMS (m/z): $[M+Na^+]^+$ calcd. for $C_{41}H_{43}NaO_8P^+$, 717.2588; found, 717.2588.

dibenzyl 2,3,4-tri-*O*-benzoyl-*α*-L-fucopyranosyl phosphate (3iα)

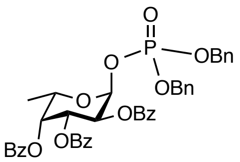

3iα, prepared via general procedure (A), eluting with Hexanes/EtOAc/Et_3N (5/1/0.01 to 2/1/0.01), R_f= 0.2 (Hexanes/EtOAc, 2/1), colorless oil, 31.3 mg, 57% yield, α/β = 9.7/1. $[\alpha]_D^{25}$ = -188 (c 2.7 $CHCl_3$); ^{1}H NMR (400 MHz, $CDCl_3$) δ 8.09-7.23 (m, 25H, ArH), 6.22 (dd, J = 6.2, 3.4 Hz, 1H, H-1), 5.93 (dd, J = 10.8, 3.3 Hz, 1H, H-3), 5.75 (td, J = 5.5, 4.7, 3.2 Hz, 2H, H-2, H-4), 5.14-4.90 (m, 4H, 2 x CH_2Ph), 4.52-4.36 (m, 1H, H-5), 1.18 (d, J = 6.5 Hz, 3H, H-6); ^{13}C NMR (101 MHz, $CDCl_3$) δ = 166.0, 165.9, 165.6, 135.7, 135.6, 135.5, 133.7, 133.5, 133.3, 130.03, 129.96, 129.8, 129.3, 129.2, 129.0, 128.8, 128.73, 128.71, 128.69, 128.6, 128.4, 127.94, 127.91, 95.4, 71.5, 69.7,

69.6, 68.4, 68.3, 67.5, 16.1; ^{31}P NMR (162 MHz, $CDCl_3$) δ = -2.4; HRMS (m/z): $[M+Na^+]^+$ calcd. for $C_{41}H_{37}NaO_{11}P^+$, 759.1966; found, 759.1965.

dibenzyl 2,3,4-tri-*O*-benzoyl-*β*-L-fucopyranosyl phosphate (3iβ)

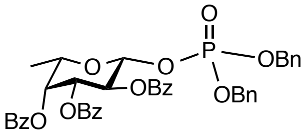

3iβ, prepared via general procedure (A) with additional ^{i}Pr$_2$NEt (0.2 equiv.), eluting with Hexanes/EtOAc/Et_3N (5/1/0.01 to 3/2/0.01), R_f = 0.2 (Hexanes/EtOAc, 3/2), colorless oil, 34.2 mg, 62% yield, α/β = 1/20. $[\alpha]_D^{25}$ = -120 (*c* 3.4 $CHCl_3$); ^{1}H NMR (400 MHz, $CDCl_3$) δ 8.12-7.00 (m, 25H, Ar*H*), 5.89 (dd, *J* = 10.4, 8.0 Hz, 1H, H-2), 5.75 (dd, *J* = 3.5, 1.1 Hz, 1H, H-4), 5.67 (dd, *J* = 8.0, 7.2 Hz, 1H, H-1), 5.57 (dd, *J* = 10.4, 3.4 Hz, 1H, H-3), 5.20-5.04 (m, 2H, 2 x C*H*$_2$Ph), 4.86 (dd, *J* = 11.7, 6.6 Hz, 1H, H-6), 4.77 (dd, *J* = 11.7, 7.1 Hz, 1H, H-6'), 4.21 (qd, *J* = 6.4, 1.2 Hz, 1H, H-5), 1.35 (d, *J* = 6.3 Hz, 1H); ^{13}C NMR (101 MHz, $CDCl_3$) δ = 166.0, 165.6, 165.4, 135.7, 135.6, 135.3, 133.7, 133.6, 133.4, 130.1, 130.0, 129.9, 129.3, 129.1, 128.9, 128.8, 128.62, 128.60, 128.49, 128.46, 128.40, 128.1, 127.6, 97.2 (d, *J* = 4.9 Hz), 71.9 (bs), 71.0, 70.8, 69.9 (d, *J* = 9.2 Hz), 69.8 (d, *J* = 9.2 Hz), 69.5 (d, *J* = 9.2 Hz), 16.3; ^{31}P NMR (162 MHz, $CDCl_3$) δ = -2.9; HRMS (m/z): $[M+Na^+]^+$ calcd. for $C_{41}H_{37}NaO_{11}P^+$, 759.1966; found, 759.1965.

dibenzyl 2-azido-3,4,6-tri-*O*-benzyl-2-deoxy-*α*-D-glucopyranosyl phosphate (3j)

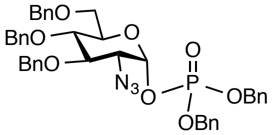

3j, prepared via general procedure (A), eluting with Hexanes/EtOAc/Et_3N (5/1/0.01 to 2/1/0.01), R_f= 0.2 (Hexanes/EtOAc, 2/1), colorless oil, 34.2 mg, 62% yield, α/β = 8.6/1. $[\alpha]_D^{25}$ = 25.8 (*c* 1.4, $CHCl_3$); ^{1}H NMR (400 MHz, $CDCl_3$) δ 7.35-7.14 (m, 30H, Ar*H*), 5.81 (dd, *J* = 6.2, 3.3 Hz, 1H, H-1), 5.17-5.01 (m, 4H, C*H*Ph x 4), 4.87-4.77 (m, 3H, C*H*Ph), 4.55-4.51 (m, 2H, C*H*Ph x 2), 4.40 (d, *J* = 12.0 Hz, 1H, C*H*Ph), 4.00-3.83 (m, 2H, H-3, H-4), 3.78 (t, *J* = 9.5 Hz, 1H, H-5), 3.64 (dd, *J* = 11.0, 3.0 Hz, 1H, H-6'), 3.55 (dt, *J* = 10.1, 3.3 Hz, 1H, H-2), 3.43 (dd, *J* = 11.1, 1.9 Hz, 1H, H-6'); ^{13}C NMR (101 MHz, $CDCl_3$) δ = 138.0, 137.8, 137.7, 135.83, 135.76, 128.71, 128.66, 128.63, 128.59, 128.56, 128.2, 128.12, 128.08, 128.02, 127.99, 127.93, 127.8, 96.4 (d, *J* = 5.7 Hz), 80.1, 75.7, 75.2, 73.6, 73.0, 69.7 (d, *J* = 5.4 Hz), 69.6 (d, *J* = 5.4 Hz), 67.8, 63.6 (d, *J* = 8.7 Hz); ^{31}P NMR (162 MHz, $CDCl_3$) δ = -2.5; HRMS (m/z): $[M+Na^+]^+$ calcd. for $C_{41}H_{42}N_3NaO_8P^+$, 758.2602; found, 758.2604.

dibenzyl 2-azido-3,4,6-tri-*O*-benzyl-2-deoxy-*α*-D-galactopyranosyl phosphate (3k)

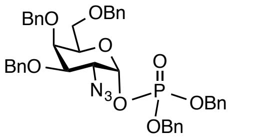

3k, prepared via general procedure (A), eluting with Hexanes/EtOAc/Et_3N (5/1/0.01 to 2/1/0.01), R_f = 0.2 (Hexanes/EtOAc, 2/1), colorless oil, 34.9 mg, 63% yield, α/β = 7.3/1. $[\alpha]_D^{25}$ = 51.6 (*c* 2.0 $CHCl_3$); ^{1}H NMR (400 MHz, $CDCl_3$) δ 7.40-7.21 (m, 25H, Ar*H*), 5.78 (dd, *J* = 6.0, 3.4 Hz, 1H, H-1), 5.13-5.04 (m, 4H, CH_2Ph x 2), 4.86 (d, *J* = 11.2 Hz, 1H, C*H*Ph), 4.71 (d, *J* = 11.4 Hz, 1H, C*H*Ph), 4.65 (d, *J* = 11.3 Hz, 1H, C*H*Ph), 4.51 (d, *J* = 11.2 Hz, 1H, C*H*Ph), 4.38 (d, *J* = 11.7 Hz, 1H, C*H*Ph), 4.32 (d, *J* = 11.7 Hz, 1H, C*H*Ph), 4.10 (dd, *J* = 7.4, 5.9 Hz, 1H, H-5), 4.07-4.04 (m, 2H, H-2, H-4), 3.86 (dd, *J* = 10.5, 2.6 Hz, 1H, H-3), 3.55 (dd, *J* = 9.2, 7.6 Hz, 1H, H-6), 3.40 (dd, *J* = 9.2, 5.7 Hz, 1H, H-6'); ^{13}C NMR (101 MHz, $CDCl_3$) δ = 138.2, 137.8, 137.4, 135.9, 135.8, 128.68, 128.66, 128.62, 128.58, 128.55, 128.46, 128.18, 128.16, 128.07, 127.98, 127.96, 127.92, 96.8 (d, *J* = 6.0 Hz), 75.0, 73.6, 72.9, 72.3, 71.6, 69.6 (d, *J* = 5.3 Hz), 69.4 (d, *J* = 5.3 Hz), 68.2, 59.9 (d, *J* = 8.6 Hz); ^{31}P NMR (162 MHz, $CDCl_3$) δ -2.34; HRMS (m/z): $[M+H^+]^+$calcd. for $C_{41}H_{43}N_3O_8P^+$, 736.2782; found, 736.2778.

dibenzyl 3,4,6-tri-*O*-benzyl-2-fluoro-2-deoxy-*α*-D-glucopyranosyl phosphate (3l)

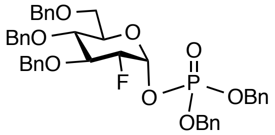

3l, prepared via general procedure (A), eluting with Hexanes/EtOAc/Et_3N (5/1/0.01 to 2/1/0.01), R_f = 0.2 (Hexanes/EtOAc, 2/1), colorless oil, 24.6 mg, 46% yield, α/β = 7.7/1. $[\alpha]_D^{25}$ = 59.5 (*c* 1.4 $CHCl_3$); ^{1}H NMR (400 MHz, $CDCl_3$) δ 7.34-7.13 (m, 25H, Ar*H*), 5.94 (dd, *J* = 6.4, 3.6 Hz, 1H, H-1), 5.09-5.06 (m, 4H, 2 x CH_2Ph), 4.83 (dd, *J* = 11.0, 7.3 Hz, 2H, 2 x C*H*Ph), 4.71 (d, *J* = 11.2 Hz, 1H, C*H*Ph), 4.65-4.48 (m, 3H, 2 x C*H*Ph, H-2), 4.40 (d, *J* = 12.1 Hz, 1H, C*H*Ph), 4.00 (dt, *J* = 12.2, 9.1 Hz, 1H, H-3), 3.91 (ddd, *J* = 10.1, 3.4, 2.0 Hz, 1H, H-5), 3.72 (t, *J* = 9.6 Hz, 1H, H-4), 3.63 (dd, *J* = 11.0, 3.4 Hz, 1H, H-6), 3.46 (dd, *J* = 10.9, 2.0 Hz, 1H, H-6'); ^{13}C NMR (101 MHz, $CDCl_3$) δ = 138.2, 138.1, 137.8, 135.8, 135.7, 128.70, 128.66, 128.6, 128.55, 128.52, 128.13, 128.07, 128.01, 127.95, 127.91, 94.9 (dd, *J* = 5.5 Hz, *J* = 23 Hz), 90.6 (dd, *J* = 8.1 Hz, *J* = 194 Hz), 80.1 (d, *J* = 16.3 Hz), 76.2 (d, *J* = 8.6 Hz), 75.4, 75.2 (d, *J* = 2.3 Hz), 73.6, 72.5, 69.7 (d, *J* = 5.5 Hz), 69.5 (d, *J* = 5.5 Hz), 67.8; ^{31}P NMR (162 MHz, $CDCl_3$) δ = -2.5; ^{19}F NMR (377 MHz, $CDCl_3$) δ = -197.8; HRMS (m/z): $[M+H^+]^+$ calcd. for $C_{41}H_{43}FO_8P^+$, 713.2674; found, 713.2672.

dibenzyl 3,4,6-tri-*O*-benzyl-2-fluoro-2-deoxy-*α*-D-mannopyranosyl phosphate (3m)

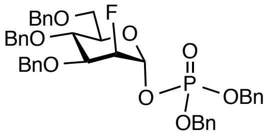

3m, prepared via general procedure (A), eluting with Hexanes/EtOAc/Et_3N (5/1/0.01 to 2/1/0.01), R_f = 0.2 (Hexanes/EtOAc, 2/1), colorless oil, 32.8 mg, 61% yield, α/β = 11/1. $[\alpha]_D^{25}$ = 19.0 (*c* 1.8 $CHCl_3$); ^1H NMR (400 MHz, $CDCl_3$) δ 7.33-7.15 (m, 25H, Ar*H*), 5.79 (td, *J* = 6.1, 2.1 Hz, 1H, H-1), 5.06-4.99 (m, 4H, 2 x CH_2Ph), 4.83 (d, *J* = 10.8 Hz, 1H, C*H*Ph), 4.66-4.47 (m, 5H, 4 x C*H*Ph, H-2), 4.43 (d, *J* = 12.1 Hz, 1H, C*H*Ph), 4.00-3.91 (m, 1H, H-4), 3.87 (ddd, *J* = 10.0, 4.3, 1.6 Hz, 1H, H-5), 3.78 (ddd, *J* = 28.9, 9.4, 2.5 Hz, 1H, H-3), 3.67 (dd, *J* = 11.0, 4.1 Hz, 1H, H-6), 3.51 (dd, *J* = 11.0, 1.8 Hz, 1H, H-6'); ^{13}C NMR (101 MHz, $CDCl_3$) δ = 138.2, 138.1, 137.8, 135.6, 135.5, 134.4, 134.2, 128.9, 128.83, 128.78, 128.6, 128.50, 128.47, 128.2, 128.1, 128.0, 127.9, 127.8, 95.4 (dd, *J* = 5.7 Hz, *J* = 32 Hz), 86.2 (dd, *J* = 11 Hz, *J* = 180 Hz), 77.7, 73.74, 73.70, 73.6, 72.4, 69.9 (d, 2^{13}C, *J* = 5.5 Hz), 68.3; ^{31}P NMR (162 MHz, $CDCl_3$) δ = -2.9; ^{19}F NMR (376 MHz, $CDCl_3$) δ = -203; HRMS (m/z): $[M+H^+]^+$ calcd. for $C_{41}H_{43}FO_8P^+$, 713.2674; found, 713.2672.

dibenzyl 2,3,6-tri-*O*-benzyl-4-fluoro-4-deoxy-*α*-D-galactopyranosyl phosphate (3n)

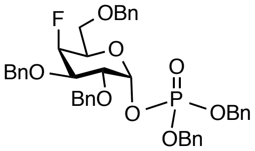

3n, prepared via general procedure (A), eluting with Hexanes/EtOAc/Et_3N (5/1/0.01 to 2/1/0.01), R_f = 0.2 (Hexanes/EtOAc, 2/1), colorless oil, 33.5 mg, 63% yield, α/β = 13/1. $[\alpha]_D^{25}$ = 35.1 (*c* 2.6 $CHCl_3$); ^1H NMR (400 MHz, $CDCl_3$) δ 7.23-7.17 (m, 25H, Ar*H*), 5.86 (dd, *J* = 6.8, 3.3 Hz, 1H, H-1), 5.01-4.86 (m, 4H, CH_2Ph x 2), 4.80 (dd, *J* = 50.0, 2.4 Hz, 1H, H-4), 4.69 (s, 2H, CH_2Ph), 4.66 (s, 2H, CH_2Ph), 4.39 (s, 2H, CH_2Ph), 4.00 (dt, *J* = 29.7, 6.7 Hz, 1H, H-5), 3.90 (dt, *J* = 10.1, 3.2 Hz, 1H, H-2), 3.74 (ddd, *J* = 27.9, 10.0, 2.5 Hz, 1H, H-3), 3.56 (dd, *J* = 9.5, 7.3 Hz, 1H, H-6), 3.42 (ddd, *J* = 9.4, 6.0, 1.4 Hz, 1H, H-6'); ^{13}C NMR (101 MHz, $CDCl_3$) δ = 138.0, 137.9, 137.8, 136.0, 135.94, 135.89, 135.86, 134.4, 134.2, 129.4, 129.3, 128.7, 128.63, 128.57, 128.56, 128.52, 128.46, 128.2, 128.1, 128.0, 127.94, 127.89, 127.87, 127.84, 96.2 (d, *J* = 6.2 Hz), 87.8, 86.0, 75.2, 75.1, 73.75, 73.67, 72.6, 70.3, 69.5 (d, *J* = 5.2 Hz), 69.3 (d, *J* = 5.2 Hz), 67.6

(d, J = 5.7 Hz); ^{31}P NMR (162 MHz, $CDCl_3$) δ = -2.1; ^{19}F NMR (377 MHz, $CDCl_3$) δ = -218; HRMS (m/z): $[M+H^+]^+$calcd. for $C_{41}H_{43}FO_8P^+$, 713.2674; found, 713.2672.

dibenzyl 3,4,6-tri-*O*-benzyl-2-fluoro-2-deoxy-*α*-D-galactopyranosyl phosphate (3o)

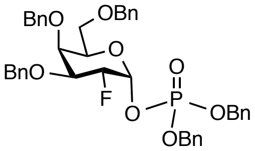

3o, prepared via general procedure (A), eluting with Hexanes/EtOAc/Et_3N (5/1/0.01 to 2/1/0.01), R_f= 0.2 (Hexanes/EtOAc, 2/1), colorless oil, 31.3 mg, 59% yield, α/β = 9/1. $[\alpha]_D^{25}$ = 41.6 (c 3.5 $CHCl_3$); ^{1}H NMR (400 MHz, $CDCl_3$) δ 7.35-7.20 (m, 25H, Ar*H*), 5.94 (dd, J = 6.2, 3.7 Hz, 1H, H-1), 5.07-4.94 (m, 5H, H-2, 2 x C*H*$_2$Ph), 4.97-4.86 (m, 1H, C*H*Ph), 4.76 (d, J = 11.9 Hz, 1H, C*H*Ph), 4.65 (d, J = 11.9 Hz, 1H, C*H*Ph), 4.54 (d, J = 11.2 Hz, 1H, C*H*Ph), 4.40-4.31 (m, 2H, C*H*$_2$Ph), 4.11 (t, J = 6.6 Hz, 1H, H-5), 4.01 (bs, 1H, H-4), 3.94 (td, J = 10.0, 2.8 Hz, 1H, H-3), 3.53 (dd, J = 9.3, 7.3 Hz, 1H, H-6), 3.42 (dd, J = 9.2, 5.8 Hz, 1H, H-6'); ^{13}C NMR (101 MHz, $CDCl_3$) δ = 138.2, 138.0, 137.7, 135.8, 135.7, 128.6, 128.52, 128.49, 128.43, 128.3, 128.2, 128.0, 127.9, 127.84, 127.82, 127.80, 127.5, 95.4 (dd, J = 6.2 Hz, J = 23 Hz,), 88.4 (dd, J = 7.8 Hz, J = 191 Hz), 76.3 (d, J = 16 Hz), 75.1, 75.0, 73.5, 73.0, 71.5, 69.5 (d, J = 5.3 Hz), 69.3 (d, J = 5.3 Hz), 68.1; ^{31}P NMR (162 MHz, $CDCl_3$) δ = -2.4; ^{19}F NMR (376 MHz, $CDCl_3$) δ = -206.7; HRMS (m/z): $[M+H^+]^+$ calcd. for $C_{41}H_{43}FO_8P^+$, 713.2674; found, 713.2670.

(2*R*,3*R*,4*R*,4a*R*,10b*S*)-3,4-bis(benzyloxy)-2-((benzyloxy)methyl)-2,3,4,4a,6,10b-hexahydropyrano[3,2-*c*] isochromene (3p)

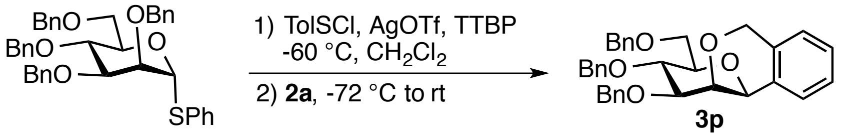

To a mixture of phenyl 2,3,4,6-tetra-*O*-benzyl-1-thio-mannoside (38 mg, 0.06 mmol), TTBP (18.6 mg, 0.075 mmol) and freshly activated 4 Å molecular sieves (50 mg) in anhydrous CH_2Cl_2 (2 mL) under argon atmosphere was added *p*-TolSCl (7.9 μL, 0.06 mmol) and AgOTf (15 mg, 0.06 mmol) at -60 °C. After stirring 5 min, **2a** (14 mg, 0.05 mmol) was added in one portion at -60 °C. The resulting mixture was slowly warmed to room temperature within 2 h, and then quenched with Et_3N (1 mL) and filtered through Celite. After concentration, the residue was purified through flash silica gel column chromatography (Hexanes/EtOAc, 10/1) to give compound **3p** as colorless oil (22.6 mg, 72% yield) based on the donor. R_f= 0.2 (Hexanes/EtOAc, 7/1), $[\alpha]_D^{25}$ = 31.9

(c 1.6 $CHCl_3$); ^{1}H NMR (400 MHz, $CDCl_3$) δ 7.28-7.12 (m, 20H, Ar*H*), 5.12 (d, *J* = 1.8 Hz, 1H, H-1), 4.81 (d, *J* = 10.6 Hz, 1H, C*H*Ph), 4.59-4.43 (m, 3H, 3 x C*H*Ph), 3.90 (t, *J* = 9.6 Hz, 1H, H-4), 3.66-3.62 (m, 2H, H-3, H-6), 3.57-3.49 (m, 2H, H-2, H-6'), 3.48 (dd, *J* = 10.1, 4.7 Hz, 1H, H-5); ^{13}C NMR (101 MHz, $CDCl_3$) δ = 138.5, 138.47, 138.4, 138.1, 128.6, 128.52, 128.47, 128.2, 128.1, 127.92, 127.89, 127.86, 127.82, 127.80, 127.7, 93.5, 79.6, 75.4, 74.8, 74.2, 73.6, 72.8, 72.6, 72.2, 69.2; HRMS (m/z): $[M+H^+]^+$ calcd. for $C_{34}H_{35}O_5^+$, 523.2479; found, 523.2478.

1.3.2 Hydrogenolysis procedure (B) and phosphosaccharides 4a-4zg

To clearly characterize the products of bis-glycosyl benzyl phosphotriester by using NMR analysis, the chirality of phosphorus atom was eliminated by removing the benzyl group on phosphorus. The deprotection reaction was straightforward to perform, in quantitative yield and free of purification. Notably, the azido groups of **4t**-**4z**, **4za**, **4e**, **4j** and **4o** were simultaneously reduced to amino groups.

General procedure (B) for removal of benzyl group of benzyl phosphate. To a solution of phosphotriester (40 mg) in EtOAc/MeOH (2.0 mL/2.0 mL) was added Et_3N (40 μL) and Pd/C (40 mg). Then, the mixture was stirred under an atmosphere of H_2 at room temperature for 0.5 h until TLC showed the completion of hydrogenolysis. After filtration through syringe filter, the filtrate was concentrated to afford a colorless oil, which was tested via NMR analysis to determine the structure and α/β ratio (by ^{31}P NMR). Notably, because the diastereomeric anomers with amino group on sugar ring (**4t-4z** and **4za**) do not show separated ^{31}P signals, the α/β ratio was determined before hydrogenolysis by using ^{31}P NMR spectra of phosphotriester.

methyl 2,3,4,6-tetra-*O*-benzyl-*α*-D-glucopyranosyl-phosphoryl-(→6)-2,3,4-tri-*O*-benzoyl-*α*-D-glucopyranoside triethylammonium salt (4a)

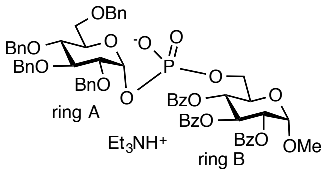

4a, prepared first via procedure (A), purified via flash chromatography by eluting with Hexanes/EtOAc/Et_3N (5/1/0.01 to 2/1/0.01) to give an intermediate product (R_f = 0.2 (Hexanes/EtOAc, 2/1), 53.3 mg, 59% yield); then procedure (B), quantitative yield, colorless oil, α/β = 20/1. $[\alpha]_D^{25}$ = 54.3 (c 2.7 $CHCl_3$); ^{1}H NMR (400 MHz, $CDCl_3$) δ 7.91-7.08 (m, 35H, Ar*H*), 6.02 (t, *J* = 9.7 Hz, 1H, H-3^A), 5.80 (dd, *J* = 8.2, 3.2 Hz, 1H, H-1^B), 5.39 (t, *J* = 9.8 Hz, 1H, H-4^A), 5.12-5.02 (m, 2H, H-1^A, H-2^A), 4.85 (d, *J* = 10.9

Hz, 1H, C*H*Ph), 4.80 (d, *J* = 11.8 Hz, 1H, C*H*Ph), 4.75 (d, *J* = 11.1 Hz, 1H, C*H*Ph), 4.67 (d, *J* = 10.9 Hz, 1H, C*H*Ph), 4.56 (d, *J* = 11.8 Hz, 1H, C*H*Ph), 4.43 (t, *J* = 12.5 Hz, 2H, C*H*Ph x 2), 4.31 (d, *J* = 12.2 Hz, 1H, C*H*Ph), 4.19 (ddd, *J* = 9.6, 6.8, 1.8 Hz, 1H, H-5^A), 4.14-4.07 (m, 1H, H-6^A), 4.06 – 3.86 (m, 3H, H-5^B, H-6'^{A}, H-3^B), 3.63 (t, *J* = 9.6 Hz, 1H, H-4^B), 3.57 (dd, *J* = 10.7, 2.9 Hz, 1H, H-6^B), 3.50 (d, *J* = 11.5 Hz, 2H, H-2^B, H-6'^{B}), 3.34 (s, 3H, CH_3); ^{13}C NMR (101 MHz, $CDCl_3$) δ = 166.0, 165.9, 165.3, 139.0, 138.9, 138.8, 138.3, 133.4, 133.2, 133.1, 130.03, 129.96, 129.8, 129.5, 129.33, 129.30, 128.5, 128.4, 128.3, 128.1, 127.87, 127.85, 127.7, 127.6, 127.5, 96.6, 93.1 (d, *J* = 6.1 Hz), 81.6, 80.0 (d, *J* = 7.5 Hz), 75.6, 74.8, 73.4, 72.3, 72.1, 71.3, 71.0, 69.5, 69.1 (d, *J* = 8.4 Hz), 68.5, 64.3 (bs), 55.6; ^{31}P NMR (162 MHz, $CDCl_3$) δ = -1.6; HRMS (m/z): [M-Et_3NH^++Na^++H^+]$^+$ calcd. for $C_{62}H_{61}NaO_{17}P^+$, 1131.3539; found, 1131.3533.

2,3,4,6-tetra-*O*-benzyl-*α*-D-glucopyranosyl-phosphoryl-(→6)-1,2:3,4-di-*O*-isopropylidene-*α*-D-galactopyranose triethylammonium salt (4b)

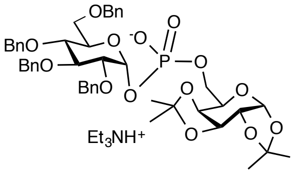

4b, prepared first via procedure (A), purified via flash chromatography by eluting with Hexanes/EtOAc/Et_3N (5/1/0.01 to 2/1/0.01) to give an intermediate product (R_f = 0.2 (Hexanes/EtOAc, 2/1), 41 mg, 57% yield); then procedure (B), quantitative yield, colorless oil, α/β = 12/1. $[\alpha]_D^{25}$ = 17.1 (*c* 3.0 $CHCl_3$); ^{1}H NMR (400 MHz, $CDCl_3$) δ 7.34-7.08 (m, 20H, Ar*H*), 5.83 (dd, *J* = 8.1, 3.3 Hz, 1H, H-1^B), 5.41 (d, *J* = 5.0 Hz, 1H, H-1^A), 4.88 (d, *J* = 10.9 Hz, 1H, C*H*Ph), 4.83 (d, *J* = 11.8 Hz, 1H, C*H*Ph), 4.77 (d, *J* = 11.1 Hz, 1H, C*H*Ph), 4.70 (d, *J* = 10.9 Hz, 1H, C*H*Ph), 4.58-4.52 (m, 2H, C*H*Ph x 2), 4.45-4.43 (m, 2H, C*H*Ph, H-3^A), 4.37 (d, *J* = 12.2 Hz, 1H, C*H*Ph), 4.19-4.15 (m, 2H, H-2^A, H-4^A), 4.09-3.92 (m, 5H, H-3^B, H-5^B, H-5^A, H-6^A, H-6' ^{A}), 3.71-3.63 (m, 2H, H-4^B, H-6^B), 3.60 (dd, *J* = 10.9, 2.0 Hz, 1H, H-6'^{B}), 3.53 (dt, *J* = 9.7, 2.9 Hz, 1H, H-2^B), 1.42 (s, 3H, CH_3), 1.30 (s, 3H, CH_3), 1.21 (s, 3H, CH_3), 1.16 (s, 3H, CH_3); ^{13}C NMR (101 MHz, $CDCl_3$) δ = 139.1, 138.9, 138.7, 138.4, 128.5, 128.4, 128.3, 128.1, 128.04, 128.00, 127.65, 127.61, 127.53, 127.46, 109.1, 108.5, 96.4, 93.1, 81.8, 80.1, 75.6, 74.8, 73.5, 72.0, 71.3, 71.0, 68.7; HRMS (m/z): [M-Et_3NH^++Na^++H^+]$^+$ calcd. for $C_{46}H_{55}NaO_{14}P^+$, 885.3222; found, 885.3226.

methyl 2,3,4,6-tetra-*O*-benzyl-*α*-D-glucopyranosyl-phosphoryl-(→4)-2,3,6-tri-*O*-benzyl-*α*-D-glucopyranoside triethylammonium salt (4c)

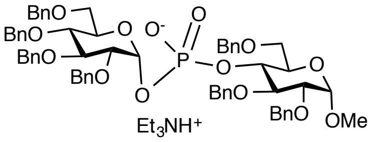

4c, prepared first via procedure (A), purified by flash chromatography by eluting with Hexanes/EtOAc/Et_3N (5/1/0.01 to 2/1/0.01) to give an intermediate product (R_f = 0.2 (Hexanes/EtOAc, 2/1), 48.6 mg, 56% yield); then procedure (B), quantitative yield, colorless oil, α/β = 13/1. $[\alpha]_D^{25}$ = 48.6 (*c* 2.2 $CHCl_3$); ^{1}H NMR (400 MHz, $CDCl_3$) δ 7.43-7.06 (m, 35H, Ar*H*), 5.86 (dd, *J* = 7.9, 3.2 Hz, 1H, H-1^B), 5.13 (d, *J* = 11.3 Hz, 1H, C*H*Ph), 4.75 (t, *J* = 12.1 Hz, 4H), 4.63 (d, *J* = 12.2 Hz, 1H, C*H*Ph), 4.58 (d, *J* = 12.1 Hz, 1H, C*H*Ph), 4.52-4.42 (m, 8H, C*H*Ph x 7, H-1^A), 4.34 (d, *J* = 12.0 Hz, 1H, C*H*Ph), 4.31-4.21 (m, 1H, H-4^A), 4.11-3.99 (m, 2H, H-6^A, H-5^B), 3.93-3.72 (m, 4H, H-3^B, H-3^A, H-5^A, H-6'^{A}), 3.67-3.50 (m, 4H, H-2^B, H-4^B, H-6^B, H-6'^{B}), 3.38 (dd, *J* = 9.7, 3.7 Hz, 1H, H-2^A), 3.27 (s, 3H, C*H*$_3$); ^{13}C NMR (101 MHz, $CDCl_3$) δ = 140.3, 139.4, 139.1, 138.9, 138.7, 138.5, 138.3, 128.41, 128.37, 128.3, 128.2, 128.12, 128.9, 128.05, 128.00, 129.97, 127.9, 127.83, 127.78, 127.73, 127.66, 127.52, 127.49, 127.42, 127.0, 126.9, 98.0, 93.3 (d, *J* = 6.0 Hz), 81.9, 81.5 (d, *J* = 2.9 Hz), 80.5 (d, *J* = 8.0 Hz), 79.4, 77.8, 75.5, 75.2, 74.9, 74.7 (d, *J* = 6.4 Hz), 73.54, 73.51, 72.3, 71.3, 70.6 (d, *J* = 3.8 Hz), 70.0, 69.0, 55.2; ^{31}P NMR (162 MHz, $CDCl_3$) δ = -2.5; HRMS (m/z): [M-Et_3NH^++Na^++H^+]$^+$ calcd. for $C_{62}H_{67}NaO_{14}P^+$, 1089.4161; found, 1089.4163.

methyl 2,3,4,6-tetra-*O*-benzyl-*α*-D-glucopyranosyl-phosphoryl-(→4)-2,3,6-tri-*O*-benzoyl-*α*-D-galactopyranoside triethylammonium salt (4d)

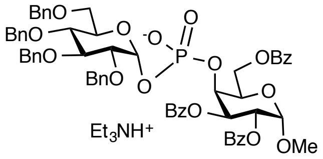

4d, prepared via first procedure (A), purified by flash chromatography by eluting with Hexanes/EtOAc/Et_3N (5/1/0.01 to 2/1/0.01) to give an intermediate product (R_f = 0.2 (Hexanes/EtOAc, 2/1), 52.7 mg, 59% yield); then procedure (B), quantitative yield, colorless oil, α/β = 13/1. $[\alpha]_D^{25}$ = 96.8 (*c* 2.4 $CHCl_3$); ^{1}H NMR (400 MHz, $CDCl_3$) δ 8.08-7.11 (m, 35H, Ar*H*), 5.78 (dd, *J* = 7.7, 3.4 Hz, 1H, H-1^B), 5.60 (d, *J* = 2.0 Hz, 2H, H-2^A, H-3^A), 5.11 (d, *J* = 2.3 Hz, 1H, H-1^A), 4.97 (d, *J* = 10.3 Hz, 1H, H-4^A), 4.84 (d, *J* = 11.1 Hz, 1H, C*H*Ph), 4.76 (d, *J* = 11.0 Hz, 2H, C*H*Ph, H-6^A), 4.72-4.57 (m, 3H, H-6'^{A}, 2 x C*H*Ph), 4.51-4.44 (m, 3H, 3 x C*H*Ph), 4.34 (d, *J* = 12.0 Hz, 1H, C*H*Ph), 4.09 (dt, *J* = 10.2, 2.4 Hz, 1H, H-5^B), 3.97 (t, *J* = 9.3 Hz, 1H, H-3^B), 3.94-3.87 (m, 1H, H-5^A), 3.74 (dd, *J* = 11.0, 3.2 Hz, 1H, H-6^B), 3.69-3.57 (m, 2H, H-4^B, H-6'^{B}), 3.45 (dt, *J*

= 9.6, 3.0 Hz, 1H, H-2^B), 3.29 (s, 3H, OMe); ^{13}C NMR (101 MHz, CDCl$_3$) δ = 166.5, 166.3, 166.2, 139.3, 139.1, 138.6, 133.1, 132.9, 132.6, 130.9, 130.5, 130.3, 130.0, 129.82, 129.78, 128.5, 128.42, 128.37, 128.32, 128.29, 128.22, 127.93, 127.89, 127.7, 127.50, 127.45, 127.39, 97.3, 93.6 (d, *J* = 5.9 Hz), 81.9, 80.3 (d, J = 7.7 Hz), 75.5, 74.7, 73.4, 72.5, 71.7 (d, J = 5.4 Hz), 71.4, 69.8, 69.7 (d, *J* = 2.3 Hz), 69.0, 68.6 (d, *J* = 3.6 Hz), 65.3, 55.1; ^{31}P NMR (162 MHz, CDCl$_3$) δ = -1.4; HRMS (m/z): [M-Et$_3$NH$^+$+Na$^+$+H$^+$]$^+$ calcd. for $C_{62}H_{61}NaO_{17}P^+$, 1131.3539; found, 1131.3537.

***tert*-butyldimethylsilyl 2,3,4,6-tetra-*O*-benzyl-*α*-D-glucopyranosyl-phosphoryl-(→3)-2-amino-4,6-*O*-benzylidene-2-deoxy-*β*-D-glucopyranoside triethylammonium salt (4e)**

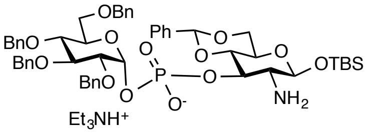

4e, prepared first via procedure (A), purified by flash chromatography by eluting with Hexanes/EtOAc/Et$_3$N (5/1/0.01) to give an intermediate product (R*f* = 0.2 (Hexanes/EtOAc, 4/1), 44 mg, 53% yield); then procedure (B), quantitative yield, colorless oil, α/β = 17/1. $[\alpha]_D^{25}$ = 25.4 (*c* 1.9 CHCl$_3$); ^{1}H NMR (400 MHz, CDCl$_3$) δ 7.42-7.04 (m, 25H, Ar*H*), 5.83 (dd, *J* = 8.0, 3.3 Hz, 1H, H-1^B), 5.30 (s, 1H, C*H*Ph), 4.74-4.69 (m, 3H, 3 x C*H*Ph), 4.54-4.33 (m, 6H, H-1^A, 5 x C*H*Ph), 4.03 (d, *J* = 10.2 Hz, 2H, H-5^B, H-6^A), 3.87 (t, *J* = 9.3 Hz, 1H, H-3^B), 3.69-3.39 (m, 7H, H-2^B, H-3^A, H-4^A, H-4^B,H-6^B, H-6'^{B}, H-6'^{A}), 3.27 (t, *J* = 8.4 Hz, 1H, H-5^A), 2.74 (t, *J* = 8.6 Hz, 1H, H-2^A), 0.78 (s, 9H), 0.01 (s, 3H), 0.00 (s, 2H); ^{13}C NMR (101 MHz, CDCl$_3$) δ = 139.1, 138.8, 138.5, 138.2, 137.9, 129.4, 128.8, 128.5, 128.45, 128.39, 128.33, 128.28, 128.2, 128.04, 128.01, 127.98, 127.91, 127.8, 127.73, 127.66, 127.5, 126.7, 119.1, 116.0, 101.6, 93.4 (bs), 81.7, 80.5, 80.2, 77.7, 75.5, 74.8, 73.5, 72.1, 71.3, 69.0, 68.7, 66.6, 60.0, 8.5, -4.0, -4.8; ^{31}P NMR (162 MHz, CDCl$_3$) δ = -1.9; HRMS (m/z): [M-Et$_3$NH$^+$+2H$^+$]$^+$ calcd. for $C_{53}H_{67}NO_{13}PSi^+$, 984.4114; found, 984.4111.

methyl 2,3,4,6-tetra-*O*-benzyl-*α*-D-galactopyranosyl-phosphoryl-(→6)-2,3,4-tri-*O*-benzoyl-*α*-D-glucopyranoside triethylammonium salt (4f)

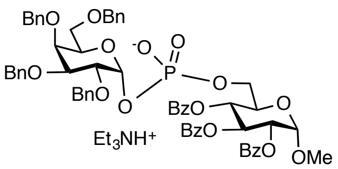

4f, prepared first via general procedure (A), purified by flash chromatography by eluting with Hexanes/EtOAc/Et$_3$N (5/1/0.01 to 3/1/0.01) to give an intermediate

product (R_f = 0.2 (Hexanes/EtOAc, 2/1), 57.5 mg, 64% yield); then procedure (B), quantitative yield, colorless oil, α/β = 14/1. $[\alpha]_D^{25}$ = 48.0 (*c* 4.5 $CHCl_3$); ^{1}H NMR (400 MHz, $CDCl_3$) δ 7.90-71.6 (m, 35H, Ar*H*), 6.02 (t, *J* = 9.8 Hz, 1H, H-3^A), 5.76 (dd, *J* = 8.0, 3.3 Hz, 1H, H-1^B), 5.37 (t, *J* = 9.8 Hz, 1H, H-4^A), 5.10 (dd, *J* = 10.1, 3.6 Hz, 1H, H-2^A), 5.05 (d, *J* = 3.7 Hz, 1H, H-1^A), 4.84 (d, *J* = 11.4 Hz, 1H, C*H*Ph), 4.76 (d, *J* = 11.8 Hz, 1H, C*H*Ph), 4.68 (d, *J* = 11.9 Hz, 1H, C*H*Ph), 4.59 (dd, *J* = 11.9, 6.3 Hz, 2H, C*H*Ph x 2), 4.47 (d, *J* = 11.4 Hz, 1H, C*H*Ph), 4.41 (d, *J* = 11.7 Hz, 1H, C*H*Ph), 4.33 (d, *J* = 11.7 Hz, 1H, C*H*Ph), 4.19-4.07 (m, 3H, H-5^B, H-5^A, H-6^A), 4.03-3.99 (m, 1H, H-6'^{A}), 3.97-3.94 (m, 1H, H-2^B), 3.87-3.85 (m, 2H, H-3^B, H-4^B), 3.47 (d, *J* = 6.7 Hz, 2H, H-6^B, H-6'^{B}), 3.32 (s, 3H, CH_3); ^{13}C NMR (101 MHz, $CDCl_3$) δ = 165.9, 165.8, 165.2, 139.2, 139.1, 139.0, 138.3, 133.3, 133.1, 129.98, 129.94, 129.7, 129.5, 129.4, 129.3, 128.44, 128.39, 128.34, 128.31, 128.27, 128.22, 128.16, 128.0, 127.8, 127.7, 127.5, 127.44, 127.39, 127.2, 96.6, 93.9 (d, *J* = 6.3 Hz), 78.6, 76.6 (d, *J* = 7.2 Hz), 75.3, 74.9, 73.4, 73.0, 72.34, 72.28, 71.1, 69.9, 69.6, 69.2 (d, *J* = 8.6 Hz), 68.9, 64.3 (d, *J* = 5.3 Hz), 55.5; ^{31}P NMR (162 MHz, $CDCl_3$) δ = -1.5; HRMS (m/z): $[M-Et_3NH^++2H^+]^+$ calcd. for $C_{62}H_{62}O_{17}P^+$, 1109.3719; found, 1109.3724.

2,3,4,6-tetra-*O*-benzyl-*α*-D-galactopyranosyl-phosphoryl-(→6)-1,2:3,4-di-*O*-isopropylidene-*α*-D-galactopyranose triethylammonium salt (4g)

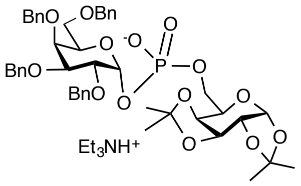

4g, prepared first via general procedure (A), purified by flash chromatography by eluting with Hexanes/EtOAc/Et_3N (5/1/0.01 to 3/1/0.01) to give an intermediate product (R_f = 0.2 (Hexanes/EtOAc, 2/1), 45.7 mg, 64% yield); then procedure (B), quantitative yield, colorless oil, α/β > 20/1. $[\alpha]_D^{25}$ = 10.8 (*c* 1.7 $CHCl_3$); ^{1}H NMR (400 MHz, $CDCl_3$) δ 7.35-7.14 (m, 20H, Ar*H*), 5.77 (dd, *J* = 7.8, 3.2 Hz, 1H, H-1^B), 5.41 (d, *J* = 5.0 Hz, 1H, H-1^A), 4.85 (d, *J* = 11.4 Hz, 1H, C*H*Ph), 4.81 (d, *J* = 11.9 Hz, 1H, C*H*Ph), 4.74 (d, *J* = 11.9 Hz, 1H, C*H*Ph), 4.64 (d, *J* = 12.0 Hz, 1H, C*H*Ph), 4.61 (d, *J* = 11.9 Hz, 1H, C*H*Ph), 4.49 (d, *J* = 11.4 Hz, 1H, C*H*Ph), 4.45-4.37 (m, 2H, C*H*Ph), 4.32 (d, *J* = 11.8 Hz, 1H, C*H*Ph), 4.25-3.90 (m, 10H, H-2^A, H-3^A, H-4^A,H-5^A, H-6^A, H-6'^{A}, H-2^B, H-3^B, H-4^B, H-5^B), 3.55-3.46 (m, 2H, H-6^B, H-6'^{B}), 1.42 (s, 3H, CH_3), 1.30 (s, 3H, CH_3), 1.21 (s, 3H, CH_3), 1.17 (s, 3H, CH_3); ^{13}C NMR (101 MHz, $CDCl_3$) δ = 139.2, 139.14, 139.05, 138.4, 128.5, 128.4, 128.29, 128.27, 128.21, 128.0, 127.9, 127.7,

127.52, 127.50, 127.4, 127.3, 109.1, 108.5, 96.5, 93.9 (d, *J* = 6.4 Hz), 78.7, 76.53, 76.45, 75.3, 75.0, 73.4, 73.0, 72.3, 71.0, 70.8 (d, *J* = 1.8 Hz), 69.9, 68.9, 67.9 (d, *J* = 8.2 Hz), 64.6 (d, *J* = 5.2 Hz), 26.3, 26.2, 25.2, 24.5; ^{31}P NMR (162 MHz, $CDCl_3$) δ = -1.2; HRMS (m/z): $[M-Et_3NH^++2H^+]^+$ calcd. for $C_{46}H_{56}O_{14}P^+$, 863.3402; found, 863.3404.

methyl 2,3,4,6-tetra-*O*-benzyl-*α*-D-galactopyranosyl-phosphoryl-(→4)-2,3,6-tri-*O*-benzyl-*α*-D-glucopyranoside triethylammonium salt (4h)

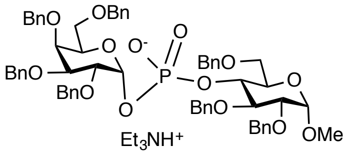

4h, prepared first via general procedure (A), purified by flash chromatography by eluting with Hexanes/EtOAc/Et_3N (5/1/0.01 to 3/1/0.01) to give an intermediate product (R_f = 0.2 (Hexanes/EtOAc, 3/1), 53.8 mg, 62% yield); then procedure (B), quantitative yield, colorless oil, α/β > 20/1. $[\alpha]_D^{25}$ = 46.8 (*c* 2.5 $CHCl_3$); ^{1}H NMR (400 MHz, $CDCl_3$) δ 7.43-7.10 (m, 35H, Ar*H*), 5.85 (dd, *J* = 8.0, 3.2 Hz, 1H, H-1^B), 5.08 (d, *J* = 10.9 Hz, 1H, C*H*Ph), 4.83-4.72 (m, 3H, 3 x C*H*Ph), 4.64 (d, *J* = 12.2 Hz, 1H, C*H*Ph), 4.58-4.39 (m, 8H, H-1^A, 7 x C*H*Ph), 4.33 (td, *J* = 13.3, 12.6, 7.6 Hz, 4H, 3 x C*H*Ph, H-4^A), 4.15 (t, *J* = 6.5 Hz, 1H, H-5^B), 4.05 (d, *J* = 10.5 Hz, 1H, H-6^B), 3.93 (dt, *J* = 10.0, 2.9 Hz, 1H, H-2^B), 3.86 (t, *J* = 9.3 Hz, 1H, H-3^A), 3.78 (dd, *J* = 10.1, 2.7 Hz, 1H, H-3^B), 3.76-3.62 (m, 3H, H-4^B, H-6'^{B}, H-5^A), 3.49-3.29 (m, 3H, H-2^A, H-6^A, H-6'^{A}), 3.26 (s, 3H, OMe); ^{13}C NMR (101 MHz, $CDCl_3$) δ = 140.2, 139.4, 139.3, 139.0, 138.9, 138.6, 138.3, 128.5, 128.4, 128.33, 128.29, 128.6, 128.22, 128.15, 128.1, 128.05, 127.98, 127.8, 127.6, 127.5, 127.4, 127.31, 127.26, 127.1, 127.0, 98.0, 94.2 (d, *J* = 6.1 Hz), 81.5 (d, *J* = 3.1 Hz), 79.5, 78.7, 75.4, 75.3, 74.9, 74.6 (d, *J* = 6.4 Hz), 73.6, 73.4, 73.2, 73.1, 72.5, 70.7 (d, *J* = 3.4 Hz), 70.2, 70.0, 69.4, 55.1; ^{31}P NMR (162 MHz, $CDCl_3$) δ = -2.4; HRMS (m/z): $[M-Et_3NH^++2H^+]^+$ calcd. for $C_{62}H_{68}O_{14}P^+$, 1067.4341; found, 1067.4341.

methyl 2,3,4,6-tetra-*O*-benzyl-*α*-D-galactopyranosyl-phosphoryl-(→4)-2,3,6-tri-*O*-benzoyl-*α*-D-galactopyranoside triethylammonium salt (4i)

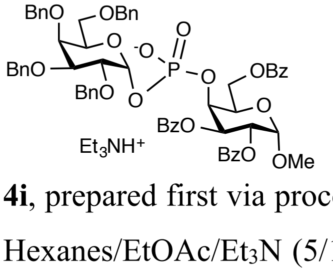

4i, prepared first via procedure (A), purified by flash chromatography by eluting with Hexanes/EtOAc/Et_3N (5/1/0.01 to 3/1/0.01) to give an intermediate product (R_f = 0.2 (Hexanes/EtOAc, 3/1), 54 mg, 60% yield); then procedure (B), quantitative yield,

colorless oil, α/β > 20/1. $[\alpha]_D^{25}$ = 85.6 (*c* 2.5 $CHCl_3$); ^{1}H NMR (400 MHz, $CDCl_3$) δ 8.03-7.13 (m, 35H, Ar*H*), 5.79 (dd, *J* = 7.1, 3.4 Hz, 1H, H-1^B), 5.58 (dd, *J* = 10.8, 3.6 Hz, 1H, H-2^A), 5.46 (dd, *J* = 10.8, 3.1 Hz, 1H, H-3^A), 5.03 (d, *J* = 3.5 Hz, 1H, H-1^A), 4.89 (dd, *J* = 14.9, 11.2 Hz, 2H, H-4^A, C*H*Ph), 4.79 – 4.73 (m, 2H, C*H*Ph, H-6^A), 4.67-4.58 (m, 4H, 3 x C*H*Ph, H-6'^{A}), 4.50 (d, *J* = 11.4 Hz, 1H, C*H*Ph), 4.41-4.35 (m, 2H, CH_2Ph), 4.31 (t, *J* = 6.7 Hz, 1H, H-5^B), 4.05 (dd, *J* = 10.0, 2.9 Hz, 1H, H-3^B), 3.98-3.94 (m, 2H, H-2^B, H-4^B), 3.57 (d, *J* = 8.8 Hz, 1H, H-5^A), 3.53-3.45 (m, 2H, H-6^B, H-6'^{B}), 3.27 (s, 3H, OMe); ^{13}C NMR (101 MHz, $CDCl_3$) δ = 166.4, 166.3, 166.2, 139.4, 139.1, 138.9, 138.5, 133.1, 132.8, 132.5, 131.2, 130.6, 130.2, 130.0, 129.9, 129.7, 128.7, 128.43, 128.37, 128.30, 128.27, 128.2, 128.0, 127.6, 127.5, 127.4, 127.2, 97.1, 94.6 (d, *J* = 6.0 Hz), 79.4, 76.6, 75.4, 75.0, 73.2, 73.1, 72.9, 71.4 (d, *J* = 5.5 Hz), 69.8 (d, *J* = 1.5 Hz), 69.7, 69.6, 69.2, 68.6 (d, *J* = 3.8 Hz), 65.6; ^{31}P NMR (162 MHz, $CDCl_3$) δ = -1.3; HRMS (m/z): $[M-Et_3NH^+]^-$ calcd. for $C_{62}H_{60}O_{17}P^-$, 1107.3574; found, 1107.3572.

***tert*-butyldimethylsilyl 2,3,4,6-tetra-*O*-benzyl-*α*-D-galactopyranosyl-phosphoryl-(→3)-2-amino-4,6-*O*-benzylidene-2-deoxy-*β*-D-glucopyranoside triethylammonium salt (4j)**

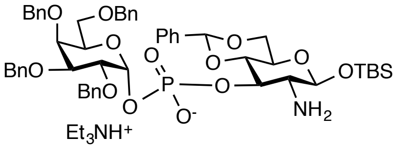

4j, prepared first via procedure (A), purified by flash chromatography by eluting with Hexanes/EtOAc/Et_3N (4/1/0.01) to give an intermediate product (R_f = 0.2 (Hexanes/EtOAc, 4/1), 52.2 mg, 63% yield); then procedure (B), quantitative yield, colorless oil, α/β > 20/1. $[\alpha]_D^{25}$ = 22 (*c* 2.4 $CHCl_3$); ^{1}H NMR (400 MHz, $CDCl_3$) δ 7.39-7.14 (m, 25H, Ar*H*), 5.78 (dd, *J* = 7.2, 3.3 Hz, 1H, H-1^B), 5.30 (s, 1H, C*H*$(O)_2$Ph), 4.77 (d, *J* = 11.5 Hz, 1H, C*H*Ph), 4.69 (d, *J* = 11.3 Hz, 1H, C*H*Ph), 4.56-4.47 (m, 3H, 2 x C*H*Ph, H-1^A), 4.43-4.27 (m, 4H, 4 x C*H*Ph), 4.15 (t, *J* = 6.7 Hz, 1H, H-3^A), 4.03 (dd, *J* = 10.4, 4.9 Hz, 1H, H-6^B), 3.91 (dt, *J* = 10.0, 3.0 Hz, 1H, H-2^B), 3.80 (d, *J* = 10.2 Hz, 1H, H-3^B), 3.72-3.65 (m, 1H, H-4^B), 3.51-3.37 (m, 5H, H-4^A, H-5^A, H-6'^{B}, H-6^A, H-6'^{A}), 3.29-3.22 (m, 1H, H-5^B), 2.74-2.67 (m, 1H, H-2^A), 0.78 (s, 9H), 0.01 (s, 3H), 0.00 (s, 3H); ^{13}C NMR (101 MHz, $CDCl_3$) δ = 139.1, 138.73, 138.66, 138.0, 137.8, 128.9, 128.5, 128.3, 128.22, 128.16, 128.13, 128.09, 128.04, 127.9, 127.8, 127.62, 127.56, 127.34, 127.28, 126.5, 101.3, 97.0 (bs), 94.1 (d, *J* = 6.5 Hz), 80.2 (d, *J* = 3.7 H), 78.6, 74.9, 74.8, 73.4, 72.8, 72.6, 70.1, 69.1, 68.5, 66.4, 59.7, 25.8, 1.1; ^{31}P NMR (162 MHz,

CDCl3) δ = -2.1; HRMS (m/z): $[M-Et_3NH^+]^-$ calcd. for $C_{53}H_{65}NO_{13}PSi^-$, 982.3968; found, 982.3966.

methyl 2,3,4,6-tetra-*O*-benzyl-*α*-D-mannopyranosyl-phosphoryl-(→6)-2,3,4-tri-*O*-benzoyl-*α*-D-glucopyranoside triethylammonium salt (4k)

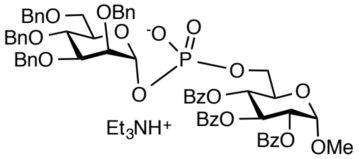

4k, prepared first via procedure (A), purified by flash chromatography by eluting with Hexanes/EtOAc/Et3N (5/1/0.01 to 3/1/0.01) to give an intermediate product (R_f = 0.2 (Hexanes/EtOAc, 2/1), 54 mg, 60% yield); then procedure (B), quantitative yield, colorless oil, α/β > 20/1. $[\alpha]_D^{25}$ = 33.9 (*c* 3.6 CHCl3); ^{1}H NMR (400 MHz, CDCl3) δ 7.90-7.11 (m, 35H, Ar*H*), 6.03 (dd, *J* = 10.2, 9.4 Hz, 1H, H-3^A), 5.67 (dd, *J* = 7.8, 2.0 Hz, 1H, H-1^B), 5.49-5.40 (m, 1H, H-4^A), 5.14 (dd, *J* = 10.2, 3.6 Hz, 1H, H-2^A), 5.03 (d, *J* = 3.6 Hz, 1H, H-1^A), 4.83 (d, *J* = 11.1 Hz, 1H, C*H*Ph), 4.68 (s, 2H, CH_2Ph), 4.55-4.45 (m, 4H, 4 x C*H*Ph), 4.35 (d, *J* = 11.9 Hz, 1H, C*H*Ph), 4.17 (ddd, *J* = 10.2, 5.9, 2.0 Hz, 1H, H-5^A), 4.04 (ddd, *J* = 11.5, 5.8, 2.1 Hz, 1H, H-6^A), 3.99-3.92 (m, 5H, H-2^B, H-3^B, H-4^B, H-5^B, H-6'^{A}), 3.66 (dd, *J* = 10.7, 3.5 Hz, 1H, H-6^B), 3.56 (dd, *J* = 10.9, 1.3 Hz, 1H, H-6'^{B}), 3.29 (s, 3H, OMe); ^{13}C NMR (101 MHz, CDCl3) δ = 165.9, 165.8, 165.3, 139.1, 138.9, 138.8, 138.7, 133.4, 133.3, 129.98, 129.88, 129.7, 129.5, 129.2, 128.5, 128.4, 128.34, 128.29, 128.25, 128.22, 128.0, 127.8, 127.7, 127.6, 127.44, 127.41, 127.3, 96.7, 94.3 (d, *J* = 5.5 Hz), 79.8, 75.7 (d, *J* = 7.2 Hz), 74.9, 74.8, 73.4, 72.9, 72.5, 72.2, 71.9, 71.0, 69.6, 69.4, 69.0 (d, *J* = 8.7 Hz), 64.3 (d, *J* = 4.9 Hz), 55.6; ^{31}P NMR (162 MHz, CDCl3) δ = -2.5; HRMS (m/z): $[M-Et_3NH^++2H^+]^+$ calcd. for $C_{62}H_{62}O_{17}P^+$, 1109.3719; found, 1109.3716.

2,3,4,6-tetra-*O*-benzyl-*α*-D-mannopyranosyl-phosphoryl-(→6)-1,2:3,4-di-*O*-isopropylidene-*α*-D-galactopyranose triethylammonium salt (4l)

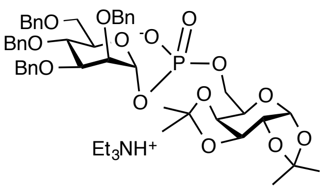

4l, prepared first via procedure (A), purified by flash chromatography by eluting with Hexanes/EtOAc/Et3N (5/1/0.01 to 3/1/0.01) to give an intermediate product (R_f = 0.2 (Hexanes/EtOAc, 2/1), 46.7 mg, 65% yield); then procedure (B), quantitative yield, colorless oil, α/β > 20/1. $[\alpha]_D^{25}$ = -14.3 (*c* 2.8 CHCl3); ^{1}H NMR (400 MHz, CDCl3) δ

7.34-7.10 (m, 20H, Ar*H*), 5.69 (d, *J* = 8.0 Hz, 1H, H-1^{B}), 5.40 (d, *J* = 5.0 Hz, 1H, H-1^{A}), 4.84 (d, *J* = 11.1 Hz, 1H, C*H*Ph), 4.76-4.64 (m, 2H, CH_2Ph), 4.58 (d, *J* = 12.0 Hz, 1H, C*H*Ph), 4.55-4.39 (m, 6H, 5 x C*H*Ph, H-4^{A}), 4.19 (dd, *J* = 5.2, 2.3 Hz, 1H, H-2^{A}), 4.14 (d, *J* = 8.0 Hz, 1H, H-3^{A}), 4.03-3.93 (m, 7H, H-2^{B}, H-3^{B}, H-4^{B}, H-5^{B}, H-5^{A}, H-6^{A}, H-6'^{A}), 3.71 (dd, *J* = 10.9, 3.3 Hz, 1H, H-6^{B}), 3.63 (d, *J* = 10.8 Hz, 1H, H-6'^{B}); ^{13}C NMR (101 MHz, $CDCl_3$) δ = 139.1, 138.9, 138.9, 138.7, 128.33, 128.27, 128.0, 127.8, 127.6, 127.5, 127.40, 127.35, 109.2, 108.5, 96.4, 94.3 (d, *J* = 5.3 Hz), 79.8, 75.7 (d, *J* = 7.0 Hz), 74.9, 74.8, 73.4, 72.9, 72.5, 71.8, 71.1, 70.8, 70.7, 69.7, 67.9 (d, *J* = 8.9 Hz), 64.8 (d, *J* = 5.2 Hz), 26.3, 26.1, 25.1, 24.5; ^{31}P NMR (162 MHz, $CDCl_3$) δ = -2.0; HRMS (m/z): $[M-Et_3NH^{+}+2H^{+}]^{+}$ calcd. for $C_{46}H_{56}O_{14}P^{+}$, 863.3402; found, 863.3406.

methyl 2,3,4,6-tetra-*O*-benzyl-*α*-D-mannopyranosyl-phosphoryl-(→4)-2,3,6-tri-*O*-benzyl-*α*-D-glucopyranoside triethylammonium salt (4m)

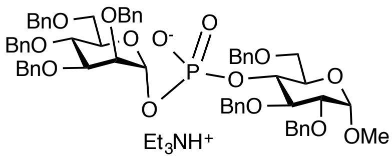

4m, prepared first via procedure (A), purified by flash chromatography by eluting with Hexanes/EtOAc/Et_3N (5/1/0.01 to 3/1/0.01) to give an intermediate product (R_f = 0.2 (Hexanes/EtOAc, 2/1), 52.0 mg, 60% yield); then procedure (B), quantitative yield, colorless oil, α/β > 20/1. $[\alpha]_D^{25}$ = 19.3 (*c* 2.2 $CHCl_3$); ^{1}H NMR (400 MHz, $CDCl_3$) δ 7.42-7.10 (m, 35H, Ar*H*), 5.72 (dd, *J* = 7.6, 1.7 Hz, 1H, H-1^{B}), 5.08 (d, *J* = 11.0 Hz, 1H, C*H*Ph), 4.79 (d, *J* = 11.1 Hz, 1H, C*H*Ph), 4.73 (d, *J* = 11.1 Hz, 1H, C*H*Ph), 4.69-4.61 (m, 3H, 3 x C*H*Ph), 4.58-4.36 (m, 7H, H-1^{A}, 6 x C*H*Ph), 4.34-4.17 (m, 2H, CH_2Ph), 4.13-3.95 (m, 2H, H-3^{B}, H-4^{A}), 3.95-3.80 (m, 5H, H-2^{B}, H-3^{A}, H-5^{B}, H-6^{B}, H-6'^{B}), 3.74 (ddd, *J* = 10.1, 6.3, 1.8 Hz, 1H, H-5^{A}), 3.68-3.62 (m, 2H, H-6^{A}, H-6'^{A}), 3.59 (dd, *J* = 10.8, 1.9 Hz, 1H, H-4^{B}), 3.36-3.26 (m, 4H, H-2^{A}, OMe); ^{13}C NMR (101 MHz, $CDCl_3$) δ = 139.9, 139.0, 138.8, 138.6, 138.50, 138.45, 128.43, 128.36, 128.35, 128.28, 128.2, 128.1, 128.0, 127.9, 127.8, 127.6, 127.52, 127.47, 127.42, 127.35, 127.20, 127.16, 98.0, 94.6, 81.4, 80.0, 79.3, 75.5, 75.0, 74.9, 74.8, 73.52, 73.46, 73.43, 72.8, 72.4, 71.8, 70.8, 70.0, 69.8, 55.2; ^{31}P NMR (162 MHz, $CDCl_3$) δ = -3.3; HRMS (m/z): $[M-Et_3NH^{+}]^{-}$ calcd. for $C_{62}H_{66}O_{14}P^{-}$, 1065.4196; found, 1065.4193.

methyl 2,3,4,6-tetra-*O*-benzyl-*α*-D-mannopyranosyl-phosphoryl-(→4)-2,3,6-tri-*O*-benzoyl-*α*-D-galactopyranoside triethylammonium salt (4n)

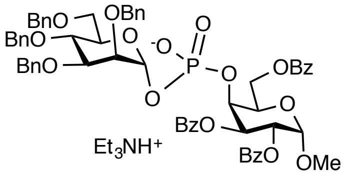

4n, prepared first via procedure (A), purified by flash chromatography by eluting with Hexanes/EtOAc/Et_3N (5/1/0.01 to 4/1/0.01) to give an intermediate product (R_f = 0.2 (Hexanes/EtOAc, 3/1), 55.7 mg, 62% yield); then procedure (B), quantitative yield, colorless oil, α/β > 20/1. $[\alpha]_D^{25}$ = 56.4 (*c* 2.3 $CHCl_3$); ^{1}H NMR (400 MHz, $CDCl_3$) δ 8.04-7.11 (m, 35H, Ar*H*), 5.79-5.67 (m, 2H, H-1^B, H-3^A), 5.56 (dd, *J* = 10.7, 3.6 Hz, 1H, H-2^A), 5.12 (d, *J* = 3.6 Hz, 1H, H-1^A), 5.03 (dd, *J* = 10.2, 3.1 Hz, 1H, H-4^A), 4.81 (d, *J* = 11.1 Hz, 1H, C*H*Ph), 4.67-4.51 (m, 5H, H-6^A, H-6'^{A}, 3 x C*H*Ph), 4.46 (d, *J* = 11.5 Hz, 2H, 2 x C*H*Ph), 4.34 (t, *J* = 12.6 Hz, 2H, 2 x C*H*Ph), 4.24 (dd, *J* = 8.2, 4.2 Hz, 1H, H-5^A), 3.99 (s, 2H, H-2^B, H-5^B), 3.94-3.91 (m, 2H, H-3^B, H-4^B), 3.70 (dd, *J* = 10.9, 4.7 Hz, 1H, H-6^B), 3.62 (d, *J* = 10.7 Hz, 1H, H-6'^{B}), 3.33 (s, 3H, OMe); ^{13}C NMR (101 MHz, $CDCl_3$) δ = 166.42, 166.36, 166.1, 139.2, 139.0, 138.7, 133.2, 133.1, 132.8, 130.4, 130.3, 130.2, 130.0, 129.8, 129.6, 128.5, 128.4, 128.3, 128.2, 128.1, 127.9, 127.8, 127.6, 127.4, 127.3, 127.2, 97.5, 94.8 (d, *J* = 5.0 Hz), 79.8, 75.8 (d, *J* = 7.9 Hz), 74.8, 73.4, 72.84, 72.77, 72.3 (d, *J* = 5.3 Hz), 71.6, 69.9, 69.7, 69.5 (d, *J* = 2.7 Hz), 68.6 (d, *J* = 3.2 Hz), 64.7, 55.4; ^{31}P NMR (162 MHz, $CDCl_3$) δ = -2.9; HRMS (m/z): $[M-Et_3NH^++2H^+]^+$ calcd. for $C_{62}H_{62}O_{17}P^+$, 1109.3719; found, 1109.3714.

***tert*-butyldimethylsilyl 2,3,4,6-tetra-*O*-benzyl-*α*-D-mannopyranosyl-phosphoryl-(→3)-2-amino-4,6-*O*-benzylidene-2-deoxy-*β*-D-glucopyranoside triethylammonium salt (4o)**

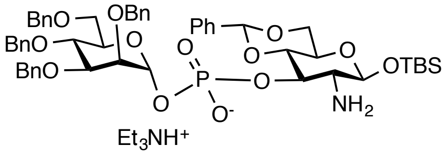

4o, prepared first via procedure (A), purified by flash chromatography by eluting with Hexanes/EtOAc/Et_3N (4/1/0.01) to give an intermediate product (R_f = 0.2 (Hexanes/EtOAc, 4/1), 52.8 mg, 64% yield); then procedure (B), quantitative yield, colorless oil, α/β > 20/1. $[\alpha]_D^{25}$ = -9.5 (*c* 2.0 $CHCl_3$); ^{1}H NMR (400 MHz, $CDCl_3$) δ 7.32-7.01 (m, 25H, Ar*H*), 5.57 (dd, *J* = 7.2, 1.6 Hz, 1H, H-1^B), 5.32 (s, 1H, C*H*Ph), 4.69 (d, *J* = 11.2 Hz, 1H, C*H*Ph), 4.54 (s, 2H, C*H*$_2$Ph), 4.49-4.43 (m, 2H, H-1^A, C*H*Ph), 4.38-4.16 (m, 6H, 5 x C*H*Ph, H-3^A), 4.06 (dd, *J* = 10.4, 4.9 Hz, 1H, H-6^A), 3.98-3.91 (m, 1H, H-5^B), 3.79-3.77 (m, 3H, H-2^B, H-3^B, H-4^B), 3.62-3.48 (m, 4H, H-4^A, H-6^B, H-

6’^{B}, H-6’^{A}), 3.23-3.14 (m, 2H, H-2^{A}, H-5^{A}), 0.78 (s, 9H), -0.00 (s, 3H), -0.01 (s, 3H); ^{13}C NMR (101 MHz, $CDCl_3$) δ = 139.2, 139.0, 138.8, 138.7, 137.7, 129.0, 128.4, 128.29, 128.25, 128.2, 128.1, 127.9, 127.8, 127.6, 127.53, 127.50, 127.43, 127.38, 127.3, 126.8, 101.7, 97.7, 94.7 (d, *J* = 5.9 Hz), 80.14 (d, *J* = 2.9 Hz), 80.09, 75.4 (d, *J* = 4.7 Hz), 75.3, 75.1, 74.8, 73.5, 72.7, 72.5, 71.9, 70.0, 69.0 (d, *J* = 3.0 Hz), 68.6, 66.6, 25.7, -4.3, -5.1; ^{31}P NMR (162 MHz, $CDCl_3$) δ = -3.3; HRMS (m/z): $[M-Et_3NH^++2H^+]^+$ calcd. for $C_{53}H_{67}NO_{13}PSi^+$, 984.4114; found, 984.4110.

methyl 2,3,4-tri-*O*-benzyl-*α*-L-rhamnopyranosyl-phosphoryl-(→6)-2,3,4-tri-*O*-benzoyl-*α*-D-glucopyranoside triethylammonium salt (4p)

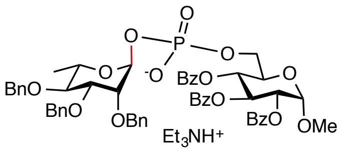

4p, prepared first via procedure (A), purified by flash chromatography by eluting with Hexanes/EtOAc/Et_3N (5/1/0.01 to 3/1/0.01) to give an intermediate product (R_f = 0.2 (Hexanes/EtOAc, 2/1), 48.6 mg, 59% yield); then procedure (B), quantitative yield, colorless oil, α/β > 20/1. $[\alpha]_D^{25}$ = 27.0 (*c* 2.1 $CHCl_3$); ^{1}H NMR (400 MHz, $CDCl_3$) δ 7.91-7.15 (m, 30H, Ar*H*), 6.04 (t, *J* = 9.9 Hz, 1H, H-3^{A}), 5.61-5.50 (m, 2H, H-4^{A}, H-1^{B}), 5.16 (dd, *J* = 10.2, 3.5 Hz, 1H, H-2^{A}), 5.06 (d, *J* = 3.5 Hz, 1H, H-1^{A}), 4.86 (d, *J* = 11.2 Hz, 1H, C*H*Ph), 4.76-4.62 (m, 2H, CH_2Ph), 4.54 (d, *J* = 11.2 Hz, 1H, C*H*Ph), 4.49 (s, 2H, CH_2Ph), 4.22-4.14 (m, 1H, H-5^{A}), 4.09-4.04 (m, 1H, H-6^{A}), 3.97 (dt, *J* = 11.1, 5.2 Hz, 1H, H-6’^{A}), 3.93-3.80 (m, 3H, H-2^{B}, H-3^{B}, H-5^{B}), 3.51 (t, *J* = 9.7 Hz, 1H, H-4^{B}), 3.35 (s, 3H, OCH_3), 1.09 (d, *J* = 6.2 Hz, 3H, CH_3); ^{13}C NMR (101 MHz, $CDCl_3$) δ = 166.0, 165.9, 165.3, 139.1, 139.0, 138.8, 133.4, 133.2, 133.1, 130.05, 130.01, 129.8, 129.6, 129.5, 129.3, 128.5, 128.4, 128.3, 128.0, 127.9, 127.6, 127.49, 127.46, 127.4, 96.9, 94.2 (d, *J* = 5.7 Hz), 80.4, 79.7, 75.8 (d, *J* = 7.7 Hz), 75.1, 72.5, 72.3, 71.9, 71.1, 69.2, 69.1, 69.0, 68.9, 64.1 (d, *J* = 5.4 Hz), 55.7, 18.1. ^{31}P NMR (162 MHz, $CDCl_3$) δ = -2.4; HRMS (m/z): $[M-Et_3NH^+]^-$ calcd. for $C_{55}H_{54}O_{16}P^-$, 1001.3155; found, 1001.3160.

2,3,4-tri-*O*-benzyl-*α*-L-rhamnopyranosyl-phosphoryl-(→6)-1,2:3,4-di-*O*-isopropylidene-*α*-D-galactopyranose triethylammonium salt (4q)

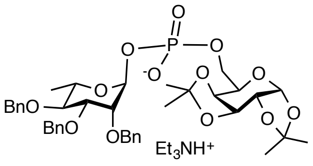

4q, prepared first via procedure (A), purified by flash chromatography by eluting with Hexanes/EtOAc/Et_3N (5/1/0.01 to 3/1/0.01) to give an intermediate product (R_f = 0.2 (Hexanes/EtOAc, 2/1), 38.6 mg, 61% yield); then procedure (B), quantitative yield, colorless oil, α/β > 20/1. $[\alpha]_D^{25}$ = -14.8 (*c* 1.9 $CHCl_3$); ^{1}H NMR (400 MHz, $CDCl_3$) δ 7.33-71.6 (m, 15H, Ar*H*), 5.59 (dd, *J* = 7.8, 1.7 Hz, 1H, H-1^B), 5.41 (d, *J* = 5.0 Hz, 1H, H-1^A), 4.88 (d, *J* = 11.1 Hz, 1H, C*H*Ph), 4.72-4.65 (m, 2H, CH_2Ph), 4.57-4.48 (m, 4H, 3 x C*H*Ph, H-4^A), 4.27-4.16 (m, 2H, H-2^A, H-3^A), 4.05-3.81 (m, 6H, H-2^B, H-3^B, H-5^B, H-5^A, H-6^A, H-6'^{A}), 3.62-3.49 (m, 1H, H-4^B), 1.42 (s, 3H), 1.32 (s, 3H, Me), 1.24 (s, 3H, Me), 1.23 (s, 3H, Me), 1.22 (m, 6H, 2 x Me), 1.20 (s, 3H, Me); ^{13}C NMR (101 MHz, $CDCl_3$) δ = 139.1, 139.0, 138.8, 128.4, 128.3, 128.0, 127.9, 127.6, 127.5, 127.4, 109.3, 108.5, 96.5, 94.5 (d, *J* = 5.6 Hz), 80.4, 79.8, 75.9 (d, *J* = 8.2 Hz), 75.2, 72.7, 71.9, 71.0, 70.8, 70.75, 70.73, 69.0, 67.7 (d, *J* = 9.5 Hz), 64.5 (d, J = 5.2 Hz), 26.3, 26.1, 25.1, 24.5, 18.2; ^{31}P NMR (162 MHz, $CDCl_3$) δ = -2.1; HRMS (m/z): $[M-Et_3NH^++Na^++H^+]^+$ calcd. for $C_{39}H_{49}NaO_{13}P^+$, 779.2803; found, 779.2798.

methyl 2,3,4-tri-*O*-benzyl-*α*-L-rhamnopyranosyl-phosphoryl-(→4)-2,3,6-tri-*O*-benzyl-*α*-D-glucopyranoside triethylammonium salt (4r)

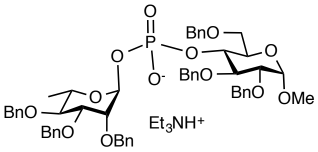

4r, prepared first via procedure (A), purified by flash chromatography by eluting with Hexanes/EtOAc/Et_3N (5/1/0.01 to 3/1/0.01) to give an intermediate product (R_f = 0.2 (Hexanes/EtOAc, 2/1), 46.2 mg, 59% yield); then procedure (B), quantitative yield, colorless oil, α/β > 20/1. $[\alpha]_D^{25}$ = 9.9 (*c* 3.3 $CHCl_3$); ^{1}H NMR (400 MHz, $CDCl_3$) δ 7.43-7.10 (m, 30H, Ar*H*), 5.61 (dd, *J* = 7.1, 2.0 Hz, 1H, H-1^B), 5.06 (d, *J* = 11.0 Hz, 1H, C*H*Ph), 4.89-4.81 (m, 1H, C*H*Ph), 4.71 (d, *J* = 11.1 Hz, 1H, C*H*Ph), 4.69- 4.64 (m, 1H, C*H*Ph), 4.62 (s, 2H, CH_2Ph), 4.57-4.47 (m, 5H, H-1^A, 4 x C*H*Ph), 4.41-4.34 (m, 2H, CH_2Ph), 4.19-4.12 (m, 1H, H-4^B), 4.00-3.90 (m, 3H, H-2^B, H-3^B, H-5^B, H-6^A), 3.88-3.78 (m, 3H, H-3^A, H-5^A), 3.71 (dd, *J* = 10.9, 6.4 Hz, 1H, H-6'^{A}), 3.60-3.48 (m, 1H, H-4^A), 3.46-3.38 (m, 1H, H-2^A), 3.33 (s, 3H, OMe), 1.19 (d, *J* = 6.3 Hz, 3H, CH_3); ^{13}C NMR (101 MHz, $CDCl_3$) δ = 139.7, 139.0, 138.9, 138.71, 138.68, 138.3, 128.3, 128.29, 128.18, 128.1, 128.07, 128.04, 128.01, 127.72, 127.68, 127.3, 127.2, 127.1, 127.0, 97.8, 94.5 (d, *J* = 5.3 Hz), 81.1 (d, *J* = 3.1 Hz), 80.3 , 79.8, 79.0, 75.7 (d, *J* = 8.1 Hz), 75.0, 74.9, 74.5 (d, *J* = 6.4 Hz), 73.3, 72.5, 71.7, 70.6 (d, *J* = 3.4 Hz), 69.9, 68.9,

55.1; ^{31}P NMR (162 MHz, $CDCl_3$) δ = -3.6; HRMS (m/z): $[M-Et_3NH^{+}+H^{+}+Na^{+}]^{+}$ calcd. for $C_{55}H_{61}NaO_{13}P^{+}$, 983.3742; found, 983.3749.

methyl 2,3,4-tri-*O*-benzyl-*α*-L-rhamnopyranosyl-phosphoryl-(→4)-2,3,6-tri-*O*-benzoyl-*α*-D-galactopyranoside triethylammonium salt (4s)

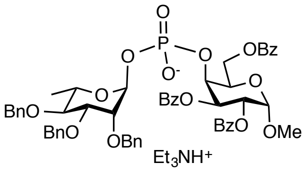

4s, prepared first via procedure (A), purified by flash chromatography by eluting with Hexanes/EtOAc/Et_3N (4/1/0.01) to give an intermediate product (R_f = 0.2 (Hexanes/EtOAc, 4/1), 44.3 mg, 54% yield); then procedure (B), quantitative yield, colorless oil, α/β > 20/1. $[\alpha]_D^{25}$ = 61.5 (*c* 1.8 $CHCl_3$); ^{1}H NMR (400 MHz, $CDCl_3$) δ 8.02-7.12 (m, 30H, Ar*H*), 5.72-5.65 (m, 1H, H-3^{A}), 5.62 (dd, *J* = 7.2, 1.9 Hz, 1H, H-1^{B}), 5.57 (dd, *J* = 10.8, 3.6 Hz, 1H, H-2^{A}), 5.14 (d, *J* = 3.6 Hz, 1H, H-1^{A}), 5.03 (dd, *J* = 10.2, 3.1 Hz, 1H, H-4^{A}), 4.84 (d, *J* = 11.2 Hz, 1H, C*H*Ph), 4.71-4.53 (m, 5H, 3 x CHPh, H-6^{A}, H-6'^{A}), 4.50-4.38 (m, 2H, CH_2Ph), 4.31 (dd, *J* = 8.3, 3.9 Hz, 1H, H-5^{A}), 3.98 (bs, 1H, H-2^{B}), 3.93 (ddd, *J* = 12.4, 7.8, 4.8 Hz, 1H, H-5^{B}), 3.86 (dd, *J* = 9.5, 3.1 Hz, 1H, H-3^{B}), 3.52 (t, *J* = 9.5 Hz, 1H, H-4^{B}), 3.31 (s, 3H, OMe), 1.18 (d, *J* = 6.1 Hz, 3H, CH_3); ^{13}C NMR (101 MHz, $CDCl_3$) δ = 166.4, 166.3, 166.1, 139.2, 139.1, 138.9, 133.2, 133.0, 132.8, 130.34, 130.29, 130.0, 129.8, 129.7, 128.5, 128.4, 128.34, 128.29, 128.25, 128.0, 127.8, 127.6, 127.40, 127.36, 127.3, 97.4, 95.0 (d, *J* = 5.4 Hz), 80.4, 79.9, 76.0 (d, *J* = 8.4 Hz), 75.0, 72.9, 72.4 (d, *J* = 5.3 Hz), 71.8, 69.8 (d, *J* = 2.8 Hz), 69.6, 69.1, 68.6 (bs), 64.8, 55.3, 18.2; ^{31}P NMR (162 MHz, $CDCl_3$) δ = -2.9; HRMS (m/z): $[M-Et_3NH^{+}+Na^{+}+H^{+}]^{+}$ calcd. for $C_{55}H_{55}NaO_{16}P^{+}$, 1025.3120; found, 1025.3112.

methyl 2-amino-3,4,6-tri-*O*-benzyl-2-deoxy-*α*-D-glucopyranosyl-phosphoryl-(→6)-2,3,4-tri-*O*-benzoyl-*α*-D-glucopyranoside triethylammonium salt (4t)

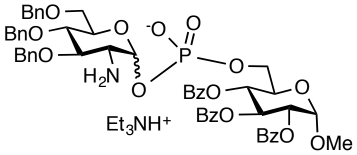

4t, prepared first via procedure (A), purified by flash chromatography by eluting with Hexanes/EtOAc/Et_3N (5/1/0.01 to 3/1/0.01) to give an intermediate product (R_f= 0.2 (Hexanes/EtOAc, 2/1), 56.1 mg, 66% yield, α/β = 7.6/1); then procedure (B), quantitative yield, colorless oil. $[\alpha]_D^{25}$ = 66.6 (*c* 2.2 $CHCl_3$); ^{1}H NMR (400 MHz, $CDCl_3$) δ 7.89-7.15 (m, 30H, Ar*H*), 6.09 (t, *J* = 9.8 Hz, 1H, H-3^{A}), 5.70 (dd, *J* = 7.5, 3.2 Hz, 1H, H-1^{B}), 5.51 (t, *J* = 9.8 Hz, 1H, H-4^{A}), 5.18 (dd, *J* = 10.2, 3.6 Hz, 1H, H-2^{A}), 5.09

(d, J = 3.7 Hz, 1H, H-1^A), 4.88 (d, J = 11.2 Hz, 1H, CHPh), 4.81-4.69 (m, 2H, 2 x CHPh), 4.54 (d, J = 11.7 Hz, 2H, 2 x CHPh), 4.41 (d, J = 12.1 Hz, 1H, CHPh), 4.25-3.98 (m, 4H, H-5^A, H-5^B, H-6^B, H-6'^{B}), 3.79 (t, J = 9.5 Hz, 1H, H-3^B), 3.73-3.53 (m, 3H, H-4^B, H-6^A, H-6'^{A}), 3.35 (s, 3H), 2.95-2.91 (m, 1H, H-2^B); ^{13}C NMR (101 MHz, $CDCl_3$) δ = 165.79, 165.77, 165.2, 138.6, 138.4, 138.2, 133.3, 133.2, 133.0, 129.92, 129.88, 129.81, 129.6, 129.4, 129.20, 129.16, 128.4, 128.34, 128.28, 128.23, 128.19, 128.0, 127.90, 127.86, 127.84, 127.7, 127.6, 127.6, 127.5, 96.7, 95.1, 78.4, 75.4, 74.5, 73.4, 72.1, 72.0, 70.9, 69.3, 68.9, 68.8, 68.4, 64.2, 55.6, 55.5; ^{31}P NMR (162 MHz, $CDCl_3$) δ = -2.2; HRMS (m/z): [M-Et_3NH^+]$^-$ calcd. for $C_{55}H_{55}NO_{16}P^-$, 1016.3264; found, 1016.3259.

2-amino-3,4,6-tri-*O*-benzyl-2-deoxy-*α*-D-glucopyranosyl-phosphoryl-(→6)-1,2:3,4-di-*O*-isopropylidene-*α*-D-galactopyranose triethylammonium salt (4u)

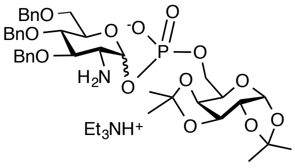

4u, prepared first via procedure (A), purified by flash chromatography by eluting with Hexanes/EtOAc/Et_3N (5/1/0.01 to 3/1/0.01) to give an intermediate product (R_f = 0.2 (Hexanes/EtOAc, 2/1), 44 mg, 66% yield, α/β = 8.4/1); then procedure (B), quantitative yield, colorless oil. $[\alpha]_D^{25}$ = 19.0 (*c* 1.8 $CHCl_3$); ^{1}H NMR (400 MHz, $CDCl_3$) δ 7.28-7.06 (m, 15H, ArH), 5.68 (dd, J = 7.7, 3.3 Hz, 1H, H-1^B), 5.35 (d, J = 5.0 Hz, 1H, H-1^A), 4.85-4.73 (m, 2H, CH_2Ph), 4.67 (d, J = 11.2 Hz, 1H, CHPh), 4.51 (d, J = 12.1 Hz, 1H, CHPh), 4.47 (d, J = 11.2 Hz, 1H, CHPh), 4.41 (dd, J = 7.9, 2.4 Hz, 1H, H-3^A), 4.36 (d, J = 12.1 Hz, 1H, CHPh), 4.15 (dd, J = 5.0, 2.3 Hz, 1H, H-2^A), 4.06 (dd, J = 7.9, 1.6 Hz, 1H, H-4^A), 3.99-3.89 (m, 4H, H-5^B, H-5^A, H-6^A, H-6'^{A}), 3.84 (t, J = 9.6 Hz, 1H, H-3^B), 3.71-3.65 (m, 1H, H-6^B), 3.65-3.60 (m, 1H, H-4^B), 3.56 (dd, J = 11.1, 1.9 Hz, 1H, H-6'^{B}), 3.07-3.00 (m, 1H, H-2^B), 1.38 (s, 3H, CH_3), 1.28 (s, 3H, CH_3), 1.18 (s, 3H, CH_3), 1.16 (s, 3H, CH_3); ^{13}C NMR (101 MHz, $CDCl_3$) δ = 137.4, 137.2, 137.1, 127.4, 127.3, 127.3, 127.0, 126.9, 126.6, 126.5, 108.1, 107.6, 95.2, 93.2 (bs), 79.1, 77.4, 74.3, 73.5, 72.4, 71.0, 69.8, 69.6, 69.5, 67.4, 66.4 (d, J = 8.8 Hz), 63.4 (d, J = 5.2 Hz), 53.9, 25.0, 24.9, 23.9, 23.3; ^{31}P NMR (162 MHz, $CDCl_3$) δ = -1.9; HRMS (m/z): [M-Et_3NH^+]$^-$ calcd. for $C_{39}H_{49}NO_{13}P^-$, 770.2947; found, 770.2945.

methyl 2-amino-3,4,6-tri-*O*-benzyl-2-deoxy-*α*-D-glucopyranosyl-phosphoryl-(→4)-2,3,6-tri-*O*-benzyl-*α*-D-glucopyranoside triethylammonium salt (4v)

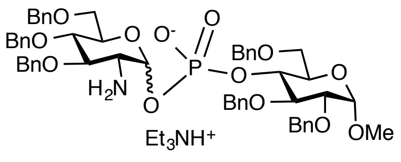

4v, prepared first via procedure (A), purified by flash chromatography by eluting with Hexanes/EtOAc/Et_3N (5/1/0.01 to 3/1/0.01) to give an intermediate product (R_f = 0.2 (Hexanes/EtOAc, 2/1), 52.9 mg, 65% yield, α/β = 6.6/1); then procedure (B), quantitative yield, colorless oil. $[\alpha]_D^{25}$ = 34.2 (*c* 2.4 $CHCl_3$); ^{1}H NMR (400 MHz, $CDCl_3$) δ 7.41-7.02 (m, 30H, Ar*H*), 5.71 (dd, *J* = 6.7, 3.2 Hz, 1H, H-1^B), 5.02 (d, *J* = 11.1 Hz, 1H, C*H*Ph), 4.74 (d, *J* = 11.2 Hz, 1H, C*H*Ph), 4.68-4.40 (m, 9H, 8 x C*H*Ph, H-1^A), 4.32 (d, *J* = 12.1 Hz, 1H, C*H*Ph), 4.26-4.11 (m, 2H, C*H*Ph, H-4^A), 4.01 (d, *J* = 9.4 Hz, 1H, H-5^B), 3.94 (dd, *J* = 11.0, 1.8 Hz, 1H, H-6^A), 3.84 (t, *J* = 9.3 Hz, 1H, H-3^A), 3.80-3.73 (m, 1H, H-5^A), 3.71-3.59 (m, 2H, H-6'^{A}, H-6^B), 3.59-3.47 (m, 3H, H-3^B, H-4^B, H-6'^{B}), 3.39 (dd, *J* = 9.7, 3.7 Hz, 1H, H-2^A), 3.30 (s, 3H, OMe), 2.82-2.78 (m, 1H, H-2^B); ^{13}C NMR (101 MHz, $CDCl_3$) δ = 138.4, 137.8, 137.4, 137.2, 136.9, 127.4, 127.3, 127.25, 127.19, 127.1, 127.0, 126.8, 126.63, 126.58, 126.52, 126.47, 126.3, 126.24, 126.18, 96.7, 93.8 (bs), 80.2, 80.0, 78.2, 77.3, 74.4, 74.1, 73.4, 73.4, 72.4, 72.3, 72.2, 70.9, 69.6 (bs), 68.7 (bs), 67.3 (bs), 54.4, 54.1; ^{31}P NMR (162 MHz, $CDCl_3$) δ = -2.5; HRMS (m/z): $[M-Et_3NH^+]^-$ calcd. for $C_{55}H_{61}NO_{13}P^-$, 974.3886; found, 974.3882.

methyl 2-amino-3,4,6-tri-*O*-benzyl-2-deoxy-*α*-D-glucopyranosyl-phosphoryl-(→4)-2,3,6-tri-*O*-benzoyl-*α*-D-galactopyranoside triethylammonium salt (4w)

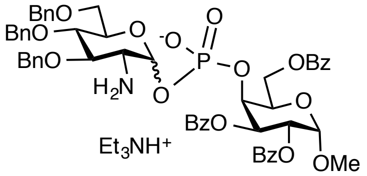

4w, prepared first via procedure (A), purified by flash chromatography by eluting with Hexanes/EtOAc/Et_3N (4/1/0.01) to give an intermediate product (R_f = 0.2 (Hexanes/EtOAc, 4/1), 56.1 mg, 66% yield, α/β = 3/1); then procedure (B), quantitative yield, colorless oil. $[\alpha]_D^{25}$ = 82.1 (*c* 3.5 $CHCl_3$); α anomer: ^{1}H NMR (400 MHz, $CDCl_3$) δ 8.10-7.13 (m, 30H, Ar*H*), 5.84-5.69 (m, 2H, H-1^B, H-3^A), 5.57 (dd, *J* = 10.7, 3.6 Hz, 1H, H-2^A), 5.16 (d, *J* = 3.7 Hz, 1H, H-1^A), 5.00 (dd, *J* = 10.0, 3.1 Hz, 1H, H-4^A), 4.73 (s, 2H, C*H*$_2$Ph), 4.70-4.49 (m, 5H, H-6^A, H-6'^{A}, 3 x C*H*Ph), 4.42 (d, *J* = 11.8 Hz, 1H, C*H*Ph), 4.28-4.20 (m, 1H, H-5^A), 4.08 (d, *J* = 10.1 Hz, 1H, H-5^B), 3.85-3.59 (m, 4H, H-3^B,H-4^B, H-6^B, H-6'^{B}), 3.41 (s, 3H, OMe), 2.87-2.85 (m, 1H, H-2^B); ^{13}C NMR (101 MHz, $CDCl_3$) δ = 166.7, 166.3, 166.0, 138.6, 138.4, 138.3, 133.2, 133.1, 133.0, 130.3, 130.0, 129.9, 129.8, 129.7, 129.6, 128.45, 128.41, 128.35, 128.30, 128.26, 128.1, 127.9,

127.8, 127.59, 127.57, 127.50, 97.3, 94.7, 80.3, 78.5, 75.3, 74.4, 73.5, 72.2, 72.1, 69.6, 69.3, 68.8, 68.2, 65.2, 55.3; ^{31}P NMR (162 MHz, $CDCl_3$) δ = -2.1; HRMS (m/z): [M-Et_3NH^+]$^-$ calcd. for $C_{55}H_{55}NO_{16}P^-$, 1016.3264; found, 1016.3285.

methyl 2-amino-3,4,6-tri-*O*-benzyl-2-deoxy-*α*-D-galactopyranosyl-phosphoryl-(→6)-2,3,4-tri-*O*-benzoyl-*α*-D-glucopyranoside triethylammonium salt (4x)

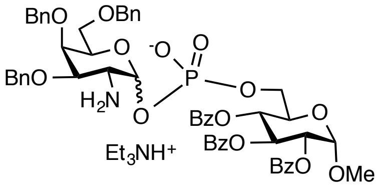

4x, prepared first via procedure (A), purified by flash chromatography by eluting with Hexanes/EtOAc/Et_3N (5/1/0.01 to 3/1/0.01) to give an intermediate product (R_f = 0.2 (Hexanes/EtOAc, 2/1), 56.1 mg, 66% yield, α/β = 9.7/1); then procedure (B), quantitative yield, colorless oil. $[\alpha]_D^{25}$ = 59.1 (*c* 1.9 $CHCl_3$); ^{1}H NMR (400 MHz, $CDCl_3$) δ 7.90-7.14 (m, 30H, Ar*H*), 5.98 (t, *J* = 9.8 Hz, 1H, H-2^A), 5.69 (bs, 1H, H-1^B), 5.39 (t, *J* = 10 Hz, 1H, H-3^A), 5.07 (d, *J* = 10.0 Hz, 1H, H-1^A), 4.97 (s, 1H, H-4^B), 4.66 (d, *J* = 11.3 Hz, 1H, C*H*Ph), 4.45-4.27 (m, 5H, C*H*Ph, C*H*$_2$Ph x 2), 4.07-3.98 (m, 5H, H-5^B, H-4^A, H-5^A, H-6^A, H-6'^{A}), 3.78 (s, 1H, H-3^B), 3.49-3.43 (m, 3H, H-2^B, H-6^B, H-6'^{B}), 3.20 (s, 3H, C*H*$_3$); ^{13}C NMR (101 MHz, $CDCl_3$) δ = 165.9, 165.3, 138.6, 138.3, 138.0, 133.4, 133.3, 133.0, 130.1, 130.0, 129.8, 129.6, 129.4, 129.3, 128.6, 128.5, 128.5, 128.3, 128.2, 128.0, 127.8, 127.7, 96.7, 93.9 (bs), 74.7, 73.5, 72.3, 71.9, 71.1, 70.6, 69.5, 68.9 (d, *J* = 7.9 Hz), 68.5, 64.5 (bs), 55.6, 45.6; ^{31}P NMR (162 MHz, $CDCl_3$) δ = -2.5; HRMS (m/z): [M-Et_3NH^+]$^-$ calcd. for $C_{55}H_{55}NO_{16}P^-$, 1016.3264; found, 1016.3264.

2-amino-3,4,6-tri-*O*-benzyl-2-deoxy-*α*-D-galactopyranosyl-phosphoryl-(→6)-1,2:3,4-di-*O*-isopropylidene-*α*-D-galactopyranose triethylammonium salt (4y)

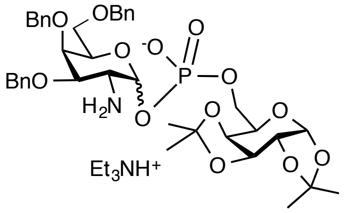

4y, prepared first via procedure (A), purified by flash chromatography by eluting with Hexanes/EtOAc/Et_3N (5/1/0.01 to 3/1/0.01) to give an intermediate product (R_f = 0.2 (Hexanes/EtOAc, 2/1), 44 mg, 66% yield, α/β = 7.9/1); then procedure (B), quantitative yield, colorless oil. $[\alpha]_D^{25}$ = 13.5 (*c* 2.6 $CHCl_3$); ^{1}H NMR (400 MHz, $CDCl_3$) δ 7.36-7.15 (m, 15H, Ar*H*), 5.68 (bs, 1H, H-1^B), 5.32 (d, *J* = 5.0 Hz, 1H, H-1^A), 4.68 (d, *J* = 11.1 Hz, 2H, 2 x C*H*Ph), 4.58 (d, *J* = 11.0 Hz, 1H, C*H*Ph), 4.42-4.28 (m, 4H, 3 x C*H*Ph,

H-3^{A}), 4.18 (td, J = 6.3, 3.3 Hz, 1H, H-5^{A}), 4.08 (ddd, J = 15.2, 6.5, 2.5 Hz, 2H, H-2^{A}, H-4^{A}), 4.02-3.82 (m, 5H, H-3^{B}, H-4^{B}, H-5^{B}, H-6^{A}, H-6'^{A}), 3.61-3.39 (m, 3H, H-2^{B}, H-6^{B}, H-6'^{B}), 1.34 (s, 3H, CH_3), 1.25 (s, 3H, CH_3), 1.12 (s, 3H, CH_3); ^{13}C NMR (101 MHz, $CDCl_3$) δ = 138.8, 138.3, 138.1, 128.55, 128.46, 128.41, 128.3, 127.9, 127.84, 127.79, 127.68, 127.5, 109.2, 108.7, 96.3, 93.6, 74.7, 73.4, 72.5 (bs), 72.0 (bs), 70.9, 70.7, 70.3, 68.4, 67.4 (d, J = 7.6 Hz), 64.6 (bs), 51.0, 26.2, 26.1, 25.0, 24.5; ^{31}P NMR (162 MHz, $CDCl_3$) δ = -2.0; HRMS (m/z): [M-Et_3NH^+]$^-$ calcd. for $C_{39}H_{49}NO_{13}P^-$, 770.2947; found, 770.2947.

methyl 2-amino-3,4,6-tri-*O*-benzyl-2-deoxy-*α*-D-galactopyranosyl-phosphoryl-(→4)-2,3,6-tri-*O*-benzyl-*α*-D-glucopyranoside triethylammonium salt (4z)

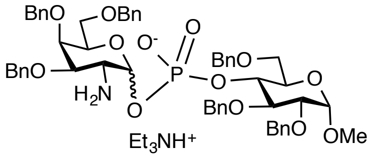

4z, prepared first via procedure (A), purified by flash chromatography by eluting with Hexanes/EtOAc/Et_3N (5/1/0.01 to 3/1/0.01) to give an intermediate product (R_f = 0.2 (Hexanes/EtOAc, 2/1), 54 mg, 66% yield, α/β = 11/1); then procedure (B), quantitative yield, colorless oil. $[\alpha]_D^{25}$ = 39.7 (c 2.3 $CHCl_3$); ^{1}H NMR (400 MHz, $CDCl_3$) δ 5.75 (dd, J = 6.8, 3.2 Hz, 1H, H-1^{B}), 4.97 (d, J = 10.8 Hz, 1H, C*H*Ph), 4.72 (d, J = 10.9 Hz, 1H, C*H*Ph), 4.58 (d, J = 11.8 Hz, 2H, 2 x C*H*Ph), 4.53-4.37 (m, 4H, H-1^{A}, 3 x C*H*Ph), 4.37-4.16 (m, 6H, 5 x C*H*Ph, H-4^{A}), 4.11 (dd, J = 8.3, 5.6 Hz, 1H, H-5^{B}), 3.85 (t, J = 9.3 Hz, 2H, H-5^{A}, H-6^{A}), 3.80-3.65 (m, 3H, H-3^{A}, H-4^{B}, H-6'^{B}), 3.64-3.56 (m, 1H, H-3^{B}), 3.46 (t, J = 8.6 Hz, 1H, H-6^{B}), 3.39-3.26 (m, 6H, H-2^{B}, H-6'^{A}, H-2^{A}, OMe); ^{13}C NMR (101 MHz, $CDCl_3$) δ = 139.5, 138.9, 138.4, 138.3, 137.9, 137.7, 128.4, 128.34, 128.26, 128.23, 128.19, 128.0, 127.9, 127.81, 127.78, 127.73, 127.66, 127.55, 127.4, 127.2, 97.8, 93.9 (bs), 81.0, 79.4, 77.2, 75.1, 74.6, 74.4 (d, J = 7.0 Hz), 73.3, 73.2, 72.0, 71.5, 70.6 (d, J = 3.9 Hz), 70.3, 69.5, 68.1, 55.1, 51.3; ^{31}P NMR (162 MHz, $CDCl_3$) δ = -2.3; HRMS (m/z): [M-Et_3NH^+]$^-$ calcd. for $C_{55}H_{61}NO_{13}P^-$, 974.3886; found, 974.3882.

methyl 2-amino-3,4,6-tri-*O*-benzyl-2-deoxy-*α*-D-galactopyranosyl-phosphoryl-(→4)-2,3,6-tri-*O*-benzoyl-*α*-D-galactopyranoside triethylammonium salt (4za)

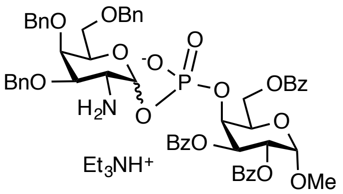

4za, prepared first via procedure (A), purified by flash chromatography by eluting with Hexanes/EtOAc/Et_3N (4/1/0.01) to give an intermediate product (R_f = 0.2 (Hexanes/EtOAc, 4/1), 56.0 mg, 66% yield, α/β = 8/1); then procedure (B), quantitative yield, colorless oil. $[\alpha]_D^{25}$ = 78.4 (*c* 2.4 $CHCl_3$); ^1H NMR (400 MHz, $CDCl_3$) δ 8.12-7.22 (m, 30H, Ar*H*), 5.85-5.71 (m, 2H, H-1^B, H-3^A), 5.59 (dd, *J* = 10.6, 3.6 Hz, 1H, H-2^A), 5.12 (d, *J* = 3.6 Hz, 1H, H-1^A), 5.04 (dd, *J* = 10.1, 3.0 Hz, 1H, H-4^A), 4.81 (dd, *J* = 11.8, 8.8 Hz, 1H, H-6^A), 4.76 (d, *J* = 11.4 Hz, 1H, C*H*Ph), 4.62-4.42 (m, 6H, 5 x C*H*Ph, H-6'^{A}), 4.31 (t, *J* = 6.8 Hz, 1H, H-5^B), 4.17 (d, *J* = 7.9 Hz, 1H, H-5^A), 3.88 (bs, 1H, H-4^B), 3.75 (d, *J* = 10.5 Hz, 1H, H-3^B), 3.67 (dd, *J* = 9.1, 6.0 Hz, 1H, H-6^B), 3.59 (t, *J* = 8.4 Hz, 1H, H-6^B), 3.45 (dd, *J* = 8.3, 4.9 Hz, 1H, H-2^B), 3.33 (s, 3H, OMe); ^{13}C NMR (101 MHz, $CDCl_3$) δ = 166.7, 166.04, 166.00, 138.8, 138.5, 138.2, 133.15, 133.09, 130.28, 130.26, 130.21, 130.0, 129.8, 129.7, 128.5, 128.42, 128.38, 128.29, 128.1, 128.0, 127.7, 127.6, 127.5, 97.3, 95.4, 74.7, 73.4, 72.6, 72.4 (d, *J* = 4.9 Hz), 71.7, 70.5, 69.9, 69.4 (d, *J* = 3.9 Hz), 69.1, 68.3, 65.2, 55.3, 51.4 (d, *J* = 6.9 Hz); ^{31}P NMR (162 MHz, $CDCl_3$) δ = -2.3; HRMS (m/z): $[M-Et_3NH^+]^-$ calcd. for $C_{55}H_{55}NO_{16}P^-$, 1016.3264; found, 1016.3260.

2,3,4,6-tetra-*O*-benzyl-*α*-D-mannopyranosyl-phosphoryl-(→3)-*N*-(9-fluorenylmethoxycarbonyl)-L-serine allyl ester (4zb)

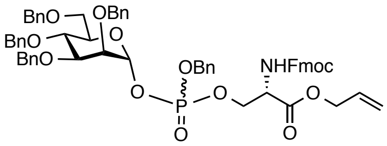

4zb prepared via procedure (A), colorless oil, 31.8 mg, 40% yield, α/β > 20/1, R_f = 0.2 (Hexanes/EtOAc, 3/2). Compound **4zb** was characterized without hydrogenolysis. ^1H NMR (400 MHz, $CDCl_3$) δ 7.74-7.13 (m, 76H, Ar*H*), 6.16 (d, *J* = 8.5 Hz, 1H, N*H*), 6.09 (d, *J* = 8.4 Hz, 1H, N*H'*), 5.89-5.78 (m, 3H, C*H*=CH_2, C*H'*=CH_2, H-1), 5.68 (dd, *J* = 6.2, 2.1 Hz, 1H, H-1'), 5.29-5.25 (2H, CH=C*H*H, CH=C*H'*H), 5.21-5.18 (m, 2H, CH=CH*H*, CH=CH*H'*), 5.05 (d, *J* = 8.6 Hz, 2H, CH_2Ph), 4.97 (d, *J* = 8.9 Hz, 2H, CH_2Ph), 4.85 (dd, *J* = 10.8, 2.1 Hz, 2H, CH_2Ph), 4.74-4.17 (m, 30H, 2 x CH (Fmoc), 2 x C*H*NH, 7 x CH_2Ph, 2 x CH_2OP, 2 x CH_2(Fmoc), 2 x C*H_2*CH=CH_2), 4.03-3.60 (m, 12H, 2 x H-2, 2 x H-3, 2 x H-4, 2 x H-5, 2 x H-6, 2 x H-6'); ^{13}C NMR (101 MHz, $CDCl_3$) δ = 168.7, 156.1, 143.9, 143.9, 141.4, 138.33, 138.27, 138.2, 137.9, 135.6, 135.51, 135.45, 131.5, 131.4, 128.9, 128.8, 128.53, 128.49, 128.45, 128.44, 128.23, 128.16, 128.11, 128.09, 128.05, 128.01, 127.99, 127.96, 127.92, 127.85, 127.82, 127.79, 127.74, 127.70, 127.67, 127.3, 125.41, 125.38, 120.1, 120.0, 119.2, 96.9 (d, *J*

= 6.8 Hz), 96.6 (d, J = 6.0 Hz), 93.0, 79.8, 78.9, 75.34, 75.30, 75.24, 75.18, 75.0, 74.8, 74.7, 74.6, 74.32, 74.25, 74.19, 73.5, 73.4, 73.1, 73.0, 72.9, 72.5, 72.41, 72.37, 72.0, 70.0, 69.9, 69.8, 69.04, 69.00, 67.5, 66.6, 54.6, 47.2; ^{31}P NMR (162 MHz, $CDCl_3$) δ = -2.3, -2.8; HRMS (m/z): $[M+H^+]^+$ calcd. for $C_{62}H_{63}NO_{13}P^+$, 1060.4032; found, 1060.4029.

dibenzyl 2,3,4,6-tetra-*O*-benzyl-*α*-D-galactosyl-(1→4)-2,4,6-tri-*O*-benzyl-*α*-D-glucopyranosyl phosphate (4zc)

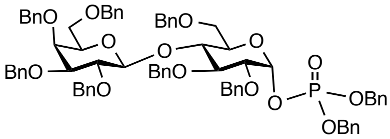

4zc, prepared first via procedure (A), purified by flash chromatography by eluting with Hexanes/EtOAc/Et_3N (5/1/0.01 to 2/1/0.01) to give the product (R_f = 0.2 (Hexanes/EtOAc, 2/1), 54.2 mg, 59% yield, α/β = 15/1); α anomer: $[\alpha]_D^{25}$ = 38.9 (c 4.4 $CHCl_3$); ^{1}H NMR (400 MHz, $CDCl_3$) δ 7.36-7.10 (m, 45H, ArH), 5.89 (dd, J = 6.8, 3.4 Hz, 1H, H-1^A), 5.06-4.95 (m, 6H, 6 x CHPh), 4.80-4.65 (m, 8H, 8 x CHPh), 4.55 (d, J = 11.4 Hz, 1H, CHPh), 4.47 (d, J = 12.1 Hz, 1H, CHPh), 4.36 (d, J = 11.9 Hz, 1H, CHPh), 4.33-4.28 (m, 2H, CHPh, H-1^B), 4.24 (d, J = 11.8 Hz, 1H, CHPh), 3.98 (t, J = 9.5 Hz, 1H, H-4^A), 3.90 (d, J = 2.9 Hz, 1H, H-4^B), 3.83-3.71 (m, 4H, H-2^B, H-3^B, H-3^A, H-6^A), 3.57-3.51 (m, 2H, H-2^A, H-6^B), 3.39-3.33 (m, 4H, H-5^A, H-6'^{A}, H-5^B, H-6'^{B}); ^{13}C NMR (101 MHz, $CDCl_3$) δ = 139.3, 139.2, 138.9, 138.6, 138.3, 138.1, 138.0, 136.0, 128.61, 128.56, 128.49, 128.47, 128.44, 128.41, 128.29, 128.27, 128.19, 128.08, 127.98, 127.93, 127.90, 127.85, 127.80, 127.77, 127.71, 127.65, 127.5, 127.5, 127.2, 102.9, 96.1 (d, J = 6.4 Hz), 82.6, 80.1, 79.5, 78.5 (d, J = 7.2 Hz), 75.9, 75.5, 75.4, 74.8, 73.8, 73.6, 73.5, 73.3, 72.72, 72.68, 69.4 (d, J = 5.3 Hz), 69.2 (d, J = 5.2 Hz), 68.3, 67.5; ^{31}P NMR (162 MHz, $CDCl_3$) δ = -2.1; HRMS (m/z): $[M+Na^+]^+$ calcd. for $C_{75}H_{77}O_{14}PNa^+$, 1255.4943; found, 1255.4938.

methyl 2,3,4,6-tetra-*O*-benzyl-*α*-D-galactosyl-(1→4)-2,3,6-tri-*O*-benzyl-*α*-D-glucopyranosyl-phosphoryl-(→6)-2,3,4-tri-*O*-benzoyl-*α*-D-glucopyranoside triethylammonium salt (4zd)

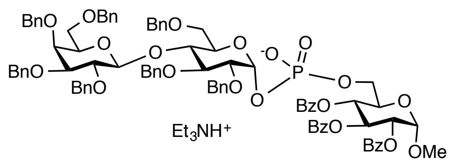

4zd, prepared first via procedure (A), purified by flash chromatography by eluting with Hexanes/EtOAc/Et_3N (5/1/0.01 to 3/1/0.01) to give an intermediate product (R_f = 0.4 (Hexanes/EtOAc, 2/1), 44.8 mg, 37% yield, α/β = 9.7/1); then procedure (B), quantitative yield, colorless oil. $[\alpha]_D^{25}$ = 43.7 (*c* 2.6 $CHCl_3$); ^1H NMR (400 MHz, $CDCl_3$) δ 6.08 (t, *J* = 9.8 Hz, 1H, H-3^A), 5.79 (dd, *J* = 8.2, 3.4 Hz, 1H, H-1^B), 5.44 (t, *J* = 9.8 Hz, 1H, H-4^A), 5.15 (dd, *J* = 10.2, 3.6 Hz, 1H, H-2^A), 5.06 (d, *J* = 3.6 Hz, 1H, H-1^A), 4.98 (t, *J* = 11.7 Hz, 2H, 2 x C*H*Ph), 4.82 (dd, *J* = 11.6, 4.2 Hz, 2H, 2 x C*H*Ph), 4.75 (d, *J* = 11.5 Hz, 1H, C*H*Ph), 4.68-4.63 (m, 4H, 4 x C*H*Ph), 4.55 (d, *J* = 11.4 Hz, 1H, C*H*Ph), 4.48 (d, *J* = 12.2 Hz, 1H, C*H*Ph), 4.43-4.33 (m, 2H, H-1^C, C*H*Ph), 4.31 (d, *J* = 12.2 Hz, 1H, C*H*Ph), 4.26-4.21 (m, 2H, H-5^A, C*H*Ph), 4.19-4.15 (m, 1H, H-6^A), 4.12-4.00 (m, 3H, H-4^B, H-5^B,H-$6'^A$), 3.90-3.87 (m, 3H, H-3^B, H-4^C, H-6^B), 3.74 (dd, *J* = 9.7, 7.6 Hz, 1H, H-2^C), 3.56-3.45 (m, 3H, H-2^B, H-6^C, H-$6'^C$), 3.38-3.24 (m, 6H, CH_3, H-3^C, H-5^C, H-$6'^B$); ^{13}C NMR (101 MHz, $CDCl_3$) δ = 165.9, 165.3, 139.7, 139.3, 139.1, 138.8, 138.5, 138.38, 133.36, 133.2, 133.04, 130.00, 129.78, 129.8, 129.6, 129.5, 129.4, 128.50, 128.47, 128.42, 128.39, 128.33, 128.29, 128.26, 128.24, 128.0, 127.91, 127.89, 127.80, 127.7, 127.6, 127.5, 127.41, 127.38, 127.36, 127.0, 103.0, 96.6, 93.2 (d, *J* = 6.1 Hz), 82.6, 80.1, 79.7, 79.4 (d, *J* = 7.4 Hz), 76.4, 75.3, 75.1, 74.9, 74.0, 73.5, 73.1, 73.1, 72.8, 72.5, 72.3, 71.4, 71.1, 69.6, 69.2 (d, *J* = 8.8 Hz), 68.4, 68.1, 64.4 (d, *J* = 4.4 Hz), 55.5; ^{31}P NMR (162 MHz, $CDCl_3$) δ = -1.5; HRMS (m/z): $[M-Et_3NH^+]^-$ calcd. for $C_{89}H_{88}O_{22}P^-$, 1539.5510; found, 1539.5503.

methyl 2,3,4,6-tetra-*O*-benzyl-*α*-D-galactosyl-(1→4)-2,3,6-tri-*O*-benzyl-*α*-D-glucopyranosyl-phosphoryl-(→4)-2,3,6-tri-*O*-benzyl-*α*-D-glucopyranoside triethylammonium salt (4ze)

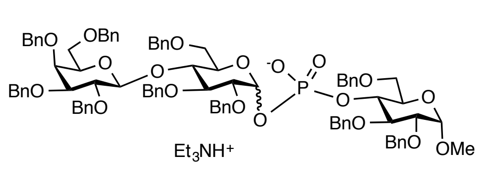

4ze, prepared first via procedure (A), purified by flash chromatography by eluting with Hexanes/EtOAc/Et_3N (5/1/0.01 to 3/1/0.01) to give an intermediate product (R_f= 0.2 (Hexanes/EtOAc, 3/1), 58.8 mg, 49% yield, α/β = 2.8/1); then procedure (B), quantitative yield, colorless oil. ^{1}H NMR (400 MHz, $CDCl_3$) δ 7.51-7.05 (m, 50H, Ar*H*), 5.89 (dd, *J* = 8.4, 3.5 Hz, 1H), 5.23 (d, *J* = 11.5 Hz, 1H, C*H*Ph), 5.02-4.93 (m, 2H, 2 x C*H*Ph), 4.85-4.29 (m, 19H, 16 x C*H*Ph, H-1^A, H-1^C, H-4^A), 4.20 (d, *J* = 11.8 Hz, 1H, C*H*Ph), 4.13-3.30 (m, 17H, H-2^A, H-3^A, H-5^A, H-6^A, H-$6'^A$, H-2^B, H-3^B, H-4^B, H-5^B, H-6^B, H-$6'^B$, H-2^C, H-3^C, H-4^C, H-5^C, H-6^C, H-$6'^C$,CH_3); ^{13}C NMR (101 MHz, $CDCl_3$) δ = 140.3, 139.7, 139.3, 139.2, 139.0, 138.9, 138.7, 138.5, 138.4, 138.3, 128.45, 128.41, 128.38, 128.32, 128.25, 128.20, 128.15, 128.11, 128.08, 128.05, 128.01, 127.98, 127.95, 127.88, 127.85, 127.82, 127.75, 127.71, 127.61, 127.59, 127.56, 127.53, 127.51, 127.45, 127.41, 127.37, 127.04, 126.97, 126.87, 103.2, 98.0, 93.3 (d, *J* = 5.9 Hz), 82.6, 81.4, 80.1, 79.9, 79.3, 75.3, 75.2, 74.9, 74.8, 74.7, 73.9, 73.5, 73.4, 73.2, 73.2, 73.1, 72.8, 72.7, 72.6, 71.3, 70.5, 69.8, 68.3, 68.2, 55.1; ^{31}P NMR (162 MHz, $CDCl_3$) δ = -2.2 (α), -3.0 (β); HRMS (m/z): [M-Et_3NH^+]$^-$ calcd. for $C_{89}H_{94}O_{19}P^-$, 1497.6132; found, 1497.6132.

methyl 2,3,4,6-tetra-*O*-benzyl-*α*-D-galactosyl-(1→4)-2,3,6-tri-*O*-benzyl-*α*-D-glucopyranosyl-phosphoryl-(→4)-2,3,6-tri-*O*-benzoyl-*α*-D-galactopyranoside triethylammonium salt (4zf)

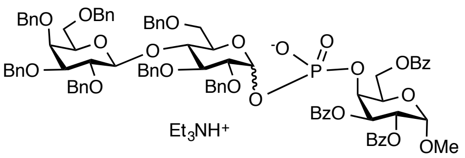

4zf, prepared first via procedure (A), purified by flash chromatography by eluting with Hexanes/EtOAc/Et_3N (5/1/0.01 to 7/2/0.01) to give an intermediate product (R_f= 0.2 (Hexanes/EtOAc, 3/1), 70.2 mg, 57% yield, α/β = 4.6/1); then procedure (B), quantitative yield, colorless oil. ^{1}H NMR (400 MHz, $CDCl_3$) δ 8.19-7.02 (m, 50H, Ar*H*), 5.79 (dd, *J* = 7.9, 3.6 Hz, 1H, H-1^B), 5.69-5,61 (m, 2H, H-2^A, H-3^A), 5.16 (d, *J* = 3.2 Hz, 1H, H-1^A), 5.08-5.05 (m, 2H, H-4^A, C*H*Ph), 4.99-4.23 (m, 17H, H-1^C, H-6^A, H-$6'^A$, 14 x C*H*Ph), 4.16-3.30 (m, 16H, H-5^A, H-2^B, H-3^B, H-4^B, H-5^B, H-6^B, H-$6'^B$, H-2^C, H-

3[C], H-4[C], H-5[C], H-6[C], H-6'[C], CH_3); ^{13}C NMR (101 MHz, $CDCl_3$) δ = 166.5, 166.24, 166.20, 139.8, 139.3, 139.1, 138.9, 138.8, 138.4, 133.2, 132.8, 132.6, 130.9, 130.5, 130.3, 129.9, 129.81, 129.77, 128.49, 128.45, 128.39, 128.30, 128.28, 128.24, 128.22, 128.19, 128.15, 128.11, 128.0, 127.93, 127.90, 127.83, 127.78, 127.73, 127.70, 127.5, 127.44, 127.39, 127.35, 127.32, 126.9, 103.0, 97.2, 93.5 (d, *J* = 6.0 Hz), 82.6, 80.1, 80.0, 79.6 (d, *J* = 8.0 Hz), 76.4, 75.4, 75.3, 74.8, 74.1, 73.5, 73.1, 72.8, 72.8, 71.6, 71.5, 69.7, 69.6, 68.6, 68.4, 68.3, 65.2, 55.1; ^{31}P NMR (162 MHz, $CDCl_3$) δ = -0.9 (α), -2.2 (β); HRMS (m/z): $[M-Et_3NH^+]^-$ calcd. for $C_{89}H_{88}O_{22}P^-$, 1539.5510; found, 1539.5500

dibenzyl 2,3,4,6-tetra-*O*-benzoyl-*α*-D-mannosyl-(1→4)-(2,3,4,6-tetra-*O*-benzoyl-*α*-D-mannosyl-(1→6))-2,3-di-*O*-benzyl-*α*-D-glucopyranosyl phosphate (4zg)

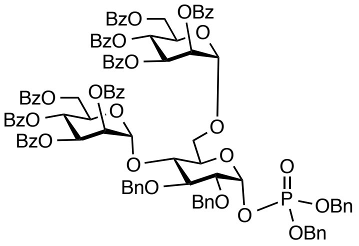

4zg, prepared first via procedure (A), purified by flash chromatography by eluting with Hexanes/EtOAc/Et_3N (5/1/0.01 to 3/2/0.01) to give the product (R_f = 0.2 (Hexanes/EtOAc, 3/2), 59.5 mg, 45% yield, α/β = 1.1/1); α anomer: $[\alpha]_D^{25}$ = -5.9 (*c* 1.1 $CHCl_3$); ^{1}H NMR (400 MHz, $CDCl_3$) δ 8.11-6.89 (m, 60H, Ar*H*), 6.08-6.17 (m, 2H), 6.00 (dd, *J* = 6.8, 3.1 Hz, 1H, H-1[A]), 5.91 (dd, *J* = 10.1, 3.2 Hz, 1H), 5.86 (dd, *J* = 10.2, 3.3 Hz, 1H), 5.83-5.80 (m, 2H), 5.69 (d, *J* = 1.9 Hz, 1H, H-1), 5.25-5.19 (m, 2H, 2 x C*H*Ph), 5.16 (d, *J* = 1.8 Hz, 1H), 5.14-5.00 (m, 3H, 3 x C*H*Ph), 4.84-4.59 (m, 7H, 6 x C*H*Ph), 4.52 (dt, *J* = 10.0, 3.6 Hz, 1H), 4.43 (dd, *J* = 12.3, 3.7 Hz, 1H); ^{13}C NMR (101 MHz, $CDCl_3$) δ 166.2, 166.1, 165.7, 165.7, 165.6, 165.4, 165.0, 138.2, 137.5, 136.0, 133.5, 133.42, 133.37, 133.33, 133.2, 133.10, 133.07, 130.3, 130.1, 130.0, 129.92, 129.87, 129.85, 129.6, 129.5, 129.4, 129.3, 129.21, 129.18, 128.8, 128.7, 128.62, 128.58, 128.54, 128.49, 128.41, 128.37, 128.32, 128.25, 128.16, 128.0, 127.9, 127.7, 127.4, 99.7, 98.7, 95.0 (d, *J* = 6.4 Hz), 80.1, 79.9 (d, *J* = 7.2 Hz), 76.3, 75.3, 73.2, 72.1, 70.5, 70.41, 70.36, 70.2, 70.0, 69.8, 69.70, 69.66, 69.60, 69.5, 67.3, 67.2, 66.7, 63.3,

62.8; ^{31}P NMR (162 MHz, $CDCl_3$) δ = -2.3; HRMS (m/z): $[M+Na^+]^+$ calcd. for $C_{102}H_{89}O_{27}PNa^+$, 1799.5221; found, 1799.5220.

1.3.3 Procedure (C) for donor synthesis and characterization

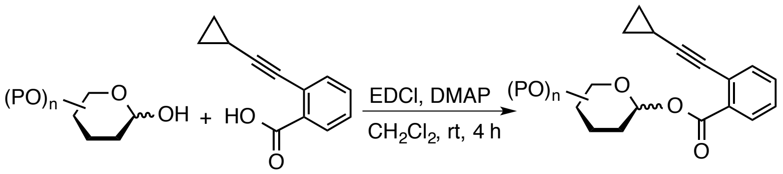

General procedure (C) for the synthesis of glycosyl *ortho*-alkynylbenzoate: to a solution of protected glycose (1.0 mmol) in CH_2Cl_2 (10 mL) was added *ortho*-alkynyl benzoic acid (279 mg, 1.5 mmol), DMAP (24 mg, 0.2 mmol) and 1-ethyl-3-(3-dimethylaminopropyl)carbodiimide hydrochloride (EDC) (344 mg, 1.8 mmol), respectively. After stirred for 4 h, the reaction mixture was diluted with EtOAc, washed with H_2O, saturated $NaHCO_3$ and brine, dried over Na_2SO_4. After filtration, the filtrate was concentrated to give a residue. The residue was purified via flash silica gel chromatography (Hexanes/EtOAc, 20/1 → 10/1) to afford the α/β mixed donors.

2,3,4-tri-*O*-benzyl-*α/β*-D-xylopyranosyl *ortho*-cyclopropylethynylbenzoate (1b)

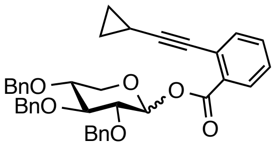

1b, prepared via procedure (C), R_f= 0.2 (Hexanes/EtOAc, 12/1), colorless oil, 553 mg, 93% yield, α/β = 1/2. α-anomer: ^{1}H NMR (400 MHz, $CDCl_3$) δ 7.97-7.20 (m, 19H, Ar*H*), 6.52 (d, *J* = 3.6 Hz, 1H), 4.95-4.62 (m, 6H, 3 x CH_2Ph), 4.05-3.99 (m, 2H, H-4, H-5), 3.85-3.65 (m, 1H, H-2), 3.55-3.44 (m, 2H, H-3, H-5')1.55-1.47 (m, 1H, CH_2C*H*CH_2), 0.87-0.77 (m, CH_2CHCH_2); β-anomer: ^{1}H NMR (400 MHz, $CDCl_3$) δ 7.97-7.20 (m, 19H, Ar*H*), 5.87 (d, *J* = 7.3 Hz, 1H), 4.95-4.62 (m, 6H, 3 x CH_2Ph), 3.85-3.65 (m, 4H, H-2, H-3, H-4, H-5), 1.55-1.47 (m, 1H, CH_2C*H*CH_2), 0.87-0.77 (m, CH_2CHCH_2); mixture of α and β anomers ^{13}C NMR (101 MHz, $CDCl_3$) δ = 164.8, 164.2, 138.9, 138.6, 138.2, 138.1, 138.0, 137.9, 134.7, 134.5, 132.2, 132.1, 131.1, 130.9, 130.6, 130.5, 128.61, 128.57, 128.52, 128.50, 128.47, 128.4, 128.2, 128.1, 128.04, 128.01, 127.97, 127.9, 127.8, 127.7, 127.14, 127.06, 125.5, 125.2, 100.2, 100.0, 95.2, 91.0, 83.5, 81.4, 80.3, 78.9, 77.59, 77.56, 75.8, 75.6, 75.0, 74.9, 74.6, 73.8, 73.4, 73.3, 64.6, 62.7, 9.1, 9.03, 9.01, 0.9; HRMS (m/z): $[M+Na^+]^+$ calcd. for $C_{38}H_{36}NaO_6^+$, 611.2404; found, 611.2400.

6-*O*-acetyl-2,3,4-tri-*O*-benzyl-*α/β*-D-glucopyranosyl *ortho*-cyclopropylethynylbenzoate (1c)

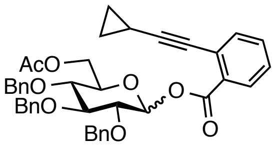

1c, prepared via procedure (C), R_f= 0.2 (Hexanes/EtOAc, 8/1), colorless oil, 614 mg, 93% yield, α/β = 1/1.3. α anomer: ^{1}H NMR (400 MHz, $CDCl_3$) δ 7.97-7.21 (m, 19H, Ar*H*), 6.64 (d, *J* = 3.6 Hz, 1H), 5.02-4.56 (m, 6H, 6 x C*H*Ph), 4.37-4.25 (m, 2H, H-6, H-6'), 4.19-4.12 (m, 1H, H-3), 3.86-3.61 (m, 3H, H-2, H-4, H-5), 2.03 (s, 3H, CH_3), 1.57-1.49 (m, 1H, $CH_2C\mathit{H}CH_2$), 0.85-0.76 (m, 4H, $C\mathit{H}_2CHC\mathit{H}_2$); β anomer: ^{1}H NMR (400 MHz, $CDCl_3$) δ 7.97-7.21 (m, 19H, Ar*H*), 5.92 (d, *J* = 7.9 Hz, 1H), 5.02-4.56 (m, 6H, 6 x C*H*Ph), 4.37-4.25 (m, 2H, H-6, H-6'), 4.19-4.12 (m, 1H, H-3), 3.86-3.61 (m, 3H, H-2, H-4, H-5), 2.03 (s, 3H, CH_3), 1.57-1.49 (m, 1H, $CH_2C\mathit{H}CH_2$), 0.85-0.76 (m, 4H, $C\mathit{H}_2CHC\mathit{H}_2$); mixture of α and β anomers: ^{13}C NMR (101 MHz, $CDCl_3$) δ = 170.8, 164.6, 164.0, 138.7, 138.4, 137.89, 137.85, 137.77, 137.74, 134.8, 134.5, 132.3, 132.2, 131.02, 130.96, 130.8, 130.5, 128.7, 128.60, 128.57, 128.50, 128.30, 128.26, 128.16, 128.14, 128.08, 128.04, 128.02, 127.9, 127.8, 127.2, 127.1, 125.6, 125.2, 100.3, 100.1, 94.5, 90.7, 85.0, 81.9, 75.9, 75.4, 75.1, 75.0, 74.6, 73.9, 73.2, 71.7, 63.0, 62.8, 21.0, 9.16, 9.12, 9.05, 9.02, 0.90, 0.87; HRMS (m/z): $[M+Na^+]^+$ calcd. for $C_{41}H_{40}NaO_8^+$, 683.2615; found, 683.2611.

methyl-2,3,4-tri-*O*-benzyl-*α/β*-D-glucopyranuronatosyl *ortho*cyclopropylethynylbenzoate (1d)

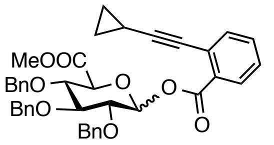

1d, prepared via procedure (C), R_f= 0.2 (Hexanes/EtOAc, 8/1), colorless oil, 595 mg, 92% yield, α/β = 1/2. α-anomer: ^{1}H NMR (400 MHz, $CDCl_3$) δ 7.97-7.24 (m, 19H, Ar*H*), 6.65 (d, *J* = 3.6 Hz, 1H), 4.98-4.60 (m, 6H, 6 x C*H*Ph), 4.17-3.78 (m, 4H, H-2, H-3, H-4, H-5), 3.71 (s, 3H, OMe), 1.61-1.49 (m, 1H, $CH_2C\mathit{H}CH_2$), 0.90-0.89 (m, 4H, $C\mathit{H}_2CHC\mathit{H}_2$); β-anomer: ^{1}H NMR (400 MHz, $CDCl_3$) δ 7.97-7.24 (m, 19H, Ar*H*), 5.96 (d, *J* = 7.2 Hz, 1H), 4.98-4.60 (m, 6H, 6 x C*H*Ph), 4.17-3.78 (m, 4H, H-2, H-3, H-4, H-5), 3.68 (s, 3H, OMe), 1.61-1.49 (m, 1H, $CH_2C\mathit{H}CH_2$), 0.90-0.89 (m, 4H, $C\mathit{H}_2CHC\mathit{H}_2$); α and β anomers: ^{13}C NMR (101 MHz, $CDCl_3$) δ = 168.7, 168.2, 163.5, 162.9, 137.7, 137.4, 137.02, 136.96, 136.90, 136.7, 134.0, 133.7, 131.5, 131.4, 130.2, 130.0, 129.8, 129.3, 127.73, 127.70, 127.67, 127.6, 127.4, 127.34, 127.27, 127.23, 127.20, 127.18, 127.10, 127.07, 126.99, 126.3, 126.2, 124.9, 124.5, 99.6, 93.6, 89.9, 83.1, 80.4, 79.9,

78.39, 78.37, 77.8, 75.1, 75.0, 74.7, 74.4, 74.22, 74.18, 73.7, 72.5, 72.0, 51.8, 8.35, 8.29, 8.23, 8.20, 0.1, -0.0; HRMS (m/z): [M+Na$^+$]$^+$ calcd. for $C_{40}H_{38}NaO_8^+$, 669.2459; found, 669.2458.

2,3,4,6-tetra-*O*-benzyl-*α/β*-D-galactopyranosyl *ortho*-cyclopropylethynylbenzoate (1e)

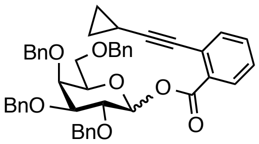

1e, prepared via procedure (C), the α/β mixture can be separated by gel silica chromatography, 701 mg, 99% yield, α/β = 1/3. α-anomer: R_f= 0.3 (Hexanes/EtOAc, 10/1), $[\alpha]_D^{25}$ = 57.7 (*c* 1.4, $CHCl_3$); ^{1}H NMR (400 MHz, $CDCl_3$) δ 7.88-7.23 (m, 20H, Ar*H*), 6.67 (d, *J* = 3.6 Hz, 1H, H-1), 4.99 (d, *J* = 11.3 Hz, 1H, C*H*Ph), 4.85 (d, *J* = 11.9 Hz, 1H, C*H*Ph), 4.81-4.74 (m, 2H, 2 x C*H*Ph), 4.70 (d, *J* = 11.6 Hz, 1H), 4.61 (d, *J* = 11.3 Hz, 1H, C*H*Ph), 4.50-4.37 (m, 2H, 2 x C*H*Ph), 4.31-4.23 (m, 2H, H-2, H-5), 4.15-4.03 (m, 2H, H-3, H-4), 3.63 (t, *J* = 8.5 Hz, 1H, H-6), 3.56 (dd, *J* = 9.0, 5.4 Hz, 1H, H-6'), 1.45-1.38 (m, 1H, CH_2C*H*CH_2), 0.87-0.74 (m, 4H, CH_2CH_2); ^{13}C NMR (101 MHz, $CDCl_3$) δ = 164.7, 138.8, 138.7, 138.3, 138.0, 134.7, 131.9, 131.5, 130.9, 128.52, 128.47, 128.40, 128.3, 128.1, 128.0, 127.9, 127.8, 127.7, 127.62, 127.58, 127.2, 125.1, 99.5, 91.8, 78.7, 75.8, 75.09, 75.85, 74.9, 73.7, 73.2, 73.1, 72.3, 68.4, 9.1, 9.1, 0.8; HRMS (m/z): [M+Na$^+$]$^+$ calcd. for $C_{46}H_{44}NaO_7^+$, 731.2979; found, 731.2977. β-anomer: R_f= 0.2 (Hexanes/EtOAc, 10/1), $[\alpha]_D^{25}$ = -10.9 (*c* 2.6, $CHCl_3$); ^{1}H NMR (400 MHz, $CDCl_3$) δ 7.93-7.19 (m, 20H, Ar*H*), 5.84 (d, *J* = 8.1 Hz, 1H, H-1), 4.96 (d, *J* = 11.3 Hz, 1H, C*H*Ph), 4.83-4.75 (m, 4H, 4 x C*H*Ph), 4.63 (d, *J* = 11.4 Hz, 1H, C*H*Ph), 4.50-4.38 (m, 2H, 2 x C*H*Ph), 4.10 (dd, *J* = 9.8, 8.0 Hz, 1H, H-2), 4.03 (d, *J* = 2.8 Hz, 1H, H-4), 3.79 (dd, *J* = 7.9, 5.4 Hz, 1H, H-5), 3.73-3.65 (m, 2H, H-3, H-6), 3.62 (dt, *J* = 9.0, 3.7 Hz, 1H, H-6'); ^{13}C NMR (101 MHz, $CDCl_3$) δ = 163.9, 138.4, 138.1, 138.0, 137.6, 134.0, 131.8, 130.7, 130.3, 128.3, 128.2, 128.1, 128.0, 127.9, 127.8, 127.6, 127.5, 127.4, 126.7, 125.3, 82.3, 78.0, 75.1, 74.7, 74.4, 74.0, 73.3, 73.2, 72.7, 67.7, 8.7, 8.7, 0.5; HRMS (m/z): [M+Na$^+$]$^+$ calcd. for $C_{46}H_{44}NaO_7^+$, 731.2979; found, 731.3002.

2,3,4,6-tetra-*O*-benzyl-*α/β*-D-mannopyranosyl *ortho*-cyclopropylethynylbenzoate (1f)

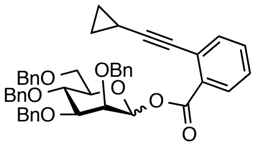

1f, prepared via procedure (C), R_f= 0.2 (Hexanes/EtOAc, 10/1), colorless oil, 638 mg, 90% yield, α/β = 2/1. α anomer: ^{1}H NMR (400 MHz, $CDCl_3$) δ 7.81-7.13 (m, 20H, Ar*H*), 6.53 (d, *J* = 2.0 Hz, 1H, H-1), 4.94-4.53 (m, 8H, C*H*$_2$Ph x 4), 4.19 (t, *J* = 9.7 Hz, 1H, H-4), 4.09 (dd, *J* = 9.5, 3.0 Hz, 1H, H-3), 4.07-4.03 (m, 1H, H-5), 3.90 (dd, *J* = 3.1, 2.1 Hz, 1H, H-2), 3.85 (dd, *J* = 11.1, 4.2 Hz, 1H, H-6), 3.75 (dd, *J* = 11.2, 1.9 Hz, 1H, H-6'), 1.57-1.47 (m, 1H, CH_2C*H*CH_2), 0.81-0.76 (m, 4H, C*H*$_2$CHC*H*$_2$); β anomer: ^{1}H NMR (400 MHz, $CDCl_3$) δ 8.07-7.12 (m, 24H, Ar*H*), 5.90 (d, *J* = 1.0 Hz, 1H), 4.93-4.53 (m, 8H, 8 x C*H*Ph), 4.22-4.03 (m, 2H, H-3, H-4), 3.90-3.65 (m, 4H, H-2, H-5, H-6, H-6'); mixture of α and β anomers: ^{13}C NMR (101 MHz, $CDCl_3$) δ = 164.4, 164.0, 161.9, 138.6, 138.5, 138.44, 138.41, 138.36, 138.29, 138.17, 138.06, 134.7, 134.5, 134.4, 133.0, 132.21, 132.17, 131.7, 130.92, 130.88, 130.8, 130.3, 128.6, 128.5, 128.42, 128.38, 128.31, 128.2, 128.13, 128.11, 128.06, 128.02, 127.9, 127.83, 127.76, 127.74, 127.63, 127.61, 127.2, 127.1, 127.0, 126.1, 125.6, 125.0, 101.2, 100.3, 100.2, 94.0, 92.9, 82.2, 79.4, 76.7, 75.4, 75.2, 75.01, 75.96, 74.5, 74.33, 74.29, 74.1, 73.8, 73.6, 73.5, 72.6, 72.3, 72.25, 69.2, 69.0, 9.13, 9.08, 9.03, 8.99, 0.8, 0.7; HRMS (m/z): $[M+Na^+]^+$ calcd. for $C_{46}H_{44}NaO_7^+$, 731.2979; found, 731.3002.

3,4-di-*O*-acetyl-2-azido-6-*O*-dibenzyloxyphosphoryl-2-deoxy-*α/β*-D-mannopyranosyl *ortho*-cyclopropylethynylbenzoate (1g)

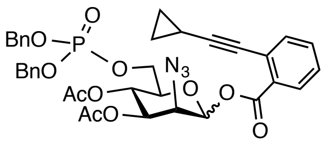

1g, prepared via procedure (C), the α/β mixture can be separated by gel silica chromatography, 631 mg, 99% yield, α/β = 4/1. α-anomer: R_f= 0.3 (Hexanes/EtOAc, 3/2), $[\alpha]_D^{25}$ = -35.6 (*c* 1.1 $CHCl_3$); ^{1}H NMR (400 MHz, $CDCl_3$) δ 7.90-7.31 (m, 14H, Ar*H*), 6.36 (d, *J* = 1.8 Hz, 1H, H-1), 5.56 (dd, *J* = 9.9, 3.7 Hz, 1H, H-3), 5.48 (t, *J* = 9.9 Hz, 1H, H-4), 5.14-4.93 (m, 4H, 2 x C*H*$_2$Ph), 4.32-4.19 (m, 2H, H-2, H-5), 4.17-4.09 (m, 2H, H-6, H-6'), 2.11 (s, 3H, CH_3), 2.00 (s, 3H, CH_3), 1.57-1.51 (m, 1H, CH_2C*H*CH_2), 0.89-0.86 (m, 4H, C*H*$_2$CHC*H*$_2$); ^{13}C NMR (101 MHz, $CDCl_3$) δ = 169.3, 168.6, 162.9, 135.1, 134.3, 131.9, 130.1, 129.1, 127.9, 127.80, 127.77, 127.4, 127.3, 126.7, 124.5, 99.7, 91.6, 73.9, 71.1 (d, *J* = 7.9 Hz), 70.1, 68.9 (d, 2^{13}C, *J* = 5.9 Hz), 64.9, 64.7 (d, *J* = 4.9 Hz), 60.2, 19.95, 19.86, 0.0; ^{31}P NMR (162 MHz, $CDCl_3$) δ = -1.5; HRMS (m/z): $[M+H^+]^+$ calcd. for $C_{36}H_{37}N_3O_{11}P^+$, 718.2160; found, 718.2158.

β-anomer: R_f= 0.2 (Hexanes/EtOAc, 3/2), $[\alpha]_D^{25}$ = 53.4 (*c* 4.2 $CHCl_3$); ^{1}H NMR (400 MHz, $CDCl_3$) δ 7.92-7.22 (m, 14H, Ar*H*), 6.09 (d, *J* = 1.4 Hz, 1H, H-1), 5.33 (t, *J* = 9.8

Hz, 1H, H-4), 5.12 (dd, *J* = 9.8, 3.6 Hz, 1H, H-3), 5.08-4.94 (m, 4H, 2 x C*H*$_2$Ph), 4.27 (dd, *J* = 3.7, 1.4 Hz, 1H, H-2), 4.19-4.02 (m, 2H, H-6, H-6'), 3.83-3.72 (m, 1H, H-5), 2.13 (s, 3H, CH_3), 2.03 (s, 3H, CH_3), 1.54-1.43 (m, 1H, CH_2C*H*CH_2), 0.93-0.79 (m, 4H, C*H*$_2$CHC*H*$_2$); ^{13}C NMR (101 MHz, $CDCl_3$) δ = 169.4, 168.7, 162.4, 135.2, 135.14, 135.10, 133.8, 132.0, 130.2, 128.6, 127.9, 127.86, 127.79, 127.35, 127.31, 126.4, 125.2, 100.0, 90.9, 73.5, 73.2 (d, *J* = 7.8 Hz), 71.2, 68.9 (d, *J* = 5.9 Hz), 68.8 (d, *J* = 5.9 Hz), 64.7 (d, *J* = 5.2 Hz), 64.5, 60.7, 20.0, 19.97, 19.94, 0.0; ^{31}P NMR (162 MHz, $CDCl_3$) δ = -1.6; HRMS (m/z): $[M+H^+]^+$ calcd. for $C_{36}H_{37}N_3O_{11}P^+$, 718.2160; found, 718.2161.

2,3,4-tri-*O*-benzyl-*α/β*-D-rhamnopyranosyl *ortho*-cyclopropylethynylbenzoate (1h)

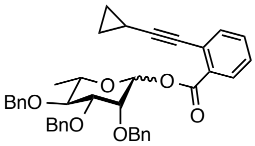

1h, prepared via procedure (C), R_f = 0.2 (Hexanes/EtOAc, 10/1), colorless oil, 597 mg, 99% yield, α/β = 2/1. α anomer: ^{1}H NMR (400 MHz, $CDCl_3$) δ 8.08-7.20 (m, 15H, Ar*H*), 6.41 (d, *J* = 1.9 Hz, 1H, H-1), 4.98 (d, *J* = 10.7 Hz, 1H, C*H*Ph), 4.87-4.74 (m, 2H, C*H*$_2$Ph), 4.66 (d, *J* = 10.6 Hz, 1H, C*H*Ph), 4.61-4.56 (m, 2H, C*H*$_2$Ph), 4.04 (dd, *J* = 9.6, 3.3 Hz, 1H, H-4), 4.01-3.97 (m, 1H, H-5), 3.89 (dd, *J* = 3.2, 2.0 Hz, 1H, H-2), 3.75 (d, *J* = 9.5 Hz, 1H, H-3), 1.57-1.50 (m, 1H, CH_2C*H*CH_2), 1.39 (d, *J* = 6.2 Hz, 3H, C*H*$_3$), 0.81-.073 (m, 4H, C*H*$_2$CHC*H*$_2$); β anomer: ^{1}H NMR (400 MHz, $CDCl_3$) δ 7.95-7.20 (m, 19H, Ar*H*), 5.85 (s, 1H, H-1), 4.99-4.58 (m, 6H, 6 x C*H*Ph), 4.09-3.53 (m, 4H, H-2, H-3, H-4, H-5), 1.42-1.33 (m, 1H, CH_2C*H*CH_2), 0.90-0.73 (m, 4H, C*H*$_2$CHC*H*$_2$); mixture of α and β anomers: ^{13}C NMR (101 MHz, $CDCl_3$) δ = 164.5, 164.0, 161.9, 138.6, 138.4, 138.3, 138.2, 138.0, 134.7, 134.6, 134.4, 133.0, 132.25, 132.16, 131.7, 130.9, 130.8, 130.3, 128.6, 128.54, 128.49, 128.46, 128.3, 128.24, 128.19, 128.13, 127.92, 127.90, 127.86, 127.82, 127.78, 127.76, 127.71, 127.3, 127.2, 127.0, 126.1, 125.6, 125.0, 101.2, 100.3, 100.1, 93.9, 92.8, 82.4, 80.0, 79.9, 79.4, 75.7, 75.6, 74.9, 74.5, 74.3, 74.0, 73.1, 72.7, 72.3, 72.2, 71.1, 18.2, 18.1, 9.15, 9.12, 9.09, 9.0; HRMS (m/z): $[M+Na^+]^+$ calcd. for $C_{39}H_{38}NaO_6^+$, 625.2561; found, 625.2564.

2,3,4-tri-*O*-benzoyl-*α/β*-D-fucopyranosyl *ortho*-cyclopropylethynylbenzoate (1i)

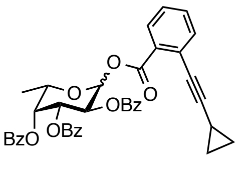

1i, prepared via procedure (C), R_f= 0.2 (Hexanes/EtOAc, 4/1), colorless oil, 554 mg, 86% yield, α/β = 1/1. Mixture of α and β anomers: ^{1}H NMR (400 MHz, $CDCl_3$) δ 8.15-7.24 (m, 30H, ArH^{α}, ArH^{β}), 6.92 (d, J = 3.6 Hz, 1H, H-1$^{\alpha}$), 6.25 (d, J = 8.3 Hz, 1H, H-1$^{\beta}$), 6.11 (dd, J = 10.7, 3.3 Hz, 1H, H-3$^{\beta}$), 6.06-5.97 (m, 2H, H-1$^{\alpha}$, H-1$^{\beta}$), 5.91 (dd, J = 3.3, 1.3 Hz, 1H, H-4$^{\beta}$), 5.80 (dd, J = 3.5, 1.1 Hz, 1H, H-4$^{\alpha}$), 5.69 (dd, J = 10.4, 3.5 Hz, 1H, H-3$^{\alpha}$), 4.92-4.72 (m, 1H, H-5$^{\beta}$), 4.43-4.23 (m, 1H, H-5$^{\alpha}$), 1.61-1.50 (m, 2H, $CH_2C\mathit{H}CH_2^{\alpha}$, $CH_2C\mathit{H}CH_2^{\beta}$), 1.39 (d, J = 6.4 Hz, 3H, H-6$^{\alpha}$), 1.34 (d, J = 6.5 Hz, 3H, H-6$^{\beta}$), 0.91-0.83 (m, 8H, $C\mathit{H}_2CHC\mathit{H}_2^{\alpha}$, $C\mathit{H}_2CHC\mathit{H}_2^{\beta}$); ^{13}C NMR (101 MHz, $CDCl_3$) δ = 165.2, 165.0, 164.9, 164.7, 163.7, 162.9, 134.3, 133.6, 132.82, 132.78, 132.6, 132.54, 132.49, 131.6, 131.5, 130.2, 130.1, 129.3, 129.2, 129.1, 129.08, 129.05, 129.01, 128.8, 128.5, 128.4, 128.3, 128.2, 128.1, 127.94, 127.87, 127.7, 127.64, 127.58, 126.6, 126.3, 125.0, 124.3, 99.7, 99.2, 92.1, 90.4, 74.1, 73.6, 71.4, 70.8, 70.3, 70.2, 68.5, 68.1, 67.5, 66.9, 15.55, 15.49, 8.3, 8.2, 0.0, -0.1; HRMS (m/z): [M+Na$^+$]$^+$ calcd. for $C_{39}H_{32}NaO_9^+$, 667.1939; found, 667.1936.

2-azido-3,4,6-tri-*O*-benzyl-2-deoxy-*α/β*-D-glucopyranosyl *ortho*-cyclopropylethynylbenzoate (1j)

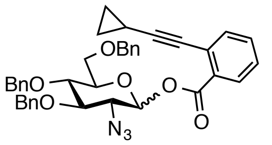

1j, prepared via procedure (C), R_f= 0.2 (Hexanes/EtOAc, 8/1), colorless oil, 611 mg, 95% yield, α/β = 1/3. β-anomer: ^{1}H NMR (400 MHz, $CDCl_3$) δ 8.02-7.12 (m, 15H, ArH), 5.72 (d, J = 8.5 Hz, 1H), 4.93-4.47 (m, 6H, $C\mathit{H}_2$Ph x 3), 3.83 (t, J = 9.3 Hz, 1H, H-4), 3.79-3.69 (m, 3H, H-2, H-6, H-6'), 3.65 -3.56 (m, 2H, H-3, H-5), 1.55-1.47 (m, 1H, CH), 0.89-0.83 (m, 4H, $C\mathit{H}_2C\mathit{H}_2$); α-anomer: ^{1}H NMR (400 MHz, $CDCl_3$) δ 7.95-7.12 (m, 15H, ArH), 6.56 (d, J = 3.6 Hz, 1H, H-1), 4.20-4.06 (m, 2H, H-3, H-5), 3.91 (t, J = 9.6 Hz, 1H, H-4), 3.81-3.58 (m, 3H, H-2, H-6, H-6'); mixture of α and β anomers: ^{13}C NMR (101 MHz, $CDCl_3$) δ = 164.3, 163.8, 138.00, 137.93, 137.90, 134.9, 134.6, 132.5, 132.3, 131.0, 130.9, 130.1, 128.63, 128.59, 128.55, 128.50, 128.24, 128.18, 128.09, 128.07, 128.02, 127.96, 127.90, 127.8, 127.2, 127.1, 125.8, 125.2, 100.4, 100.3, 93.5, 91.7, 83.5, 80.9, 76.0, 75.8, 75.7, 75.3, 75.2, 74.6, 73.8, 73.74, 73.69, 68.1, 68.0, 65.6, 63.3, 9.1, 0.85; HRMS (m/z): [M+Na$^+$]$^+$ calcd. for $C_{39}H_{37}N_3NaO_6^+$, 666.2575; found, 666.2574.

2-azido-3,4,6-tri-*O*-benzyl-2-deoxy-*α/β*-D-galactopyranosyl *ortho*-cyclopropylethynylbenzoate (1k)

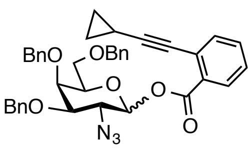

1k, prepared via procedure (C), R_f = 0.2 (Hexanes/EtOAc, 8/1), colorless oil, 618 mg, 96% yield, α/β = 1/3. α-anomer: ^{1}H NMR (400 MHz, $CDCl_3$) δ 8.00-7.25 (m, 19H, Ar*H*), 6.50 (d, *J* = 3.4 Hz, 1H, H-1), 4.94-4.40 (m, 6H, 6 x C*H*Ph), 4.26-4.08 (m, 4H, H-2, H-3, H-4, H-5), 3.76-3.49 (m, 2H, H-6, H-6'), 1.49-1.42 (m, 1H, CH_2CHCH_2), 0.86-0.76 (m, 4H, CH_2CHCH_2); β anomer: ^{1}H NMR (400 MHz, $CDCl_3$) δ 8.00-7.25 (m, 19H, Ar*H*), 4.94-4.40 (m, 6H, 6 x C*H*Ph), 5.65 (d, *J* = 8.5 Hz, 1H, H-1), 4.13-4.09 (m, 2H, H-3), 4.01 (d, *J* = 2.7 Hz, 1H, H-4), 3.76-3.49 (m, 4H, H-2, H-5, H-6, H-6'), 1.49-1.42 (m, 1H, CH_2CHCH_2), 0.86-0.76 (m, 4H, CH_2CHCH_2); mixture of α and β anomer: ^{13}C NMR (101 MHz, $CDCl_3$) δ = 164.3, 164.0, 138.4, 137.81, 137.78, 137.5, 134.9, 134.4, 132.4, 132.2, 131.1, 130.8, 130.1, 128.7, 128.68, 128.60, 128.56, 128.40, 128.35, 128.18, 128.16, 128.10, 128.08, 128.04, 127.99, 127.91, 127.86, 127.2, 127.1, 125.7, 125.1, 100.3, 99.8, 93.7, 92.0, 81.1, 78.0, 75.1, 75.0, 74.6, 74.4, 73.73, 73.70, 73.0, 72.7, 72.2, 72.1, 68.0, 67.8, 62.3, 59.6, 9.0, 0.8; HRMS (m/z): $[M+Na^+]^+$ calcd. for $C_{39}H_{37}N_3NaO_6^+$, 666.2575; found, 666.2575.

3,4,6-tri-*O*-benzyl-2-fluoro-2-deoxy-*α/β*-D-glucopyranosyl *ortho*-cyclopropylethynylbenzoate (1l)

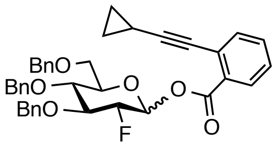

1l, prepared via procedure (C), R_f = 0.2 (Hexanes/EtOAc, 10/1), colorless oil, 558 mg, 90% yield, α/β = 1/2.3. α-anomer: ^{1}H NMR (400 MHz, $CDCl_3$) δ 8.01-7.11 (m, 19H, Ar*H*), 6.67 (d, *J* = 3.8 Hz, 1H), 4.96-4.46 (m, 7H, 3 x C*H*$_2$Ph, H-2), 4.26 (dt, *J* = 12.2, 9.2 Hz, 1H, H-3), 4.15-4.04 (m, 1H, H-5), 3.94-3.67 (m, 3H, H-4, H-6, H-6'), 1.57-1.48 (m, 1H, CH_2CHCH_2), 0.89-0.73 (m, 4H, CH_2CHCH_2); β-anomer: ^{1}H NMR (400 MHz, $CDCl_3$) δ 8.01-7.11 (m, 19H, Ar*H*), 5.96 (dd, *J* = 8.1, 3.1 Hz, 1H), 4.96-4.46 (m, 7H, 3 x C*H*$_2$Ph, H-2), 3.94-3.67 (m, 5H, H-3, H-4, H-5, H-6, H-6'), 1.57-1.48 (m, 1H, CH_2CHCH_2), 0.89-0.73 (m, 4H, CH_2CHCH_2); ^{13}C NMR (101 MHz, $CDCl_3$) δ = 164.3, 138.3, 138.1, 138.05, 137.96, 137.92, 134.8, 134.5, 132.4, 132.3, 131.05, 131.02, 130.3, 128.6, 128.5, 128.2, 128.12, 128.08, 128.04, 128.00, 127.97, 127.93, 127.90, 127.8, 127.2, 127.1, 125.6, 125.2, 100.31, 100.25, 92.4, 92.2, 91.4, 91.3, 90.4, 90.2, 89.5, 83.4, 80.9, 76.6, 76.3, 75.9, 75.5, 75.33, 75.26, 75.1, 74.9, 74.5, 73.73, 73.65, 73.2, 68.1, 68.0,

9.1, 9.0, 0.9, 0.8; ^{19}F NMR (565 MHz, $CDCl_3$) δ = -196.5, -199.0. HRMS (m/z): $[M+H^+]^+$ calcd. for $C_{39}H_{38}FO_6^+$, 621.2647; found, 621.2650.

3,4,6-tri-*O*-benzyl-2-fluoro-2-deoxy-*α/β*-D-mannopyranosyl *ortho*-cyclopropylethynylbenzoate (1m)

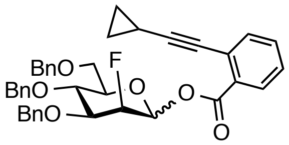

1m, prepared via procedure (C), R_f= 0.2 (Hexanes/EtOAc, 10/1), colorless oil, 576 mg, 93% yield, α/β = 3.2/1. α-anomer: ^1H NMR (400 MHz, $CDCl_3$) δ 7.81-7.63 (m, 19H, Ar*H*), 6.54 (dd, *J* = 6.2, 2.2 Hz, 1H, H-1^α), 4.89-4.51 (m, 7H, H-2^α, 3 x CH_2Ph), 4.14-4.01 (m, 2H, H-3^α, H-4^α, H-5^α), 3.85-3.64 (m, 2H, H-6^α, H-6'^{α}), 1.57-1.49 (m, 1H, CH_2CHCH_2), 0.89-0.71 (m, 4H, CH_2CHCH_2); β-anomer: ^1H NMR (400 MHz, $CDCl_3$) δ 7.81-7.63 (m, 19H, Ar*H*), 5.90 (d, *J* = 19.3 Hz, 1H, H-1^β), 4.89-4.51 (m, 7H, H-2^β, 3 x CH_2Ph), 4.14-4.01 (m, 2H, H-3^β, H-4^β, H-5^β), 3.85-3.64 (m, 2H, H-6^β, H-6' ^{β}), 1.57-1.49 (m, 1H, CH_2CHCH_2), 0.89-0.71 (m, 4H, CH_2CHCH_2); mixture of α and β anomer: ^{13}C NMR (101 MHz, $CDCl_3$) δ = 164.0, 163.9, 138.3, 138.2, 138.1, 137.8, 137.5, 134.8, 134.5, 132.5, 132.4, 131.2, 130.9, 130.3, 130.0, 128.7, 128.6, 128.53, 128.47, 128.4, 128.2, 128.13, 128.09, 128.03, 127.95, 127.9, 127.73, 127.71, 127.2, 127.1, 125.6, 125.1, 100.43, 100.35, 92.1 (d, *J* = 19 Hz)), 92.0 (d, *J* = 31 Hz), 86.4 (d, *J* = 190 Hz), 86.0 (d, *J* = 180 Hz), 80.3 (d, *J* = 17.6 Hz), 78.2 (d, *J* = 17.5 Hz), 76.5, 75.55, 75.46, 74.9, 74.7, 74.4, 74.0, 73.9, 73.7, 73.6, 72.5, 72.2, 68.6, 68.5, 9.2, 9.0, 0.82, 0.78; ^{19}F NMR (376 MHz, $CDCl_3$) δ = -203; HRMS (m/z): $[M+H^+]^+$ calcd. for $C_{39}H_{38}FO_6^+$, 621.2647; found, 621.2649.

2,3,6-tri-*O*-benzyl-4-fluoro-4-deoxy-*α/β*-D-galactopyranosyl *ortho*-cyclopropylethynylbenzoate (1n)

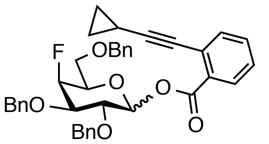

1n, prepared via procedure (C), the α/β mixture can be separated by gel silica chromatography, colorless oil, 576mg, 93% yield, α/β = 1.5/1. α-anomer: R_f= 0.25 (Hexanes/EtOAc, 10/1), $[α]_D^{25}$ = 43 (*c* 1.7 $CHCl_3$); ^1H NMR (400 MHz, $CDCl_3$) δ 7.87-7.25 (m, 19H, Ar*H*), 6.63 (d, *J* = 3.2 Hz, 1H, H-1), 5.01 (dd, *J* = 50.0, 2.3 Hz, 1H, H-4), 4.85-4.66 (m, 4H, 2 x CH_2Ph), 4.53 (s, 2H, CH_2Ph), 4.32-4.21 (m, 1H, H-5), 4.14-4.00 (m, 2H, H-2, H-3), 3.70 (t, *J* = 8.7 Hz, 1H, H-6), 3.63 (ddd, *J* = 9.2, 5.6, 1.6 Hz,

1H, H-6'); ^{13}C NMR (101 MHz, $CDCl_3$) δ = 163.9, 137.4, 137.2, 137.1, 134.1, 131.3, 130.4, 130.3, 130.2, 128.2, 127.79, 127.77, 127.74, 127.3, 127.18, 127.16, 127.1, 126.5, 124.3, 98.8, 90.7, 86.4 (d, *J* = 184 Hz), 74.9 (d, *J* = 20.0 Hz), 74.35, 74.38, 73.1, 72.8, 72.0, 70.2 (d, *J* = 18.5 Hz), 66.7 (d, *J* = 6.0 Hz), 8.33, 8.28, 0.0; ^{19}F NMR (376 MHz, $CDCl_3$) δ = -218.8; HRMS (m/z): $[M+Na^+]^+$ calcd. for $C_{39}H_{37}FNaO_6^+$, 643.2466; found, 643.2467. β-anomer: R_f = 0.2 (Hexanes/EtOAc, 10/1), $[\alpha]_D^{25}$ = -31 (*c* 2.9 $CHCl_3$); ^{1}H NMR (400 MHz, $CDCl_3$) δ 7.91-7.19 (m, 19H, Ar*H*), 5.86 (d, *J* = 8.1 Hz, 1H, H-1), 4.93 (dd, *J* = 49.7, 2.6 Hz, 1H, H-4), 4.84-4.68 (m, 4H, 2 x C*H*$_2$Ph), 4.55 (s, 2H, C*H*$_2$Ph), 4.00 (dd, *J* = 9.6, 8.1 Hz, 1H, H-2), 3.88-3.57 (m, 4H, H-3, H-5, H-6, H-6'); ^{13}C NMR (101 MHz, $CDCl_3$) δ = 163.3, 137.2, 137.0, 133.6, 131.5, 130.1, 129.6, 127.80, 127.78, 127.6, 127.4, 127.24, 127.18, 127.0, 126.3, 124.8, 99.5, 93.7, 84.8 (d, *J* = 184 Hz), 78.9, 78.7, 76.9, 74.8, 73.8, 73.0, 72.3, 72.1, 71.7, 66.4 (d, *J* = 5.5 Hz), 8.2, 8.2, -0.0; ^{19}F NMR (376 MHz, $CDCl_3$) δ = -217.5; HRMS (m/z): $[M+Na^+]^+$calcd. for $C_{39}H_{37}FNaO_6^+$, 643.2466; found, 643.2463.

3,4,6-tri-*O*-benzyl-2-fluoro-2-deoxy-*α/β*-D-galactopyranosyl *ortho*-cyclopropylethynylbenzoate (1o)

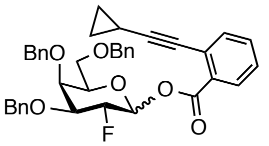

1o, prepared via procedure (C), R_f = 0.2 (Hexanes/EtOAc, 10/1), colorless oil, 613 mg, 99%, α/β = 1/3. α-anomer: ^{1}H NMR (400 MHz, $CDCl_3$) δ 8.07-7.24 (m, 19H, Ar*H*), 6.66 (d, *J* = 3.8 Hz, 1H, H-1), 5.19 (ddd, *J* = 49.6, 9.7, 3.9 Hz, 1H, H-2), 4.96-4.40 (m, 6H, 6 x C*H*Ph), 4.28-4.17 (m, 2H, H-3, H-5), 4.17-4.10 (m, 1H, H-4), 3.81-3.56 (m, 2H, H-6, H-6'), 1.49-1.34 (m, 1H, CH_2C*H*CH_2), 0.86-0.73 (m, 4H, C*H*$_2$CHC*H*$_2$); β-anomer: ^{1}H NMR (400 MHz, $CDCl_3$) δ 8.07-7.24 (m, 19H, Ar*H*), 5.92 (dd, *J* = 7.9, 4.6 Hz, 1H, H-1), 5.07-4.38 (m, 7H, H-2, 6 x C*H*Ph), 4.04 (t, *J* = 3.3 Hz, 1H, H-4), 3.84-3.79 (m, 1H, H-5), 3.75 (ddd, *J* = 12.5, 9.4, 3.1 Hz, 1H, H-3), 3.68-3.54 (m, 2H, H-6, H-6'), 1.49-1.34 (m, 1H, CH_2C*H*CH_2), 0.86-0.73 (m, 4H, C*H*$_2$CHC*H*$_2$); mixture of α and β anomers: ^{13}C NMR (101 MHz, $CDCl_3$) δ = 164.5, 164.4, 138.4, 138.2, 138.0, 137.83, 137.79, 134.8, 134.5, 134.3, 133.0, 132.3, 132.2, 131.7, 131.1, 131.0, 130.8, 130.31, 130.28, 128.60, 128.58, 128.53, 128.44, 128.40, 128.36, 128.3, 128.1, 128.02, 128.00, 127.97, 127.94 127.86, 127.81, 127.7, 127.25, 127.21, 127.0, 125.5, 125.1, 101.2, 100.2, 99.8, 92.7 (d, *J* = 25.4 Hz), 91.7, 90.8 (d, *J* = 23.1 Hz), 89.9, 89.5, 87.6, 80.2, 75.3, 75.2, 75.0, 74.5, 74.32, 74.27, 73.7, 73.0, 72.2, 67.9, 67.7, 9.08, 9.06, 8.98,

0.8, 0.73, 0.71; mixture of α and β anomers: ^{19}F NMR (376 MHz, $CDCl_3$) δ = -206, -207; HRMS (m/z): $[M+Na^+]^+$ calcd. for $C_{39}H_{37}FNaO_6^+$, 643.2466; found, 643.2464.

2,3,4,6-tetra-*O*-benzyl-*α*-D-galactosyl-(1→4)-2,4,6-tri-*O*-benzyl-*α*-D-glucopyranosyl *ortho*-cyclopropylethynylbenzoate (S1)

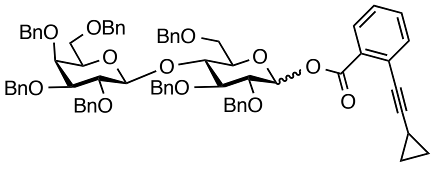

S1, prepared via procedure (C), R_f= 0.2 (Hexanes/EtOAc, 6/1), colorless oil, 540 mg, 92%, α/β = 1/1.2. ^{1}H NMR (400 MHz, $CDCl_3$) δ 7.96-7.10 (m, 39H, Ar*H*), 6.56 (d, *J* = 3.7 Hz, 1H, H-1^A, α-anomer), 5.86 (d, *J* = 7.6 Hz, 1H, H-1^A, β-anomer), 5.11-4.23 (m, 15H, 14 x C*H*Ph, H-1^B), 4.16-3.32 (m, 12H, H-2^A, H-3^A, H-4^A, H-5^A, H-6^A, H-6'^{A}, H-2^B, H-3^B, H-4^B, H-5^B, H-6^B, H-6'^{B}), 1.57-1.45 (m, 1H, CH_2CHCH_2), 0.88-0.81 (m, 4H, CH_2CHCH_2); ^{13}C NMR (101 MHz, $CDCl_3$) δ = 164.6, 164.2, 139.5, 139.2, 139.1, 138.9, 138.8, 138.7, 138.6, 138.34, 138.31, 138.2, 134.6, 134.4, 132.1, 132.0, 131.4, 130.9, 130.72, 130.66, 128.5, 128.43, 128.39, 128.37, 128.29, 128.15, 128.12, 128.08, 128.06, 128.01, 127.92, 127.88, 127.84, 127.82, 127.78, 127.68, 127.65, 127.57, 127.54, 127.46, 127.3, 127.2, 127.1, 127.0, 125.6, 125.2, 83.4, 82.8, 82.6, 80.5, 80.2, 80.1, 80.1, 78.4, 76.3, 76.0, 75.9, 75.6, 75.5, 75.4, 75.2, 74.8, 74.8, 74.6, 73.9, 73.8, 73.5, 73.4, 73.35, 73.30, 73.24, 73.20, 72.8, 72.7, 68.3, 67.8, 67.7, 9.3, 9.2, 9.1; HRMS (m/z): $[M+H^+]^+$ calcd. for $C_{73}H_{73}O_{12}^+$, 1141.5097; found, 1141.5099.

***p*-methoxyphenyl 2,3,4,6-tetra-*O*-benzoyl-*α*-D-mannosyl-(1→4)-(2,3,4,6-tetra-*O*-benzoyl-*α*-D-mannosyl-(1→6))-2,3-di-*O*-benzyl-*α*-D-glucopyranoside (S3)**

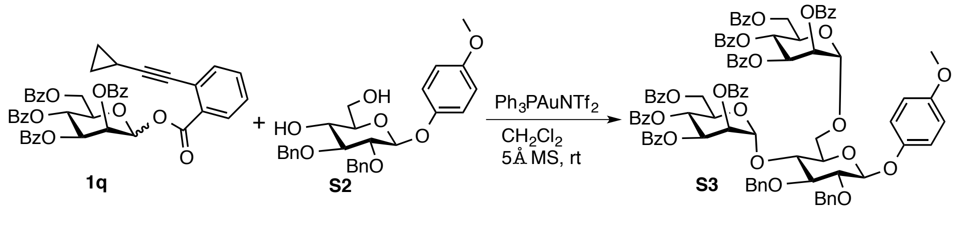

To a solution of donor **1q** (261 mg, 0.34 mmol) and acceptor **S2** (72.4 mg, 0.155 mmol) in CH_2Cl_2 (3.0 mL) was added $Ph_3PAuNTf_2$ (23 mg, 0.031 mmol) at 0 °C. After stirring for 0.5 h, the reaction was stirred at room temperature for 2 h. Then, the reaction was quenched with Et_3N (1.0 mL). After filtration, the filtrate was concentrated to give a residue, which was loaded to a silica gel column, and purified (eluting solvent:

Hexanes/EtOAc, 4/1) to give desired product **S3** (239 mg, 95% yield). R_f = 0.5 (Hexanes/EtOAc, 2/1). $[\alpha]_D^{25}$ = -31 (*c* 3.3 $CHCl_3$); ^{1}H NMR (400 MHz, $CDCl_3$) δ 8.12-6.97 (m, 54H), 6.11 (t, *J* = 10.1 Hz, 1H), 6.05-5.98 (m, 2H), 5.95-5.80 (m, 2H), 5.81-5.80 (m, 1H), 5.65 (d, *J* = 1.9 Hz, 1H), 5.25 (d, *J* = 1.6 Hz, 1H), 5.11-5.04 (m, 3H), 4.85 (d, *J* = 11.2 Hz, 1H), 4.75 (d, *J* = 11.0 Hz, 1H), 4.66-4.48 (m, 5H), 4.35 (dd, *J* = 12.2, 4.2 Hz, 1H), 4.14-4.02 (m, 3H), 3.92 (t, *J* = 8.8 Hz, 1H), 3.78-3.69 (m, 2H), 3.43 (s, 3H); ^{13}C NMR (101 MHz, $CDCl_3$) δ 166.2, 166.1, 165.64, 165.61, 165.56, 165.4, 165.0, 155.5, 152.0, 138.11, 138.06, 133.6, 133.5, 133.34, 133.25, 133.13, 133.10, 130.2, 130.04, 129.99, 129.92, 129.89, 129.82, 129.78, 129.67, 129.5, 129.4, 129.2, 129.0, 128.8, 128.7, 128.63, 128.59, 128.57, 128.50, 128.47, 128.43, 128.39, 128.2, 127.9, 127.4, 117.6, 115.1, 102.8, 99.6, 97.5, 84.2, 82.1, 77.9, 75.2, 74.8, 73.2, 70.5, 70.4, 70.0, 69.9, 69.1, 67.3, 66.5, 63.5, 62.7, 55.3; HRMS (m/z): $[M+Na^+]^+$ calcd. for $C_{95}H_{82}O_{25}Na^+$, 1645.5037; found, 1645.5034.

2,3,4,6-tetra-*O*-benzoyl-*α*-D-mannosyl-(1→4)-(2,3,4,6-tetra-*O*-benzoyl-*α*-D-mannosyl-(1→6))-2,3-di-*O*-benzyl-*α*-D-glucopyranosyl *ortho*-cyclopropylethynylbenzoate (S4)

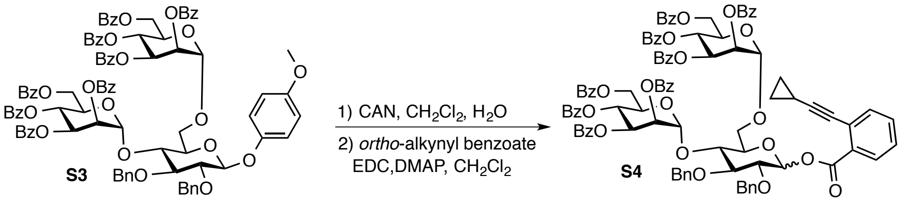

To a solution of donor **S3** (230 mg, 0.142 mmol) in CH_2Cl_2 (8.0 mL) and H_2O (2 mL) was added Ammonium Cerium (IV) Nitrate (CAN) (233 mg, 0.426 mmol) at 0 °C. After stirring at 0 °C for 1 h, the reaction was quenched with aqueous $Na_2S_2O_3$. The mixture was diluted with EtOAc, wash with saturated $NaHCO_3$, H_2O, brine, and dried over Na_2SO_4. After filtration, the filtrate was concentrated to give a residue, which was loaded to a silica gel column, and purified (eluting solvent: Hexanes/EtOAc, 4/1) to give 1-OH product (151 mg, 70% yield), R_f= 0.4 (Hexanes/EtOAc, 2/1). Then, the 1-OH compound was used to prepared the glycosyl donor **S4** via the procedure (C) (143 mg, 85% yield). ^{1}H NMR (400 MHz, $CDCl_3$) δ 8.12-6.97 (m, 54H), 6.17-5.77 (m, 7H), 5.26 (s, 1H), 5.20 (s, 1H), 4.94-3.87 (m, 12H); ^{13}C NMR (101 MHz, $CDCl_3$) δ 166.2,

166.14, 166.09, 165.65, 165.59, 165.4, 165.3, 165.2, 165.0, 164.9, 164.1, 138.2, 138.0, 137.7, 137.6, 135.0, 134.3, 133.4, 133.35, 133.30, 133.24, 133.20, 133.0, 132.2, 132.1, 131.2, 130.9, 130.6, 130.4, 130.2, 130.03, 129.97, 129.91, 129.88, 129.82, 129.78, 129.5, 129.4, 129.38, 129.35, 129.26, 129.22, 129.14, 129.07, 129.04, 128.8, 128.73, 128.66, 128.60, 128.54, 128.50, 128.44, 128.36, 128.29, 128.25, 128.19, 128.14, 127.9, 127.8, 127.7, 127.5, 127.44, 127.39, 127.33, 127.0, 125.3, 125.1, 100.2, 99.7, 99.6, 98.6, 98.2, 94.4, 90.4, 84.1, 81.2, 80.9, 79.7, 76.0, 75.3, 75.0, 74.9, 74.5, 73.2, 72.9, 70.5, 70.4, 70.3, 70.2, 70.1, 70.0, 69.4, 69.2, 67.6, 67.2, 66.6, 66.5, 63.4, 63.3, 62.8, 62.6, 9.4, 9.4, 9.1, 9.0; HRMS (m/z): $[M+H^+]^+$ calcd. for $C_{100}H_{85}O_{25}^+$, 1685.5374; found, 1685.5375.

1.3.4 Procedure (D) (E) for acceptor synthesis and characterization

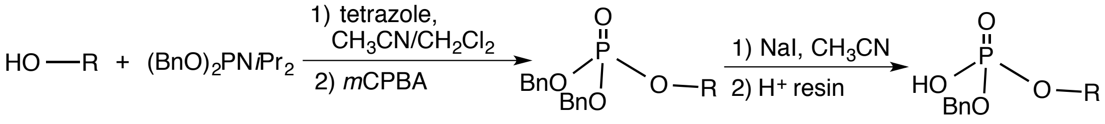

S5-S10 **2b-2g**

General procedure (D) for the synthesis of phosphoric acid acceptor. To a solution of alcohol (1.0 equiv.) in CH_3CN and CH_2Cl_2 (1/4, 0.2 M) was added $(BnO)_2PN^iPr_2$ (1.5 equiv.) and tetrazole (1.8 equiv.), and the mixture was stirred for 2 h at room temperature, after which the alcohol disappeared on TLC plate. Then, *m*CPBA (4.0 equiv.) was added into the mixture, and the reaction was stirred for 2 h, after which the reaction was quenched with $Na_2S_2O_3$ solution. After removing the CH_3CN by evaporation, the mixture was diluted with EtOAc, washed with H_2O, saturated $NaHCO_3$, brine and dried over Na_2SO_4. After filtration, the filtrate was concentrated to give a residue, which was purified via flash silica gel chromatography to give the desired product phosphotriester (**S5-S10**).

General procedure (E): To a solution of phosphotriester in CH_3CN (0.2 M) was added NaI (4.0 equiv.), then the mixture was stirred at 60 °C for 3 h. After cooling down, the reaction was quenched with Et_3N (20 equiv.). Then, the solvent was removed and the mixture was acidified with aqueous HCl (1.0 M, 3.0 mL), extracted with EtOAc (4 x 20 mL), dried over Na_2SO_4. To neutralized the strong acid of HI, Et_3N (20 equiv.) was added. After filtration, the filtrate was concentrated to give a residue, which was loaded to silica gel column which was neutralized by using Hexanes/Et_3N (100/1) and purified by eluting with solvents (Hexanes/EtOAc, 1/1→CH_2Cl_2/MeOH, 50/1→10/1) to give

the product in the form of phosphate salt. The yellow substance should be separated from the product by using the silica gel column chromatography. Then, a solution of the salt in mixed solvent of CH_2Cl_2/MeOH (3 mL/3 mL) was added H^+ resin (Dowex 50WX8, 400 mg) to acidify, and after filtration through cotton, phosphoric acid glycosyl benzyl ester (**2b-2g**) was obtained.

Methyl 6-*O*-dibenzyloxyphosphoryl-2,3,4-tri-*O*-benzoyl-*α*-D-glucopyranoside (S5)

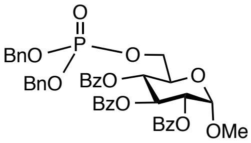

S5, prepared via procedure (D), purified via flash silica gel chromatography (Hexanes/EtOAc, 3/1 to 2/1), R_f= 0.2 (Hexanes/EtOAc, 3/2), white foam, 400 mg, 90% yield. $[\alpha]_D^{25}$ = 45.6 (*c* 1.6, $CHCl_3$); ^{1}H NMR (400 MHz, $CDCl_3$) δ 7.98-7.27 (m, 25H, Ar*H*), 6.12 (t, *J* = 9.4 Hz, 1H, H-3), 5.52 (t, *J* = 9.7 Hz, 1H, H-4), 5.22-5.18 (m, 2H, H-1, H-2), 5.09-5.01 (m, 4H, CH_2Ph x 2), 4.23-4.16 (m, 3H, H-5, H-6, H-6'), 3.41 (s, 3H, CH_3); ^{13}C NMR (101 MHz, $CDCl_3$) δ = 165.91, 165.89, 165.3, 136.0, 135.96, 135.92, 135.89, 135.86, 133.6, 133.5, 133.2, 130.1, 130.0, 129.8, 129.3, 129.2, 129.0, 128.8, 128.69, 128.65, 128.59, 128.55, 128.4, 128.2, 128.1, 128.0, 97.1, 72.1, 70.5, 69.55 (d, J = 5.7 Hz), 69.53 (d, J = 5.7 Hz), 69.1, 68.4 (d, *J* = 8.0 Hz), 66.0 (d, *J* = 5.3 Hz), 55.8; ^{31}P NMR (162 MHz, $CDCl_3$) δ = -1.2; HRMS (m/z): $[M+H^+]^+$ calcd. for $C_{42}H_{40}O_{12}P^+$, 767.2252; found, 767.2250.

methyl 2,3,4-tri-*O*-benzoyl-6-*O*-(benzyloxy-hydroxy-phosphoryl)-*α*-D-glucopyranoside (2b)

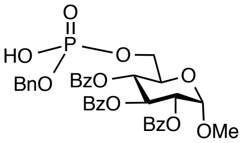

2b, prepared via procedure (E), R_f= 0.2 (CH_2Cl_2/MeOH, 10/1), white foam, 300 mg, 84% yield. $[\alpha]_D^{25}$ = 65.7 (*c* 1.7, $CHCl_3$); ^{1}H NMR (400 MHz, $CDCl_3$) δ 8.33 (bs, 1H, O*H*), 7.98-7.28 (m, 20H, Ar*H*), 6.13 (t, *J* = 9.6 Hz, 1H, H-3), 5.50 (t, *J* = 9.7 Hz, 1H, H-4), 5.24-5.20 (m, 2H, H-1, H-2), 5.08-4.99 (m, 2H, CH_2Ph), 4.33-4.05 (m, 3H, H-5, H-6, H-6'), 3.41 (s, 3H, CH_3); ^{13}C NMR (101 MHz, $CDCl_3$) δ = 165.9, 165.8, 165.5, 133.52, 133.45, 133.2, 130.1, 130.0, 129.8, 129.4, 129.2, 129.0, 128.6, 128.5, 128.44, 128.39, 127.9, 97.0, 72.1, 70.6, 69.3, 69.2, 68.5 (d, *J* = 7.9 Hz), 66.0, 55.8; ^{31}P NMR (162 MHz, $CDCl_3$) δ = 0.7; HRMS (m/z): $[M+H^+]^+$ calcd. for $C_{35}H_{34}O_{12}P^+$, 677.1782; found, 677.1781.

6-*O*-dibenzyloxyphosphoryl-1,2:3,4-di-*O*-isopropylidene-*α*-D-galactopyranose (S6)

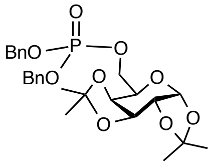

S6, prepared via procedure (D), purified by silica gel chromatography (Hexanes/EtOAc, 2/1 to 3/2), R_f= 0.2 (Hexanes/EtOAc, 3/2), white foam, 570 mg, 95% yield. $[\alpha]_D^{25}$ = -37.5 (*c* 3.3, $CHCl_3$); ^{1}H NMR (400 MHz, $CDCl_3$) δ 7.36-7.32 (m, 10H, Ar*H*), 5.53 (d, *J* = 4.9 Hz, 1H, H-1), 5.15 – 5.02 (m, 4H, C*H*2Ph x 2), 4.59 (dd, *J* = 7.9, 2.5 Hz, 1H, H-3), 4.32 (dd, *J* = 5.0, 2.5 Hz, 1H, H-2), 4.21-4.12 (m, 3H, H-4, H-6, H-6'), 4.12-4.04 (m, 1H, H-5), 1.49 (s, 3H, C*H*3), 1.41 (s, 3H, C*H*3), 1.31 (s, 3H, C*H*3), 1.30 (s, 3H, C*H*3); ^{13}C NMR (101 MHz, $CDCl_3$) δ = 136.04, 135.96, 128.8, 128.6, 128.5, 128.14, 128.07, 128.0, 109.7, 108.9, 96.4, 70.74, 70.68, 70.56, 69.4 (d, *J* =5.7 Hz, 2C), 67.4, 67.38, 66.9 (d, *J* = 7.1 Hz), 66.4 (d, *J* = 5.6 Hz), 26.1, 26.0, 25.1, 24.5; ^{31}P NMR (162 MHz, $CDCl_3$) δ -1.08; HRMS (m/z): $[M+H^+]^+$ calcd. for $C_{26}H_{34}O_9P^+$, 521.1935; found, 521.1933.

6-*O*-(benzyloxy-hydroxy-phosphoryl)-1,2:3,4-di-*O*-isopropylidene-*α*-D-galactopyranose (2c)

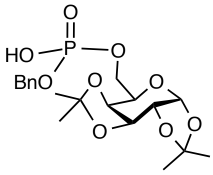

2c, prepared via procedure (E), R_f= 0.2 (CH_2Cl_2/MeOH, 9/1), white solid, 230 mg, 72% yield; $[\alpha]_D^{25}$ = -33.3 (*c* 0.8, $CHCl_3$); ^{1}H NMR (400 MHz, $CDCl_3$) δ 7.36-7.27 (m, 5H, Ar*H*), 5.50 (d, *J* = 5.0 Hz, 1H, H-1), 5.05 (d, *J* = 6.7 Hz, 2H, C*H*2Ph), 4.54 (dd, *J* = 7.8, 2.4 Hz, 1H, H-3), 4.28 (dd, *J* = 5.0, 2.4 Hz, 1H, H-2), 4.20-4.07 (m, 3H, H-4, H-6, H-6'), 4.01 (t, *J* = 6.3 Hz, 1H, H-5), 1.48 (s, 3H, C*H*3), 1.38 (s, 3H, C*H*3), 1.29 (s, 3H, C*H*3), 1.27 (s, 3H, C*H*3); ^{13}C NMR (101 MHz, $CDCl_3$) δ = 136.5, 136.4, 128.6, 128.2, 128.0, 109.7, 109.0, 96.3, 70.7, 70.6, 70.6, 69.0 (d, *J* = 4.5 Hz), 67.0 (d, *J* = 7.8 Hz), 66.0 (bs), 26.1, 26.1, 25.0, 24.5; ^{31}P NMR (162 MHz, $CDCl_3$) δ = -1.0; HRMS (m/z): $[M+H^+]^+$ calcd. for $C_{19}H_{28}O_9P^+$, 431.1465; found, 431.1464.

methyl 2,3,6-tri-*O*-benzyl-4-*O*-dibenzyloxyphosphoryl-*α*-D-glucopyranoside (S7)

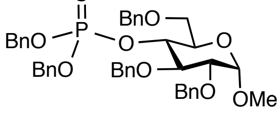

S7, prepared via procedure (D), purified by silica gel chromatography (Hexanes/EtOAc, 3/1 to 2/1), R_f = 0.2 (Hexanes/EtOAc, 2/1), colorless oil, 298 mg, 96% yield. $[\alpha]_D^{25}$ = 10.5 (*c* 1.7, $CHCl_3$); ^{1}H NMR (400 MHz, $CDCl_3$) δ 7.39-7.10 (m, 25H, Ar*H*), 4.97-4.73 (m, 7H, 7 x C*H*Ph), 4.61-4.58 (m, 2H, C*H*Ph, H-1), 4.53-4.46 (m, 2H, C*H*Ph, H-4), 4.41 (d, *J* = 11.9 Hz, 1H, C*H*Ph), 3.96 (t, *J* = 9.3 Hz, 1H, H-3), 3.83 (ddd, *J* = 10.1, 4.7, 2.0 Hz, 1H, H-5), 3.75-3.66 (m, 2H, H-6, H-6'), 3.56 (dd, *J* = 9.6, 3.6 Hz, 1H, H-2), 3.38 (s, 3H, OMe); ^{13}C NMR (101 MHz, $CDCl_3$) δ = 138.8, 138.3, 138.0, 136.04, 136.96, 128.6, 128.53, 128.48, 128.40, 128.36, 128.3, 128.1, 127.94, 127.91, 127.81, 127.77, 127.6, 127.4, 98.0, 79.9 (d, *J* = 2.9 Hz), 79.7, 75.9 (d, *J* = 7.1 Hz), 75.4, 73.6, 73.5, 69.5, 69.4 (d, 2^{13}C, *J* = 5.5 Hz), 68.6, 55.5; ^{31}P NMR (162 MHz, $CDCl_3$) δ = -0.6; HRMS (m/z): $[M+H^+]^+$ calcd. for $C_{42}H_{46}O_9P^+$, 725.2874; found, 725.2873.

methyl 2,3,6-tri-*O*-benzyl-4-*O*-(benzyloxy-hydroxy-phosphoryl)-*α*-D-glucopyranoside (2d)

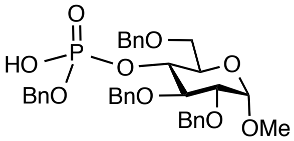

2d, prepared via procedure (E), R_f = 0.2 (CH_2Cl_2/MeOH, 10/1), white foam, 200 mg, 88% yield. $[\alpha]_D^{25}$ = 3.1 (*c* 3.9, $CHCl_3$); ^{1}H NMR (400 MHz, $CDCl_3$) δ 7.38-7.14 (m, 20H, Ar*H*), 4.92-4.81 (m, 4H, C*H*$_2$Ph x 2), 4.70 (d, *J* = 12.1 Hz, 1H, C*H*Ph), 4.55-4.50 (m, 3H, C*H*$_2$Ph, H-1), 4.44-4.37 (m, 2H, C*H*Ph, H-4), 3.92 (t, *J* = 9.3 Hz, 1H, H-3), 3.77-3.65 (m, 3H, H-5, H-6, H-6'), 3.44 (dd, *J* = 9.6, 3.6 Hz, 1H, H-2), 3.34 (s, 3H, C*H*$_3$); ^{13}C NMR (101 MHz, $CDCl_3$) δ = 138.5, 138.2, 138.1, 128.5, 128.5, 128.5, 128.3, 128.2, 128.0, 127.9, 127.7, 127.6, 127.5, 98.0, 80.1, 79.5, 76.0 (d, *J* = 7.6 Hz), 75.9, 69.3 (bs), 68.3, 55.4; ^{31}P NMR (162 MHz, $CDCl_3$) δ = -0.5; HRMS (m/z): $[M+H^+]^+$ calcd. for $C_{35}H_{40}O_9P^+$, 635.2404; found, 635.2405.

methyl 2,3,6-tri-*O*-benzoyl-4-*O*-dibenzyloxyphosphoryl-*α*-D-galactopyranoside (S8)

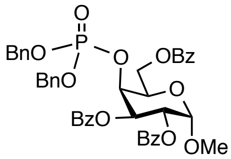

S8, prepared via procedure (D), purified by silica gel chromatography (Hexanes/EtOAc, 3/1 to 2/1), R_f = 0.2 (Hexanes/EtOAc, 3/1), colorless oil, 567 mg, 94% yield. $[\alpha]_D^{25}$ = 81.9 (*c* 4.5, $CHCl_3$); ^{1}H NMR (400 MHz, $CDCl_3$) δ 8.07-7.12 (m, 25H, Ar*H*), 5.78 (ddd, *J* = 10.8, 3.0, 1.4 Hz, 1H, H-3), 5.68 (dd, *J* = 10.8, 3.6 Hz, 1H, H-2), 5.31 (dd, *J* = 9.4,

3.0 Hz, 1H, H-4), 5.21 (d, *J* = 3.6 Hz, 1H, H-1), 4.99-4.85 (m, 4H, C*H*$_2$Ph x 2), 4.68-4.62 (m, 1H, H-6), 4.50-4.43 (m, 2H, H-5, H-6'), 3.44 (s, 3H, C*H*$_3$); ^{13}C NMR (101 MHz, CDCl$_3$) δ = 166.1, 166.08, 166.0, 135.7, 135.6, 133.5, 133.31, 133.25, 130.0, 129.9, 129.8, 129.5, 129.4, 128.63, 128.59, 128.55, 128.52, 128.4, 128.0, 127.97, 97.7, 74.5 (d, *J* = 5.4 Hz), 69.74 (d, *J* = 5.9 Hz), 69.70 (d, *J* = 5.4 Hz), 69.1 (d, *J* = 2.2 Hz), 68.6, 67.2 (d, *J* = 4.2 Hz), 62.8, 55.8; ^{31}P NMR (162 MHz, CDCl$_3$) δ = -0.9; HRMS (m/z): [M+H$^+$]$^+$ calcd. for $C_{42}H_{40}O_{12}P^+$, 767.2252; found, 767.2250.

methyl 2,3,6-tri-*O*-benzoyl-4-*O*-(benzyloxy-hydroxy-phosphoryl)-*α*-D-galactopyranoside (2e)

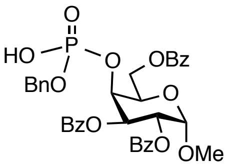

2e, prepared via procedure (E), R$_f$ = 0.35 (CH$_2$Cl$_2$/MeOH, 10/1), white foam, 440 mg, 88% yield. [α]$_D$^{25} = 114 (*c* 1.2, CHCl$_3$); ^{1}H NMR (400 MHz, CDCl$_3$) δ 7.98-7,13 (m, 20H, Ar*H*), 6.64 (br, 1H, O*H*), 5.69 (d, *J* = 10.7 Hz, 1H, H-3), 5.55 (dd, *J* = 10.7, 3.5 Hz, 1H, H-2), 5.10 (s, 1H, H-1), 5.00 (d, *J* = 9.3 Hz, 1H, H-4), 4.93-4.81 (m, 2H, C*H*$_2$Ph), 4.60 (dd, *J* = 11.7, 8.3 Hz, 1H, H-6), 4.47 (d, *J* = 11.3 Hz, 1H, H-6'), 4.23 (s, 1H, H-5), 3.36 (s, 3H, C*H*$_3$); ^{13}C NMR (101 MHz, CDCl$_3$) δ = 166.4, 166.1, 166.0, 133.3, 133.1, 133.0, 130.03, 130.01, 129.9, 129.63, 129.59, 128.5, 128.4, 128.3, 127.8, 97.3, 74.1, 69.2, 69.0, 67.5, 55.4; ^{31}P NMR (162 MHz, CDCl$_3$) δ = 0.2; HRMS (m/z): [M-H$^+$]$^-$ calcd. for $C_{35}H_{32}O_{12}P^-$, 675.1637; found, 675.1641.

***tert*-butyldimethylsilyl 2-azido-4,6-*O*-benzylidene-3-*O*-dibenzyloxyphosphoryl-2-deoxy-*β*-D-glucopyranoside (S9)**

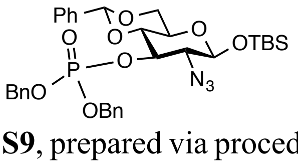

S9, prepared via procedure (D), purified by silica gel chromatography (Hexanes/EtOAc, 6/1→4/1), R$_f$ = 0.2 (Hexanes/EtOAc, 4/1), colorless oil, 340 mg, 85% yield. [α]$_D$^{25} = -51.4 (*c* 1.2, CHCl$_3$); ^{1}H NMR (400 MHz, CDCl$_3$) δ 7.44-7.01 (m, 15H, Ar*H*), 5.47 (s, 1H, PhC*H*), 5.13-5.04 (m, 2H, C*H*$_2$Ph), 4.94 (dd, *J* = 12.1, 6.6 Hz, 1H, C*H*HPh), 4.87 (dd, *J* = 12.1, 7.5 Hz, 1H, C*H*HPh), 4.69 (d, *J* = 7.6 Hz, 1H, H-1), 4.49 (dd, *J* = 9.6 Hz, *J* = 9.6 Hz, 1H, H-3), 4.30 (dd, *J* = 10.6, 5.0 Hz, 1H, H-6), 3.78 (t, *J* = 10.3 Hz, 1H, H-6'), 3.71 (t, *J* = 9.4 Hz, 1H, H-4), 3.48-3.42 (m, 2H, H-2, H-5), 0.94 (s, 9H, C(CH$_3$)$_3$), 0.18 (s, 3H, CH$_3$), 0.16 (s, 3H, CH$_3$); ^{13}C NMR (101 MHz, CDCl$_3$) δ = 136.8, 136.1,

135.97, 135.9, 129.4, 128.6, 128.5, 128.4, 128.2, 127.9, 127.4, 126.5, 102.1, 97.8, 79.4 (d, *J* = 2.9 Hz), 77.20 (d, *J* = 32 Hz), 69.6 (d, *J* = 5.6 Hz), 69.3 (d, *J* = 5.2 Hz), 68.6 (d, *J* = 3.0 Hz), 68.3, 66.3, 25.7, -4.3, -5.1; ^{31}P NMR (162 MHz, $CDCl_3$) δ = -1.9; HRMS (m/z): $[M+H^+]^+$ calcd. for $C_{33}H_{43}N_3O_8PSi^+$, 668.2552; found, 668.2547.

***tert*-butyldimethylsilyl 2-azido-4,6-*O*-benzylidene-3-*O*-(benzyloxy-hydroxy-phosphoryl)-2-deoxy-*β*-D-glucopyranoside (2f)**

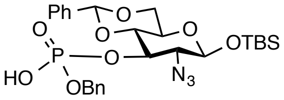

2f, prepared via procedure (E), R_f = 0.45 (CH_2Cl_2/MeOH, 10/1), colorless oil, 118 mg, 75% yield. $[\alpha]_D^{25}$ = -40.9 (*c* 1.8, $CHCl_3$); ^{1}H NMR (400 MHz, $CDCl_3$) δ 9.83 (bs, 1H, OH), 7.31-6.98 (m, 10H, Ar*H*), 5.33 (s, 1H, C*H*Ph), 4.85-4.76 (m, C*H*$_2$Ph), 4.43 (d, *J* = 7.6 Hz, 1H, H-1), 4.22-4.11 (m, 2H, H-4, H-6), 3.61 (t, *J* = 10.3 Hz, 1H, H-6'), 3.52 (t, *J* = 9.4 Hz, 1H, H-4), 3.27 (dd, *J* = 9.7, 7.6 Hz, 1H, H-2), 3.20 (td, *J* = 9.7, 5.0 Hz, 1H, H-5), 0.79 (s, 9H, ^{t}Bu), 0.01 (s, 3H, C*H*$_3$), 0.00 (s, 3H, C*H*$_3$); ^{13}C NMR (101 MHz, $CDCl_3$) δ = 136.8, 136.1, 136.0, 129.2, 128.43, 128.39, 128.1, 127.4, 126.3, 101.6, 97.6, 79.3 (d, *J* = 3.3 Hz), 69.1 (d, *J* = 5.3 Hz), 68.5 (d, *J* = 2.7 Hz), 68.4, 66.1, 25.6, 18.0, -4.2, -5.1; ^{31}P NMR (162 MHz, $CDCl_3$) δ = -0.4; HRMS (m/z): $[M+H^+]^+$ calcd. for $C_{26}H_{37}N_3O_8PSi^+$, 578.2082; found, 578.2087.

3-*O*-dibenzyloxyphosphoryl-*N*-(9-fluorenylmethoxycarbonyl)-L-serine allyl ester (S10)

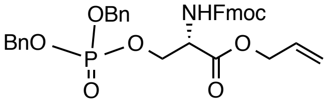

S10, prepared via procedure (D), purified by silica gel chromatography (Hexanes/EtOAc, 2/1 to 3/2), R_f = 0.2 (Hexanes/EtOAc, 2/1), colorless oil, 183 mg, 99% yield. $[\alpha]_D^{25}$ = 7.0 (*c* 1.4 $CHCl_3$); ^{1}H NMR (400 MHz, $CDCl_3$) δ 7.77-7.28 (m, 18H, Ar*H*), 5.91-5.79 (m, 2H, NH, C*H*=CH$_2$), 5.35-5.26 (m, 1H, CH*H*=CH), 5.22 (dd, *J* = 10.4, 1.4 Hz, 1H, C*H*H=CH), 5.07-4.91 (m, 4H, 2 x C*H*$_2$Ph), 4.64-4.55 (m, 3H, C*H*NH, C*H*$_2$CH=CH$_2$), 4.46-4.40 (m, 2H, CH$_2$OP), 4.33-4.19 (m, 3H, CH(Fmoc), CH$_2$(Fmoc)); ^{13}C NMR (101 MHz, $CDCl_3$) δ = 168.7, 156.0, 143.9, 143.8, 141.4, 135.64, 135.57, 131.4, 128.9, 128.8, 128.1, 127.9, 127.3, 125.31, 125.25, 120.1, 119.2, 69.9 (d, *J* = 5.7 Hz), 69.8 (d, *J* = 5.7 Hz), 67.5, 67.4 (d, *J* = 5.7 Hz), 66.7, 54.6 (d, *J* = 7.3 Hz), 47.2; ^{31}P NMR (162 MHz, $CDCl_3$) δ = -0.9; HRMS (m/z): $[M+H^+]^+$ calcd. for $C_{35}H_{35}NO_8P^+$, 628.2095; found, 628.2099.

3-*O*-(benzyloxy-hydroxy-phosphoryl)-*N*-(9-fluorenylmethoxycarbonyl)-L-serine allyl ester (2g)

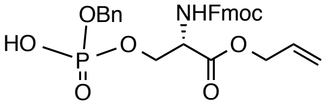

2g, prepared via procedure (E), R_f = 0.2 (CH_2Cl_2/MeOH, 10/1), white solid, 130 mg, 76% yield. $[\alpha]_D^{25}$ = 14 (*c* 0.3 $CHCl_3$); ^{1}H NMR (400 MHz, $CDCl_3$) δ 7.75-7.28 (m, 13H, Ar*H*), 5.91-5.81 (m, C*H*=CH_2), 5.30 (dd, *J* = 17.1, 1.7 Hz, 1H, CH=C*H*H), 5.24-5.17 (m, 1H, CH=CH*H*), 5.02 (d, *J* = 7.9 Hz, 2H, CH_2Ph), 4.69-4.56 (m, 3H, C*H*NH, CH_2CH=CH_2), 4.47-4.18 (m, 6H, CH(Fmoc), CH_2(Fmoc), CH_2OP, OH); ^{13}C NMR (101 MHz, $CDCl_3$) δ = 168.8, 156.1, 143.9, 141.4, 131.4, 128.81, 128.77, 128.0, 127.9, 127.3, 125.4, 120.1, 119.3, 69.7 (bs), 67.6, 67.4 (bs), 66.8, 54.6 (d, *J* = 7.1 Hz), 47.2; ^{31}P NMR (162 MHz, $CDCl_3$) δ = 0.5; HRMS (m/z): $[M+H^+]^+$ calcd. for $C_{28}H_{29}NO_8P^+$, 538.1625; found, 538.1624.

1.3.5 Global deprotection

General procedure (F): General procedure B was used to removal benzyl group of benzyl phosphate. To a solution of the residue prepared via procedure (B) in H_2O/MeOH (1.0 mL/2.0 mL) was added $HCOONH_4$ (10 equiv.), HCOOH (20 equiv.) and Pd/C (50 mg). Then, the mixture was stirred under an atmosphere of H_2 at room temperature for 12 h. After filtration through syringe filter, the filtrate was concentrated to give a residue. The desired product was obtained by purifying via P2 polyacrylamide gel column (45x0.7 cm, eluent: 10 mM aqueous NH_4HCO_3).

α-D-glucopyranosyl-1-phosphate (5)

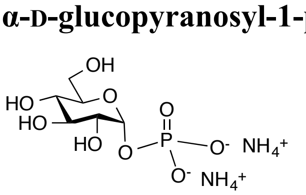

12.2 mg, 95% yield, $[\alpha]_D^{25}$ = 114 (*c* 1.0 $CHCl_3$); ^{1}H NMR (400 MHz, D_2O) δ 5.30 (dd, *J* = 7.4, 3.5 Hz, 1H, H-1), 3.84-3.68 (m, 2H, H-5, H-6), 3.65-3.54 (m, 2H, H-3, H-6’), 3.34 (ddd, *J* = 9.8, 3.5, 1.8 Hz, 1H, H-2), 3.24 (t, *J* = 9.6 Hz, 1H, H-4); ^{13}C NMR (101 MHz, D_2O) δ 93.58 (d, *J* = 5.5 Hz), 73.1, 72.16 (d, *J* = 6.8 Hz), 72.0, 69.8, 60.7; ^{31}P NMR (162 MHz, D_2O) δ = 2.2; HRMS (m/z): $[M-2NH_4^++H^+]^-$ calcd. for $C_6H_{12}O_9P^-$, 259.0224; found, 259.0230.

α-D-galactopyranosyl-1-phosphate (6)

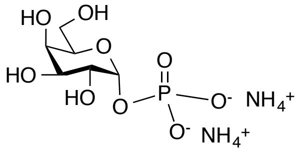

12.2 mg, 92% yield, $[\alpha]_D^{25}$ = 81 (*c* 1.1 $CHCl_3$); ^{1}H NMR (400 MHz, D_2O) δ 5.38 (dd, *J* = 7.4, 3.6 Hz, 1H, H-1), 4.11-3.98 (m, 1H, H-5), 3.88 (dd, *J* = 3.4, 1.1 Hz, 1H, H-4), 3.79 (dd, *J* = 10.3, 3.3 Hz, 1H, H-3), 3.69-3.49 (m, 3H, H-2, H-6, H-6'); ^{13}C NMR (101 MHz, D_2O) δ = 94.07 (d, *J* = 5.5 Hz), 71.2, 69.5, 69.4, 68.89 (d, *J* = 6.8 Hz), 61.3; ^{31}P NMR (162 MHz, D_2O) δ = 1.6; HRMS (m/z): $[M-2NH_4^++H^+]^-$ calcd. for $C_6H_{12}O_9P^-$, 259.0224; found, 259.0228.

methyl *α*-D-glucopyranosyl-phosphoryl-(→4)-*α*-D-glucopyranoside triammonium salt (7)

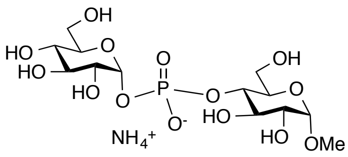

8.3 mg, 90% yield, $[\alpha]_D^{25}$ = 166 (*c* 0.5 $CHCl_3$); ^{1}H NMR (400 MHz, D_2O) δ 5.49 (dd, *J* = 6.6, 3.5 Hz, 1H, H-1^B), 4.72 (d, *J* = 3.7 Hz, 1H, H-1^A), 3.87-3.61 (m, 9H, H-2^A, H-3^A, H-5^A, H-6^A, H-6'^{A}, H-2^B, H-3^B, H-4^B, H-5^B, H-6^B, H-6'^{B}), 3.52-3.45 (m, 2H, H-2^A, H-2^B), 3.38 (t, *J* = 9.6 Hz, 1H, H-4^B), 3.32 (s, 3H); ^{13}C NMR (101 MHz, D_2O) δ = 99.1, 95.8 (d, *J* = 6.7 Hz), 73.9 (d, *J* = 6.4 Hz), 72.8 (d, *J* = 3.8 Hz), 72.3, 71.6 (d, *J* = 8.7 Hz), 71.1, 70.7 (d, *J* = 4.7 Hz), 69.2, 60.4 (d, *J* = 10.4 Hz), 55.1; ^{31}P NMR (162 MHz, D_2O) δ = -1.8; HRMS (m/z): $[M-NH_4^+]^-$ calcd. for $C_{13}H_{24}O_{14}P^-$, 435.0909; found, 435.0907.

methyl *α*-D-glucopyranosyl-phosphoryl-(→4)-*α*-D-galactopyranoside triammonium salt (8)

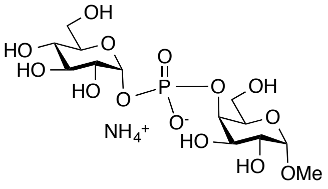

General procedure B was used to remove benzyl group of benzyl phosphate. Then, **t**o a solution of the residue (prepared via procedure B) in H_2O/MeOH (1.0 mL/2.0 mL)

was added $HCOONH_4$ (10 equiv.), HCOOH (20 equiv.) and Pd/C (50 mg). Then, the mixture was stirred under an atmosphere of H_2 at room temperature for 12 h. After filtration through syringe filter, the filtrate was concentrated to give a residue which was used for the next step without purification. The resulting residue was dissolved in MeOH/NH_2NH_2•H_2O (1.0 mL/0.4 mL), and the resulting mixture was stirred for 12 h at room temperature, after which the solvent was removed via evaporation to provide the crude product. Afterwards, the product was purified via P2 polyacrylamide gel column (45x0.7 cm, eluent: 10 mM aqueous NH_4HCO_3) to give the desired product **8** (7.8 mg, 89%). $[\alpha]_D^{25}$ = 176 (*c* 0.5 $CHCl_3$); ^{1}H NMR (400 MHz, D_2O) δ 5.57-5.39 (m, 1H, H-1^B), 4.78 (d, *J* = 2.0 Hz, 1H, H-1^A), 4.50 (d, *J* = 9.6 Hz, 1H, H-4^A), 3.91 (t, *J* = 6.5 Hz, 1H, H-5^A), 3.81-3.62 (m, 8H, H-2^A, H-3^A, H-6^A, H-6'^{A}, H-3^B, H-5^B, H-6^B, H-6'^{B}), 3.53-3.47 (m, 1H, H-2^B), 3.39 (t, *J* = 9.7 Hz, 1H, H-4^B), 3.35 (s, 3H, OMe); ^{13}C NMR (101 MHz, D_2O) δ = 99.4, 95.54 (d, *J* = 6.4 Hz), 75.18 (d, *J* = 6.3 Hz), 72.77 (d, *J* = 9.7 Hz), 71.35 (d, *J* = 8.1 Hz), 70.40, 69.1, 69.06 (d, *J* = 2.1 Hz), 68.2, 60.9, 60.3, 55.1; ^{31}P NMR (162 MHz, D_2O) δ = -1.3; HRMS (m/z): $[M-NH_4^+]^-$ calcd. for $C_{13}H_{24}O_{14}P^-$, 435.0909; found, 435.0908.

1.3.6 One-pot synthesis and characterization

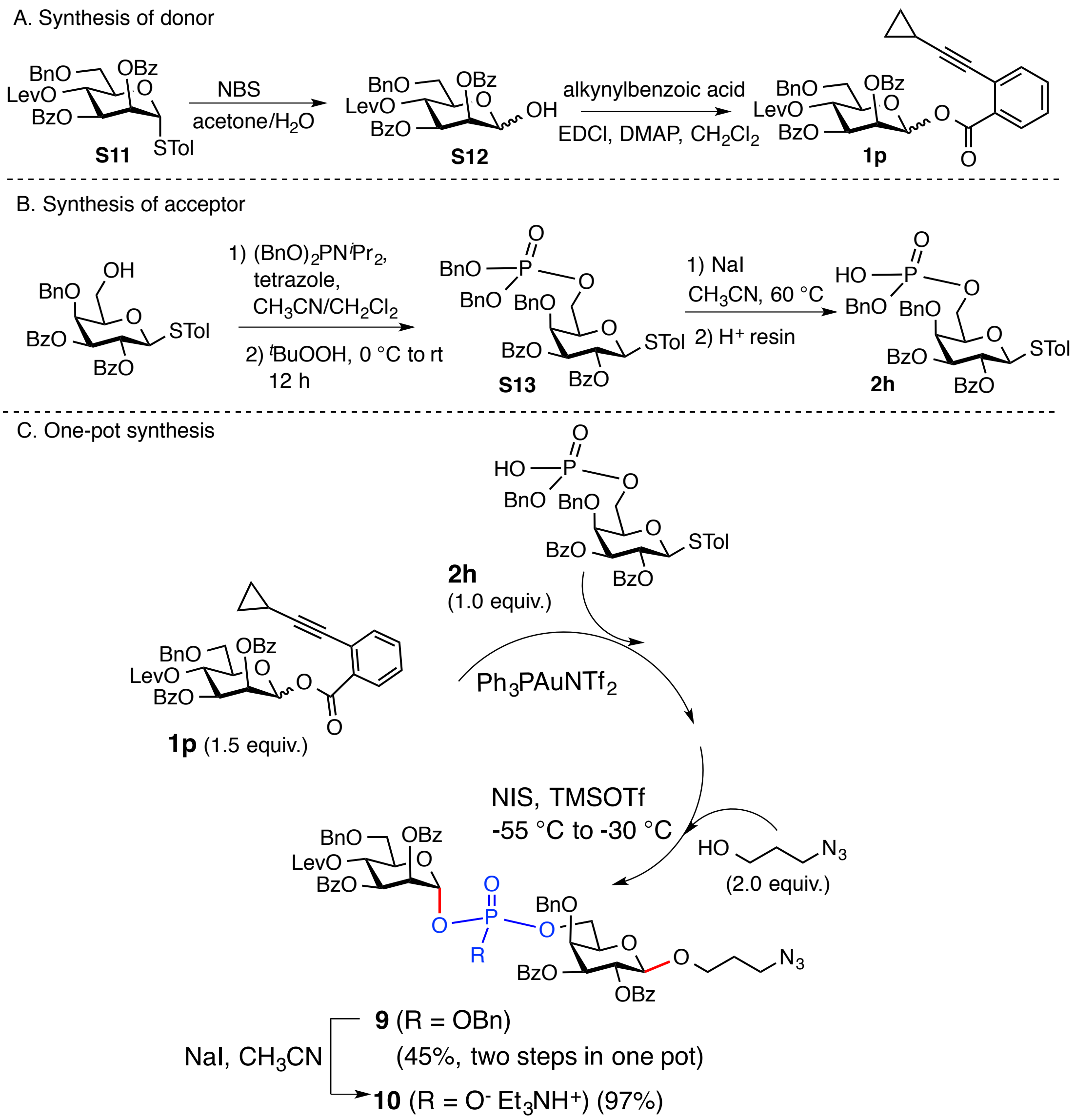

a: Yields of isolated products and yield of **9** was calculated based on donor **1p**. NIS: *N*-iodosuccinimide. TMSOTf: trimethylsilyl trifluoromethanesulfonate. Tol: tolyl. Lev: levulinoyl. Bn: benzyl. Bz: benzoyl.

6-*O*-benzyl-2,3-di-*O*-benzoyl-4-*O*-levulinyl-*α/β*-D-mannopyranose (S12)

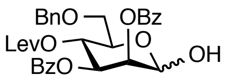

To a solution of **S11** (1.3 g, 1.9 mmol) in mixed acetone and H_2O (10 mL/2 mL) was added *N*-bromosuccinimide (NBS) (1.1 g, 3.0 equiv.) at room temperature. After stirred for 2 h, the mixture was quenched with aqueous $Na_2S_2O_3$, diluted with EtOAc, washed with H_2O, saturated $NaHCO_3$, brine and dried over Na_2SO_4. After filtration, the filtrate was concentrated to give a residue, which was purified via flash silica gel chromatography (Hexanes/EtOAc, 4/1→2/1) to give product **S12** (0.98 g, 89% yield). R_f= 0.2 (Hexanes/EtOAc, 2/1). ^{1}H NMR (400 MHz, $CDCl_3$) δ 8.03-7.29 (m, 15H, Ar*H*),

5.77 (dd, *J* = 10.1, 3.3 Hz, 1H, H-3), 5.67 (t, *J* = 10.0 Hz, 1H, H-4), 5.61 (dd, *J* = 3.3, 1.9 Hz, 1H, H-2), 5.41 (dd, *J* = 3.8, 1.9 Hz, 1H, H-1), 4.60 (q, *J* = 11.7 Hz, 2H, C*H*$_2$Ph), 4.37 (ddd, *J* = 10.0, 5.2, 3.1 Hz, 1H, H-5), 3.97 (bs, 1H, O*H*), 3.76-3.67 (m, 2H, H-6, H-6'), 2.03 (s, 3H, C*H*$_3$); ^{13}C NMR (101 MHz, CDCl$_3$) δ = 206.1, 171.9, 165.7, 165.6, 138.0, 133.5, 133.3, 130.0, 129.9, 129.51, 129.45, 128.6, 128.5, 128.2, 127.8, 92.3, 73.9, 71.0, 70.0, 69.9, 69.3, 67.2, 38.0, 29.7, 28.1; HRMS (m/z): [M+H$^+$]$^+$ calcd. for $C_{32}H_{33}O_{10}^+$, 577.2068; found, 577.2068.

6-*O*-benzyl-2,3-di-*O*-benzoyl-4-*O*-levulinyl-*α/β*-D-mannopyranosyl *ortho*-cyclopropylethynylbenzoate (1p)

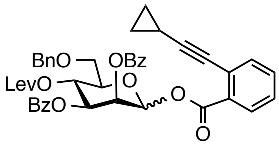

To a solution of **S12** (576 mg, 1.0 mmol) in CH_2Cl_2 (10 mL) was added *ortho*-cyclopropylethynylalkynyl benzoic acid (279 mg, 1.5 mmol), DMAP (24 mg, 0.2 mmol) and 1-ethyl-3-(3-dimethylaminopropyl) carbodiimide hydrochloride (EDCl) (344 mg, 1.8 mmol), respectively. After stirring for 4 h at room temperature, the reaction mixture was diluted with EtOAc, washed with H_2O, saturated $NaHCO_3$ and brine, dried over Na_2SO_4. After filtration, the filtrate was concentrated to give a residue. The residue was purified via flash silica gel chromatography (Hexanes/EtOAc, 6/1 → 3/1) to afford the donors **1p** (707 mg, 95% yield, α/β = 4.5/1). α-anomer: R_f = 0.25 (Hexanes/EtOAc, 2/1). $[\alpha]_D^{25}$ = -17.5 (*c* 2.0, CHCl$_3$); ^{1}H NMR (400 MHz, CDCl$_3$) δ 8.06-7.28 (m, 19H, Ar*H*), 6.59 (d, *J* = 2.0 Hz, 1H, H-1), 5.97 (t, *J* = 9.9 Hz, 1H, H-4), 5.91 (dd, *J* = 10.2, 3.2 Hz, 1H, H-3), 5.82 (dd, *J* = 3.1, 2.0 Hz, 1H, H-2), 4.73-4.54 (m, 2H, CH$_2$Ph), 4.39 (dt, *J* = 9.7, 3.3 Hz, 1H, H-5), 3.88-3.73 (m, 2H, H-6, H-6'), 2.63-2.29 (m, 4H, C*H*$_2$C*H*$_2$COCH$_3$), 2.00 (s, 3H, C*H*$_3$), 1.59-1.56 (m, 1H, CH$_2$C*H*CH$_2$), 0.92-0.86 (m, 4H, C*H*$_2$CHC*H*$_2$); ^{13}C NMR (101 MHz, CDCl$_3$) δ = 206.0, 171.7, 165.6, 165.3, 163.8, 138.3, 134.9, 133.6, 133.4, 132.5, 131.0, 130.4, 130.1, 130.0, 129.3, 129.2, 128.7, 128.5, 128.4, 128.0, 127.6, 127.4, 125.3, 100.4, 91.7, 74.7, 73.8, 72.7, 70.2, 69.5, 68.7, 66.6, 37.9, 29.7, 28.0, 9.2, 9.2, 0.9; HRMS (m/z): [M+Na$^+$]$^+$ calcd. for $C_{44}H_{40}NaO_{11}^+$, 767.2463; found, 767.2461. β-anomer: R_f = 0.2 (Hexanes/EtOAc, 2/1). $[\alpha]_D^{25}$ = -100 (*c* 2.0, CHCl$_3$); ^{1}H NMR (400 MHz, CDCl$_3$) δ 6.30 (d, *J* = 1.1 Hz, 1H, H-1), 5.99 (dd, *J* = 3.2, 1.1 Hz, 1H, H-2), 5.83 (t, *J* = 9.9 Hz, 1H, H-4), 5.56 (dd, *J* = 10.0, 3.2 Hz, 1H, H-3), 4.74-4.49 (m, 2H, C*H*$_2$Ph), 4.03 (ddd, *J* = 9.8, 4.3, 2.9 Hz, 1H, H-5), 3.85 (dd, *J* = 11.1, 2.9 Hz, 1H, H-6), 3.79 (dd, *J* = 11.1, 4.4 Hz, 1H, H-6'), 2.62-2.33 (m, 4H,

$CH_2CH_2COCH_3$), 2.01 (s, 3H, CH_3), 1.50-1.43 (m, 1H, CH_2CHCH_2), 0.86-0.82 (m, 4H, CH_2CHCH_2); ^{13}C NMR (101 MHz, $CDCl_3$) δ = 206.0, 171.7, 165.7, 165.6, 163.2, 138.2, 134.3, 133.49, 133.47, 132.3, 130.8, 130.2, 130.0, 129.9, 129.5, 129.1, 128.6, 128.5, 128.4, 128.0, 127.7, 127.0, 125.8, 100.5, 91.4, 74.9, 74.3, 73.8, 71.9, 69.5, 68.8, 66.6, 37.9, 29.6, 28.1, 9.1, 0.8; HRMS (m/z): [M+Na$^+$]$^+$ calcd. for $C_{44}H_{40}NaO_{11}^+$, 767.2463; found, 767.2461.

***p*-tolyl 4-*O*-benzyl-2,3-di-*O*-benzoyl-6-*O*-dibenzyloxyphosphoryl-1-thio-*β*-D-galactopyranoside (S13)**

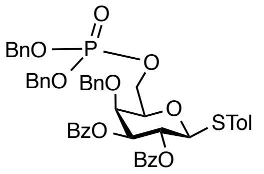

To a solution of alcohol (610 mg, 1.04 mmol) in CH_3CN (2 mL) and CH_2Cl_2 (8 mL) was added $(BnO)_2PN^iPr_2$ (523 μL, 1.56 mmol) and tetrazole (132 mg, 1.87 mmol), and the mixture was stirred for 2 h at room temperature, after which the alcohol disappeared on TLC plate. Then, *t*BuOOH (70% in H_2O, 286 μL, 2.08 mmol) was added into the mixture at 0 °C, and the reaction was warmed up slowly to room temperature and stirred overnight. After completion, the reaction was quenched with $Na_2S_2O_3$ solution. After removing the CH_3CN by evaporation, the mixture was diluted with EtOAc, washed with H_2O, saturated $NaHCO_3$, brine and dried over Na_2SO_4. After filtration, the filtrate was concentrated to give a residue, which was purified via flash silica gel column chromatography (Hexanes/EtOAc, 3/1 to 3/2) to give the desired product **S13**. R_f= 0.2 (Hexanes/EtOAc, 3/2), white foam, 500 mg, 97% yield; $[\alpha]_D^{25}$ = 45.7 (*c* 2.0, $CHCl_3$); ^{1}H NMR (600 MHz, $CDCl_3$) δ 7.96-7.01 (m, 30H, Ar*H*), 5.77 (t, *J* = 9.9 Hz, 1H, H-2), 5.33-5.25 (m, 1H, H-3), 5.09-4.97 (m, 4H, 2x CH_2Ph), 4.77 (d, *J* = 10.0 Hz, 1H, H-1), 4.63 (d, *J* = 11.4 Hz, 1H, C*H*Ph), 4.42 (d, *J* = 11.4 Hz, 1H, C*H*Ph), 4.21 (ddd, *J* = 10.4, 8.2, 6.4 Hz, 1H), 4.03 (dd, *J* = 13.0, 4.9 Hz, 2H, H-4), 3.73 (t, *J* = 6.5 Hz, 1H, H-5), 2.29 (s, 3H, CH_3); ^{13}C NMR (101 MHz, $CDCl_3$) δ = 165.9, 165.3, 138.3, 137.6, 135.9, 135.8, 133.6, 133.3, 130.0, 129.9, 129.7, 129.1, 128.8, 128.7, 128.6, 128.5, 128.4, 128.2, 128.2, 127.9, 127.8, 127.8, 127.1, 86.9, 76.7, 75.7, 75.0, 73.8, 69.7 (2C, d, *J* =5.3 Hz),

68.4, 65.3 (d, J = 5.3 Hz), 21.3; ^{31}P NMR (162 MHz, $CDCl_3$) δ = -1.0; HRMS (m/z): $[M+H^+]^+$ calcd. for $C_{48}H_{46}O_{10}PS^+$, 845.2544; found, 845.2539.

***p*-tolyl 4-*O*-benzyl-2,3-di-*O*-benzoyl-6-*O*-(benzyloxy-hydroxy-phosphoryl)-1-thio-*β*-D-galactopyranoside (2h)**

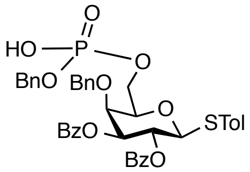

2h was prepared via the general procedure (E), R_f = 0.25 (CH_2Cl_2/MeOH, 10/1), white solid, 200 mg, 86% yield; $[\alpha]_D^{25}$ = 66.4 (c 2.0, $CHCl_3$); ^{1}H NMR (400 MHz, $CDCl_3$) δ 7.94-7.01 (m, 35H, Ar*H*), 5.78 (t, J = 10.0 Hz, 1H, H-2), 5.32 (dd, J = 10.0, 2.9 Hz, 1H, H-3), 5.07-4.99 (m, 2H, C*H*$_2$Ph), 4.80 (d, J = 9.9 Hz, 1H, H-1), 4.62 (d, J = 11.3 Hz, 1H, C*H*Ph), 4.46 (d, J = 11.3 Hz, 1H, C*H*Ph), 4.24 (dt, J = 10.2, 6.6 Hz, 1H, H-6), 4.12-4.07 (m, 2H, H-4, H-6'), 3.84 (t, J = 6.5 Hz, 1H, H-5), 2.28 (s, 3H, C*H*$_3$); ^{13}C NMR (101 MHz, $CDCl_3$) δ = 165.9, 165.3, 138.3, 137.7, 133.5, 133.3, 133.2, 130.0, 129.9, 129.8, 129.7, 129.1, 128.8, 128.7, 128.6, 128.6, 128.4, 128.4, 128.1, 128.0, 127.8, 86.7, 76.6, 75.8, 75.0, 73.7, 69.5 (d, J = 4.9 Hz), 68.4, 65.1 (d, J = 5.1 Hz), 21.3; ^{31}P NMR (162 MHz, $CDCl_3$) δ = 0.4; HRMS (m/z): $[M-H^+]^-$ calcd. for $C_{41}H_{38}O_{10}PS^-$, 753.1929; found, 753.1928.

3-azidopropanyl 6-*O*-benzyl-2,3-di-*O*-benzoyl-4-*O*-levulinyl-*α*-D-mannopyranosyl benzyloxyphosphoryl-(→6)-4-*O*-benzyl-2,3-di-*O*-benzoyl-*β*-D-galactopyranoside (9)

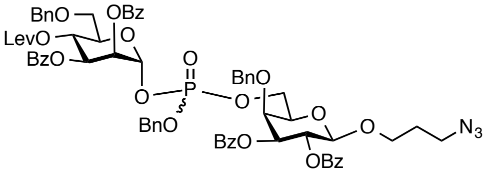

To a solution of donor **1p** (55.9 mg, 0.075 mmol) and acceptor **2h** (37.7 mg, 0.05 mmol) in $ClCH_2CH_2Cl$ (1.0 mL) was added $Ph_3PAuNTf_2$ (3.7 mg, 0.005 mmol) at 0 °C. After stirring for 0.5 h, the reaction was stirred at room temperature for 2 h. Then, the reaction mixture was cooled to -55 °C, and 3-azido-propanol (10 mg, 0.1 mmol), NIS (22 mg, 0.1 mmol) and TMSOTf (0.9 μL, 0.005 mmol) was subsequently added to the mixture at -55 °C. After warming up to -30 °C in one hour, the reaction was quenched with Et_3N (1.0 mL) and aqueous $Na_2S_2O_3$. After filtration, the filtrate was concentrated to give a residue, which was loaded to a silica gel column which was neutralized prior to use by using Hexanes/Et_3N (100/1), and purified (eluting solvent: Hexanes/EtOAc/Et_3N,

3/2/0.01) to give desired product **9** (43.2 mg, 45% yield calculated based on donor **1p**). R_f = 0.2 (Hexanes/EtOAc, 3/2). mixture of *R* and *S* anomers (R_P : S_P = 1/1): ^{1}H NMR (400 MHz, $CDCl_3$) δ 5.91-5.64 (m, 5H, H-2^A, H-3^A, H-1^B, H-2^B, H-4^B), 5.40-5.34 (m, 1H, H-3^B), 5.21-5.14 (m, 2H, C*H*$_2$Ph), 4.73-4.49 (m, 5H, 2 x C*H*$_2$Ph, H-1^A), 4.39-4.26 (m, 2H, H-6^A, H-5^B), 4.23-4.10 (m, 2H, H-6'^{A}, H-4^A), 4.00-3.88 (m, 2H, OC*H*(H)CH_2, H-5^A), 3.77-3.65 (m, 2H, H-6^B, H-6'^{B}), 3.57-3.50 (m, 1H, OC*H*(H)CH_2), 3.20-3.11 (m, 2H, C*H*$_2$N$_3$), 2.67-2.34 (m, 2H, C*H*$_2$C*H*$_2$COCH$_3$), 2.02 (s, 3H, C*H*$_3$), 1.77-1.58 (m, 2H, CH$_2$C*H*CH$_2$); ^{13}C NMR (101 MHz, $CDCl_3$) δ = 205.82, 205.79, 171.6, 165.8, 165.7, 165.5, 165.4, 165.2, 165.1, 138.2, 138.1, 137.43, 137.39, 135.5, 135.32, 135.25, 133.64, 133.59, 133.41, 133.38, 133.3, 133.2, 130.0, 129.92, 129.88, 129.7, 129.6, 129.2, 129.09, 129.06, 129.0, 128.9, 128.84, 128.80, 128.65, 128.61, 128.52, 128.50, 128.45, 128.42, 128.37, 128.24, 128.19, 127.91, 127.87, 127.6, 101.42, 101.39, 95.5, 95.4, 75.1, 74.2, 73.7, 73.65, 73.61, 73.4, 73.1, 73.0, 72.1, 72.0, 70.3, 70.2, 70.1, 69.8, 69.7, 69.42, 69.36, 68.4, 68.3, 66.5, 66.3, 65.9, 48.0, 37.9, 29.5, 29.0, 27.94, 27.92; ^{31}P NMR (162 MHz, $CDCl_3$) δ = -3.1, -3.3; HRMS (m/z): [M+Na$^+$]$^+$ calcd. for $C_{69}H_{68}N_3NaO_{20}P^+$, 1312.4026; found, 1312.4031.

3-azidopropanyl 6-*O*-benzyl-2,3-di-*O*-benzoyl-4-*O*-levulinyl-*α*-D-mannopyranosyl phosphoryl-(→6)-4-*O*-benzyl-2,3-di-*O*-benzoyl-*β*-D-galactopyranosyl phosphate triethylammonium salt (10)

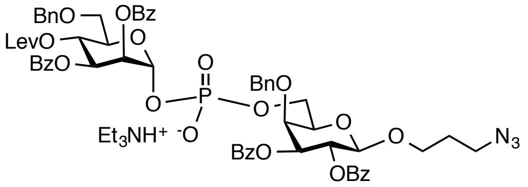

10 was prepared via procedure (E) and converted to triethylammonium salt by washing with triethylammonium bicarbonate buffer (1.0 M), R_f = 0.2 (CH_2Cl_2/MeOH, 8/1), colorless oil, 42.3 mg, 97% yield; $[\alpha]_D^{25}$ = -9.2 (*c* 4.1, $CHCl_3$); ^{1}H NMR (400 MHz, $CDCl_3$) δ 7.99-7.11 (m, 30H, Ar*H*), 5.87 (t, *J* = 9.9 Hz, 1H, H-2^A), 5.82-5.754 (m, 4H, H-1^B, H-2^B, H-4^B, H-3^A), 5.40 (dd, *J* = 10.5, 3.1 Hz, 1H, H-3^B), 4.75-4.67 (m, 3H, C*H*$_2$Ph, H-1^A), 4.61 (d, *J* = 11.7 Hz, 1H, C*H*Ph), 4.51 (d, *J* = 11.7 Hz, 1H, C*H*Ph), 4.45 (dt, *J* = 9.8, 3.3 Hz, 1H, H-5^B), 4.39-4.33 (m, 2H, H-4^A, H-6^A), 4.27-4.20 (m, 1H, H-6'^{A}), 4.14 (t, *J* = 6.7 Hz, 1H, H-5^A), 3.96 (dt, *J* = 10.5, 5.4 Hz, 1H, OC*H*(H)CH_2), 3.73 (d, *J* = 3.3 Hz, 2H, H-6^B, H-6'^{B}), 3.57 (ddd, *J* = 9.9, 8.0, 4.6 Hz, 1H, OC*H*(H)CH_2), 3.17 (td, *J* = 6.8, 2.3 Hz, 2H, C*H*$_2$N$_3$), 2.60-2.35 (m, 4H, C*H*$_2$C*H*$_2$COCH$_3$), 2.01 (s, 3H, C*H*$_3$), 1.79-1.63 (m, 2H, OCH$_2$C*H*$_2$CH$_2$); ^{13}C NMR (101 MHz, $CDCl_3$) δ = 206.0, 171.7,

165.8, 165.7, 165.52, 165.47, 138.4, 138.2, 133.3, 133.21, 133.16, 133.1, 130.0, 129.93, 129.87, 129.7, 129.54, 129.51, 129.3, 128.6, 128.44, 128.41, 128.39, 128.36, 128.2, 128.1, 128.0, 127.53, 127.49, 101.4, 93.9 (d, J = 4.6 Hz), 75.3, 74.5, 74.4, 73.8 (d, J = 6.6 Hz), 73.6, 70.9, 70.7 (d, J = 7.2 Hz), 70.5, 70.4, 69.0, 66.6, 66.3, 63.7 (d, J = 5.2 Hz), 48.1, 38.0, 29.6, 29.0, 28.1; ^{31}P NMR (162 MHz, $CDCl_3$) δ = -2.9; HRMS (m/z): $[M\text{-}Et_3NH^+]^-$ calcd. for $C_{62}H_{61}N_3O_{20}P^-$, 1198.3592; found, 1198.3586.

1.3.7 Synthesis of trisaccharide and characterization

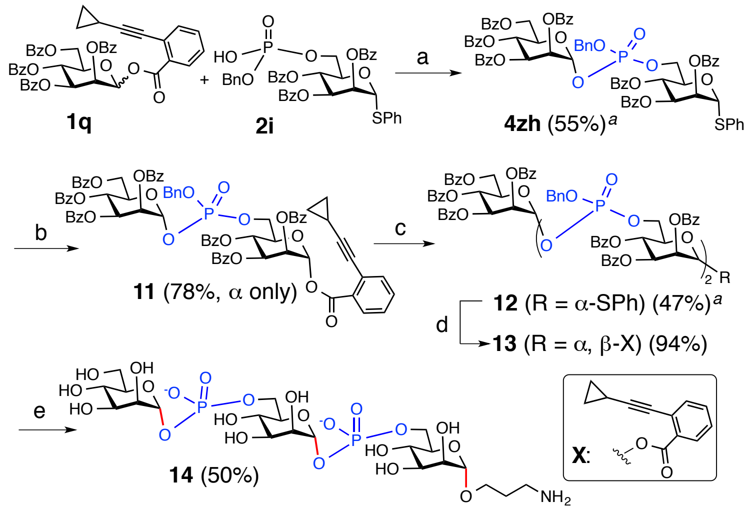

a) $Ph_3PAuNTf_2$ (10 mol%), 5Å MS, DCE. b) i. NBS, acetone, H_2O; ii. HX, EDCI, DMAP, CH_2Cl_2. c) **2i**, $Ph_3PAuNTf_2$, DCE. d) i. NBS, acetone, H_2O; ii. HX, EDCI, DMAP, CH_2Cl_2. e) i. 3-azidopropanol, $Ph_3PAuNTf_2$ (10 mol%); ii. H_2, Pd/C, HCOOH, $HCOONH_4$, THF, *t*-BuOH; iii. NH_2NH_2•H_2O, MeOH

^{a}: yields were calculated based on donor (1.5 equiv.). NBS: *N*-bromosuccinimide. EDCI: 1-(3-dimethylaminopropyl)-3-ethylcarbodiimide hydrochloride. DMAP: 4-(dimethylamino)pyridine. Bz: benzoyl.

2,3,4,6-tetra-*O*-benzoyl-*α/β*-D-mannopyranosyl *ortho*-cyclopropylethynylbenzoate (1q)

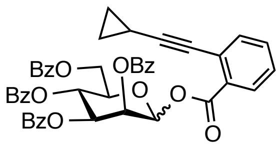

1q, prepared via procedure (C), purified via silica gel chromatography (Hexanes/EtOAc, 3/1), R_f = 0.2 (Hexanes/EtOAc, 3/1), white foam, 860 mg, 90 % yield, α/β = 3/1. α anomer: ^{1}H NMR (400 MHz, $CDCl_3$) δ 8.11-7.25 (m, 24H, Ar*H*), 6.65 (d, J = 2.2 Hz, 1H), 6.28 (t, J = 9.9 Hz, 1H, H-4), 6.12 (dd, J = 10.3, 3.1 Hz, 1H, H-3), 5.94 (bs, 1H, H-2), 4.75-4.50 (m, 3H, H-5, H-6, H-6’), 1.62-1.43 (m, 1H, CH_2CHCH_2), 0.92-0.83 (m, 4H, CH_2CHCH_2); β anomer: ^{1}H NMR (400 MHz, $CDCl_3$) δ 8.11-7.25 (m, 24H, Ar*H*), 6.44 (s, 1H), 6.28-6.11 (m, 2H, H-3, H-4), 5.80 (dd, J = 10.0, 3.2 Hz, 1H, H-2), 4.78-4.51 (m, H-6, H-6’), 4.42-4.29 (m, 1H, H-5), 1.62-1.43 (m, 1H, CH_2CHCH_2), 0.92-0.83 (m, 4H, CH_2CHCH_2); mixture of α and β anomers: ^{13}C NMR (101 MHz,

$CDCl_3$) δ = 166.25, 166.21, 165.7, 165.61, 165.59, 165.46, 165.42, 165.3, 163.9, 163.2, 134.8, 134.3, 133.8, 133.7, 133.62, 133.58, 133.50, 133.4, 133.2, 132.6, 132.4, 130.91, 130.87, 130.4, 130.13, 130.08, 129.97, 129.94, 129.91, 128.87, 129.84, 129.6, 129.2, 129.0, 128.93, 128.87, 128.79, 128.73, 128.61, 128.57, 128.49, 127.5, 127.0, 125.8, 125.3, 100.6, 100.4, 91.8, 91.4, 74.7, 73.5, 71.8, 71.4, 70.1, 69.7, 69.6, 66.6, 62.9, 62.5, 9.2, 9.1, 0.9, 0.8; HRMS (m/z): $[M+Na^+]^+$ calcd. for $C_{46}H_{36}NaO_{11}^+$, 787.2150; found, 787.2148.

phenyl 2,3,4-tri-*O*-benzoyl-6-*O*-dibenzyloxyphosphoryl-1-thio-*α*-D-mannopyranoside (S14)

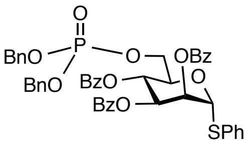

S14, prepared via the same procedure as that for compound **S9**, purified via silica gel chromatography (Hexanes/EtOAc, 2/1→3/2), R_f = 0.2 (Hexanes/EtOAc, 3/2), 600 mg, 95% yield. $[\alpha]_D^{25}$ = -6.0 (*c* 2.3, $CHCl_3$); ^{1}H NMR (400 MHz, $CDCl_3$) δ 8.07-7.19 (m, 30H, Ar*H*), 5.99 (t, *J* = 10.0 Hz, 1H, H-4), 5.93 (dd, *J* = 3.2, 1.6 Hz, 1H, H-2), 5.81 (dd, *J* = 10.0, 3.2 Hz, 1H, H-3), 5.68 (d, *J* = 1.6 Hz, 1H, H-1), 4.99-4.86 (m, 4H, 2 x C*H*$_2$Ph), 4.87-4.81 (m, 1H, H-5), 4.31-4.22 (m, 2H, H-6, H-6'); ^{13}C NMR (101 MHz, $CDCl_3$) δ = 165.54, 165.50, 165.47, 135.9, 135.8, 135.7, 133.7, 133.5, 132.8, 132.6, 130.0, 129.9, 129.4, 129.3, 129.0, 128.9, 128.8, 128.7, 128.62, 128.57, 128.52, 128.49, 128.4, 128.1, 127.9, 86.2, 72.1, 70.7 (d, *J* = 8.4 Hz), 70.5, 69.5 (d, *J* = 2.4 Hz), 69.4 (d, *J* = 2.4 Hz), 66.8, 66.0 (d, *J* = 5.1 Hz); ^{31}P NMR (162 MHz, $CDCl_3$) δ = -1.4; HRMS (m/z): $[M+H^+]^+$ calcd. for $C_{47}H_{42}O_{11}PS^+$, 845.2180; found, 845.2178.

phenyl 2,3,4-tri-*O*-benzoyl-6-*O*-(benzyloxy-hydroxy-phosphoryl)-1-thio-*α*-D-mannopyranoside (2i)

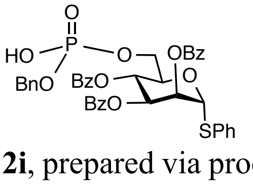

2i, prepared via procedure (E), R_f = 0.2 (CH_2Cl_2/MeOH, 10/1), white solid, 189 mg, 75% yield. $[\alpha]_D^{25}$ = -10.1 (*c* 1.4, $CHCl_3$); ^{1}H NMR (400 MHz, $CDCl_3$) δ 8.07-7.16 (m, 25H, Ar*H*), 6.01-5.88 (m, 2H, H-2, H-4), 5.84-5.74 (m, 4H, H-3, OH), 5.63 (s, 1H, H-1), 4.87 (dd, *J* = 7.1, 2.0 Hz, 2H, C*H*$_2$Ph), 4.81 (d, *J* = 6.7 Hz, 1H, H-5), 4.27-4.17 (m, 2H, H-6, H-6'); ^{13}C NMR (101 MHz, $CDCl_3$) δ = 165.6, 165.5, 135.8, 135.7, 133.72, 133.65, 133.4, 132.73, 132.69, 130.1, 130.0, 129.9, 129.5, 129.3, 129.01, 128.97, 128.8, 128.6,

128.52, 128.48, 128.4, 127.8, 86.2, 71.9, 70.7 (d, J = 8.7 Hz), 70.6, 69.2 (d, J = 5.4 Hz), 66.9, 66.0 (d, J = 4.9 Hz); ^{31}P NMR (162 MHz, $CDCl_3$) δ = 0.49; HRMS (m/z): $[M+H^+]^+$ calcd. for $C_{40}H_{36}O_{11}PS^+$, 755.1710; found, 755.1709.

phenyl 2,3,4,6-tetra-*O*-benzoyl-*α*-D-mannopyranosyl-benzyloxyphosphoryl-(→6)-2,3,4-tri-*O*-benzoyl-1-thio-*α*-D-mannopyranoside (4zh)

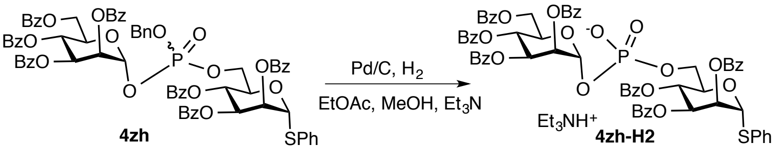

4zh, prepared via procedure (A), R_f = 0.2 (Hexanes/EtOAc, 3/2), colorless oil, 350 mg, 55% yield calculated based on donor **1q** (1.5 equiv.). The hydrogenated form (**4zh-H2)** via procedure (B) is here characterized instead of **4zh**. **4zh-H2**: $[\alpha]_D^{25}$ = -21.1 (*c* 2.3 $CHCl_3$); ^{1}H NMR (400 MHz, $CDCl_3$) δ 8.00-7.04 (m, 35H, Ar*H*), 6.05 (t, J = 10.1 Hz, 1H, H-4^B), 5.92-5.88 (m, 3H, H-2^B, H-3^B, H-4^A), 5.79-5.72 (m, 3H, H-1^B, H-2^A, H-3^A), 5.65 (s, 1H, H-1^A), 4.97-4.87 (m, 1H, H-5^A), 4.60-4.50 (m, 2H, H-5^B, H-6^B), 4.33-4.23 (m, 2H, H-6^A, H-6'^{A}), 4.12 (d, J = 11.9 Hz, 1H, H-6'^{B}); ^{13}C NMR (101 MHz, $CDCl_3$) δ = 166.1, 165.7, 165.6, 165.4, 165.2, 133.4, 133.3, 133.2, 133.1, 133.0, 132.9, 130.2, 130.1, 130.0, 129.94, 129.89, 129.84, 129.7, 129.5, 129.43, 129.35, 129.3, 129.25, 129.21, 128.62, 128.57, 128.5, 128.4, 128.3, 128.2, 94.0 (d, J = 4.5 Hz), 86.2, 72.1, 71.8 (d, J = 8.8 Hz), 71.0, 70.8 (d, J = 8.6 Hz), 70.3, 69.5, 67.4, 66.6, 65.1 (d, J = 4.9 Hz), 62.3; ^{31}P NMR (162 MHz, $CDCl_3$) δ = -3.2; HRMS (m/z): $[M-Et_3NH^++Na^++H^+]^+$ calcd. for $C_{67}H_{55}NaO_{20}PS^+$, 1265.2637; found, 1265.2644.

2,3,4,6-tetra-*O*-benzoyl-*α*-D-mannopyranosyl-benzyloxyphosphoryl-(→6)-2,3,4-tri-*O*-benzoyl-*α*-D-mannopyranosyl *ortho*-cyclopropylethynylbenzoate (11)

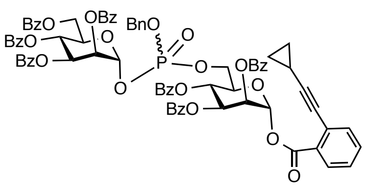

To a solution of compound **4zc** (300 mg, 0.22 mmol) in acetone/H_2O (10 mL/2 mL), *N*-bromosuccinimide (NBS) (313 mg, 1.76 mmol) was added at room temperature. After stirring for 0.5 h, the reaction was quenched with aqueous saturated $Na_2S_2O_3$ and $NaHCO_3$, and diluted with EtOAc, washed with H_2O, brine and dried over Na_2SO_4. After filtration, the filtrate was concentrated *in vacuo* to give a yellow residue. The resulting residue was loaded to silica gel column which was neutralized with solvent (Hexanes/Et_3N, 100/1), and purified with solvent (Hexanes/EtOAc/Et_3N, 4/1/0.01→ Hexanes/EtOAc/acetone/Et_3N 2/1/1/0.01) to give product (256 mg, 92%). The above

product was next installed *ortho*alkynyl benzoate to form glycsoysl donor. To a solution of disaccharide (256 mg, 0.21 mmol) in CH_2Cl_2 (2 mL) was added *ortho*-alkynyl benzoic acid (57 mg, 0.32 mmol), DMAP (5 mg, 0.04 mmol) and 1-ethyl-3-(3-dimethylaminopropyl)carbodiimide hydrochloride (EDCl) (71 mg, 0.37 mmol), respectively. After stirred for 2 h, the reaction mixture was diluted with EtOAc, washed with H_2O, saturated $NaHCO_3$ and brine, dried over Na_2SO_4. After filtration, the filtrate was concentrated to give a residue. The residue was loaded to silica gel column which was neutralized prior to use by using solvent (Hexanes/Et_3N, 100/1), and purified by eluting with solvent (Hexanes/EtOAc/Et_3N, 3/1/0.01 to 2/1/0.01) to afford the α/β mixed donors **11** (247 mg, 78% over two steps). R_f= 0.2 (Hexanes/EtOAc, 2/1). $[\alpha]_D^{25}$ = -32 (*c* 0.17 $CHCl_3$); ^1H NMR (400 MHz, $CDCl_3$) δ 8.15-8.72 (m, 78H, Ar*H*), 6.72 (s, 1H, H-1), 6.64 (s, 1H, H-1^{epimer}), 6.22-5.76 (m, 14H, H-1^B, H-$1^{Bepimer}$, H-2^A, H-3^A, H-4^A, H-2^B, H-3^B, H-4^B, H-$2^{Aepimer}$, H-$3^{Aepimer}$, H-$4^{Aepimer}$, H-$2^{Bepimer}$, H-$3^{Bepimer}$, H-$4^{Bepimer}$), 5.31-5.18 (m, 4H, 4 x C*H*Ph), 4.71-4.28 (m, 12H, H-6^A, H-6’^{A}, H-$6^{Aepimer}$, H-6’^{Aepimer}, H-6^B, H-6’^{B}, H-$6^{Bepimer}$, H-6’^{Bepimer}, H-5^A, H-5^B, H-$5^{Aepimer}$, H-$5^{Bepimer}$,), 1.64-1.55 (2H, 2 x CH_2C*H*CH_2), 0.91-0.80 (m, 8H, 2 x C*H*$_2$CHC*H*$_2$); ^{13}C NMR (101 MHz, $CDCl_3$) δ = 166.0, 165.49, 165.45, 165.4, 165.33, 165.29, 164.95, 164.91, 163.8, 135.6, 135.5, 135.3, 135.2, 134.79, 134.76, 133.73, 133.68, 133.65, 133.58, 133.51, 133.4, 133.2, 133.0, 132.5, 132.4, 131.0, 130.9, 130.3, 130.1, 130.03, 129.96, 129.90, 129.87, 129.83, 129.2, 129.1, 129.04, 129.00, 128.96, 128.79, 128.76, 128.72, 128.66, 128.55, 128.50, 128.46, 128.42, 128.35, 128.30, 128.2, 127.4, 127.3, 125.2, 100.4, 100.3, 95.6, 95.5, 91.70, 91.67, 74.8, 74.7, 72.0, 71.9, 70.7, 70.6, 70.42, 70.36, 70.0, 69.9, 69.8, 69.7, 69.5, 69.44, 69.37, 66.52, 66.45, 66.38, 66.0, 62.14, 62.08, 9.2, 9.1, 0.9; ^{31}P NMR (162 MHz, $CDCl_3$) δ = -3.1, -3.44; HRMS (m/z): $[M+Na^+]^+$ calcd. for $C_{80}H_{65}NaO_{22}P^+$, 1431.3597; found, 1431.3597.

phenyl 2,3,4,6-tetra-*O*-benzoyl-*α*-D-mannopyranosyl-benzyloxyphosphoryl-(→6)-2,3,4-tri-*O*-benzoyl-*α*-D-mannopyranosyl-benzyloxyphosphoryl-(→6)-2,3,4-tri-*O*-benzoyl-1-thio-*α*-D-mannopyranoside (12)

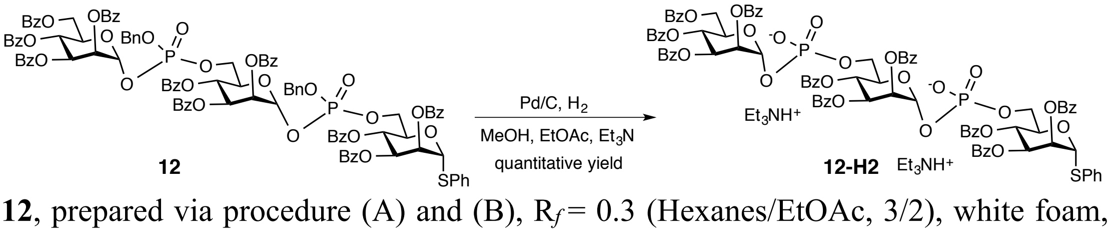

12, prepared via procedure (A) and (B), R_f= 0.3 (Hexanes/EtOAc, 3/2), white foam, 177 mg, 47% yield calculated based on donor **11** (1.5 equiv.). Because there are four

unseparated diastereomers which causing difficulty in characterization, benzyl groups of compound **12** were removed quantitatively via hydrogenolysis with Pd/C in mixed solvent of MeOH/EtOAc/Et_3N to afford a single stereomer (**12-H2**). **12-H2** is characterized as follows: $[\alpha]_D^{25}$ = -40.9 (*c* 2.2 $CHCl_3$); ^{1}H NMR (400 MHz, $CDCl_3$) δ 8.04-7.02 (m, 45H, Ar*H*), 6.08-5.65 (m, 12H, H-1^A, H-2^A, H-3^A, H-4^A, H-1^B, H-2^B, H-3^B, H-4^B, H-1^C, H-2^C, H-3^C, H-4^C), 4.94-4.54 (m, 3H, H-5^A, H-5^B, H-5^C), 4.49-4.14 (m, 6H, H-6^A, H-6'^{A}, H-6^B, H-6'^{B}, H-6^C, H-6'^{C}); ^{13}C NMR (101 MHz, $CDCl_3$) δ = 166.1, 165.72, 165.67, 165.59, 165.57, 165.45, 165.3, 165.23, 165.18, 165.08, 133.40, 133.36, 133.24, 133.18, 133.08, 133.01, 132.97, 132.8, 130.3, 130.15, 130.12, 130.0, 129.93, 129.90, 129.82, 129.78, 129.68, 129.58, 129.47, 129.41, 129.29, 129.25, 129.21, 128.7, 128.60, 128.56, 128.50, 128.44, 128.37, 128.32, 128.2, 93.9, 93.87, 93.79, 93.74, 86.1, 72.1, 71.75, 71.67, 71.1, 70.9, 70.2, 69.5, 67.6, 67.5, 66.7, 65.4, 65.0, 62.4; ^{31}P NMR (162 MHz, $CDCl_3$) δ = -3.3, -3.7; HRMS (m/z): $[M-2Et_3NH^+]^{2-}$ calcd. for $C_{94}H_{76}O_{31}P_2S^{2-}$, 897.6806; found, 897.6800.

2,3,4,6-tetra-*O*-benzoyl-*α*-D-mannopyranosyl-benzyloxyphosphoryl-(→6)-2,3,4-tri-*O*-benzoyl-*α*-D-mannopyranosyl-benzyloxyphosphoryl-(→6)-2,3,4-tri-*O*-benzoyl-*α/β*-D-mannopyranosyl *ortho*-cyclopropylethynylbenzoate (13)

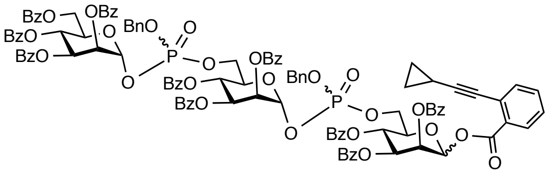

13, was prepared by using a similar procedure as that for compound **11**, and purified via silica gel column which was neutralized by Hexanes/Et_3N (100/1) (eluting solvent: Hexanes/EtOAc/Et_3N, 3/1/0.01 to 2/1/0.01), R_f = 0.2 (Hexanes/EtOAc, 3/2), white foam, 107 mg, 94% yield. ^{1}H NMR (400 MHz, $CDCl_3$) δ 8.14-7.14 (m, 64H, Ar*H*), 6.69-6.45 (m, 1H, H-1^A), 6.2-5.7 (m, 11H, H-2^A, H-3^A, H-4^A, H-1^B, H-2^B, H-3^B, H-4^B, H-1^C, H-2^C, H-3^C, H-4^C), 5.28-5.06 (m, 4H, 2 x CH_2Ph), 4.70-4.20 (m, 9H, H-5^A, H-6^A, H-6'^{A}, H-5^B, H-6^B, H-6'^{B}, H-5^C, H-6^C, H-6'^{C}); ^{13}C NMR (101 MHz, $CDCl_3$) δ = 166.01, 165.81, 165.55, 165.44, 165.37, 165.3, 164.9, 135.6, 135.4, 135.3, 134.8, 134.3, 133.8, 133.7, 133.6, 133.5, 133.4, 133.3, 133.24, 133.21, 133.08, 132.4, 131.04, 130.95, 130.42, 130.37, 130.2, 130.14, 130.7, 130.00, 129.93, 129.88, 129.3, 129.22, 129.17, 129.10, 129.06, 129.0, 128.9, 128.84, 128.79, 128.73, 128.70, 128.65, 128.62, 128.55, 128.51, 128.45, 128.40, 128.36, 128.31, 128.29, 128.25, 127.43, 127.38, 125.3, 100.4, 100.3, 95.6, 91.8, 74.8, 72.0, 71.2, 70.7, 70.61, 70.56, 70.4, 70.3, 70.1, 69.7, 69.6, 69.4,

66.4, 66.3, 66.1, 65.8, 62.0, 9.2, 0.9; ^{31}P NMR (162 MHz, $CDCl_3$) δ = -2.9, -3.0, -3.1, -3.2, -3.3, -3.3, -3.4, -3.5; HRMS (m/z): $[M+H^+]^+$ calcd for $C_{114}H_{95}O_{33}P_2^+$, 2053.5225; found, 2053.2539.

3-aminopropanyl *α*-D-mannopyranosyl-phosphoryl-(→6)-*α*-D-mannopyranosyl-phosphoryl-(→6)-*α*-D-mannopyranoside bis-ammonium salt (14)

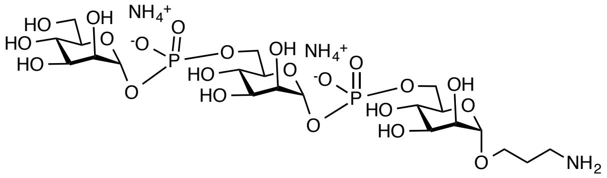

Glycosylation reaction. To a solution of donor **13** (67 mmol, 0.033 mmol) and 3-azido-propanol (6.6 mg, 0.066 mmol) in $ClCH_2CH_2Cl$ (0.6 mL) was added $Ph_3PAuNTf_2$ (2.4 mg, 0.0033 mmol) at 0 °C. After stirring for 0.5 h, the reaction was stirred at room temperature for 2 h. Then, the mixture was filtrated, and the filtrate was concentrated to give a residue, which was loaded to a silica gel column which was neutralized prior to use by using Hexanes/Et_3N (100/1), and purified (eluting solvent: Hexanes/EtOAc/Et_3N, 3/1/0.01→Hexanes/EtOAc/acetone/Et_3N, 2/1/0.5/0.01) to give the desired product of linker-tethered trisaccharide (45 mg, 70% yield).

Deprotection. To a solution of protected trisaccharide (20 mg) in THF/^{t}BuOH (1.0 mL/2.0 mL) was added $HCOONH_4$ (14 mg), HCOOH (30 μL) and Pd/C (60 mg). Then, the mixture was stirred under an atmosphere of H_2 at room temperature for 5 h. After filtration through syringe filter, the filtrate was concentrated to give a residue. The resulting residue was dissolved in $MeOH/NH_2NH_2•H_2O$ (1.0 mL/0.4 mL), and the resulting mixture was stirred for 12 h at room temperature, after which the solvent was removed via evaporation to provide the crude product. Afterwards, the product was purified via P2 polyacrylamide gel column (45x0.7 cm, eluent: 10 mM aqueous NH_4HCO_3) to give deprotected amino-tethered trisaccharide **14** (5.2 mg, 71%). $[\alpha]_D^{25}$ = -306 (*c* 0.26 $CHCl_3$); ^{1}H NMR (800 MHz, D_2O) δ 5.44-5.21 (m, 2H), 4.78 (s, 1H), 4.19-4.11 (m, 1H), 4.06 (dd, *J* = 5.5, 3.1 Hz, 2H), 3.97-3.90 (m, 3H), 3.86 (dd, *J* = 3.4, 1.7 Hz, 1H), 3.84-3.76 (m, 5H), 3.75-3.67 (m, 5H), 3.60 (t, *J* = 9.8 Hz, 1H), 3.57-3.54 (m, 2H), 3.13-2.99 (m, 2H), 1.93 (p, *J* = 6.7 Hz, 2H); ^{13}C NMR (201 MHz, D_2O) δ = 99.9, 96.2, 96.2, 73.7, 72.6, 71.9, 70.5, 70.46, 70.45, 70.41, 69.9, 69.8, 69.7, 66.5, 66.3, 65.8, 65.2, 65.1, 64.5, 60.7, 37.4, 26.7; ^{31}P NMR (162 MHz, $CDCl_3$) δ = -1.8, -2.0; HRMS (m/z): $[M-2NH_4^+ + H^+]^-$ calcd for $C_{21}H_{40}NO_{22}P_2^-$, 720.1523; found, 720.1521.

1.3.8 Supplementary Figures

NMR spectra of new compounds:

Supplementary Figure 3. ^{1}H NMR spectrum of compound **3a**

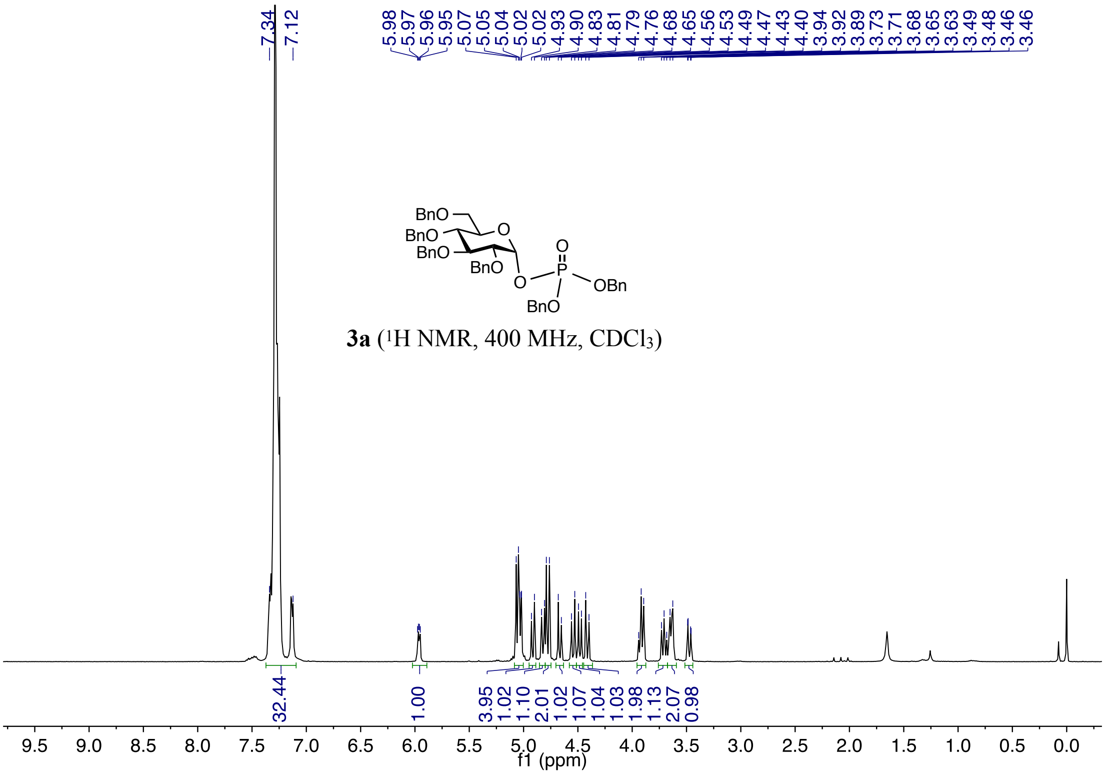

Supplementary Figure 4. ^{13}C NMR spectrum of compound **3a**

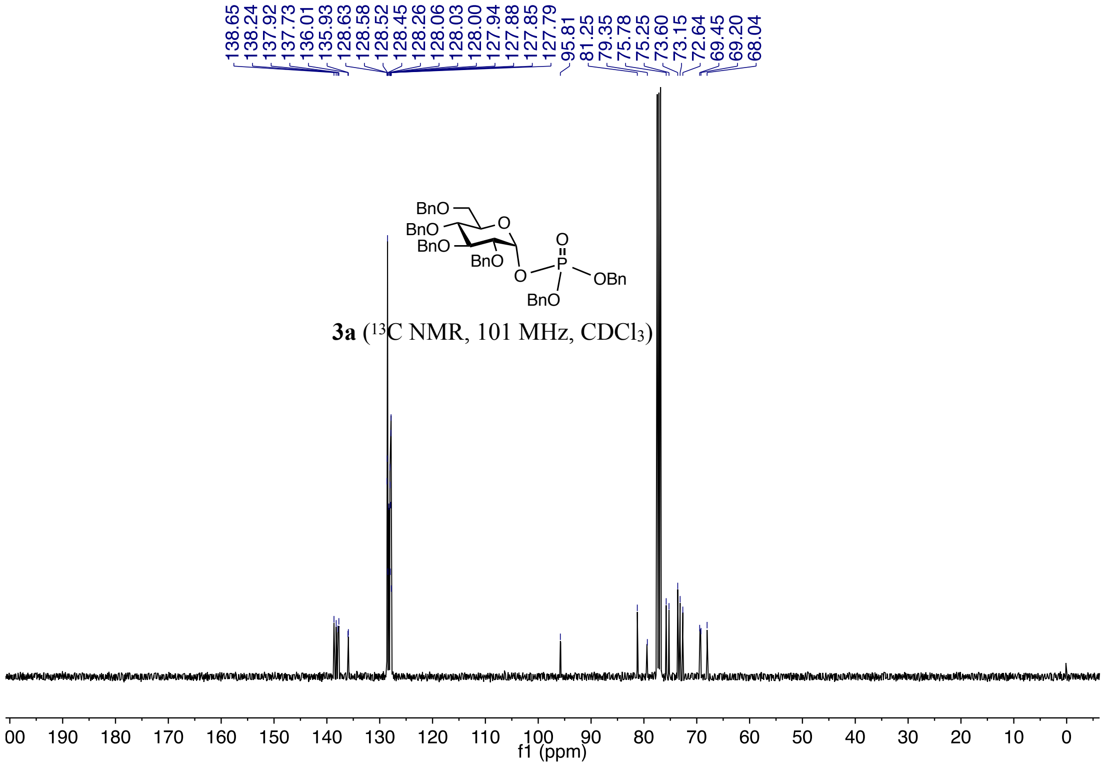

Supplementary Figure 5. ^{31}P NMR spectrum of compound **3a**

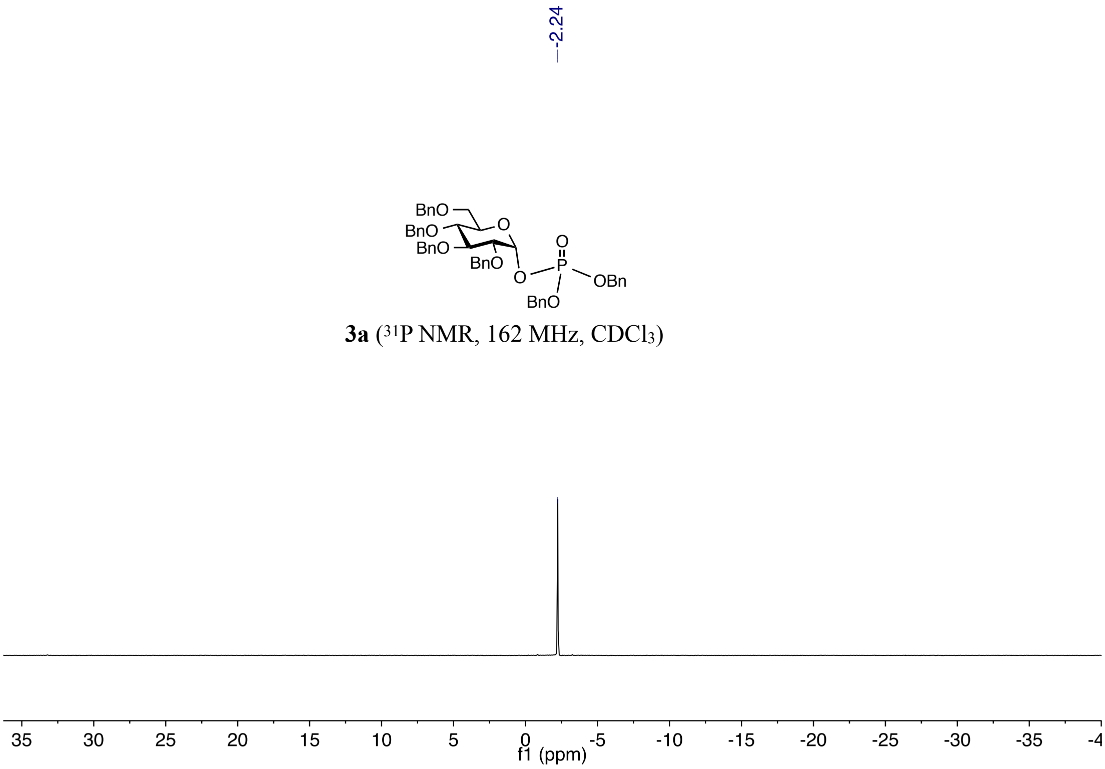

Supplementary Figure 6. ^{1}H NMR spectrum of compound **3b**

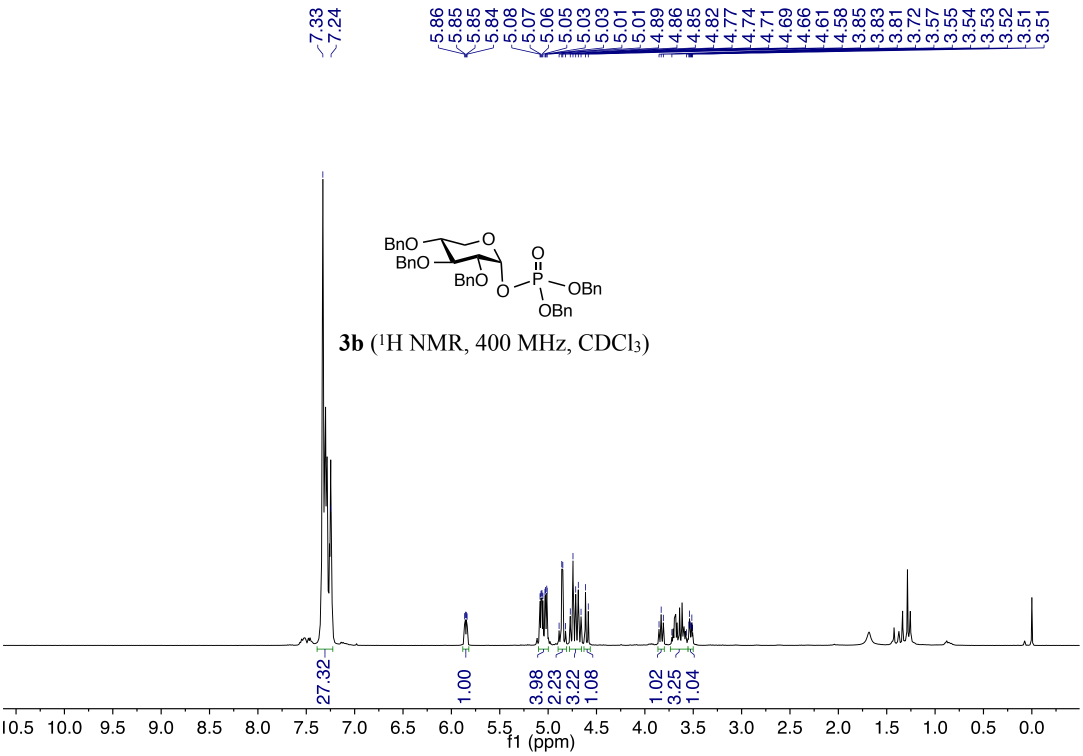

Supplementary Figure 7. ^{13}C NMR spectrum of compound **3b**

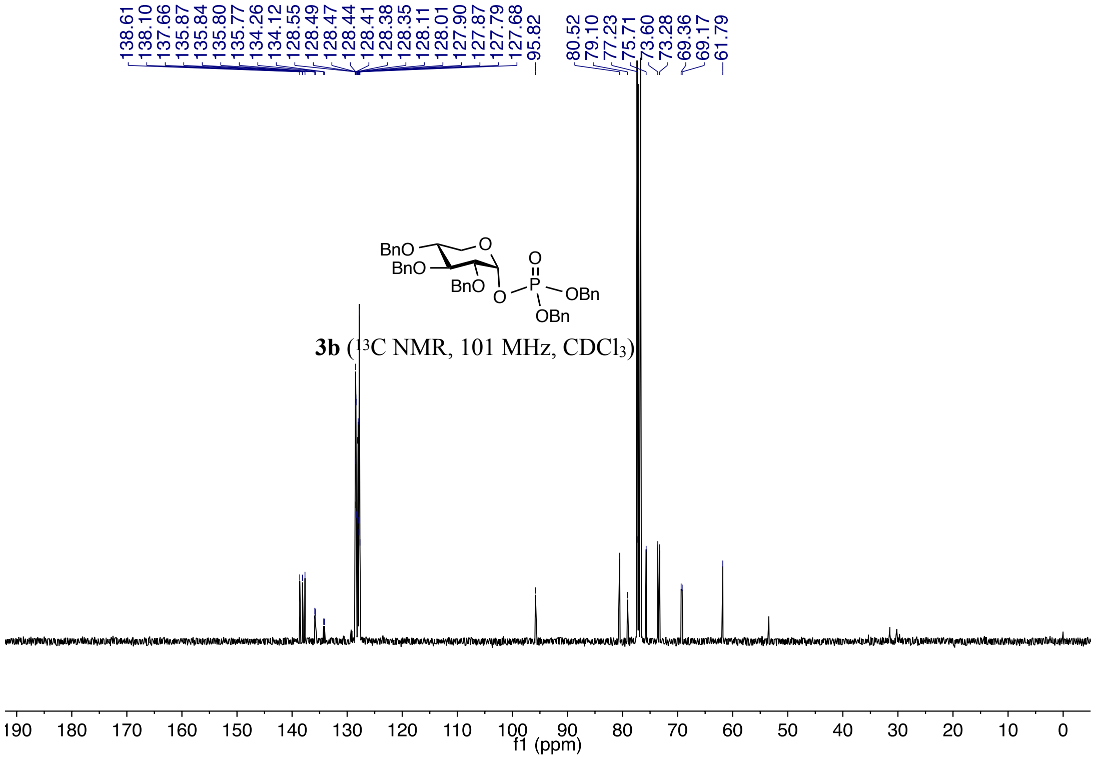

Supplementary Figure 8. ^{31}P NMR spectrum of compound **3b**

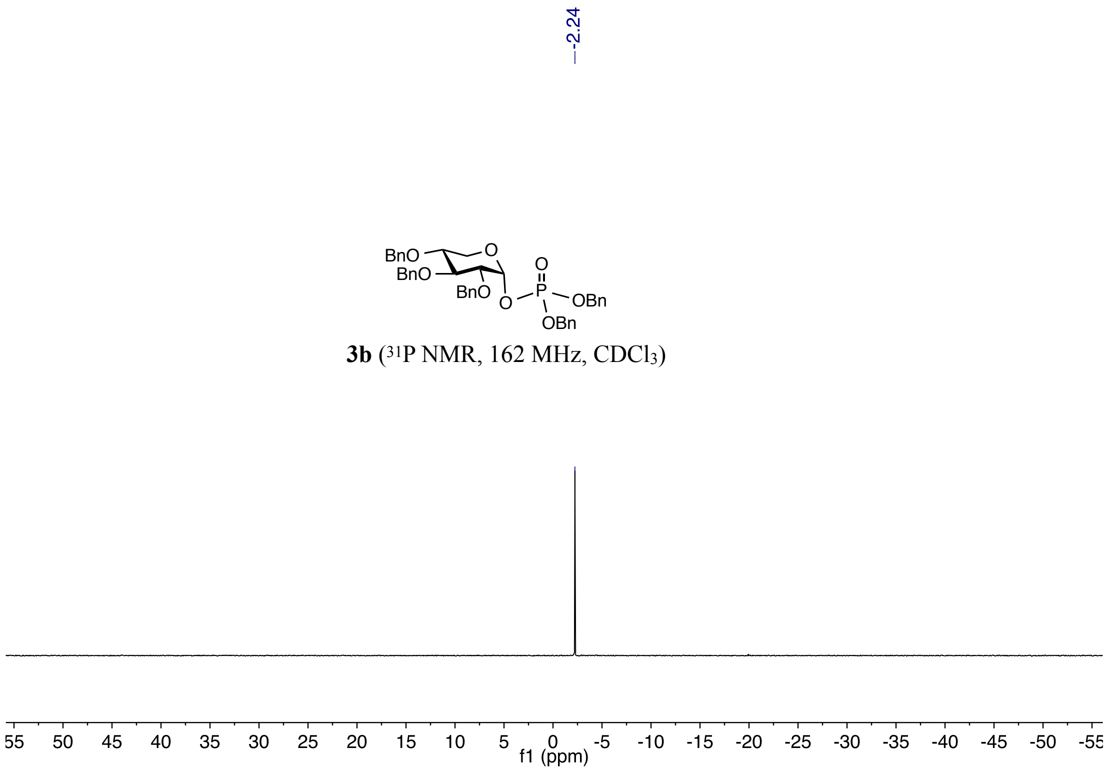

Supplementary Figure 9. ^{1}H NMR spectrum of compound **3c**

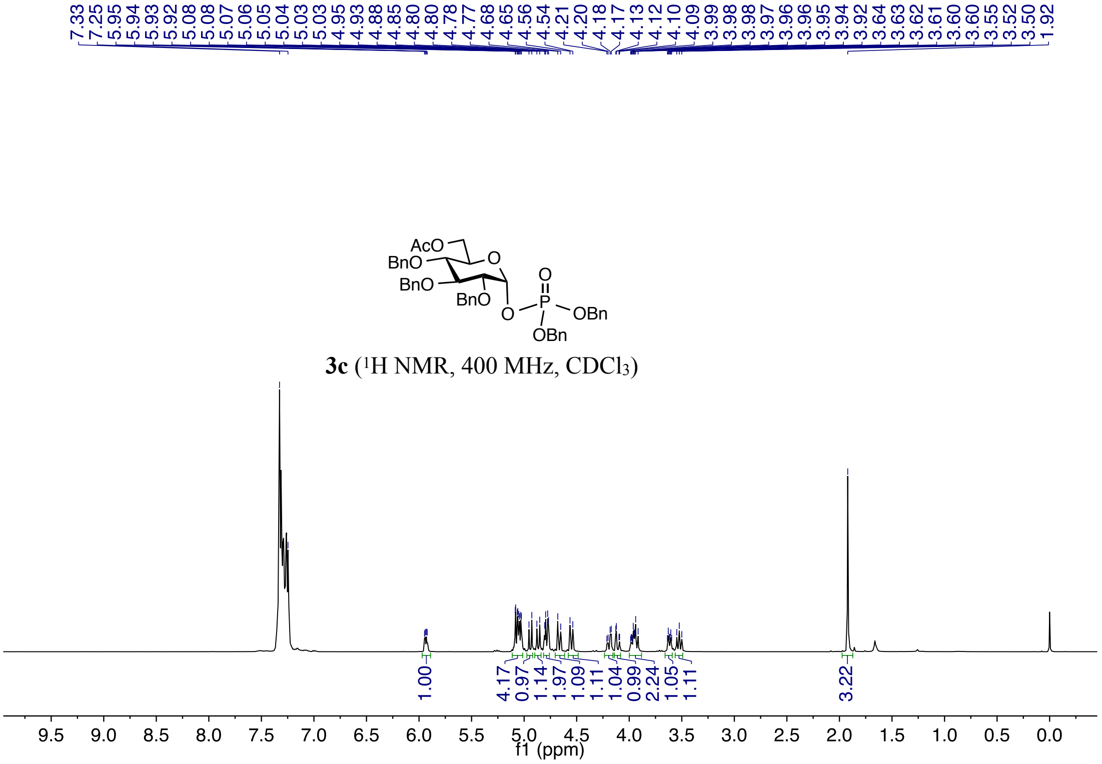

Supplementary Figure 10. ^{13}C NMR spectrum of compound **3c**

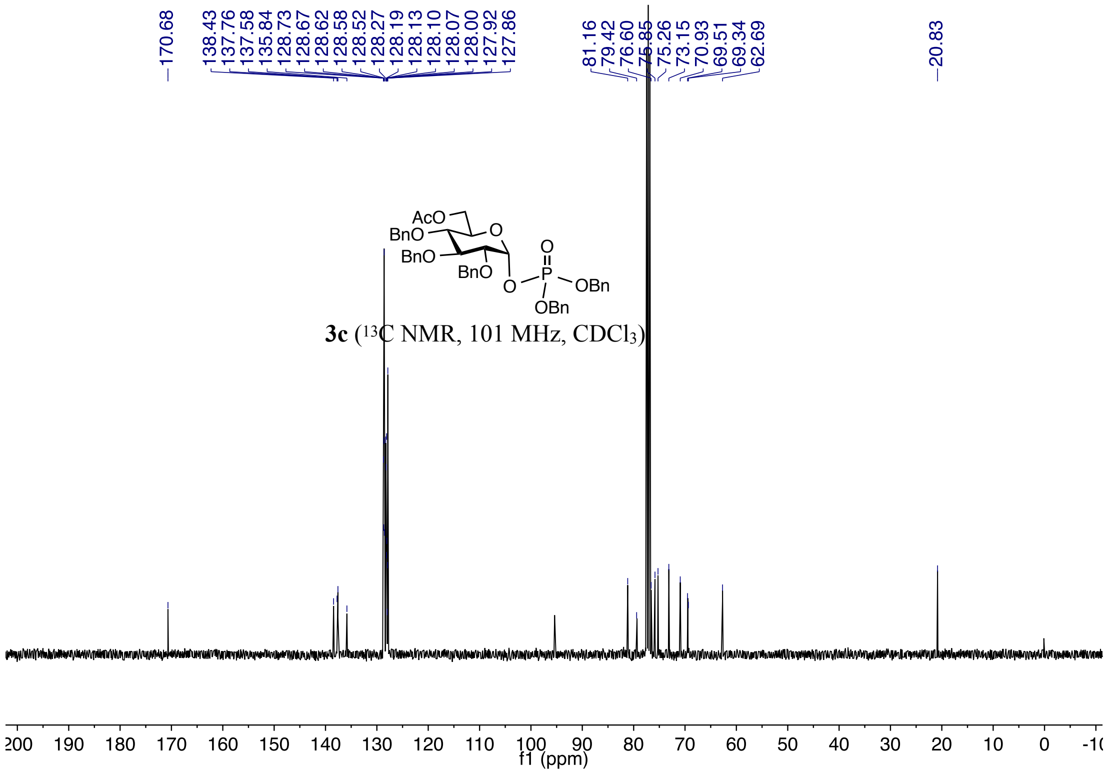

Supplementary Figure 11. ^{31}P NMR spectrum of compound **3c**

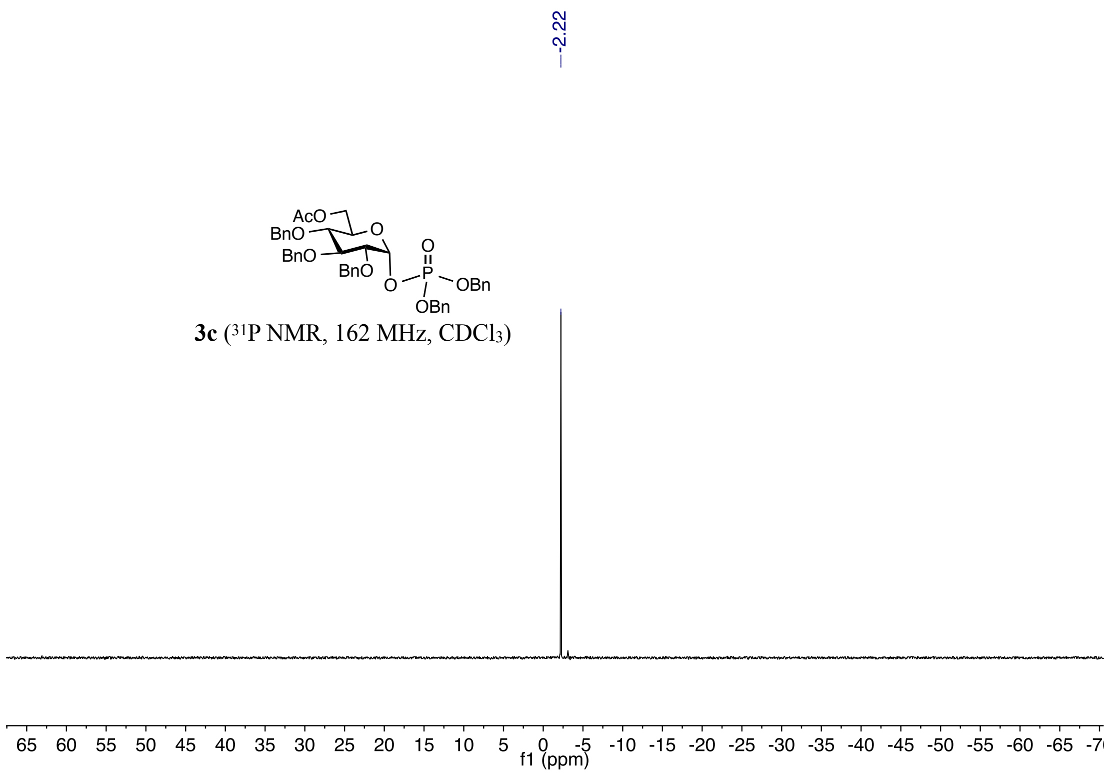

Supplementary Figure 12. ^{1}H NMR spectrum of compound **3**d

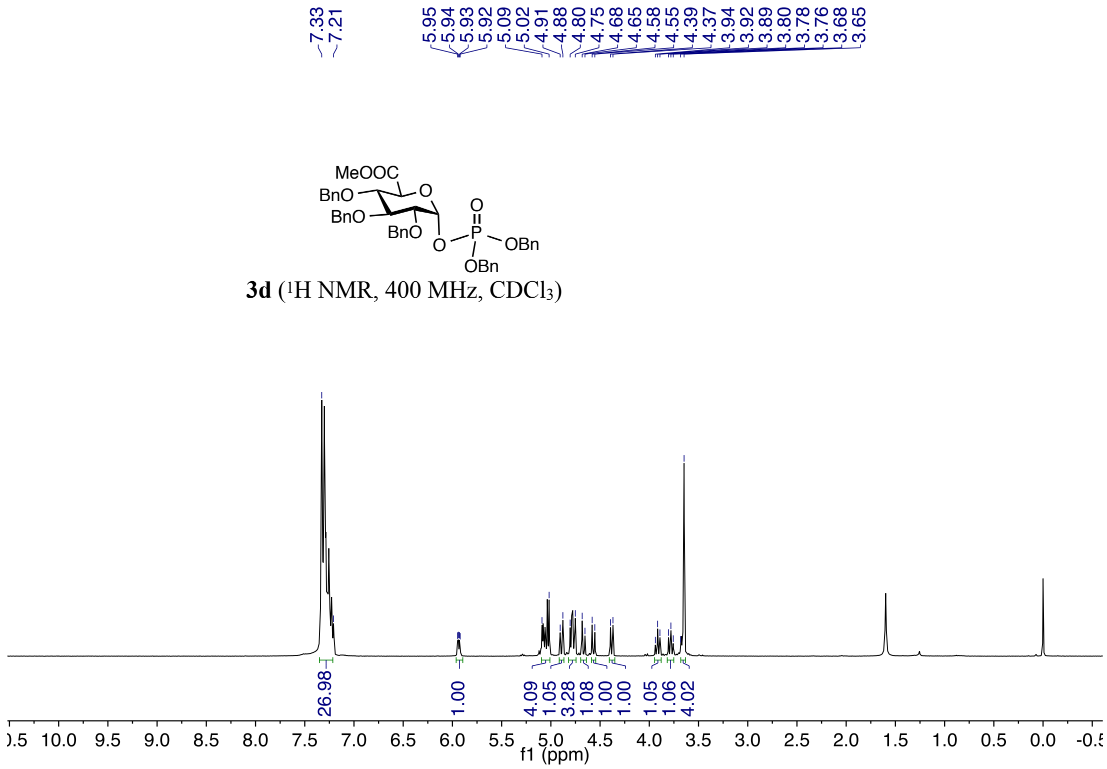

Supplementary Figure 13. ^{13}C NMR spectrum of compound **3**d

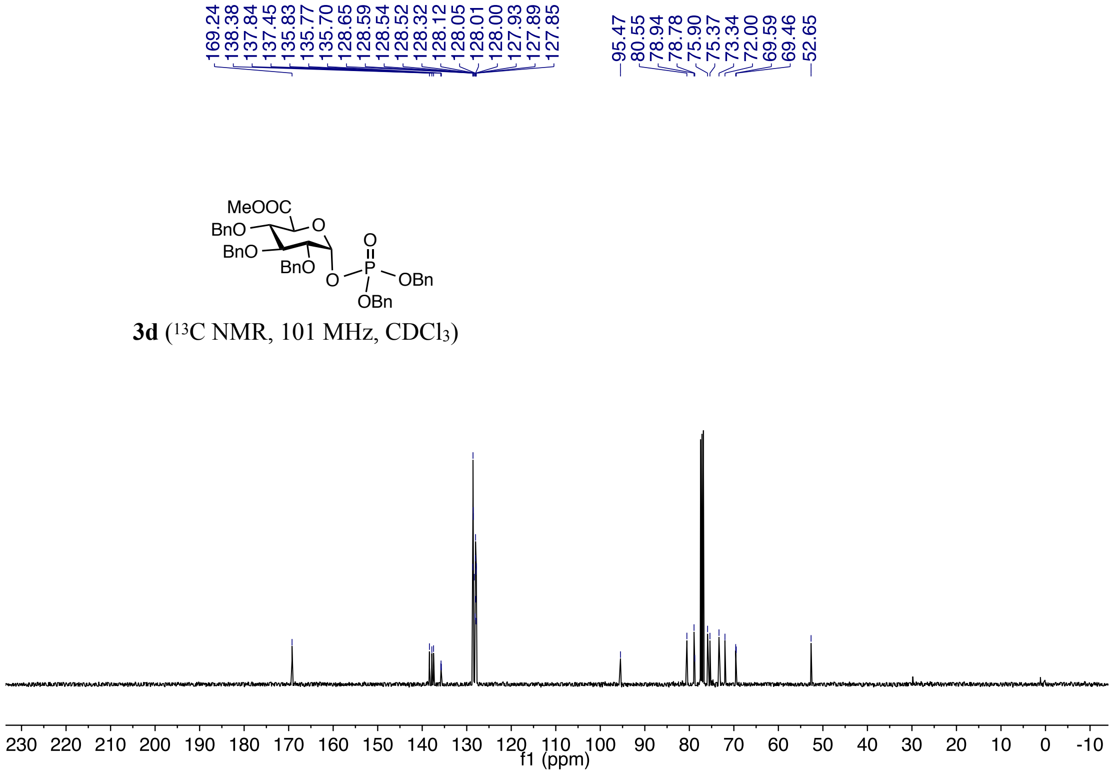

Supplementary Figure 14. ^{31}P NMR spectrum of compound **3d**

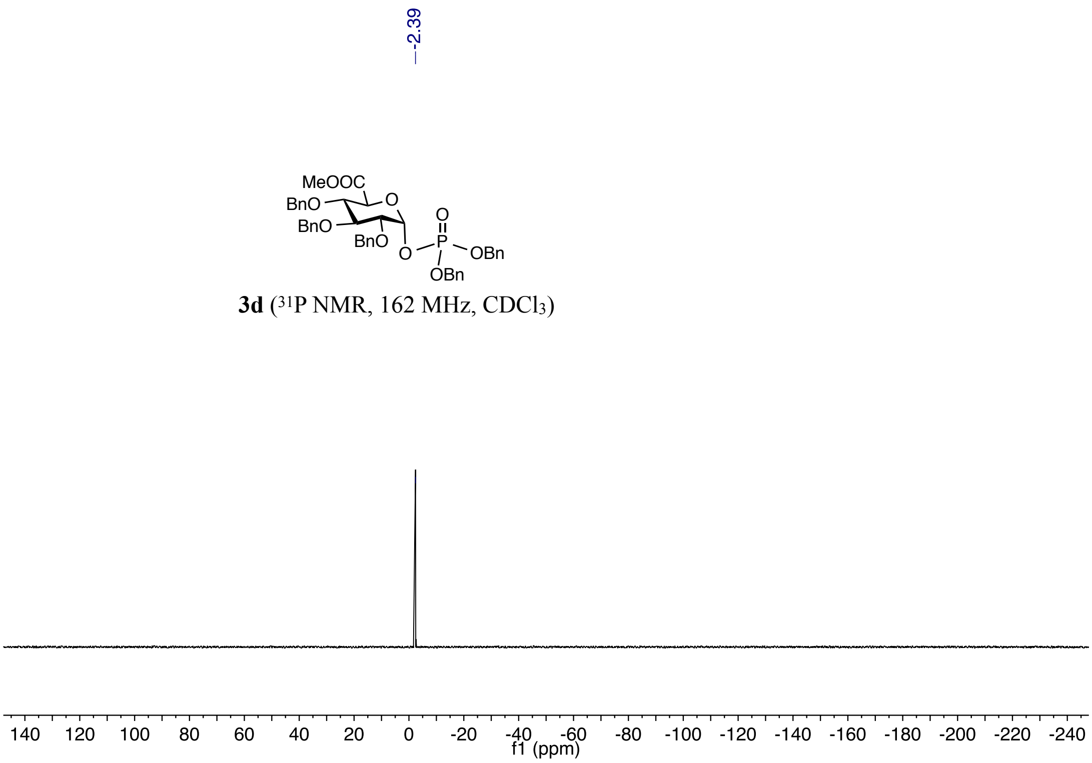

Supplementary Figure 15. ^{1}H NMR spectrum of compound **3eα**

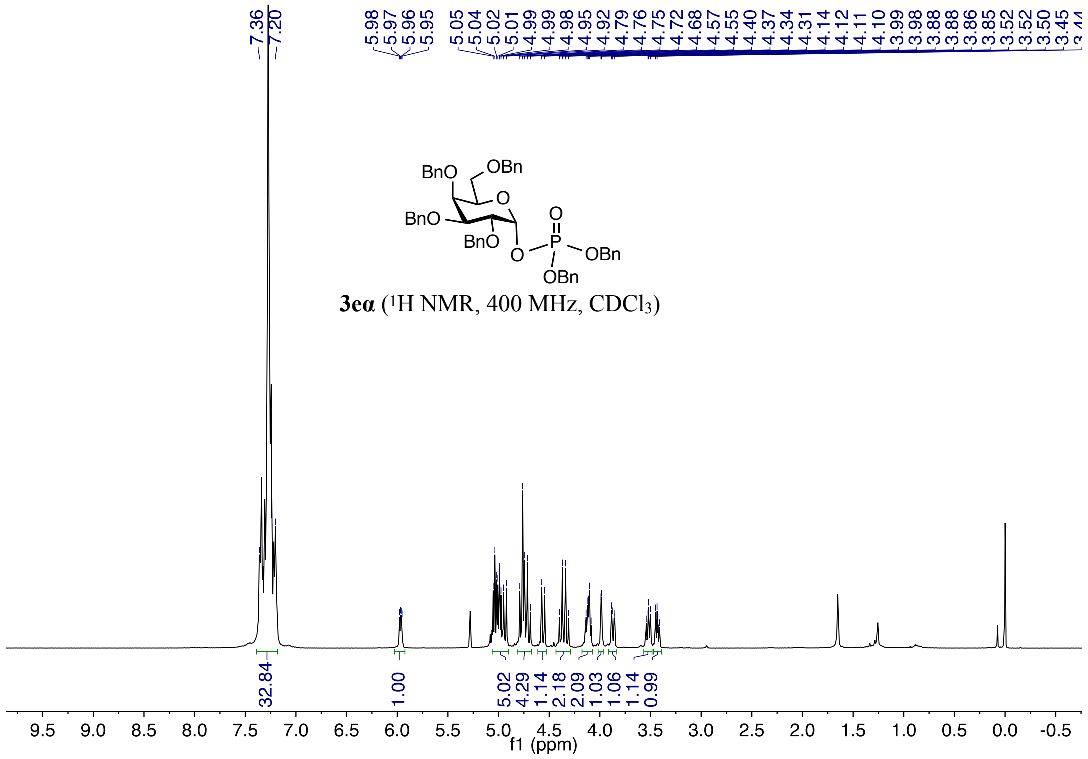

Supplementary Figure 16. ^{13}C NMR spectrum of compound **3eα**

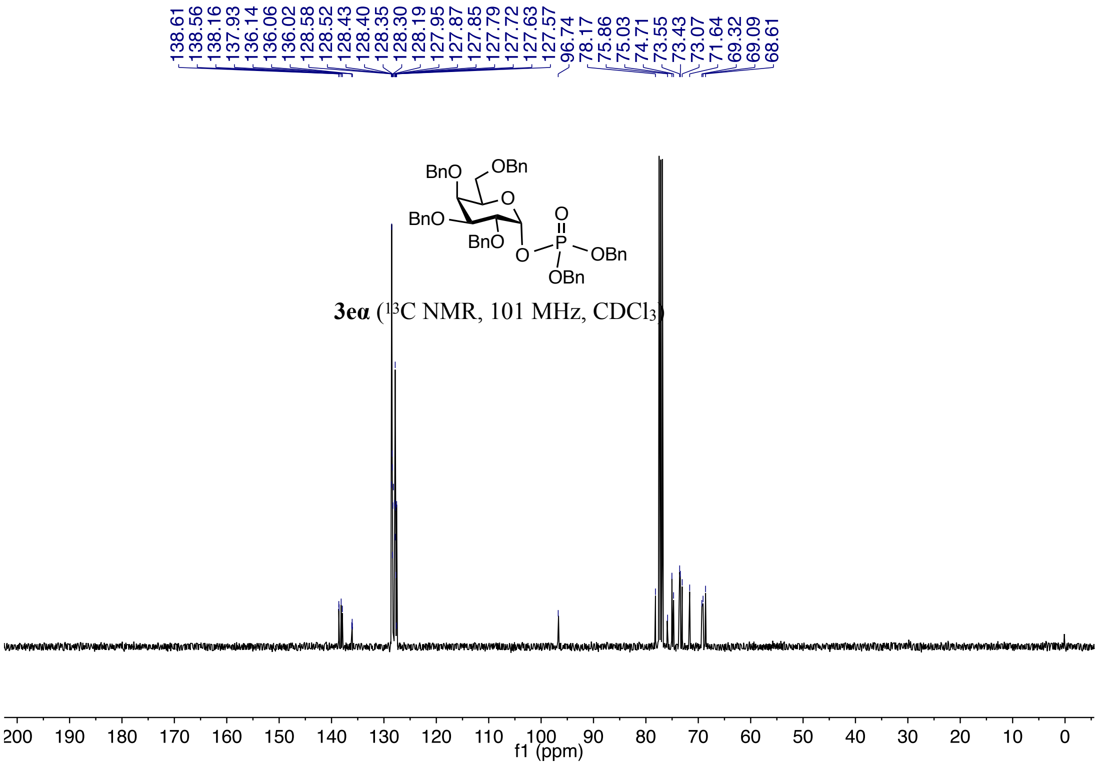

Supplementary Figure 17. ^{31}P NMR spectrum of compound **3eα**

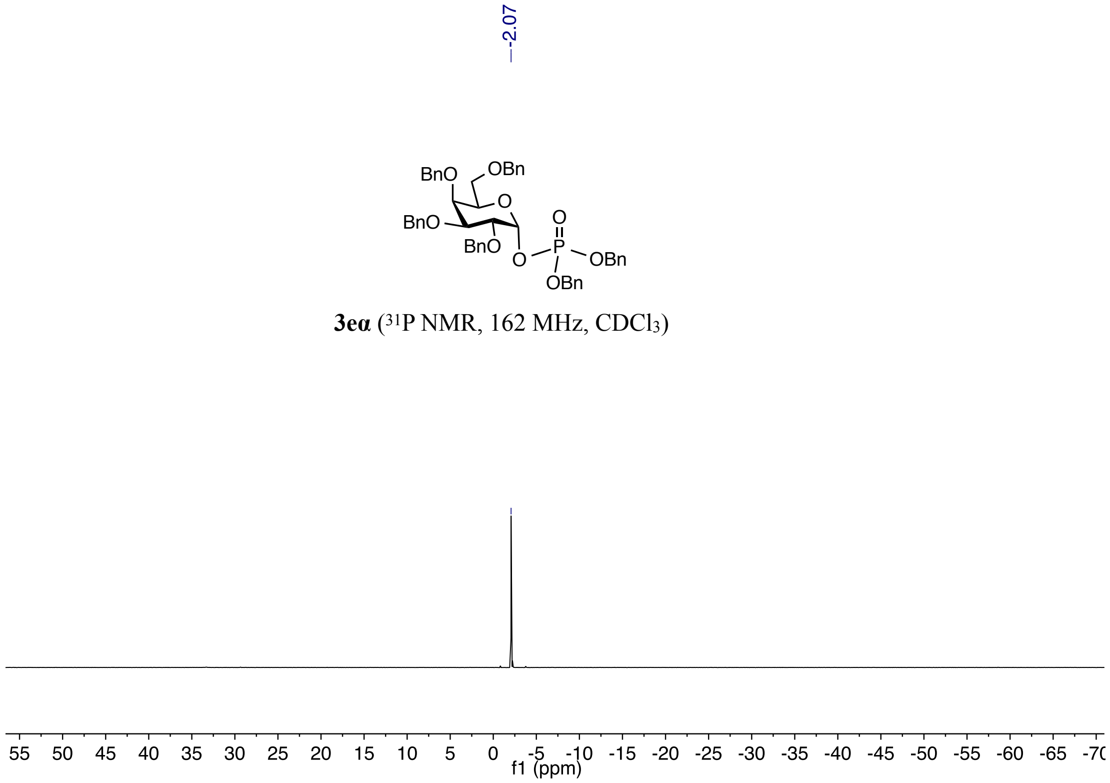

Supplementary Figure 18. ^{1}H NMR spectrum of compound **3eβ**

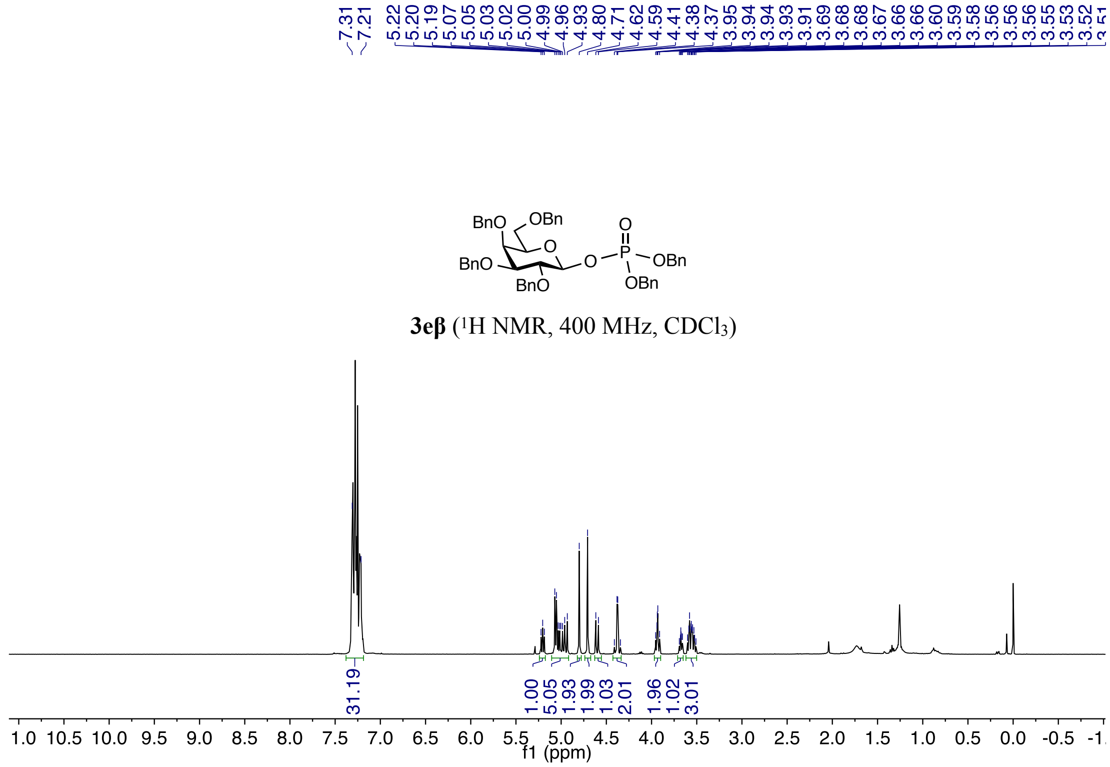

Supplementary Figure 19. ^{13}C NMR spectrum of compound **3eβ**

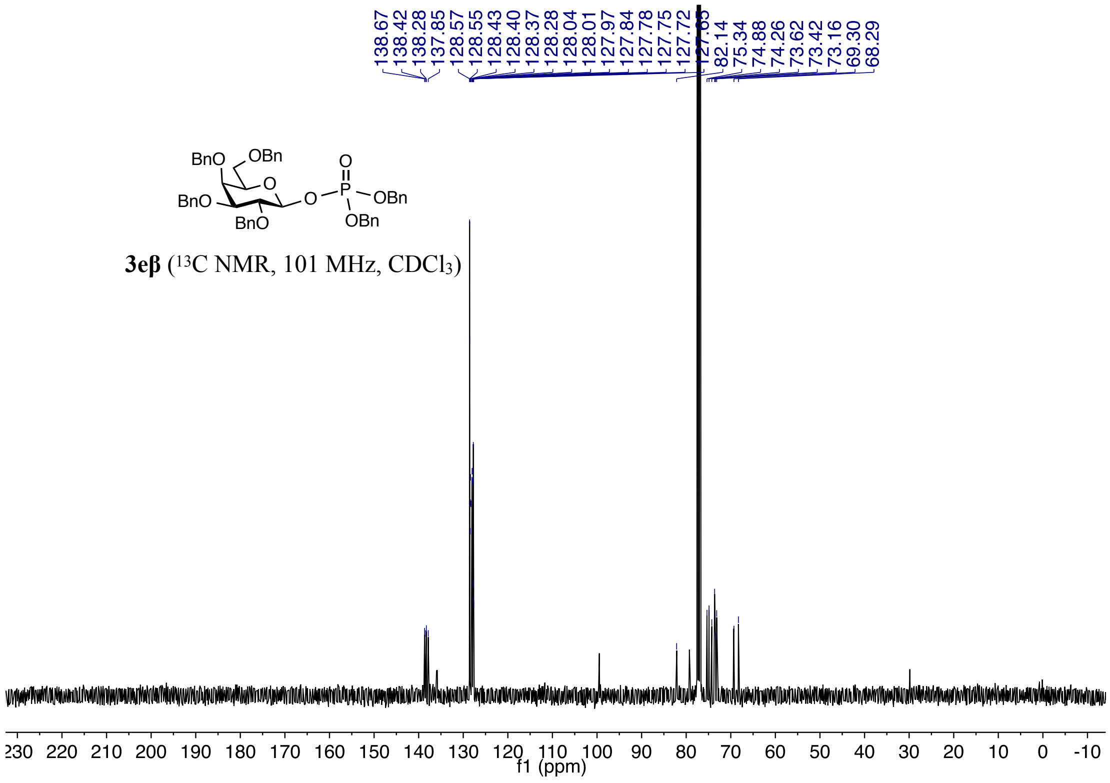

Supplementary Figure 20. ^{31}P NMR spectrum of compound **3eβ**

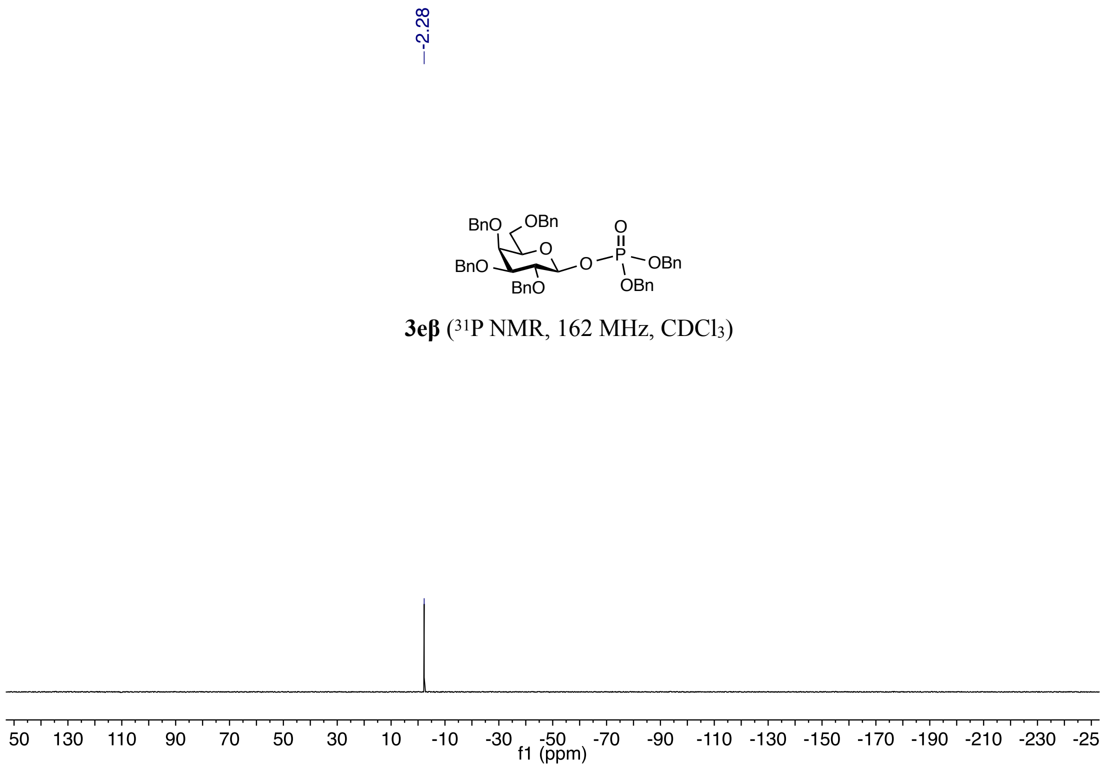

Supplementary Figure 21. ^{1}H NMR spectrum of compound **3e'**

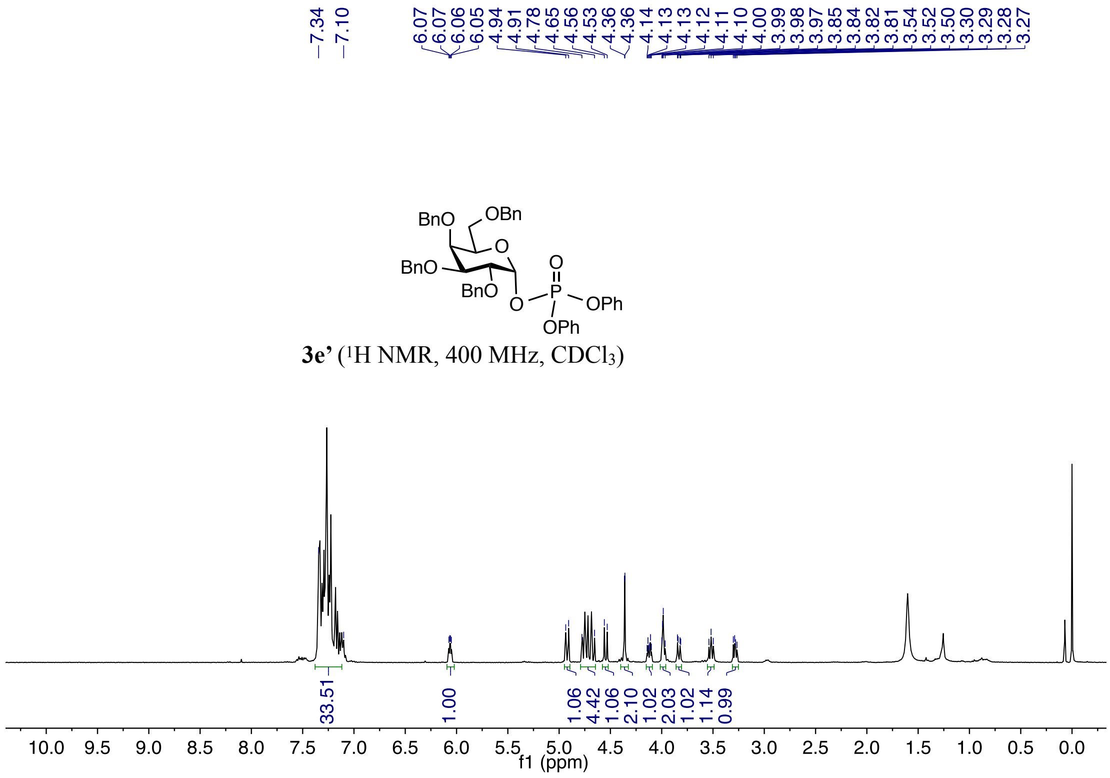

Supplementary Figure 22. ^{13}C NMR spectrum of compound **3e'**

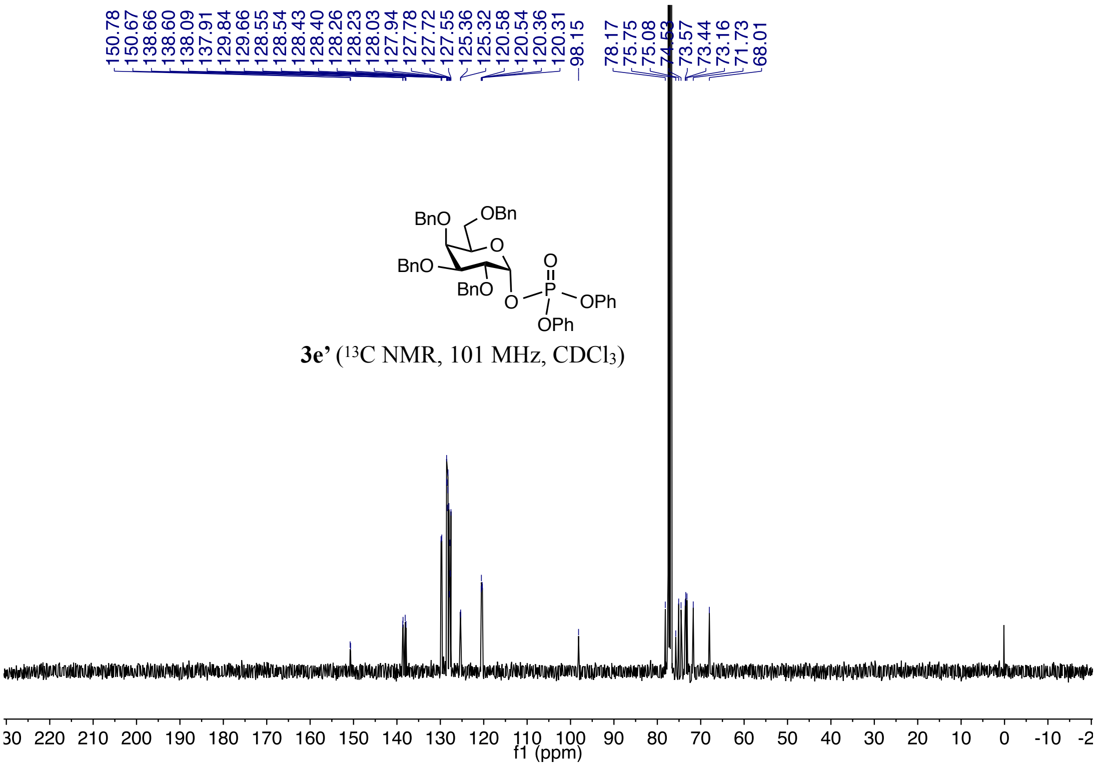

Supplementary Figure 23. ^{31}P NMR spectrum of compound **3e’**

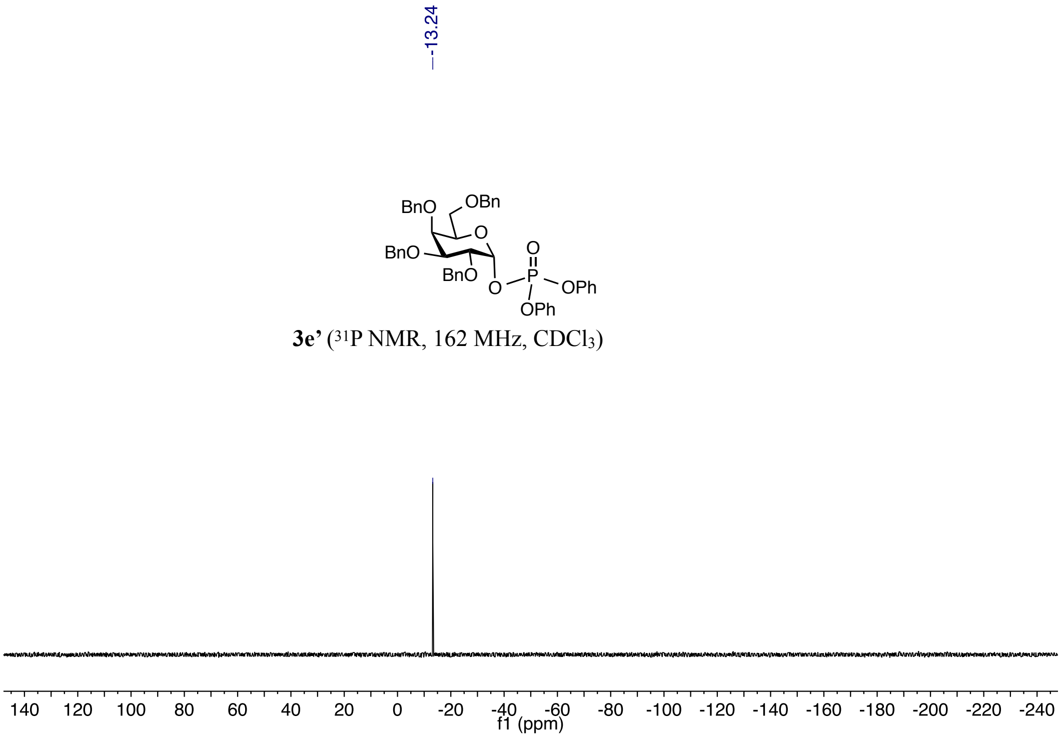

Supplementary Figure 24. ^{1}H NMR spectrum of compound **3f**

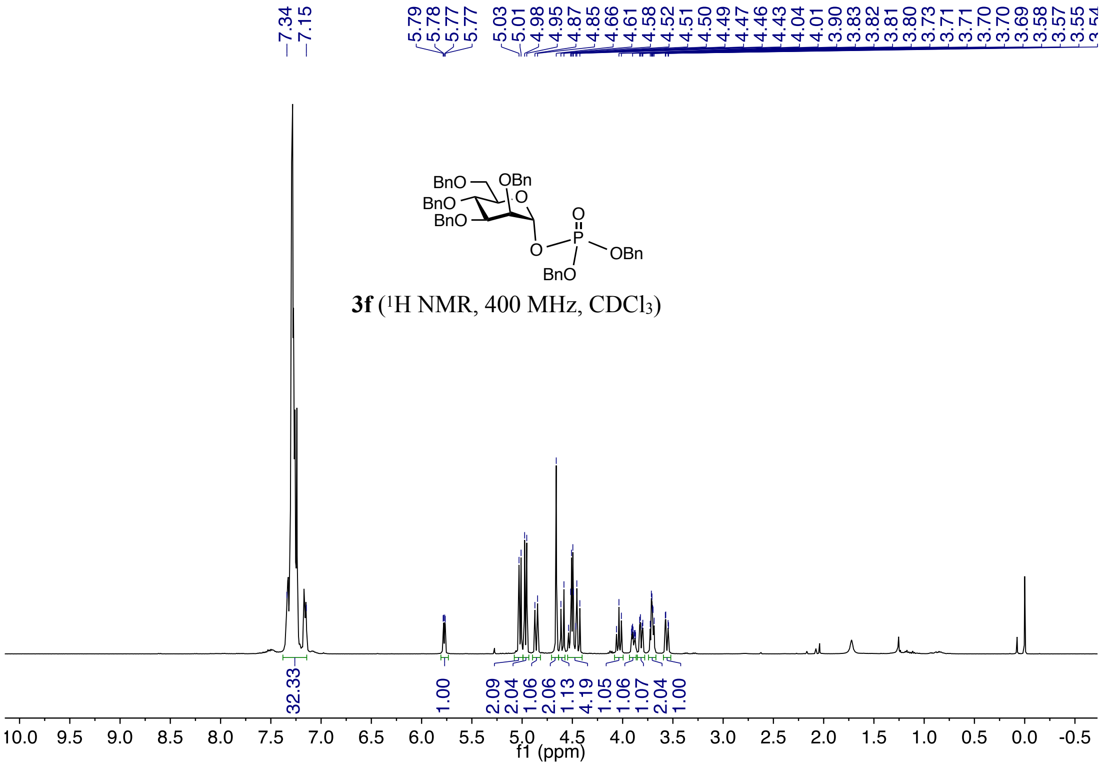

Supplementary Figure 25. ^{13}C NMR spectrum of compound **3f**

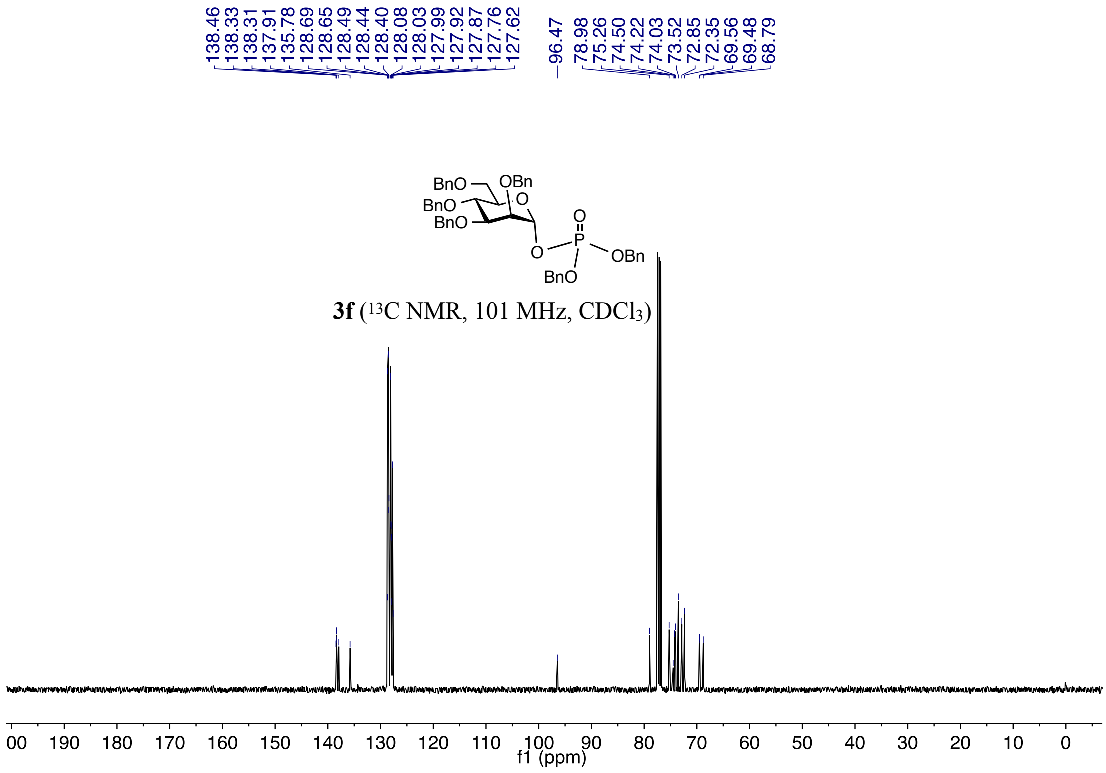

Supplementary Figure 26. ^{31}P NMR spectrum of compound **3f**

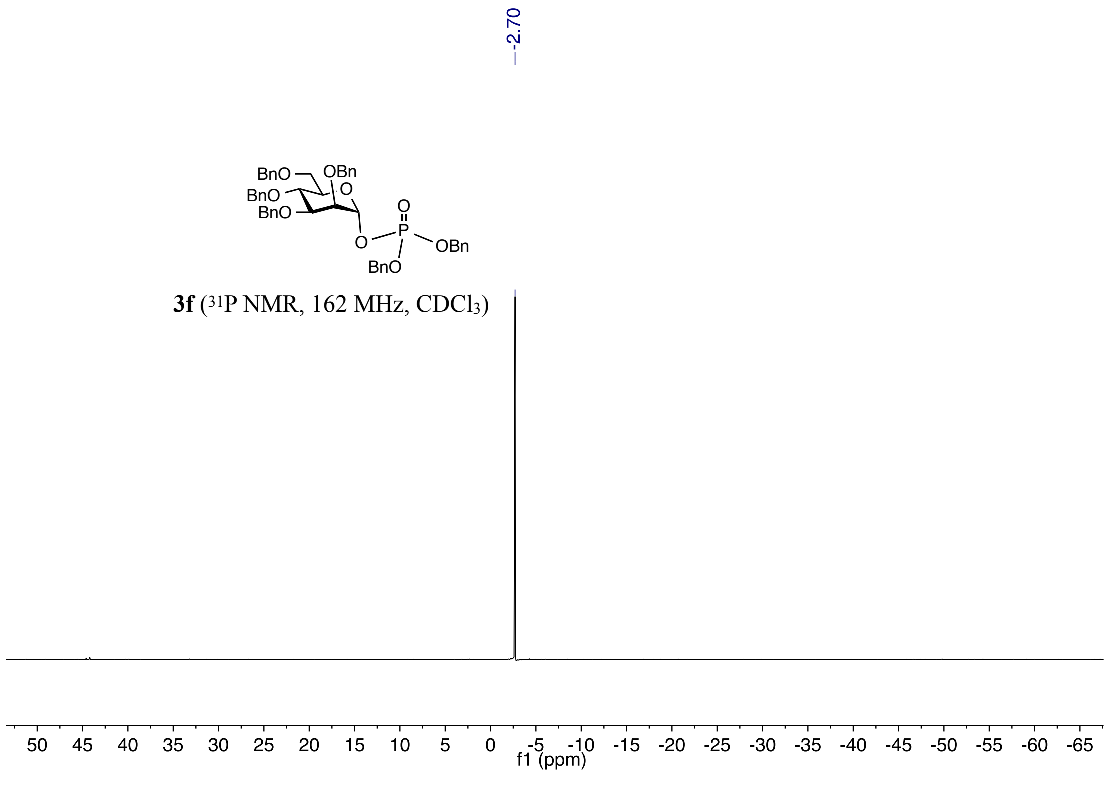

Supplementary Figure 27. ^{1}H NMR spectrum of compound **3f’**

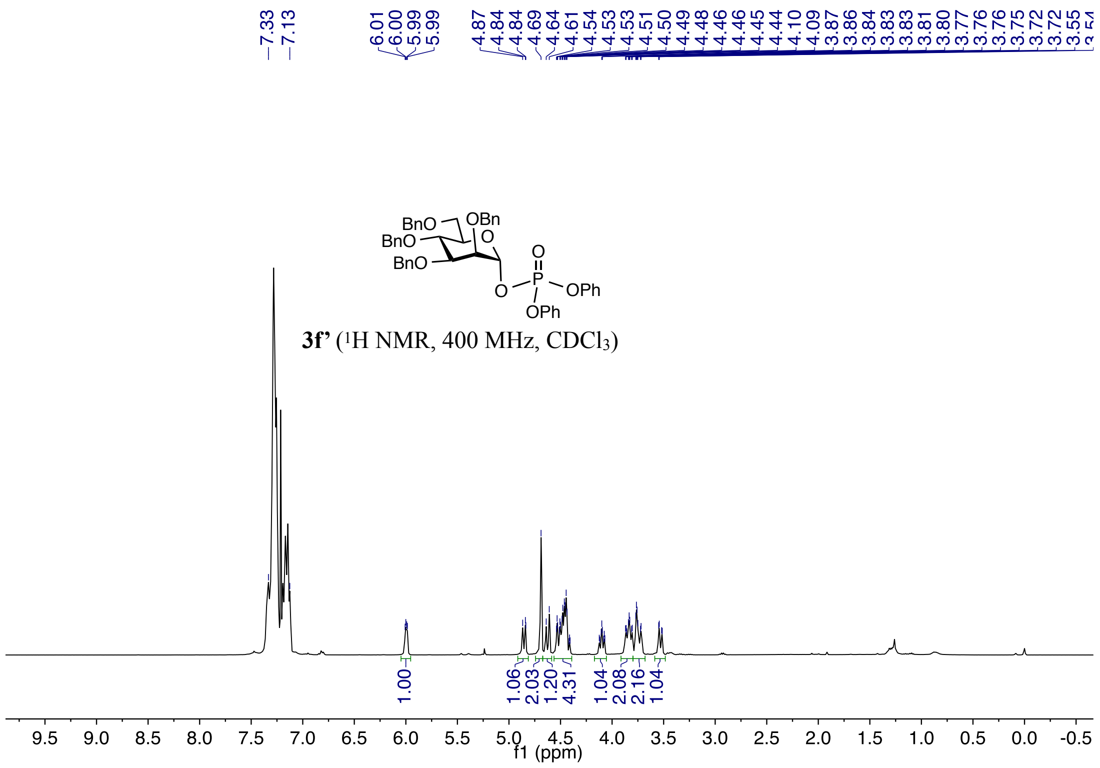

Supplementary Figure 28. ^{13}C NMR spectrum of compound **3ef’**

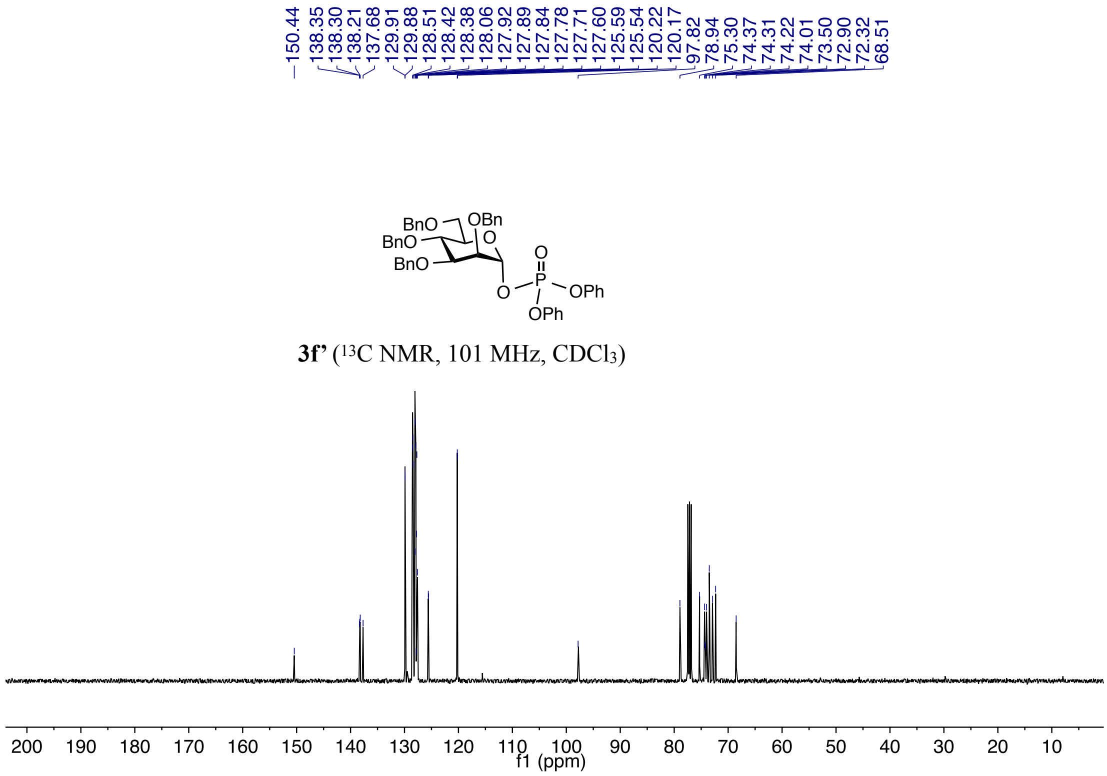

Supplementary Figure 29. ^{31}P NMR spectrum of compound **3f’**

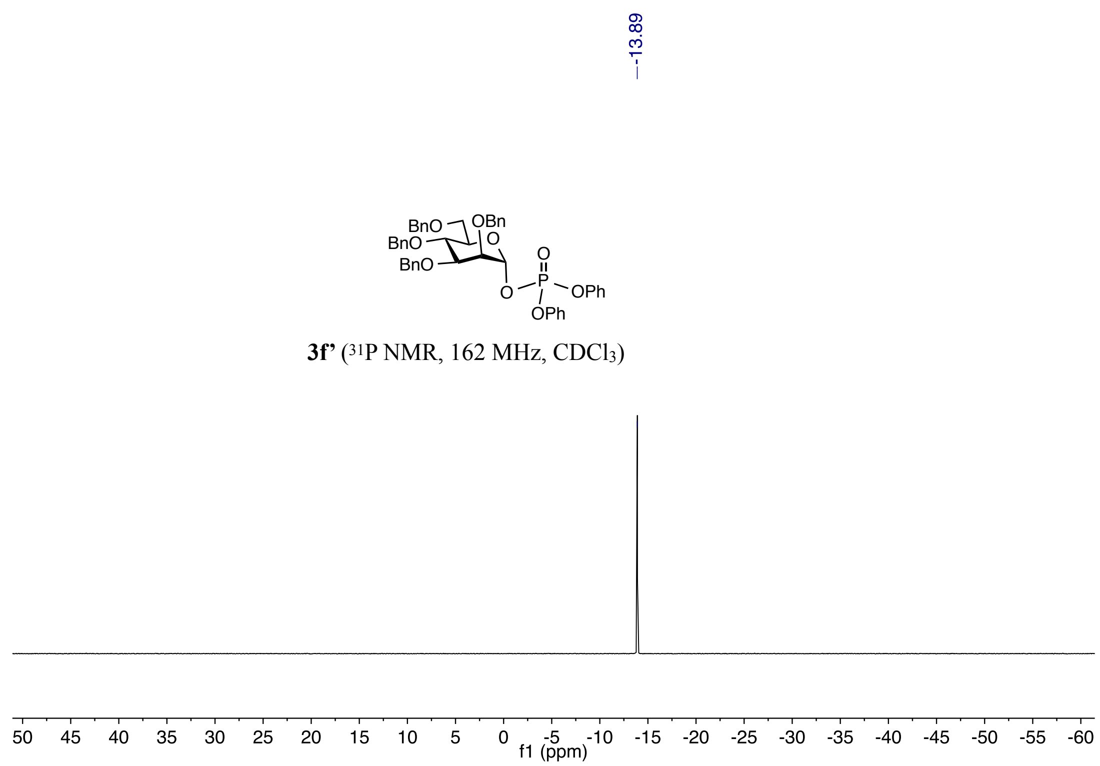

Supplementary Figure 30. ^{1}H NMR spectrum of compound **3g**

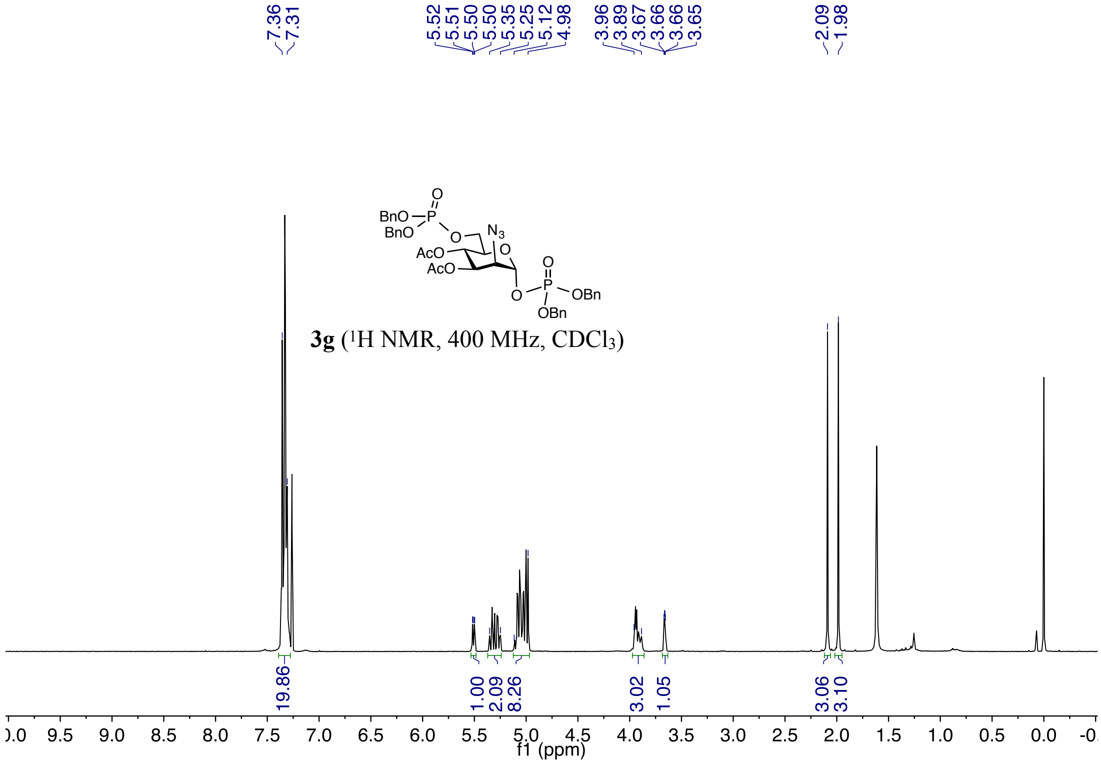

Supplementary Figure 31. ^{13}C NMR spectrum of compound **3g**

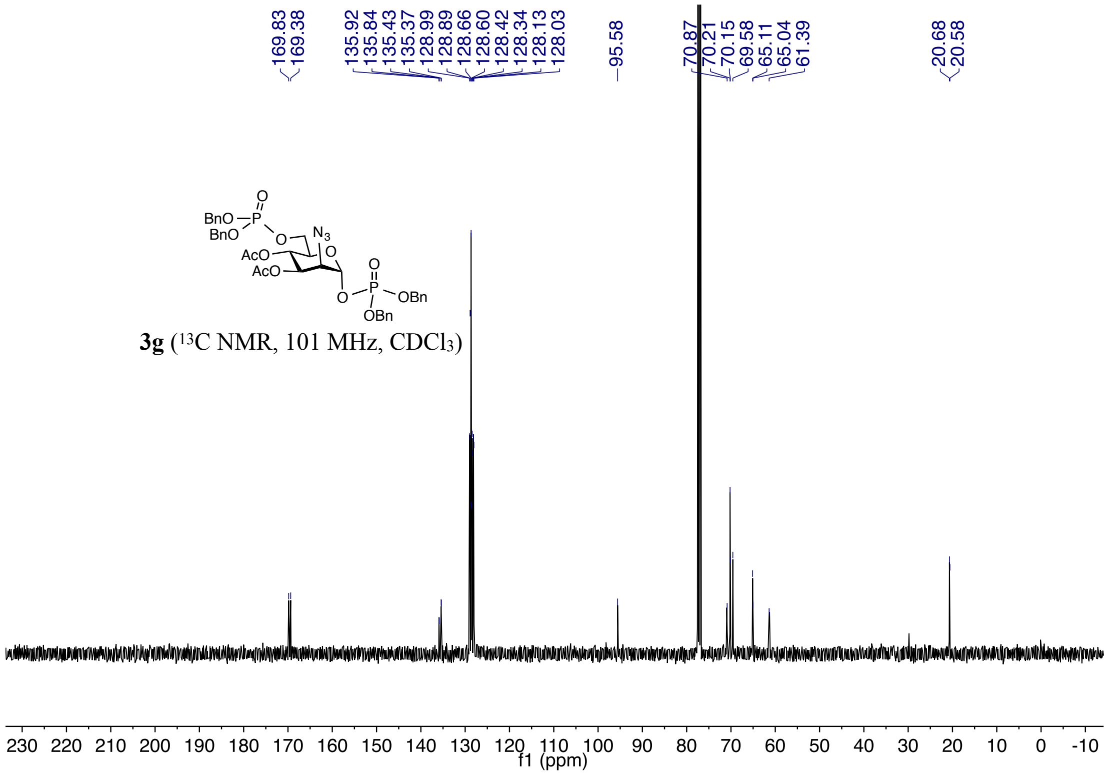

Supplementary Figure 32. ^{31}P NMR spectrum of compound **3g**

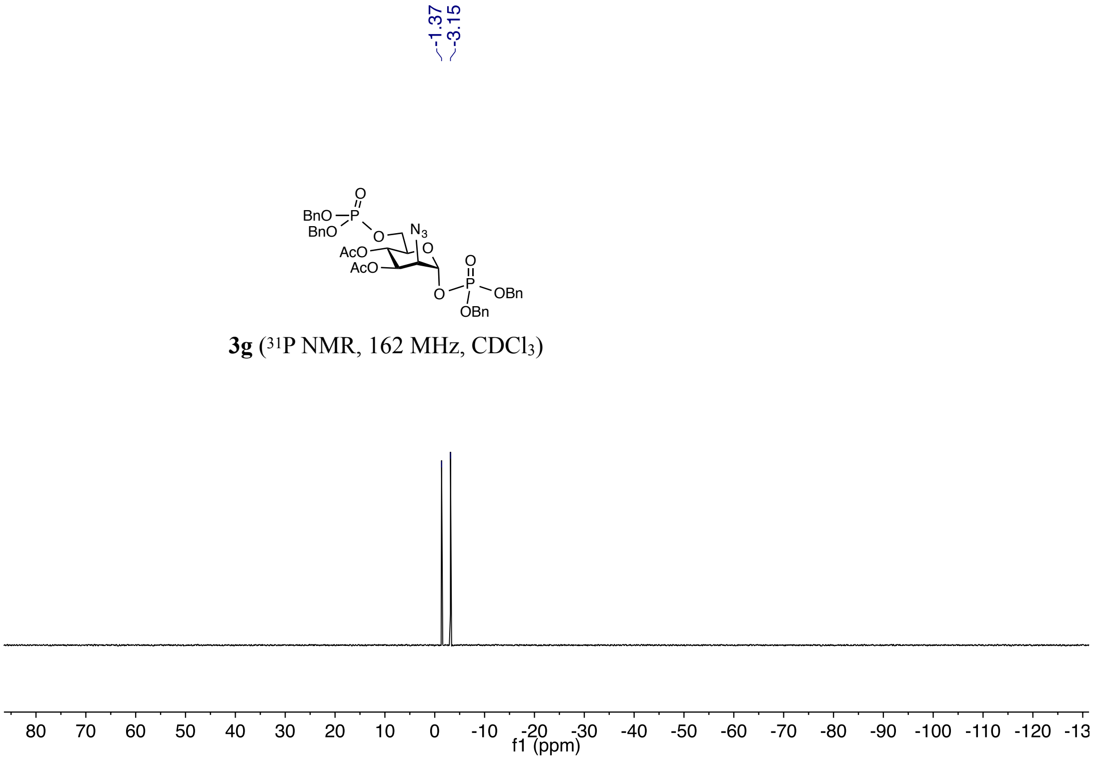

Supplementary Figure 33. ^{1}H NMR spectrum of compound **3h**

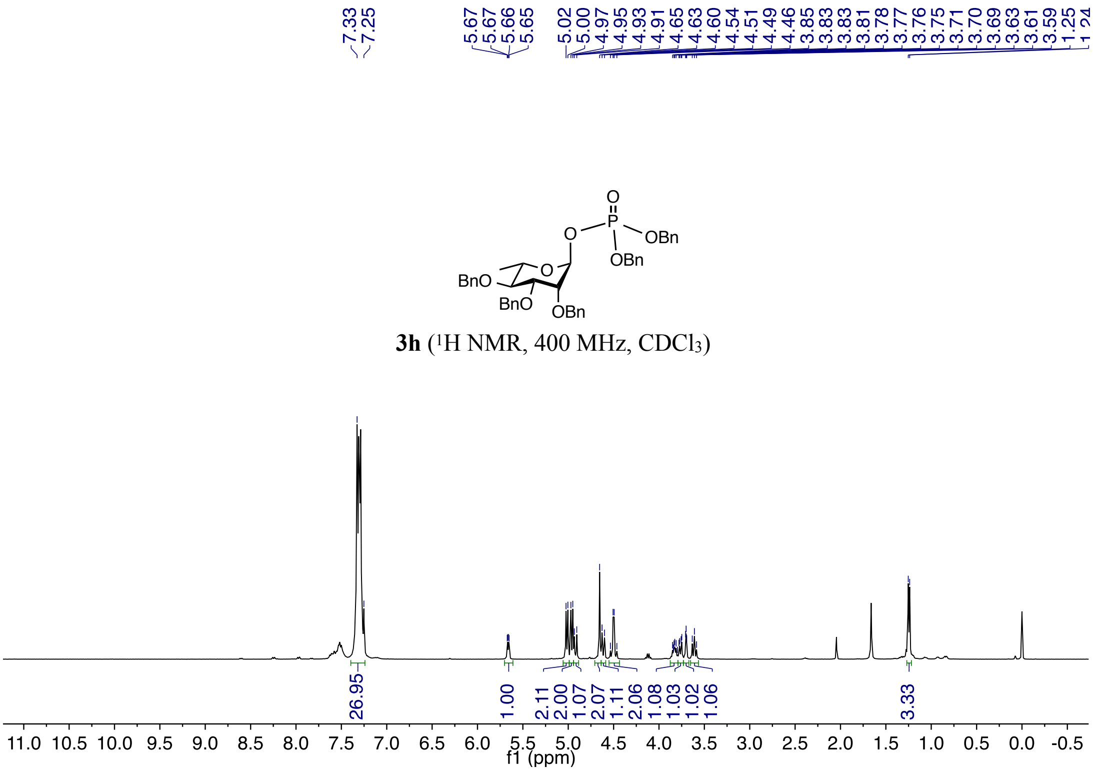

Supplementary Figure 34. ^{13}C NMR spectrum of compound **3h**

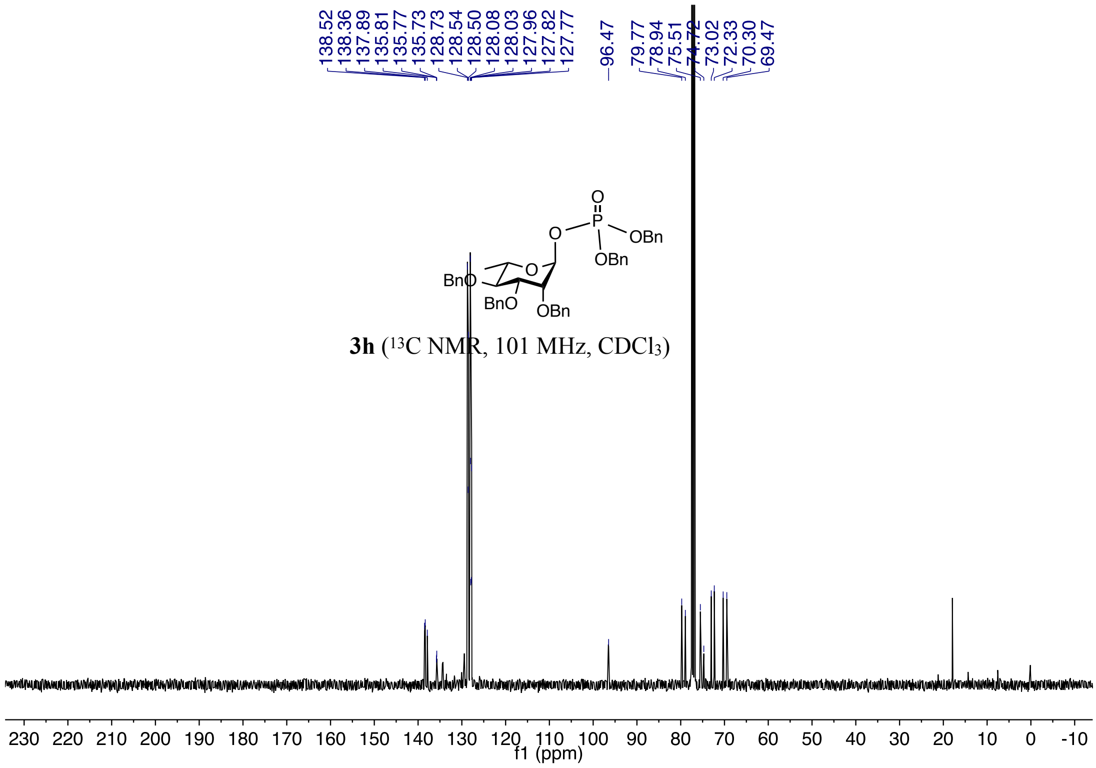

Supplementary Figure 35. ^{31}P NMR spectrum of compound **3h**

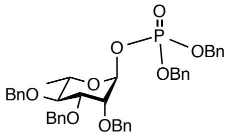

3h (^{31}P NMR, 162 MHz, $CDCl_3$)

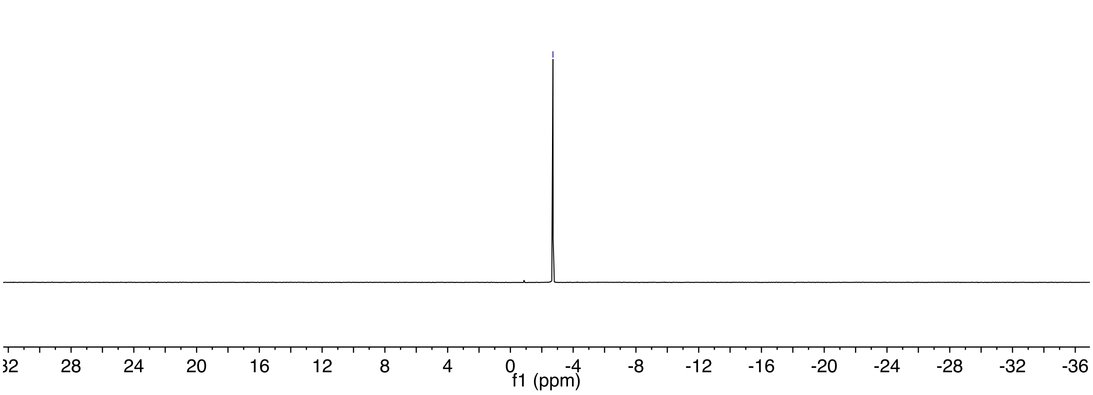

Supplementary Figure 36. ^{1}H NMR spectrum of compound **3iα**

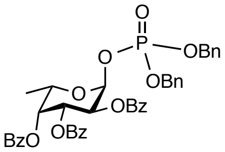

3iα (^{1}H NMR, 400 MHz, $CDCl_3$)

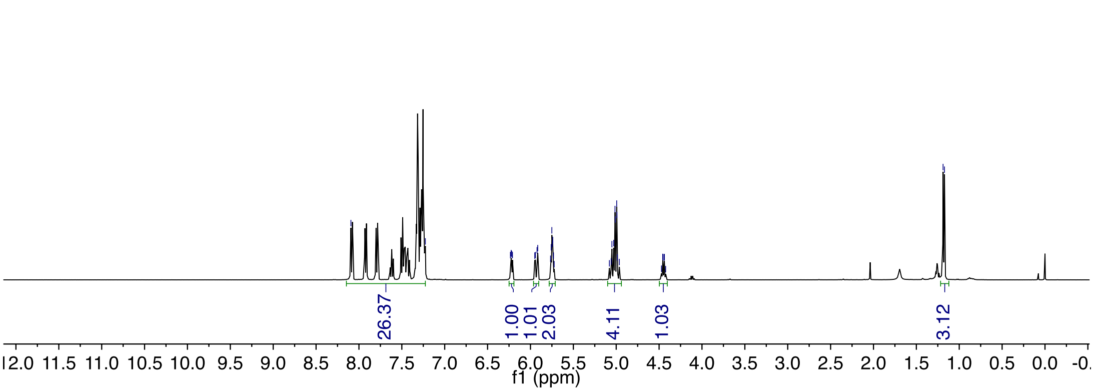

Supplementary Figure 37. ^{13}C NMR spectrum of compound **3iα**

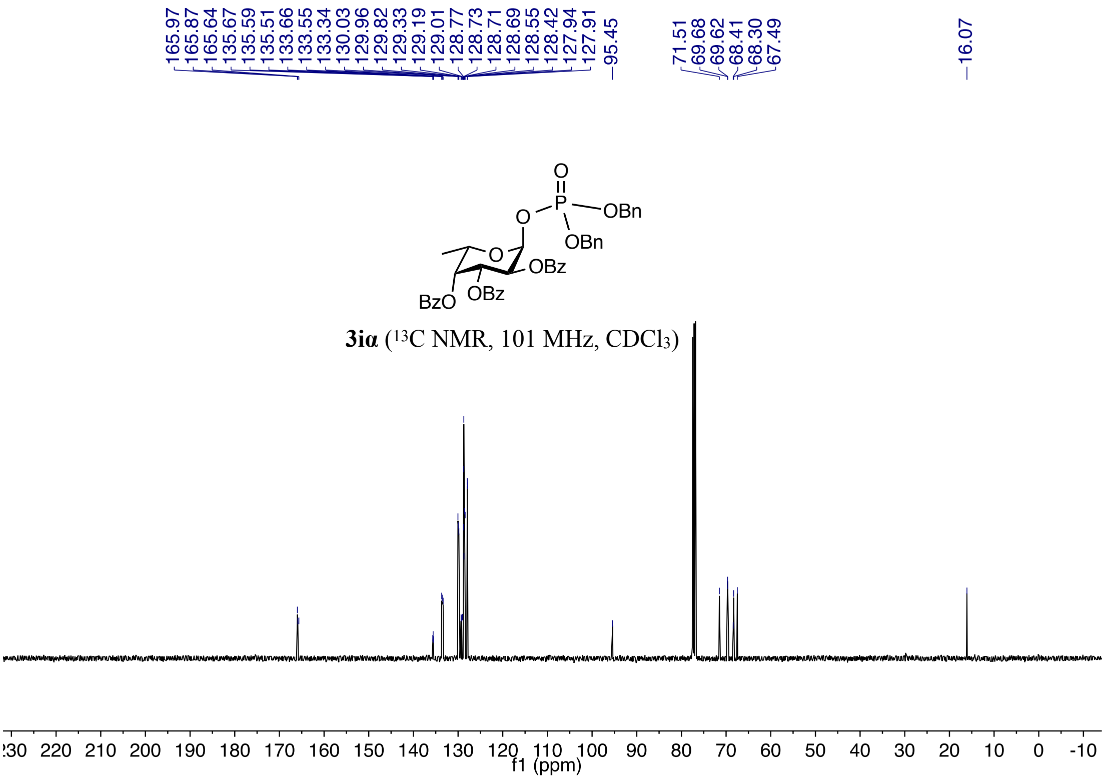

Supplementary Figure 38. ^{31}P NMR spectrum of compound **3iα**

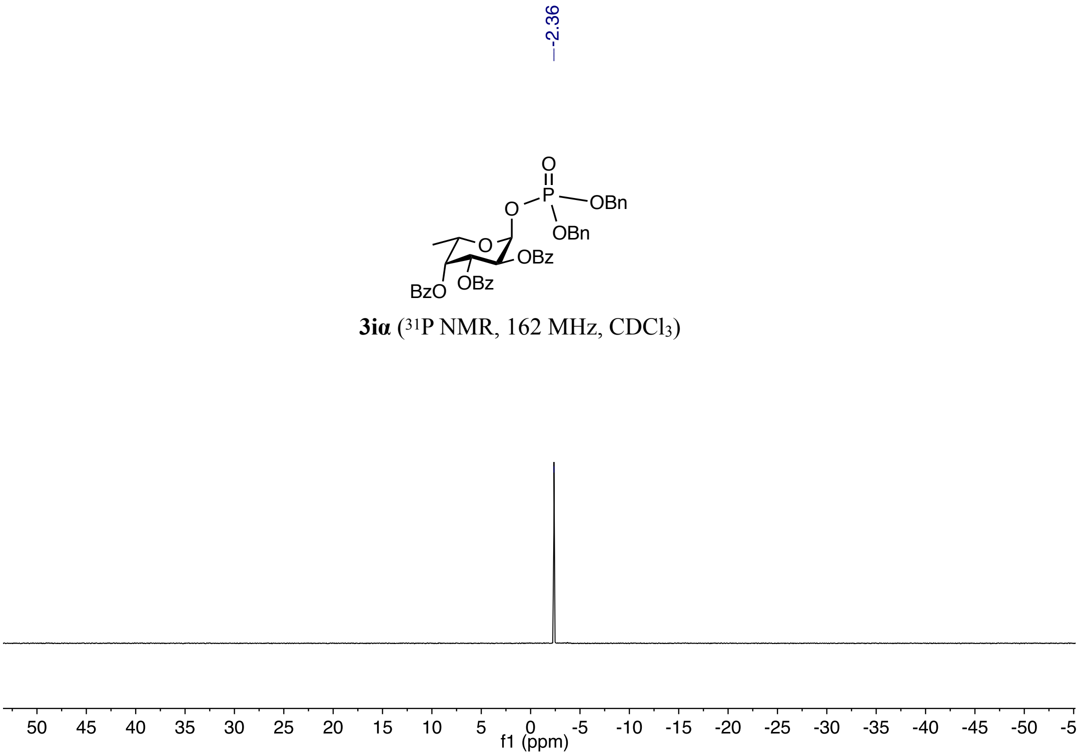

Supplementary Figure 39. ^{1}H NMR spectrum of compound **3iβ**

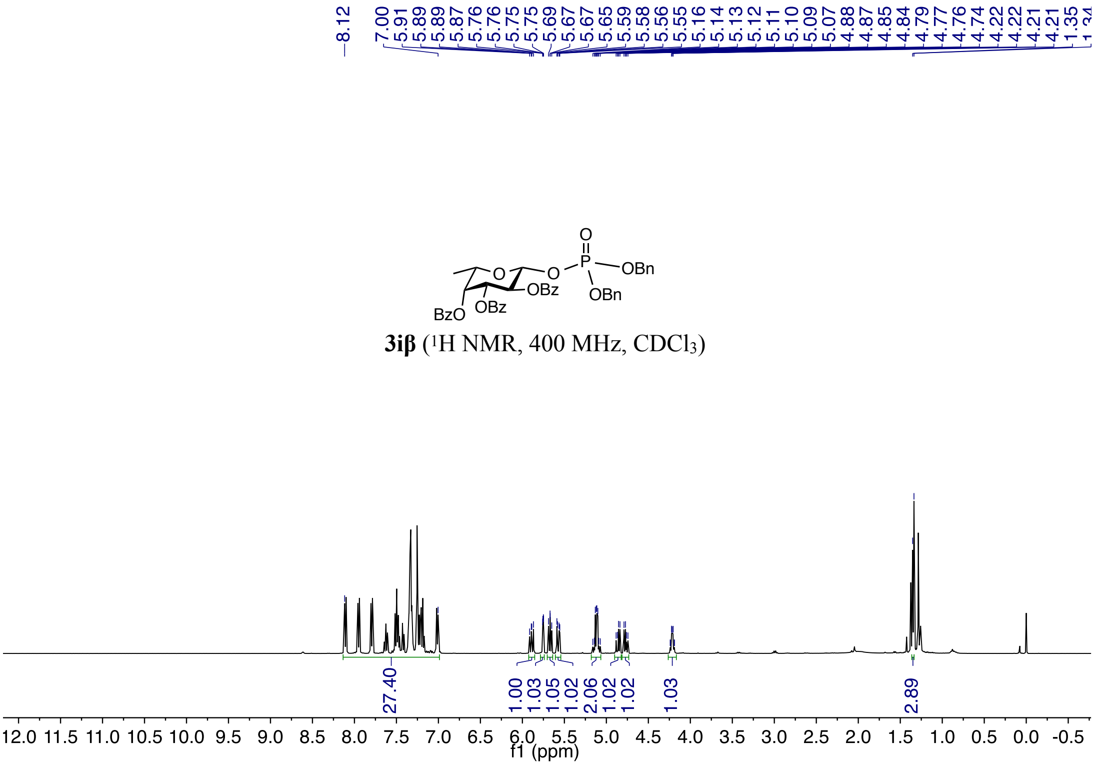

Supplementary Figure 40. ^{13}C NMR spectrum of compound **3iβ**

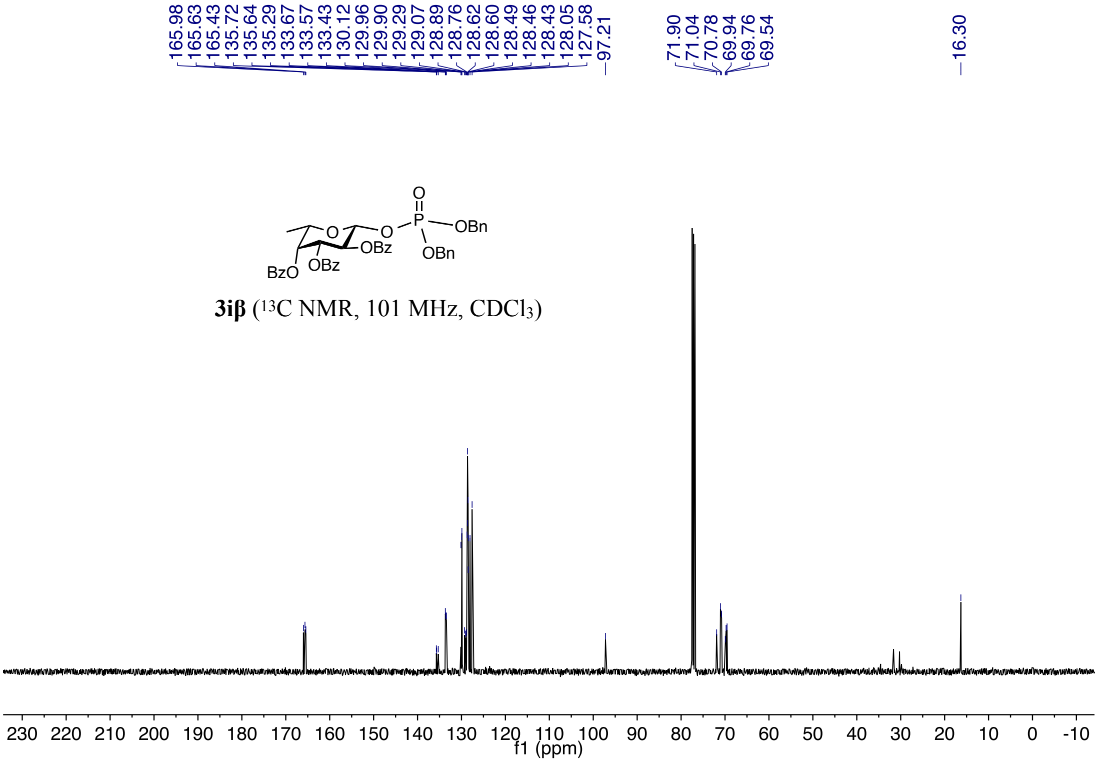

Supplementary Figure 41. ^{31}P NMR spectrum of compound **3iβ**

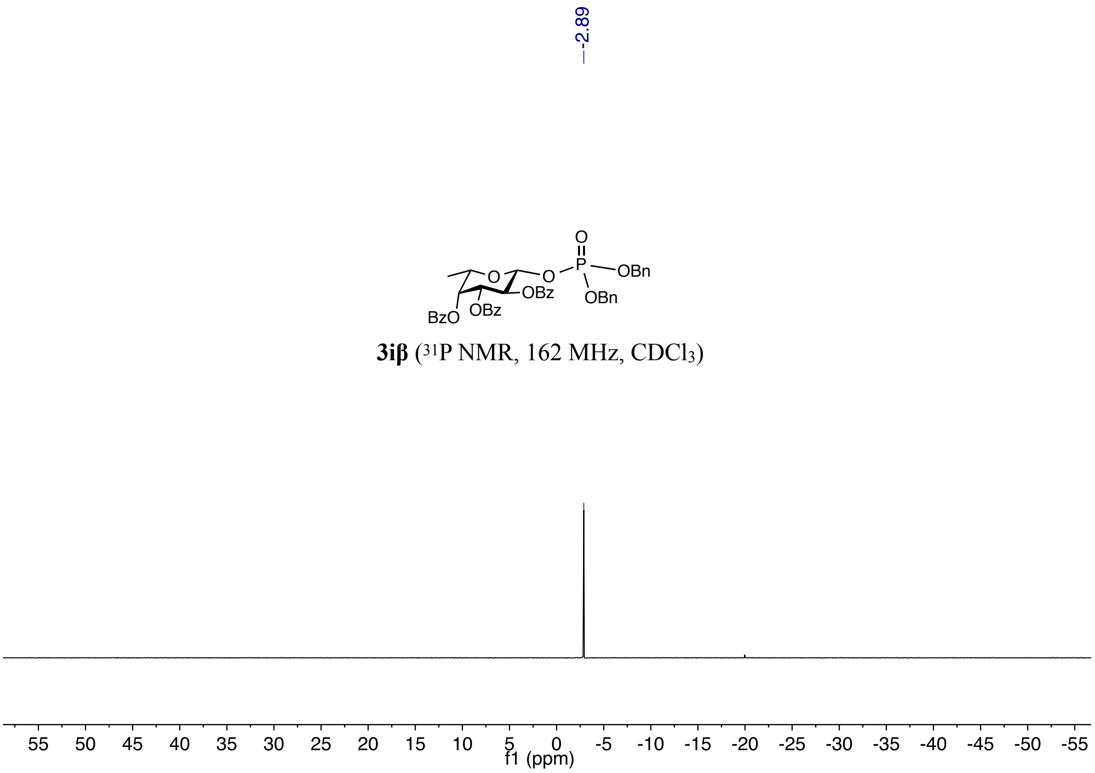

Supplementary Figure 42. ^{1}H NMR spectrum of compound **3j**

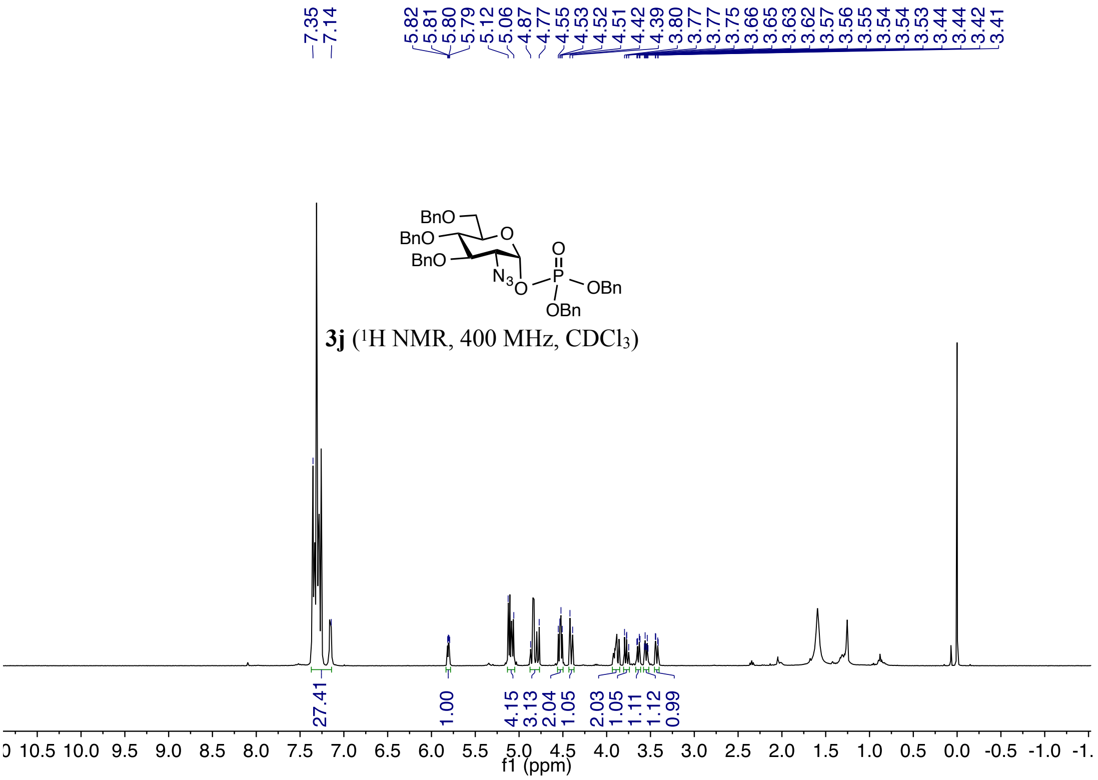

Supplementary Figure 43. ^{13}C NMR spectrum of compound **3j**

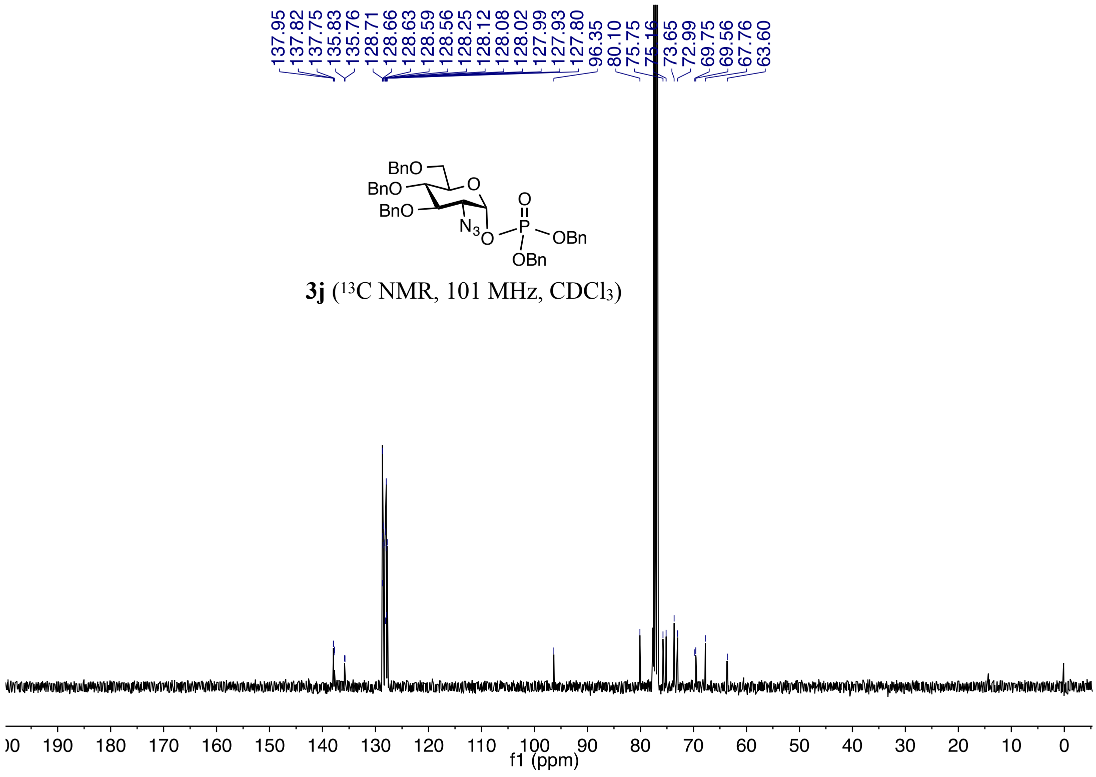

Supplementary Figure 44. ^{31}P NMR spectrum of compound **3j**

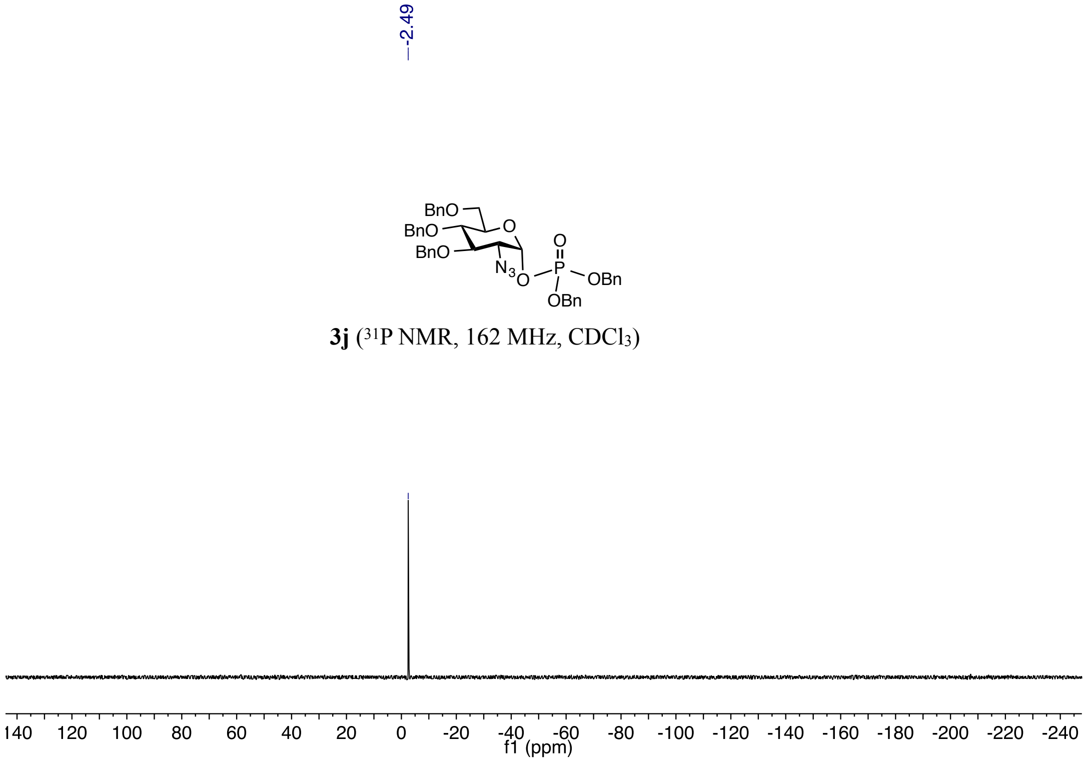

Supplementary Figure 45. ^{1}H NMR spectrum of compound **3k**

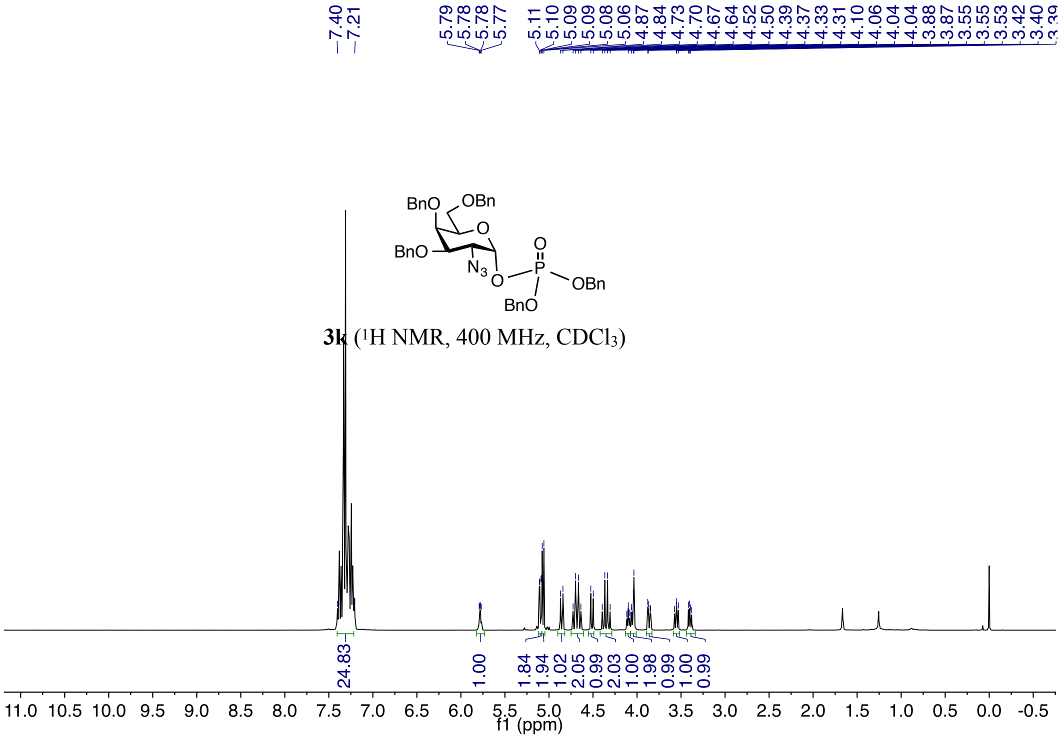

Supplementary Figure 46. ^{13}C NMR spectrum of compound **3k**

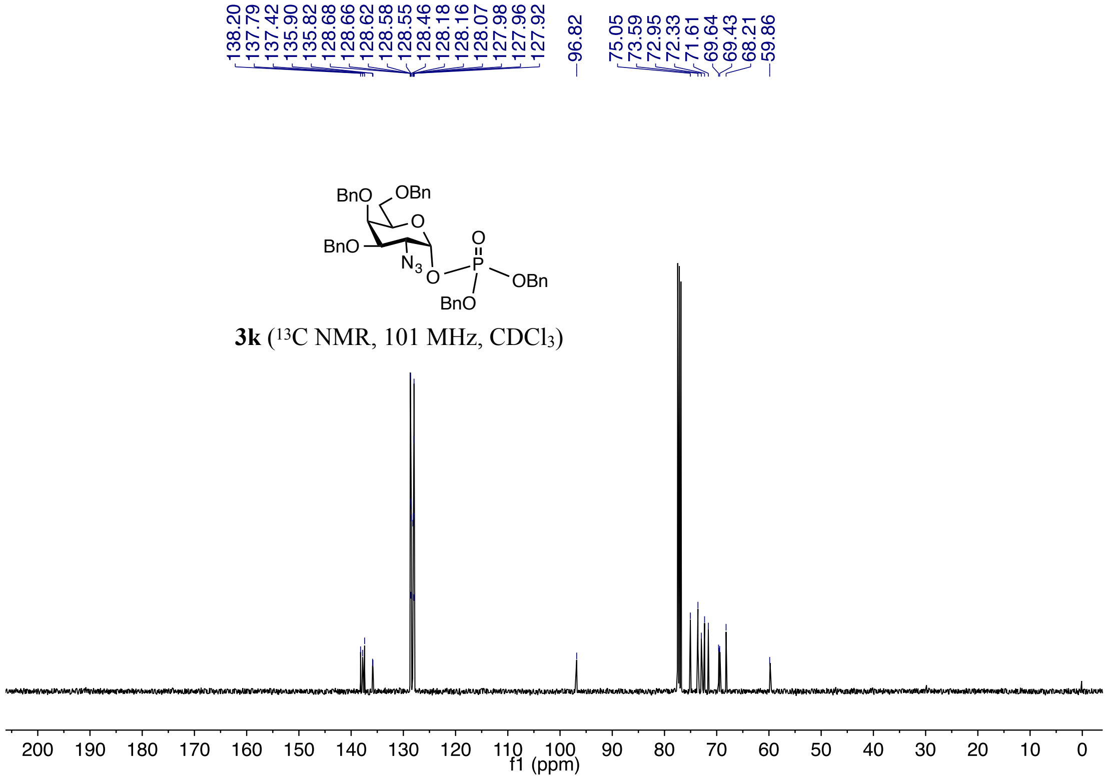

Supplementary Figure 47. ^{31}P NMR spectrum of compound **3k**

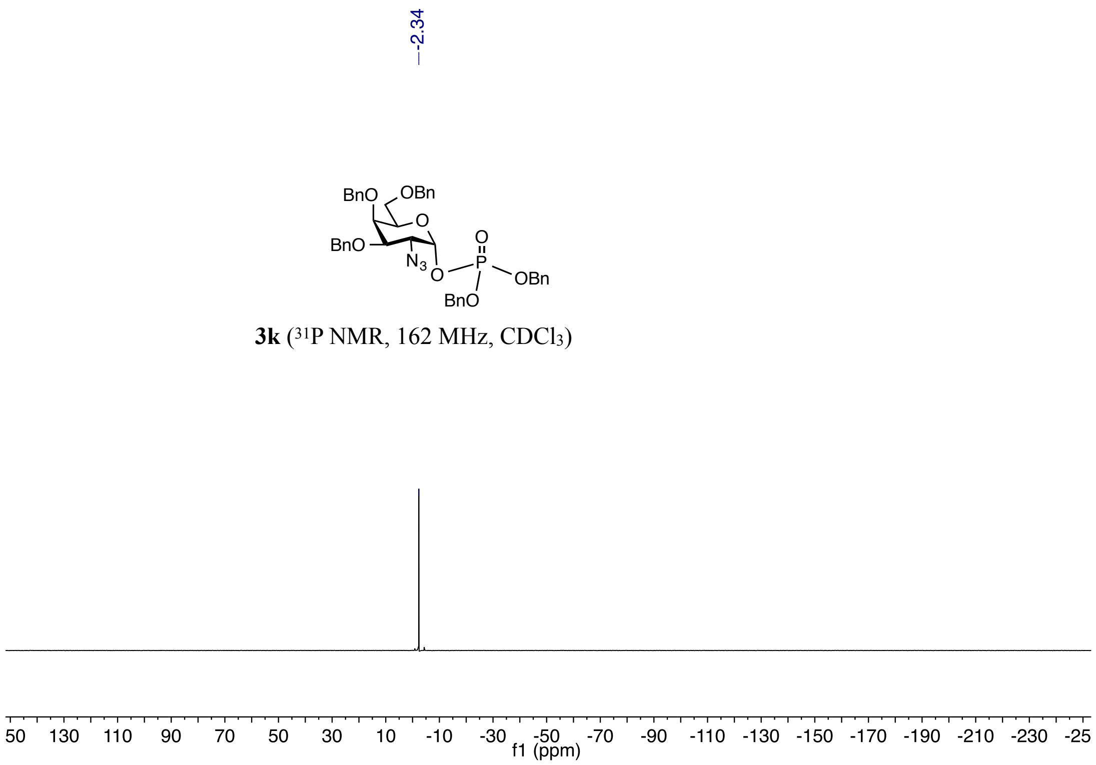

Supplementary Figure 48. ^{1}H NMR spectrum of compound **3l**

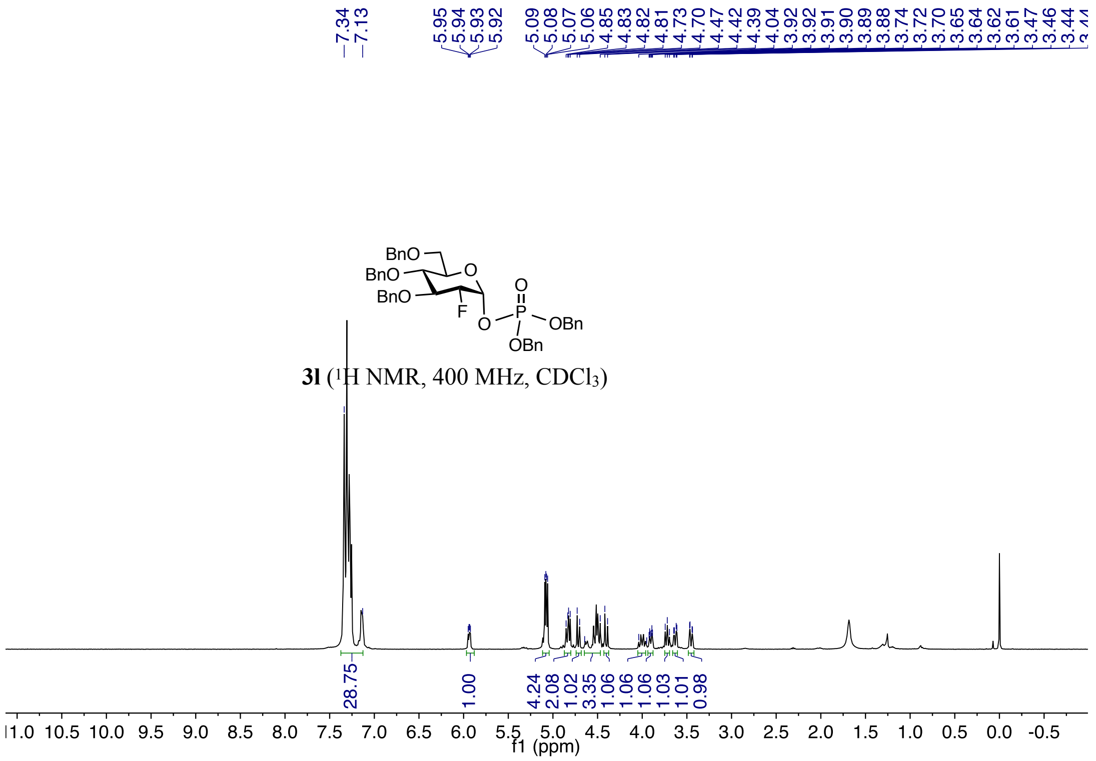

Supplementary Figure 49. ^{13}C NMR spectrum of compound **3l**

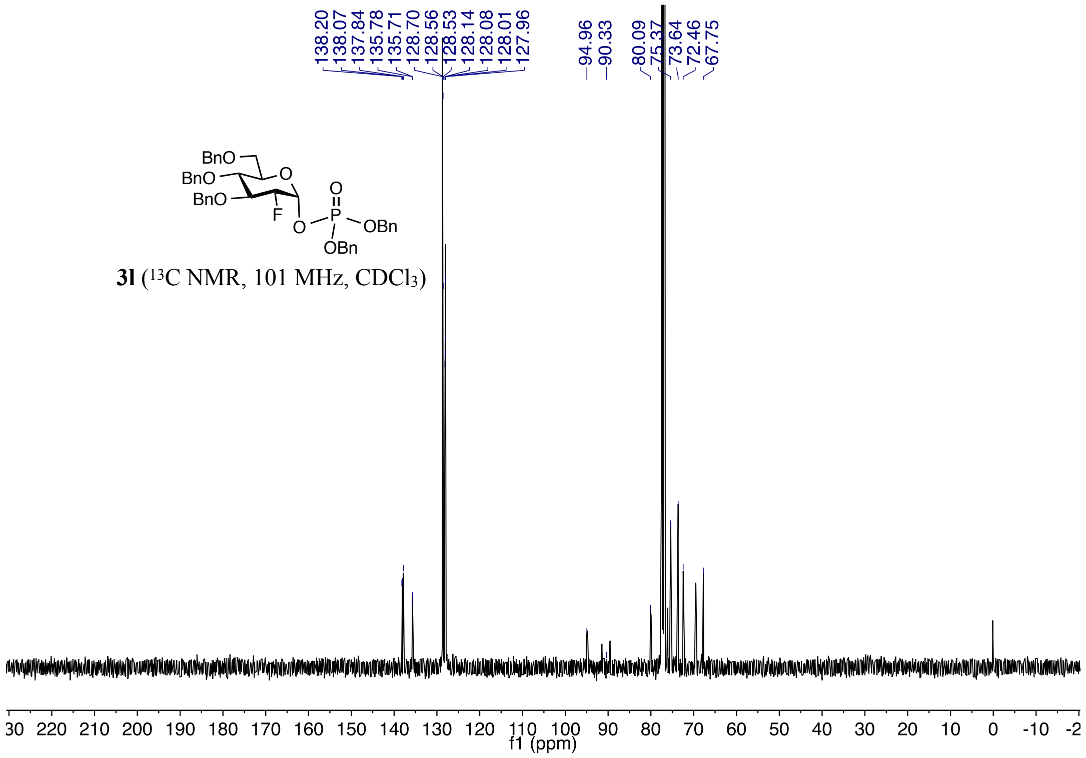

Supplementary Figure 50. ^{31}P NMR spectrum of compound **3l**

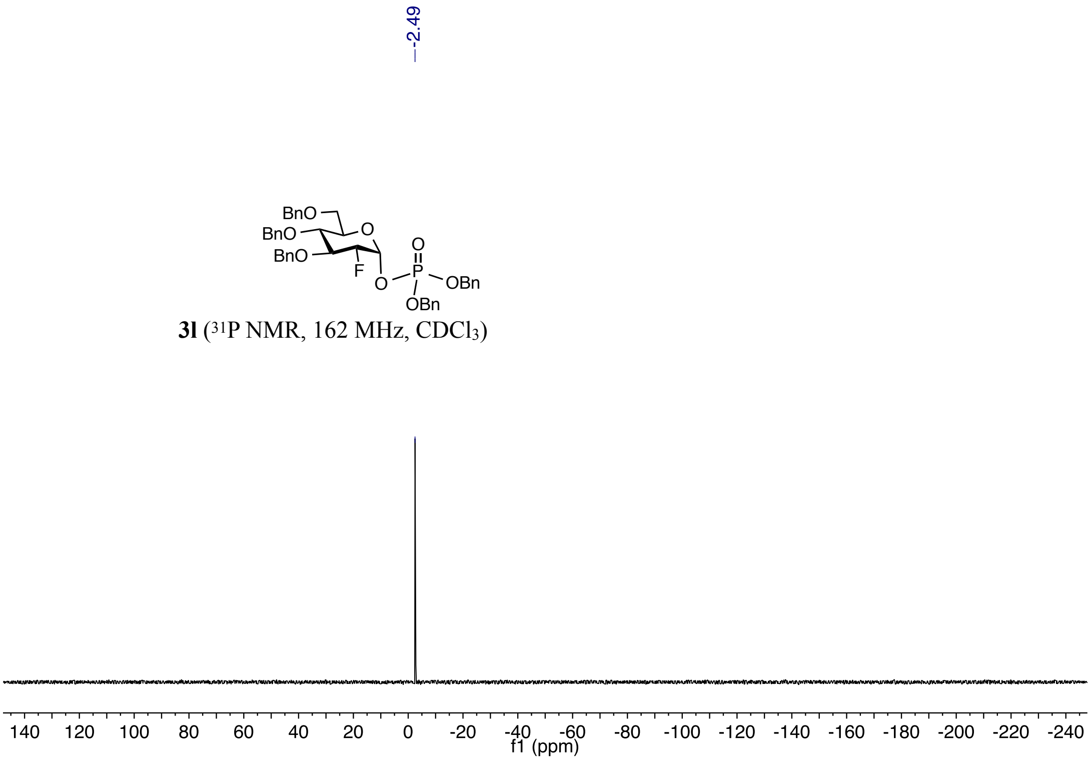

Supplementary Figure 51. ^{19}F NMR spectrum of compound **3l**

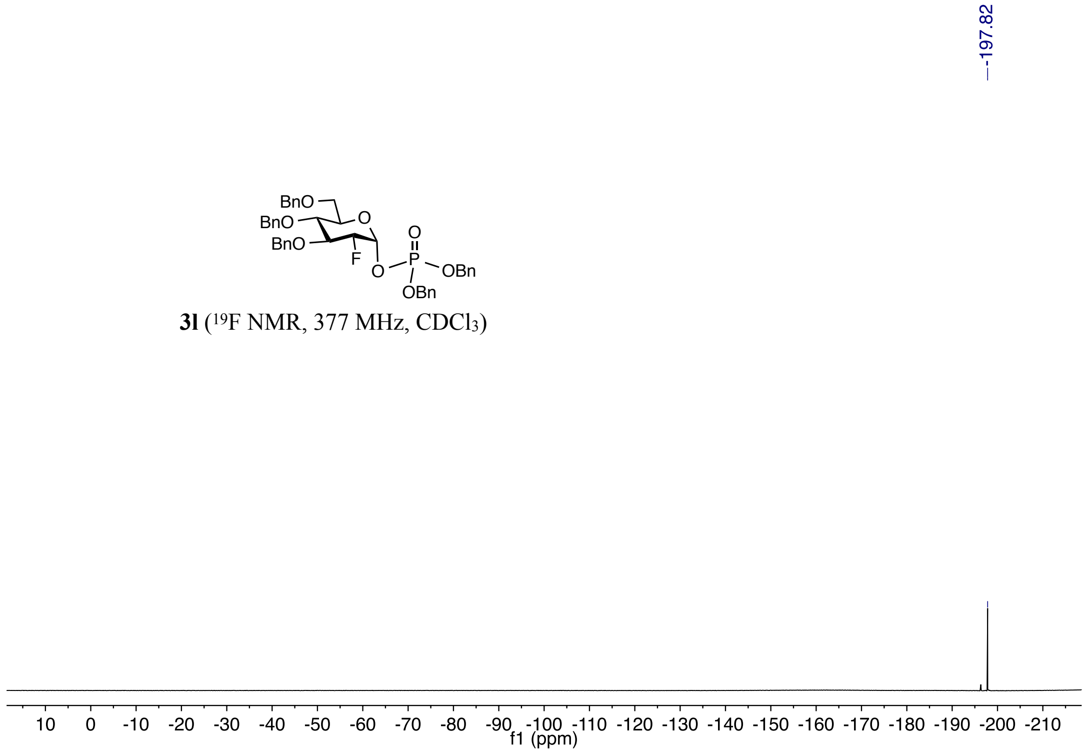

Supplementary Figure 52. ^{1}H NMR spectrum of compound **3m**

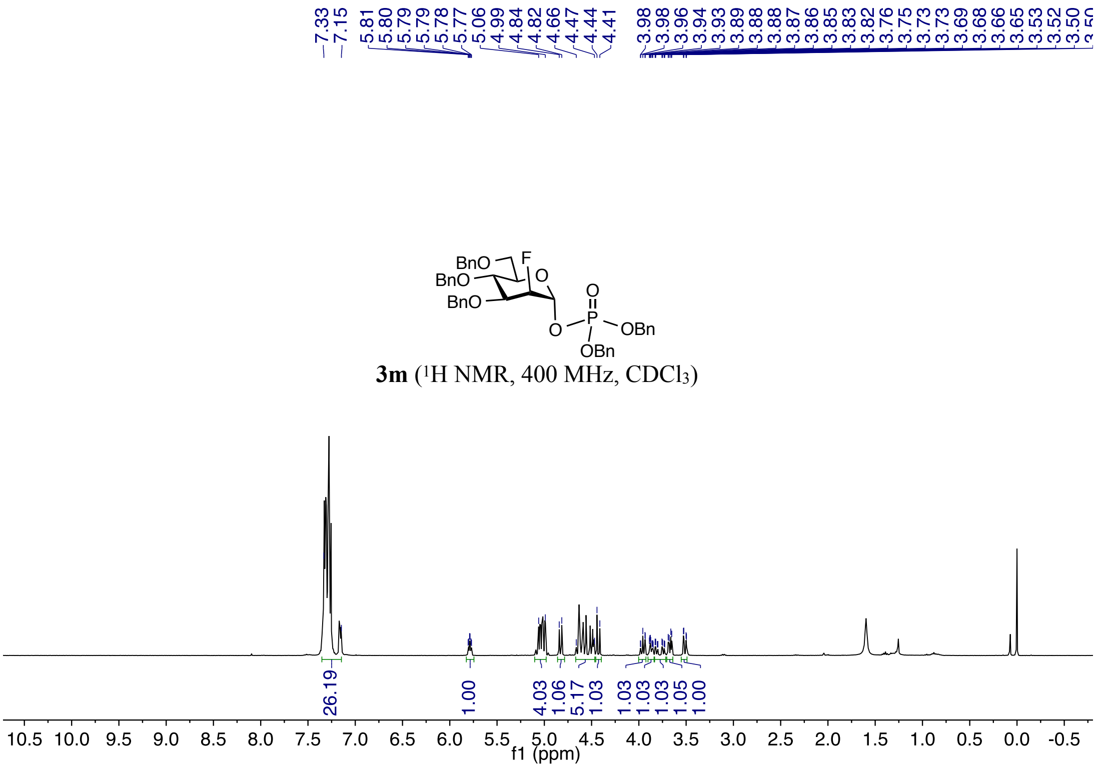

Supplementary Figure 53. ^{13}C NMR spectrum of compound **3m**

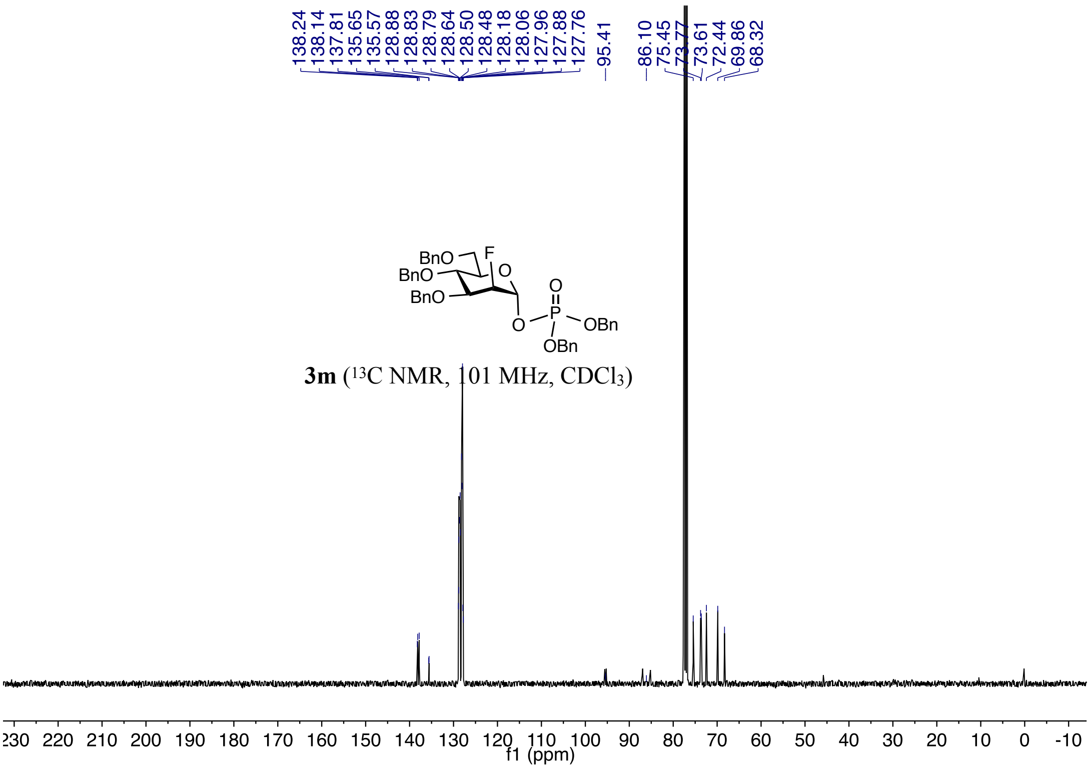

Supplementary Figure 54. ^{31}P NMR spectrum of compound **3m**

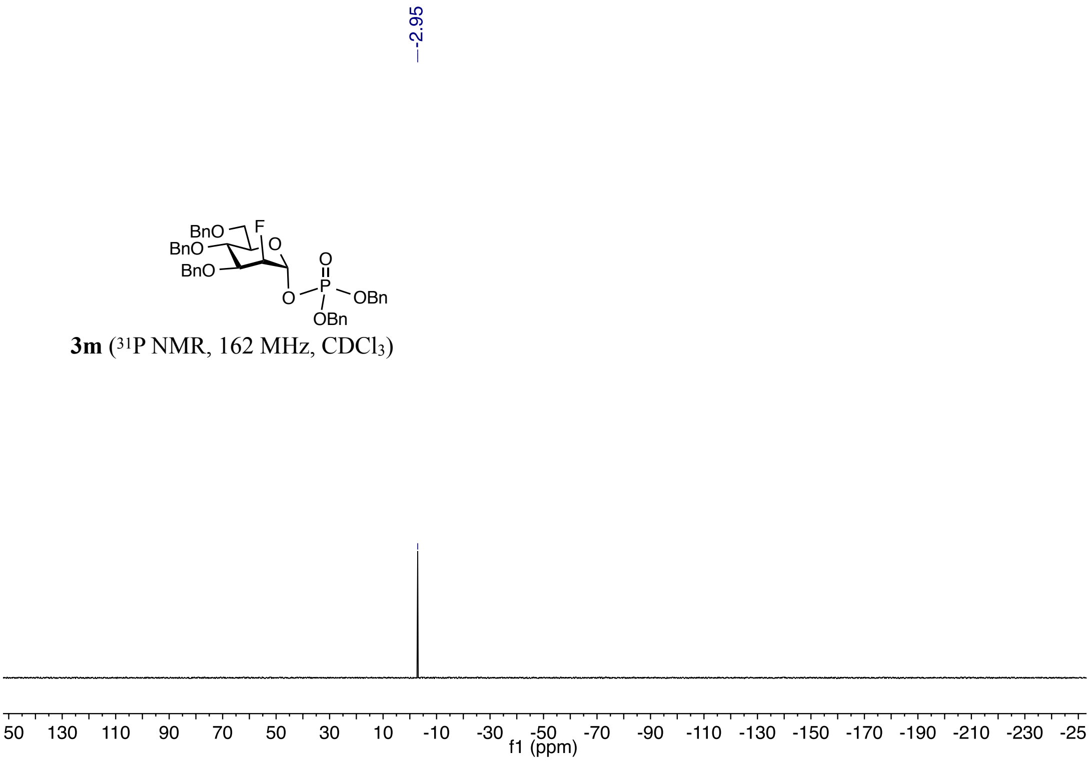

Supplementary Figure 55. ^{19}F NMR spectrum of compound **3m**

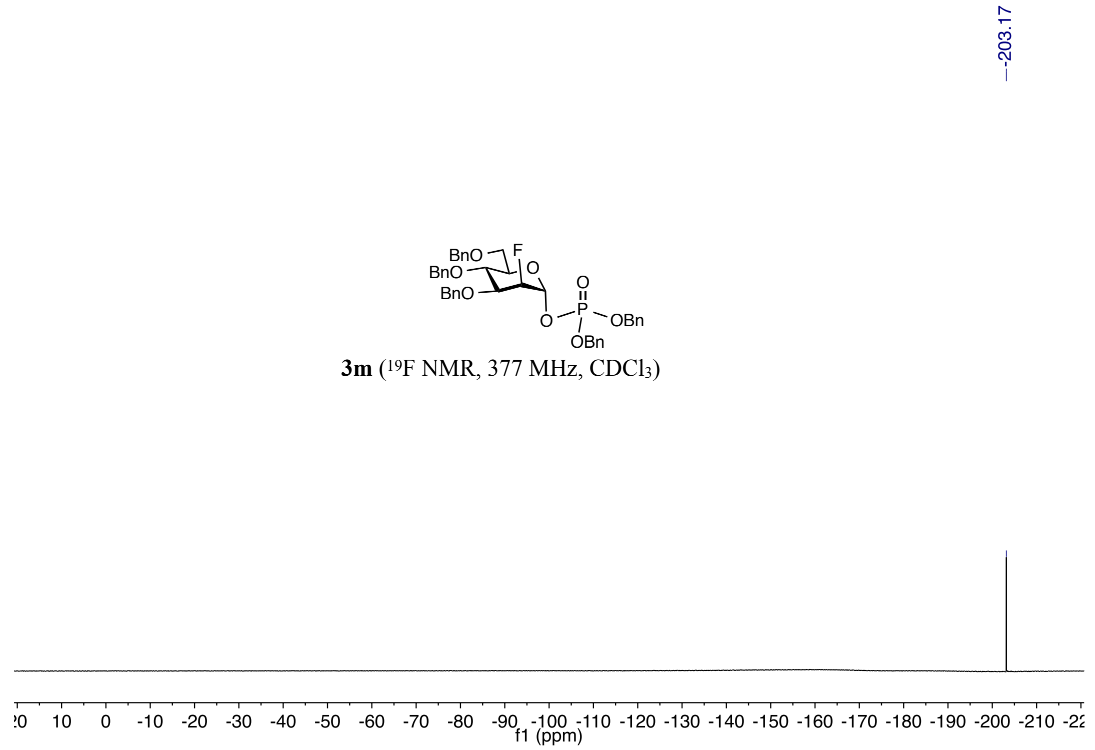

Supplementary Figure 56. ^{1}H NMR spectrum of compound **3n**

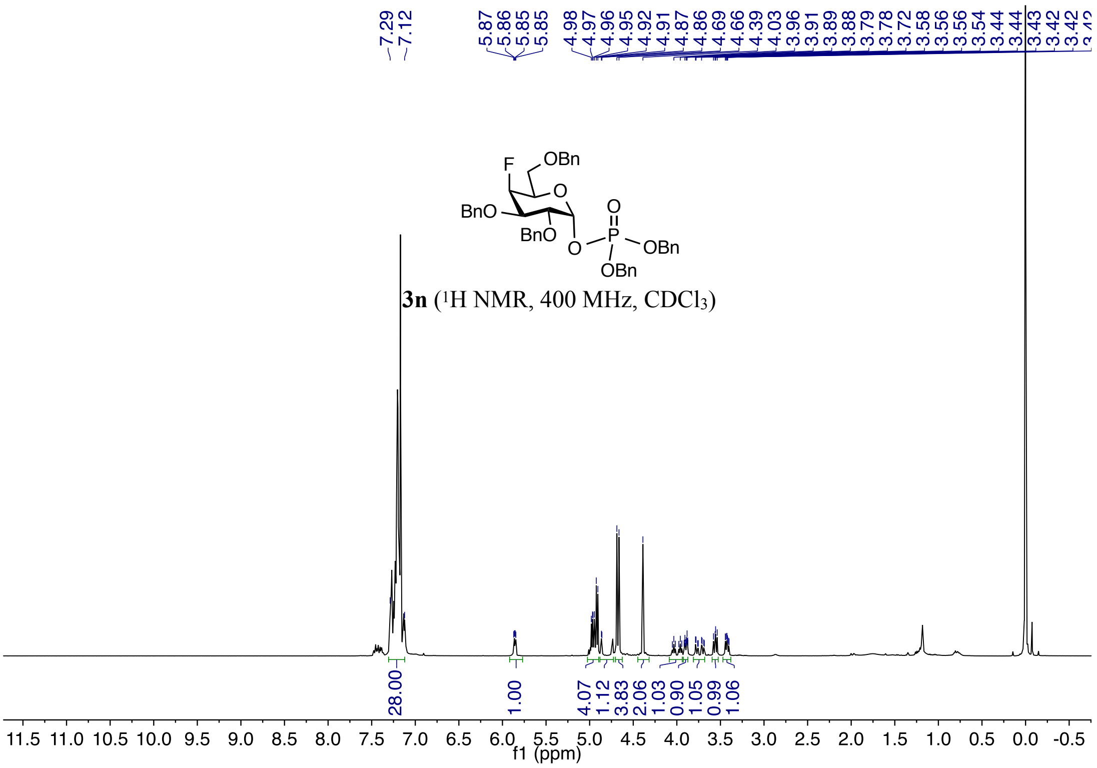

Supplementary Figure 57. ^{13}C NMR spectrum of compound **3n**

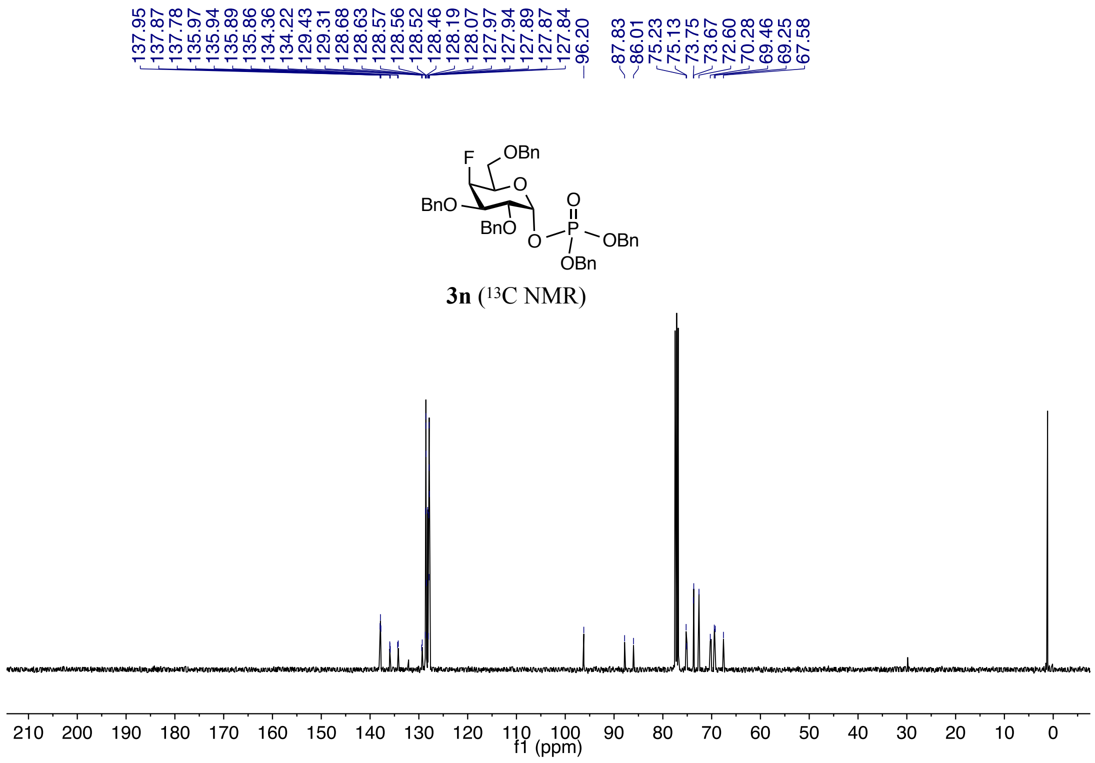

Supplementary Figure 58. ^{31}P NMR spectrum of compound **3n**

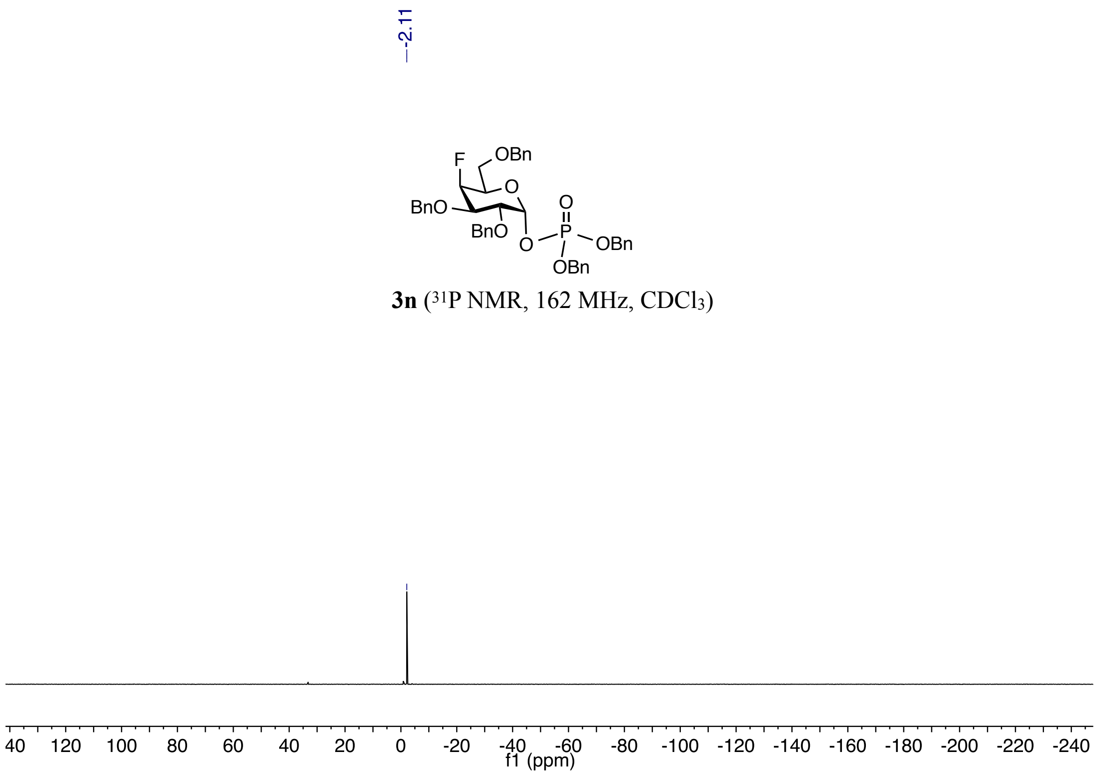

Supplementary Figure 59. ^{19}F NMR spectrum of compound **3n**

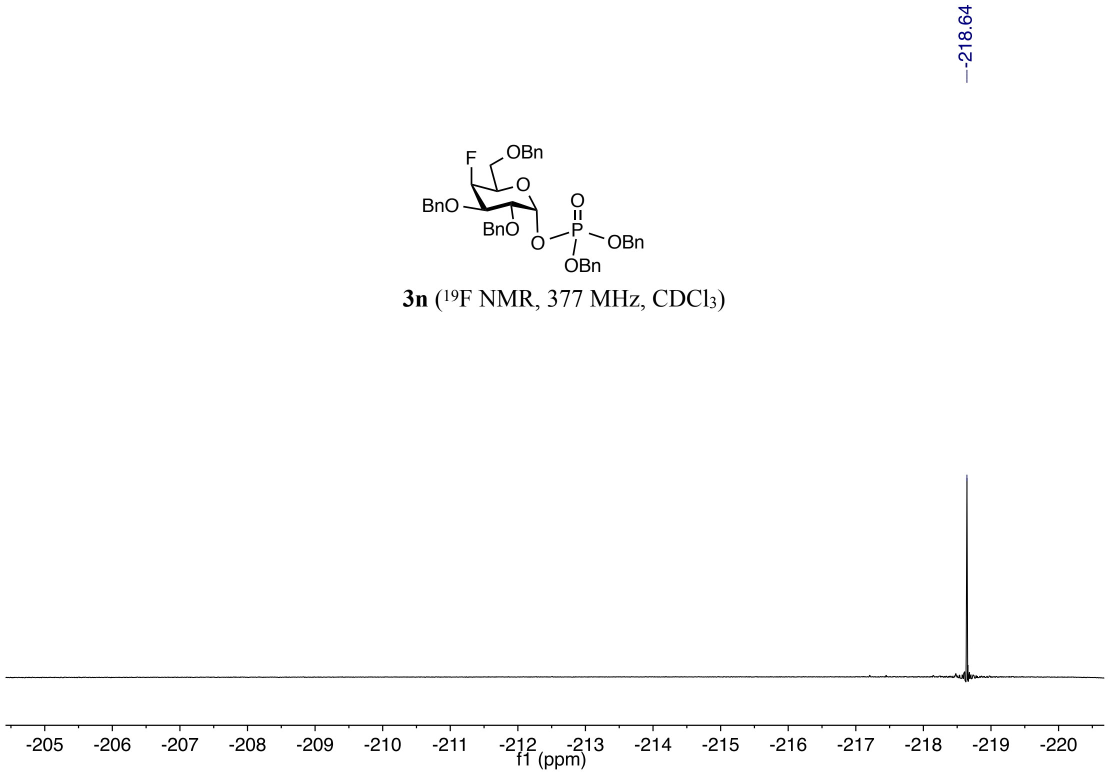

Supplementary Figure 60. ^{1}H NMR spectrum of compound **3o**

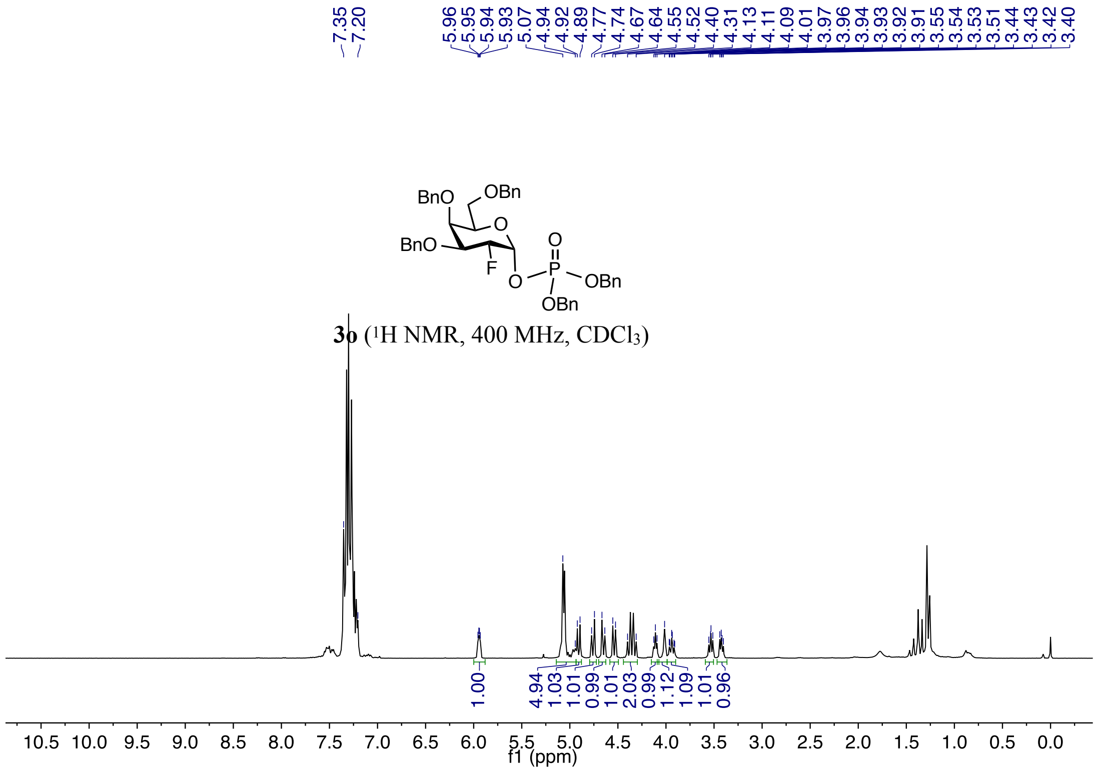

Supplementary Figure 61. ^{13}C NMR spectrum of compound **3o**

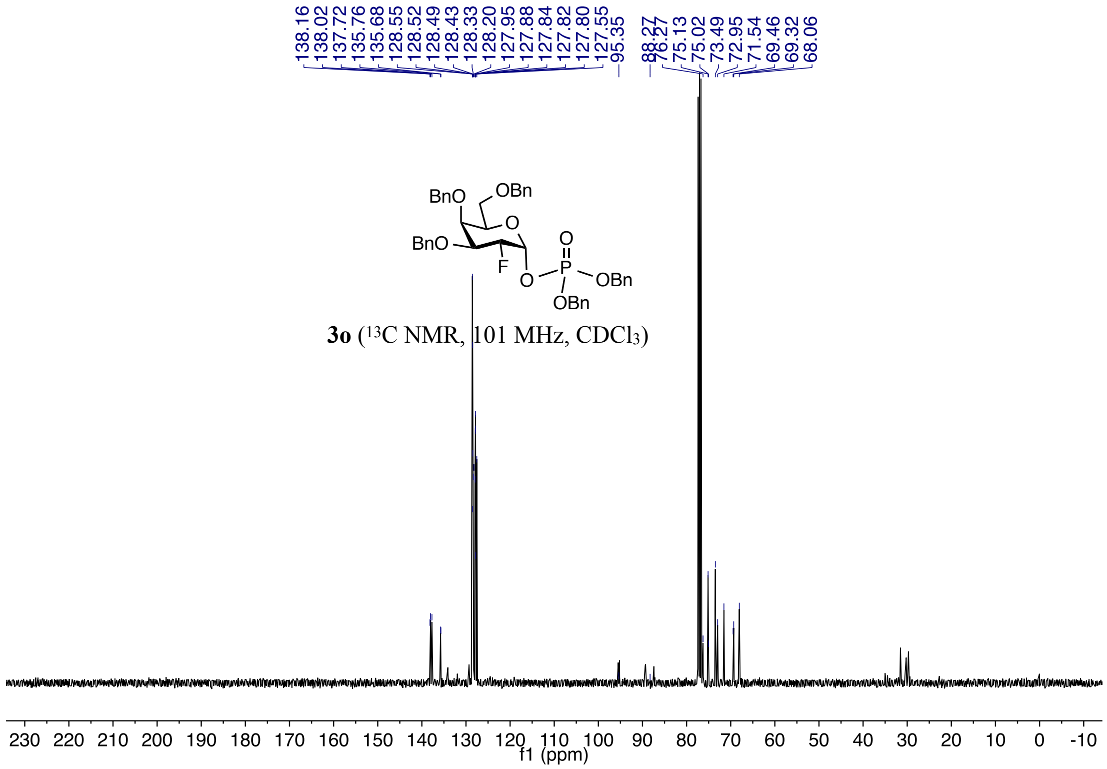

Supplementary Figure 62. ^{31}P NMR spectrum of compound **3o**

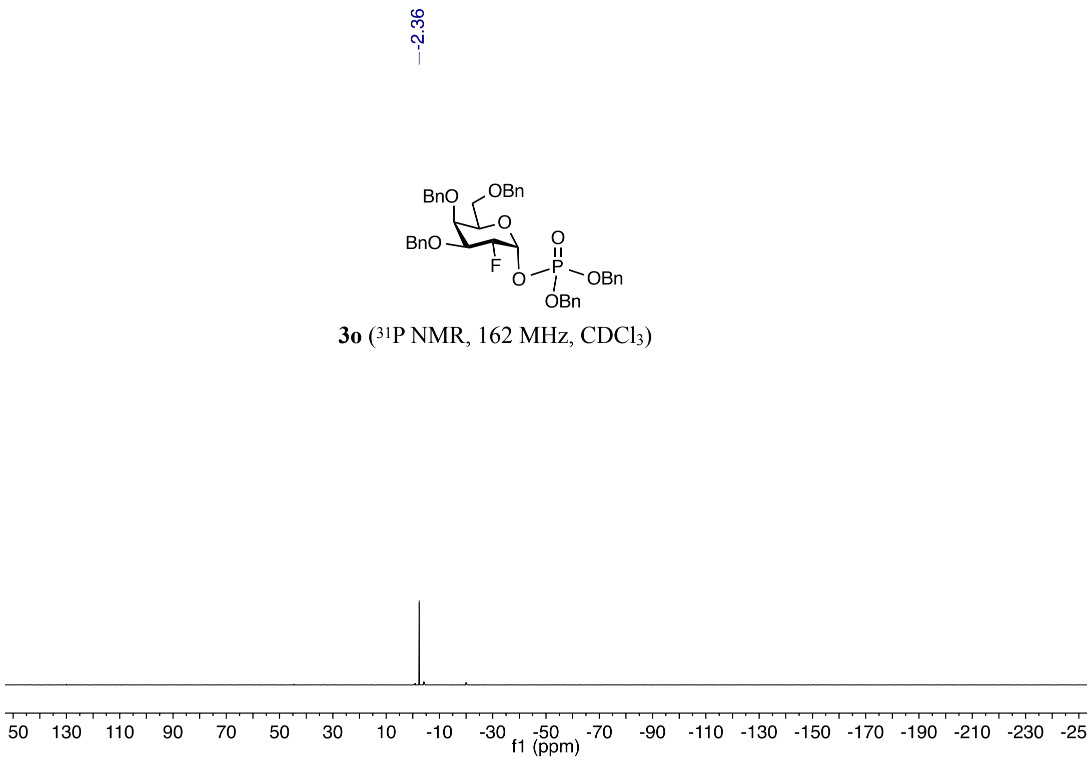

Supplementary Figure 63. ^{19}F NMR spectrum of compound **3o**

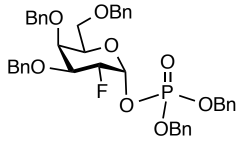

3o (^{19}F NMR, 377 MHz, $CDCl_3$)

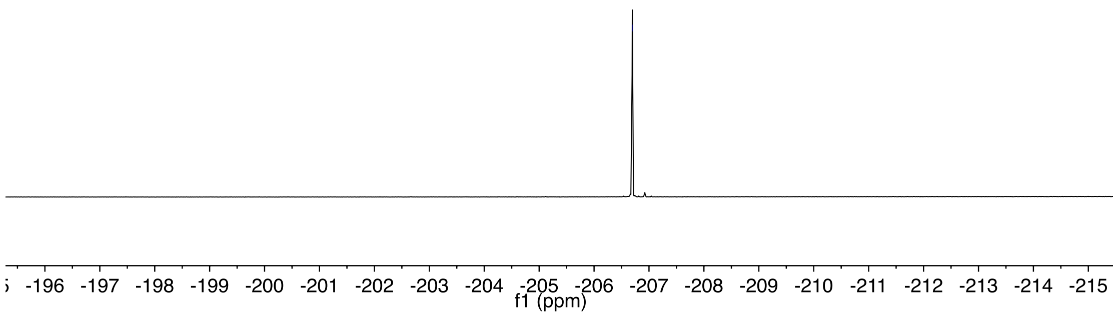

Supplementary Figure 64. ^{1}H NMR spectrum of compound **3p**

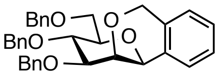

3p (^{1}H NMR, 400 MHz, $CDCl_3$)

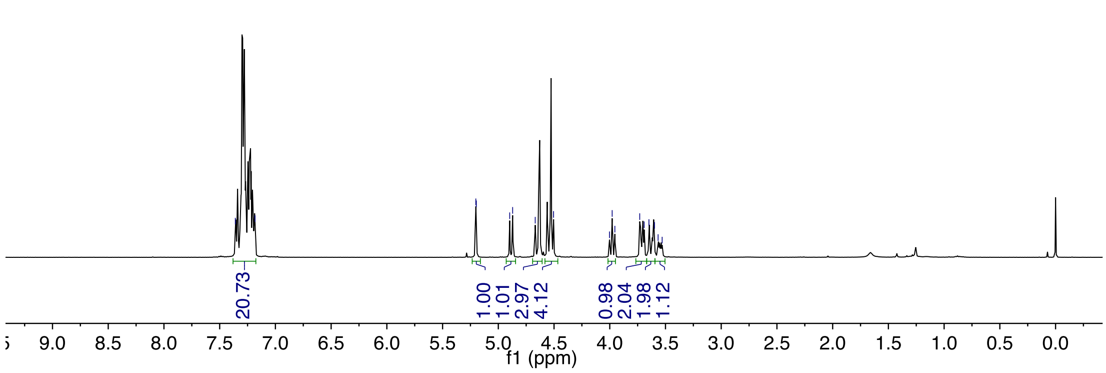

Supplementary Figure 65. ^{13}C NMR spectrum of compound **3p**

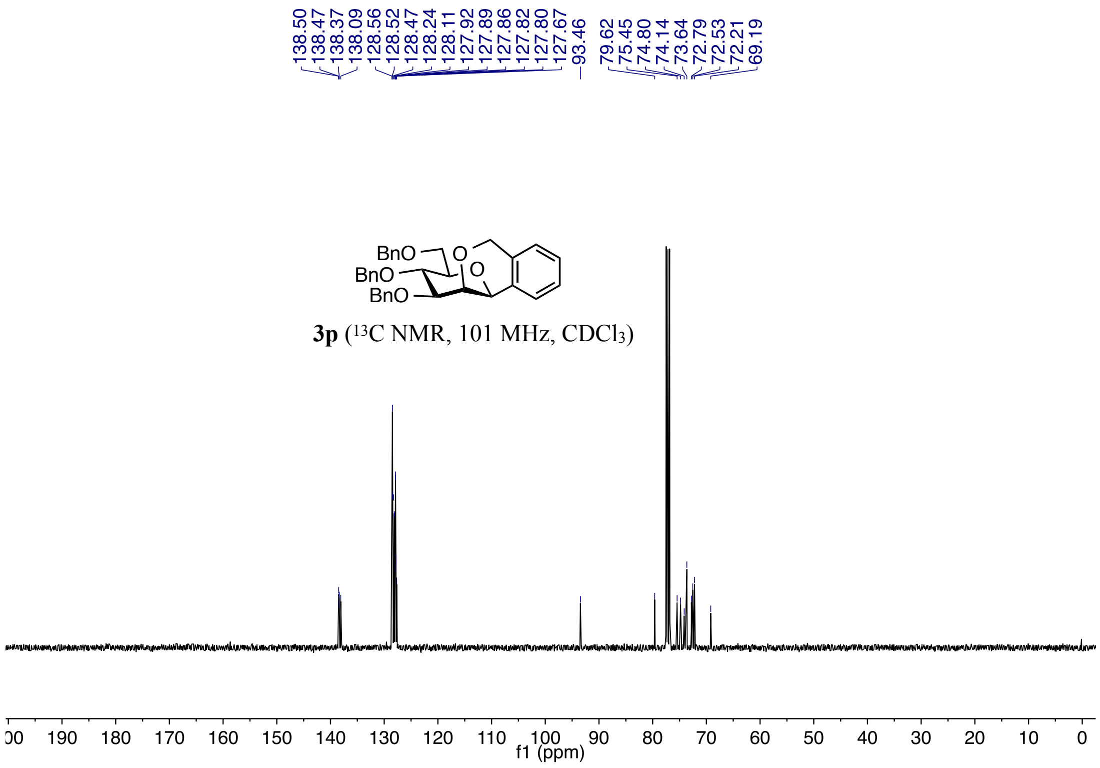

Supplementary Figure 66. ^{1}H NMR spectrum of compound **4a**

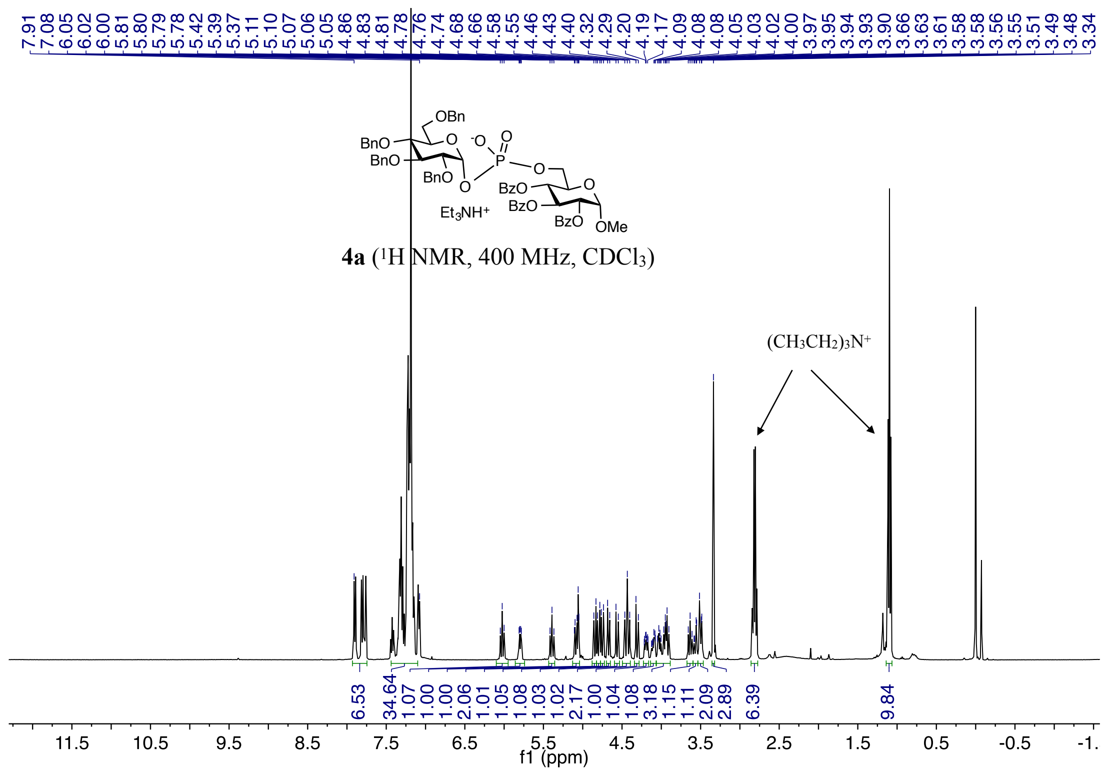

Supplementary Figure 67. ^{13}C NMR spectrum of compound **4a**

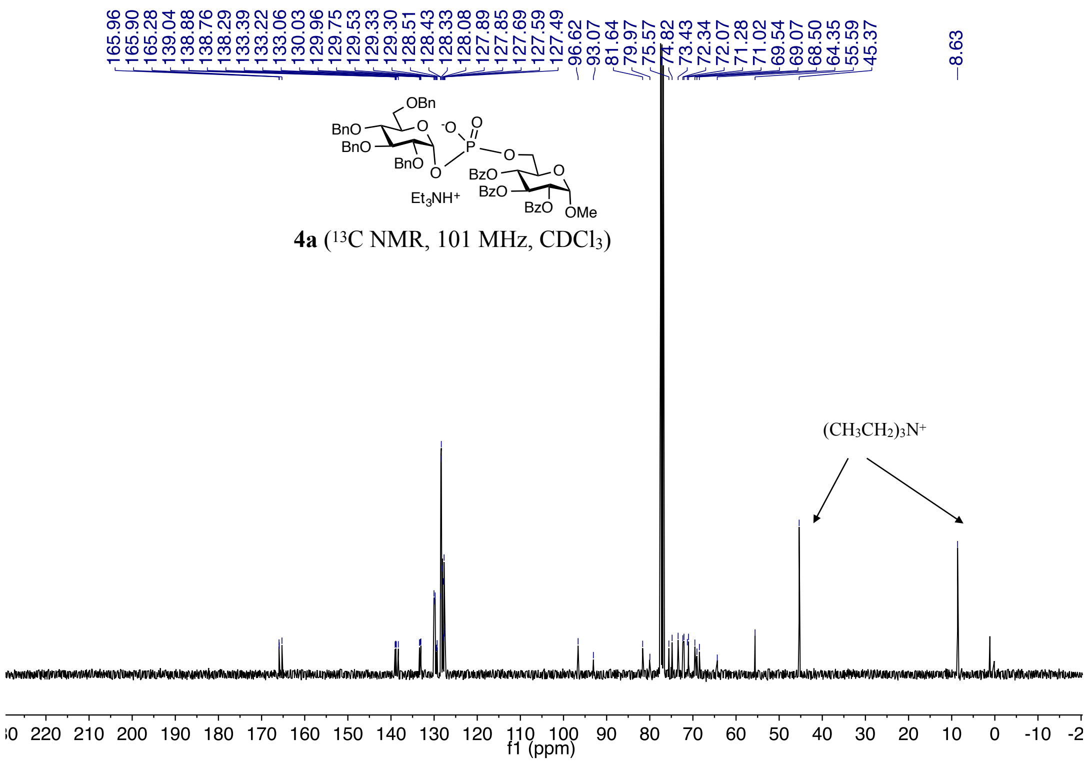

Supplementary Figure 68. ^{31}P NMR spectrum of compound **4a**

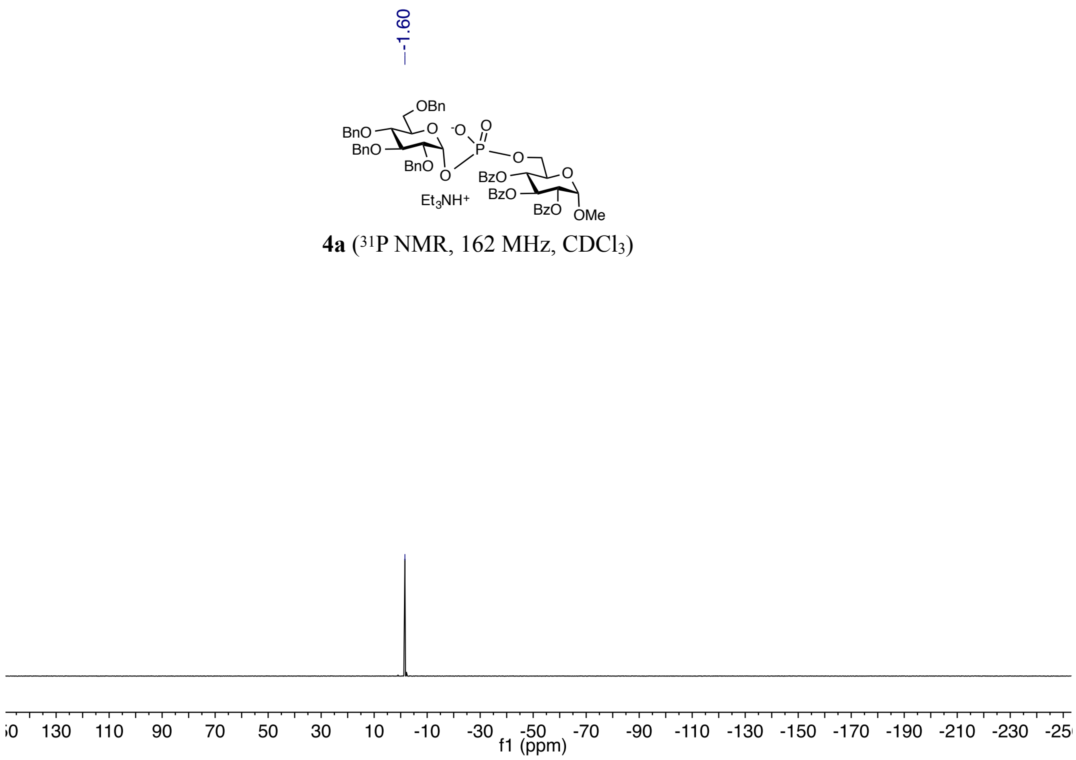

Supplementary Figure 69. ^{1}H NMR spectrum of compound **4b**

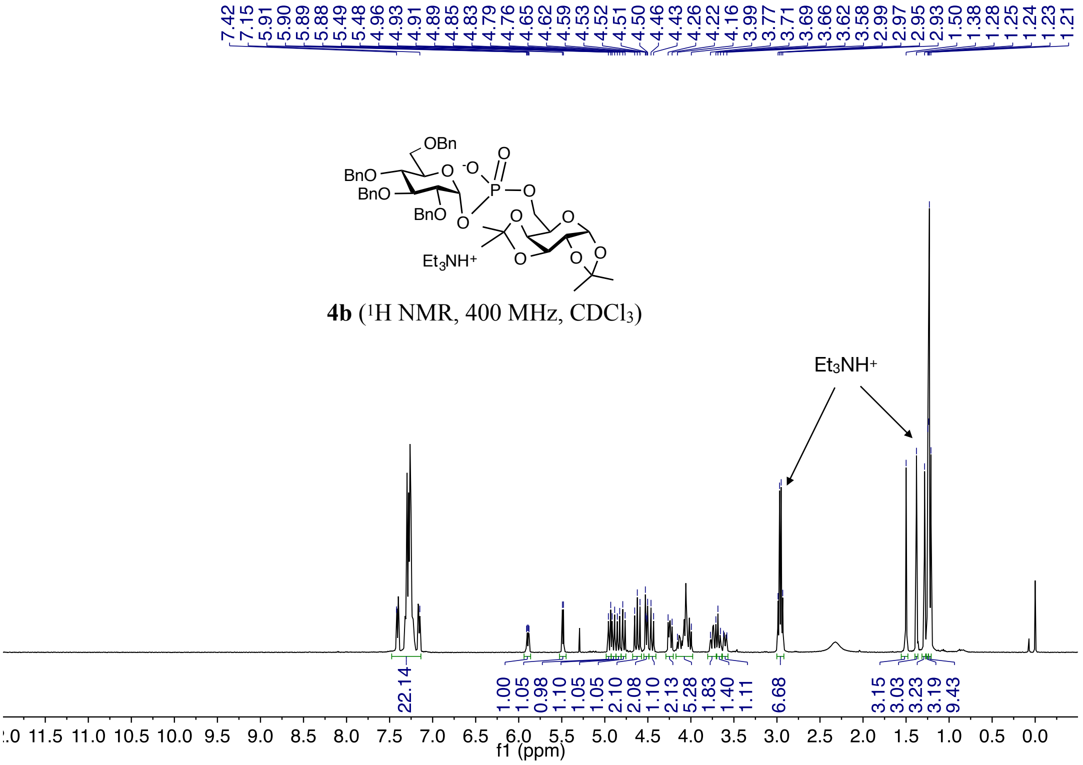

Supplementary Figure 70. ^{13}C NMR spectrum of compound **4b**

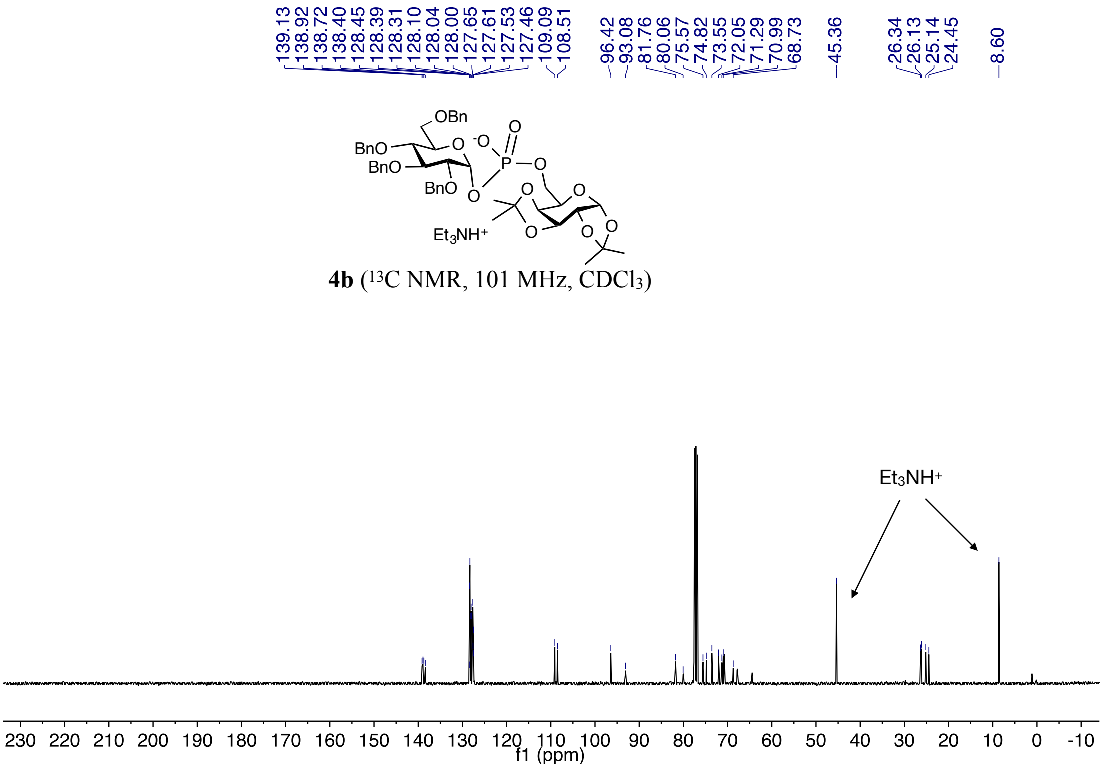

Supplementary Figure 71. ^{31}P NMR spectrum of compound **4b**

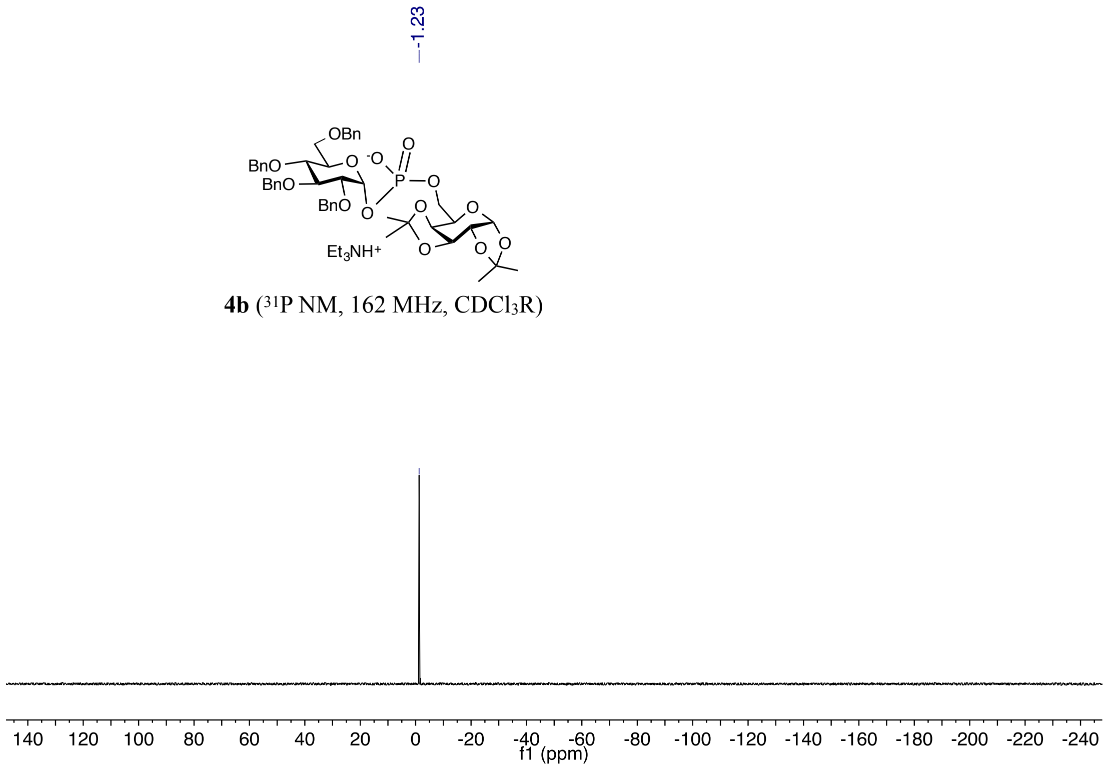

Supplementary Figure 72. ^{1}H NMR spectrum of compound **4c**

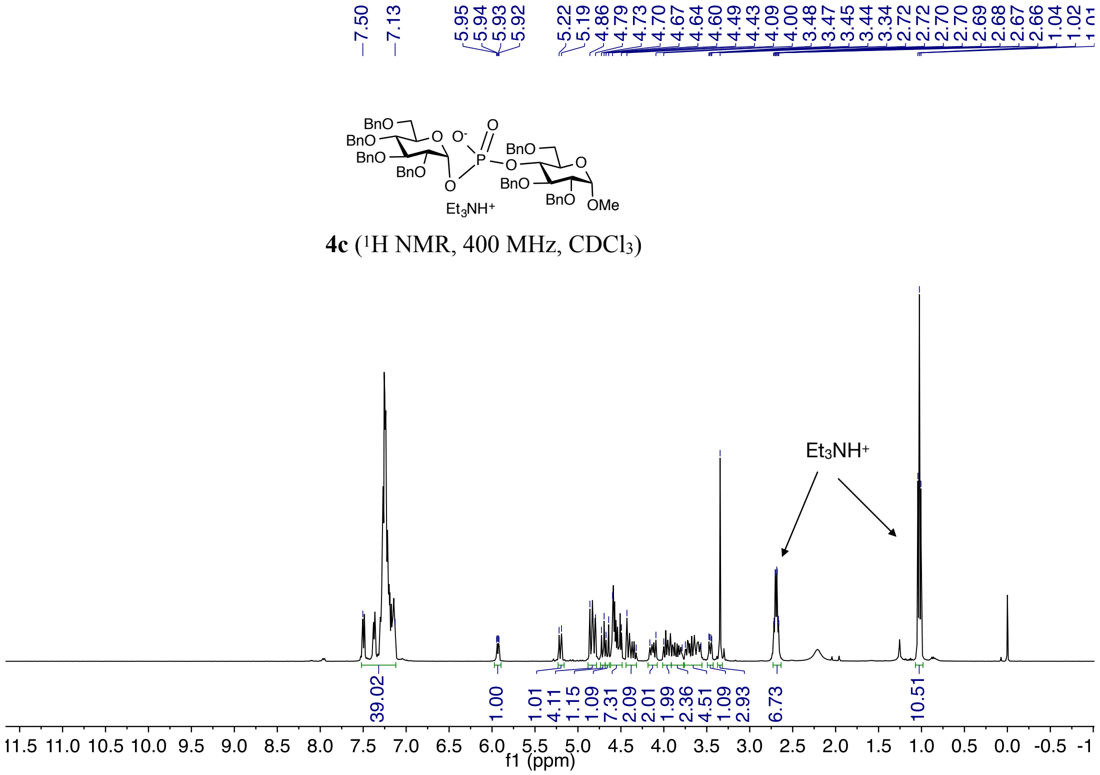

Supplementary Figure 73. ^{13}C NMR spectrum of compound **4c**

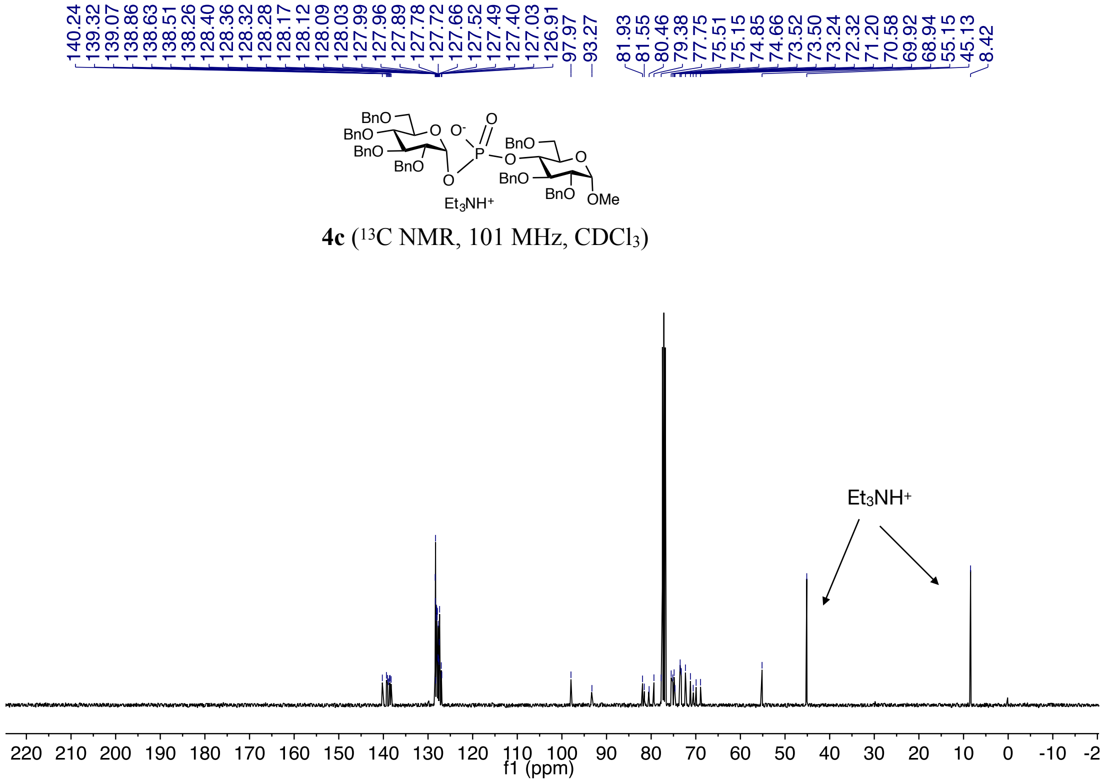

Supplementary Figure 74. ^{31}P NMR spectrum of compound **4c**

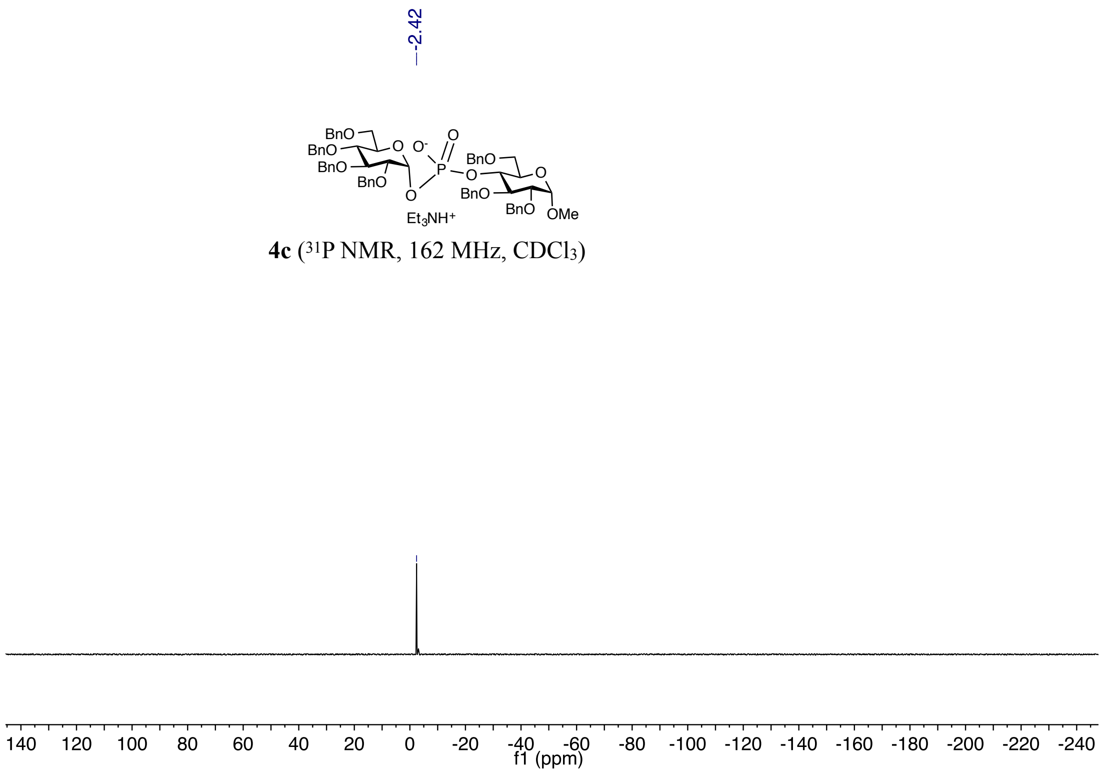

Supplementary Figure 75. ^{1}H NMR spectrum of compound **4d**

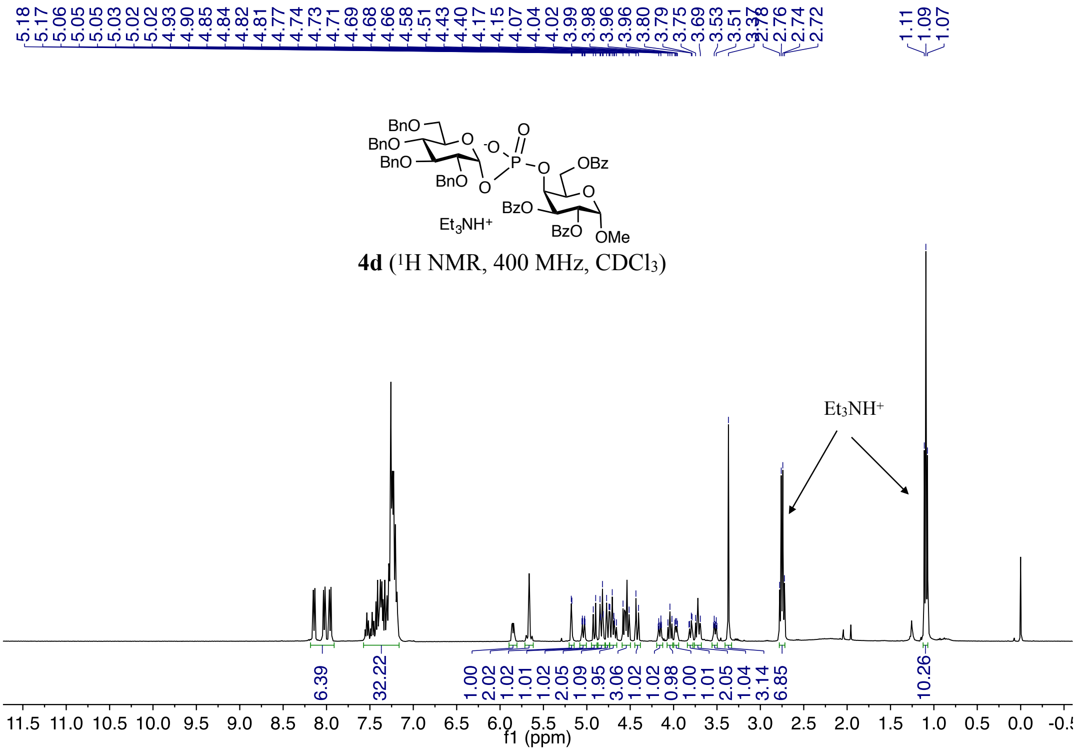

Supplementary Figure 76. ^{13}C NMR spectrum of compound **4d**

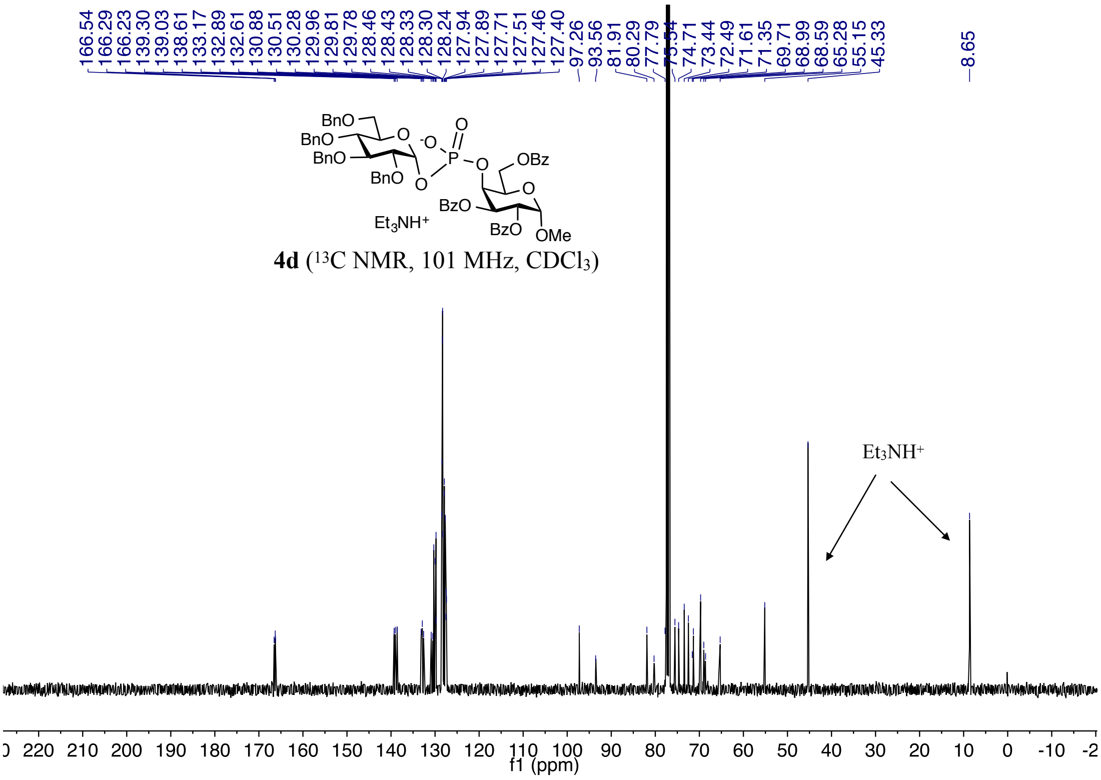

Supplementary Figure 77. ^{31}P NMR spectrum of compound **4d**

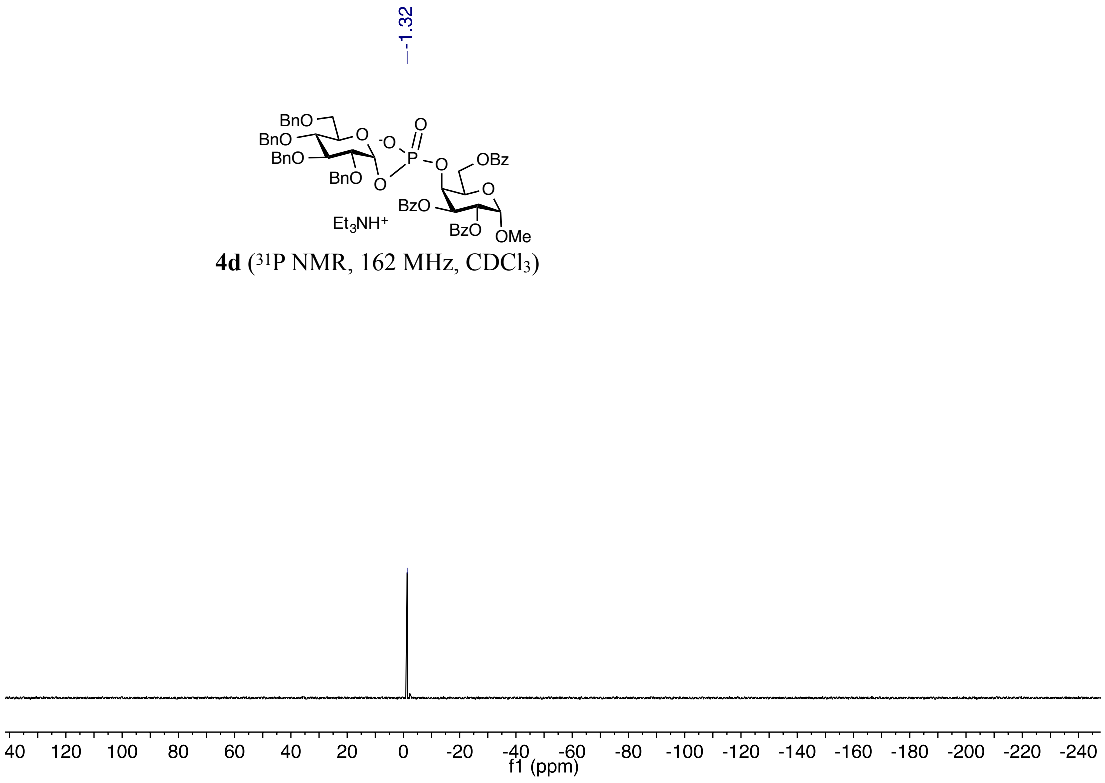

Supplementary Figure 78. ^{1}H NMR spectrum of compound **4e**

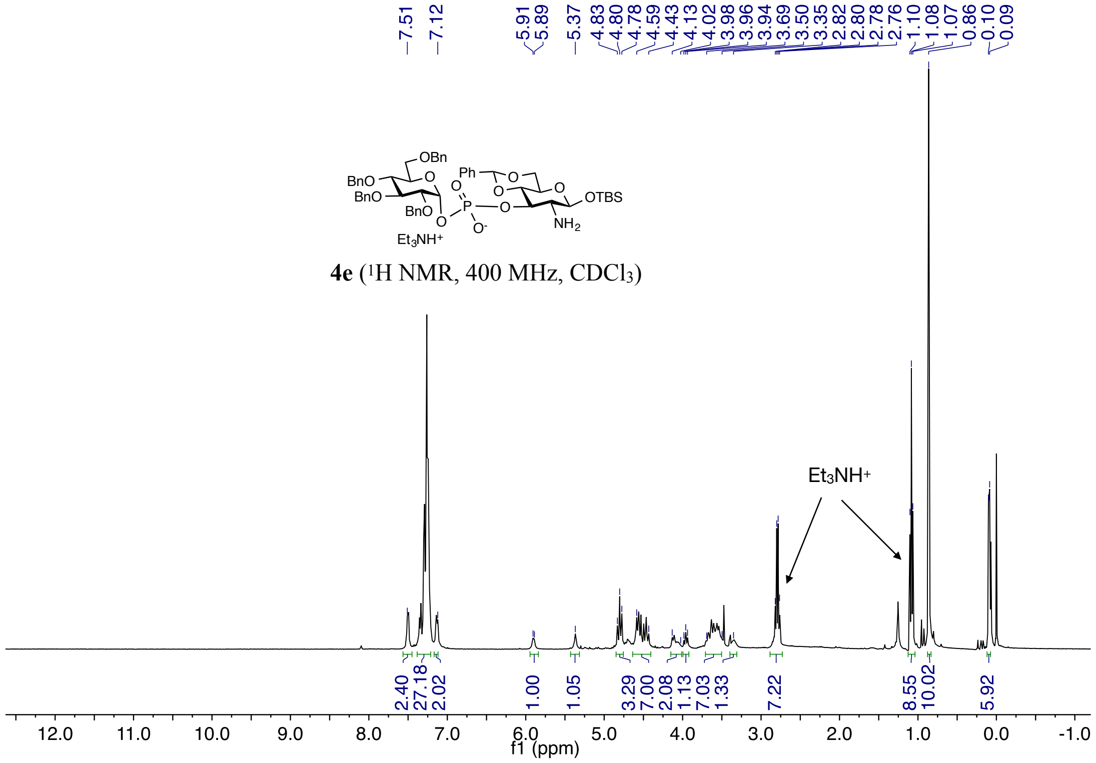

Supplementary Figure 79. ^{13}C NMR spectrum of compound **4e**

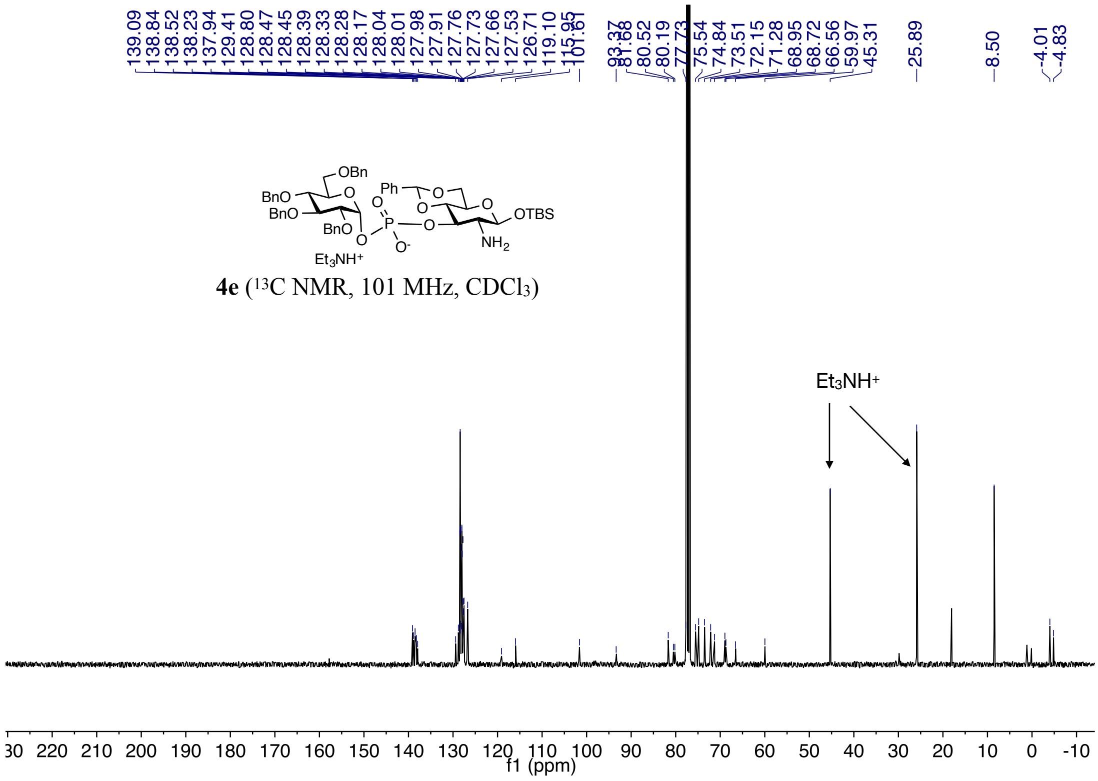

Supplementary Figure 80. ^{31}P NMR spectrum of compound **4e**

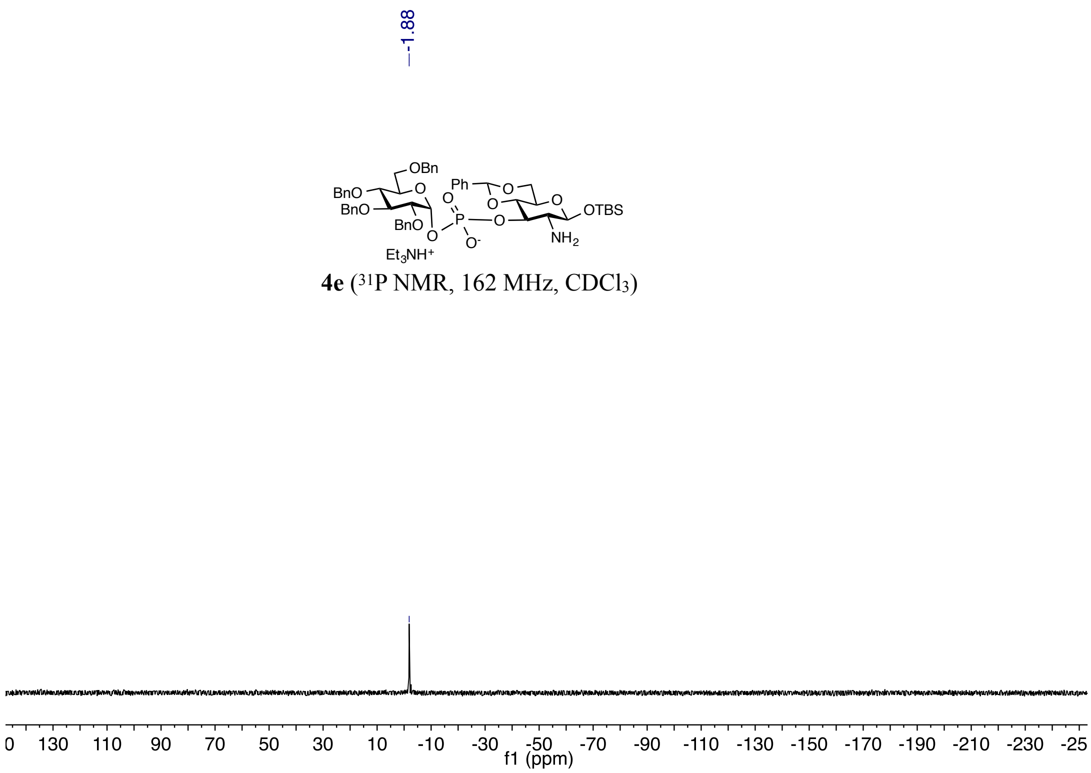

Supplementary Figure 81. ^{1}H NMR spectrum of compound **4f**

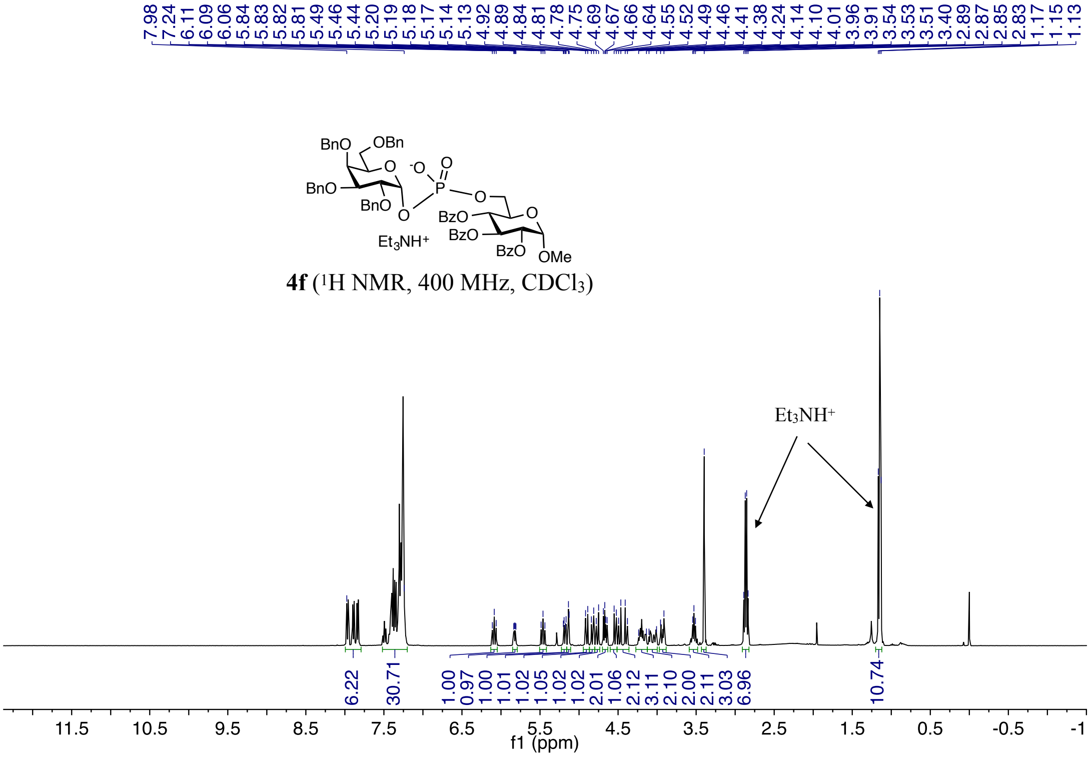

Supplementary Figure 82. ^{13}C NMR spectrum of compound **4f**

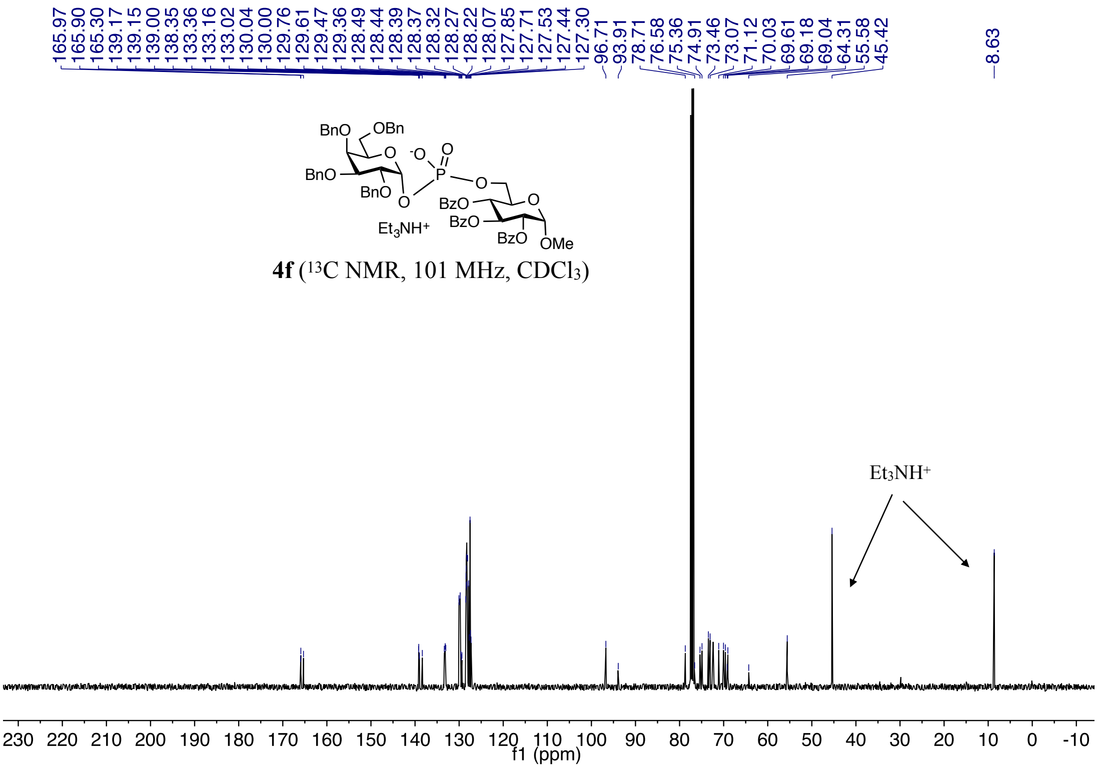

Supplementary Figure 83. ^{31}P NMR spectrum of compound **4f**

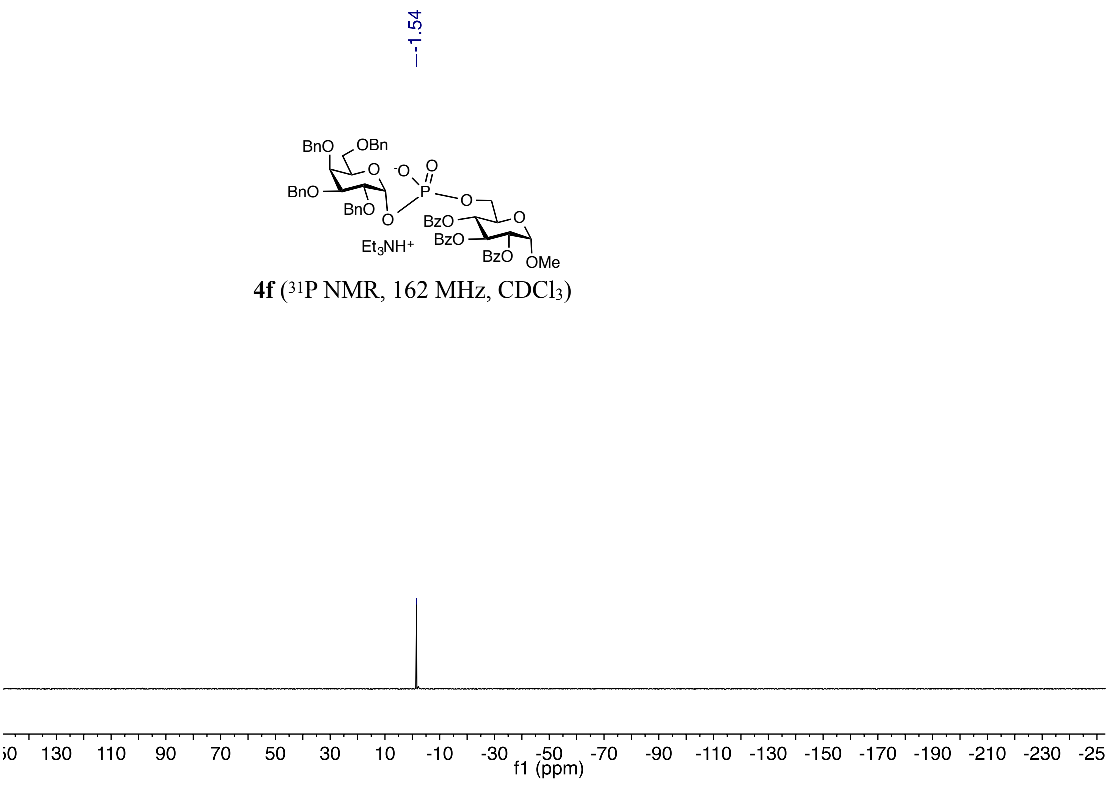

Supplementary Figure 84. ^{1}H NMR spectrum of compound **4g**

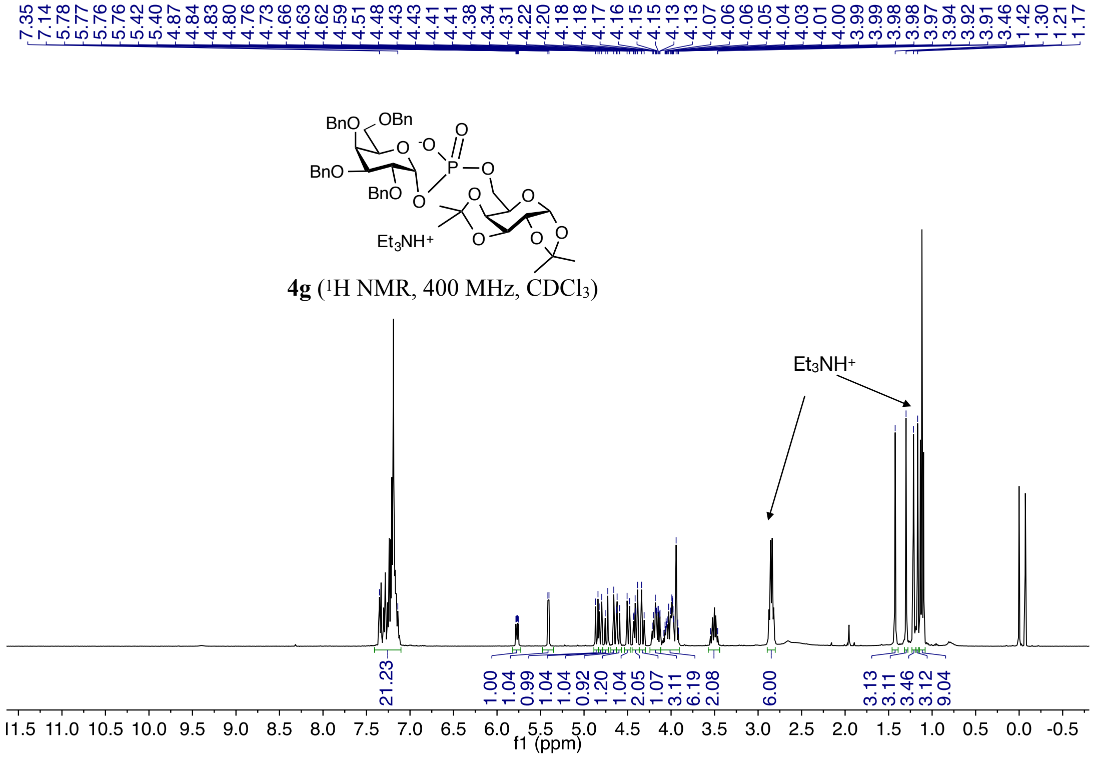

Supplementary Figure 85. ^{13}C NMR spectrum of compound **4g**

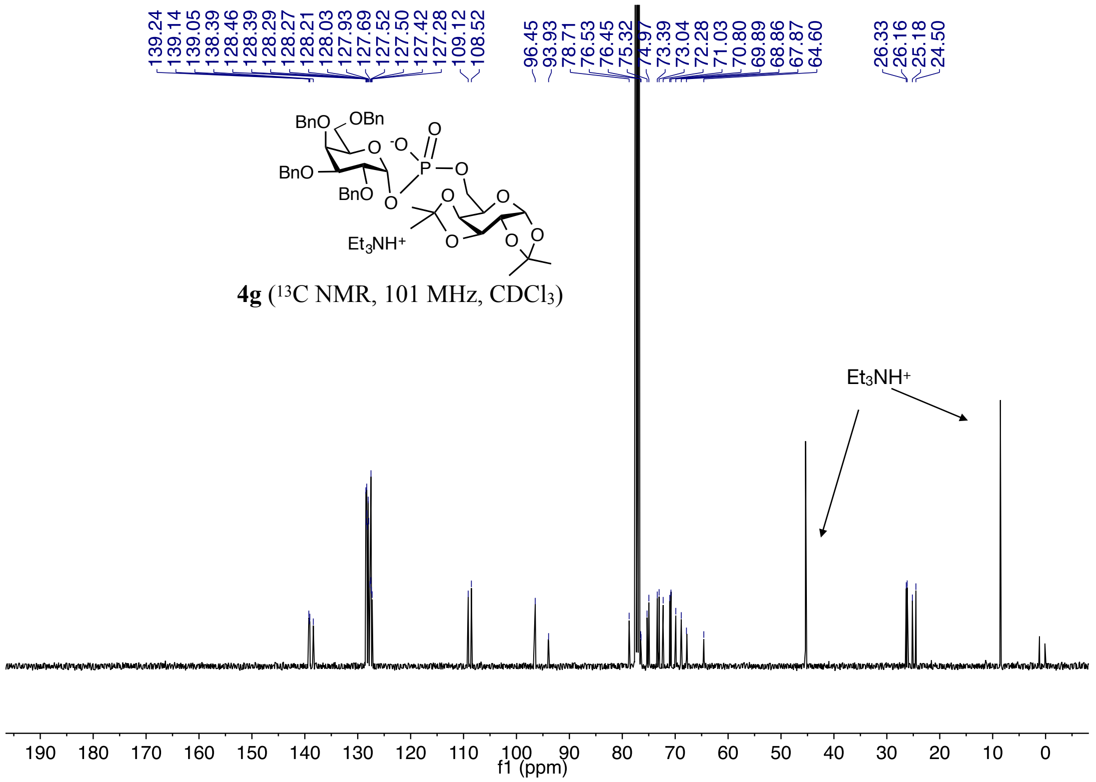

Supplementary Figure 86. ^{31}P NMR spectrum of compound **4g**

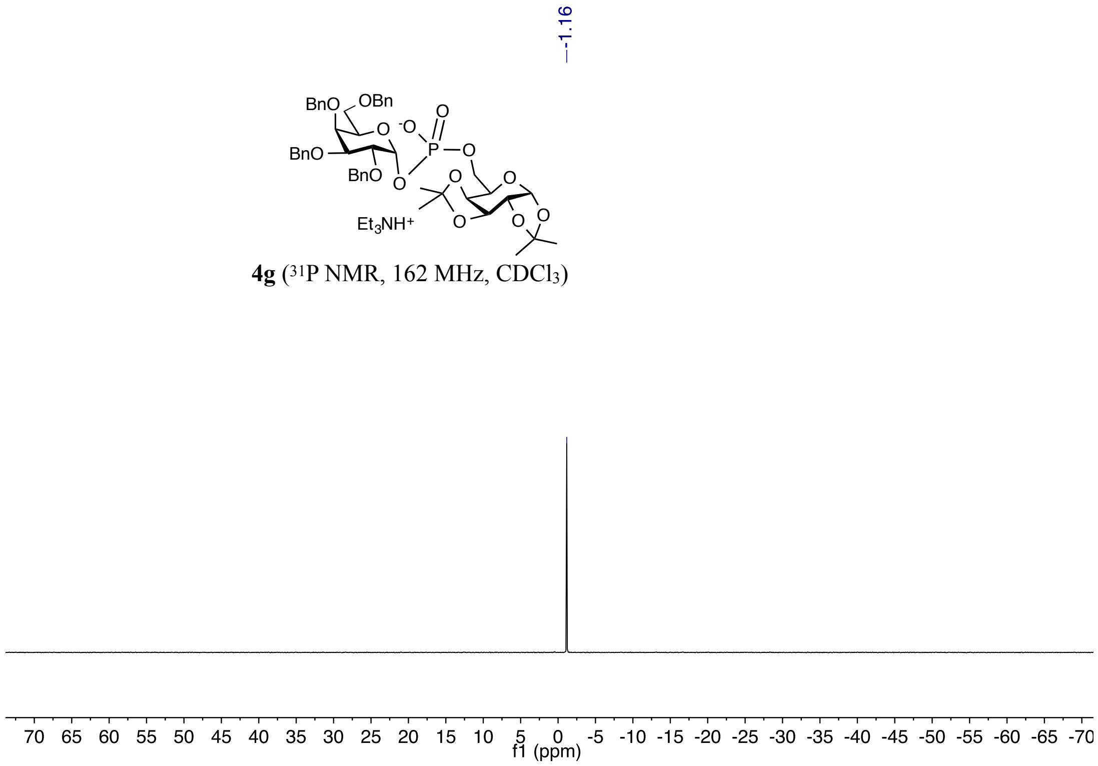

Supplementary Figure 87. ^{1}H NMR spectrum of compound **4h**

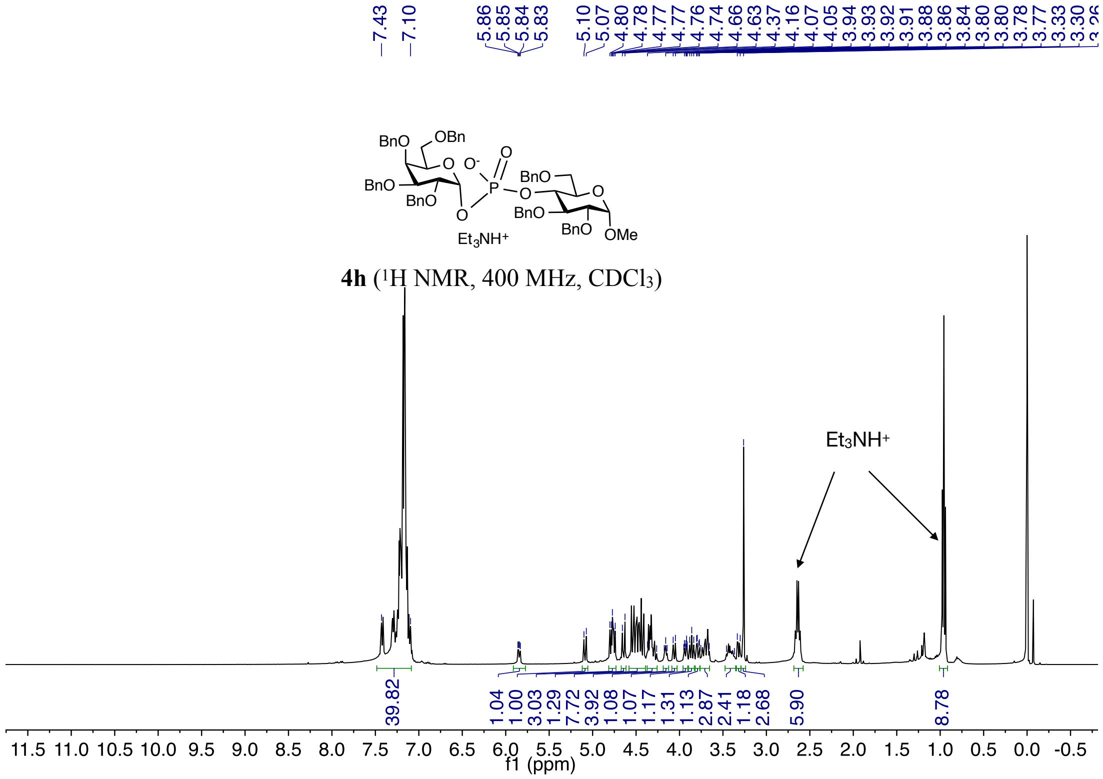

Supplementary Figure 88. ^{13}C NMR spectrum of compound **4h**

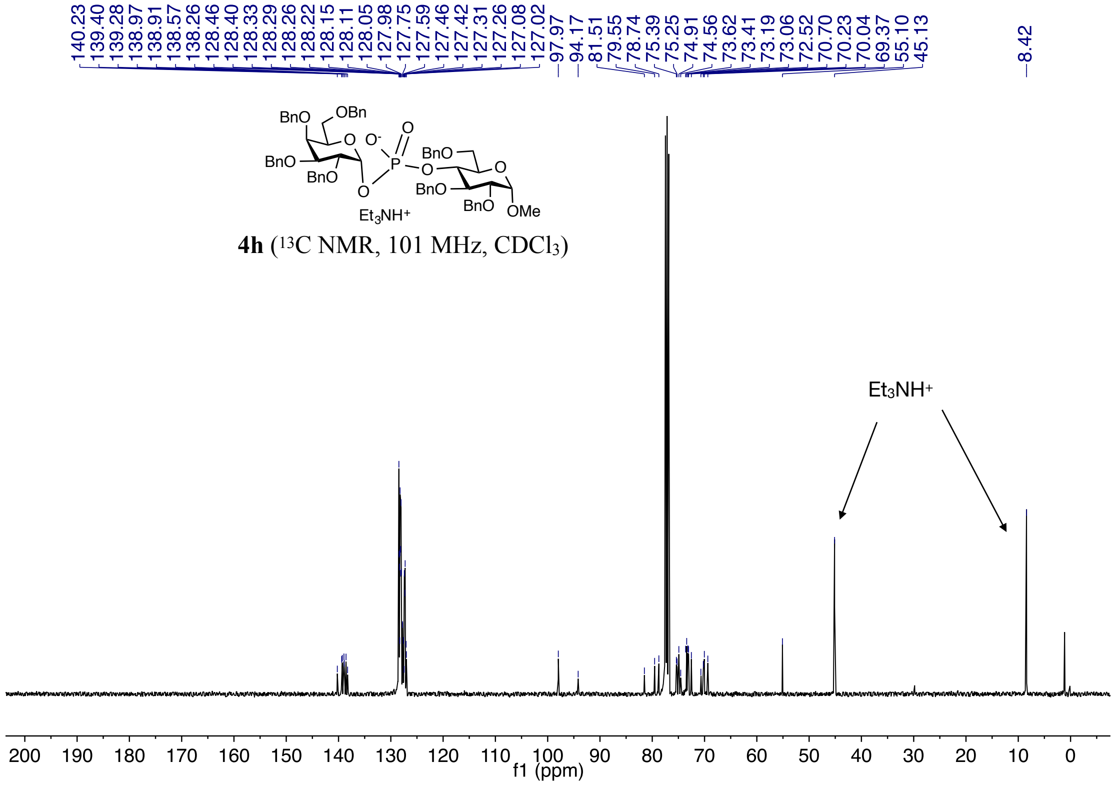

Supplementary Figure 89. ^{31}P NMR spectrum of compound **4h**

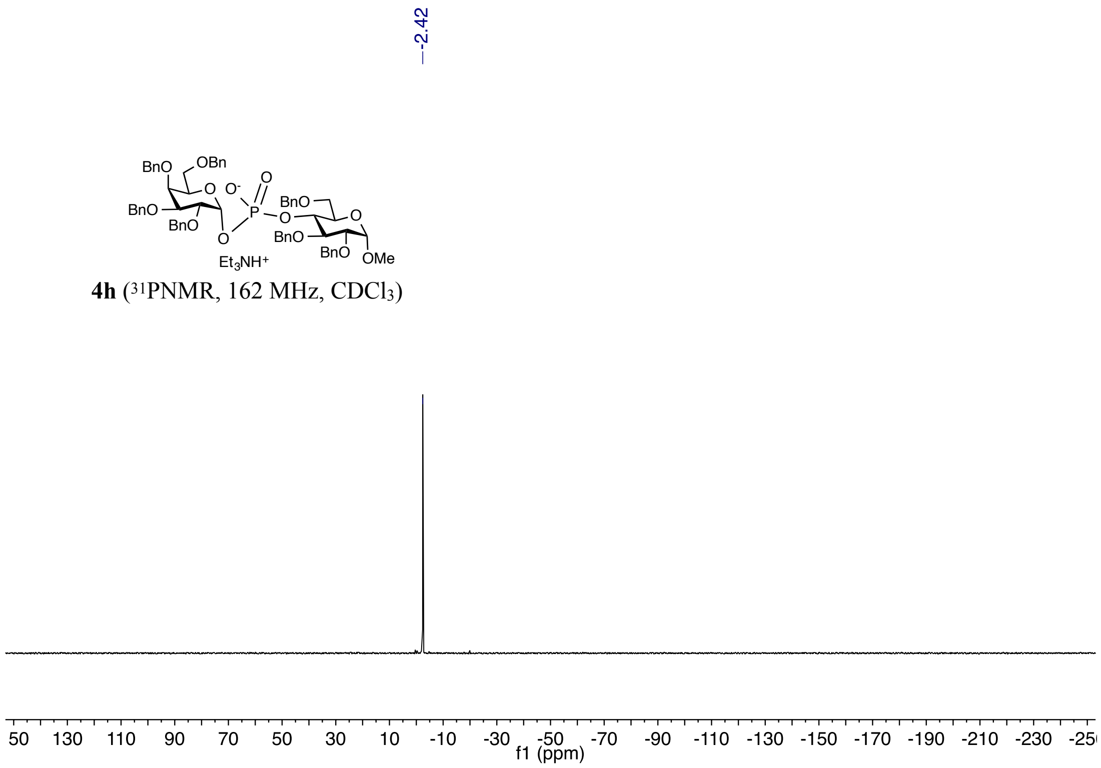

Supplementary Figure 90. ^{1}H NMR spectrum of compound **4i**

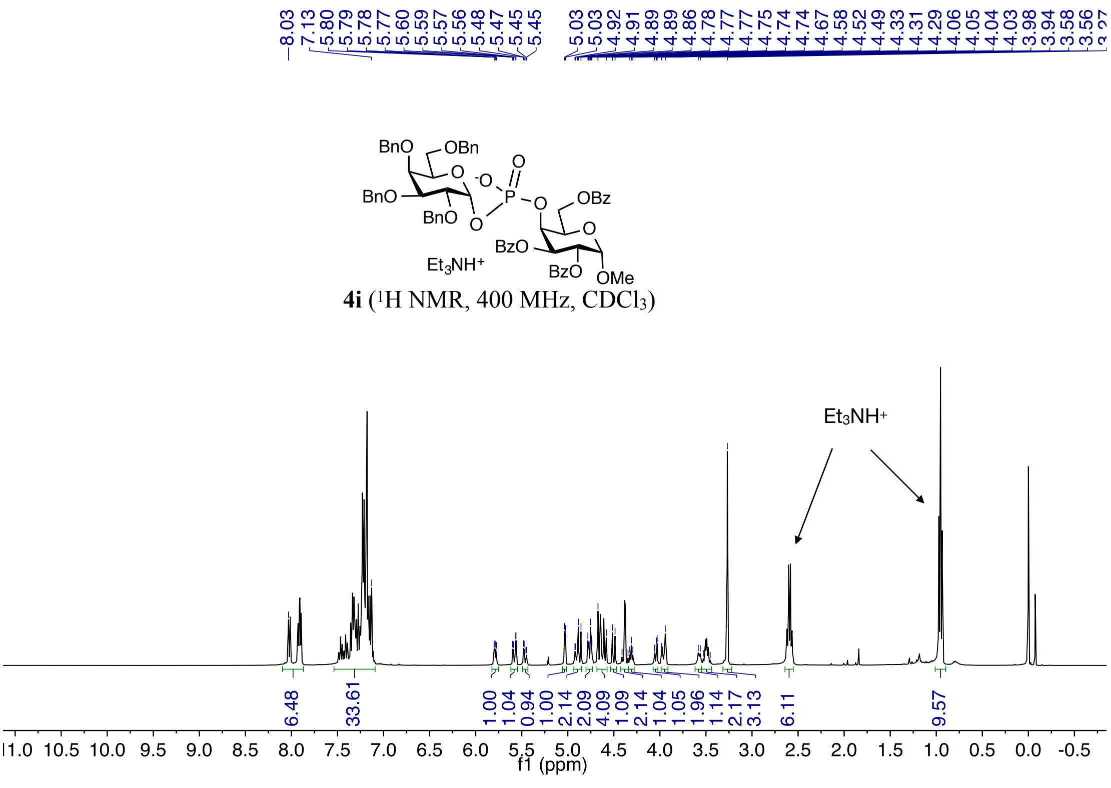

Supplementary Figure 91. ^{13}C NMR spectrum of compound **4i**

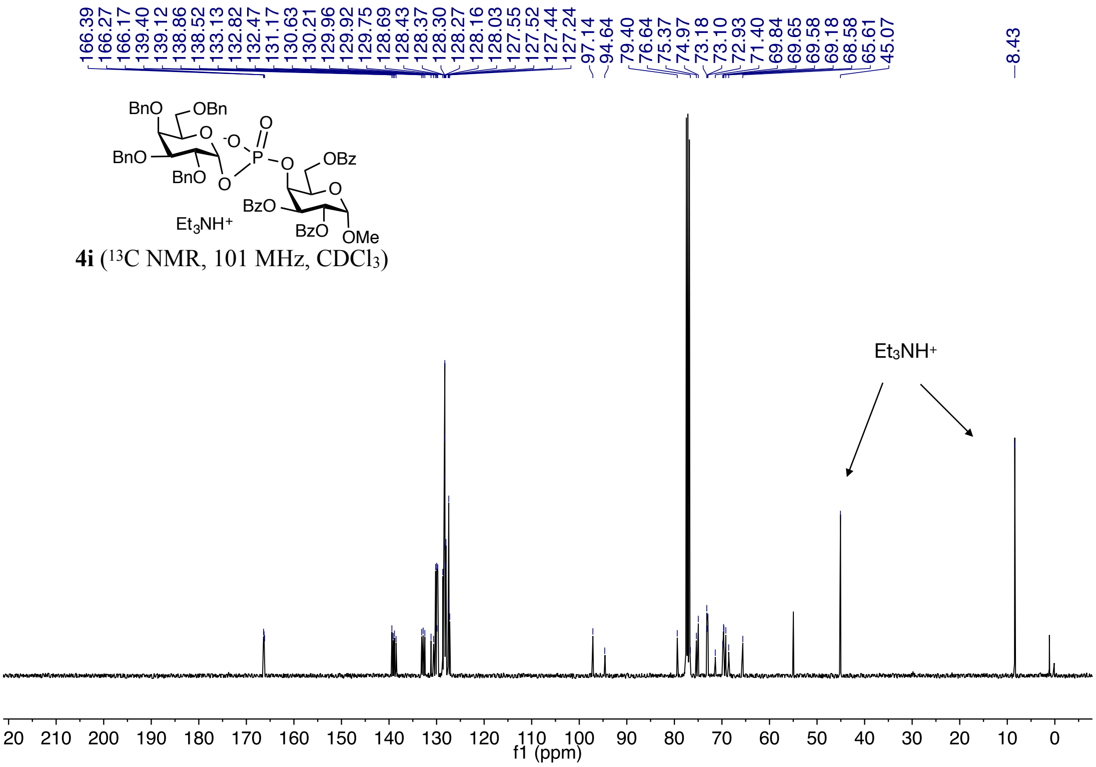

Supplementary Figure 92. ^{31}P NMR spectrum of compound **4i**

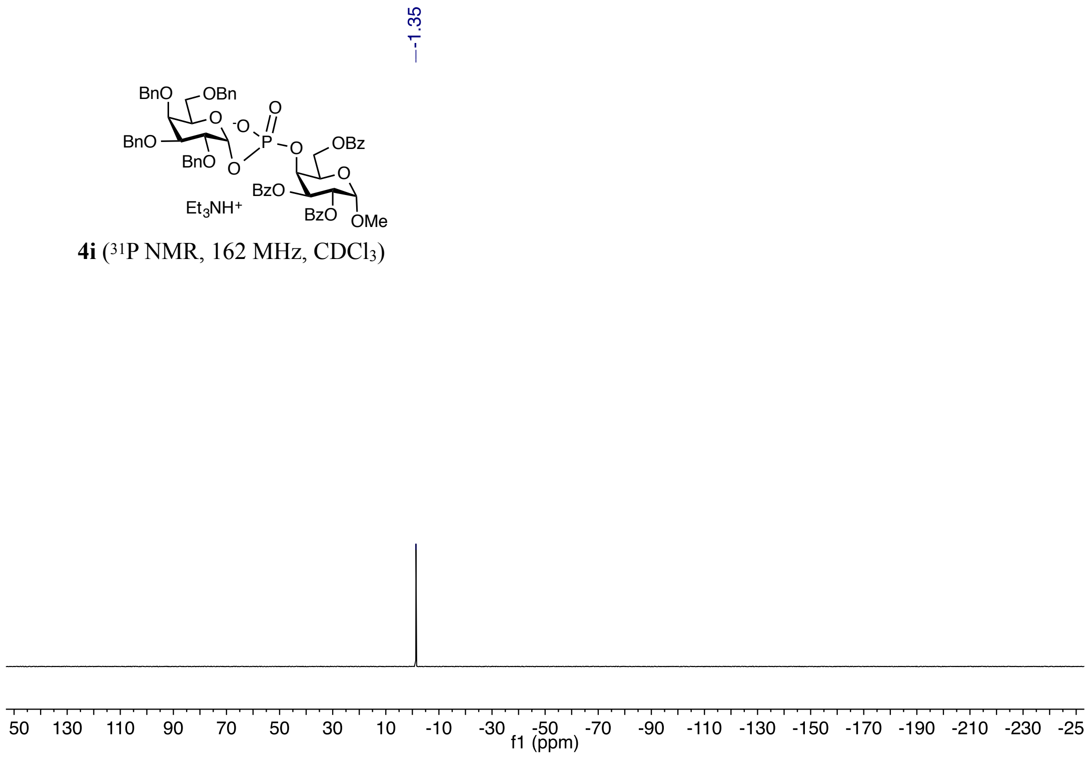

Supplementary Figure 93. ^{1}H NMR spectrum of compound **4j**

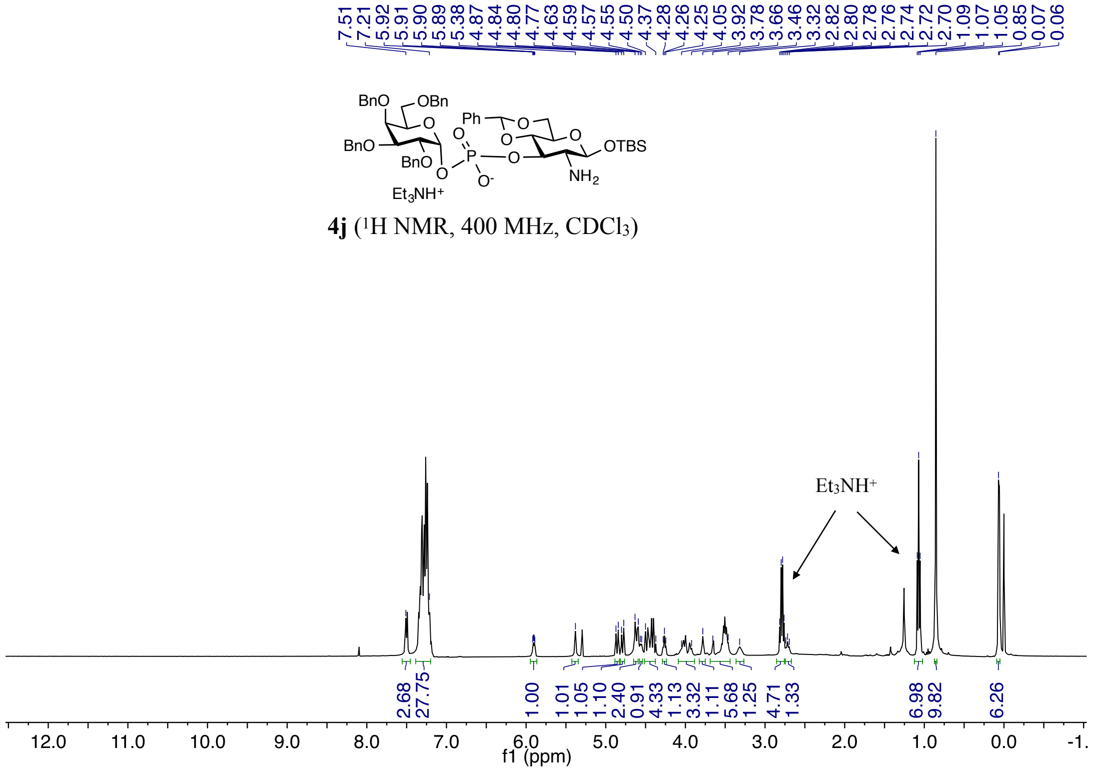

Supplementary Figure 94. ^{13}C NMR spectrum of compound **4j**

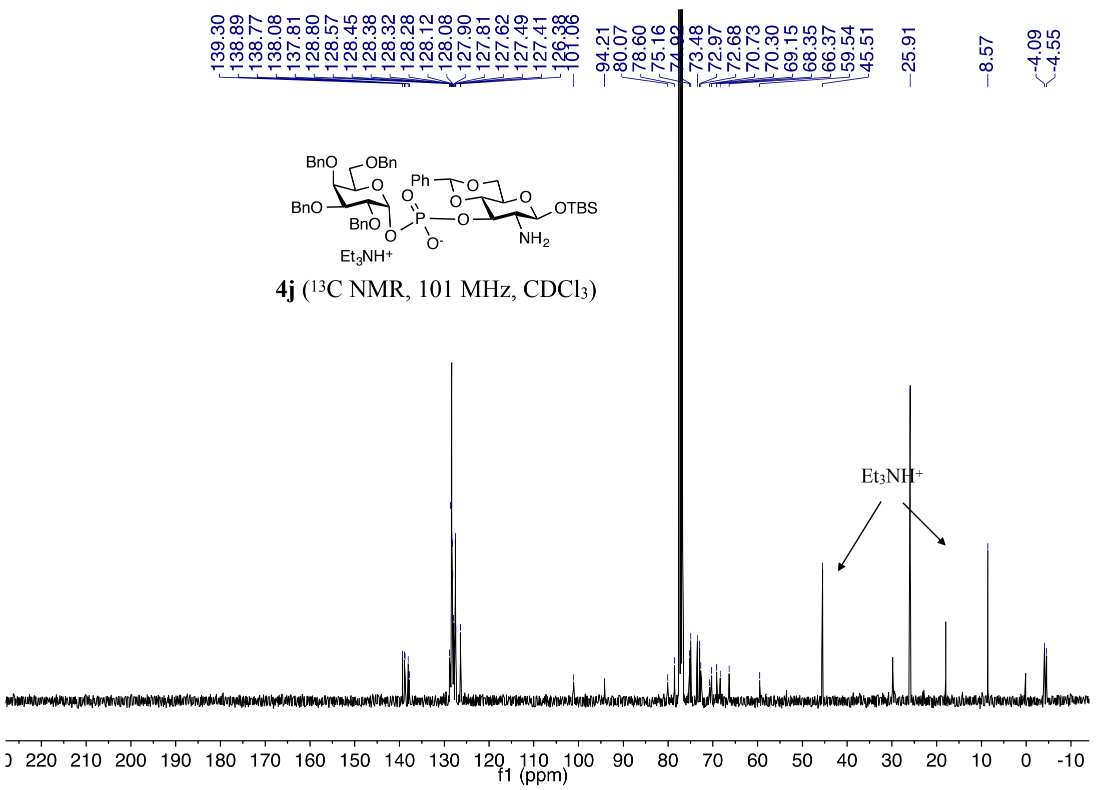

Supplementary Figure 95. ^{31}P NMR spectrum of compound **4j**

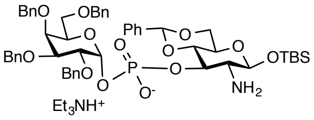

4j (^{31}P NMR, 162 MHz, $CDCl_3$)

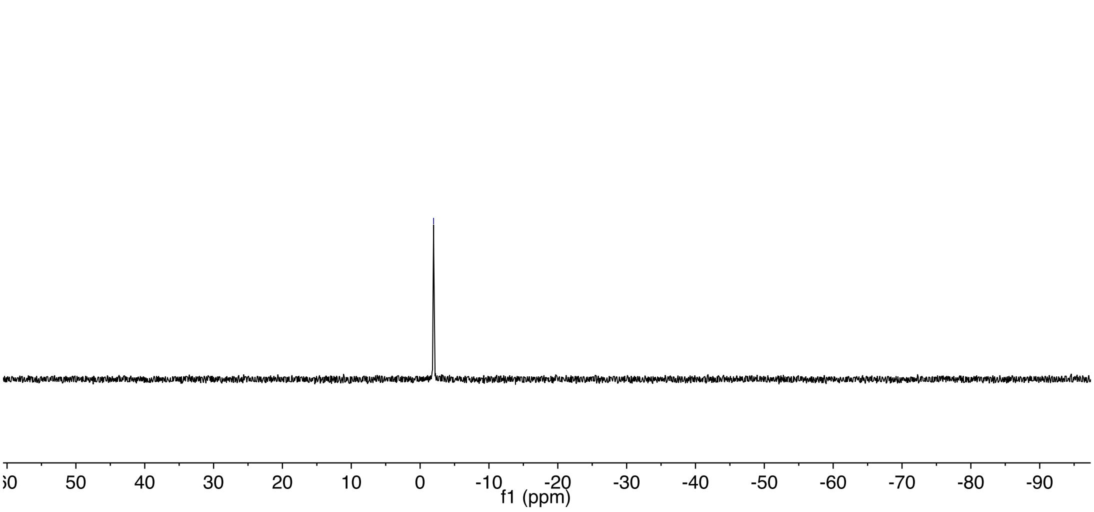

Supplementary Figure 96. ^{1}H NMR spectrum of compound **4k**

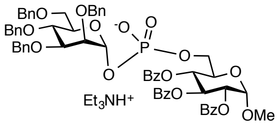

4k (^{1}H NMR, 400 MHz, $CDCl_3$)

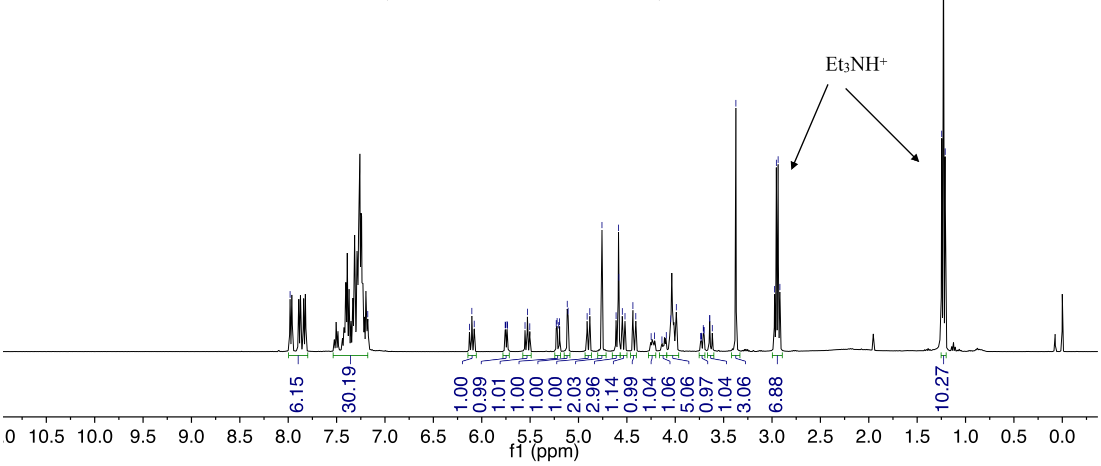

Supplementary Figure 97. ^{13}C NMR spectrum of compound **4k**

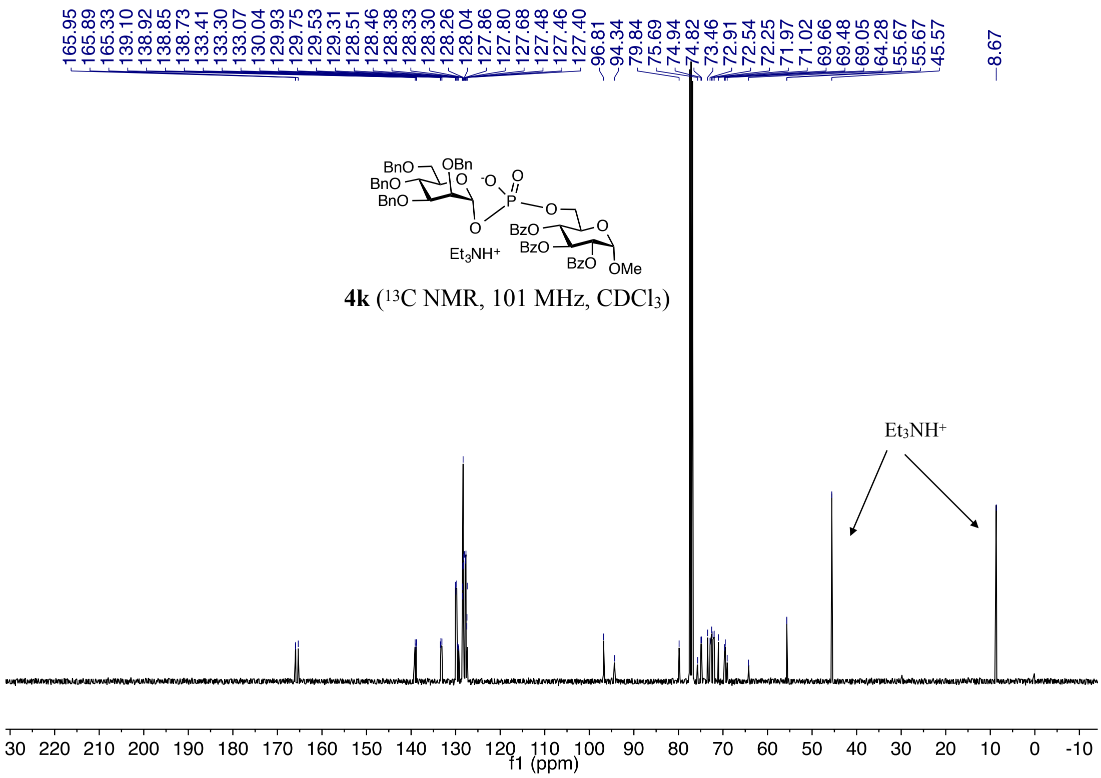

Supplementary Figure 98. ^{31}P NMR spectrum of compound **4k**

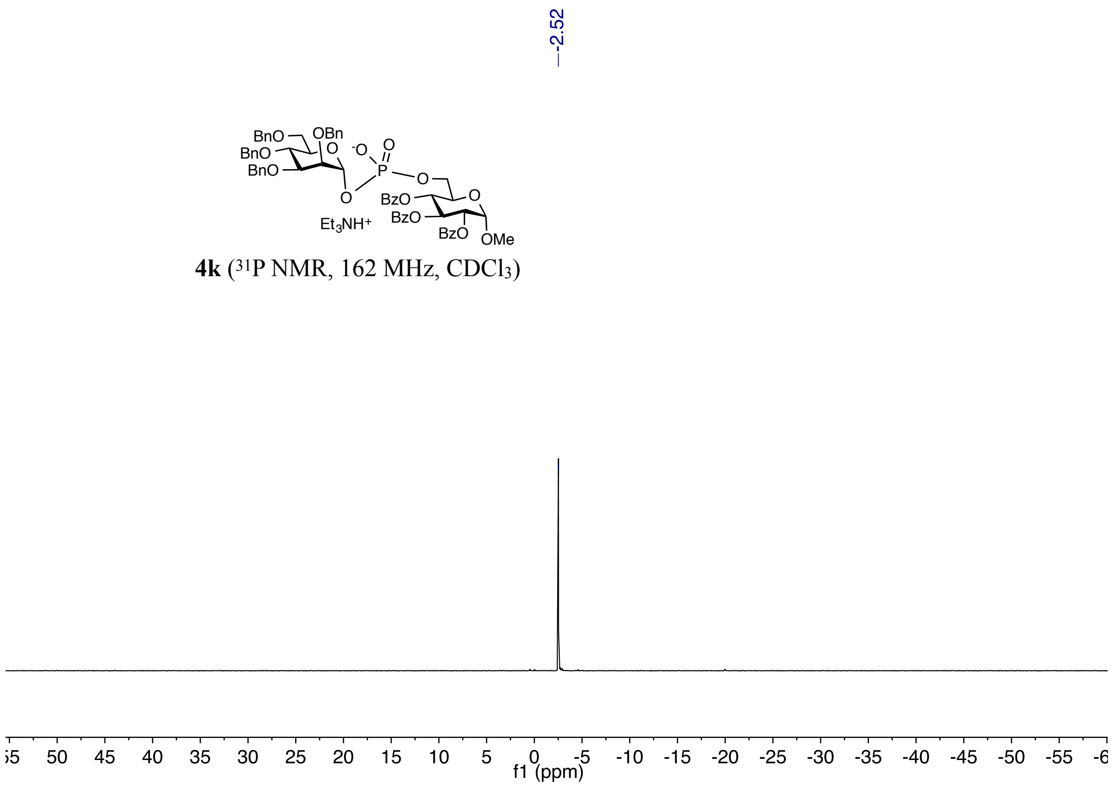

Supplementary Figure 99. ^{1}H NMR spectrum of compound **4l**

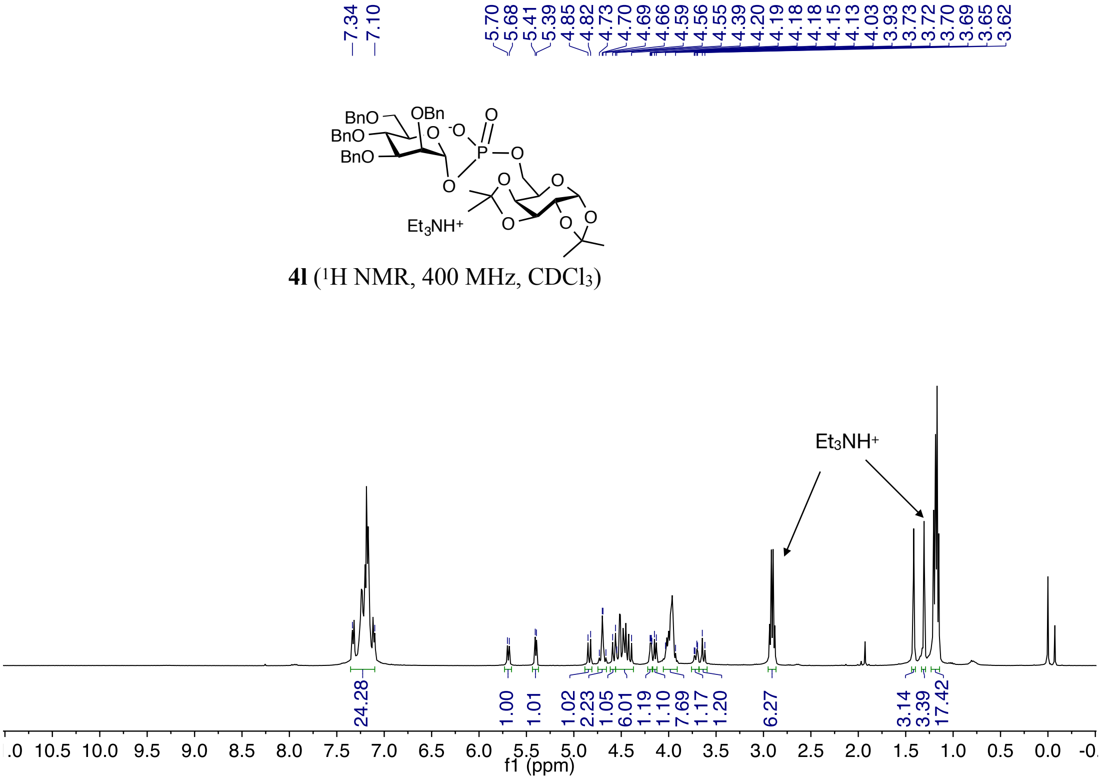

Supplementary Figure 100. ^{13}C NMR spectrum of compound **4l**

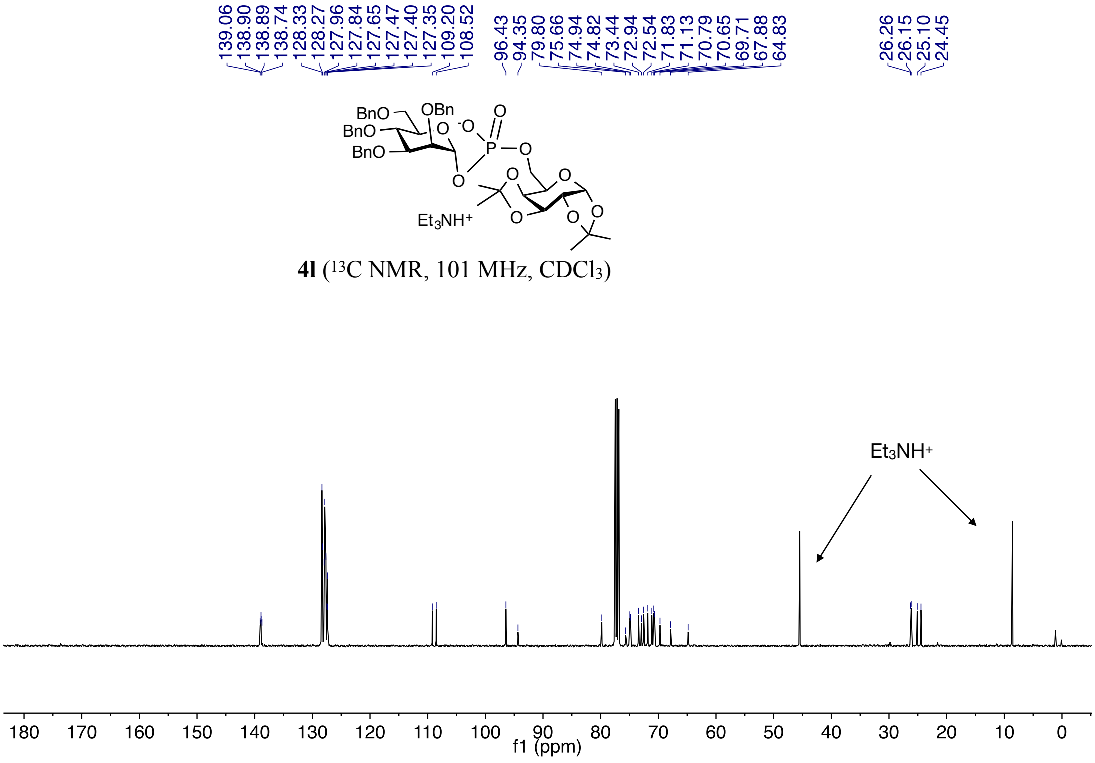

Supplementary Figure 101. ^{31}P NMR spectrum of compound **4l**

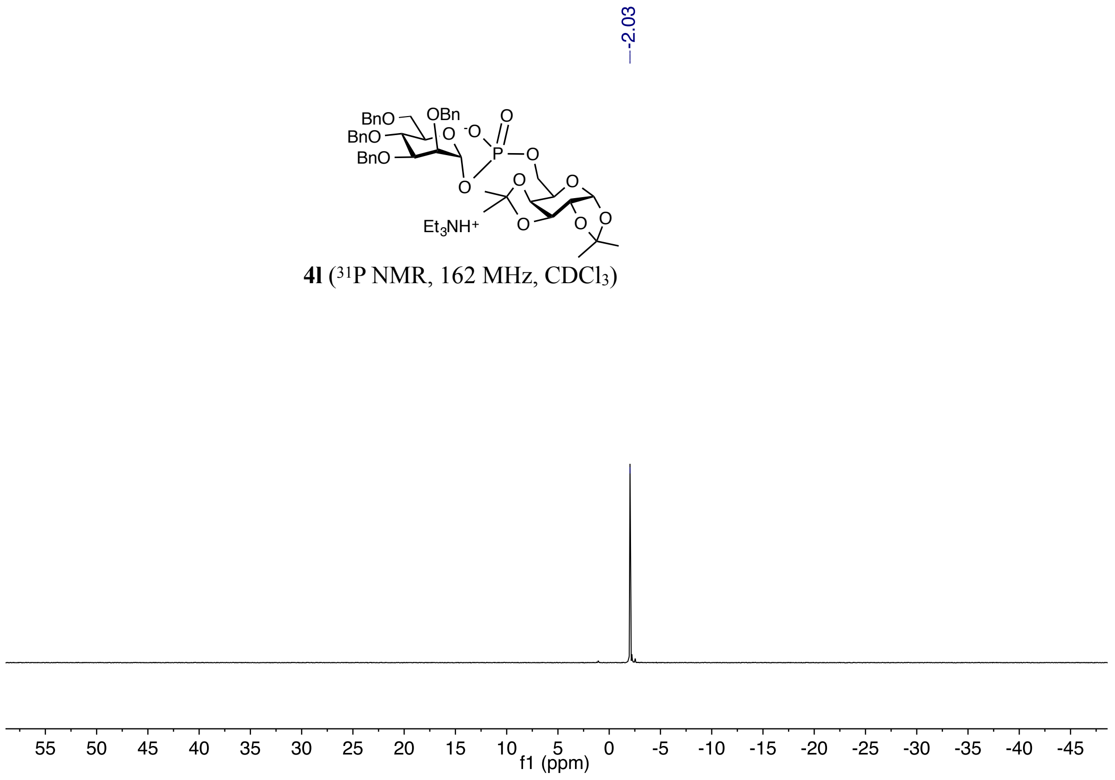

Supplementary Figure 102. ^{1}H NMR spectrum of compound **4m**

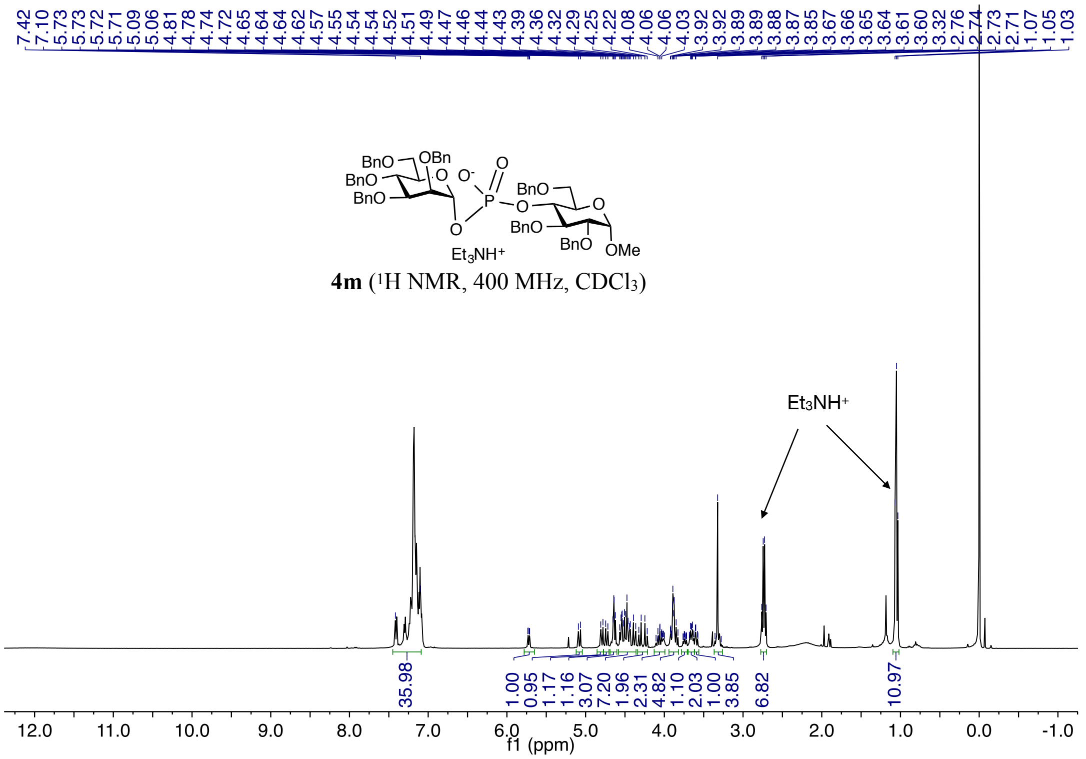

Supplementary Figure 103. ^{13}C NMR spectrum of compound **4m**

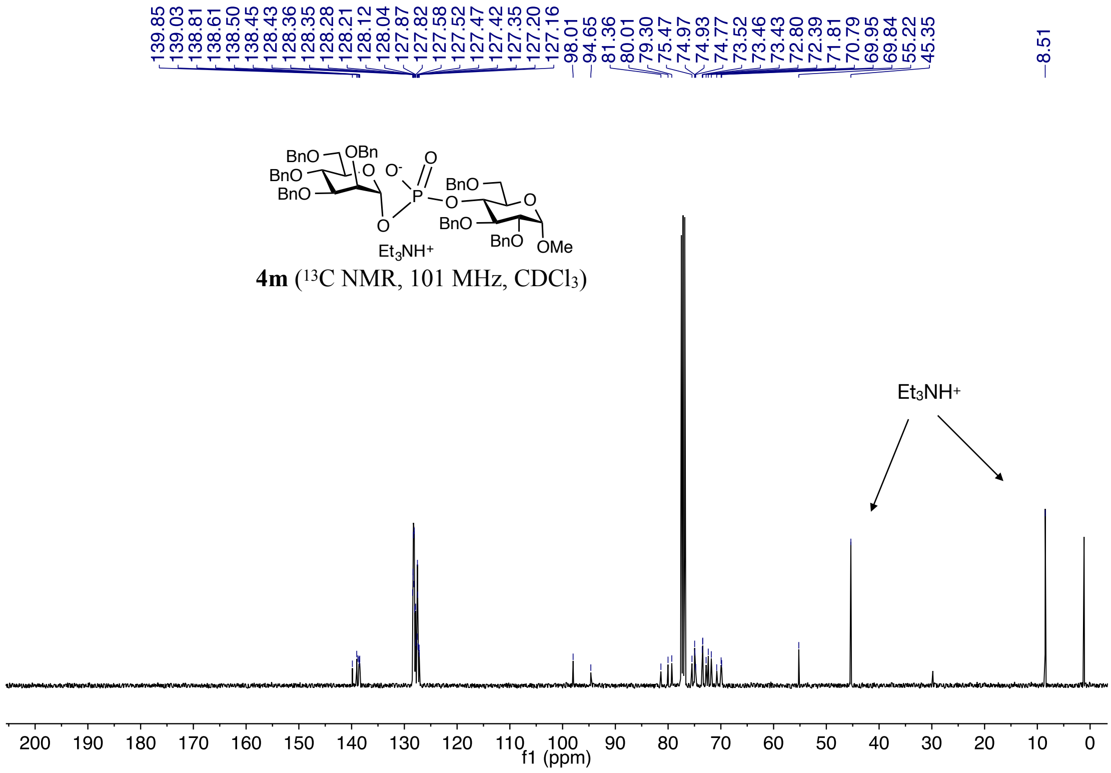

Supplementary Figure 104. ^{31}P NMR spectrum of compound **4m**

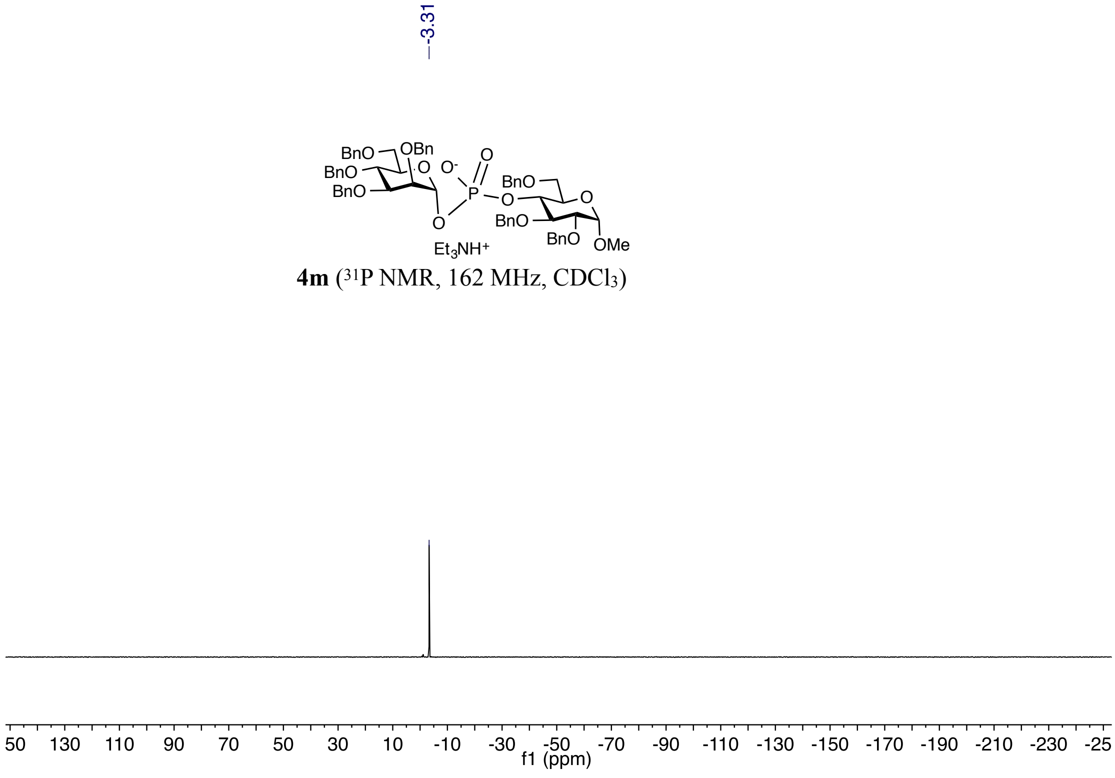

Supplementary Figure 105. ^{1}H NMR spectrum of compound **4n**

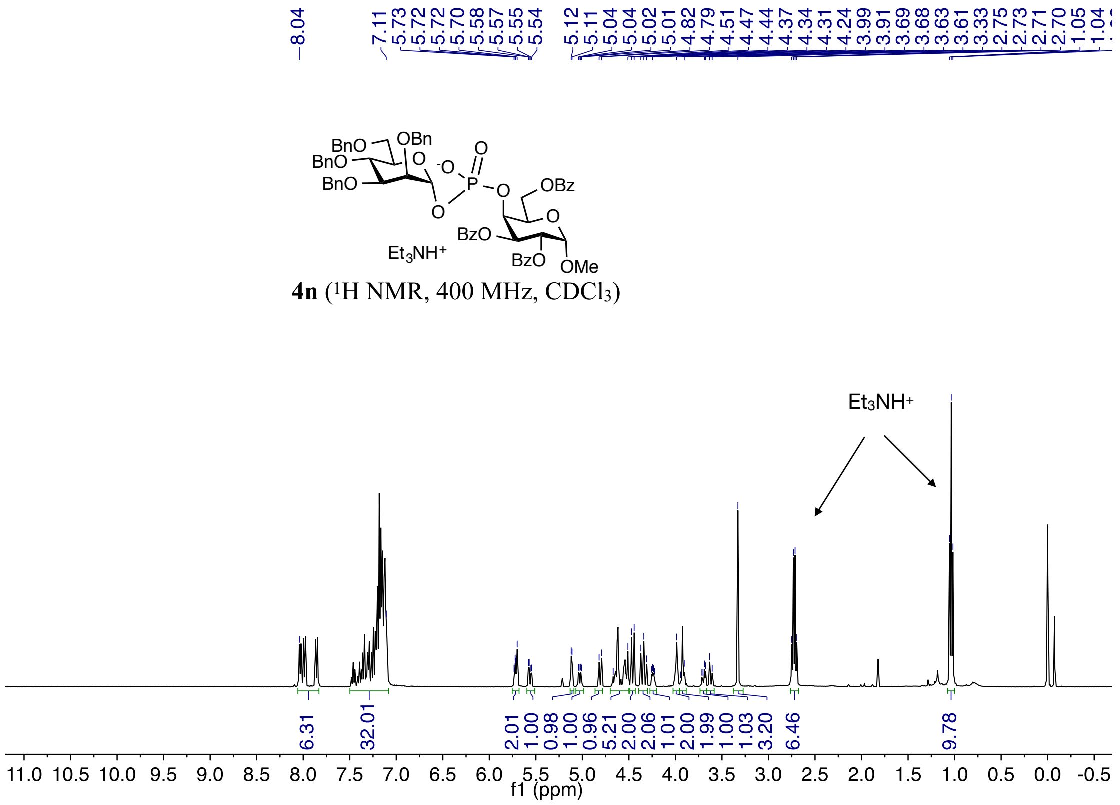

Supplementary Figure 106. ^{13}C NMR spectrum of compound **4n**

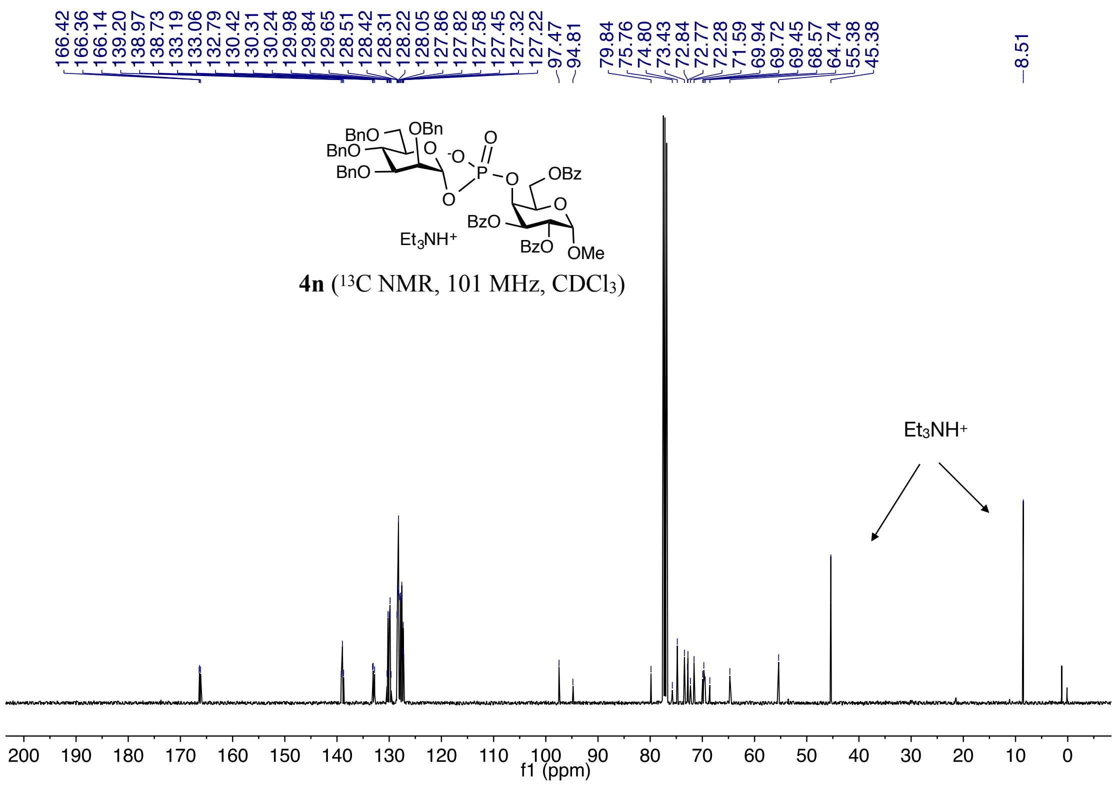

Supplementary Figure 107. ^{31}P NMR spectrum of compound **4n**

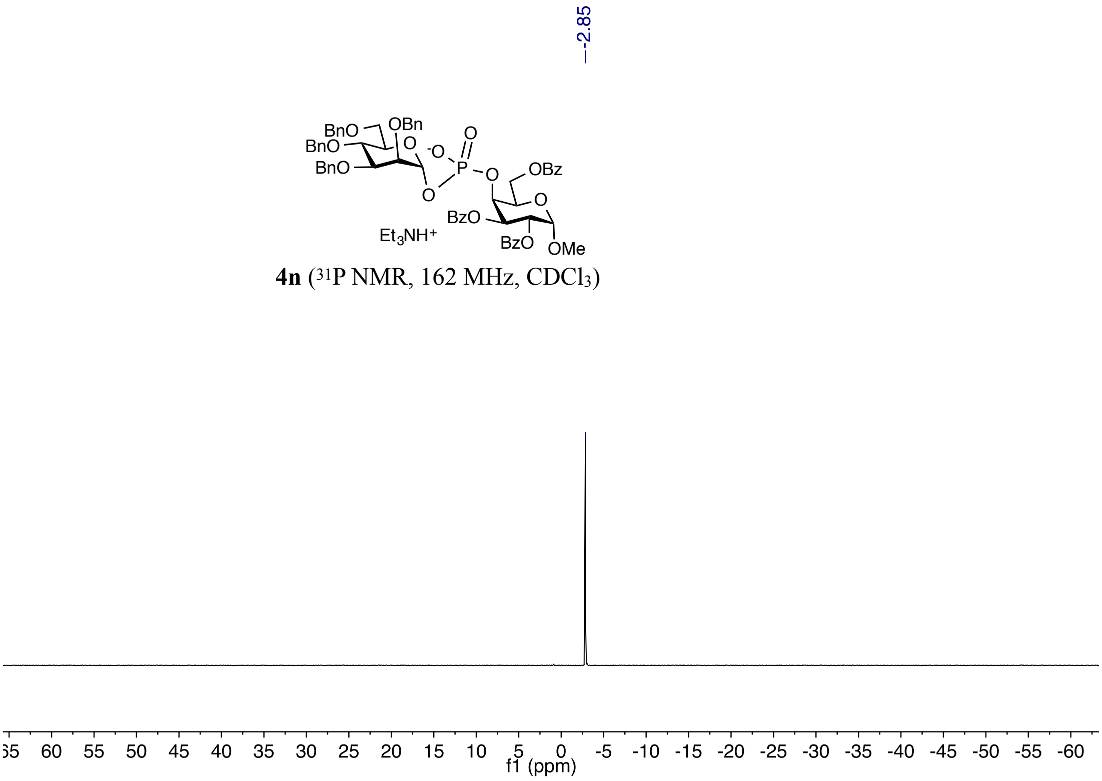

Supplementary Figure 108. ^{1}H NMR spectrum of compound **4o**

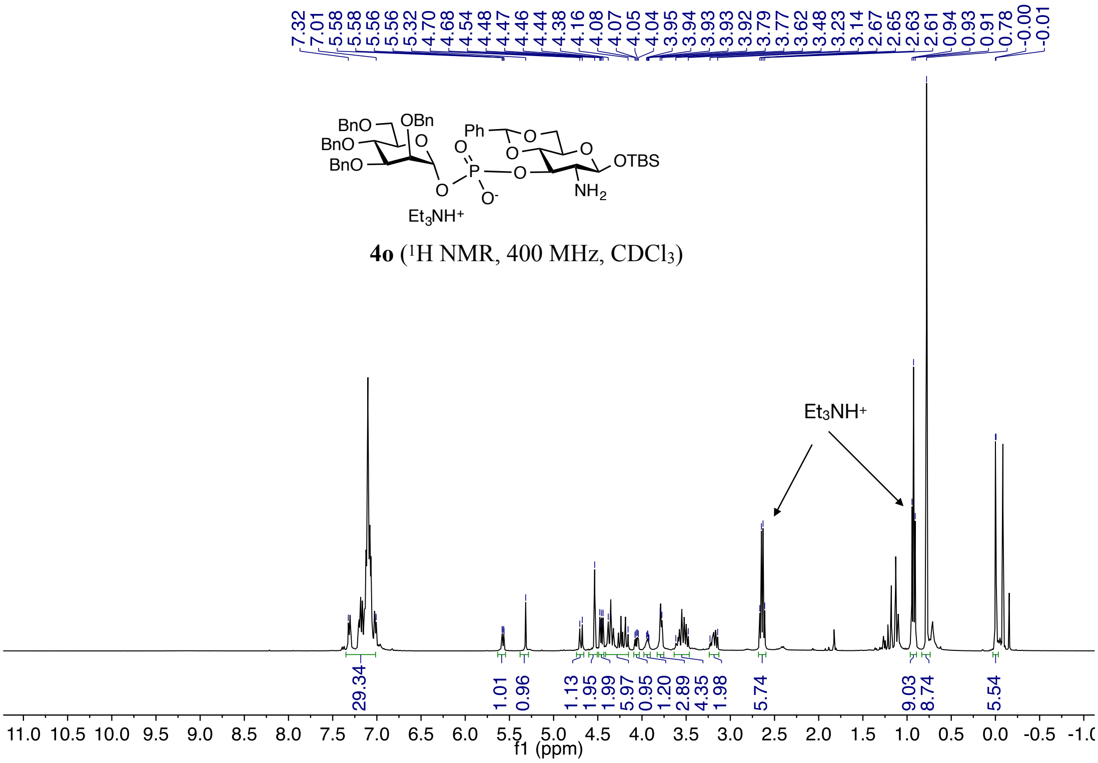

Supplementary Figure 109. ^{13}C NMR spectrum of compound **4o**

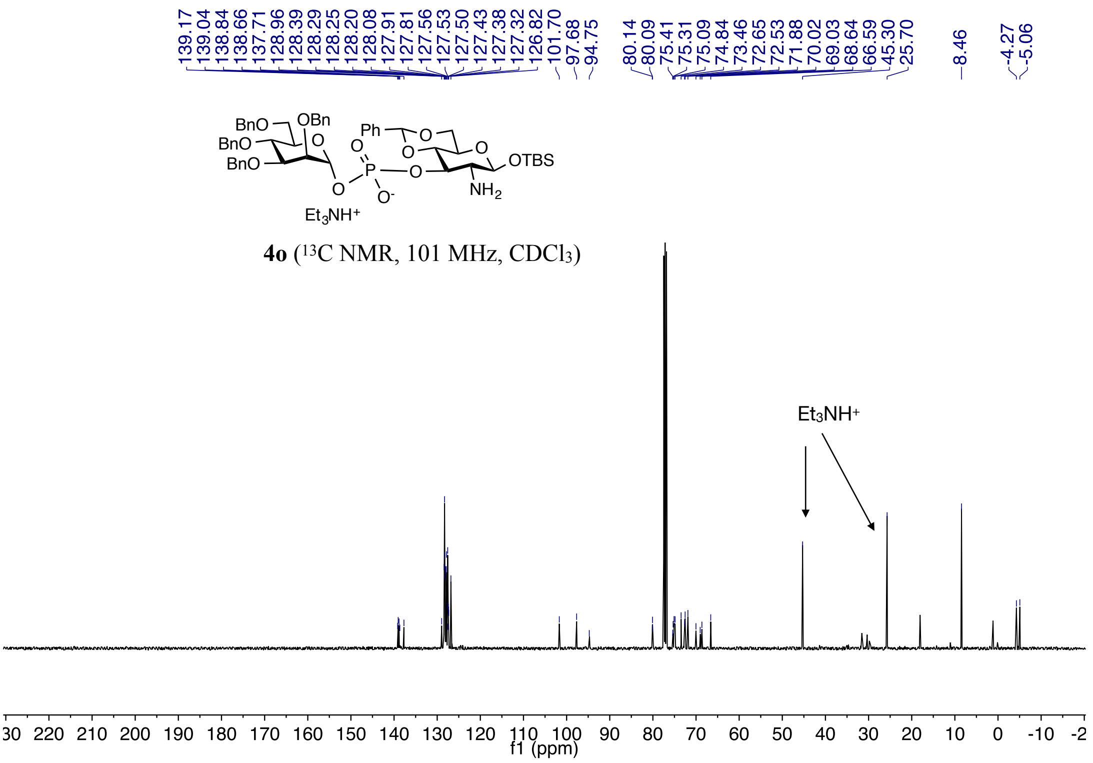

Supplementary Figure 110. ^{31}P NMR spectrum of compound **4o**

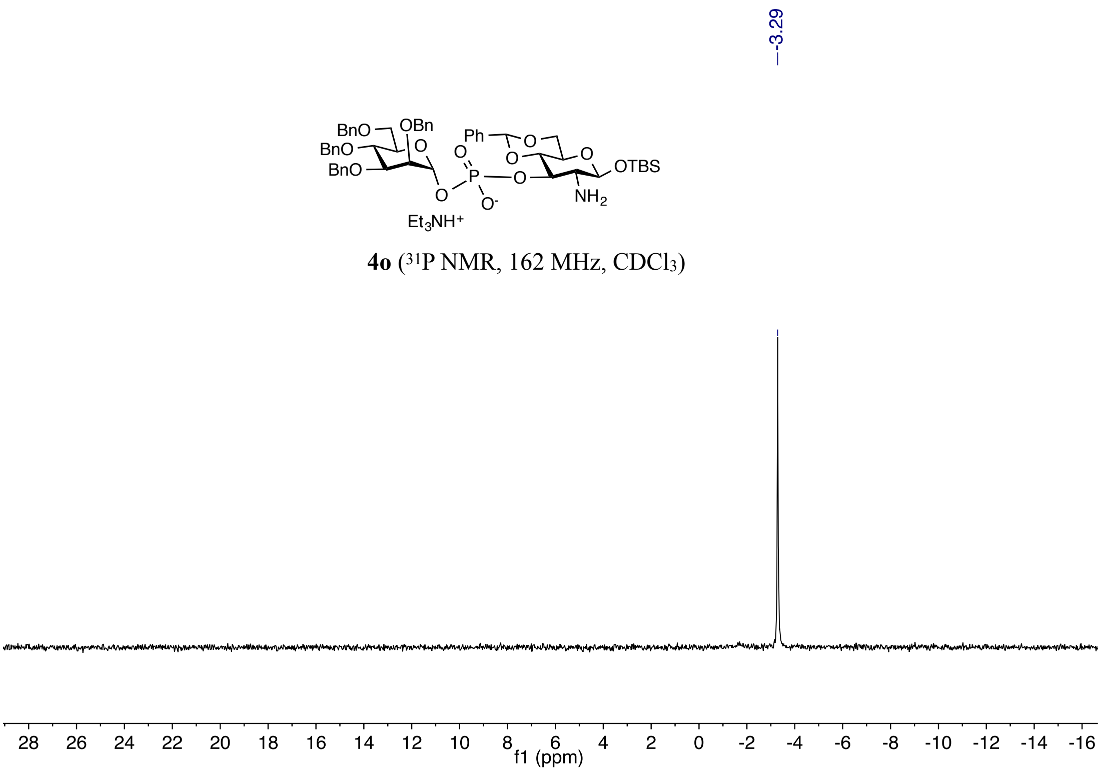

Supplementary Figure 111. ^{1}H NMR spectrum of compound **4p**

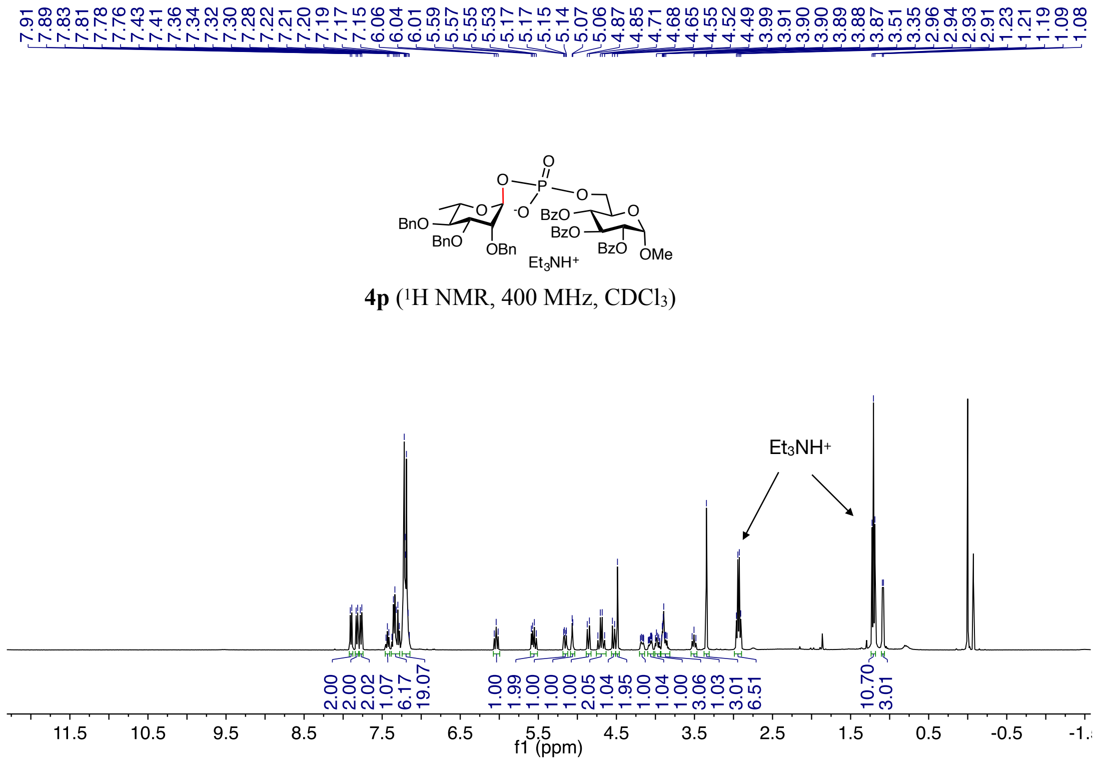

Supplementary Figure 112. ^{13}C NMR spectrum of compound **4p**

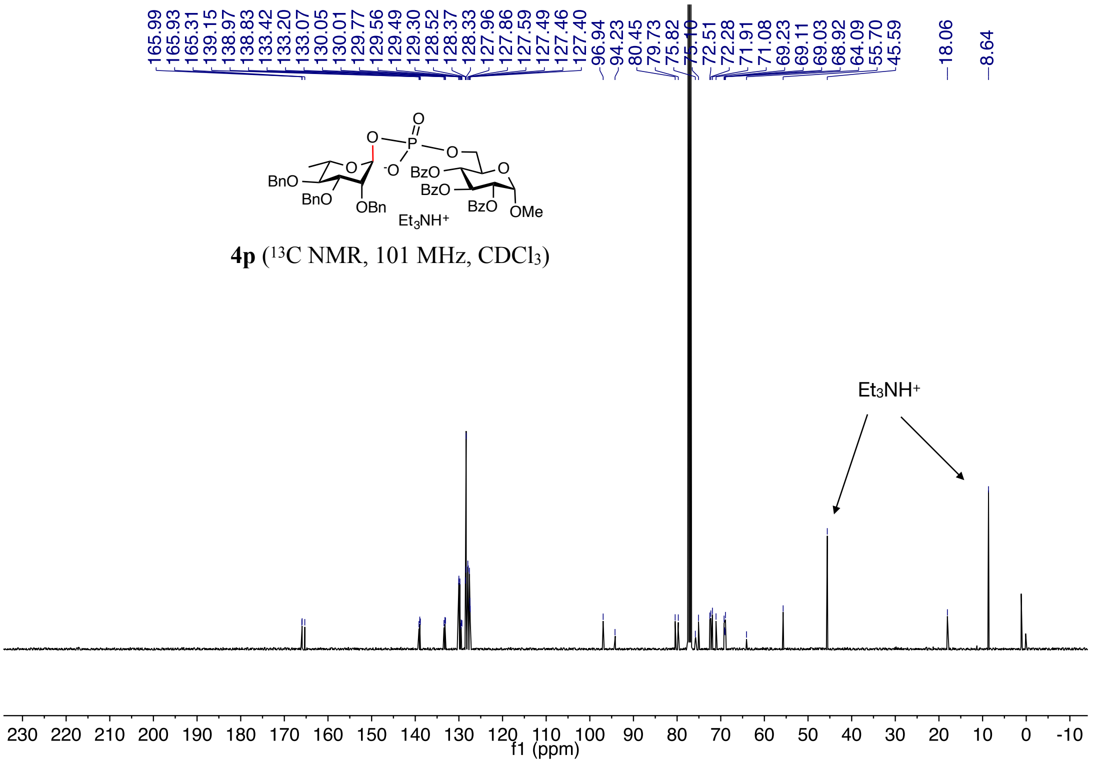

Supplementary Figure 113. ^{31}P NMR spectrum of compound **4p**

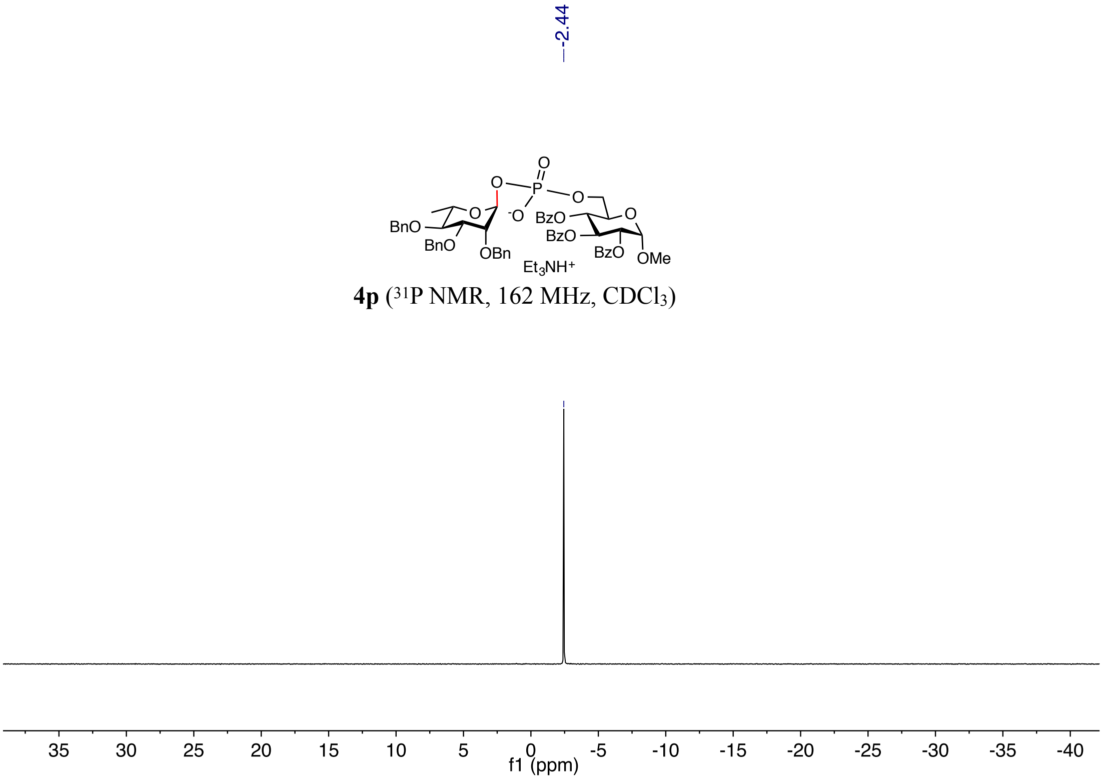

Supplementary Figure 114. ^{1}H NMR spectrum of compound **4q**

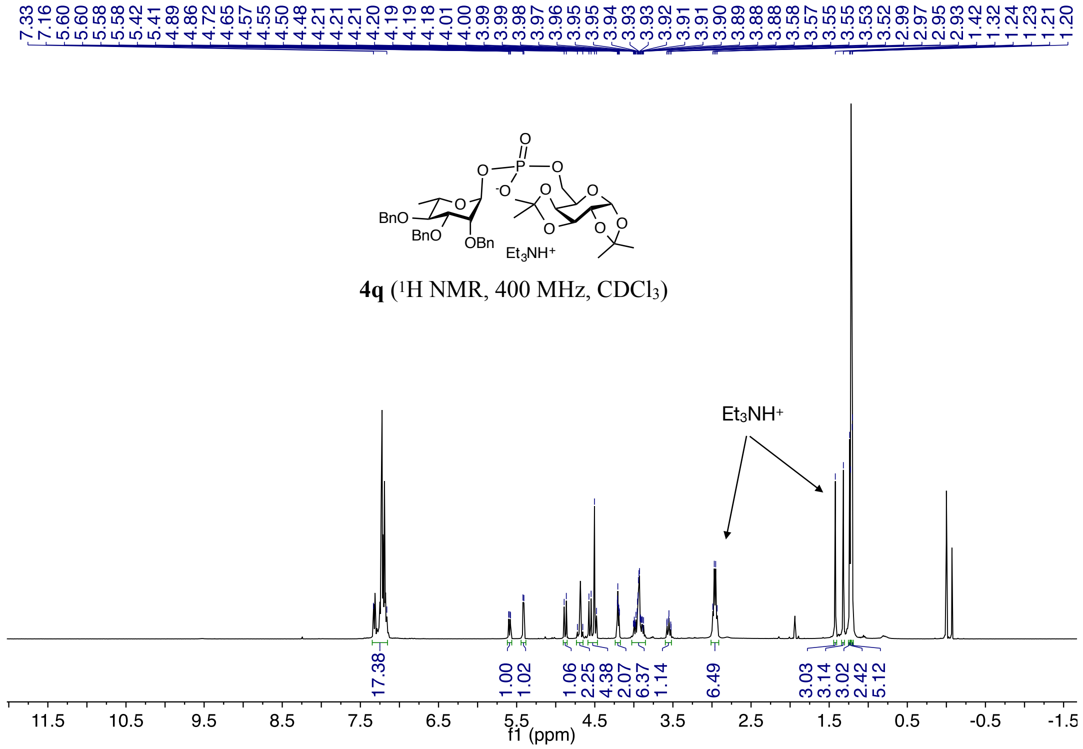

Supplementary Figure 115. ^{13}C NMR spectrum of compound **4q**

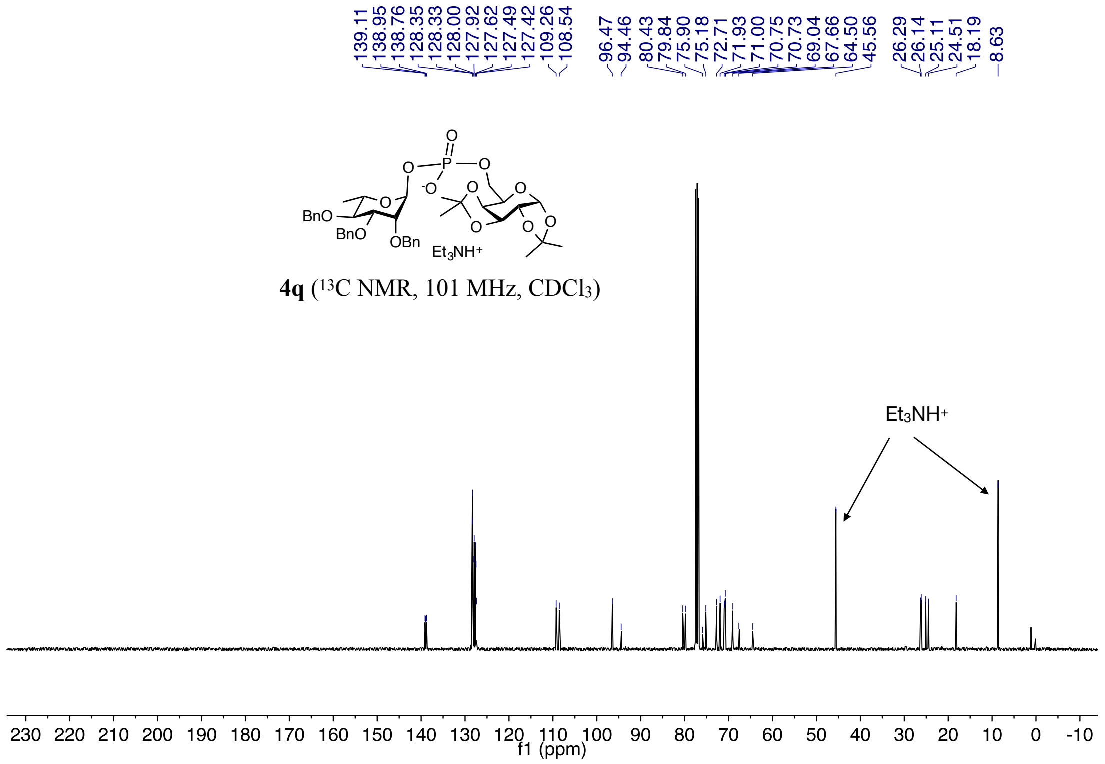

Supplementary Figure 116. ^{31}P NMR spectrum of compound **4q**

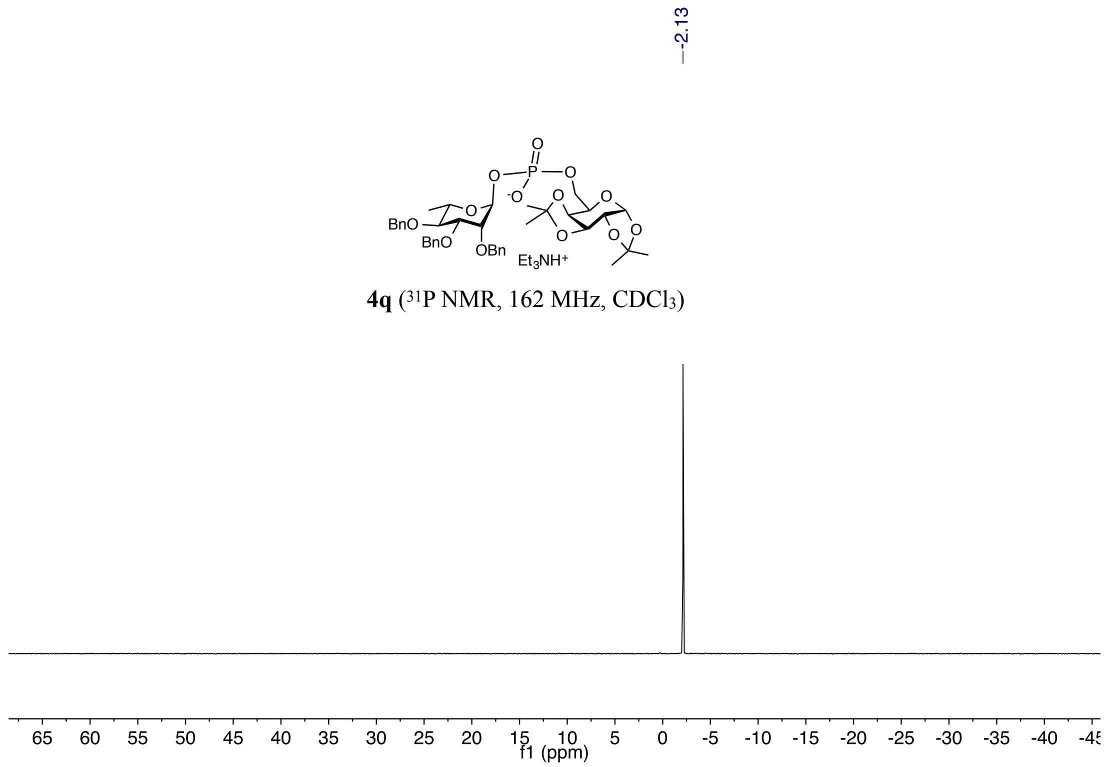

Supplementary Figure 117. ^{1}H NMR spectrum of compound **4r**

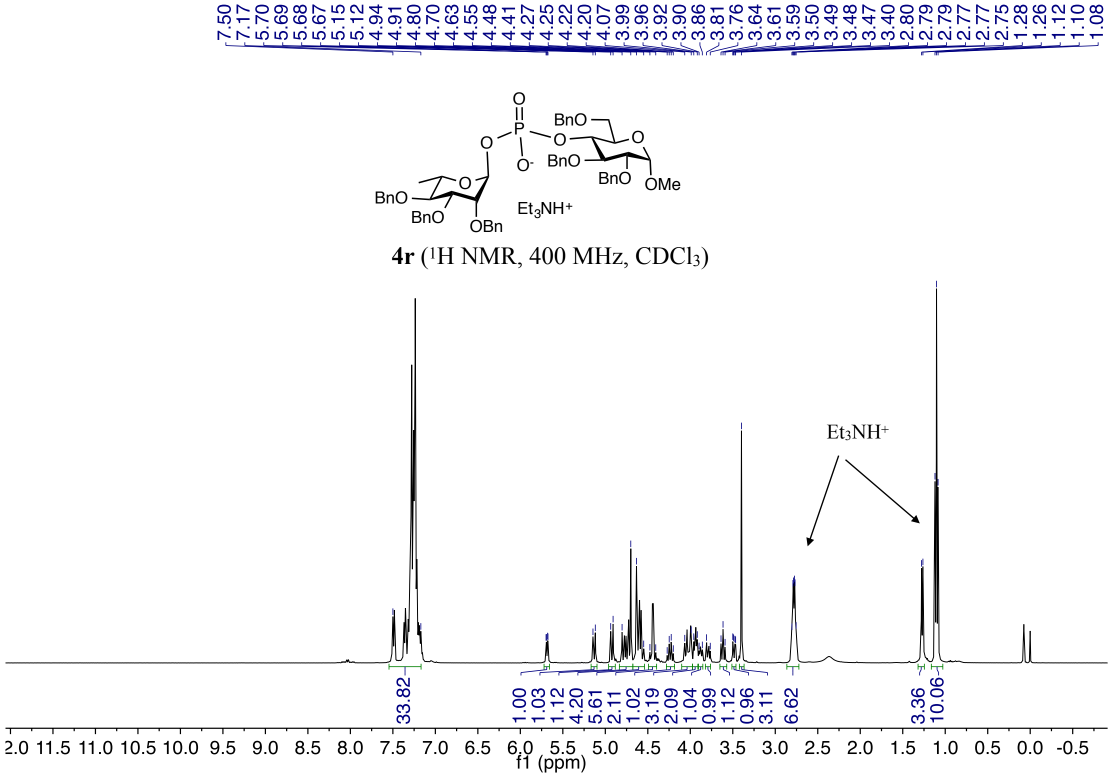

Supplementary Figure 118. ^{13}C NMR spectrum of compound **4r**

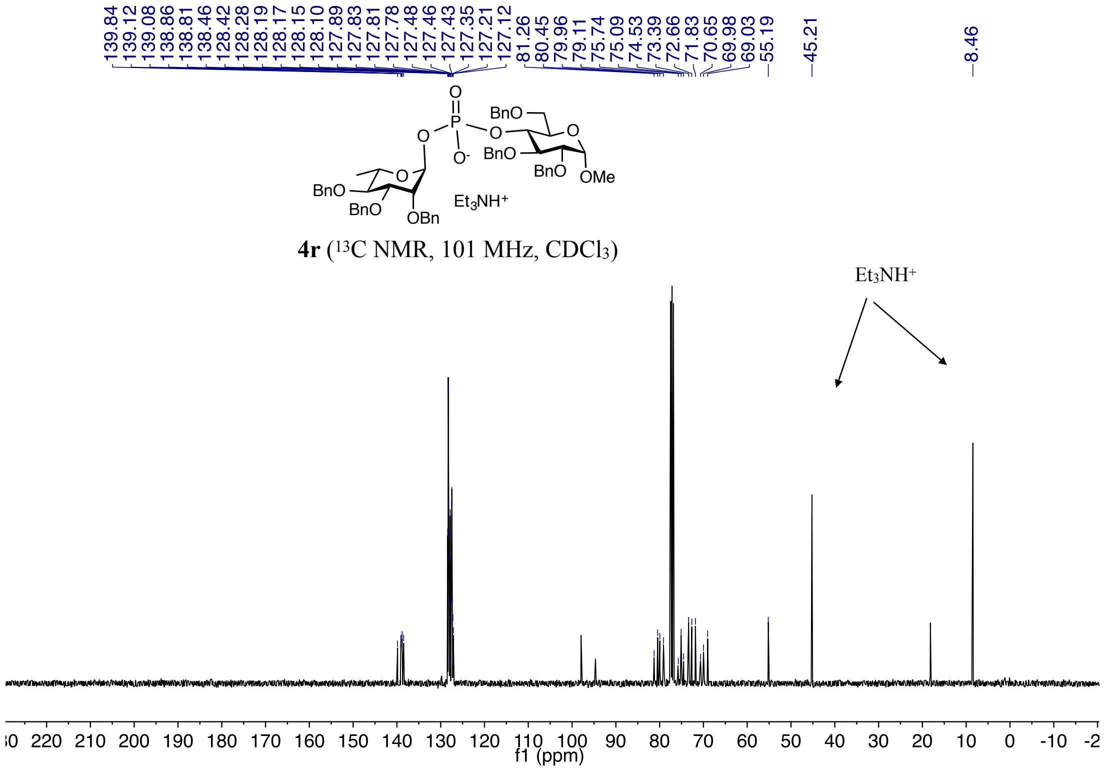

Supplementary Figure 119. ^{31}P NMR spectrum of compound **4r**

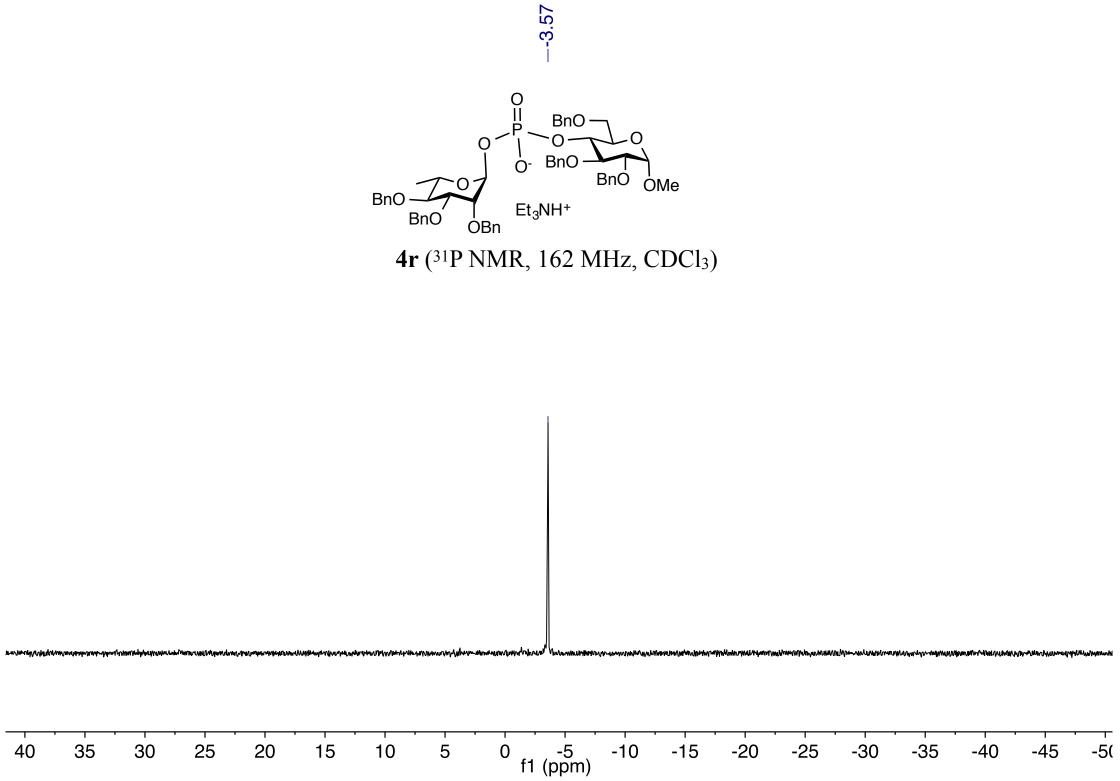

Supplementary Figure 120. ^{1}H NMR spectrum of compound **4s**

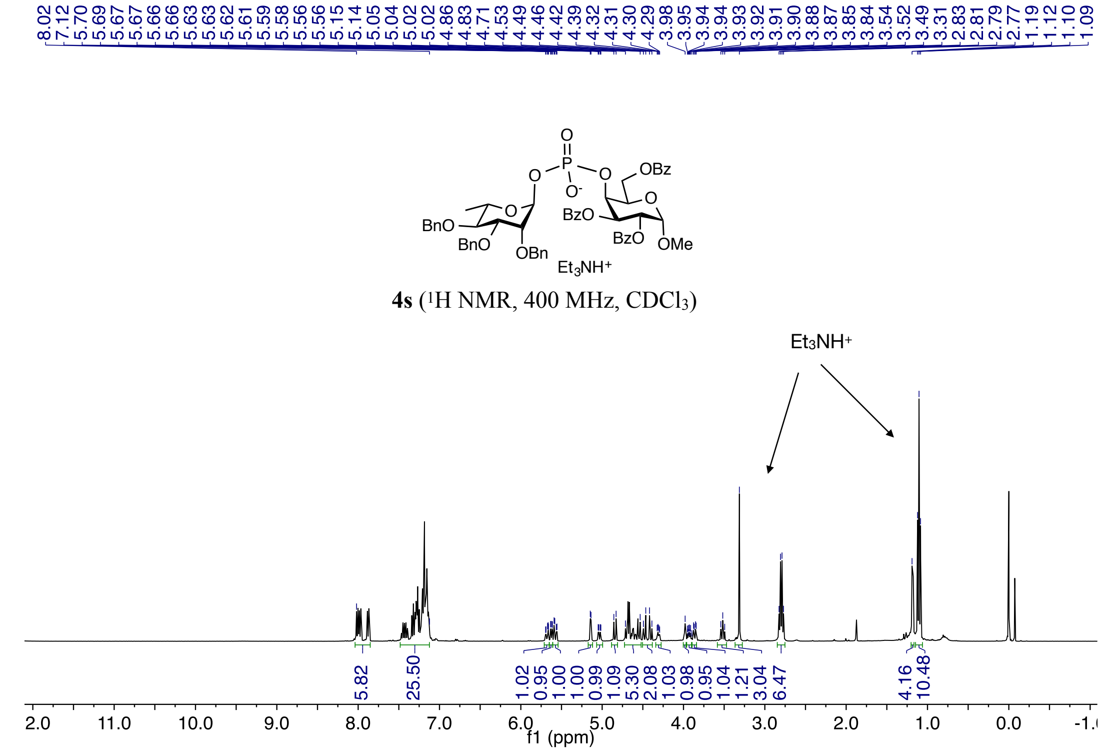

Supplementary Figure 121. ^{13}C NMR spectrum of compound **4s**

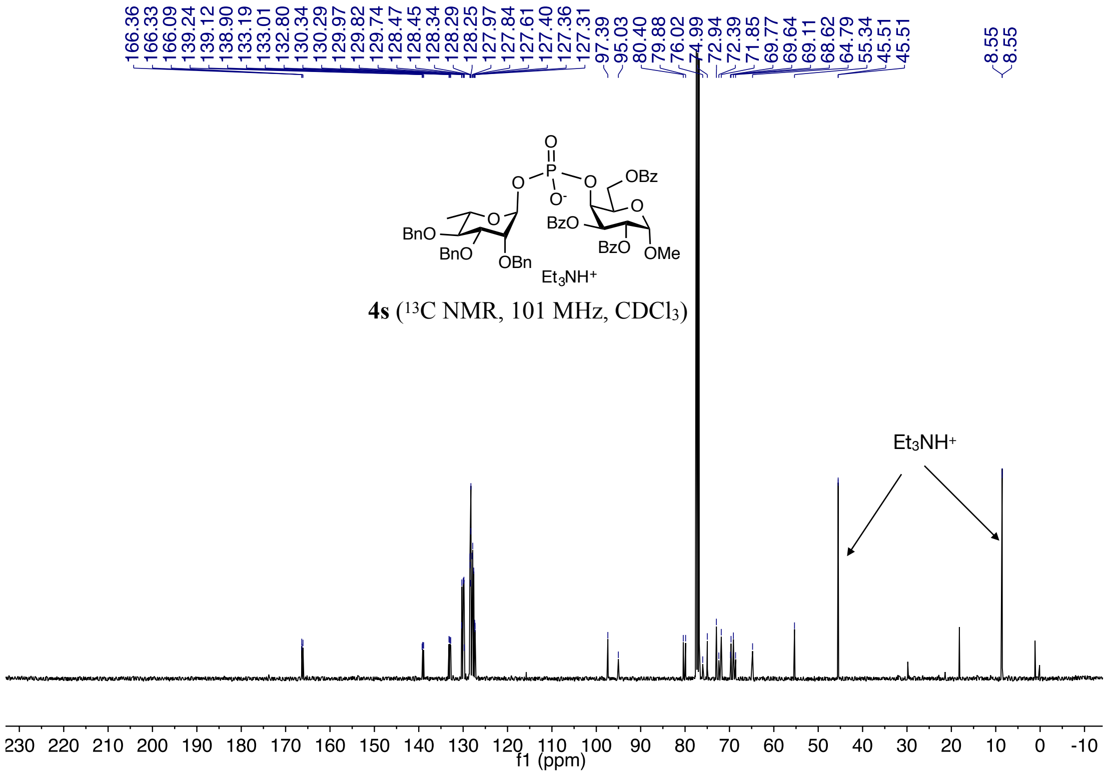

Supplementary Figure 122. ^{31}P NMR spectrum of compound **4s**

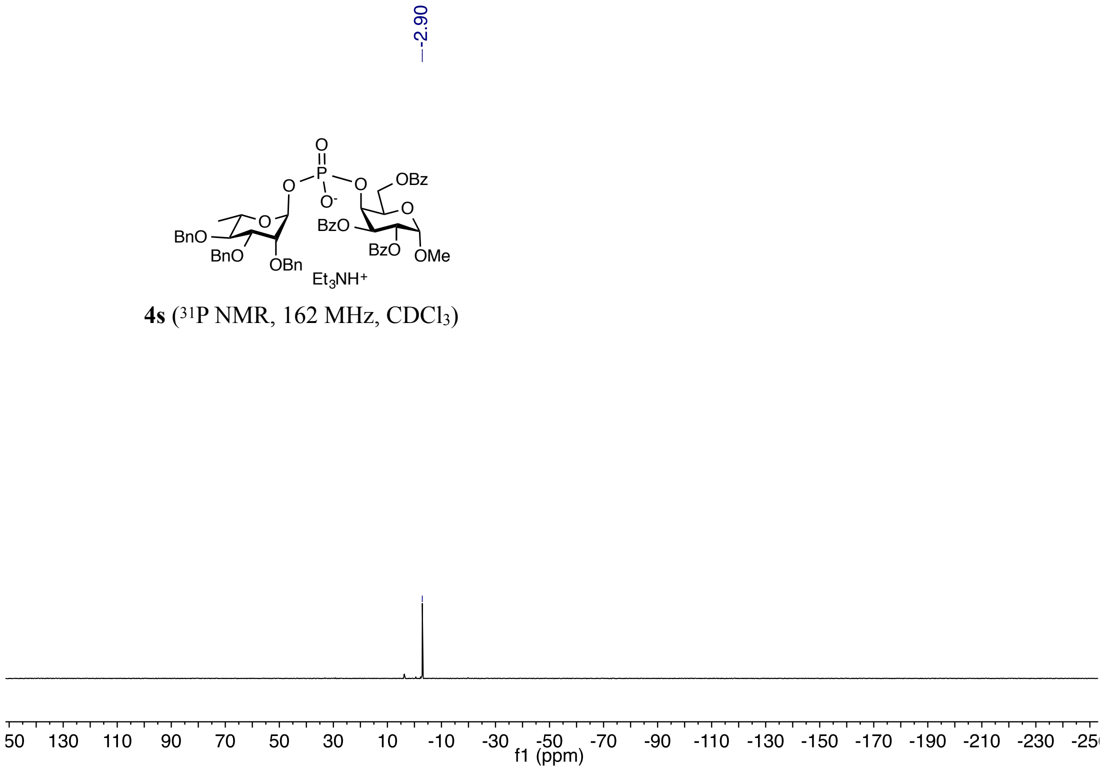

Supplementary Figure 123. ^{1}H NMR spectrum of compound **4t**

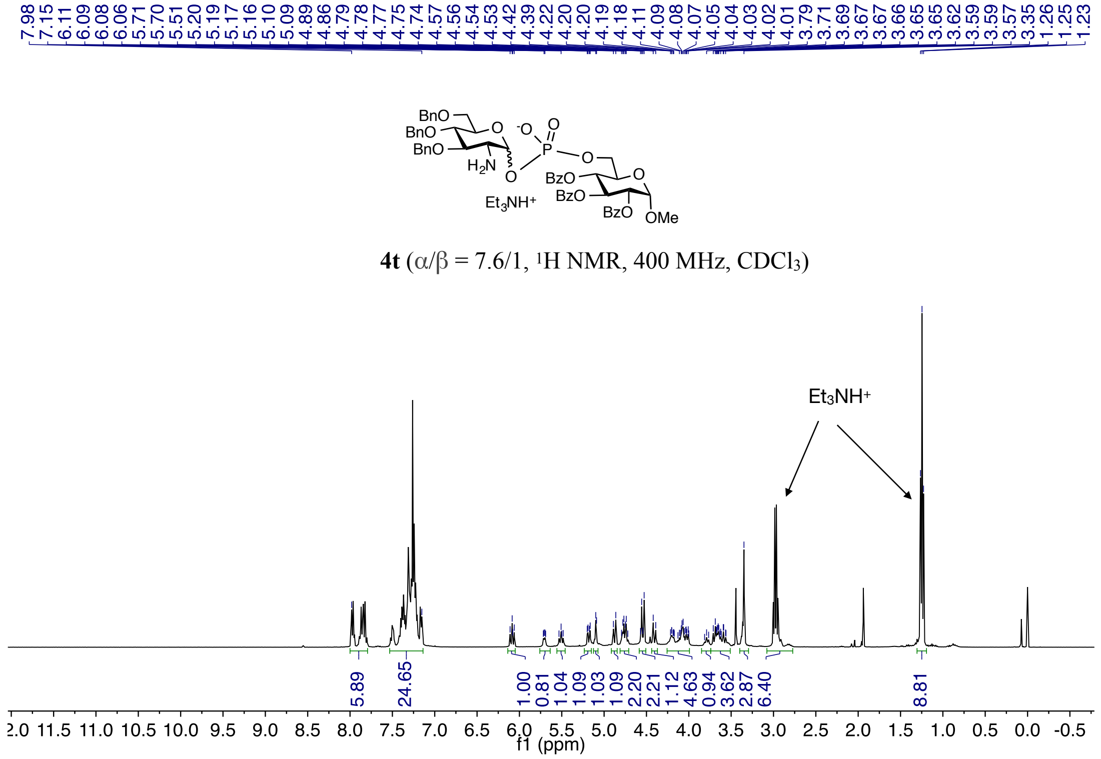

Supplementary Figure 124. ^{13}C NMR spectrum of compound **4t**

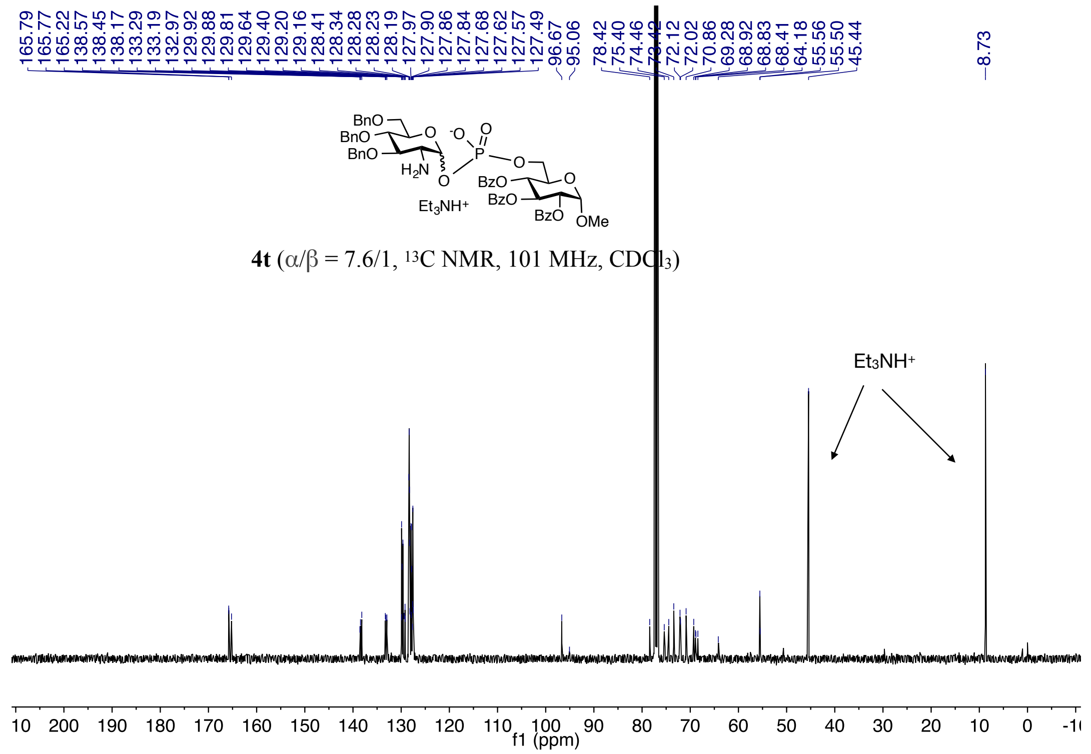

Supplementary Figure 125. ^{31}P NMR spectrum of compound **4t**

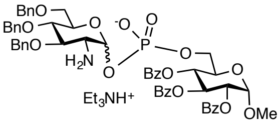

4t (α/β = 7.6/1, ^{31}P NMR, 162 MHz, $CDCl_3$)

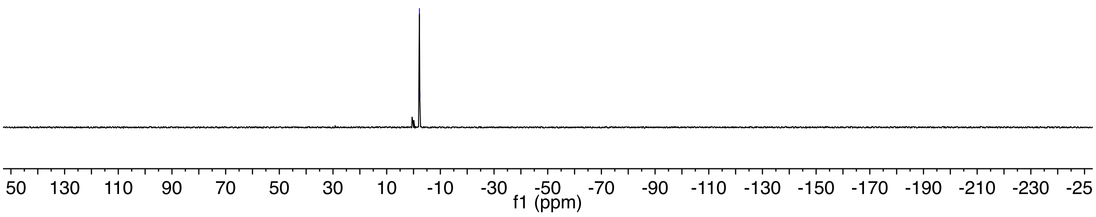

Supplementary Figure 126. ^{31}P NMR spectrum used to determined α/β ratio of **4t**

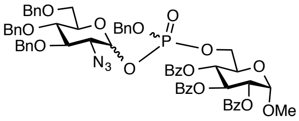

The α/β ratio was determined to be 7.6/1 by ^{31}P NMR

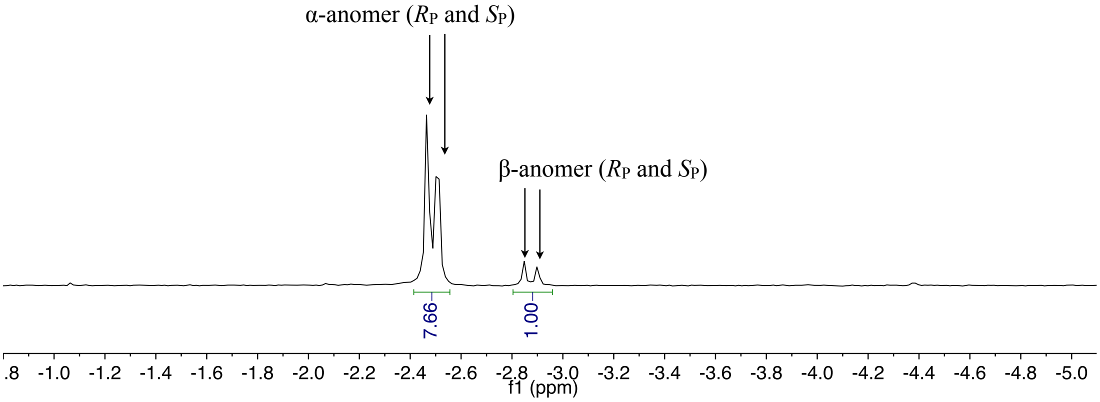

Supplementary Figure 127. ^{1}H NMR spectrum of compound **4u**

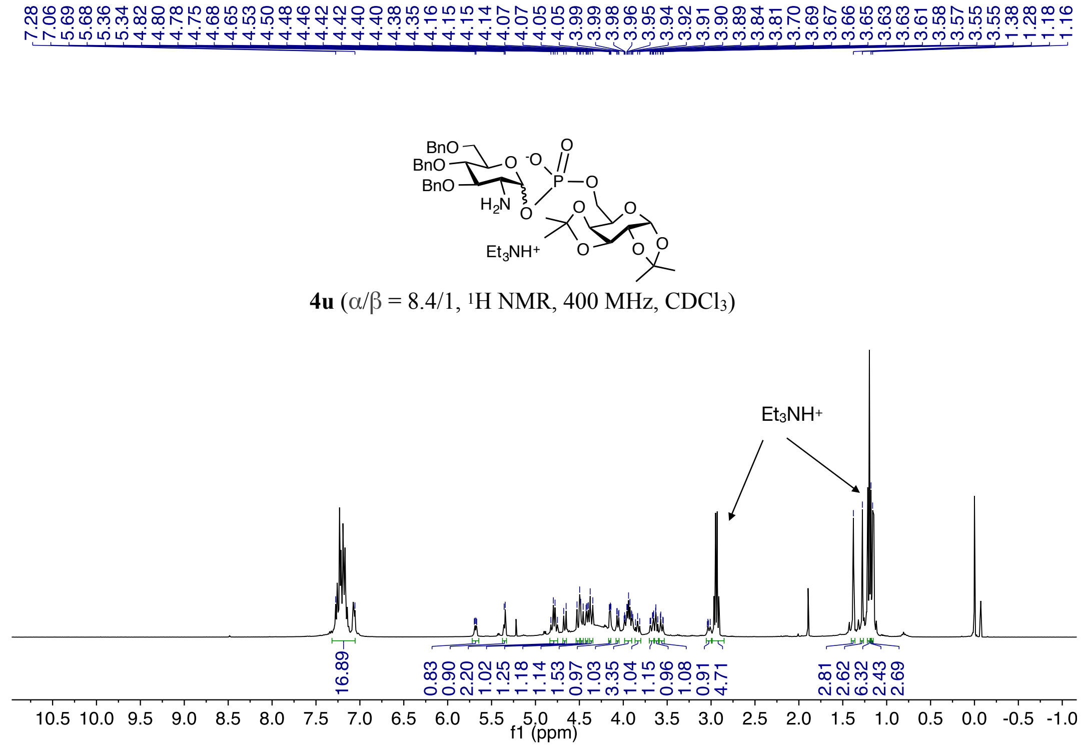

Supplementary Figure 128. ^{13}C NMR spectrum of compound **4u**

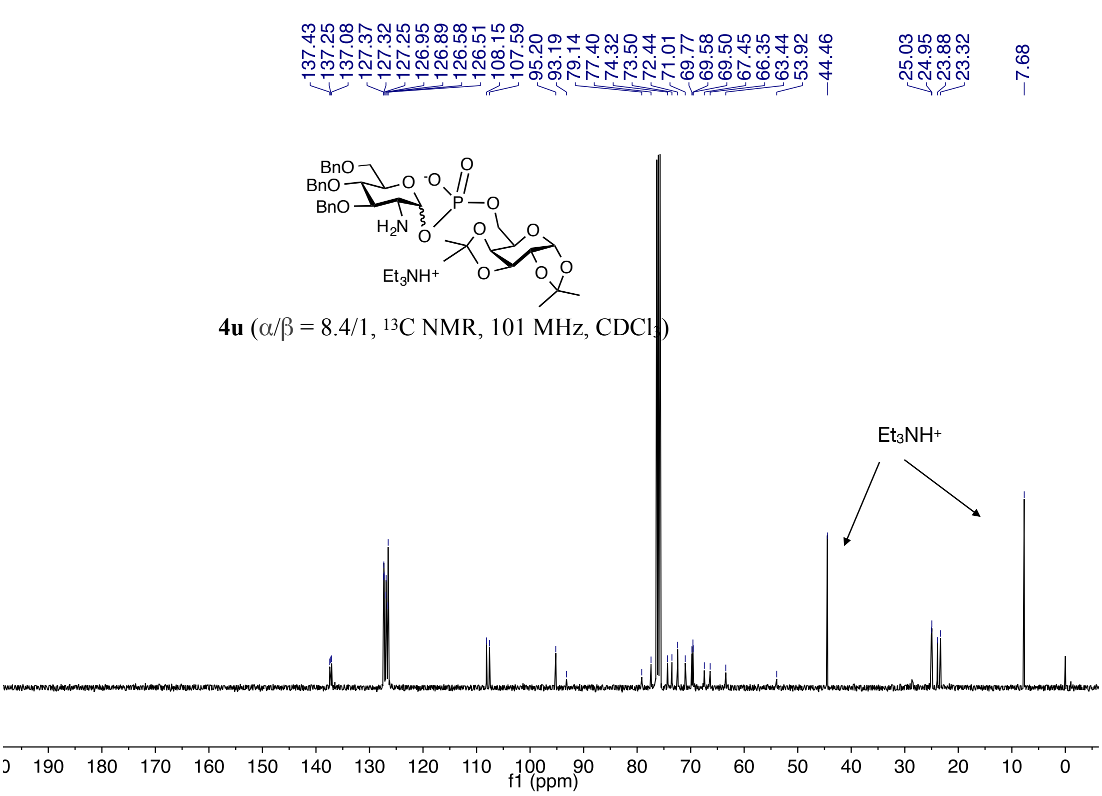

Supplementary Figure 129. ^{31}P NMR spectrum of compound **4u**

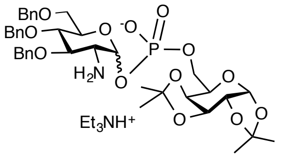

4u (α/β = 8.4/1, ^{31}P NMR, 162 MHz, $CDCl_3$)

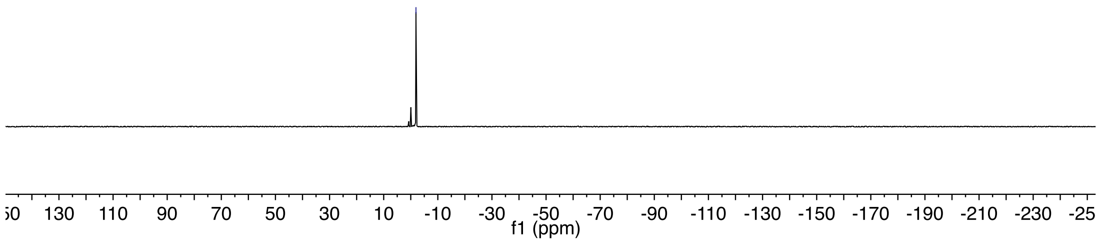

Supplementary Figure 130. ^{31}P NMR spectrum used to determined ratio of **4t**

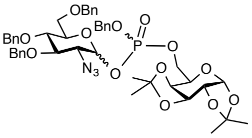

The α/β ratio was determined to be 8.4/1 by ^{31}P NMR

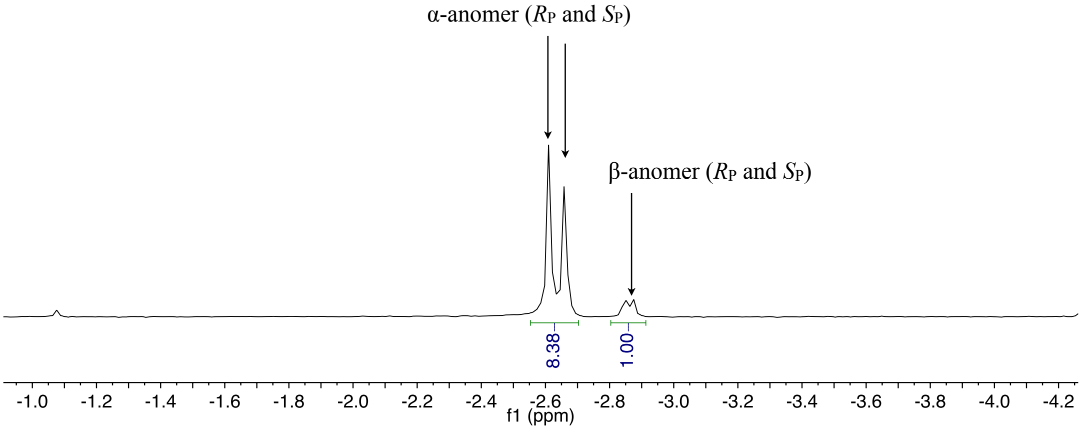

Supplementary Figure 131. ^{1}H NMR spectrum of compound **4v**

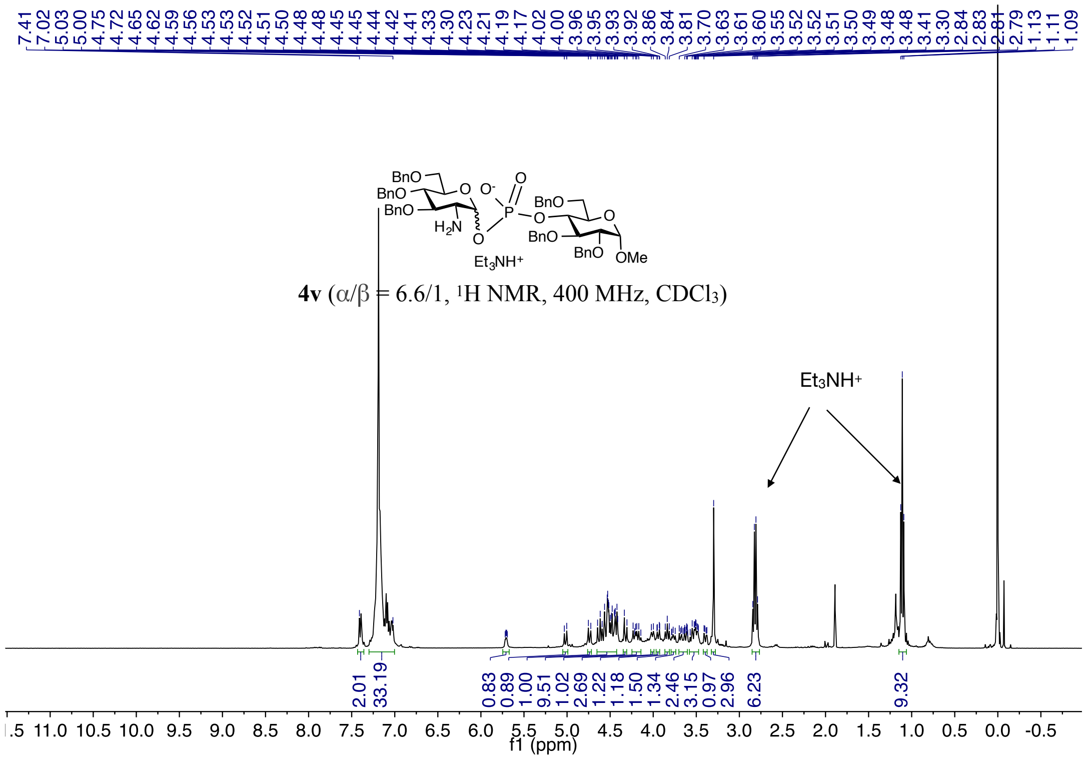

Supplementary Figure 132. ^{13}C NMR spectrum of compound **4v**

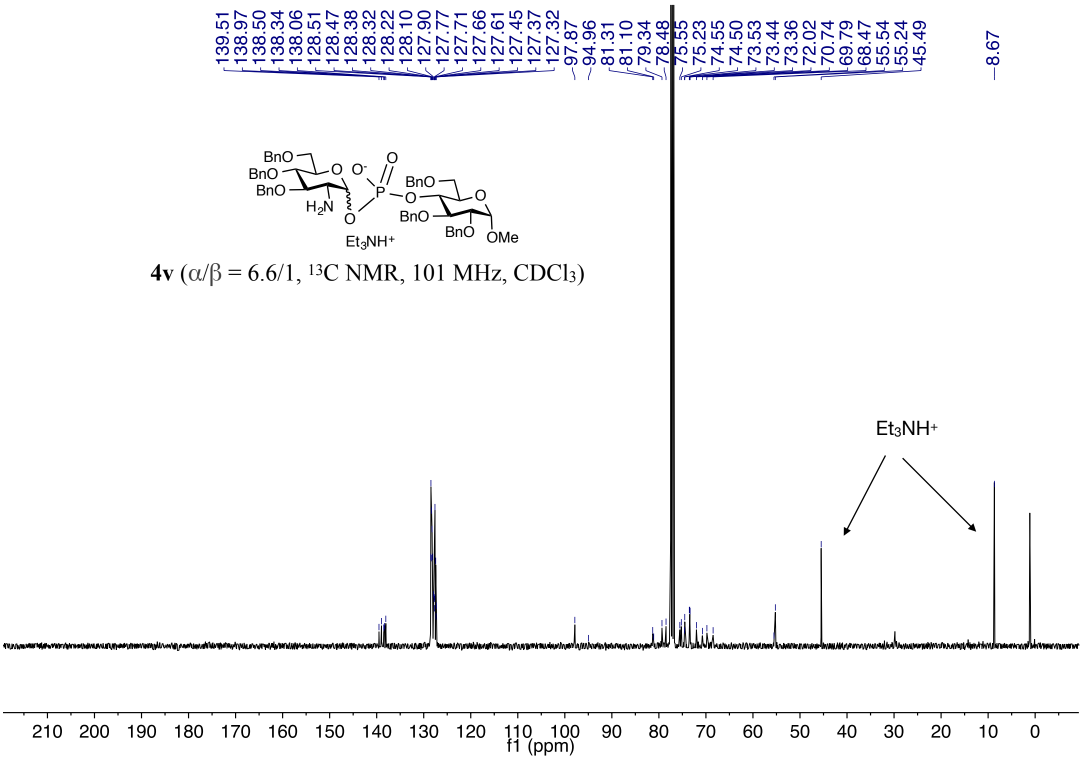

Supplementary Figure 133. ^{31}P NMR spectrum of compound **4v**

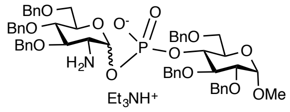

4v (α/β = 6.6/1, ^{31}P NMR, 162 MHz, $CDCl_3$)

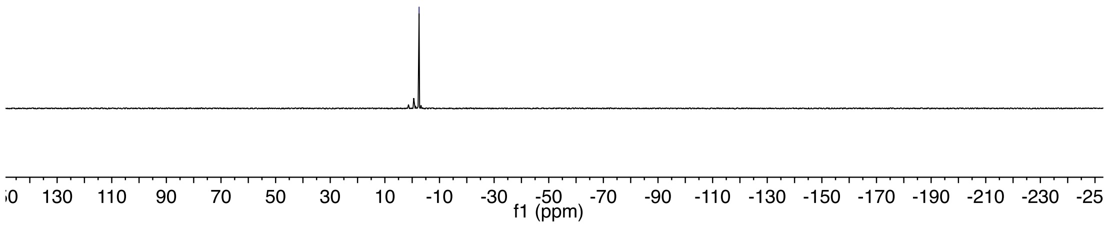

Supplementary Figure 134. ^{31}P NMR spectrum used to determined ratio of **4v**

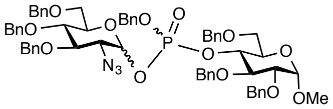

The α/β ratio was determined to be 6.6/1 by ^{31}P NMR

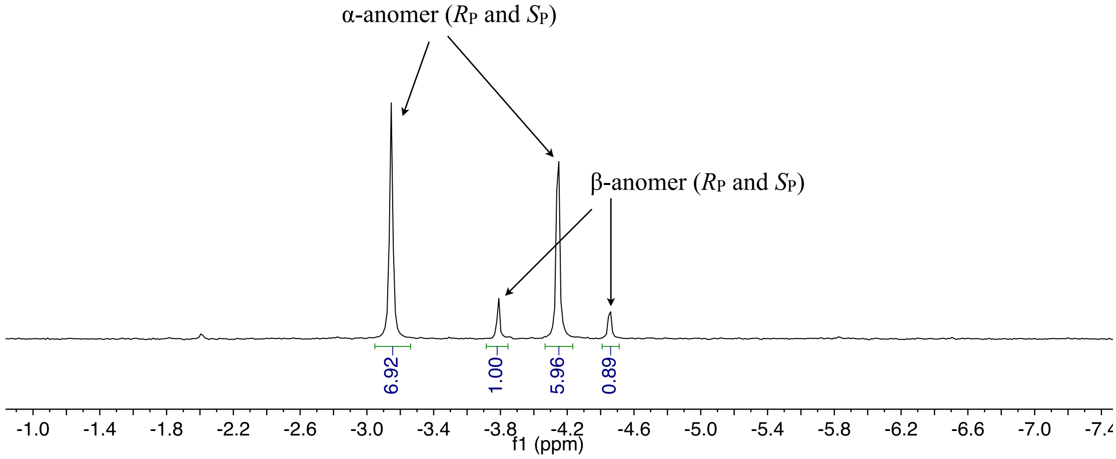

Supplementary Figure 135. ^{1}H NMR spectrum of compound **4w**

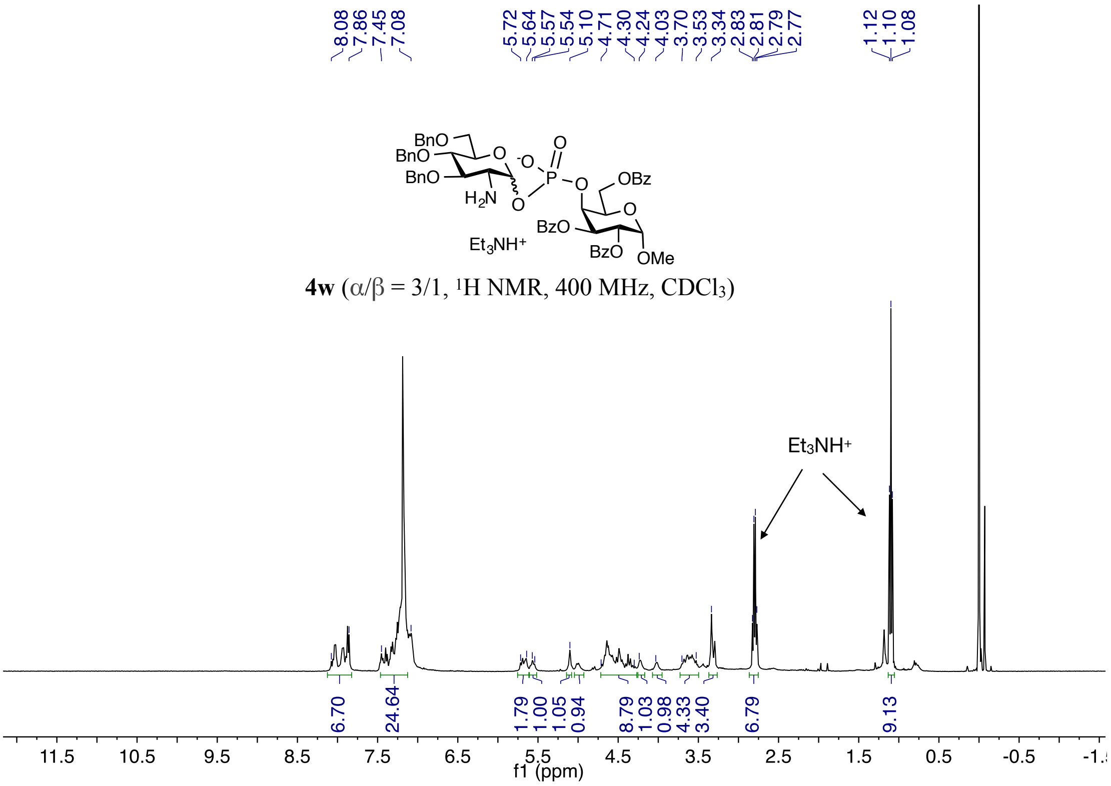

Supplementary Figure 136. ^{13}C NMR spectrum of compound **4w**

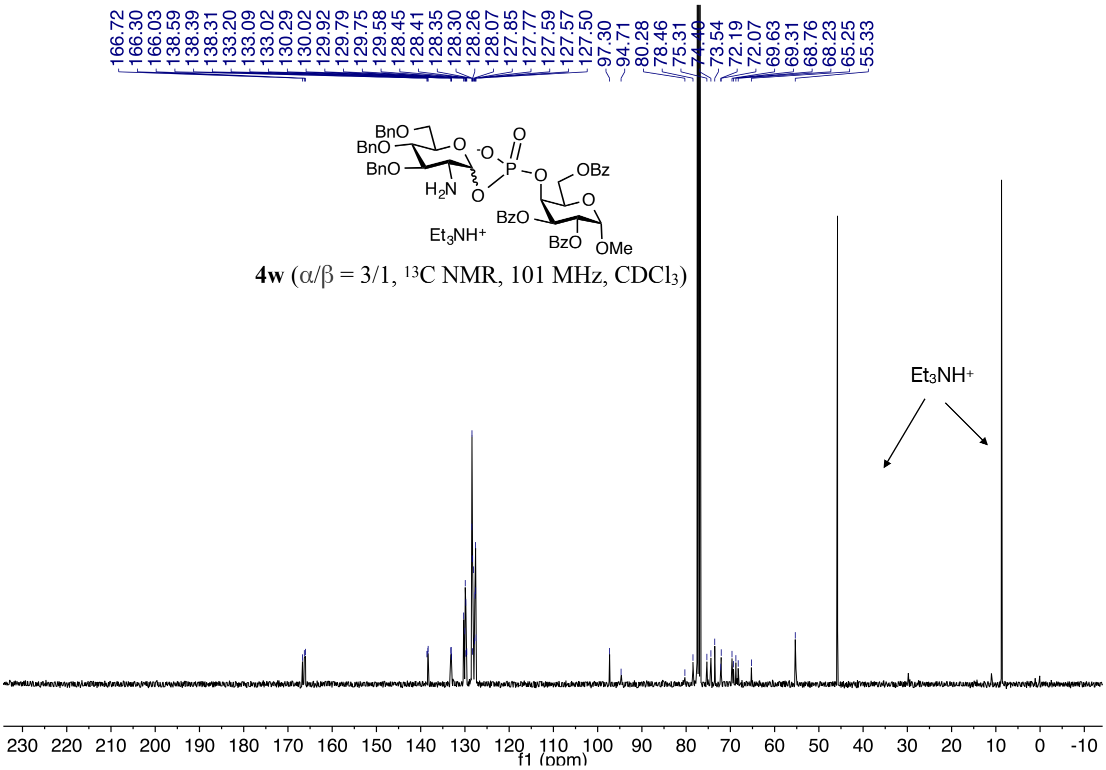

Supplementary Figure 137. ^{31}P NMR spectrum of compound **4w**

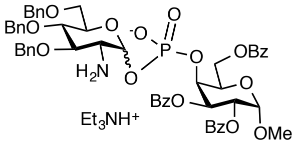

4w (α/β = 3/1, ^{31}P NMR, 162 MHz, $CDCl_3$)

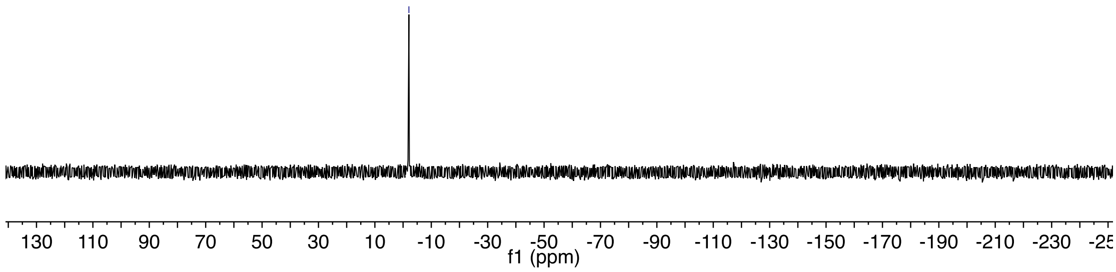

Supplementary Figure 138. ^{31}P NMR spectrum used to determine ratio of **4t**

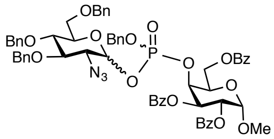

The α/β ratio was determined to be 3/1 by ^{31}P NMR

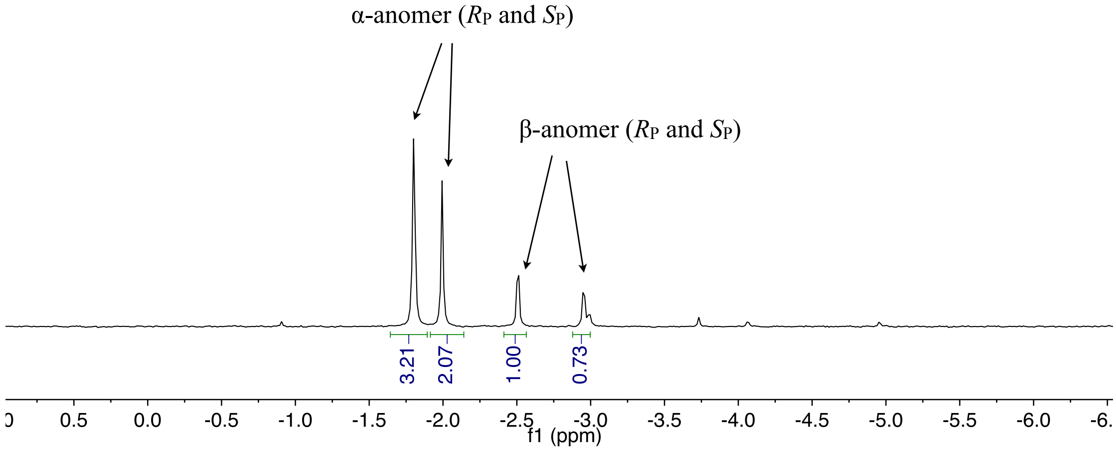

Supplementary Figure 139. ^{1}H NMR spectrum of compound **4x**

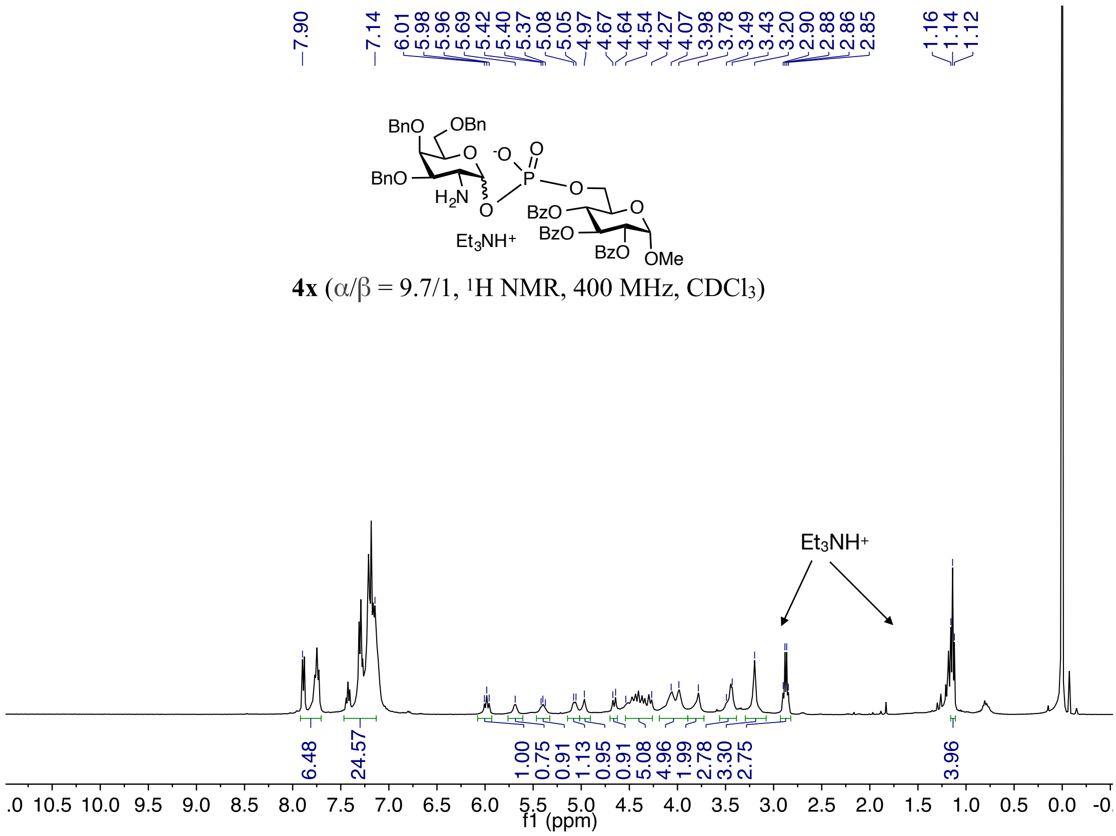

Supplementary Figure 140. ^{13}C NMR spectrum of compound **4x**

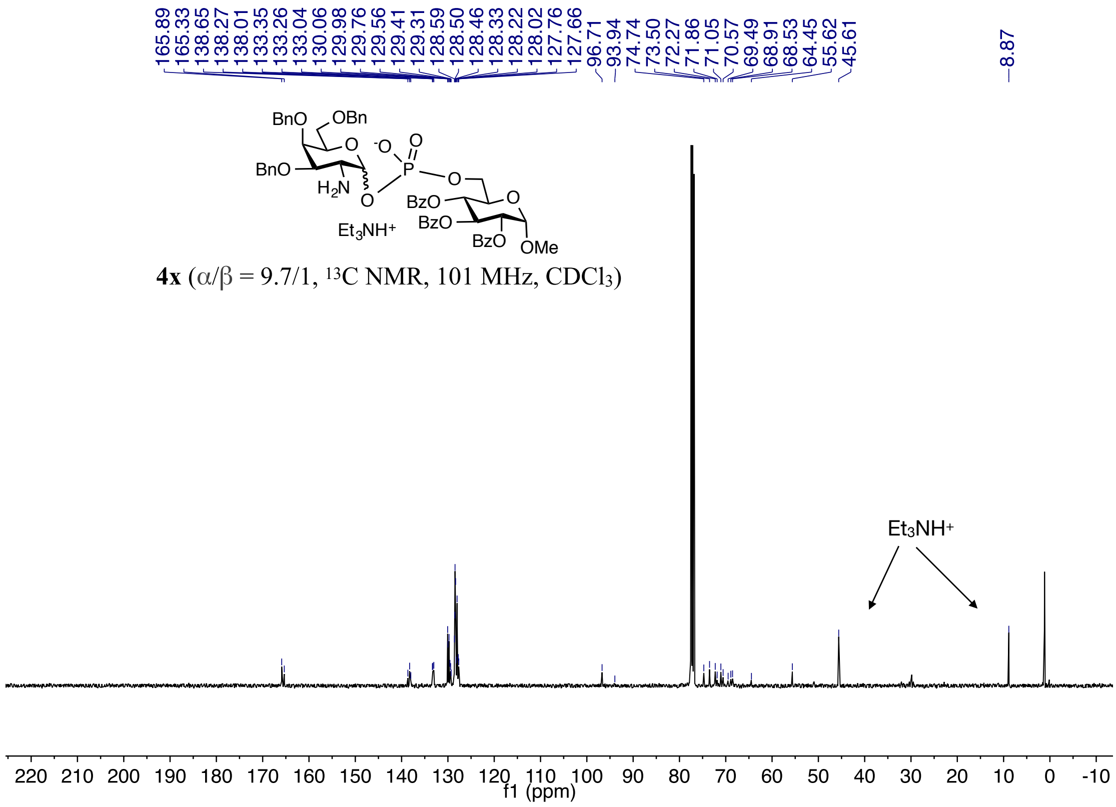

Supplementary Figure 141. ^{31}P NMR spectrum of compound **4x**

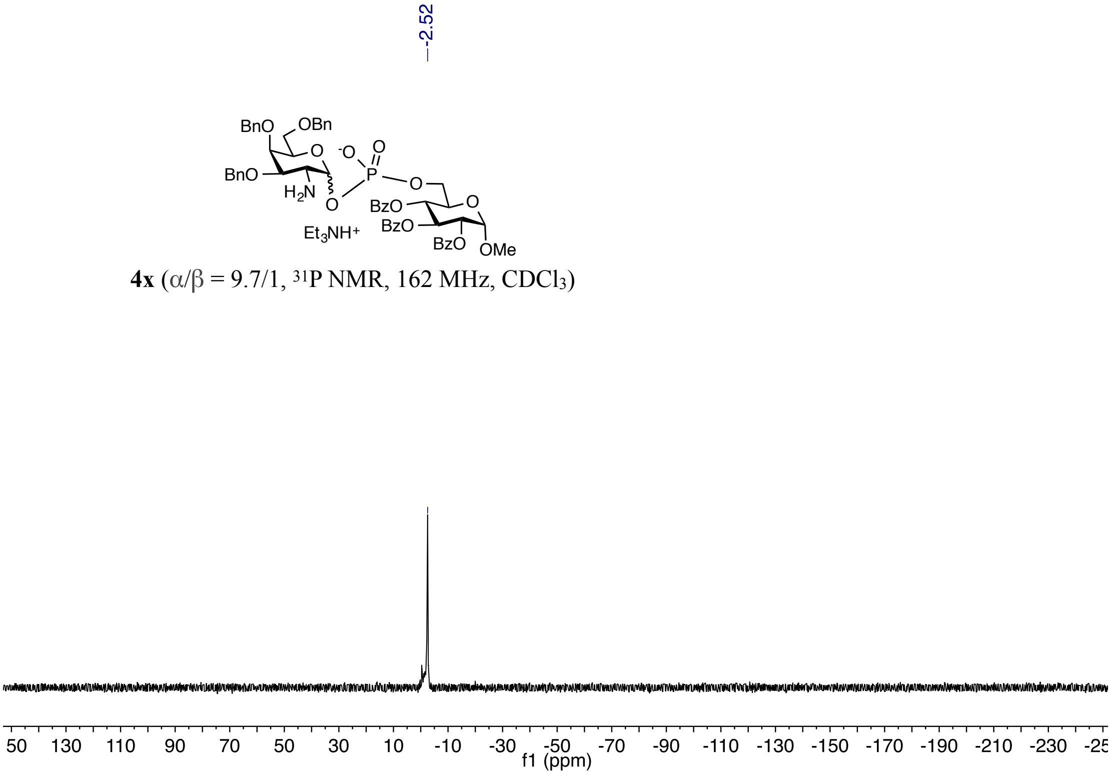

Supplementary Figure 142. ^{31}P NMR spectrum used to determined ratio of **4x**

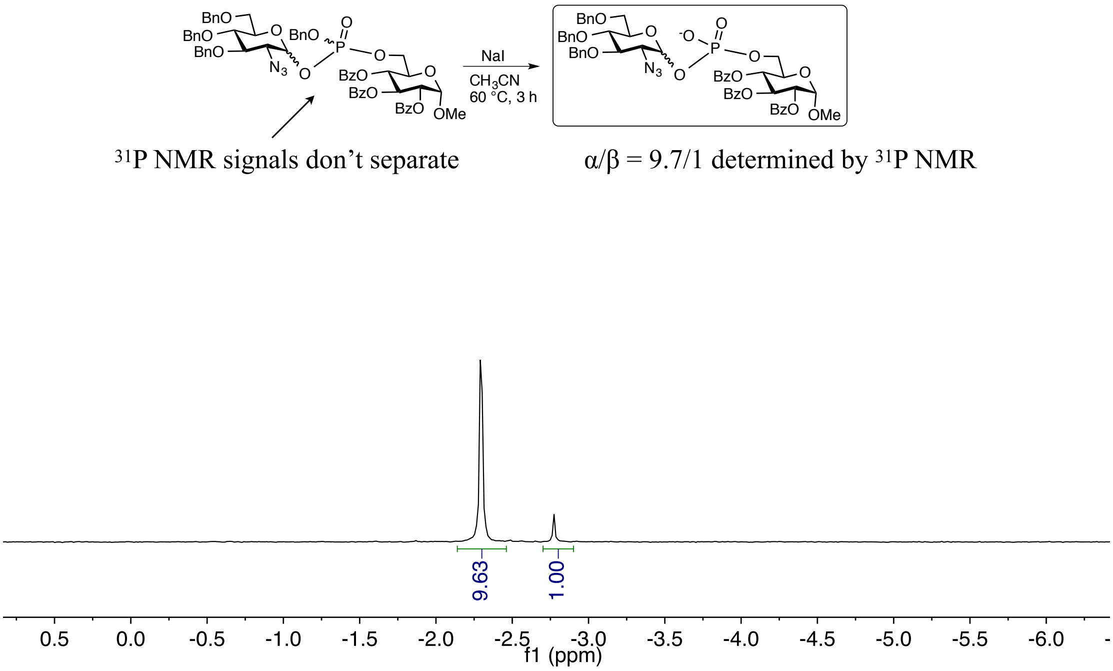

Supplementary Figure 143. ^{1}H NMR spectrum of compound **4y**

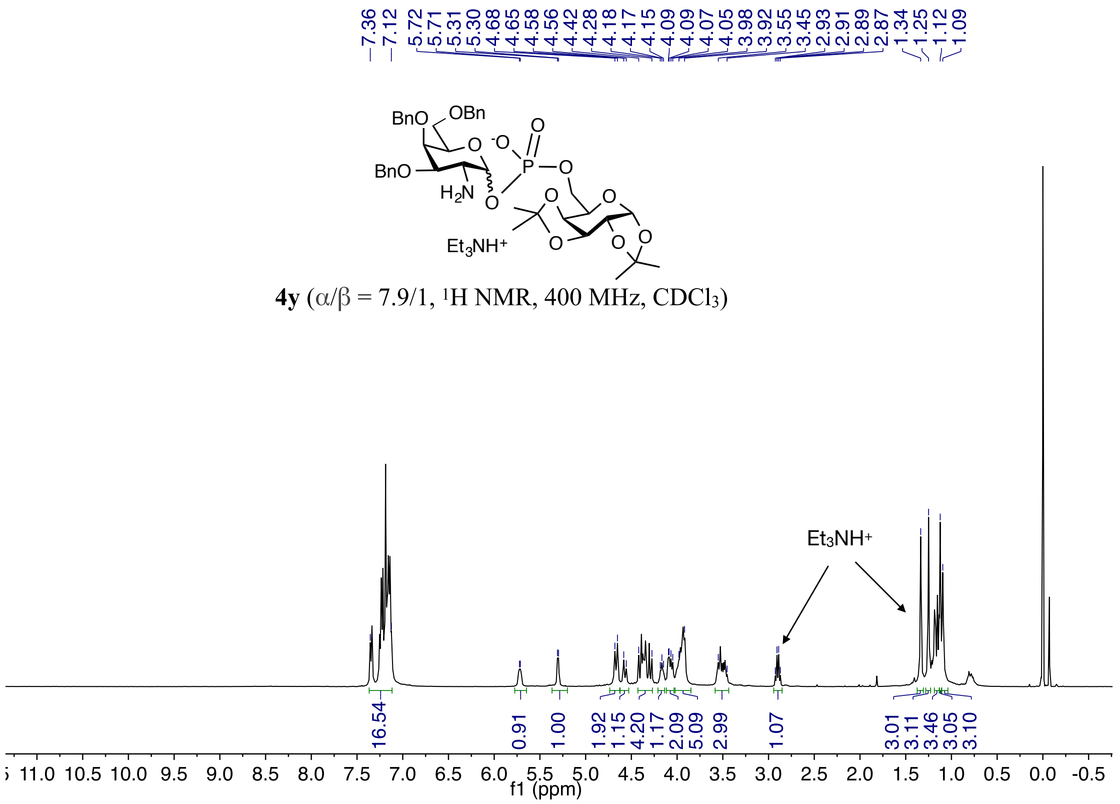

Supplementary Figure 144. ^{13}C NMR spectrum of compound **4y**

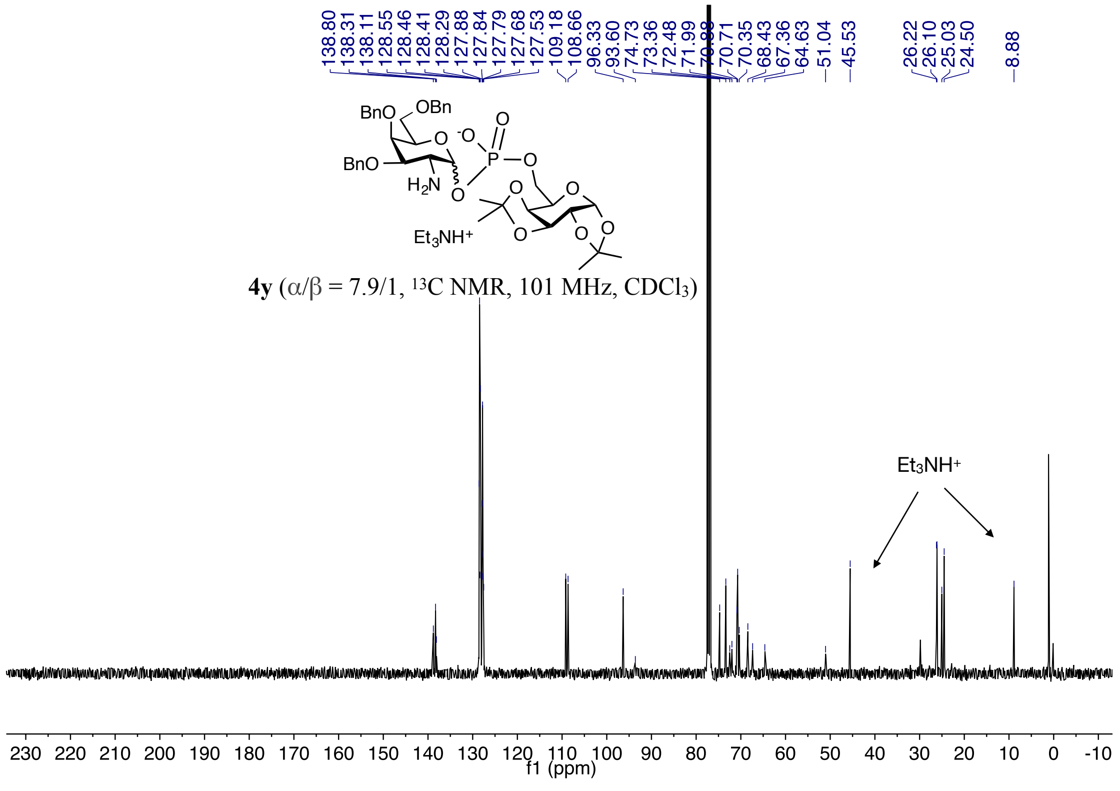

Supplementary Figure 145. ^{31}P NMR spectrum of compound **4y**

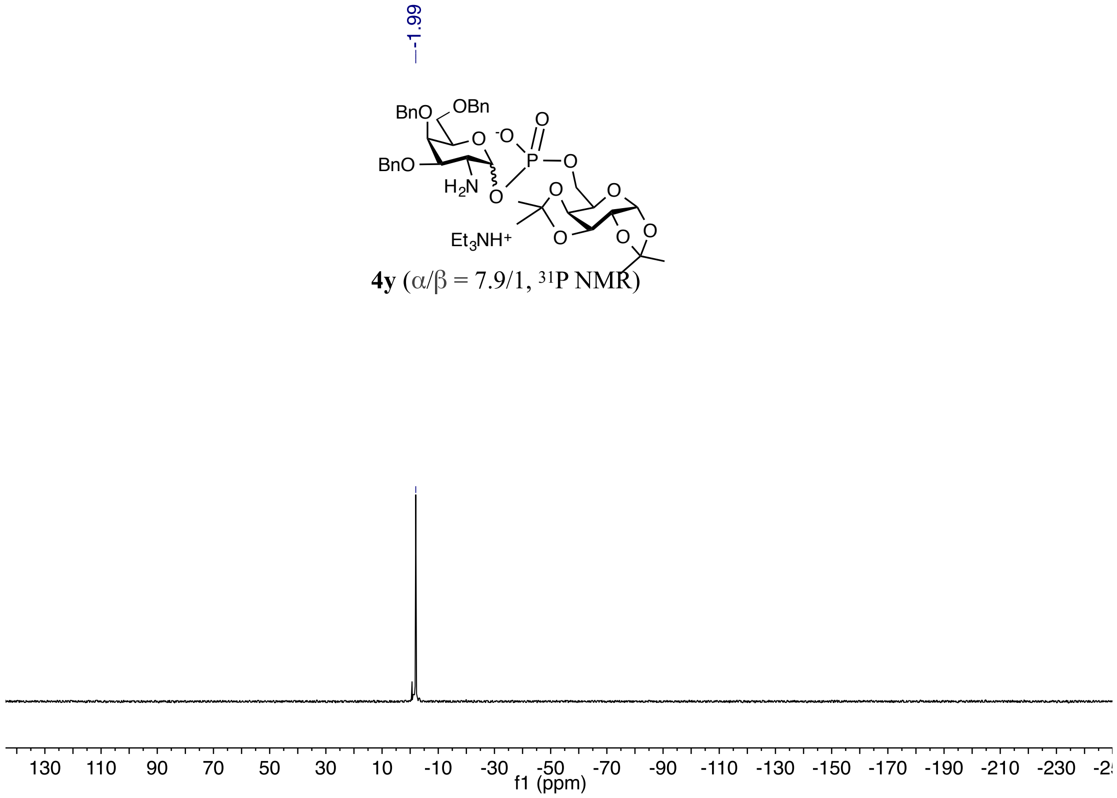

Supplementary Figure 146. ^{31}P NMR spectrum used to determined ratio of compound **4y**

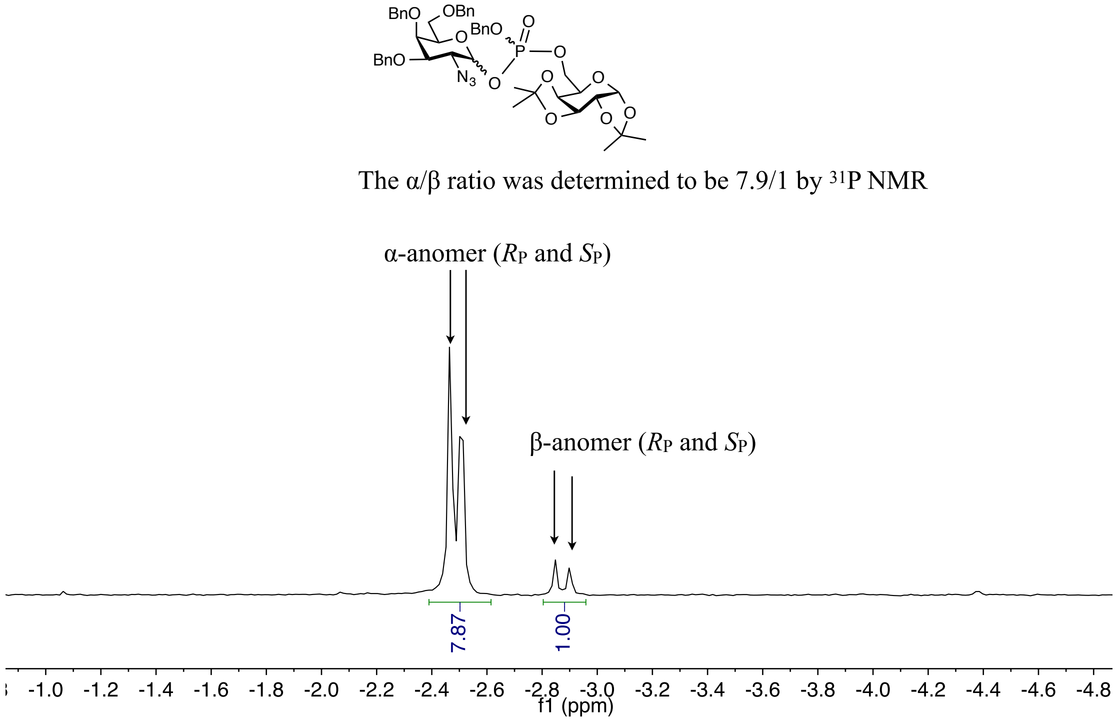

Supplementary Figure 147. ^{1}H NMR spectrum of compound **4z**

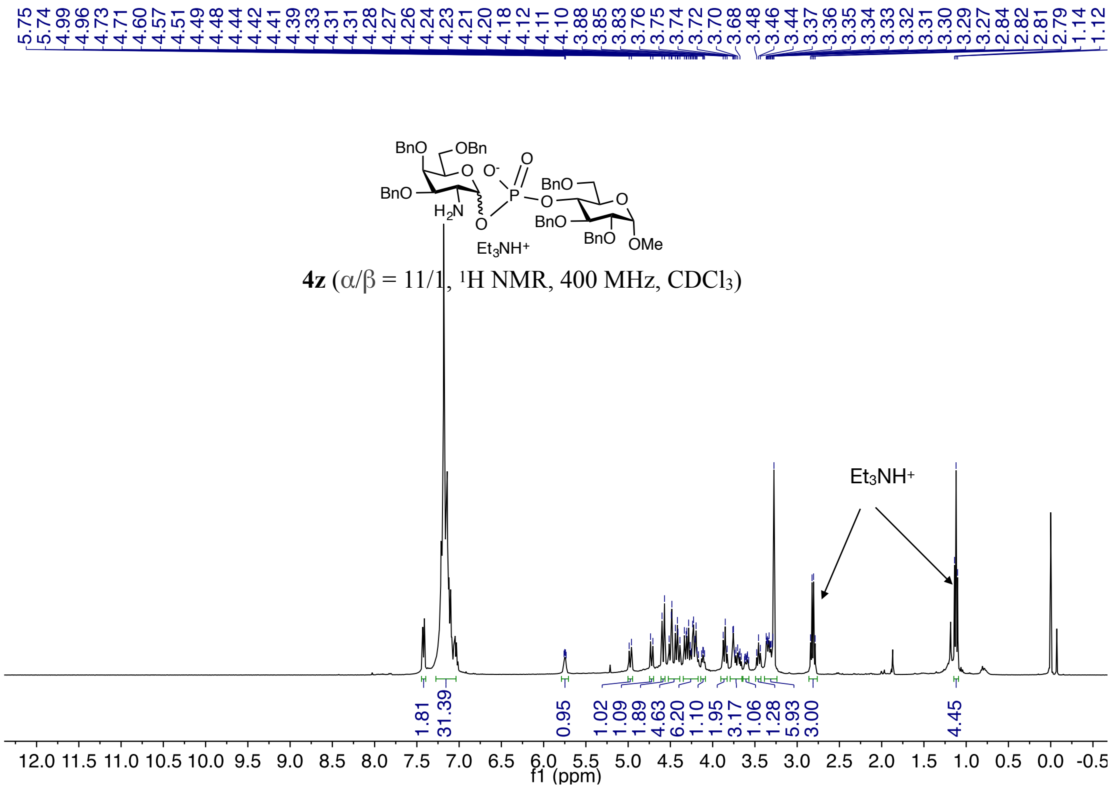

Supplementary Figure 148. ^{13}C NMR spectrum of compound **4z**

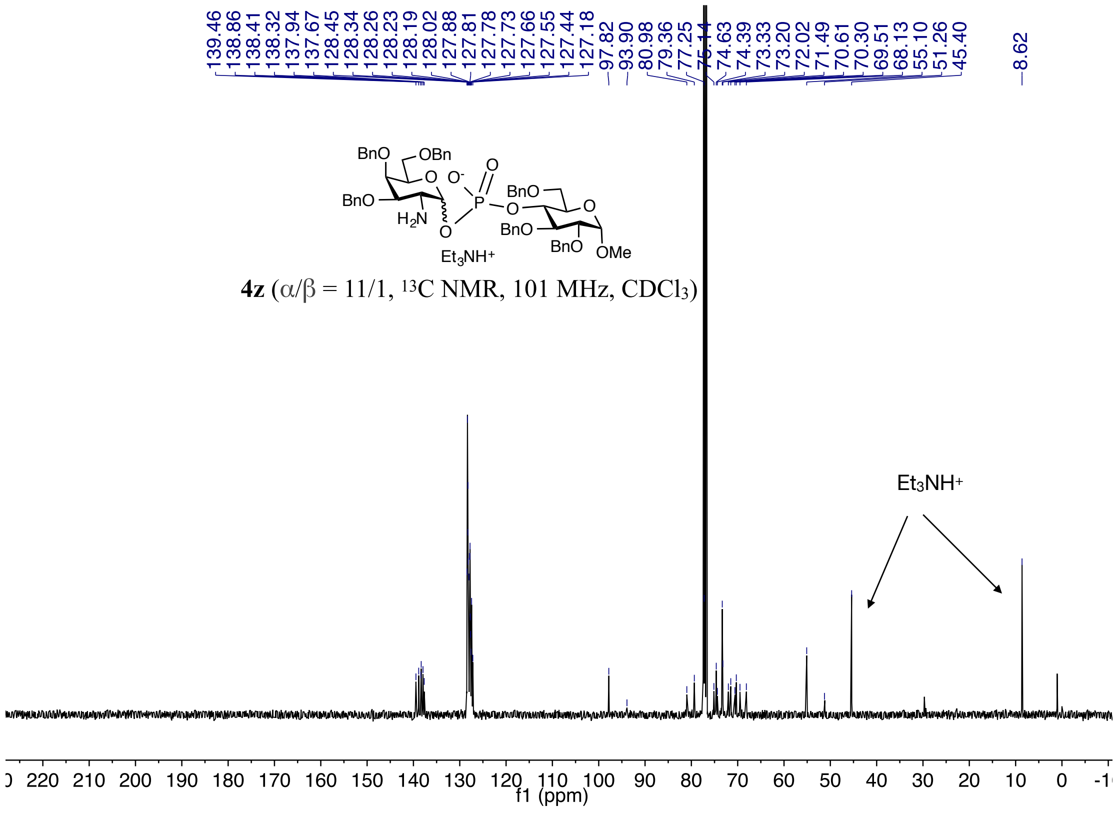

Supplementary Figure 149. ^{31}P NMR spectrum of compound **4z**

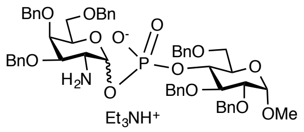

4z (α/β = 11/1, ^{31}P NMR, 162 MHz, CDCl$_3$)

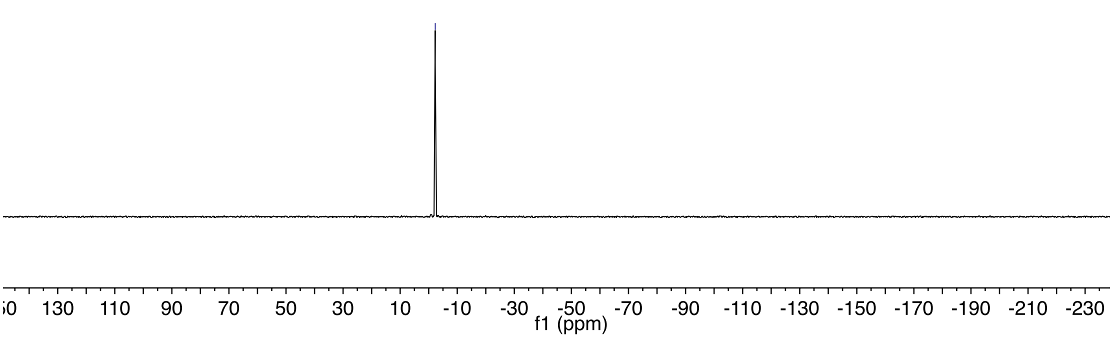

Supplementary Figure 150. ^{31}P NMR spectrum used to determined ratio of compound **4z**

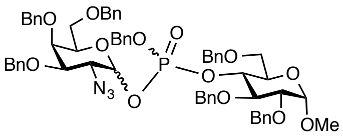

The α/β ratio was determined to be 11/1 by ^{31}P NMR

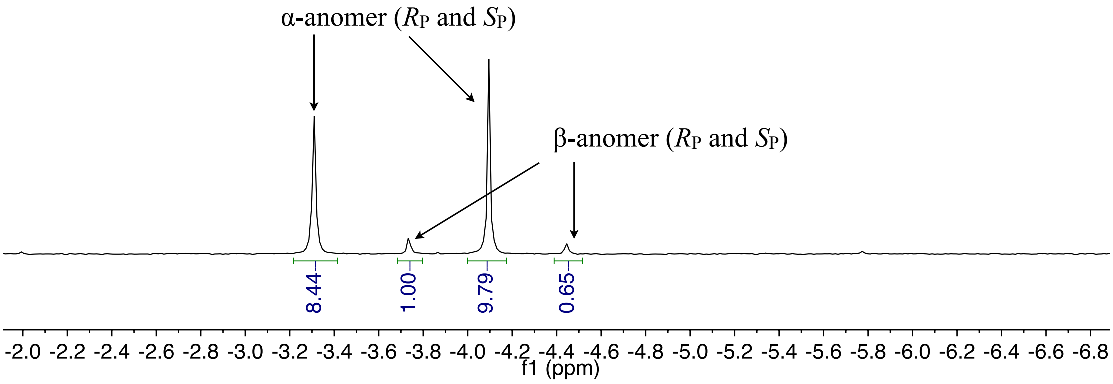

Supplementary Figure 151. ^{1}H NMR spectrum of compound **4za**

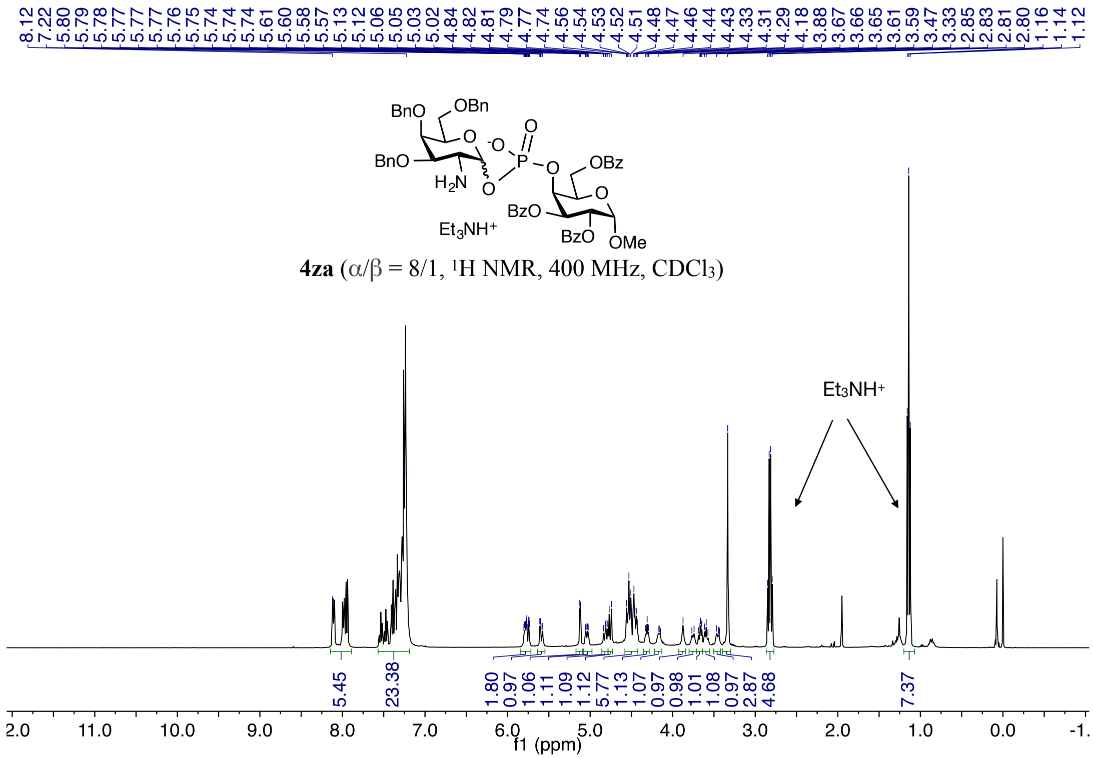

Supplementary Figure 152. ^{13}C NMR spectrum of compound **4za**

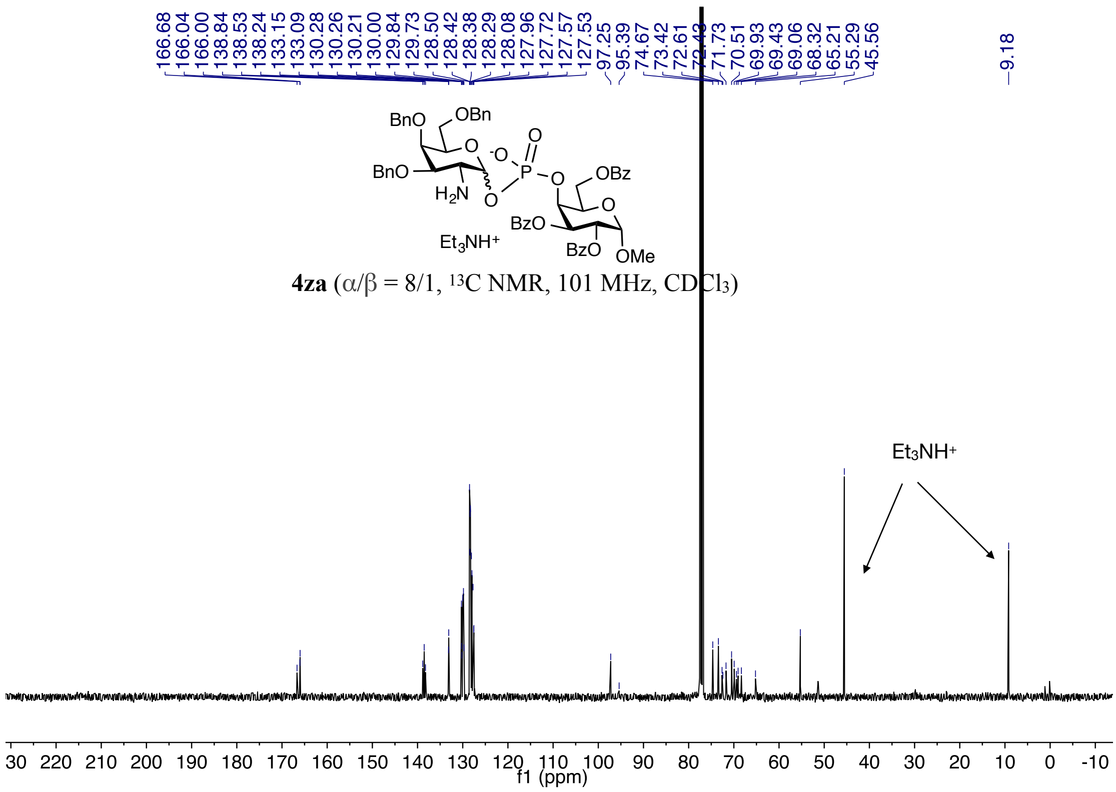

Supplementary Figure 153. ^{31}P NMR spectrum of compound **4za**

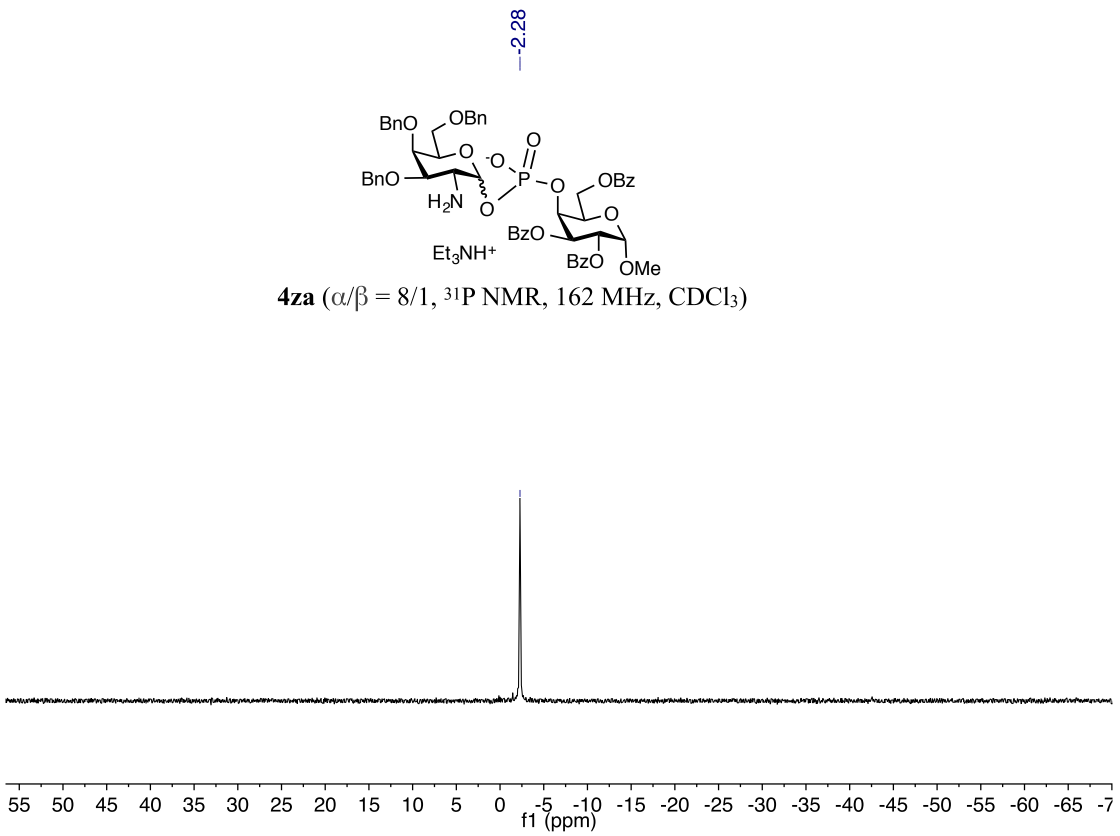

4za (α/β = 8/1, ^{31}P NMR, 162 MHz, $CDCl_3$)

Supplementary Figure 154. ^{31}P NMR spectrum used to determined ratio of **4za**

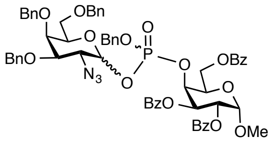

The α/β ratio was determined to be 8/1 by ^{31}P NMR

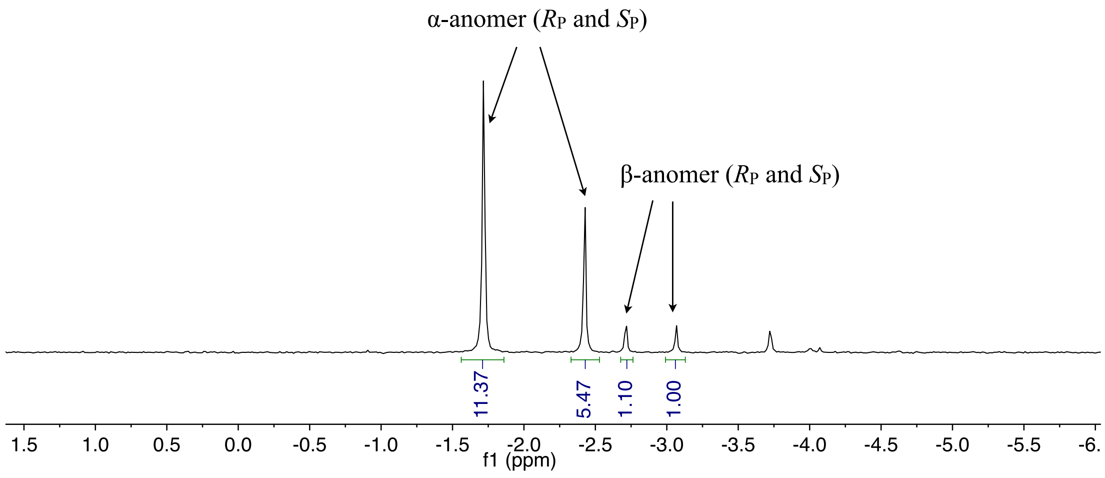

Supplementary Figure 155. ^{1}H NMR spectrum of compound **4zb**

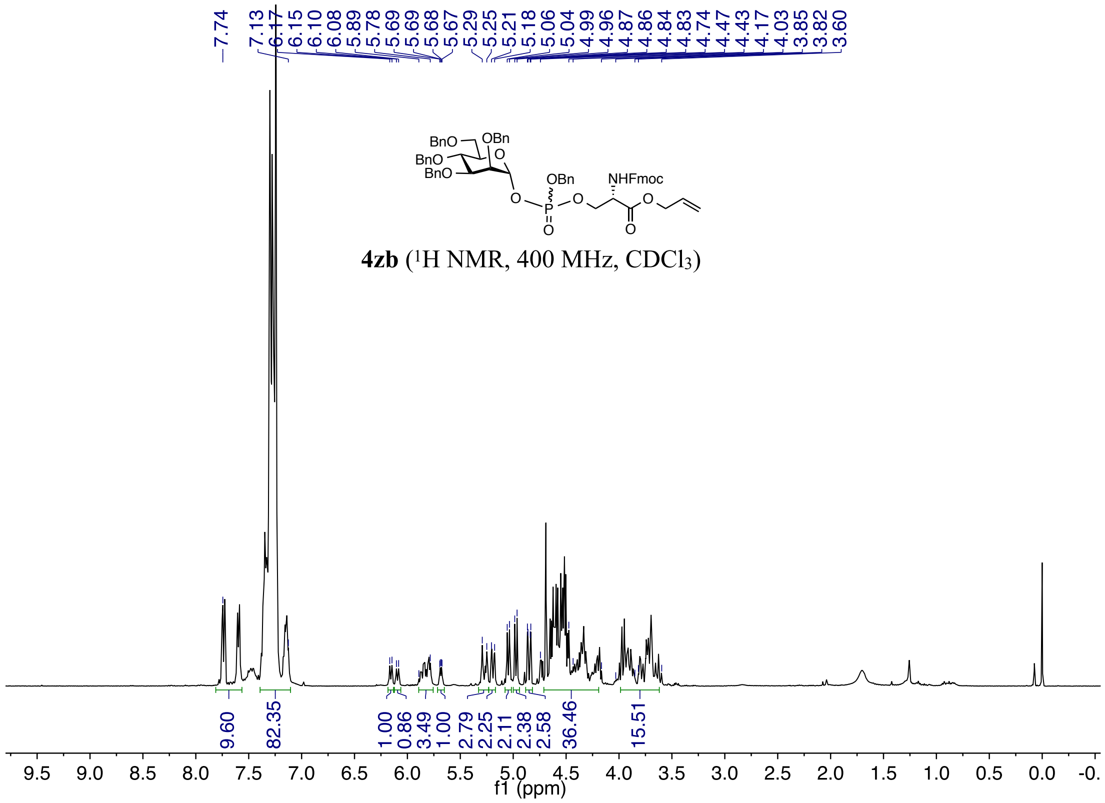

Supplementary Figure 156. ^{13}C NMR spectrum of compound **4zb**

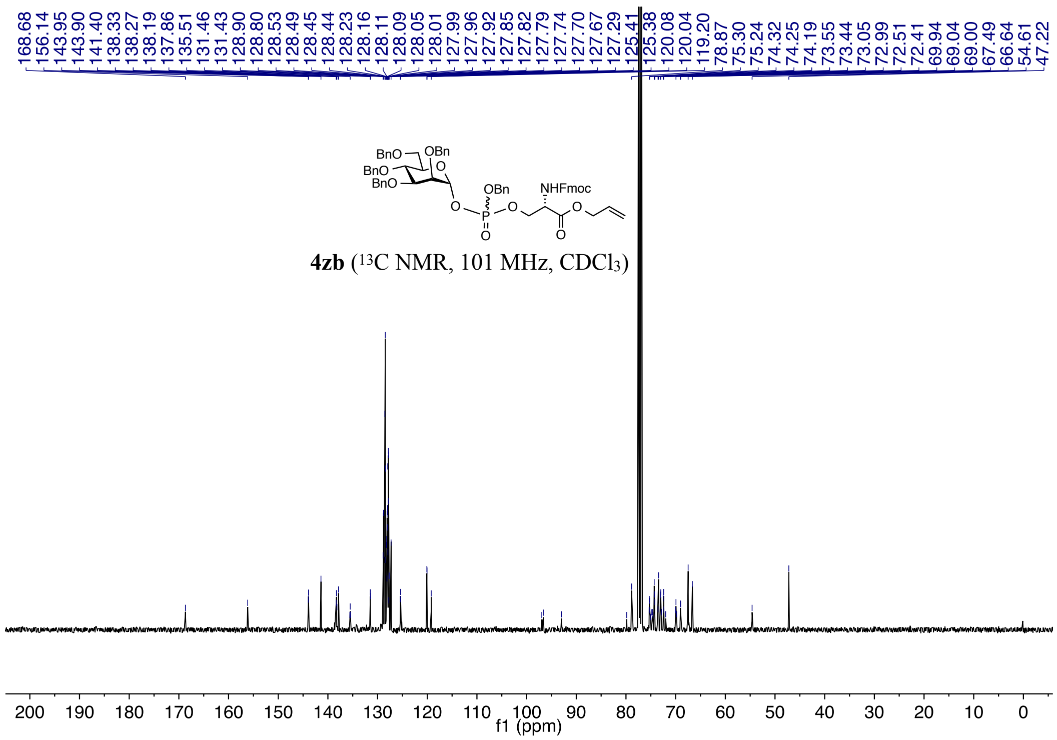

Supplementary Figure 157. ^{31}P NMR spectrum of compound **4zb**

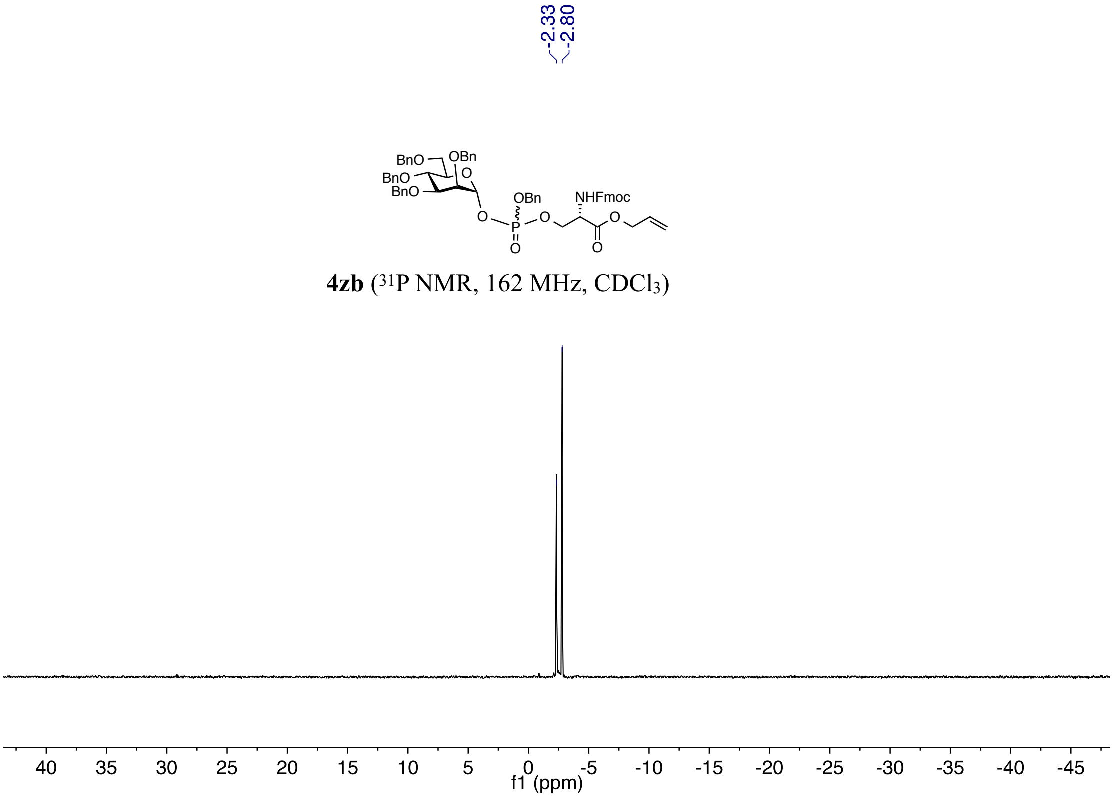

Supplementary Figure 158. ^{1}H NMR spectrum of compound **4zc**

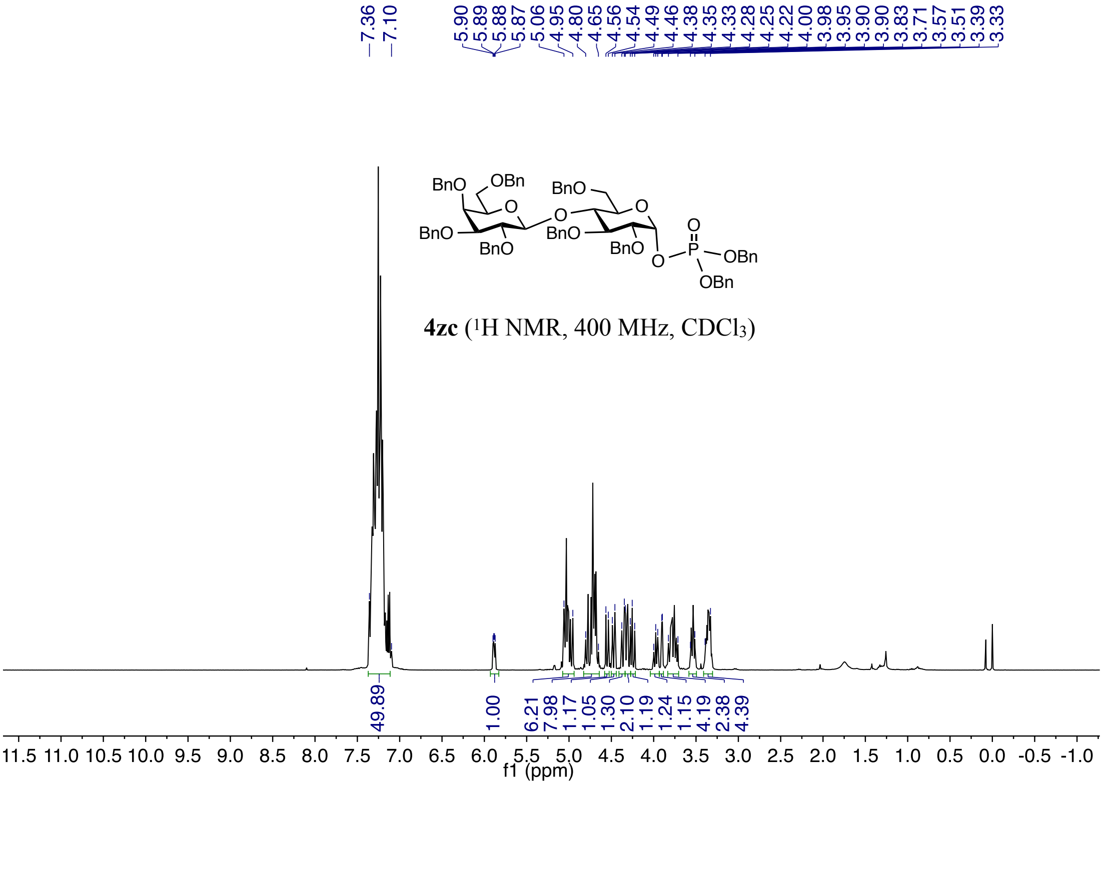

Supplementary Figure 159. ^{13}C NMR spectrum of compound **4zc**

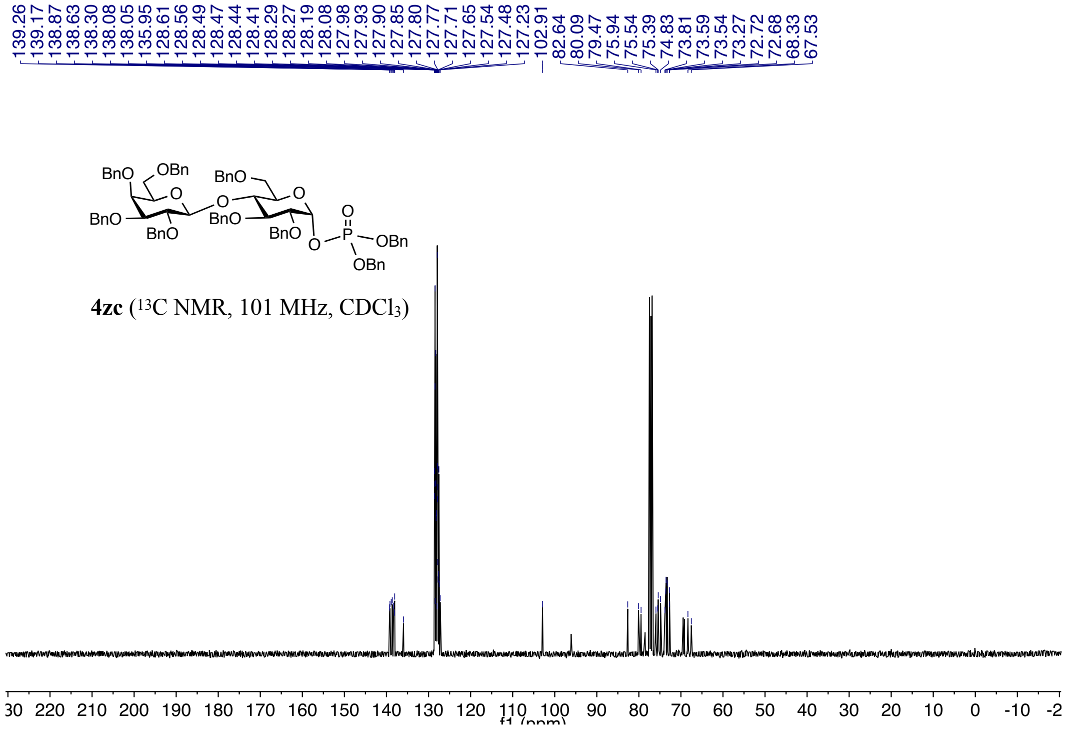

Supplementary Figure 160. ^{31}P NMR spectrum of compound **4zc**

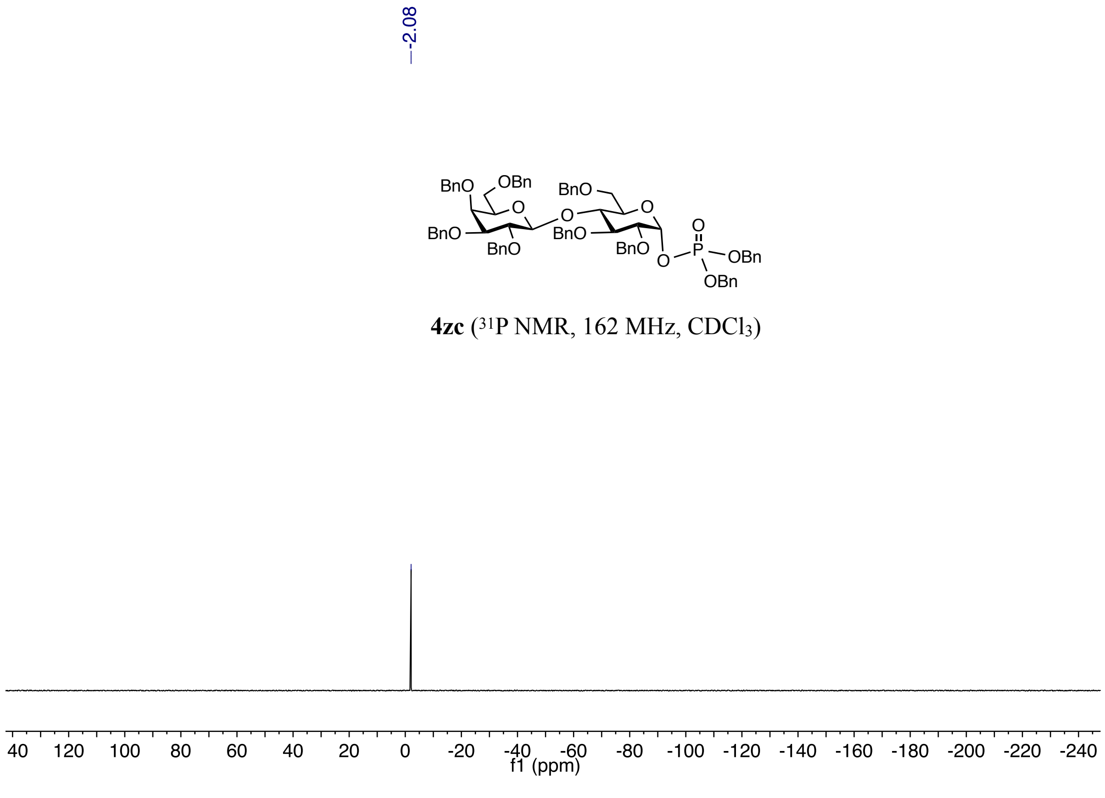

Supplementary Figure 161. ^{1}H NMR spectrum of compound **4zd**

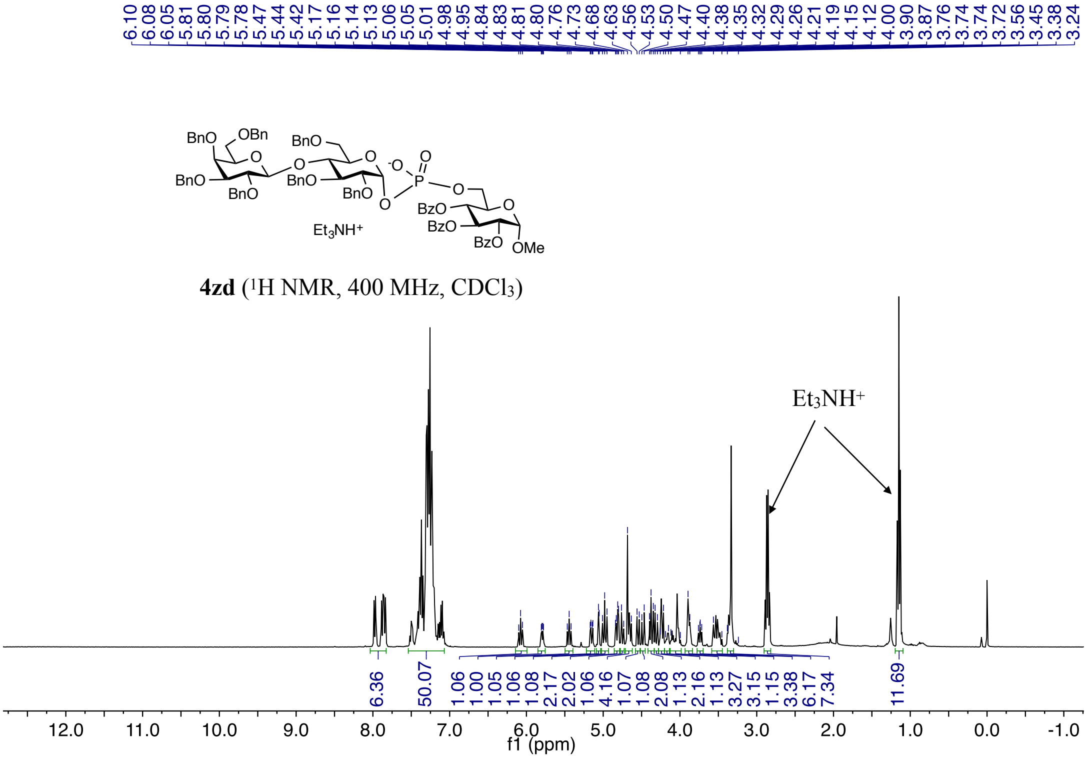

Supplementary Figure 162. ^{13}C NMR spectrum of compound **4zd**

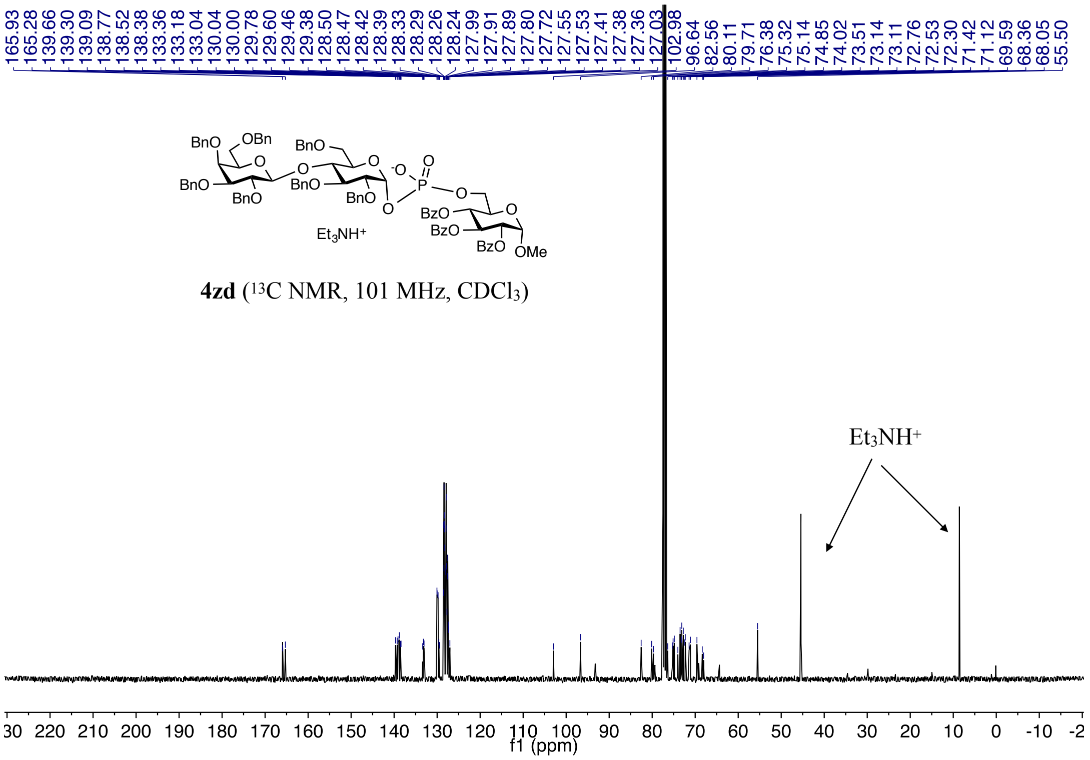

Supplementary Figure 163. ^{31}P NMR spectrum of compound **4zd**

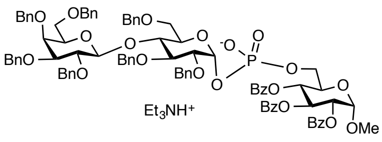

4zd (^{31}P NMR, 162 MHz, $CDCl_3$)

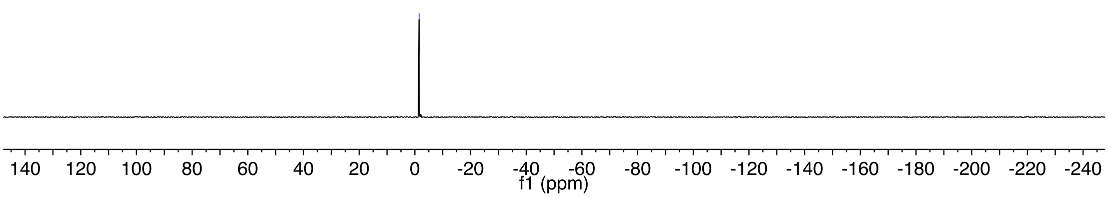

Supplementary Figure 164. ^{1}H NMR spectrum of compound **4ze**

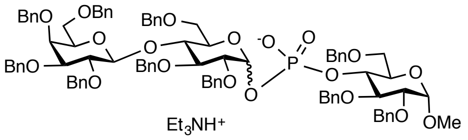

4ze (α/β = 2.8/1, ^{1}H NMR, 400 MHz, $CDCl_3$)

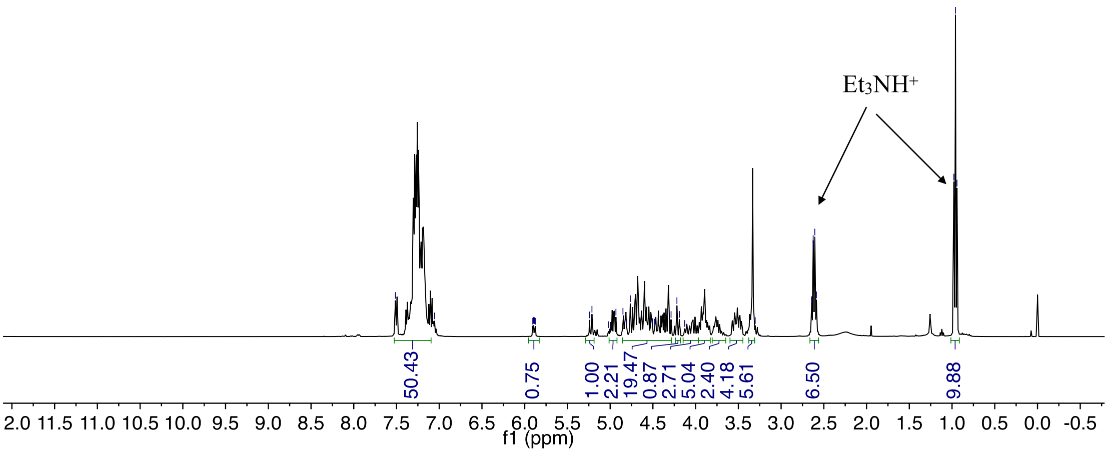

Supplementary Figure 165. ^{13}C NMR spectrum of compound **4ze**

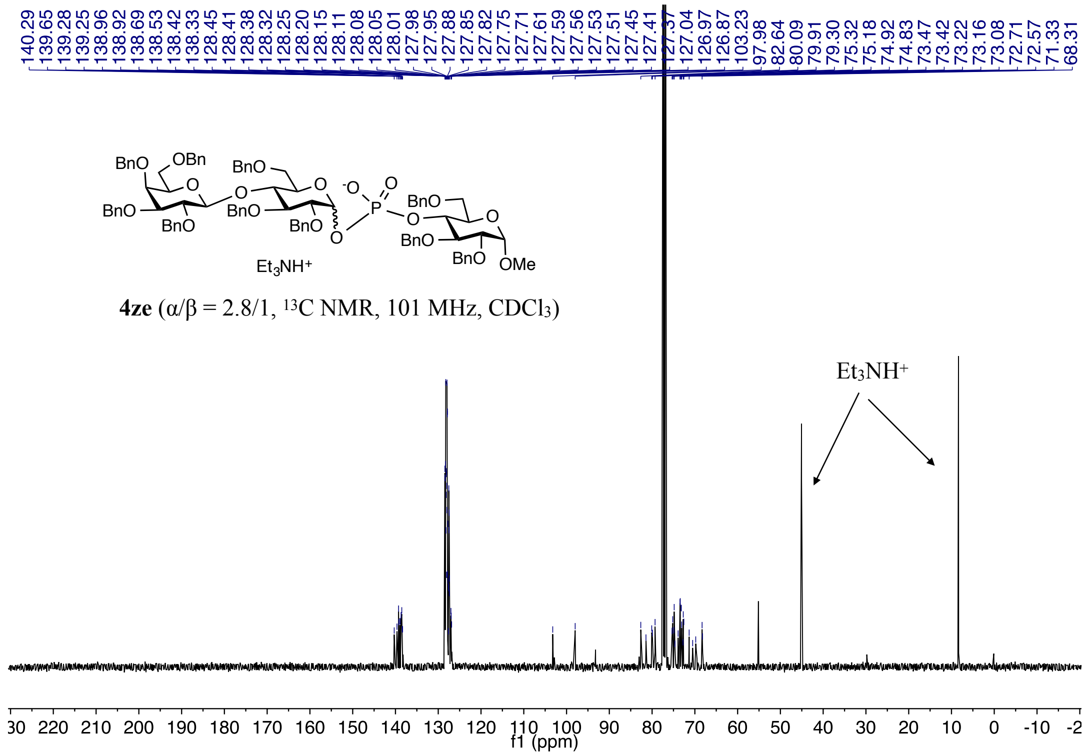

Supplementary Figure 166. ^{31}P NMR spectrum of compound **4ze**

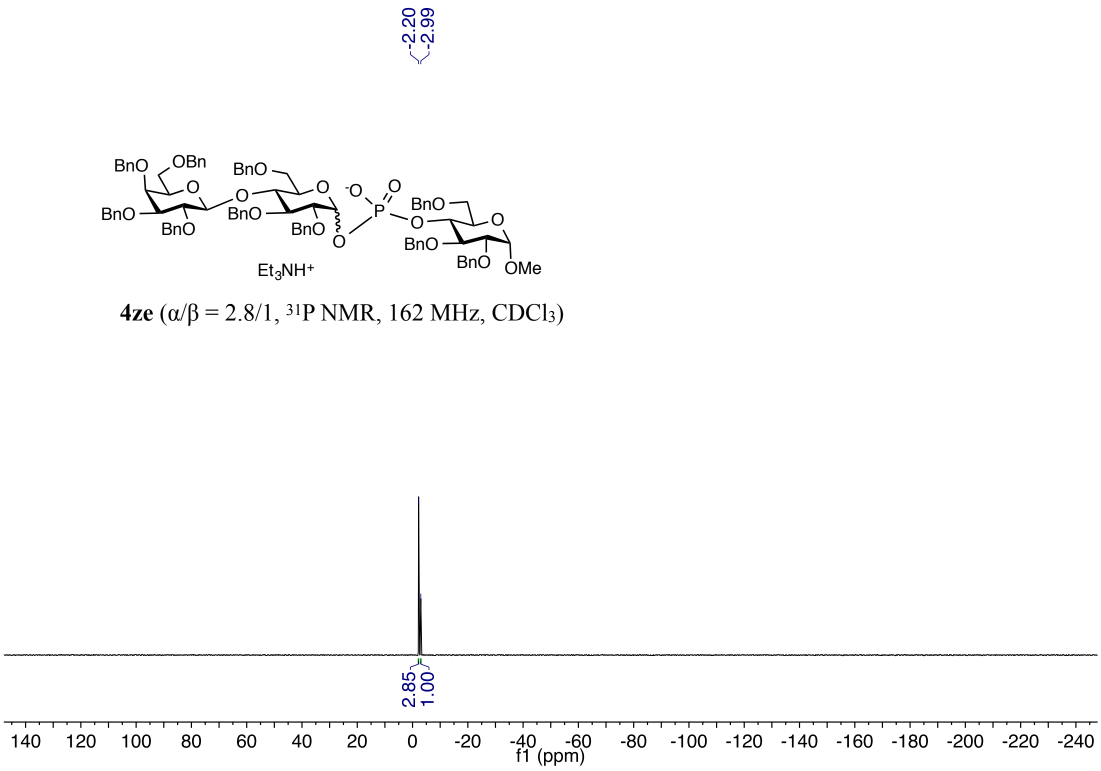

Supplementary Figure 167. ^{1}H NMR spectrum of compound **4zf**

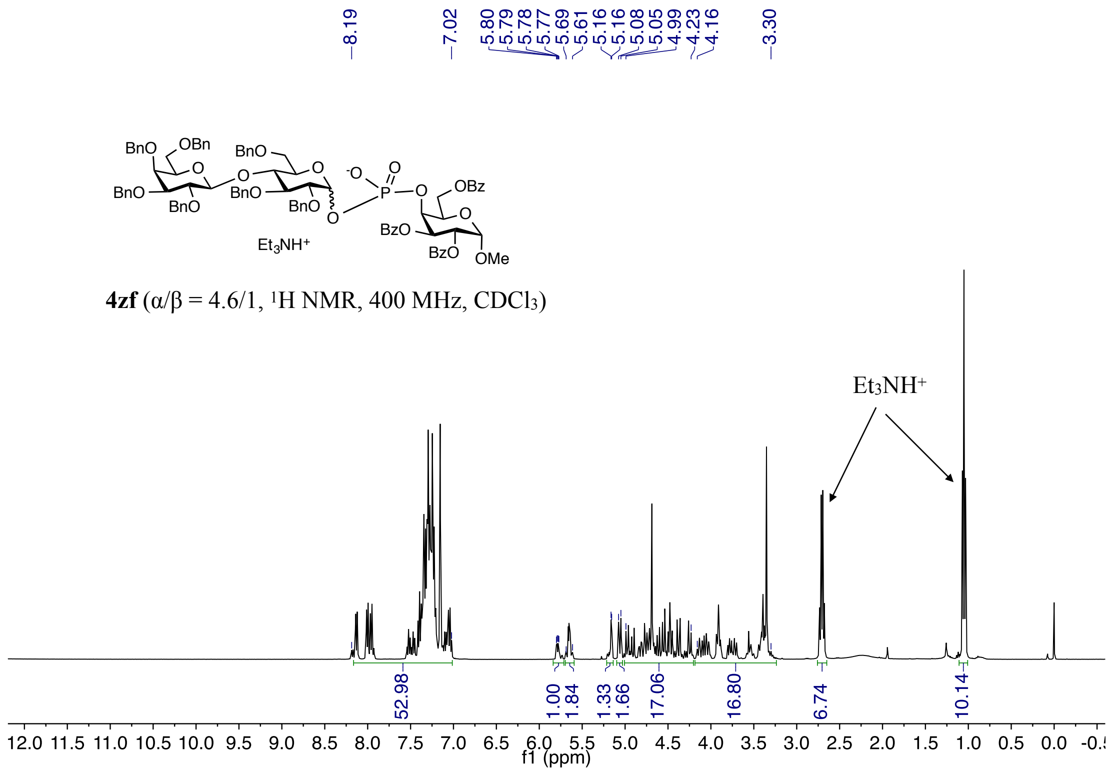

Supplementary Figure 168. ^{13}C NMR spectrum of compound **4zf**

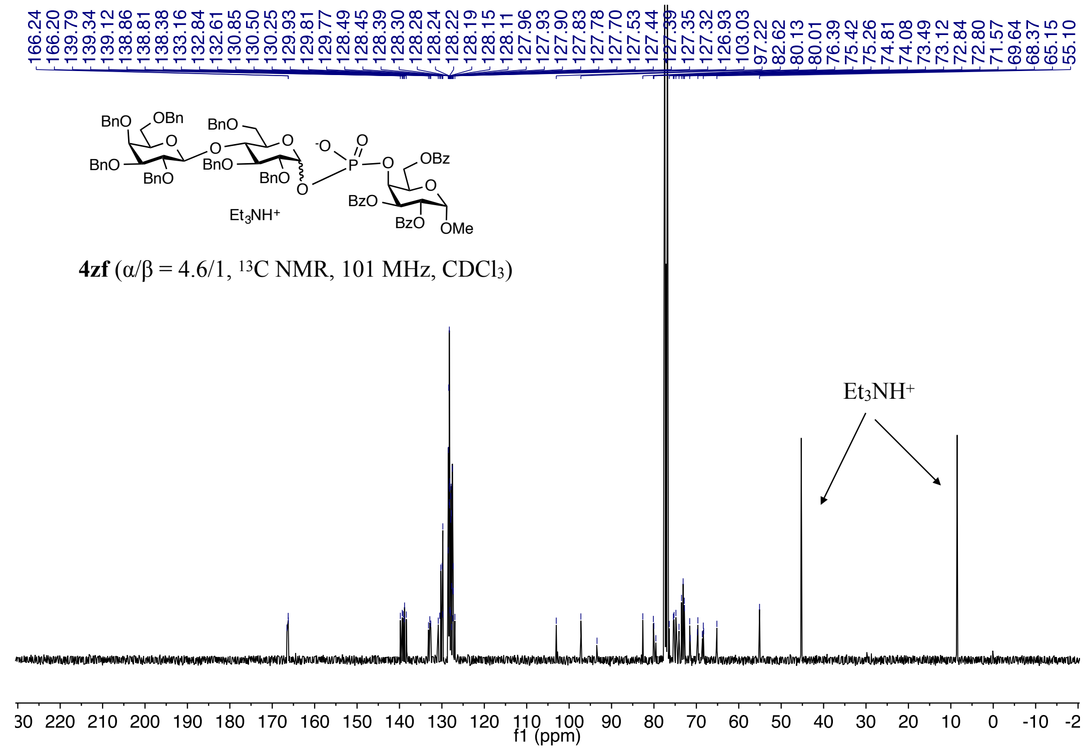

Supplementary Figure 169. ^{31}P NMR spectrum of compound **4zf**

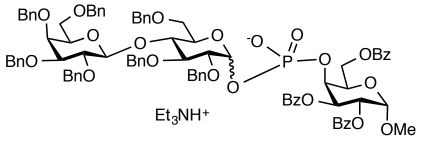

4zf (α/β = 4.6/1, ^{31}P NMR, 162 MHz, $CDCl_3$)

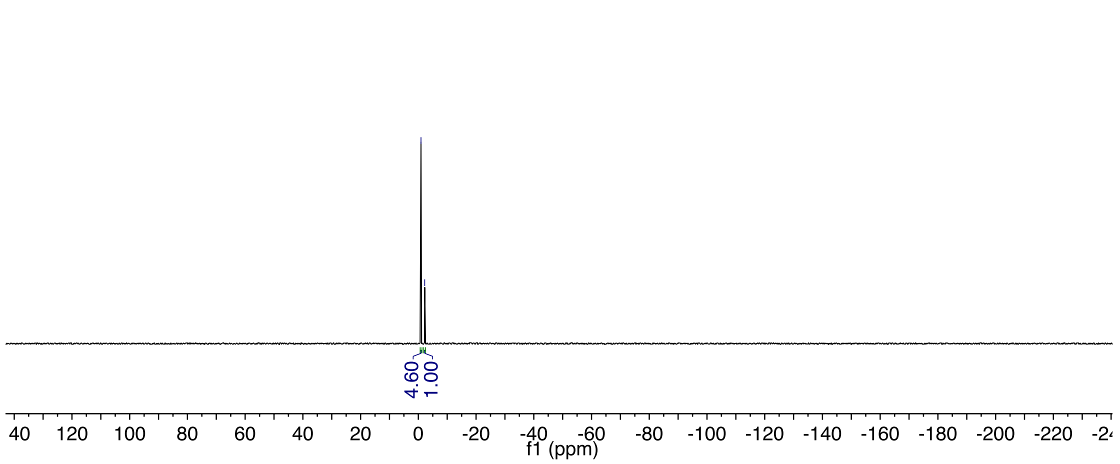

Supplementary Figure 170. ^{1}H NMR spectrum of compound **4zg**

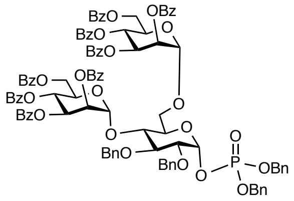

4zg (^{1}H NMR, 400 MHz, $CDCl_3$)

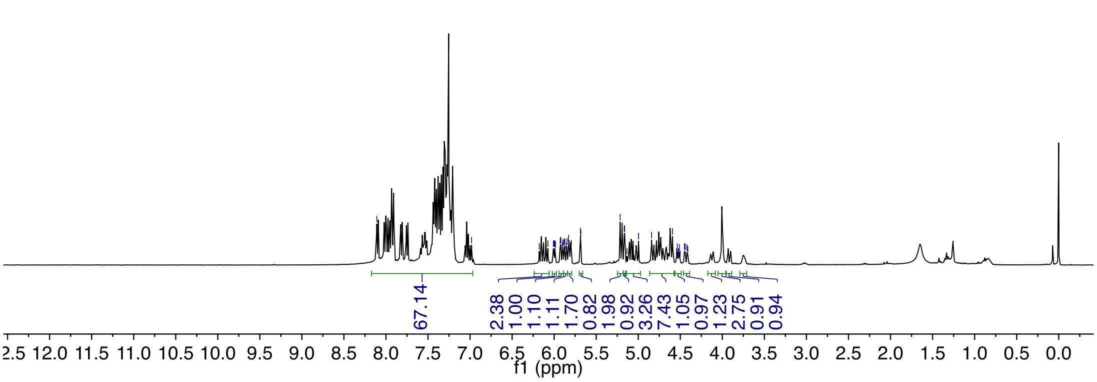

Supplementary Figure 171. ^{13}C NMR spectrum of compound **4zg**

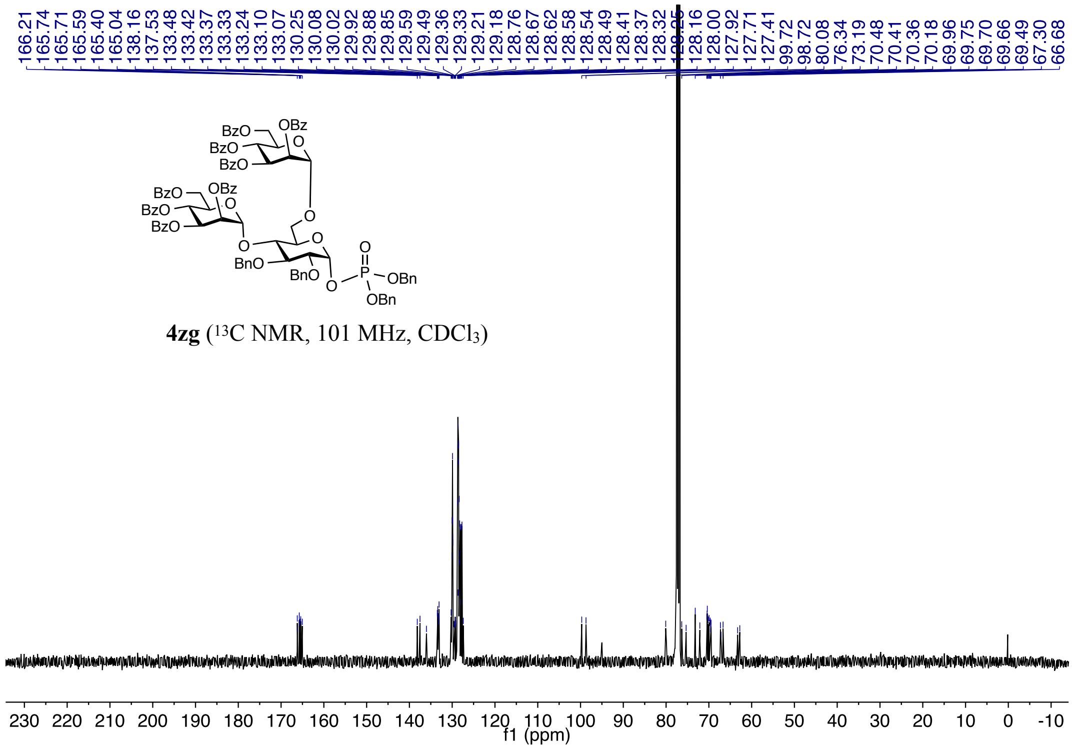

Supplementary Figure 172. ^{31}P NMR spectrum of compound **4zg**

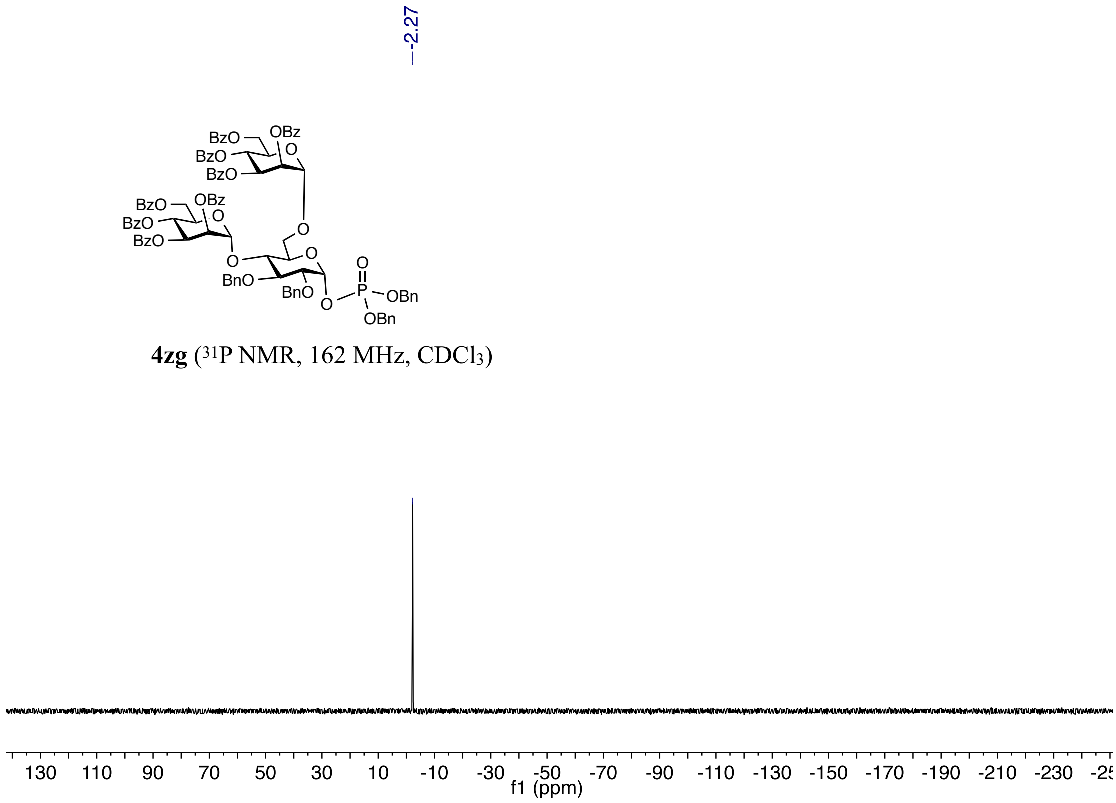

Supplementary Figure 173. ^{1}H NMR spectrum of compound **1b**

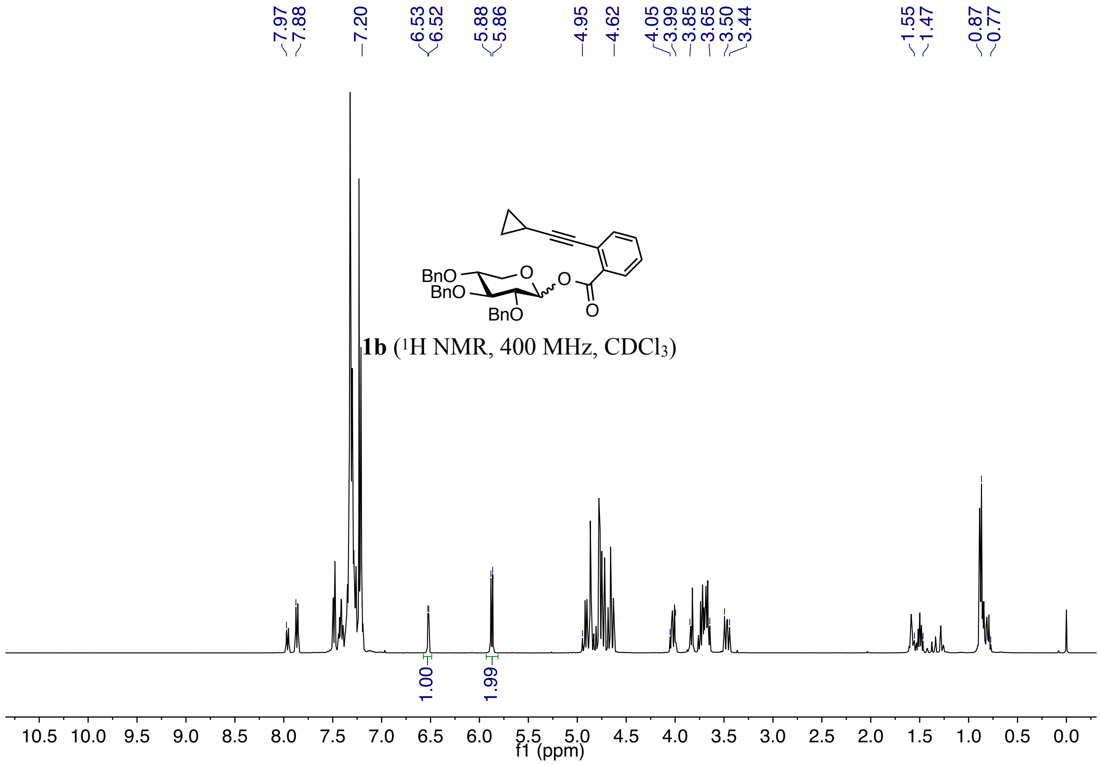

Supplementary Figure 174. ^{13}C NMR spectrum of compound **1b**

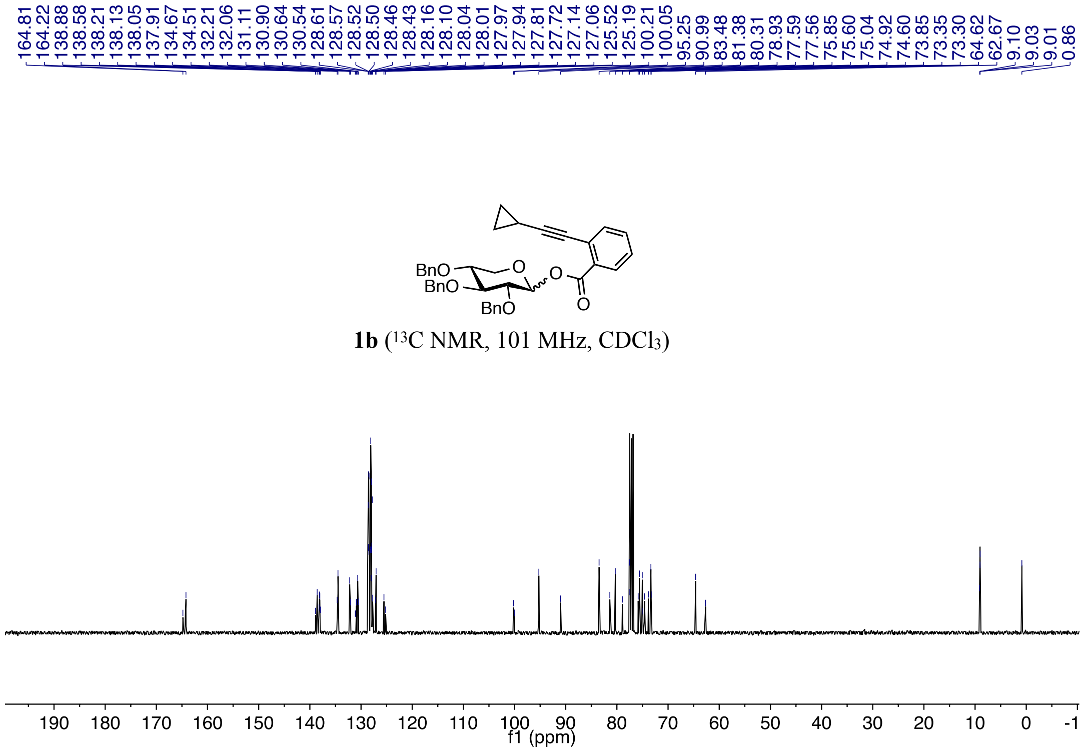

Supplementary Figure 175. ^{1}H NMR spectrum of compound **1c**

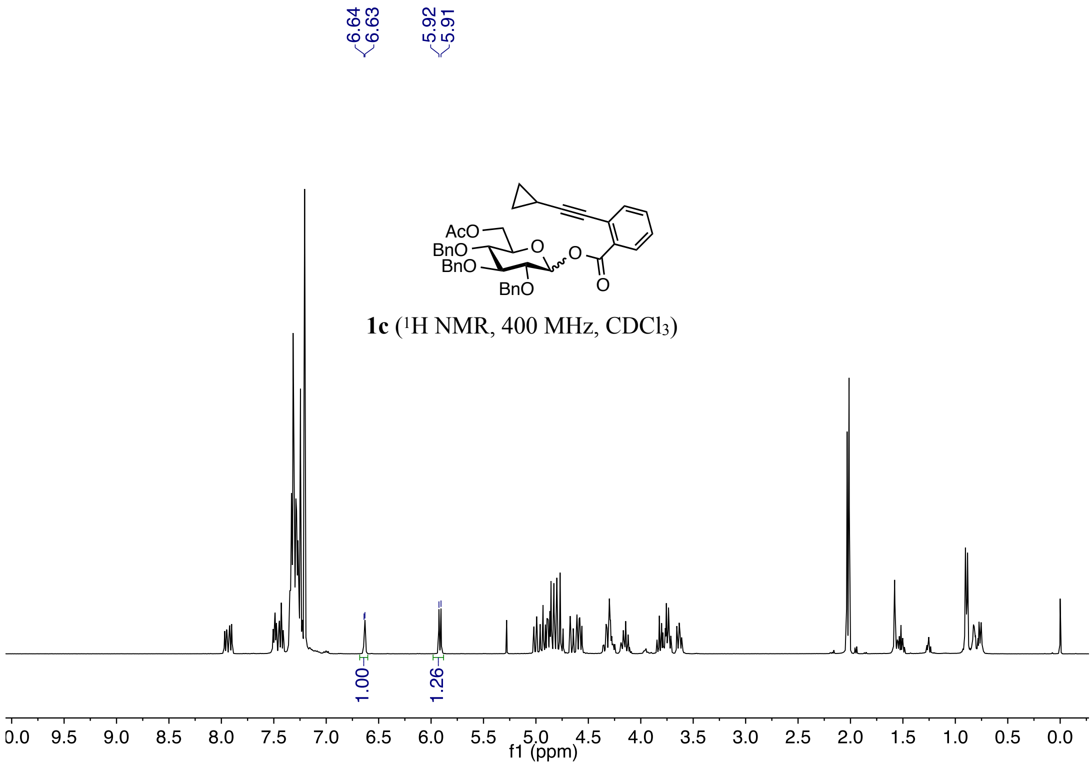

Supplementary Figure 176. ^{13}C NMR spectrum of compound **1c**

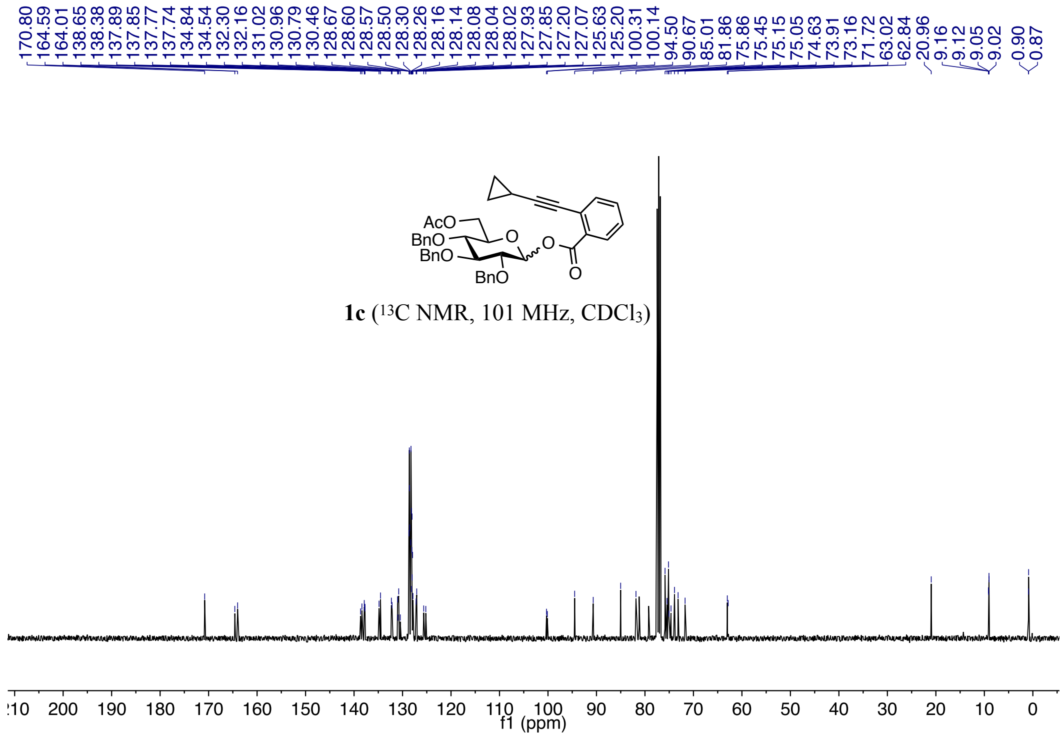

Supplementary Figure 177. ^{1}H NMR spectrum of compound **1d**

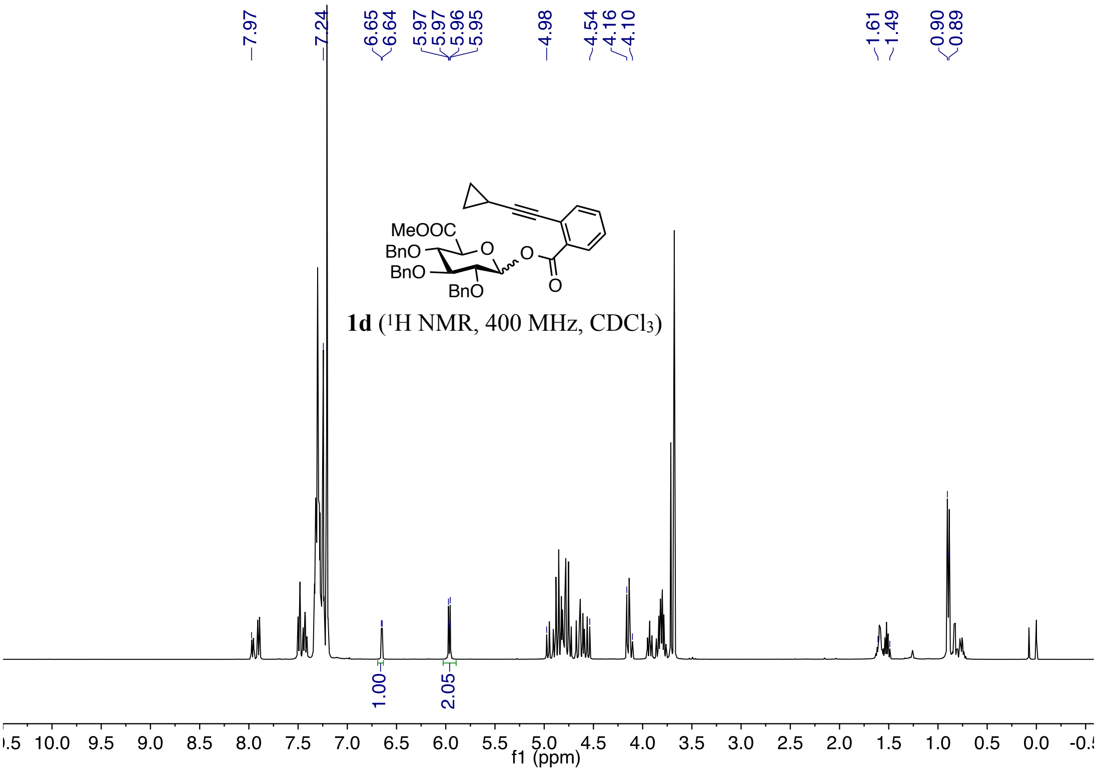

Supplementary Figure 178. ^{13}C NMR spectrum of compound **1d**

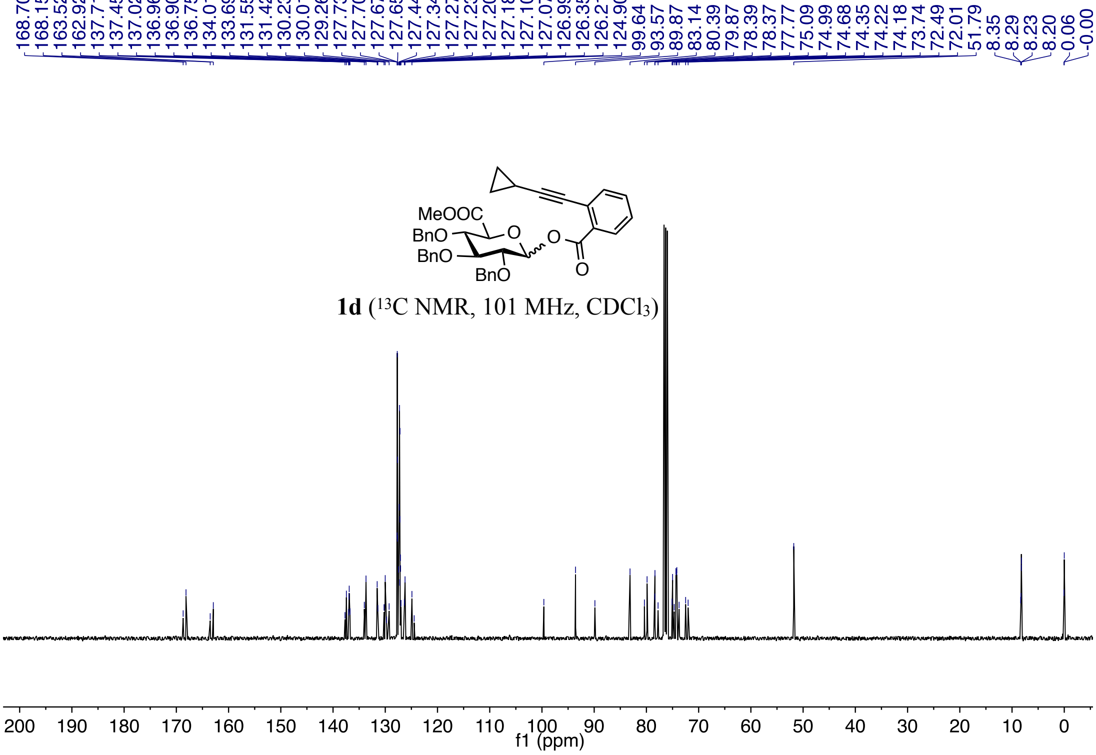

Supplementary Figure 179. ^{1}H NMR spectrum of compound **1eα**

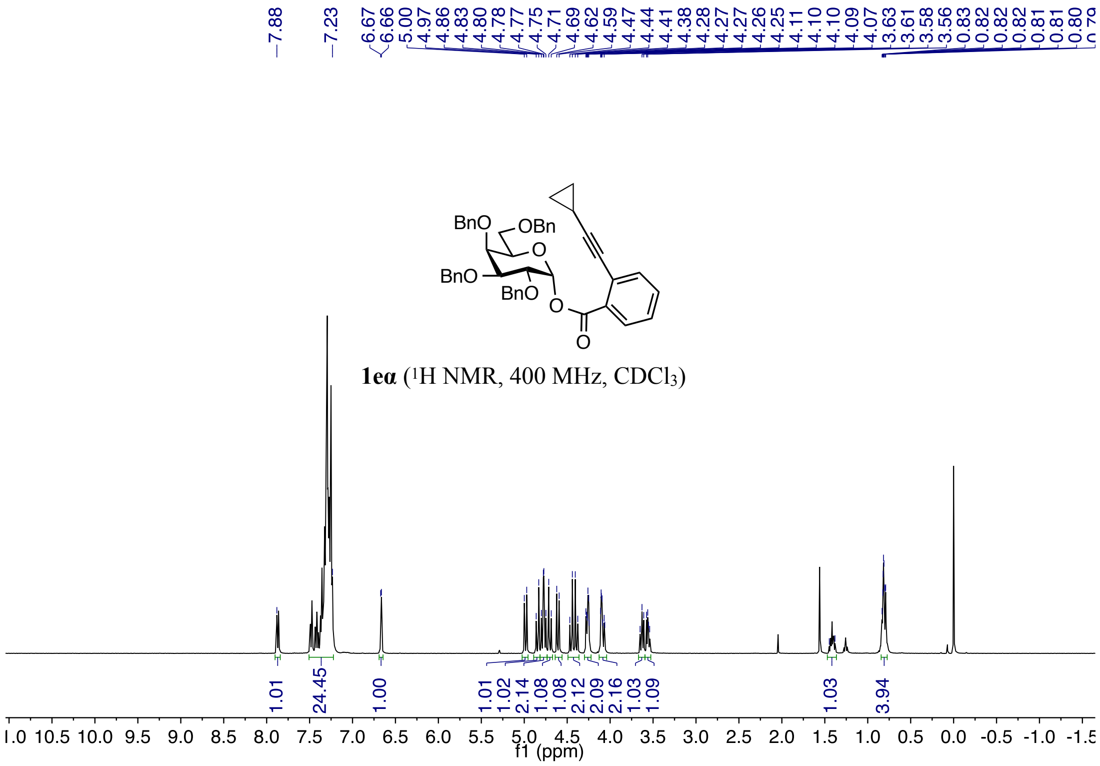

Supplementary Figure 180. ^{13}C NMR spectrum of compound **1eα**

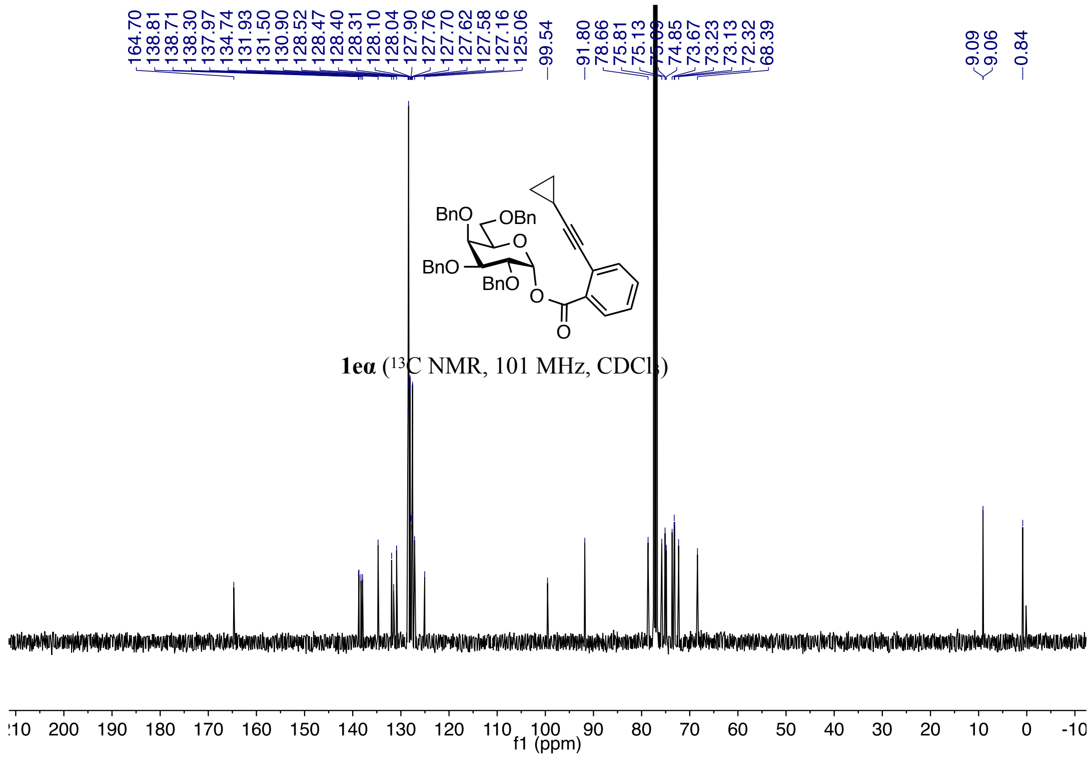

Supplementary Figure 181. ^{1}H NMR spectrum of compound **1eβ**

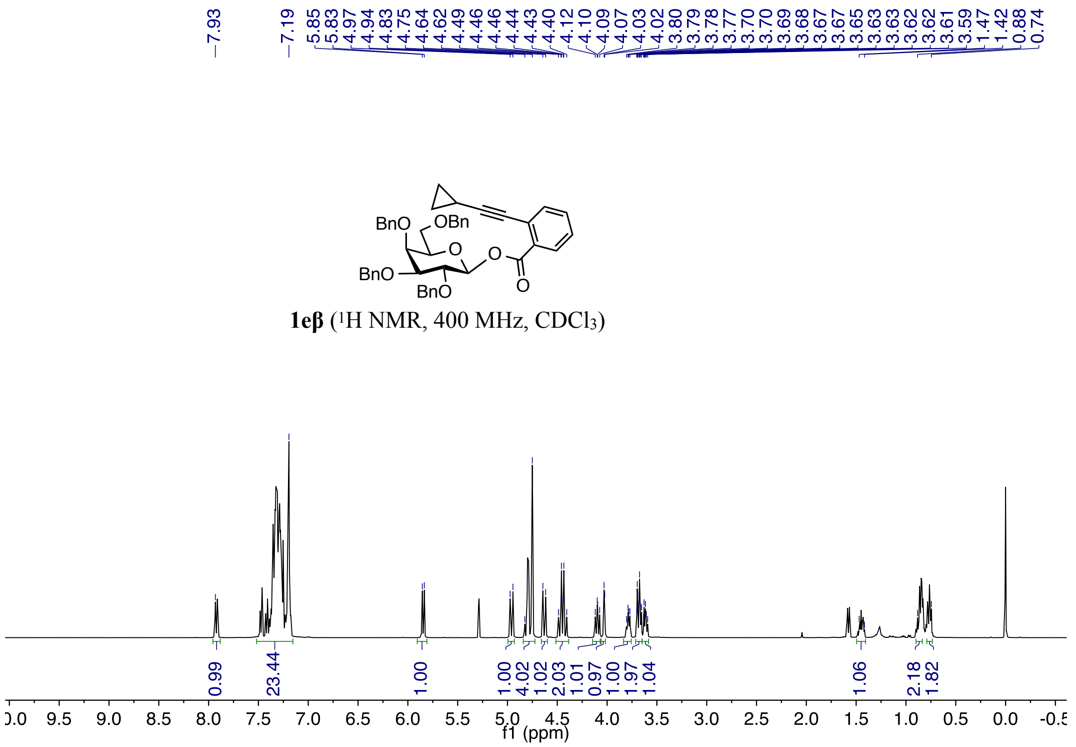

Supplementary Figure 182. ^{13}C NMR spectrum of compound **1eβ**

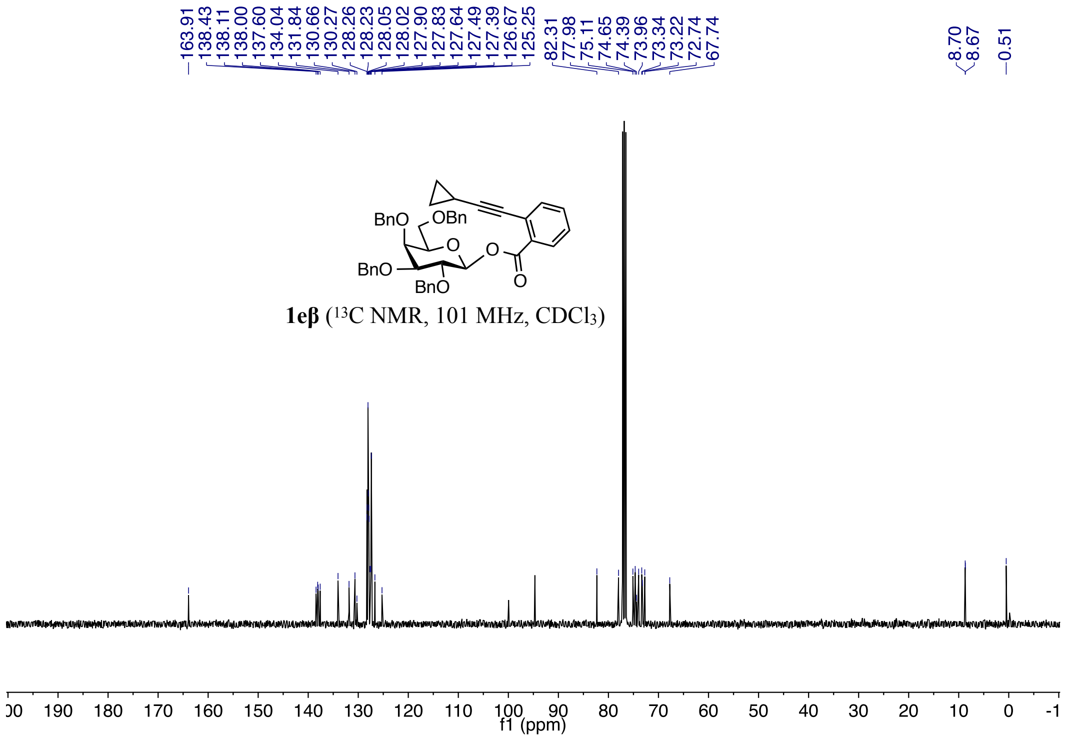

Supplementary Figure 183. ^{1}H NMR spectrum of compound **1f**

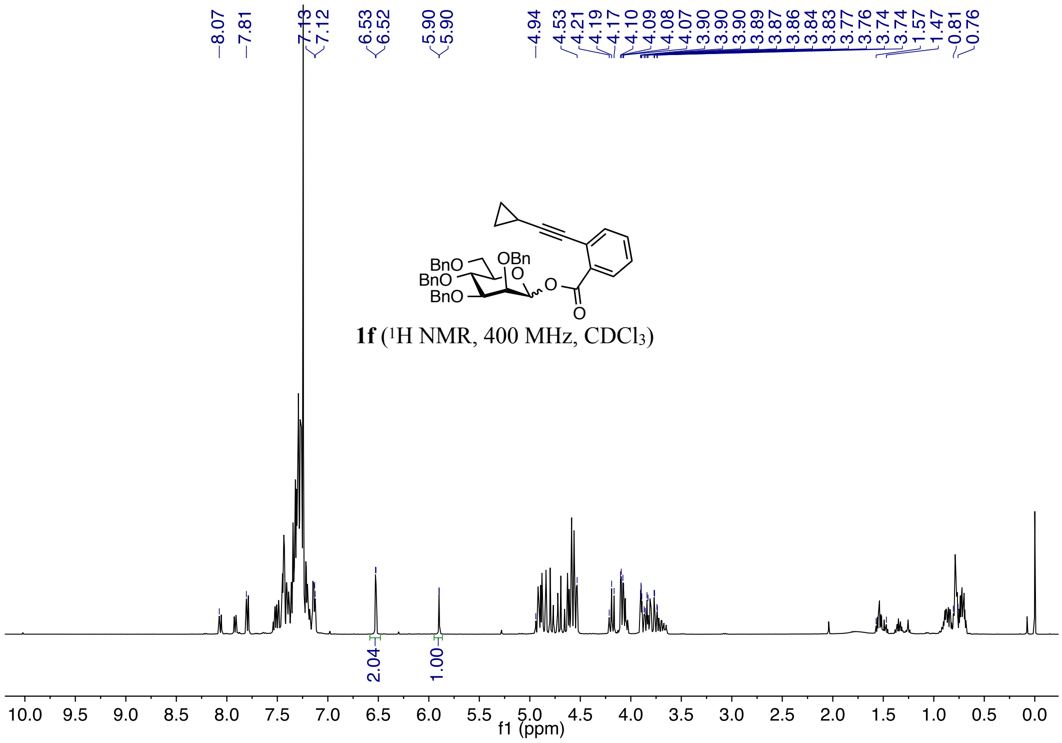

Supplementary Figure 184. ^{13}C NMR spectrum of compound **1f**

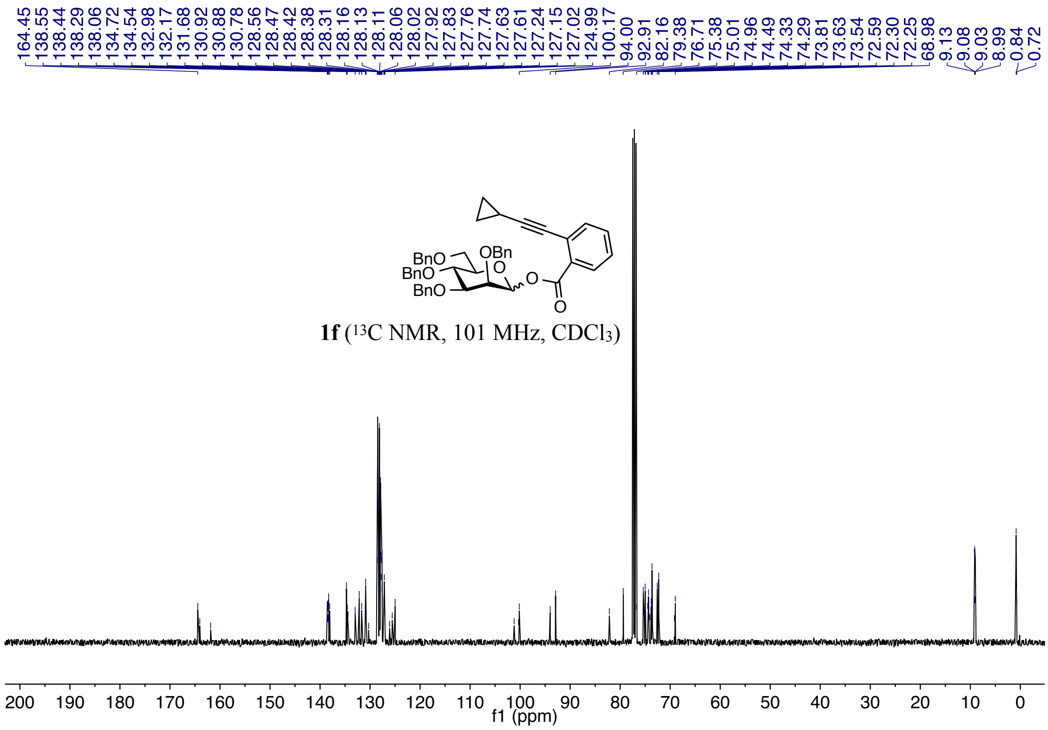

Supplementary Figure 185. ^{1}H NMR spectrum of compound **1gα**

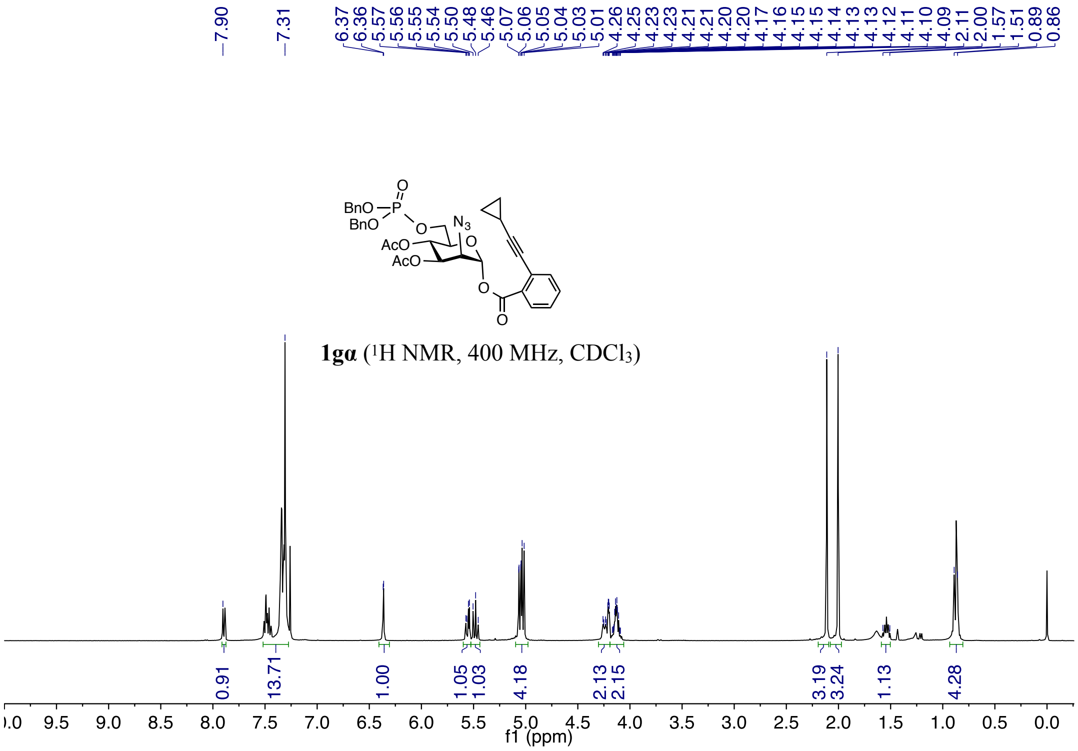

Supplementary Figure 186. ^{13}C NMR spectrum of compound **1gα**

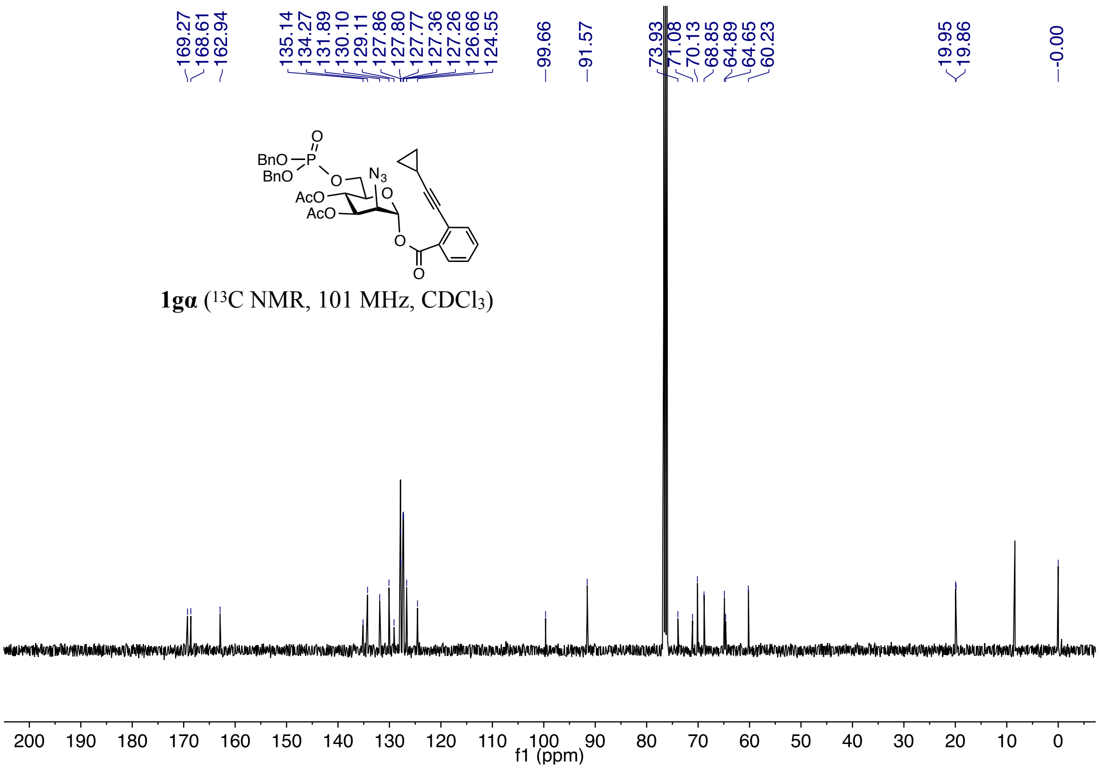

Supplementary Figure 187. ^{31}P NMR spectrum of compound **1gα**

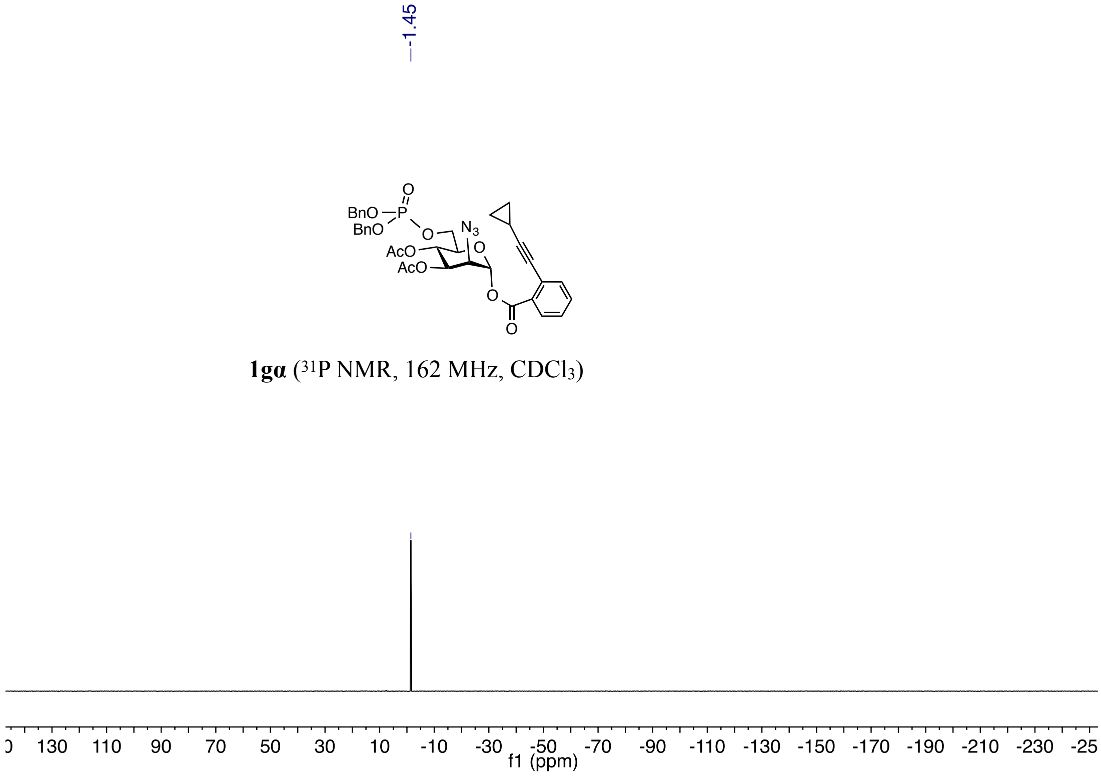

Supplementary Figure 188. ^{1}H NMR spectrum of compound **1gβ**

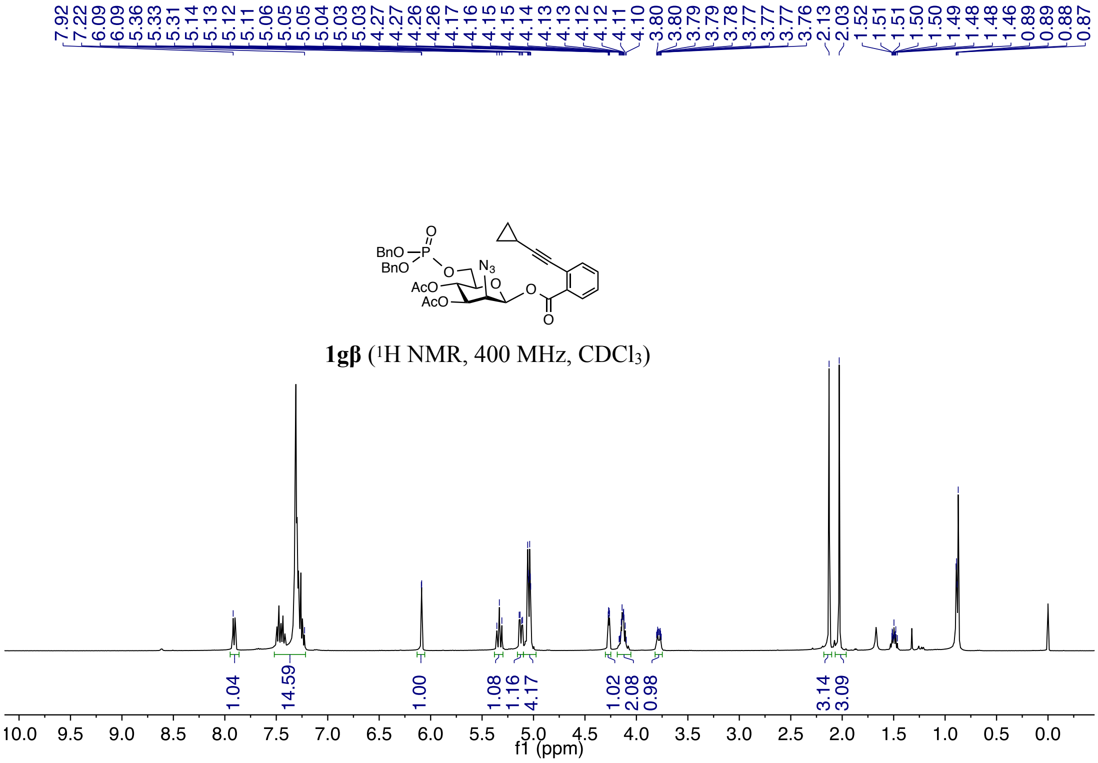

Supplementary Figure 189. ^{13}C NMR spectrum of compound **1gβ**

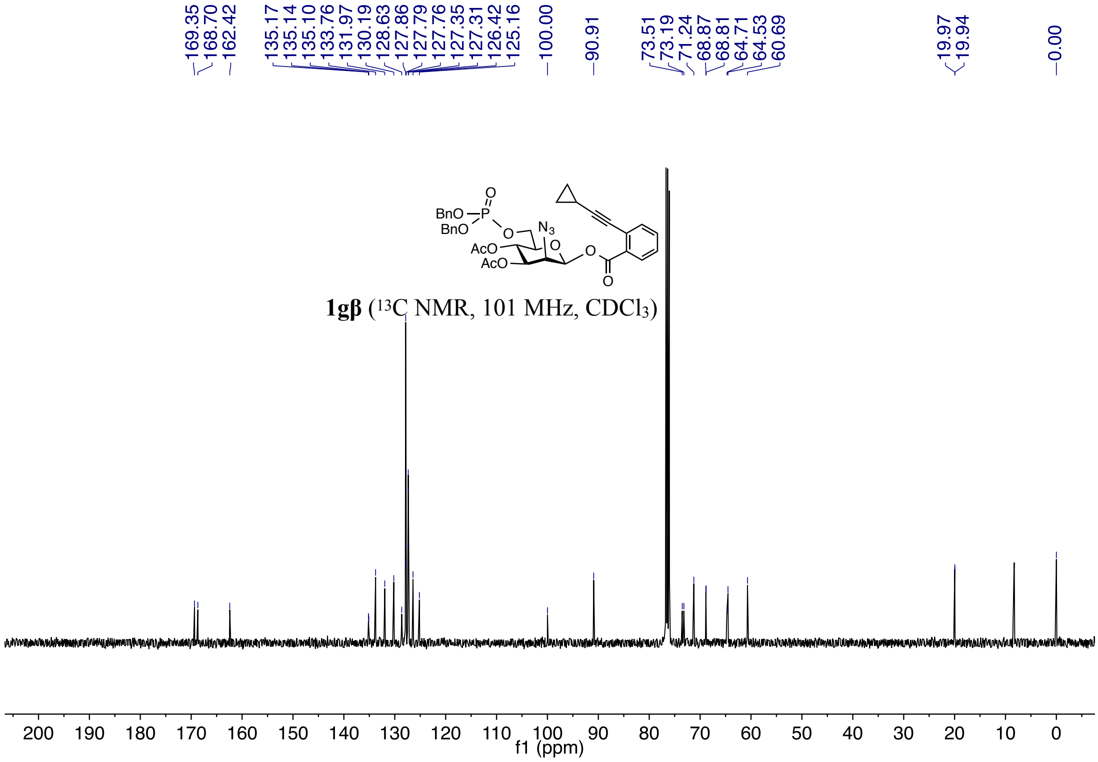

Supplementary Figure 190. ^{31}P NMR spectrum of compound **1gβ**

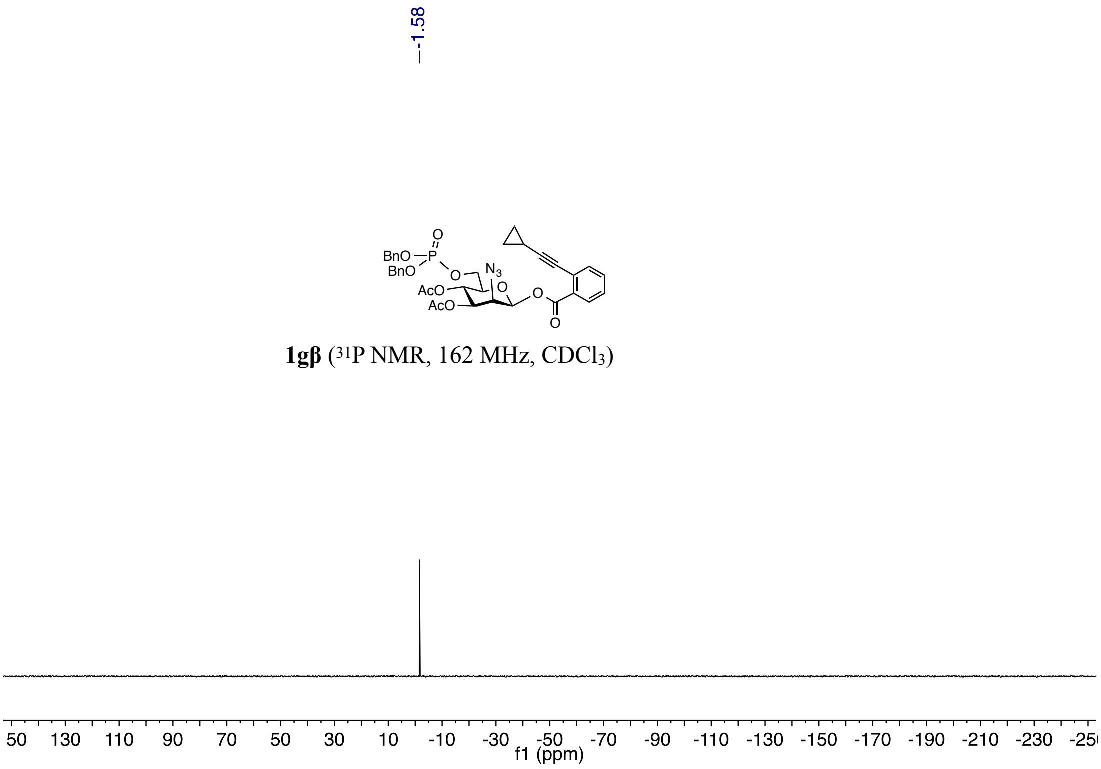

Supplementary Figure 191. ^{1}H NMR spectrum of compound **1h**

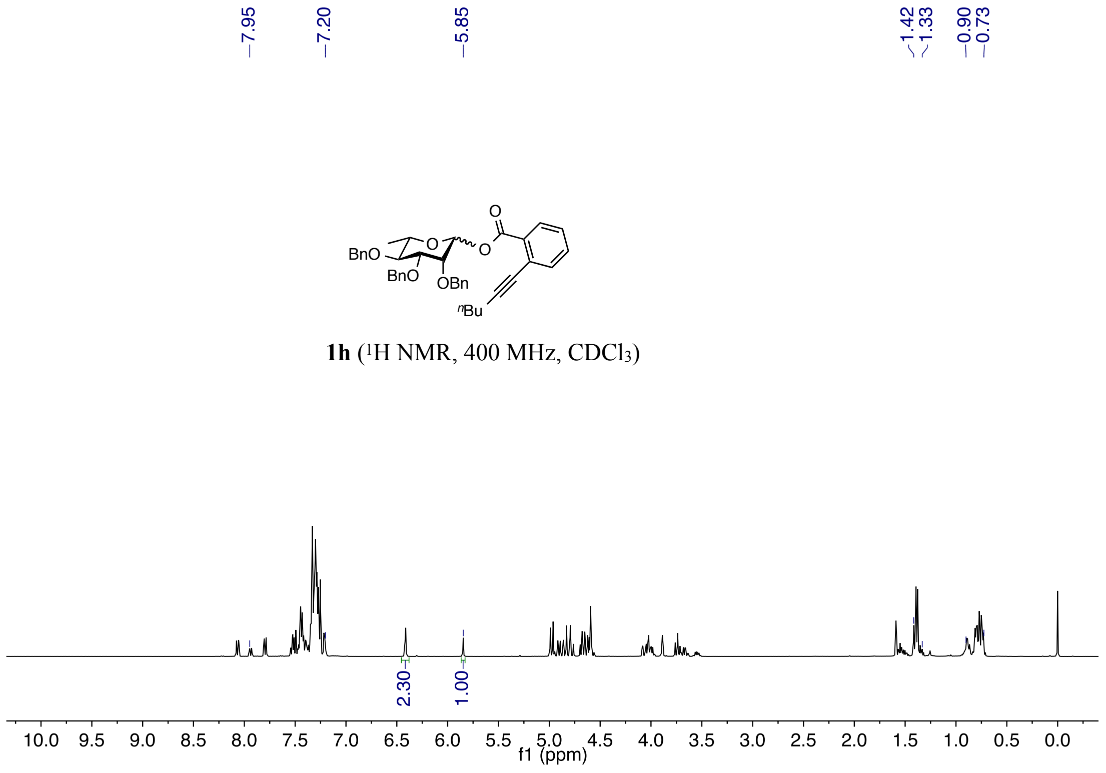

Supplementary Figure 192. ^{13}C NMR spectrum of compound **1h**

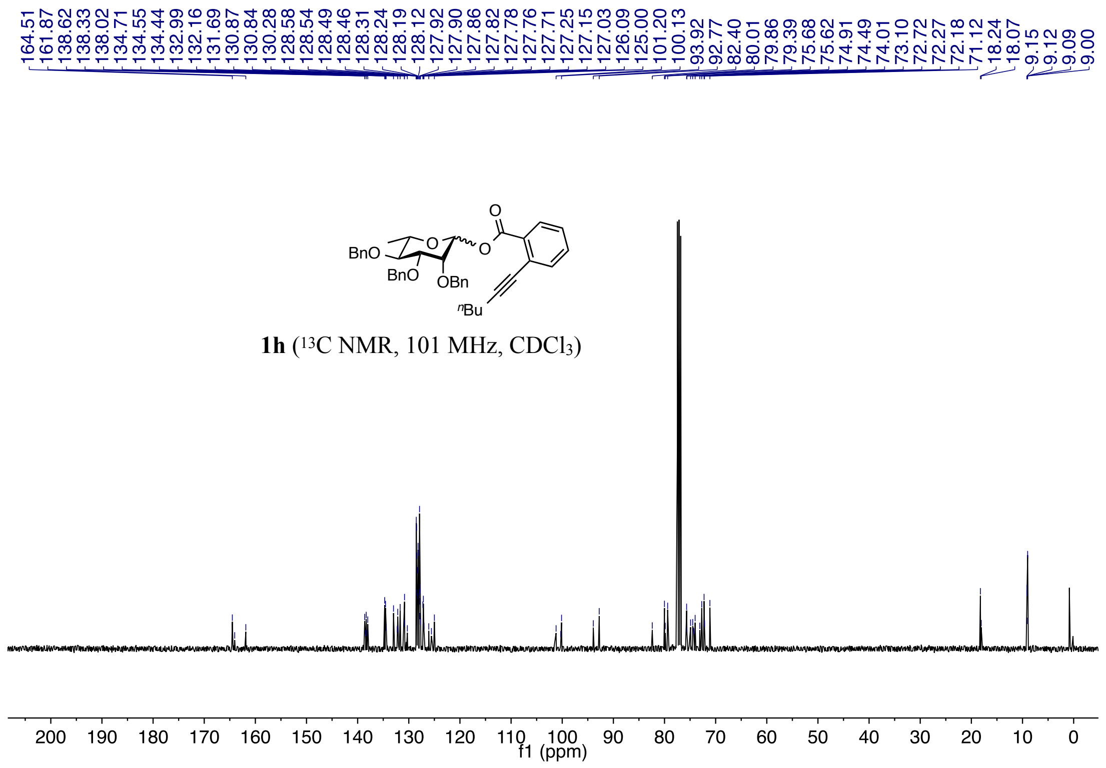

Supplementary Figure 193. ^{1}H NMR spectrum of compound **1i**

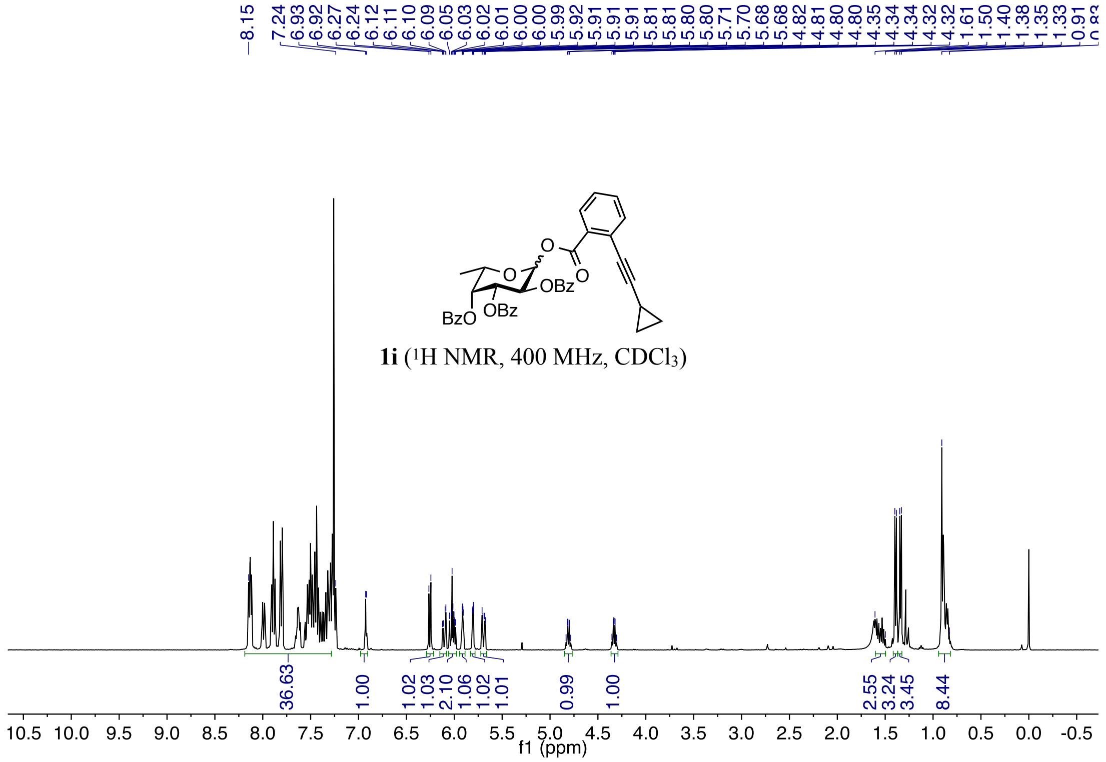

Supplementary Figure 194. ^{13}C NMR spectrum of compound **1i**

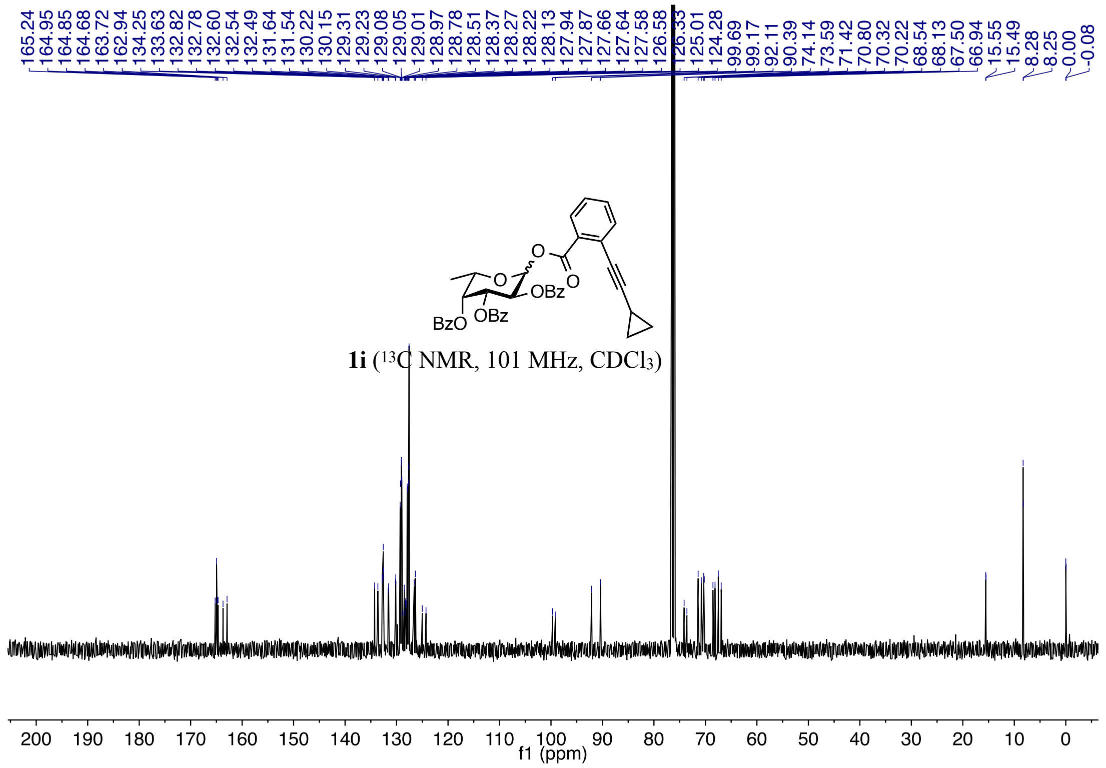

Supplementary Figure 195. ^{1}H NMR spectrum of compound **1j**

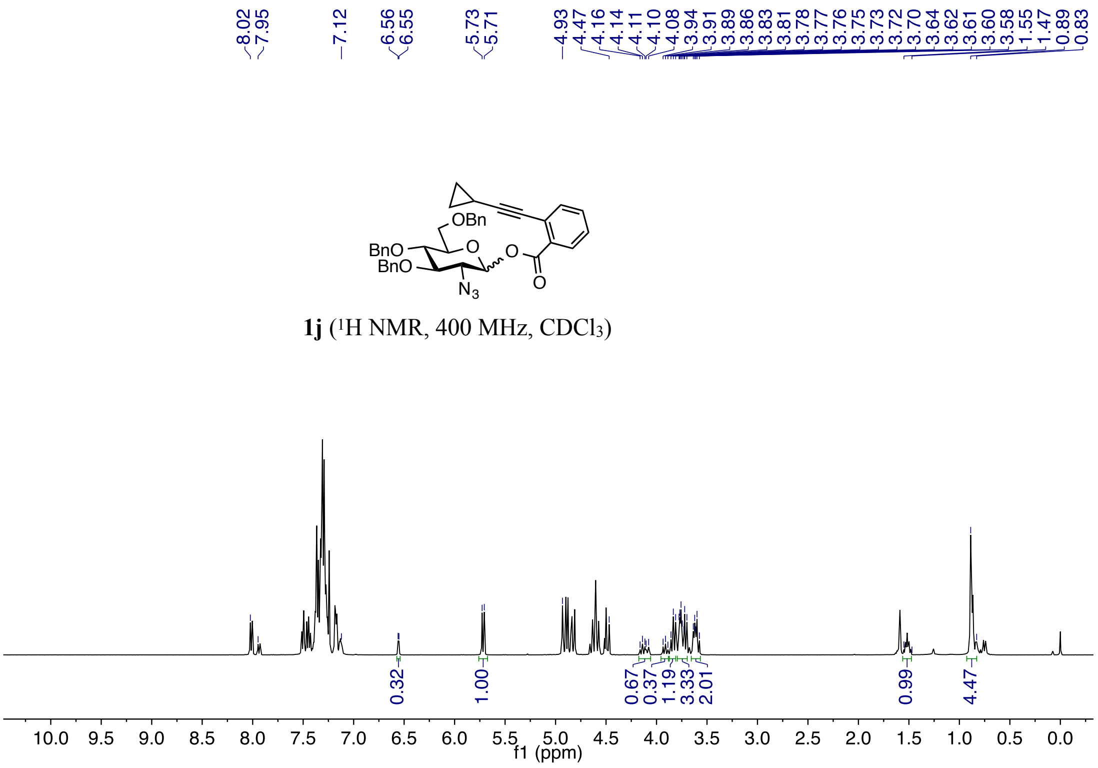

Supplementary Figure 196. ^{13}C NMR spectrum of compound **1j**

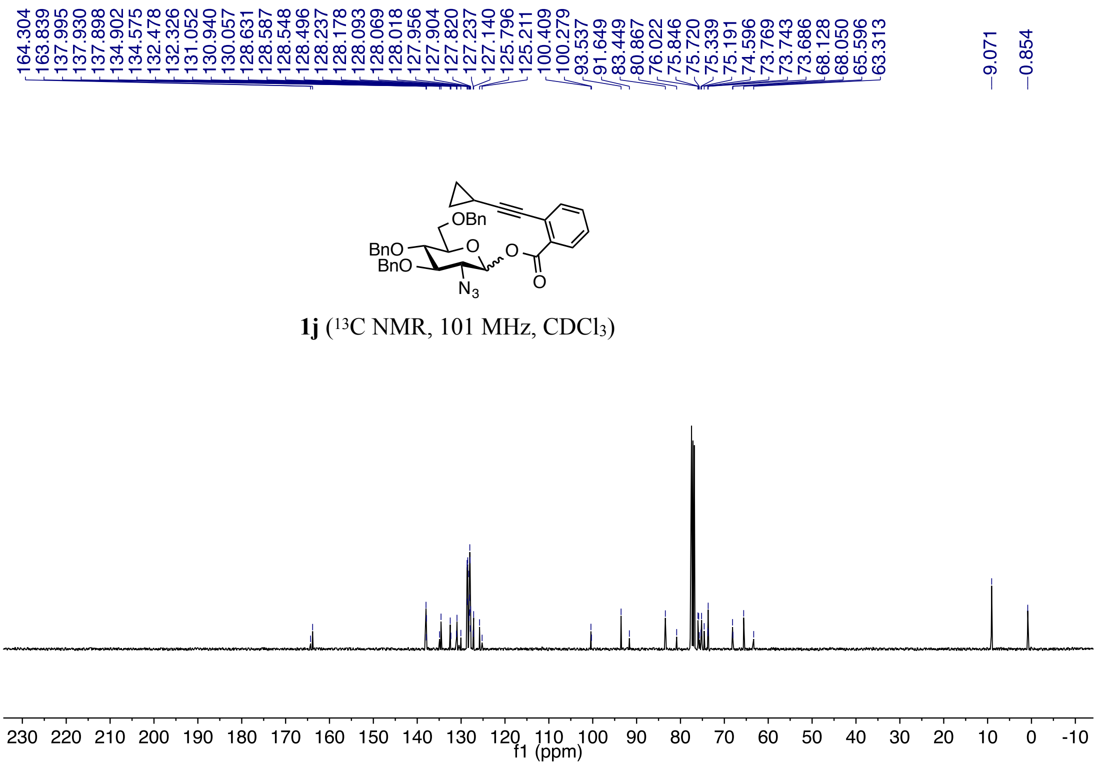

Supplementary Figure 197. ^{1}H NMR spectrum of compound **1k**

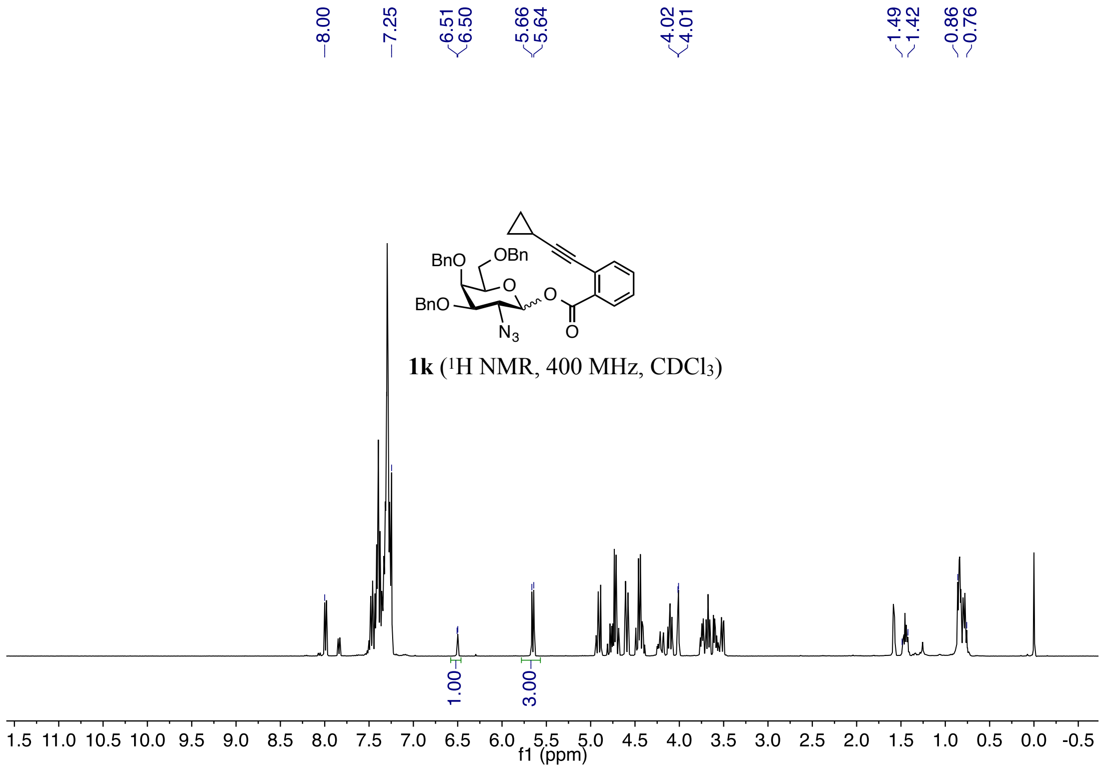

Supplementary Figure 198. ^{13}C NMR spectrum of compound **1k**

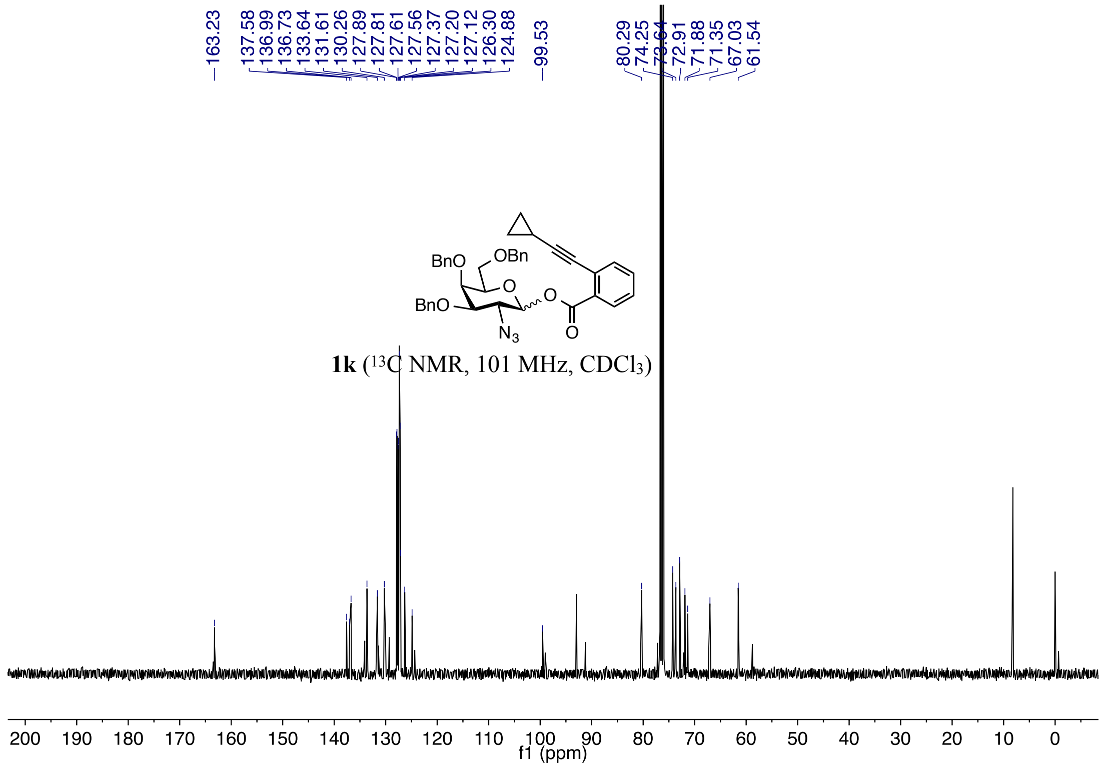

Supplementary Figure 199. ^{1}H NMR spectrum of compound **1l**

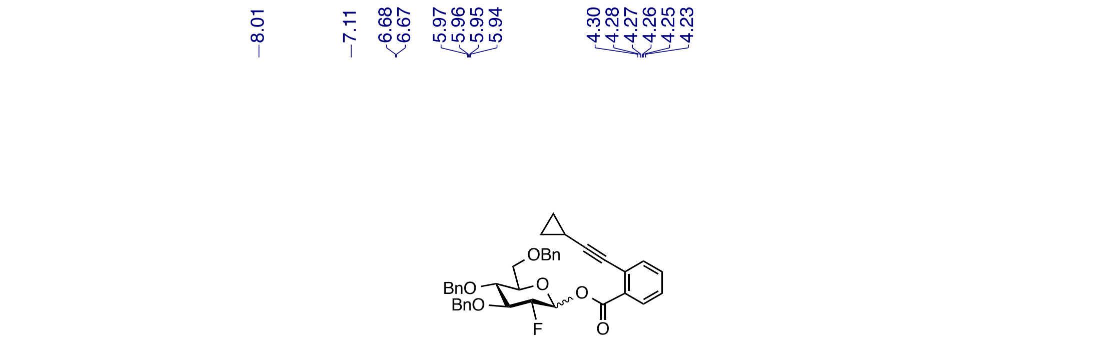

1l (^{1}H NMR, 400 MHz, $CDCl_3$)

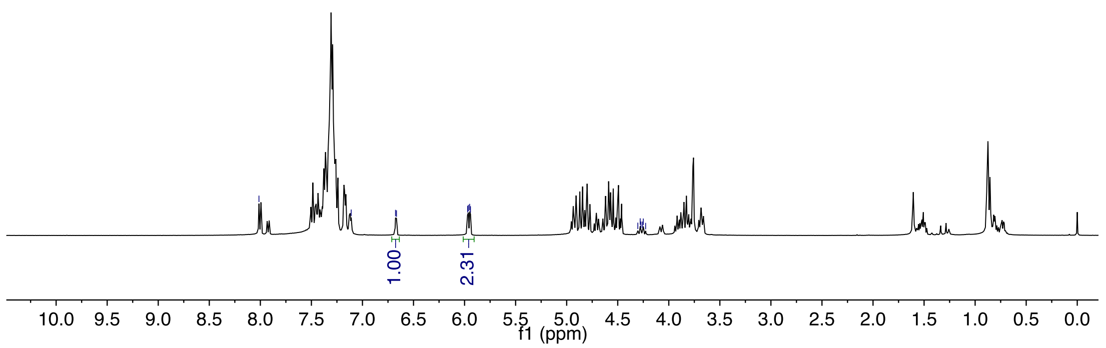

Supplementary Figure 200. ^{13}C NMR spectrum of compound **1l**

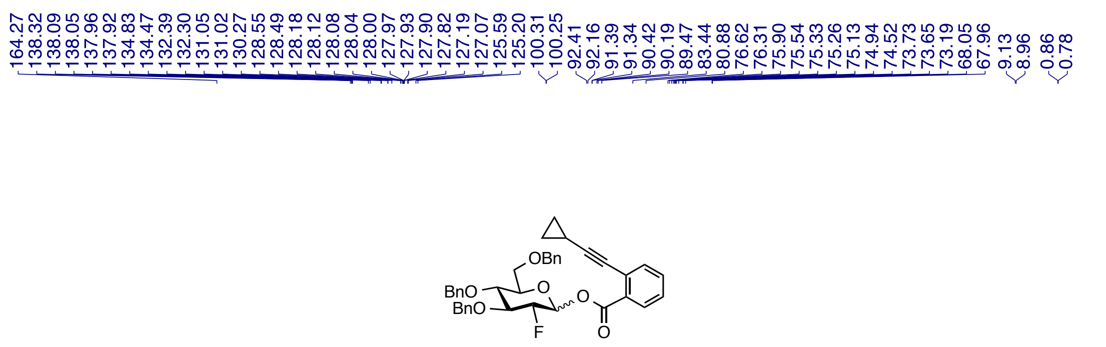

1l (^{13}C NMR, 101 MHz, $CDCl_3$)

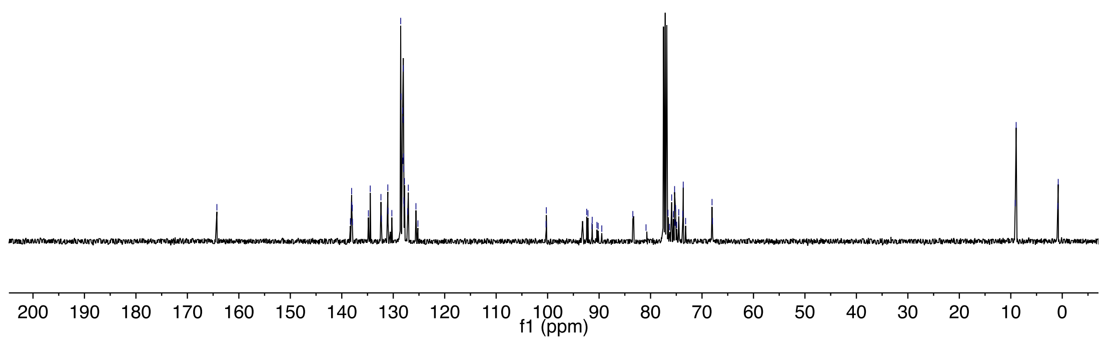

Supplementary Figure 201. ^{19}F NMR spectrum of compound **1l**

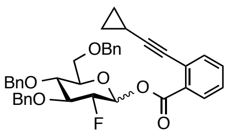

1l (^{19}F NMR)

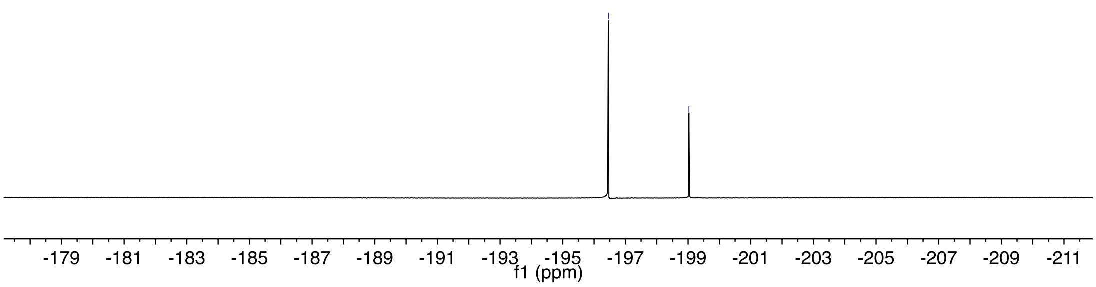

Supplementary Figure 202. ^{1}H NMR spectrum of compound **1m**

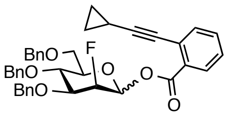

1m (^{1}H NMR, 400 MHz, $CDCl_3$)

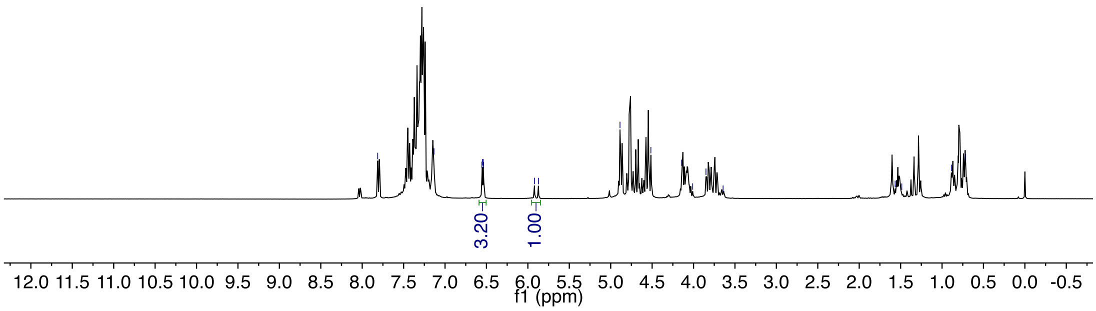

Supplementary Figure 203. ^{13}C NMR spectrum of compound **1m**

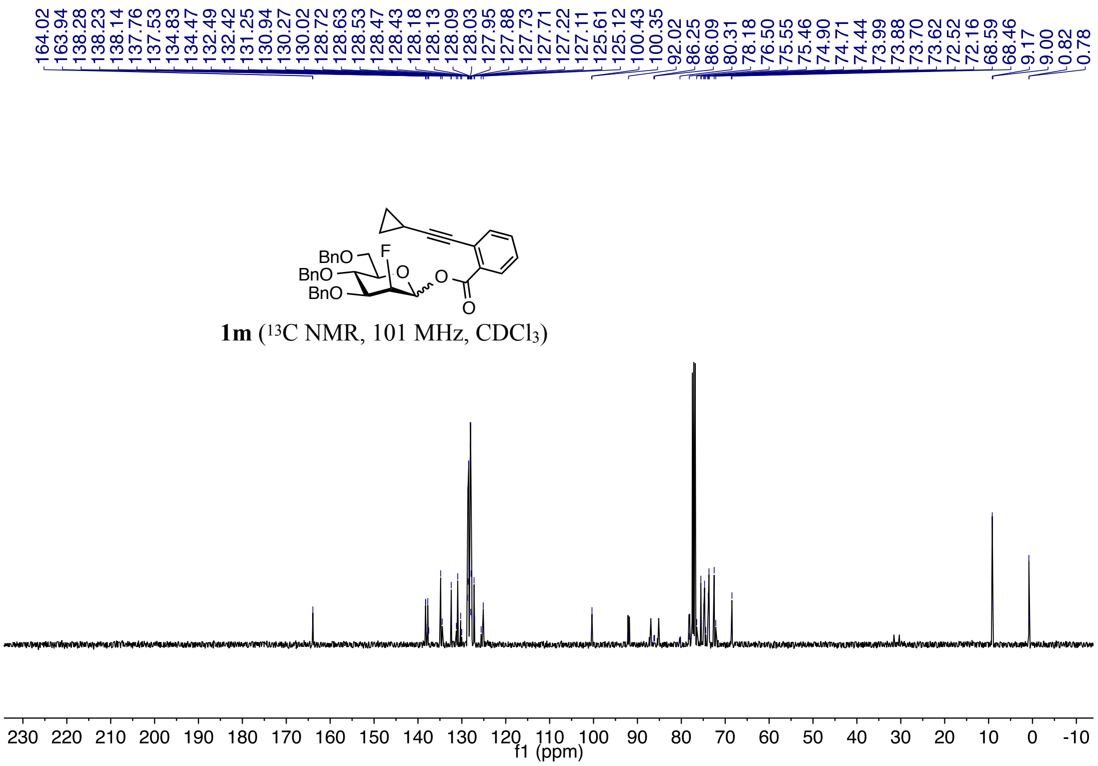

Supplementary Figure 204. ^{19}F NMR spectrum of compound **1m**

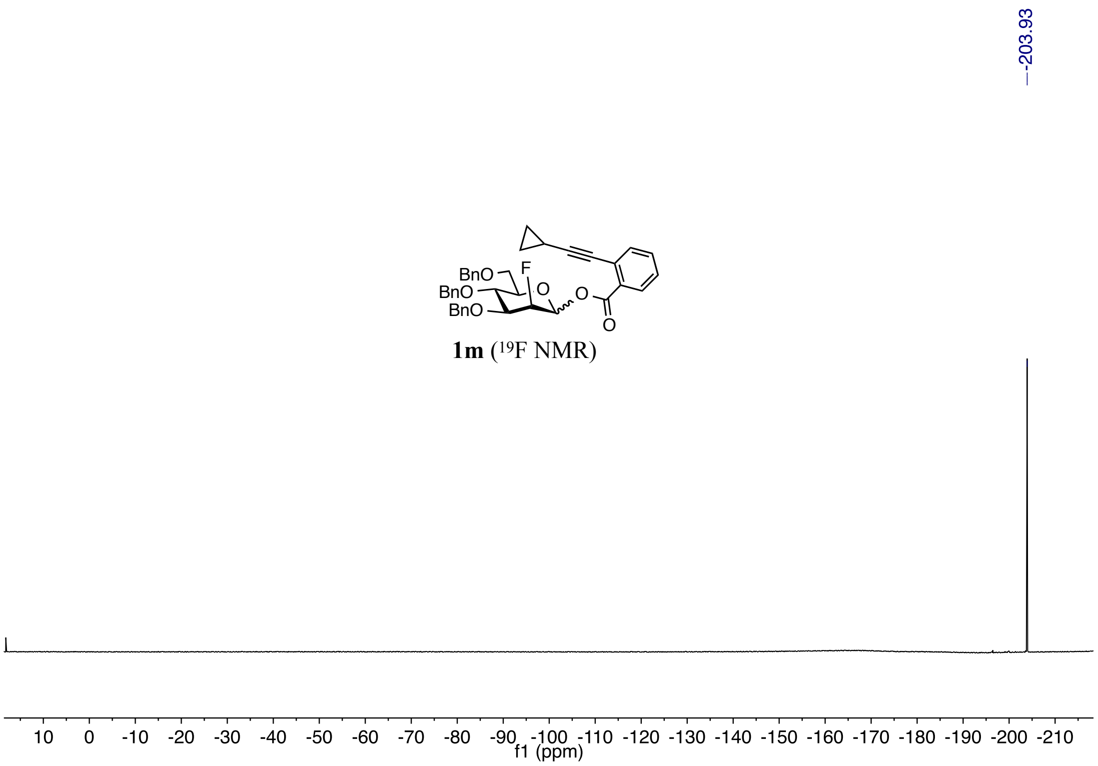

Supplementary Figure 205. ^{1}H NMR spectrum of compound **1nα**

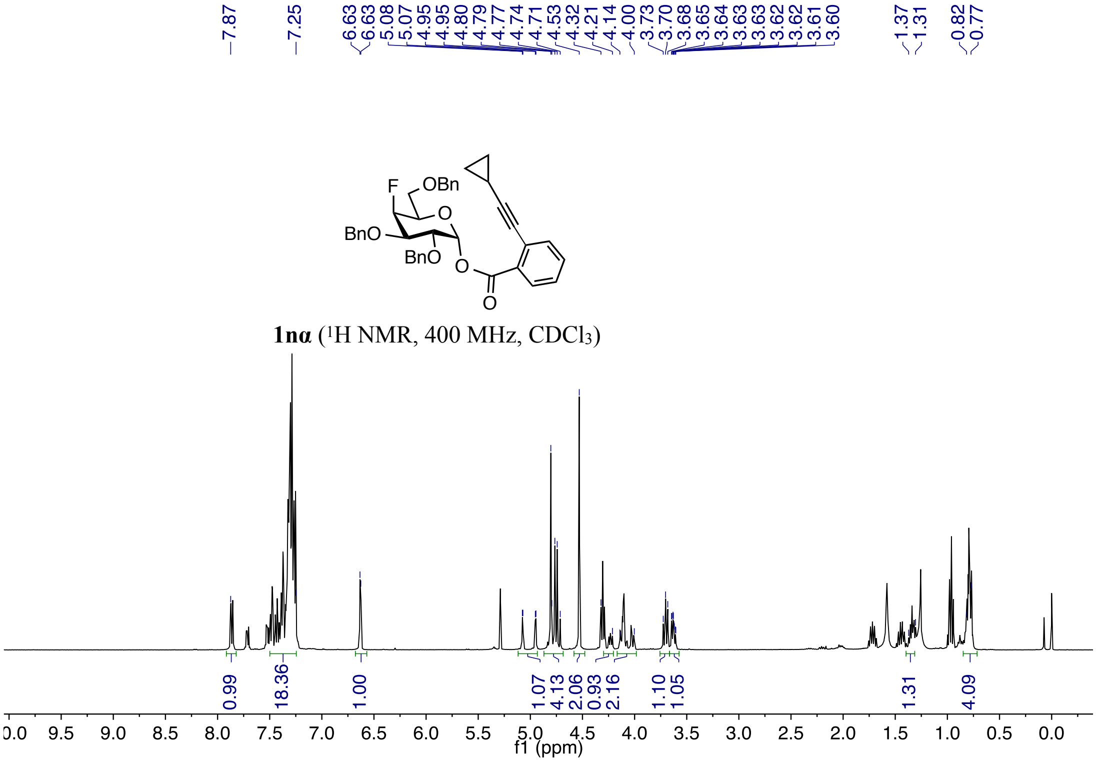

Supplementary Figure 206. ^{13}C NMR spectrum of compound **1nα**

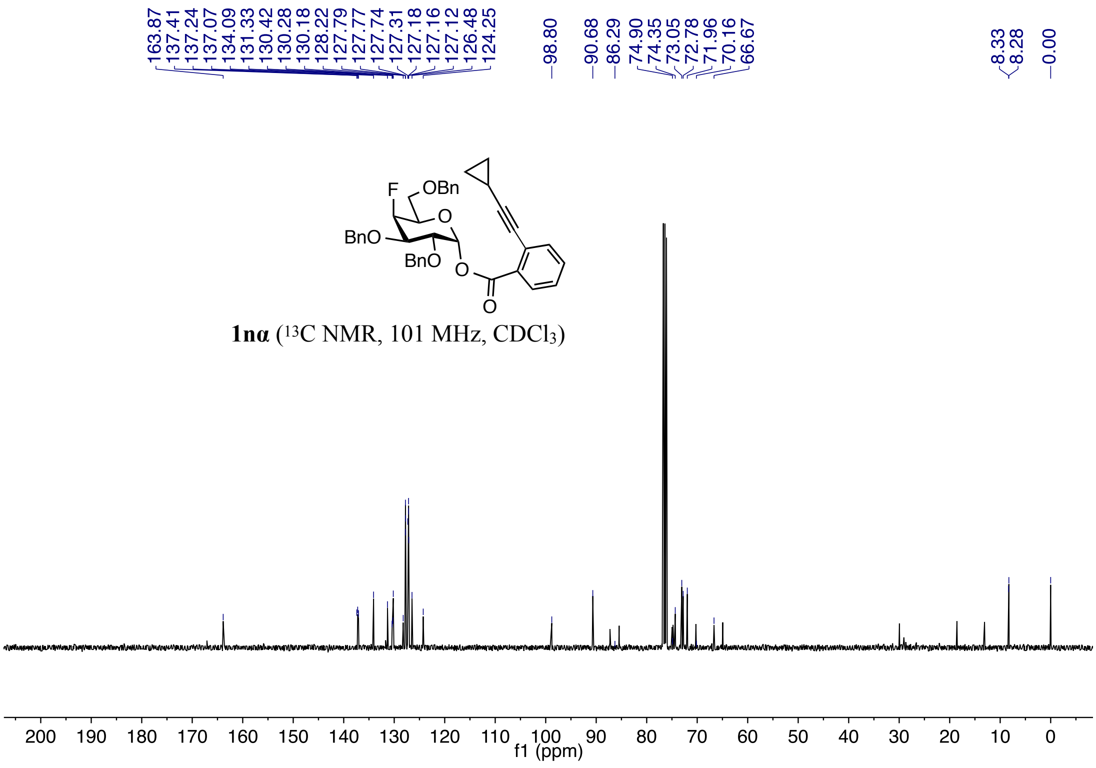

Supplementary Figure 207. ^{19}F NMR spectrum of compound **1nα**

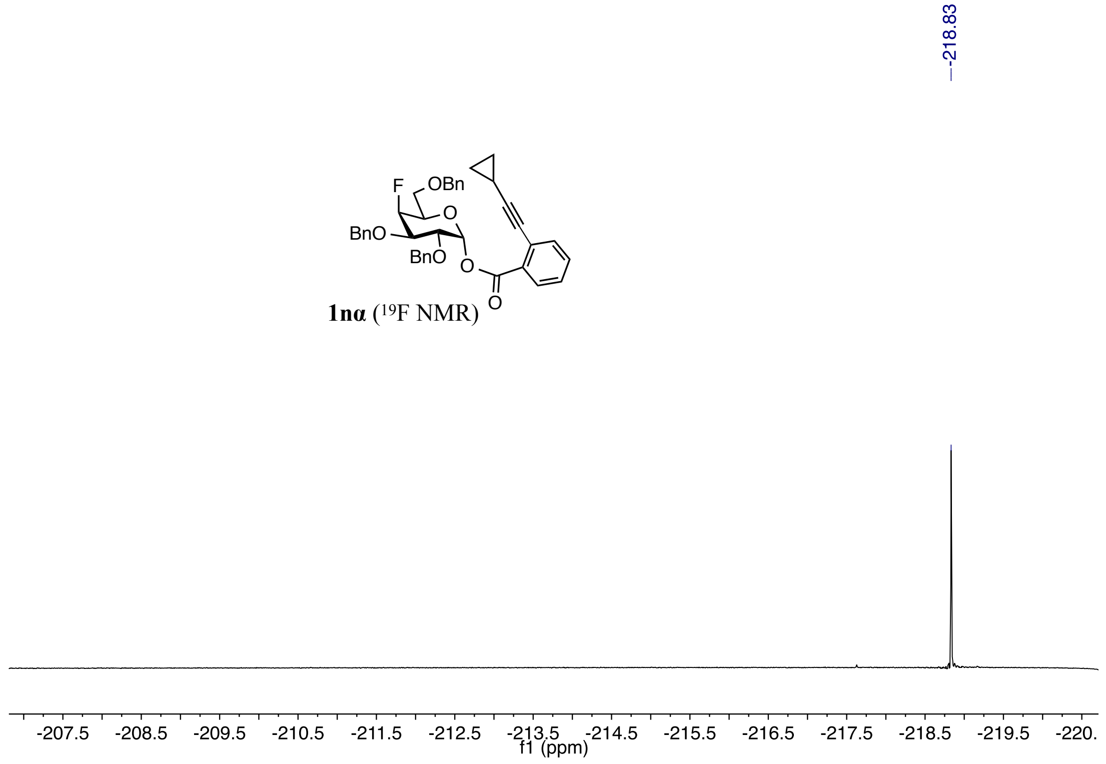

Supplementary Figure 208. ^{1}H NMR spectrum of compound **1nβ**

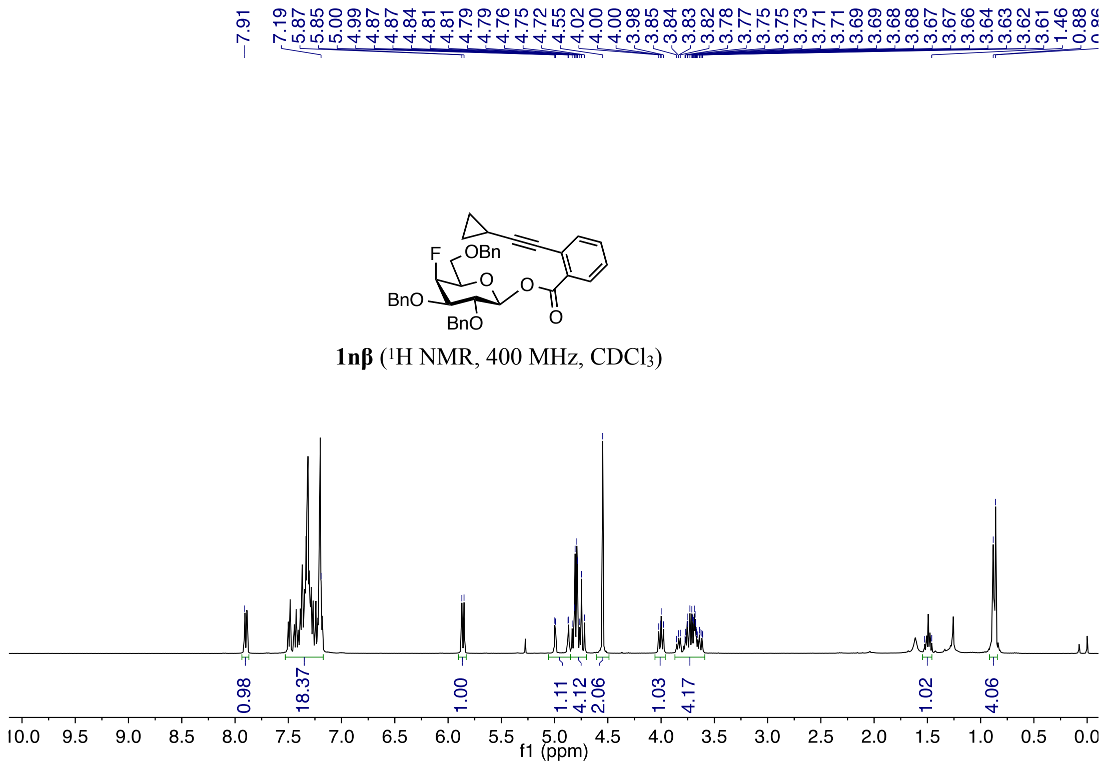

Supplementary Figure 209. ^{13}C NMR spectrum of compound **1nβ**

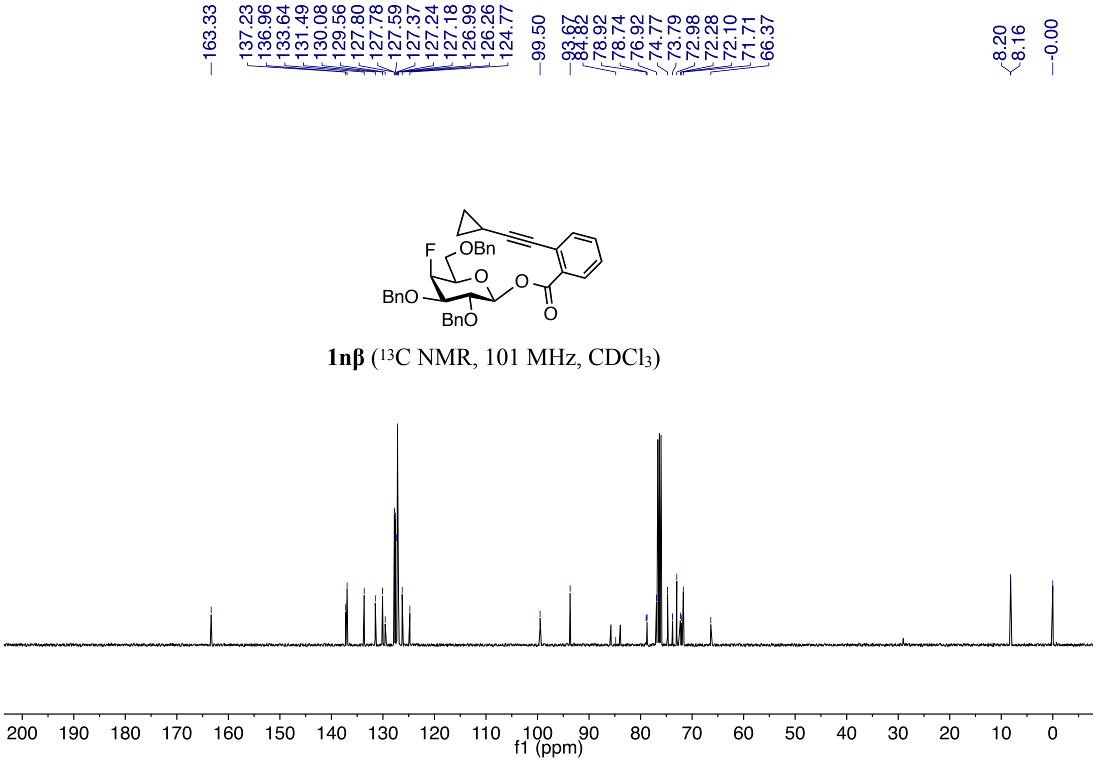

Supplementary Figure 210. ^{19}F NMR spectrum of compound **1nβ**

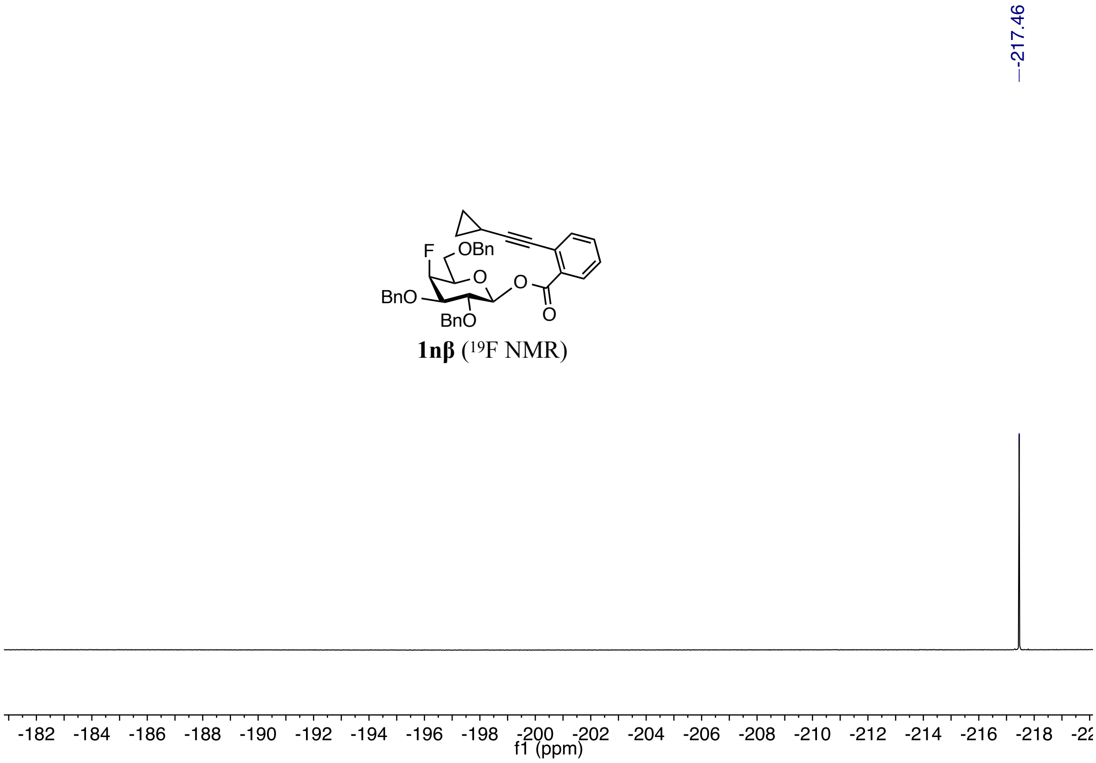

Supplementary Figure 211. ^{1}H NMR spectrum of compound **1o**

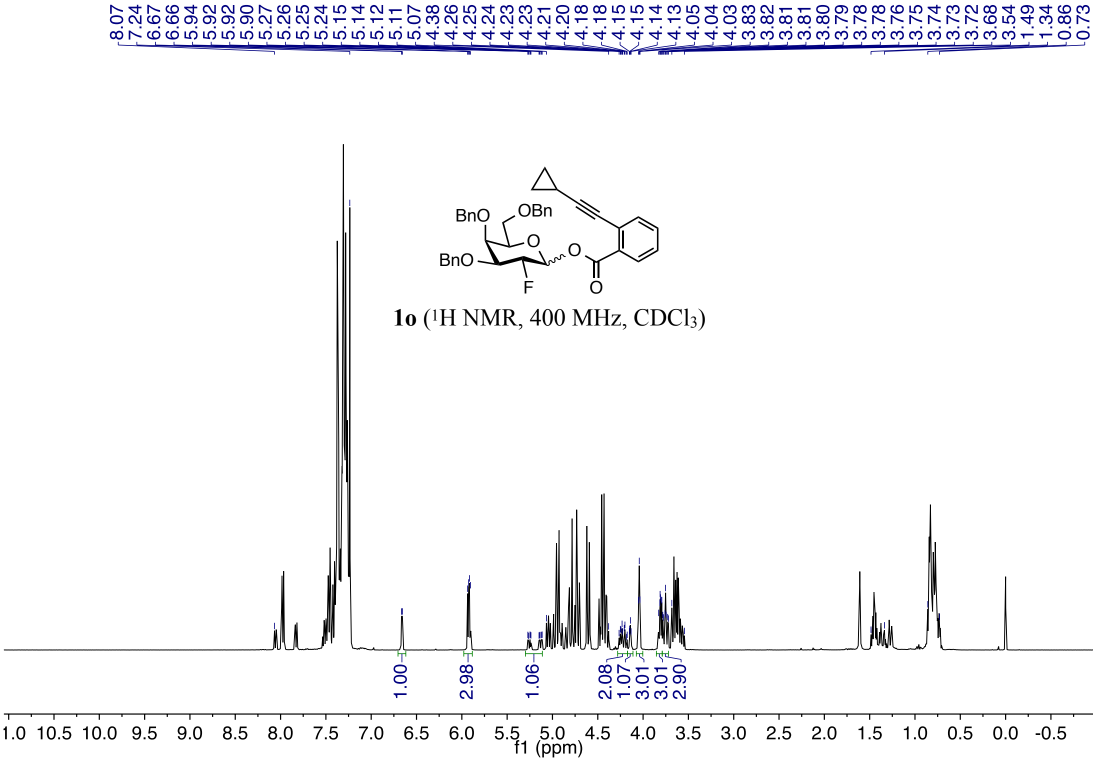

Supplementary Figure 212. ^{13}C NMR spectrum of compound **1o**

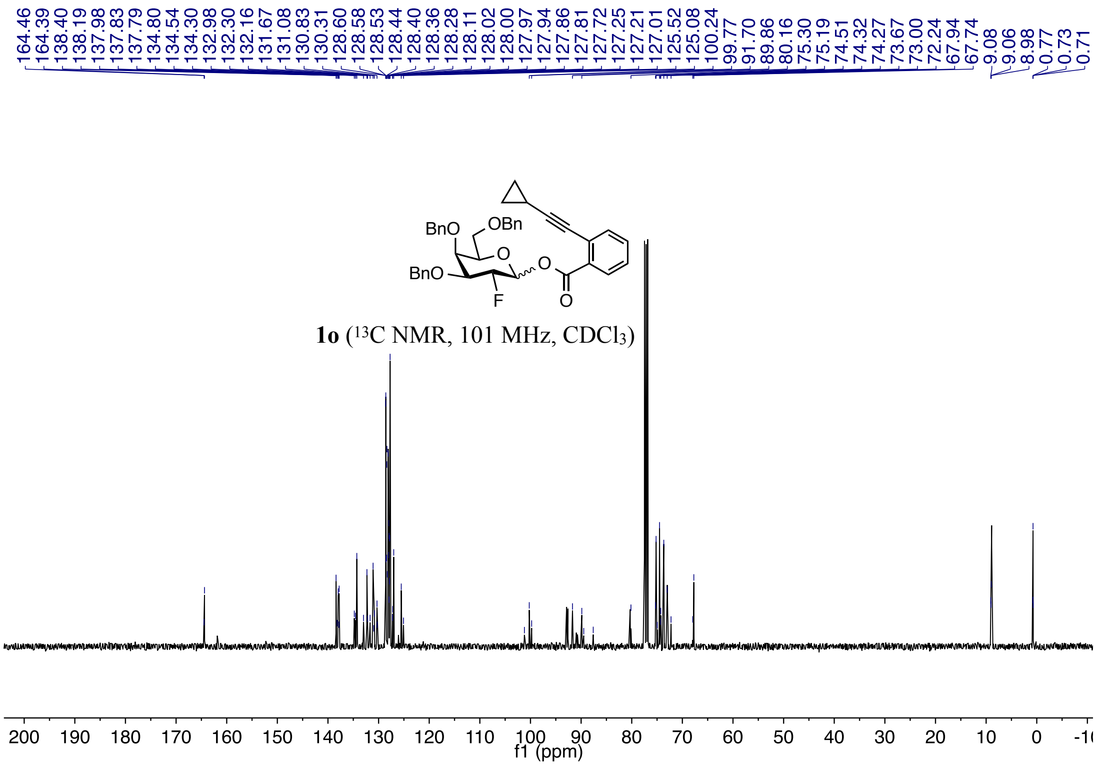

Supplementary Figure 213. ^{19}F NMR spectrum of compound **1o**

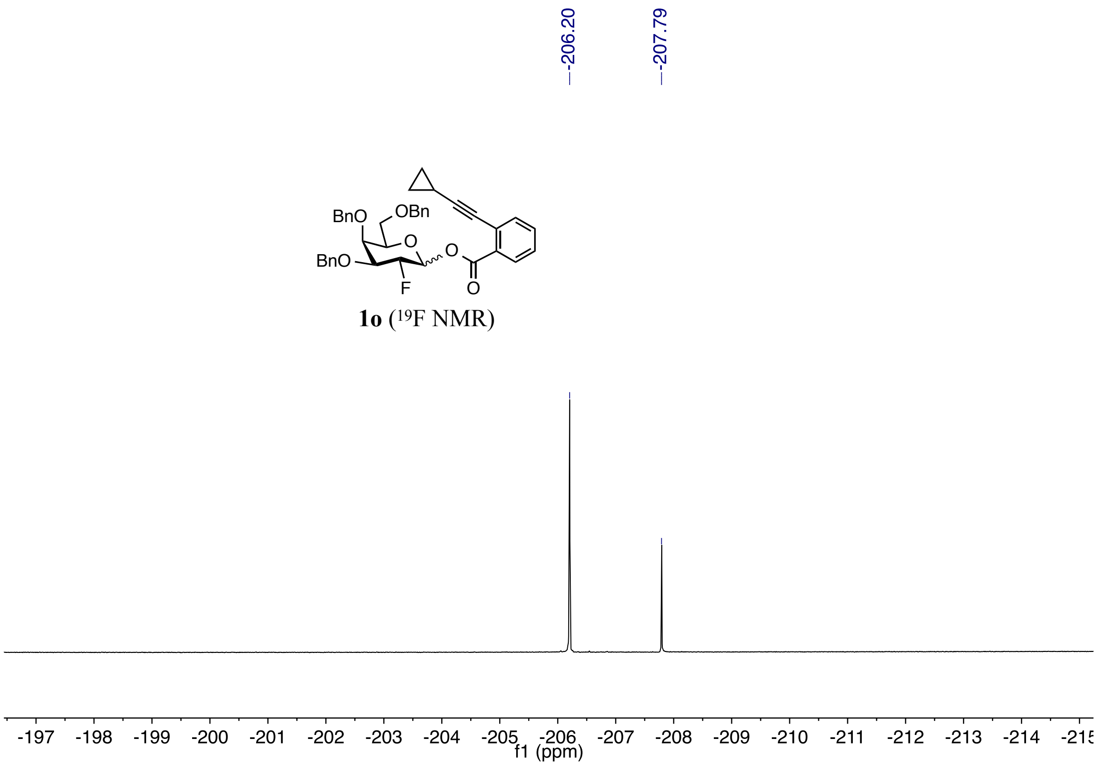

Supplementary Figure 214. ^{1}H NMR spectrum of compound **S1**

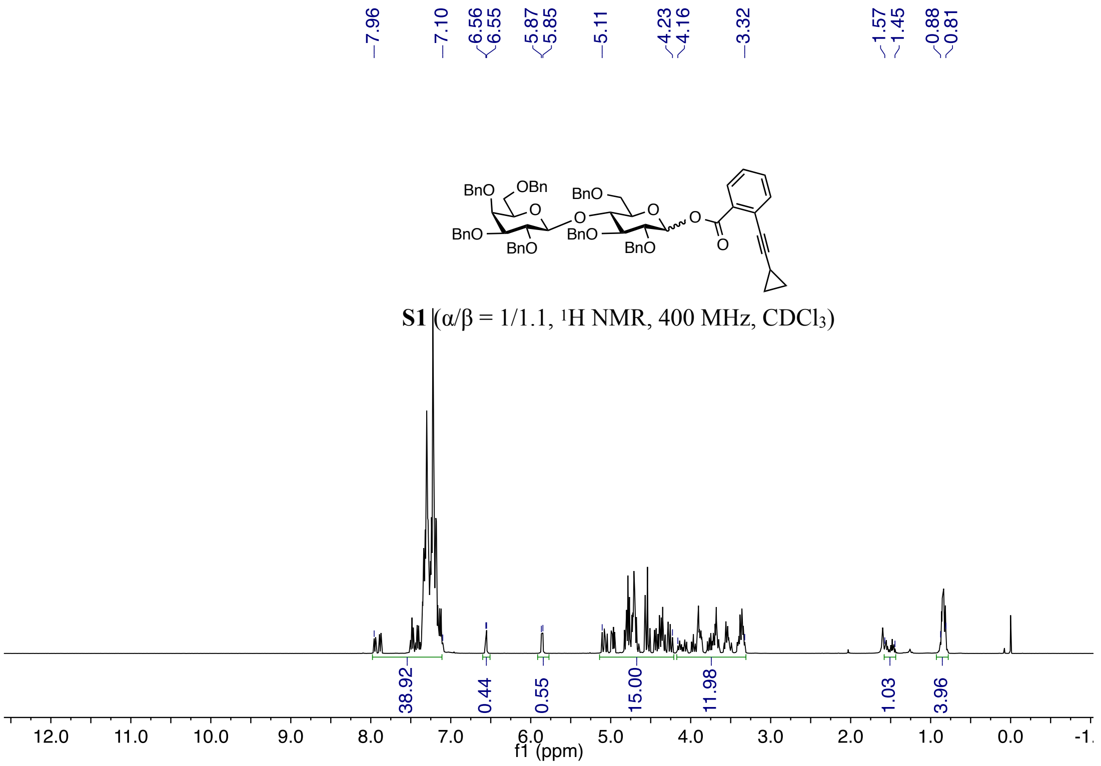

Supplementary Figure 215. ^{13}C NMR spectrum of compound **S1**

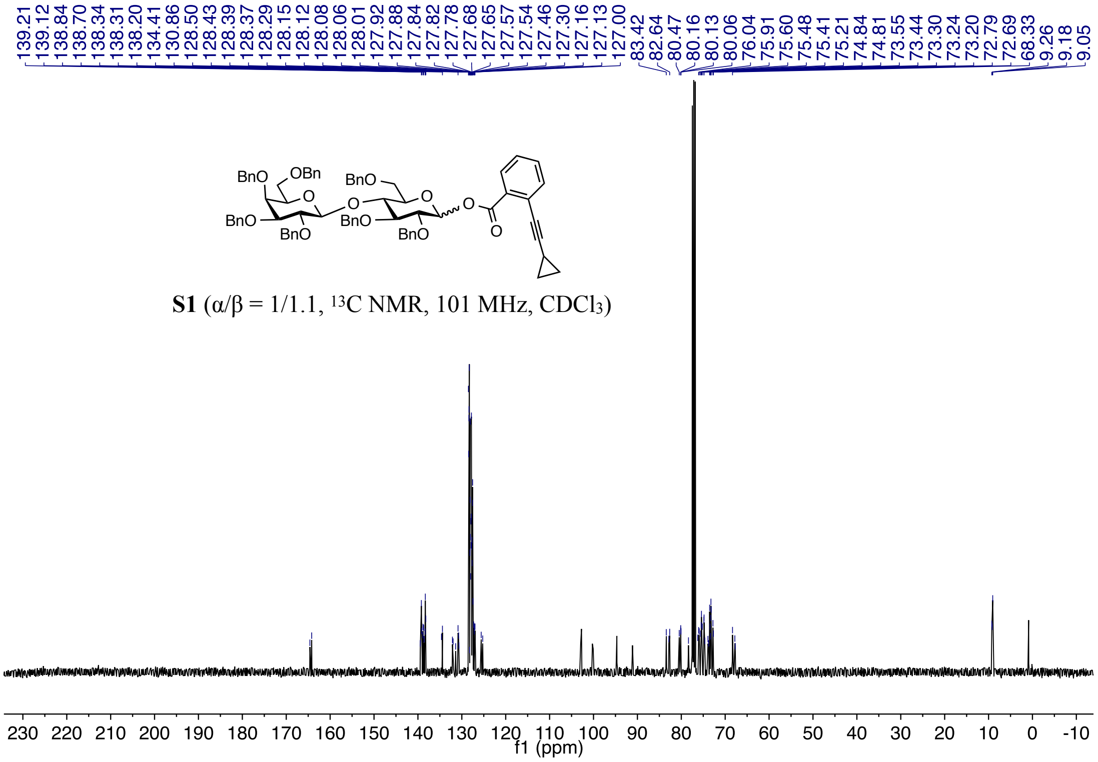

Supplementary Figure 216. ^{1}H NMR spectrum of compound **S3**

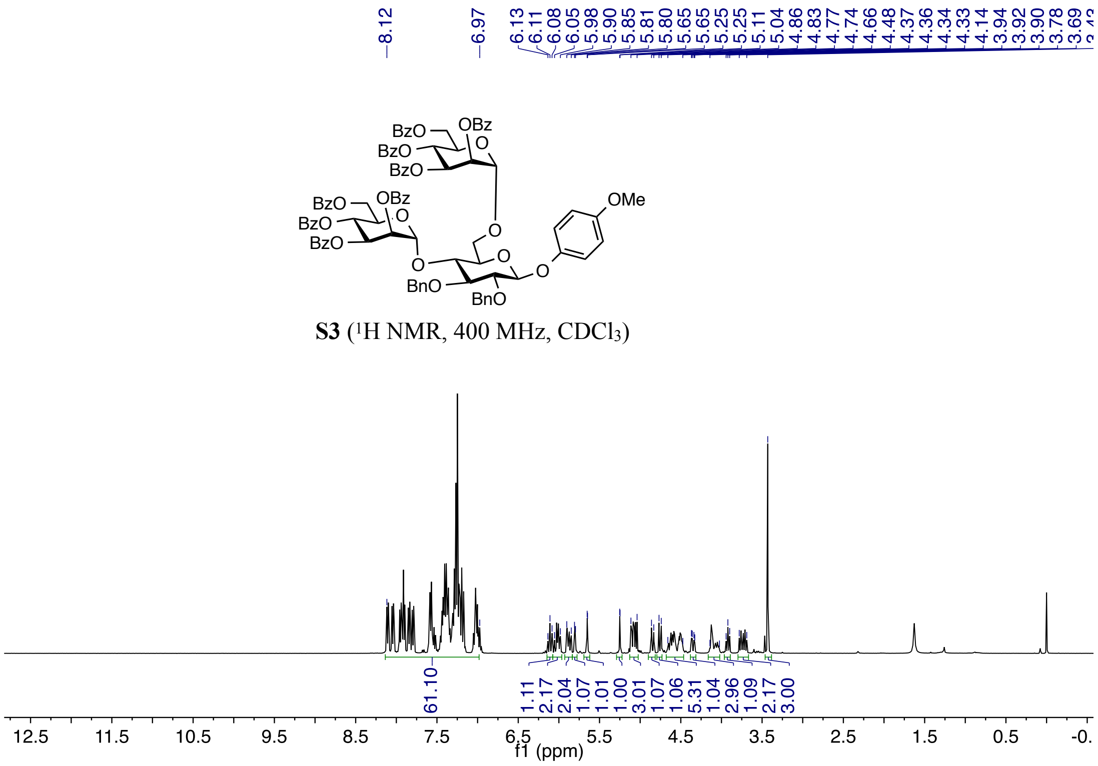

Supplementary Figure 217. ^{13}C NMR spectrum of compound **S3**

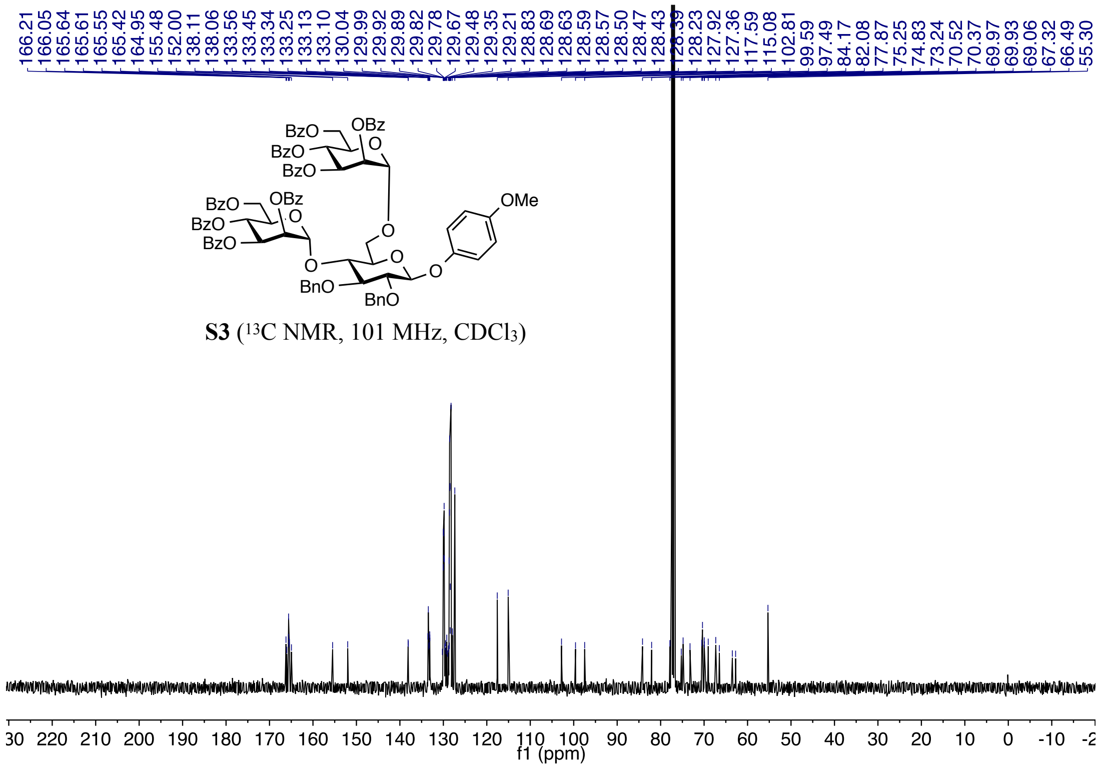

Supplementary Figure 218. ^{1}H NMR spectrum of compound **S4**

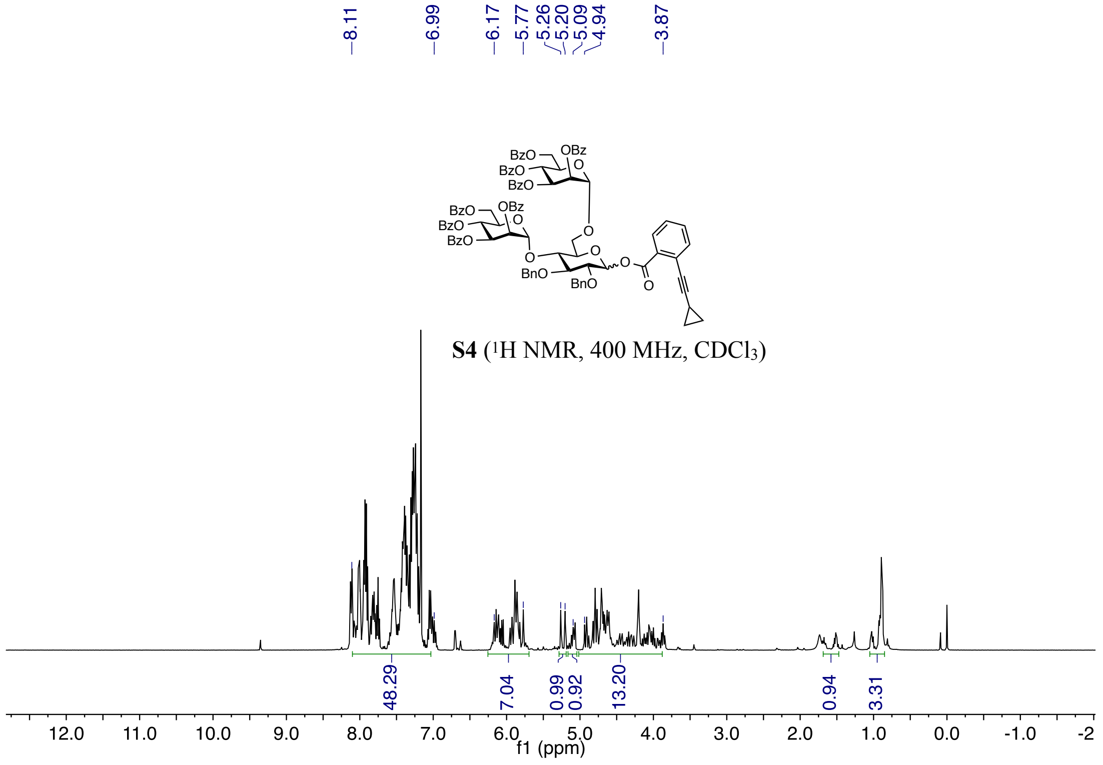

Supplementary Figure 219. ^{13}C NMR spectrum of compound **S4**

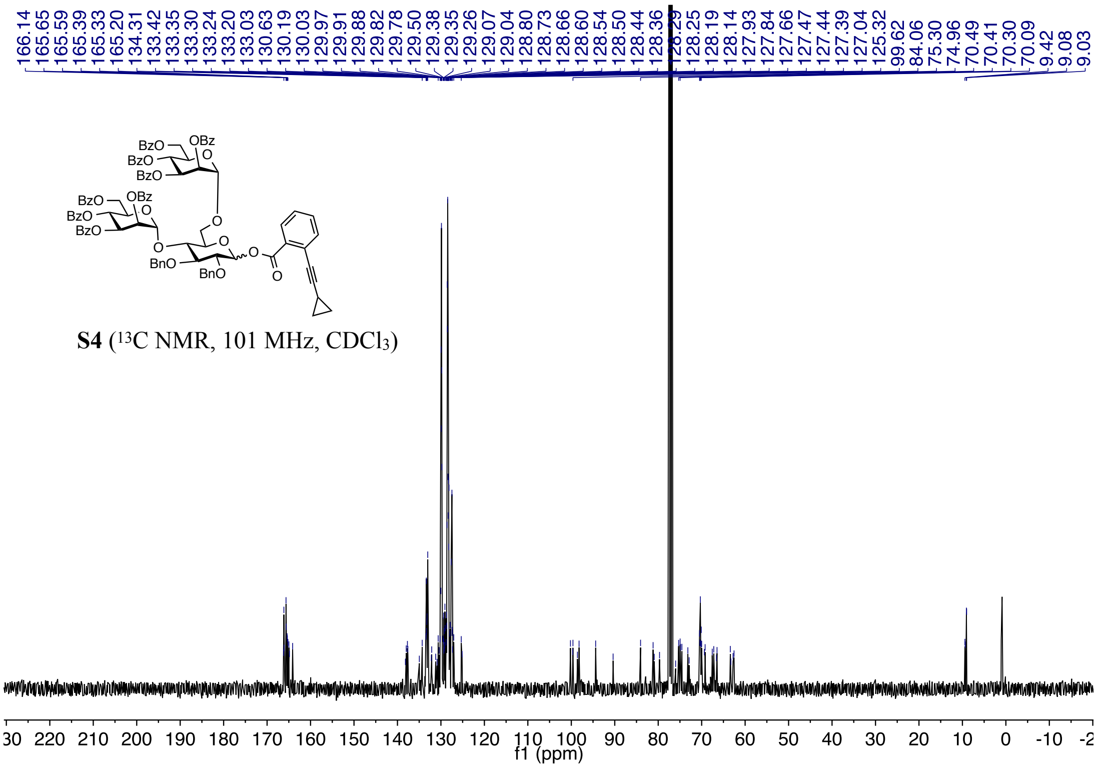

Supplementary Figure 220. ^{1}H NMR spectrum of compound **S5**

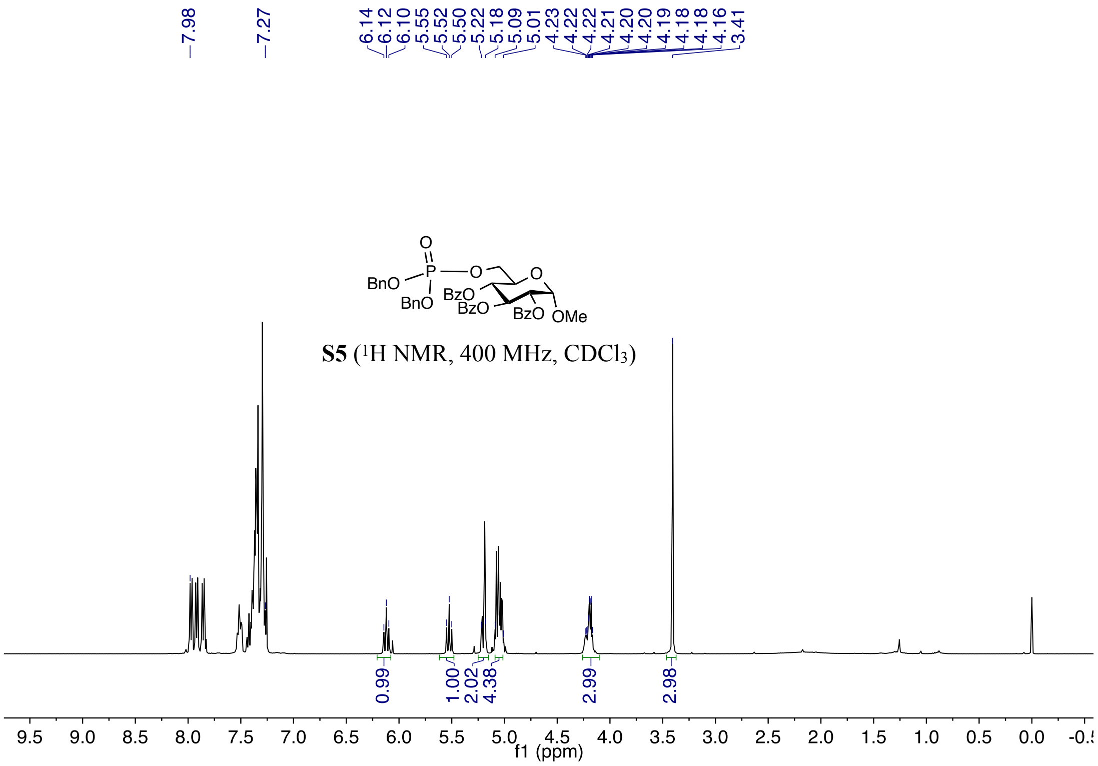

Supplementary Figure 221. ^{13}C NMR spectrum of compound **S5**

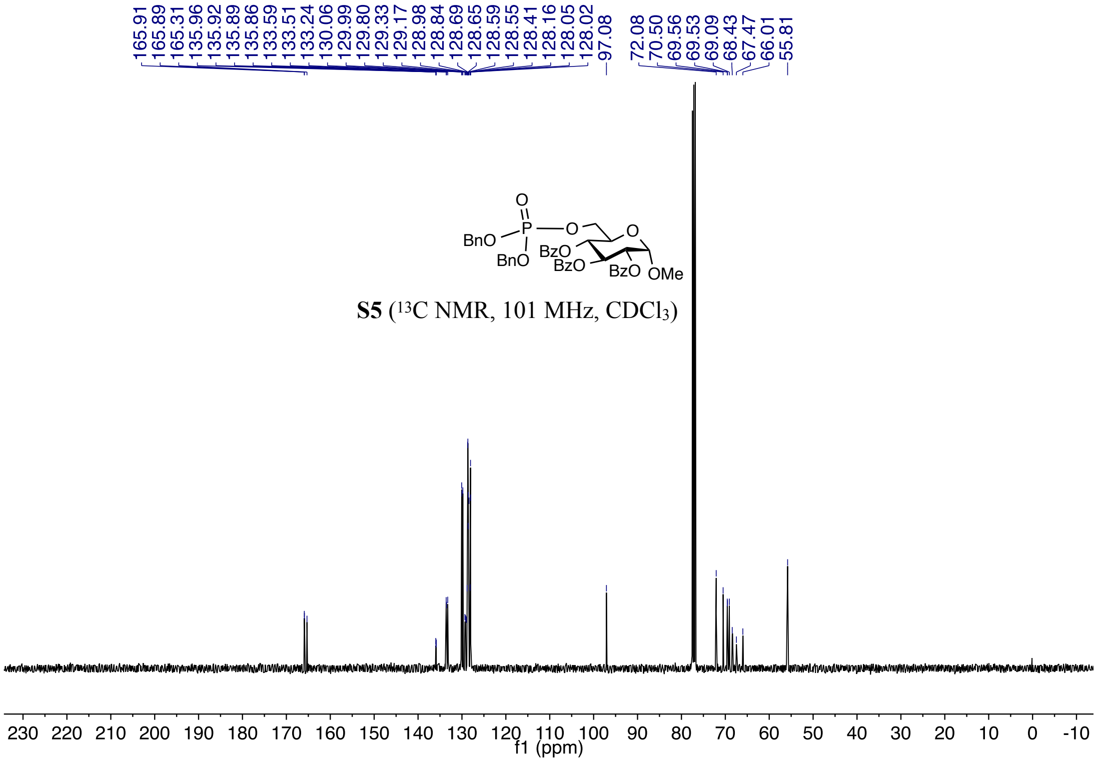

Supplementary Figure 222. ^{31}P NMR spectrum of compound **S5**

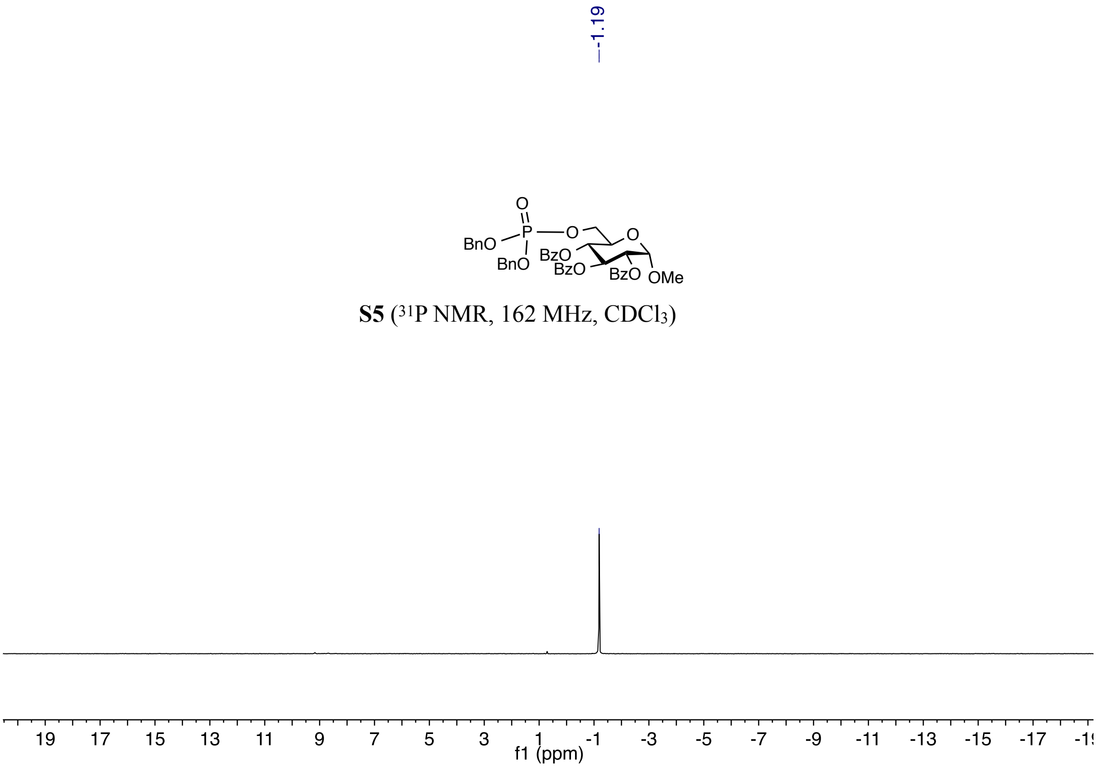

Supplementary Figure 223. ^{1}H NMR spectrum of compound **2b**

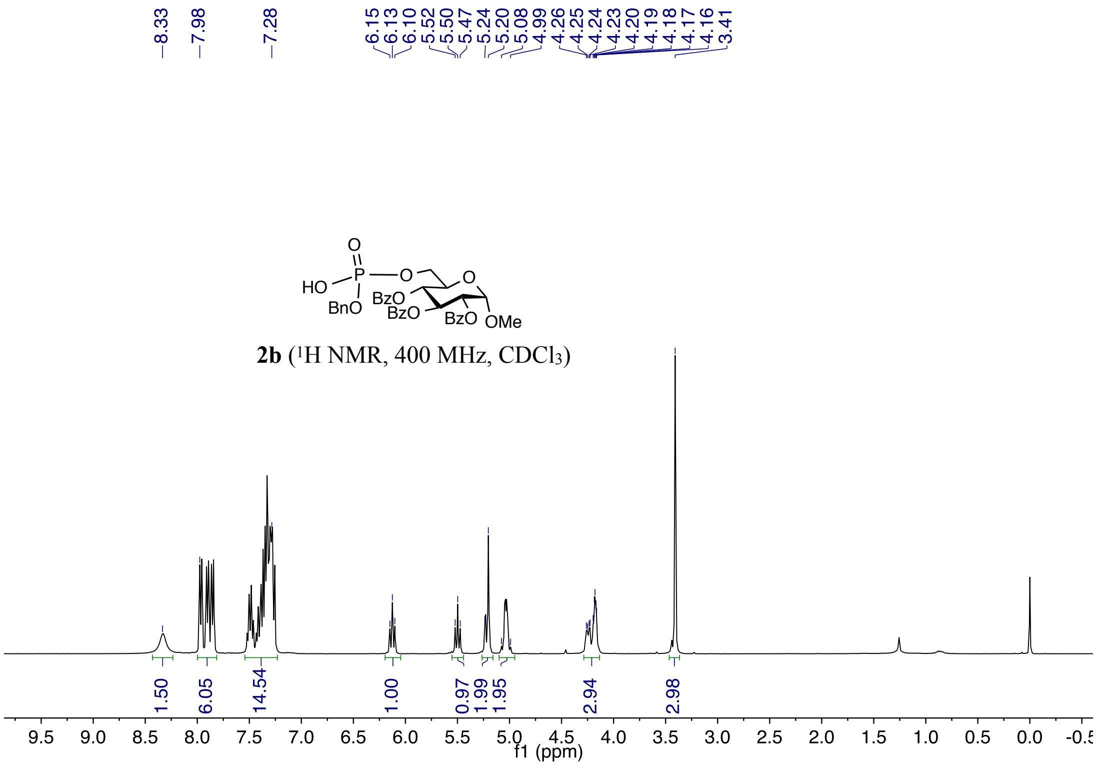

Supplementary Figure 224. ^{13}C NMR spectrum of compound **2b**

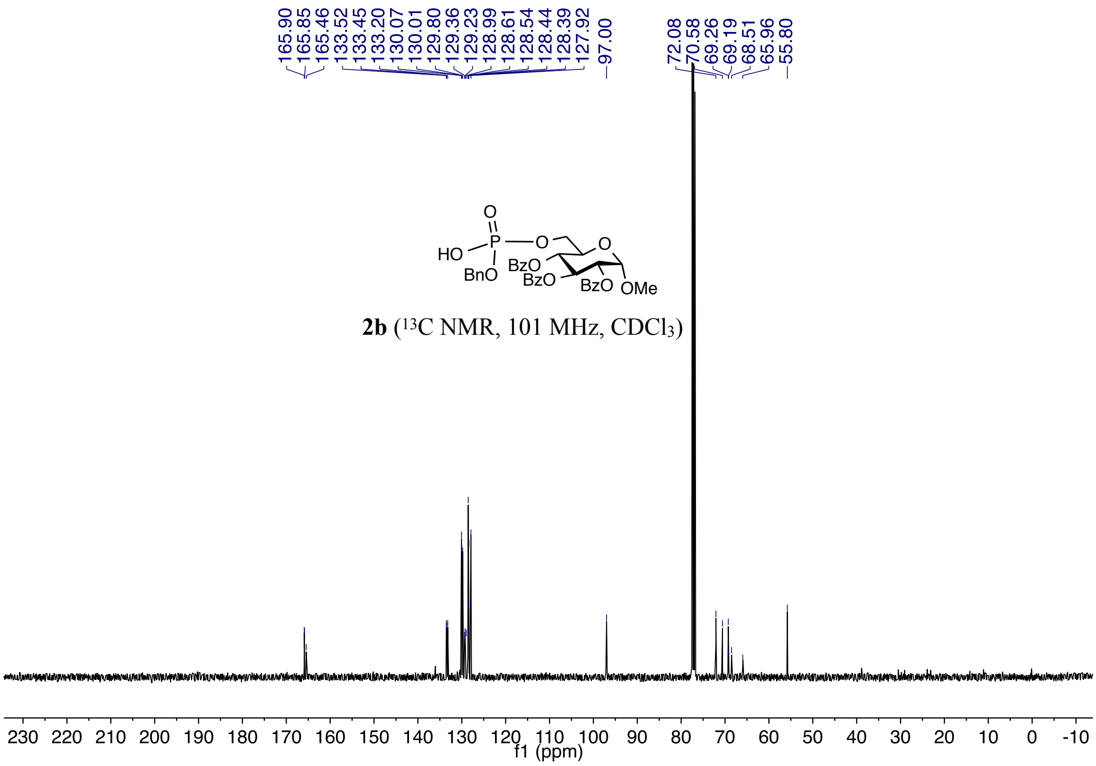

Supplementary Figure 225. ^{31}P NMR spectrum of compound **2b**

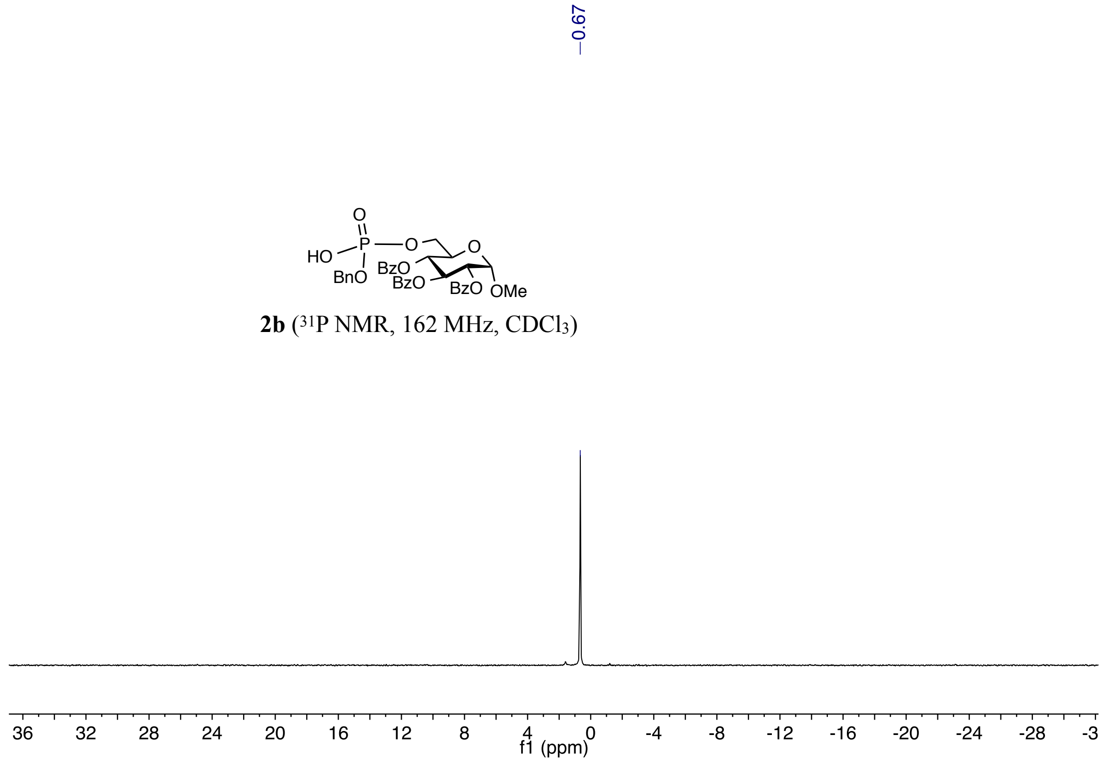

Supplementary Figure 226. ^{1}H NMR spectrum of compound **S6**

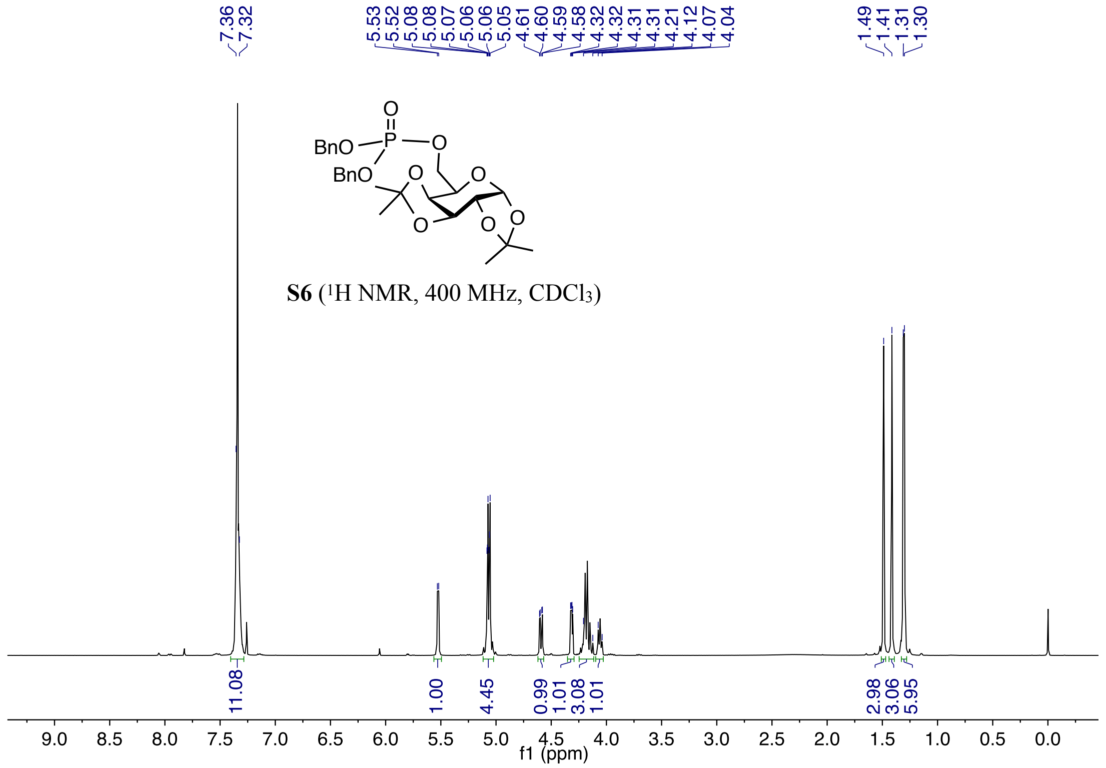

Supplementary Figure 227. ^{13}C NMR spectrum of compound **S6**

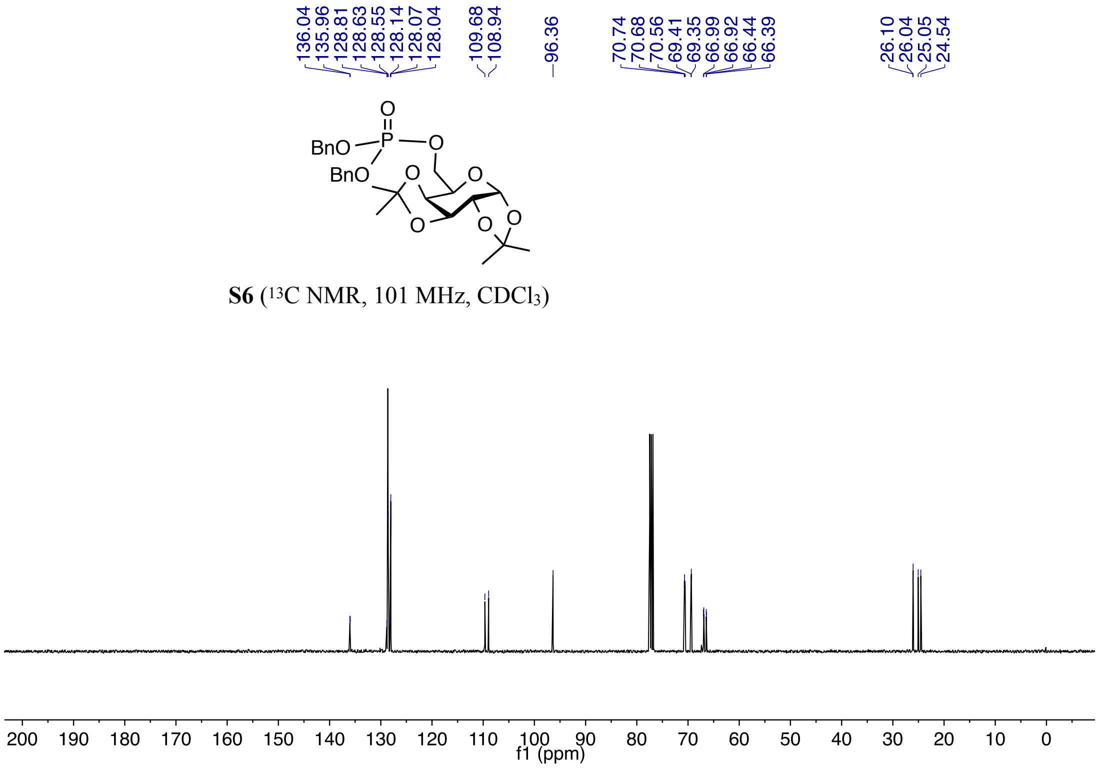

Supplementary Figure 228. ^{31}P NMR spectrum of compound **S6**

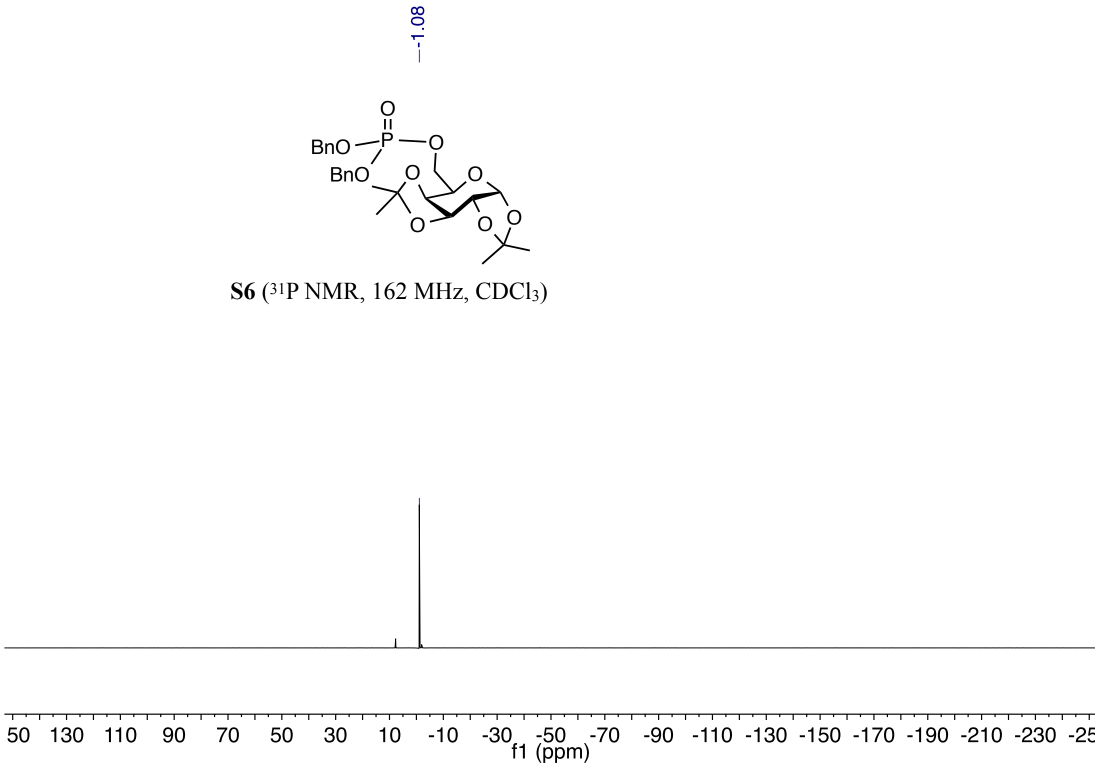

Supplementary Figure 229. ^{1}H NMR spectrum of compound **2c**

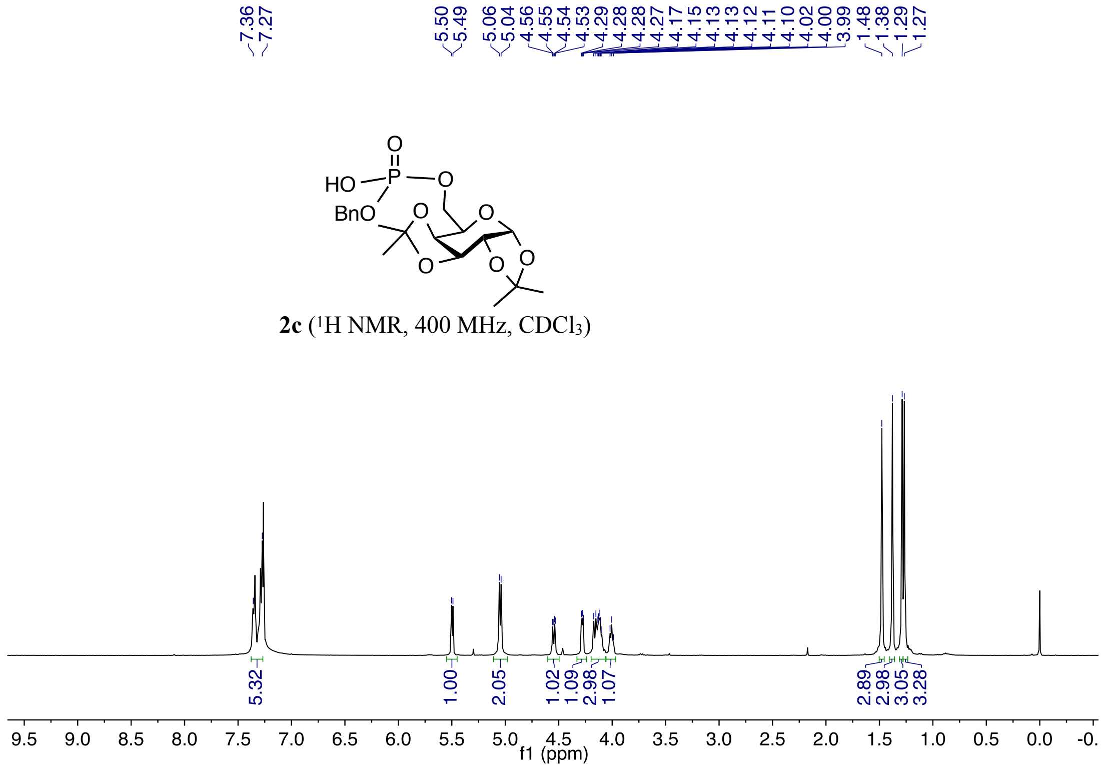

Supplementary Figure 230. ^{13}C NMR spectrum of compound **2c**

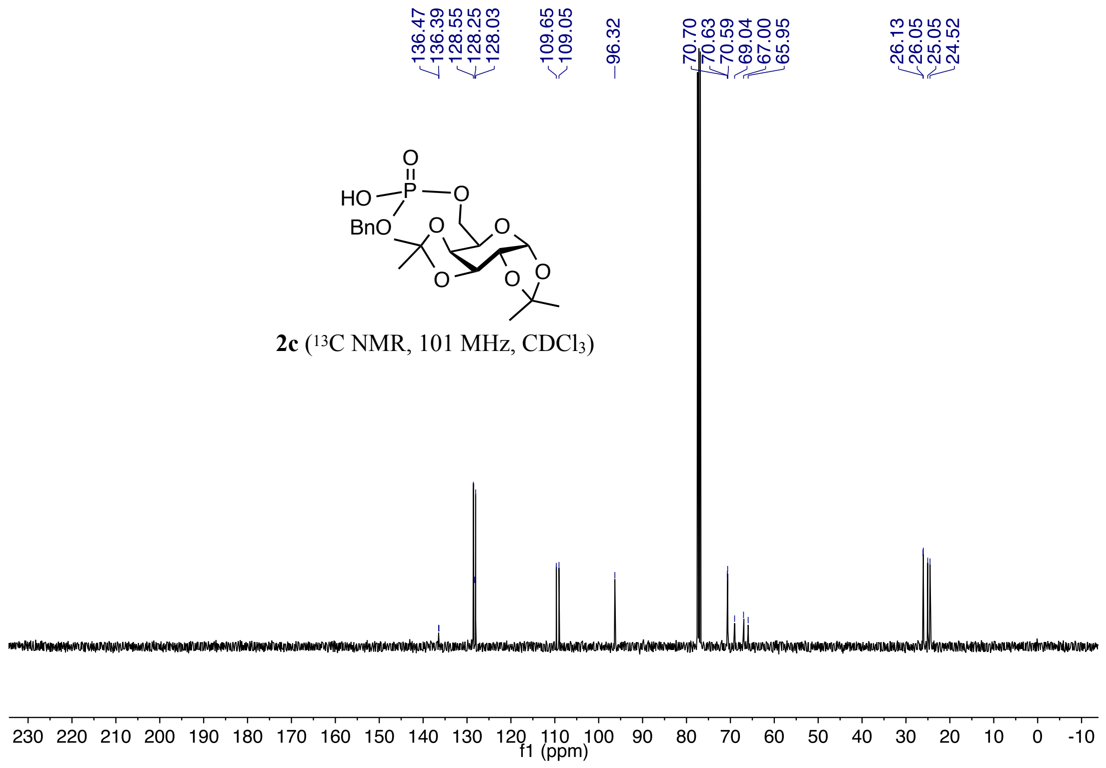

Supplementary Figure 231. ^{31}P NMR spectrum of compound **2c**

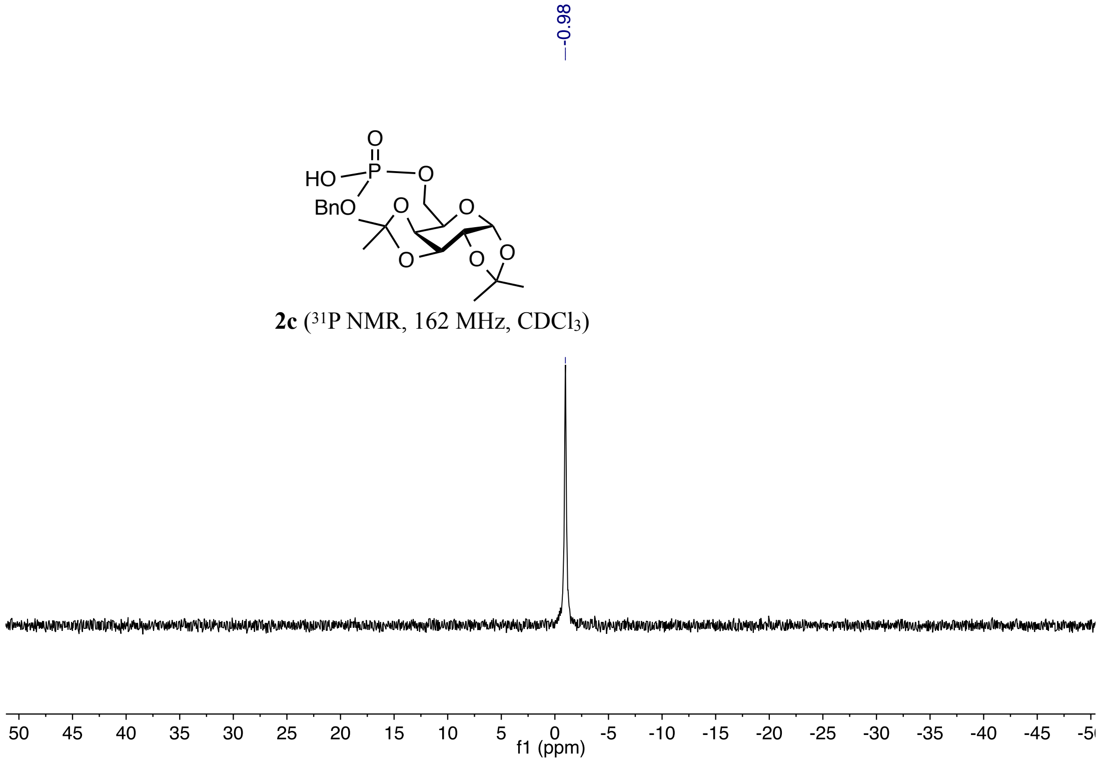

Supplementary Figure 232. ^{1}H NMR spectrum of compound **S7**

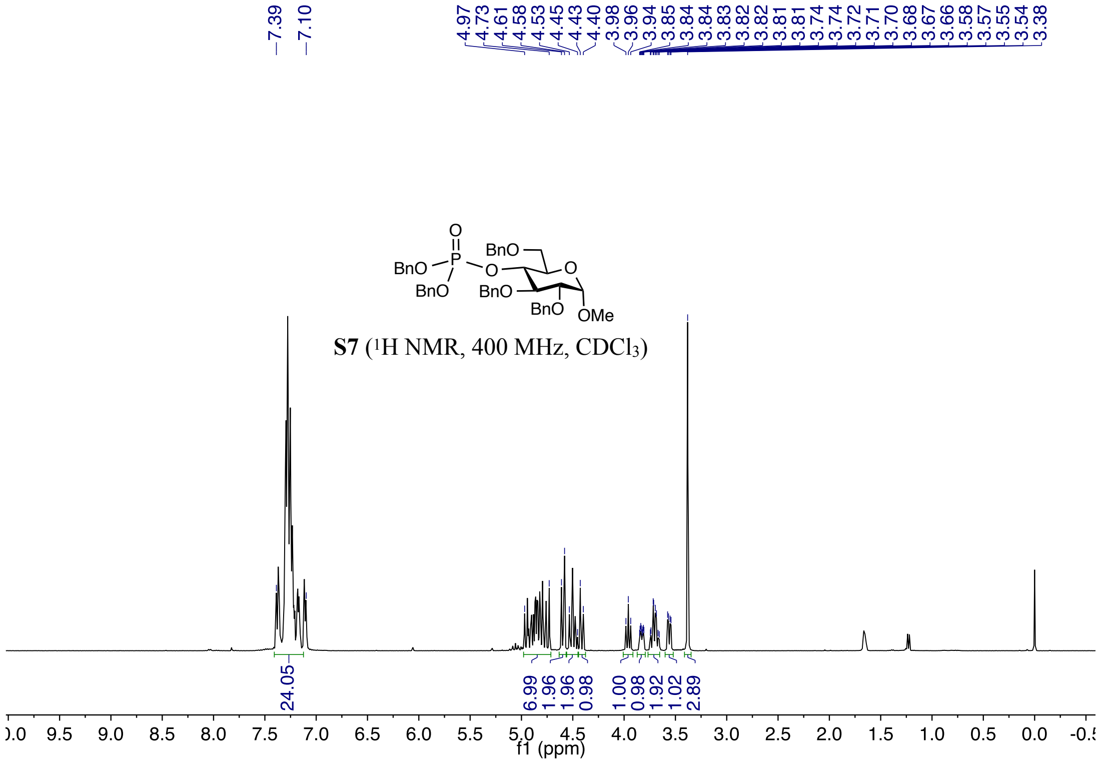

Supplementary Figure 233. ^{13}C NMR spectrum of compound **S7**

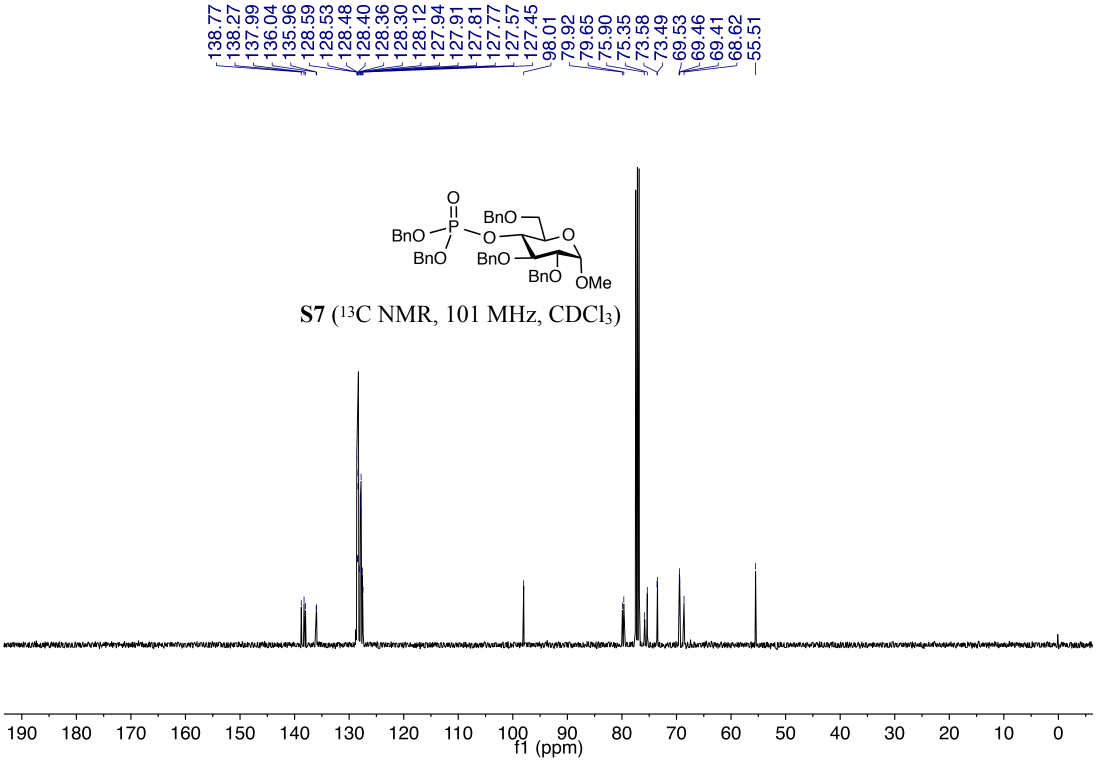

Supplementary Figure 234. ^{31}P NMR spectrum of compound **S7**

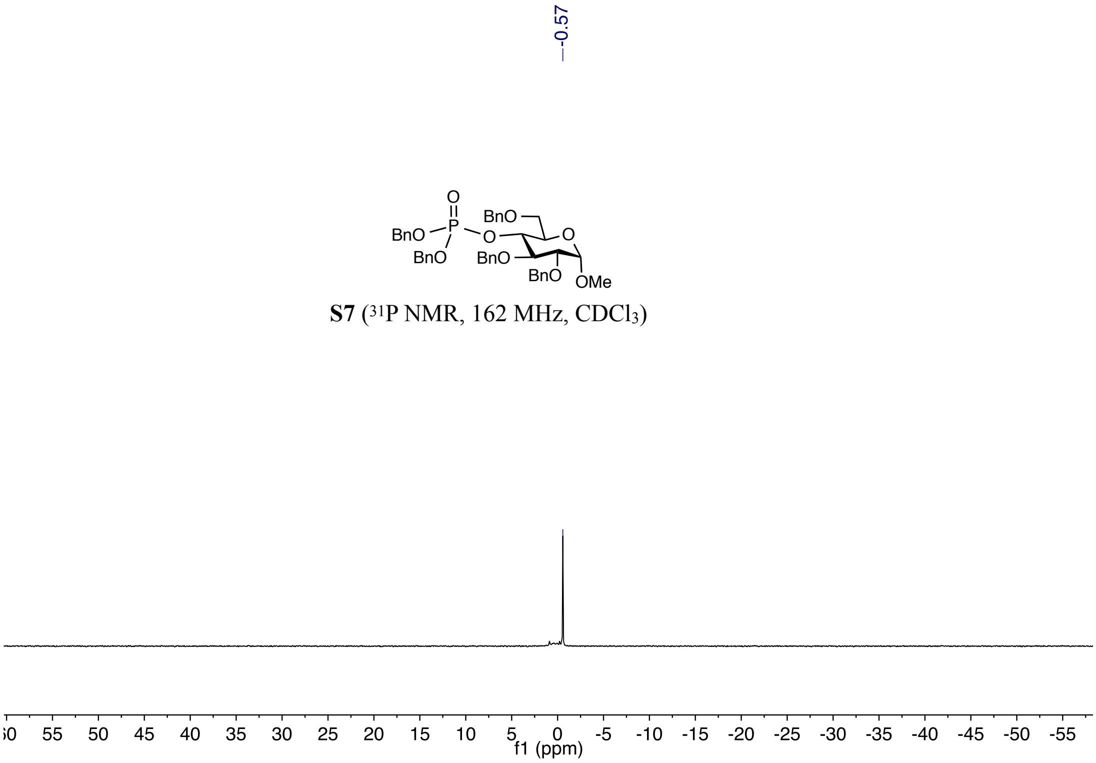

Supplementary Figure 235. ^{1}H NMR spectrum of compound **2d**

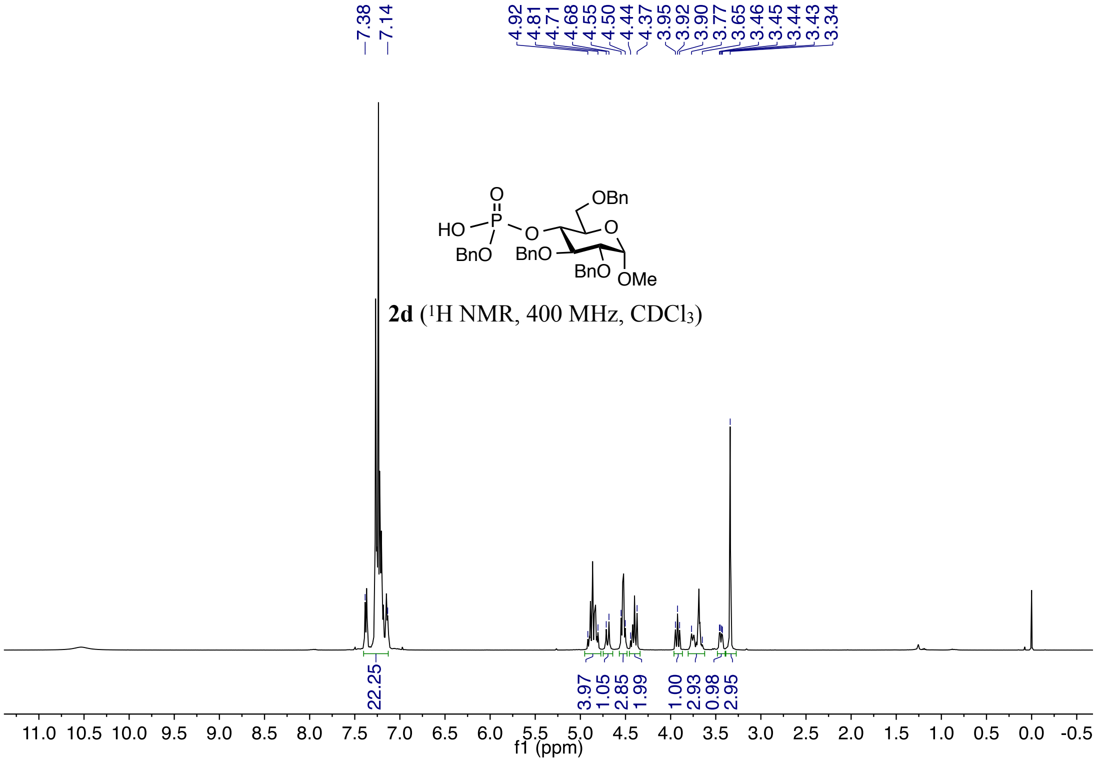

Supplementary Figure 236. ^{13}C NMR spectrum of compound **2d**

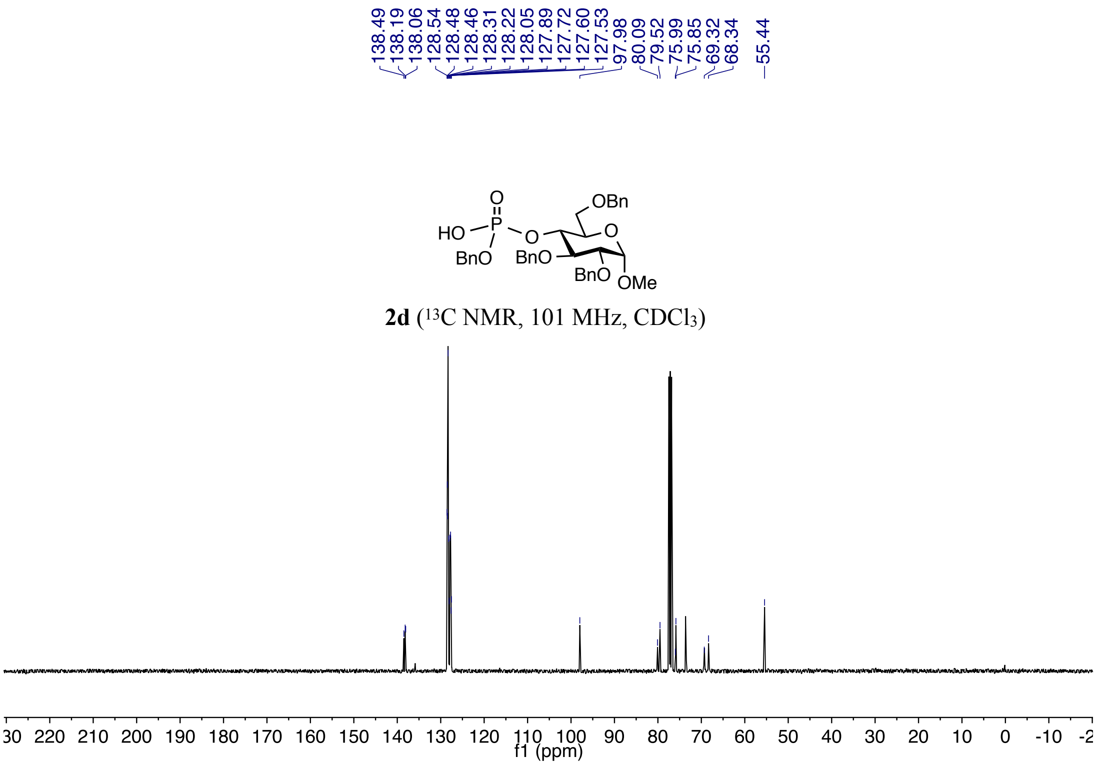

Supplementary Figure 237. ^{31}P NMR spectrum of compound **2d**

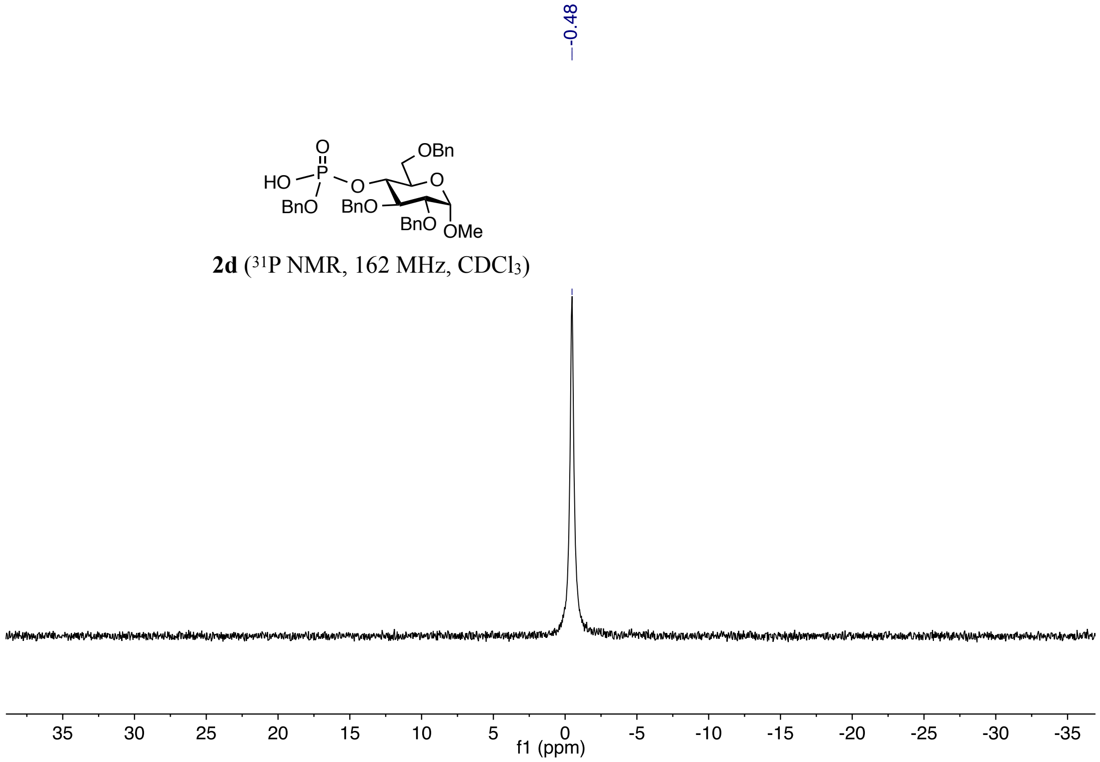

Supplementary Figure 238. ^{1}H NMR spectrum of compound **S8**

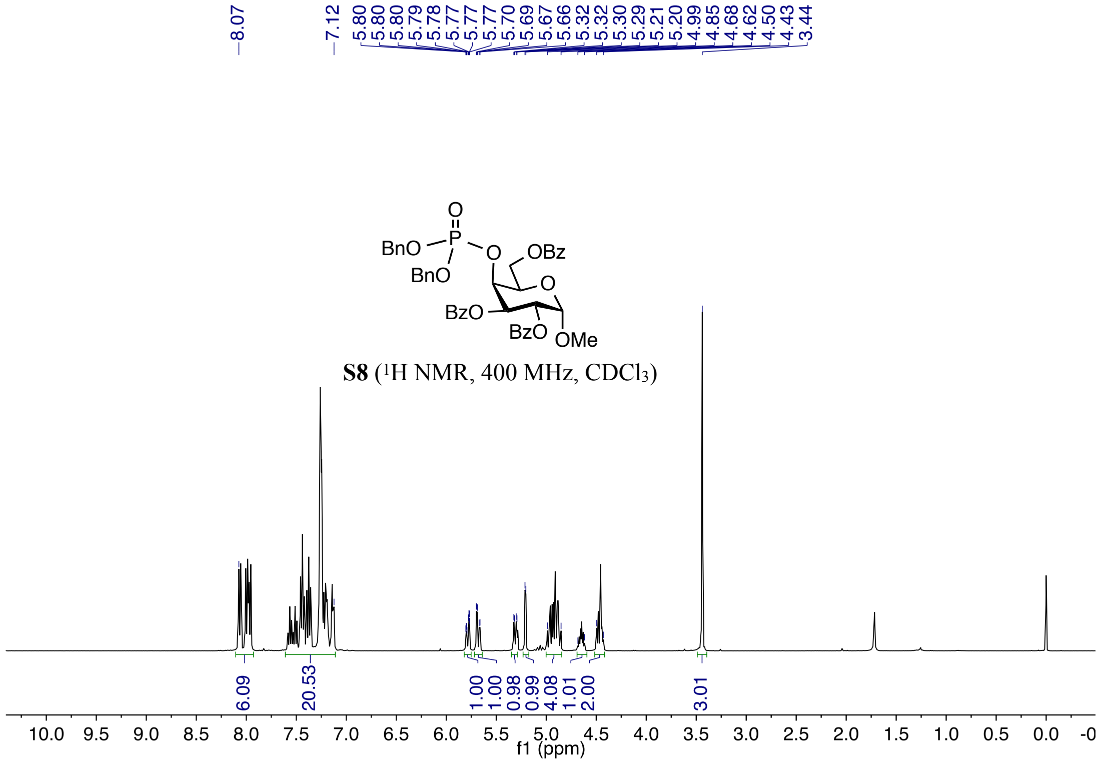

Supplementary Figure 239. ^{13}C NMR spectrum of compound **S8**

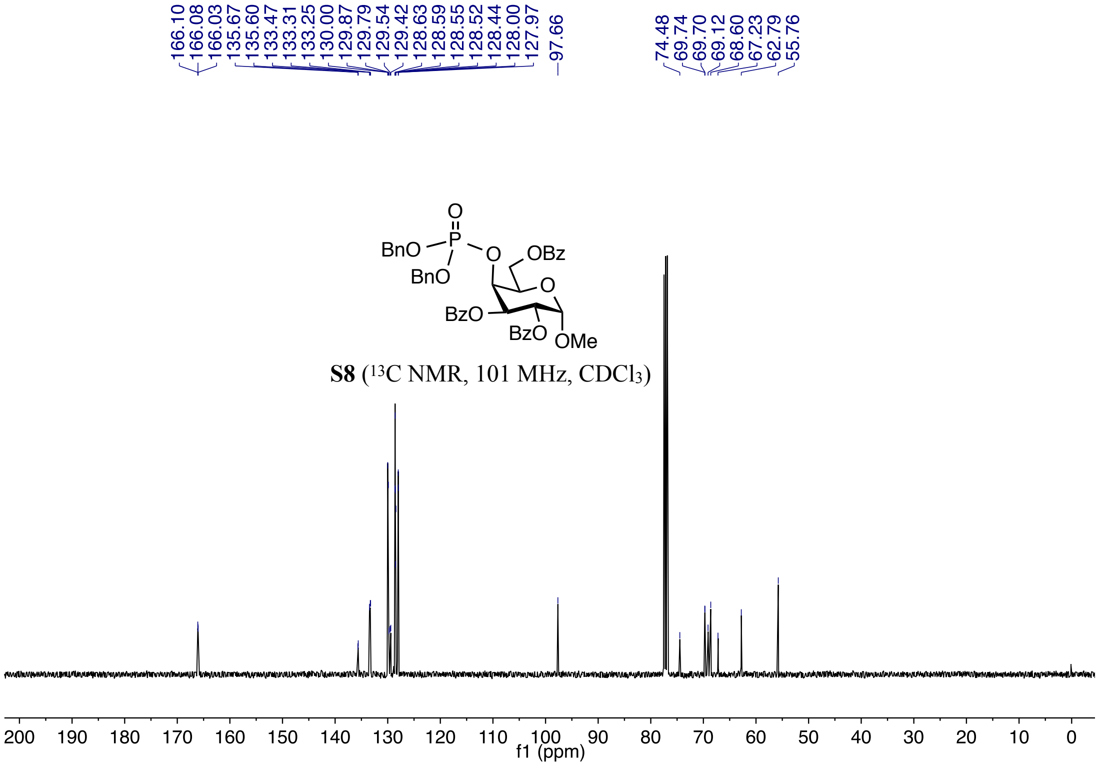

Supplementary Figure 240. ^{31}P NMR spectrum of compound **S8**

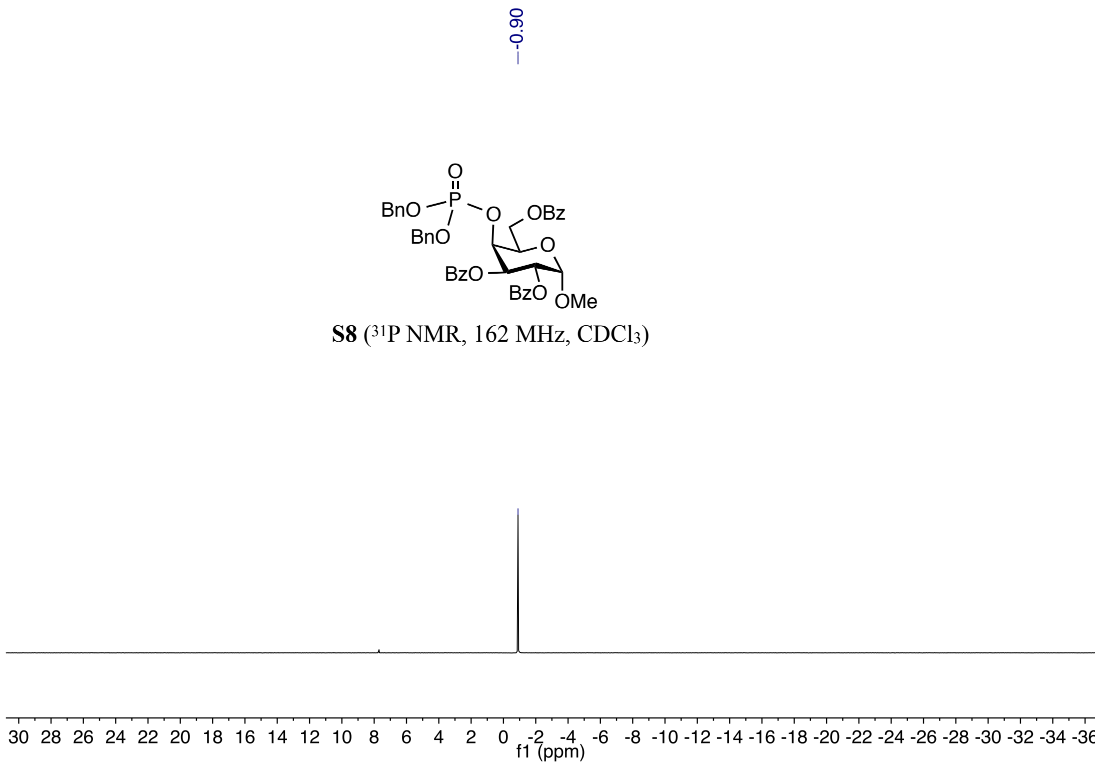

Supplementary Figure 241. ^{1}H NMR spectrum of compound **2e**

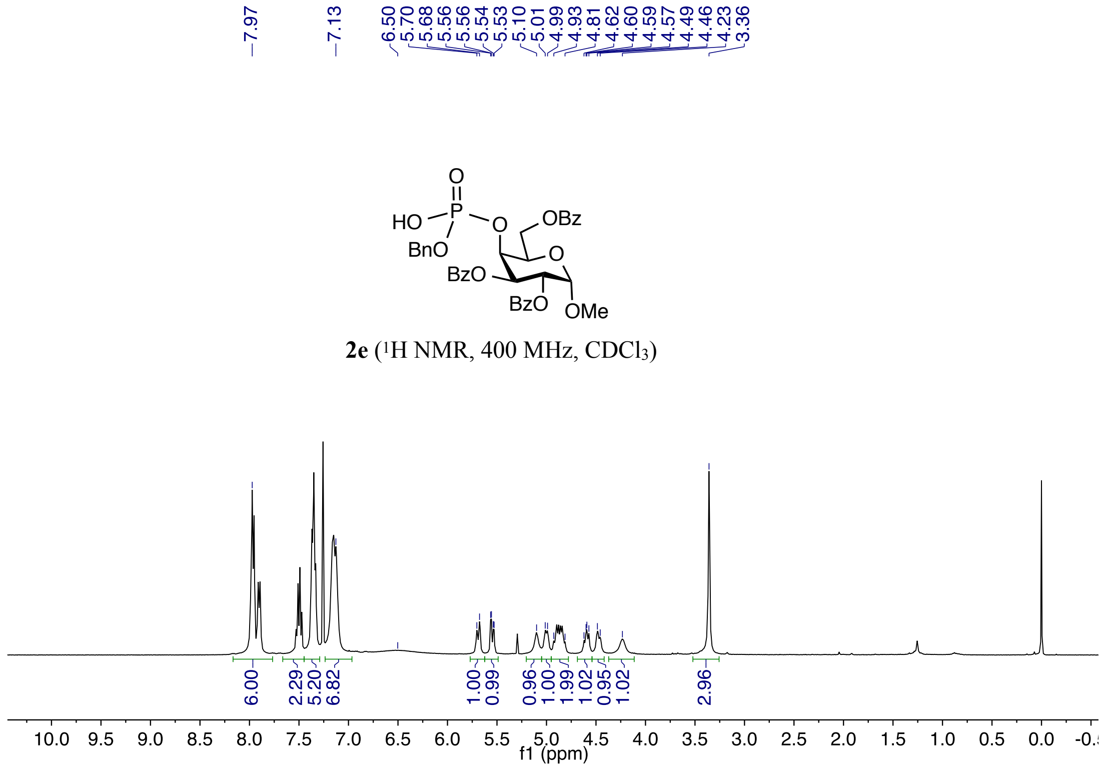

Supplementary Figure 242. ^{13}C NMR spectrum of compound **2e**

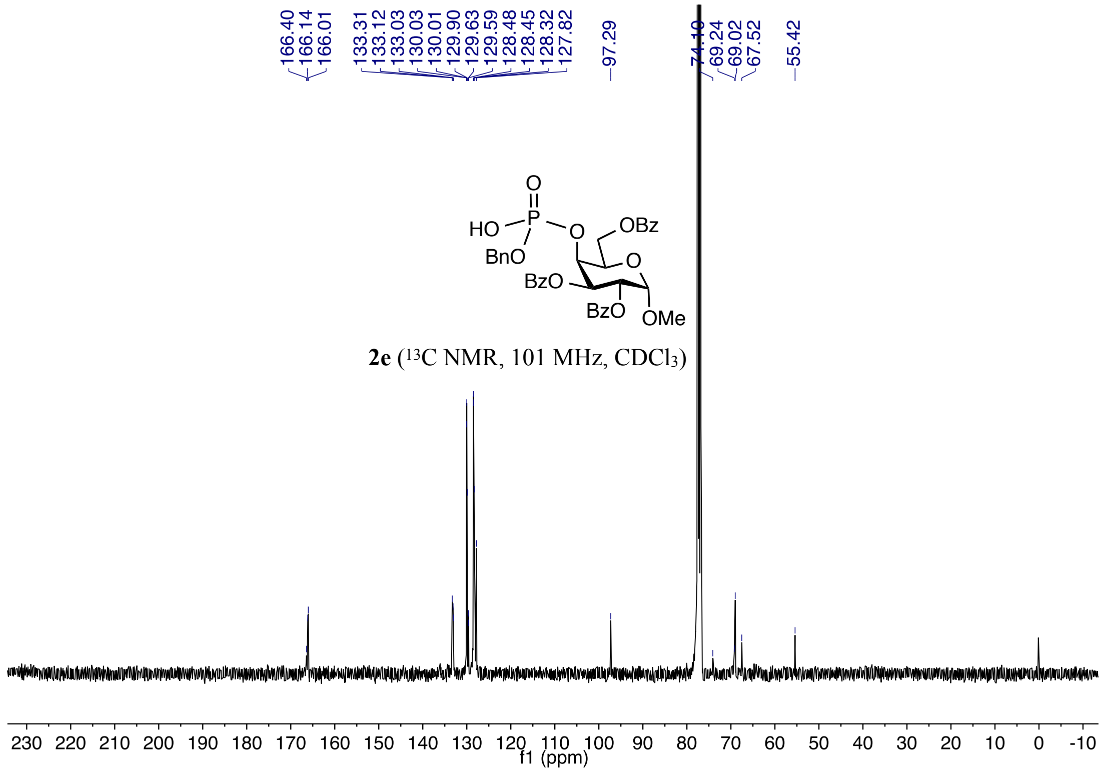

Supplementary Figure 243. ^{31}P NMR spectrum of compound **2e**

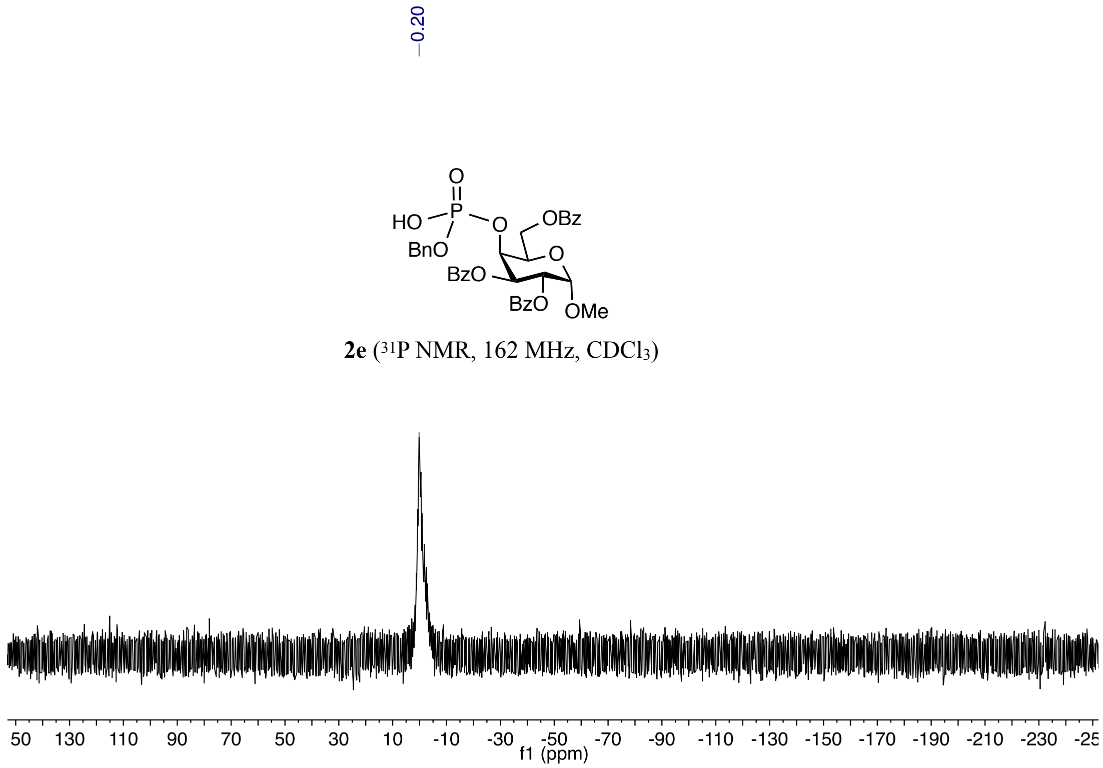

Supplementary Figure 244. ^{1}H NMR spectrum of compound **S9**

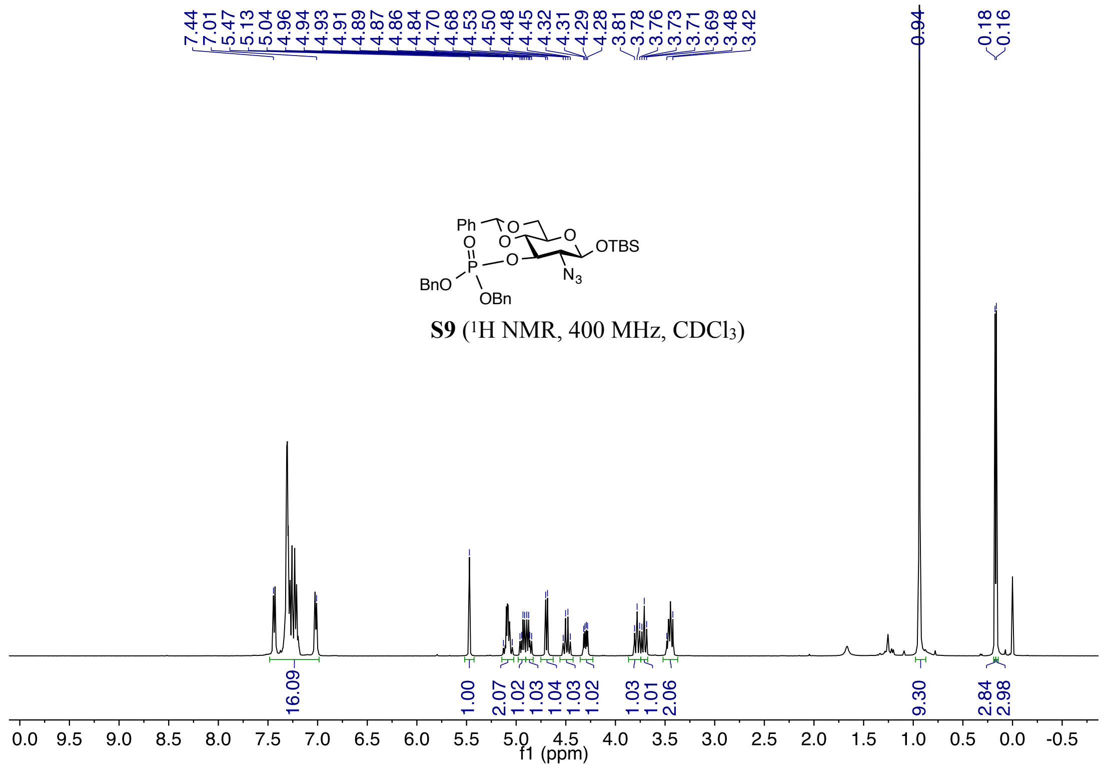

Supplementary Figure 245. ^{13}C NMR spectrum of compound **S9**

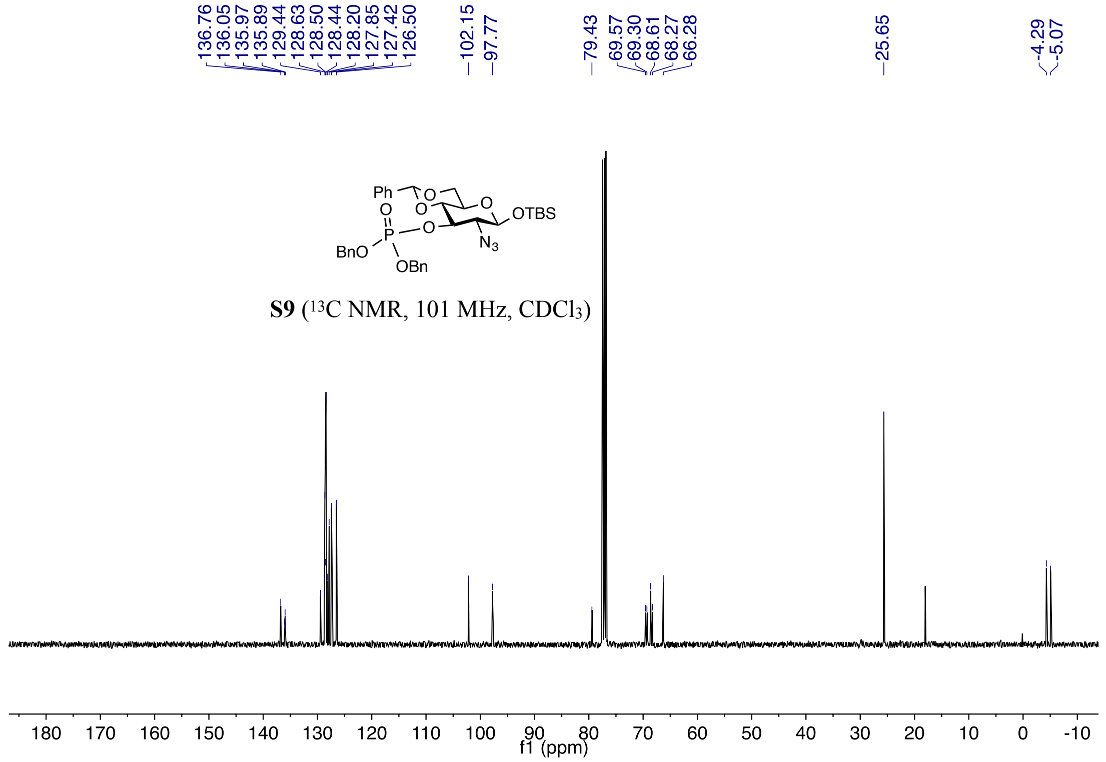

Supplementary Figure 246. ^{31}P NMR spectrum of compound **S9**

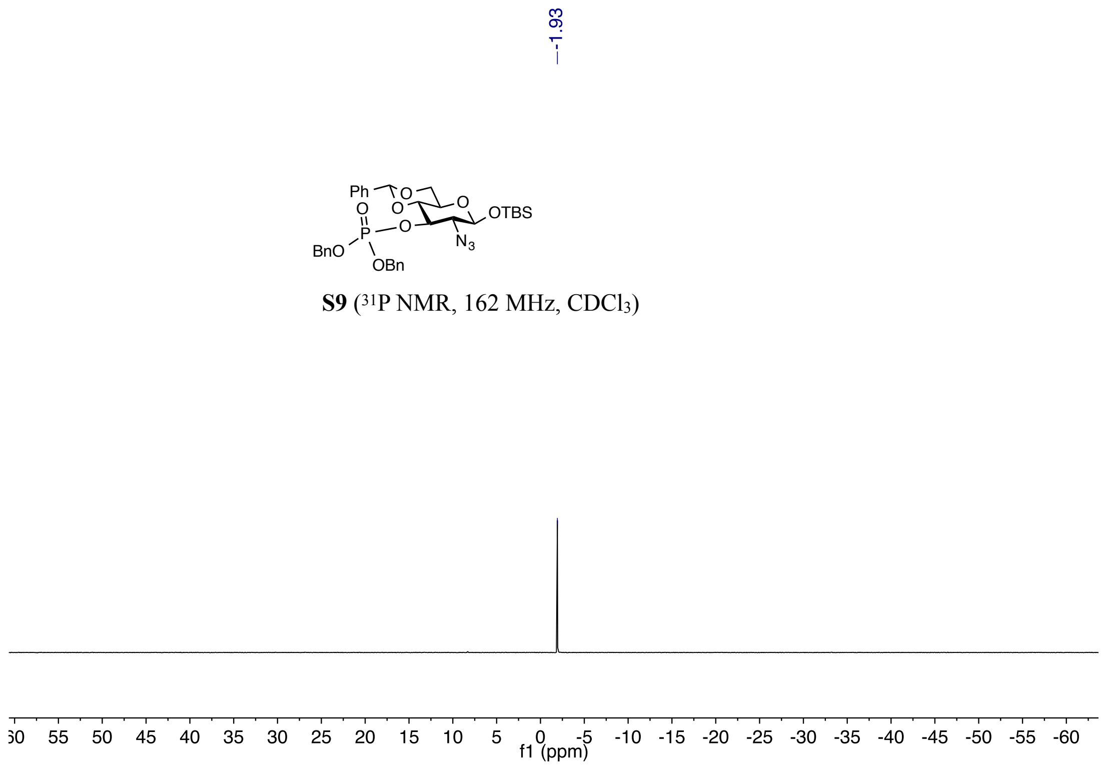

Supplementary Figure 247. ^{1}H NMR spectrum of compound **2f**

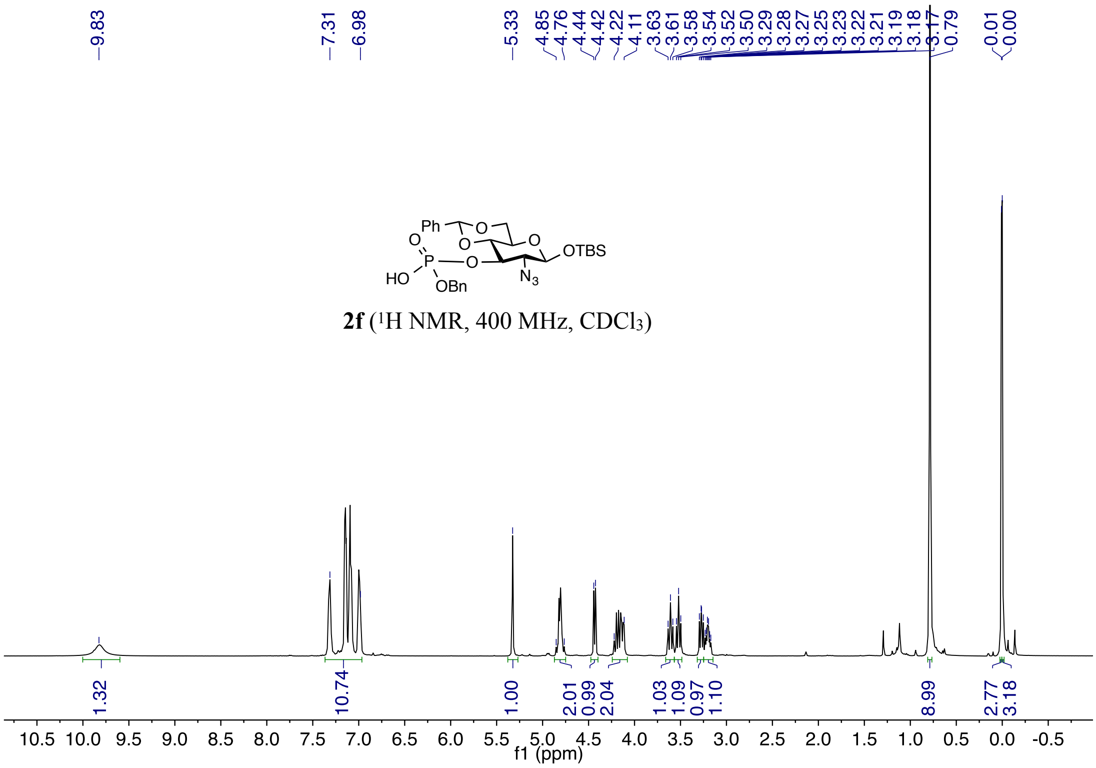

Supplementary Figure 248. ^{13}C NMR spectrum of compound **2f**

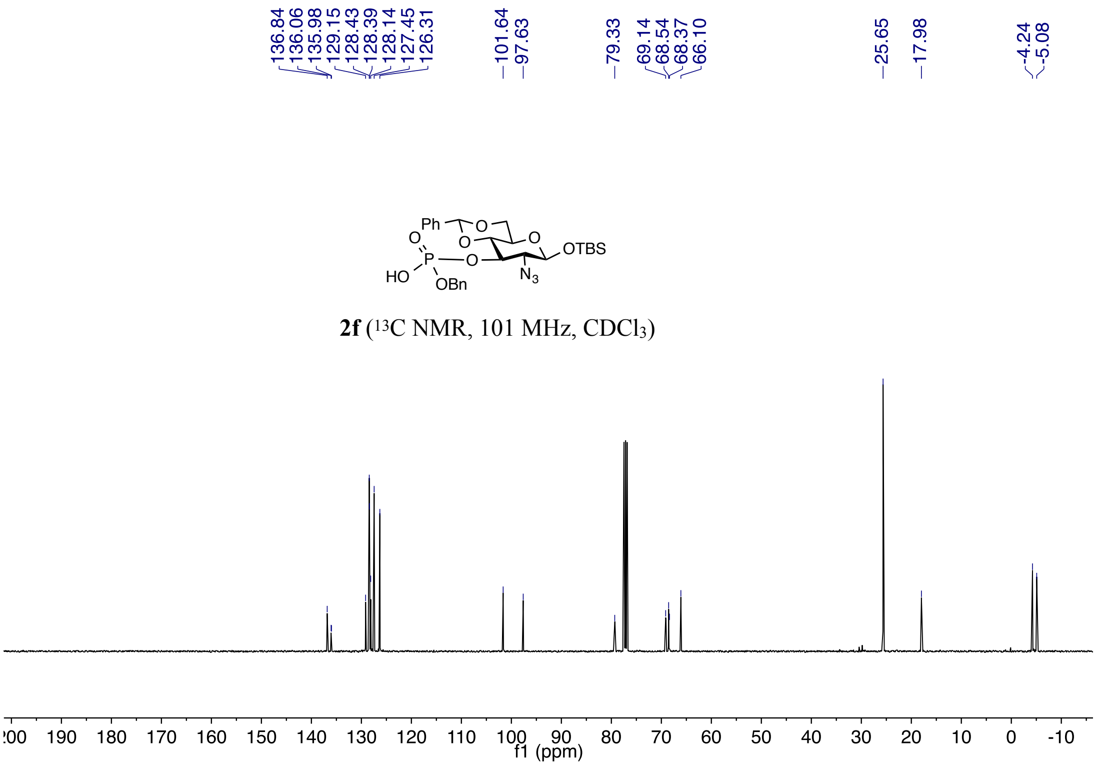

Supplementary Figure 249. ^{31}P NMR spectrum of compound **2f**

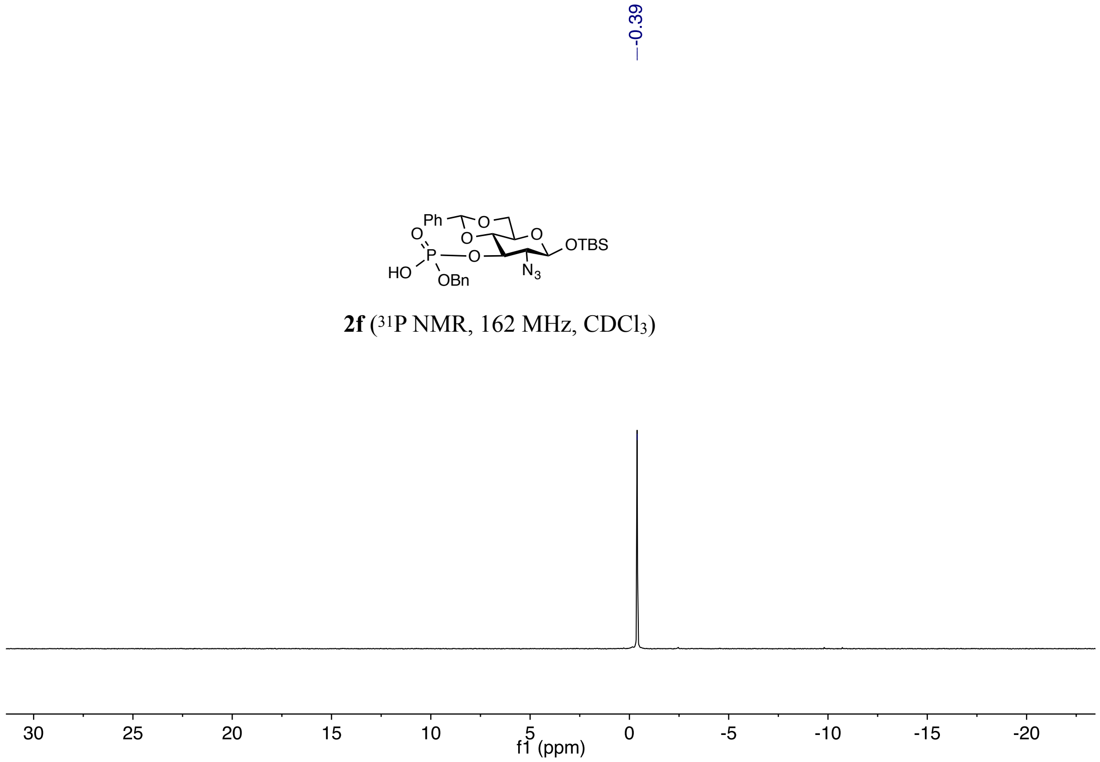

Supplementary Figure 250. ^{1}H NMR spectrum of compound **S10**

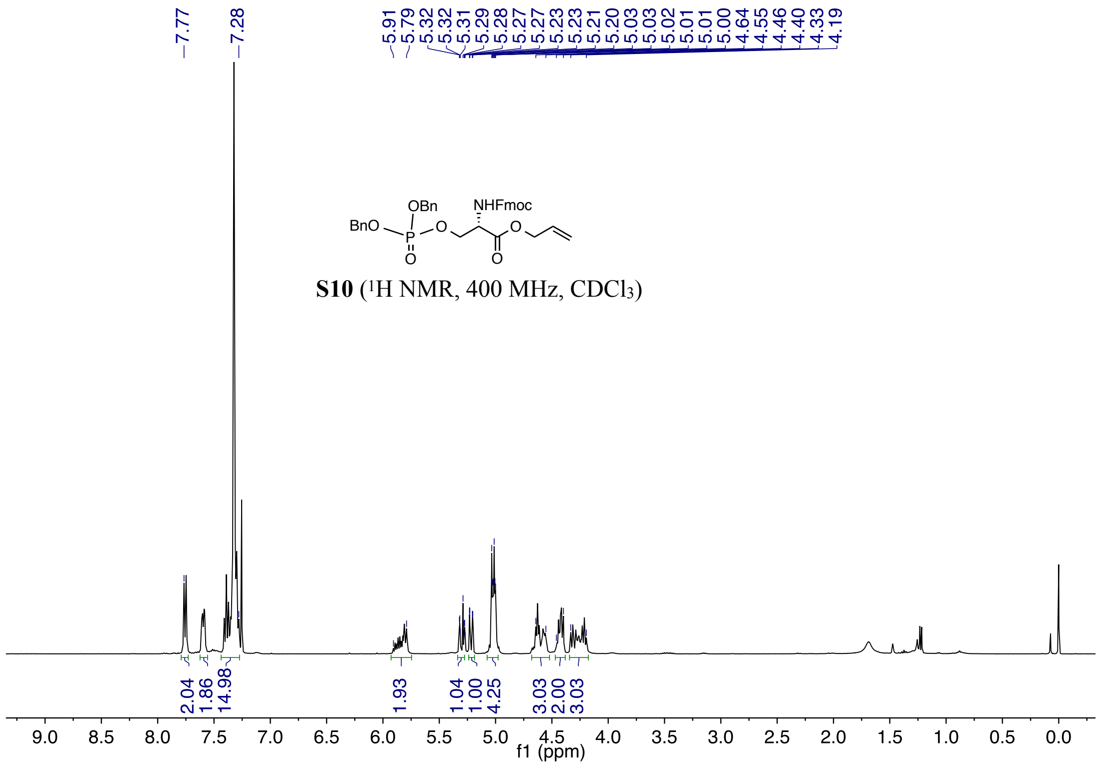

Supplementary Figure 251. ^{13}C NMR spectrum of compound **S10**

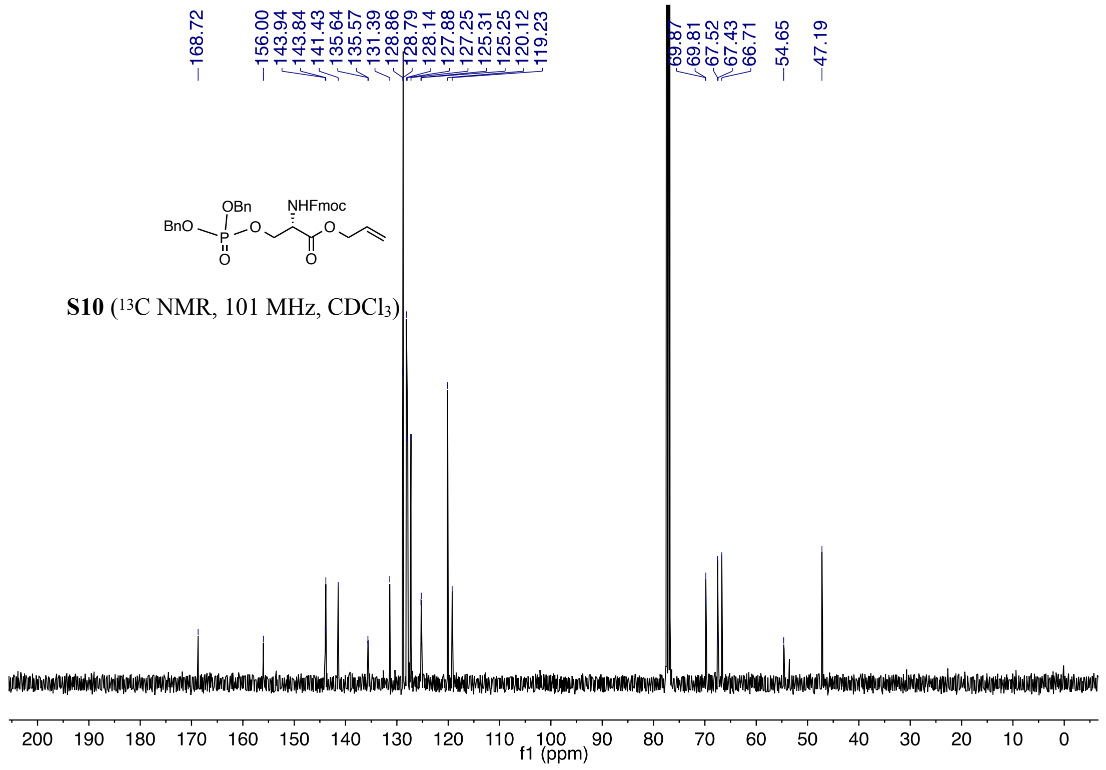

Supplementary Figure 252. ^{31}P NMR spectrum of compound **S10**

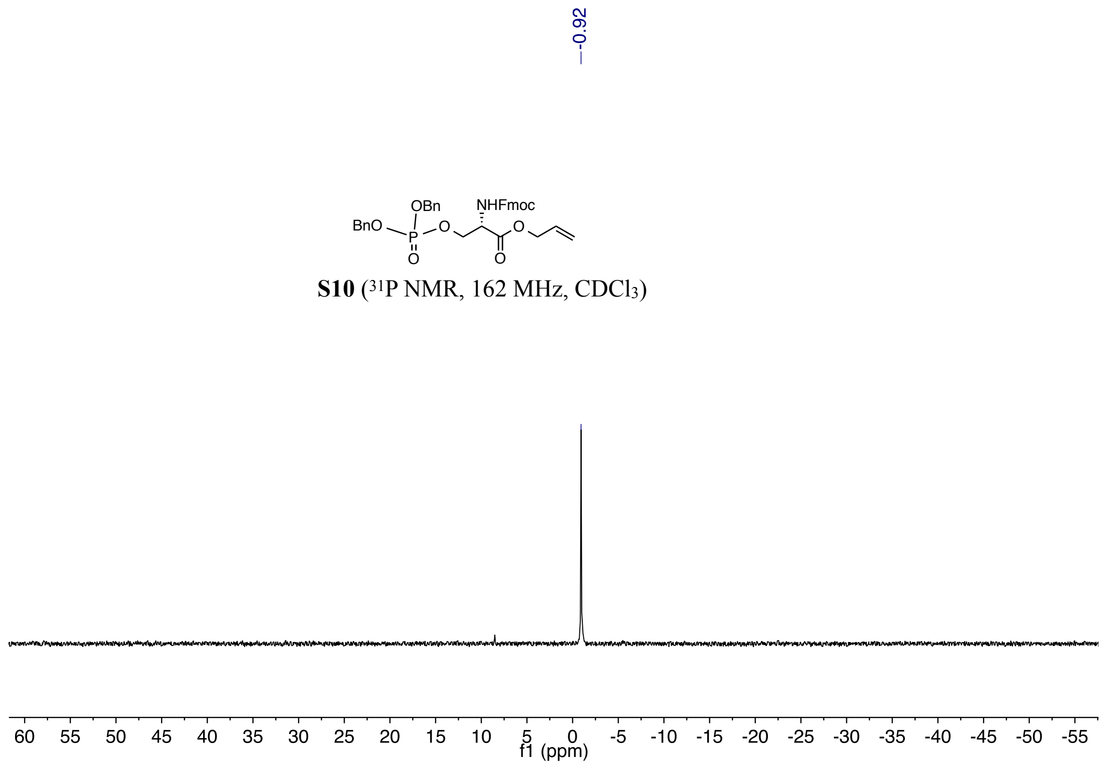

Supplementary Figure 253. ^{1}H NMR spectrum of compound **2g**

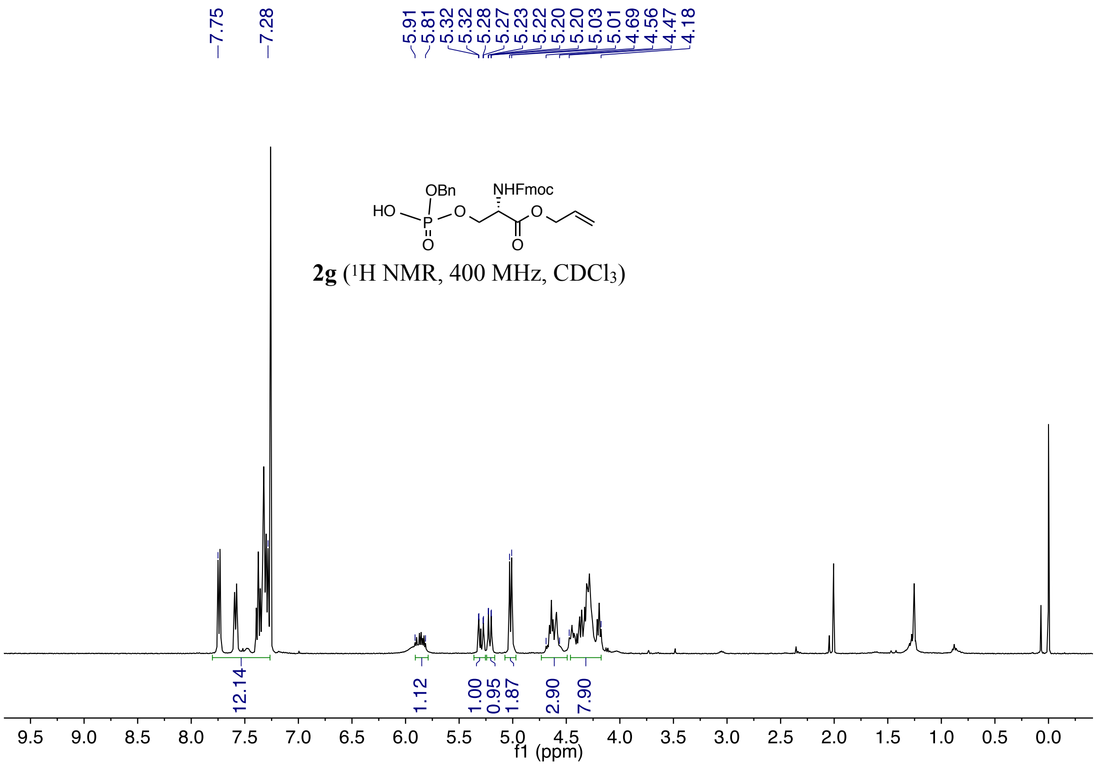

Supplementary Figure 254. ^{13}C NMR spectrum of compound **2g**

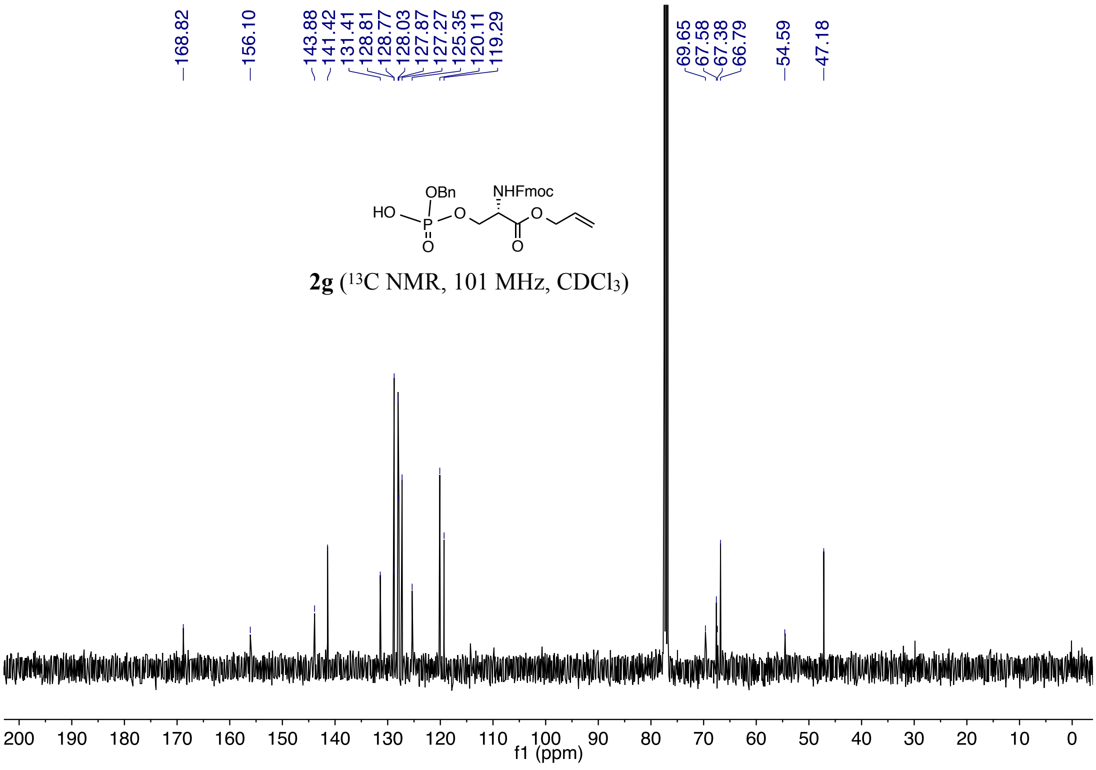

Supplementary Figure 255. ^{31}P NMR spectrum of compound **2g**

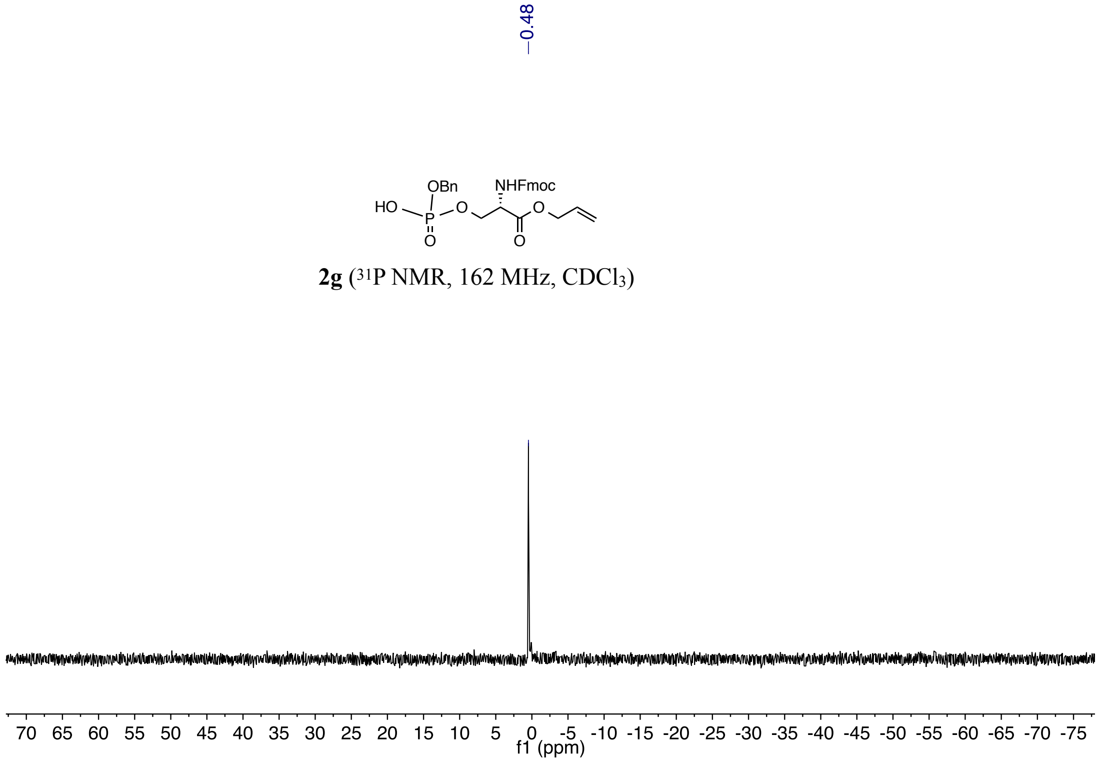

Supplementary Figure 256. ^{1}H NMR spectrum of compound **5**

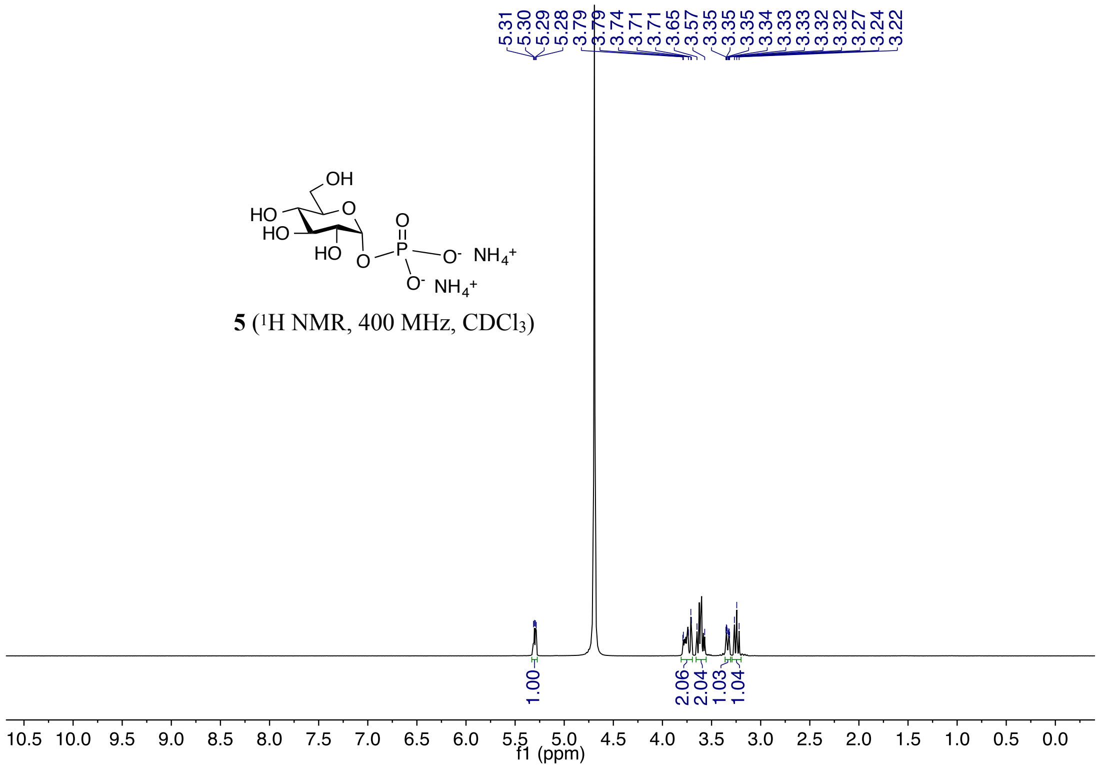

Supplementary Figure 257. ^{13}C NMR spectrum of compound **5**

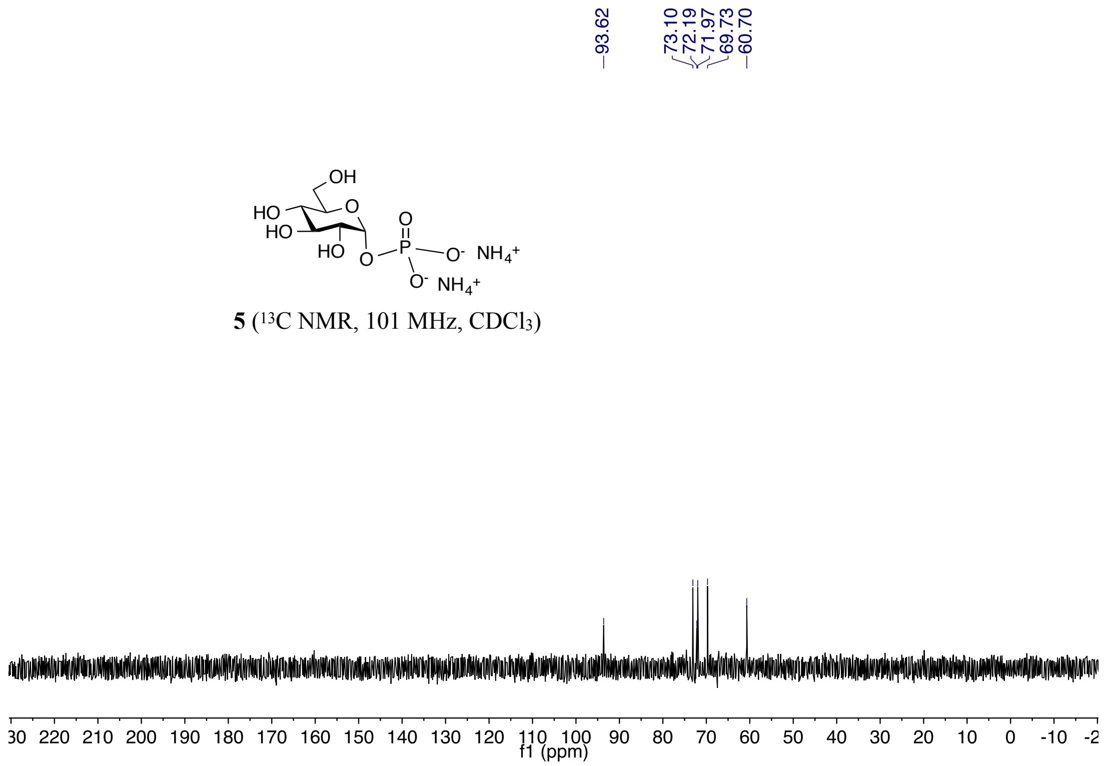

Supplementary Figure 258. ^{31}P NMR spectrum of compound **5**

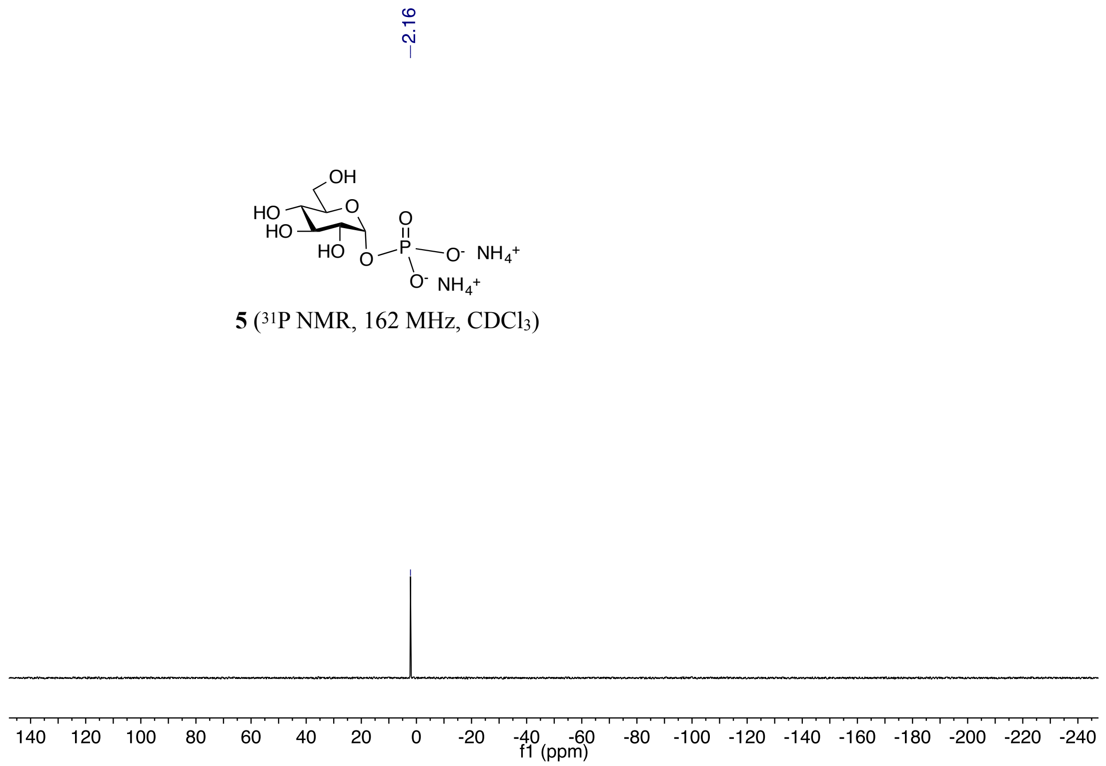

Supplementary Figure 259. ^{1}H NMR spectrum of compound **6**

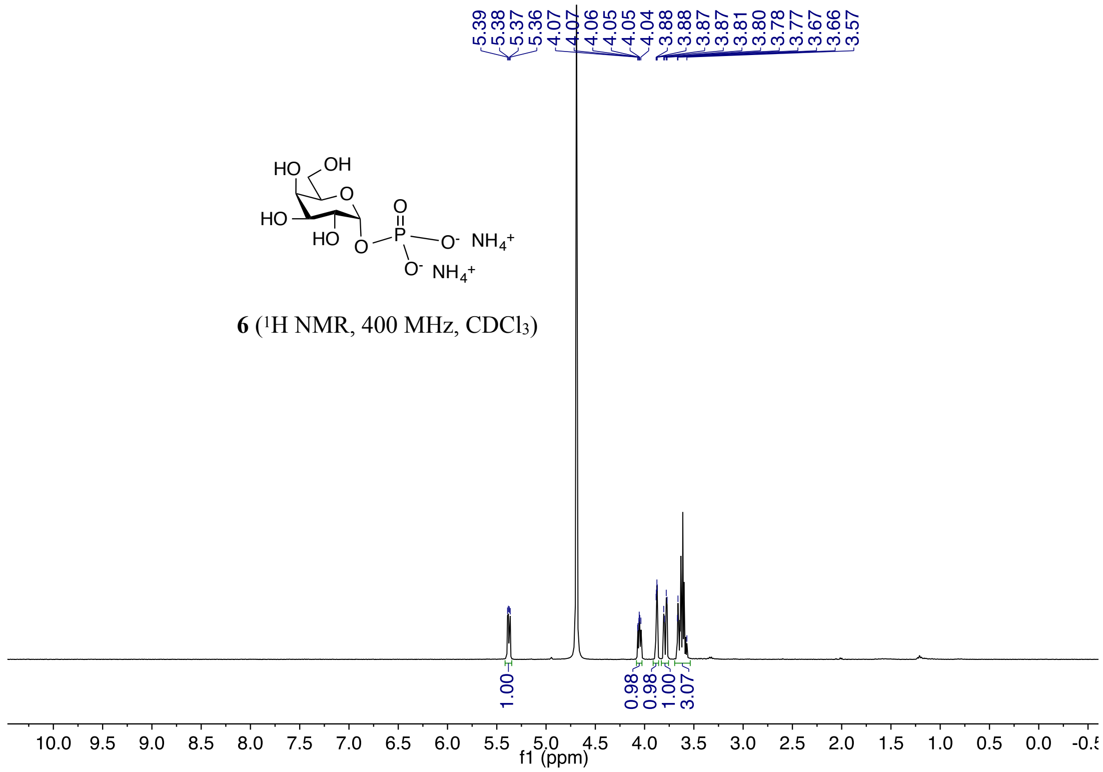

Supplementary Figure 260. ^{13}C NMR spectrum of compound **6**

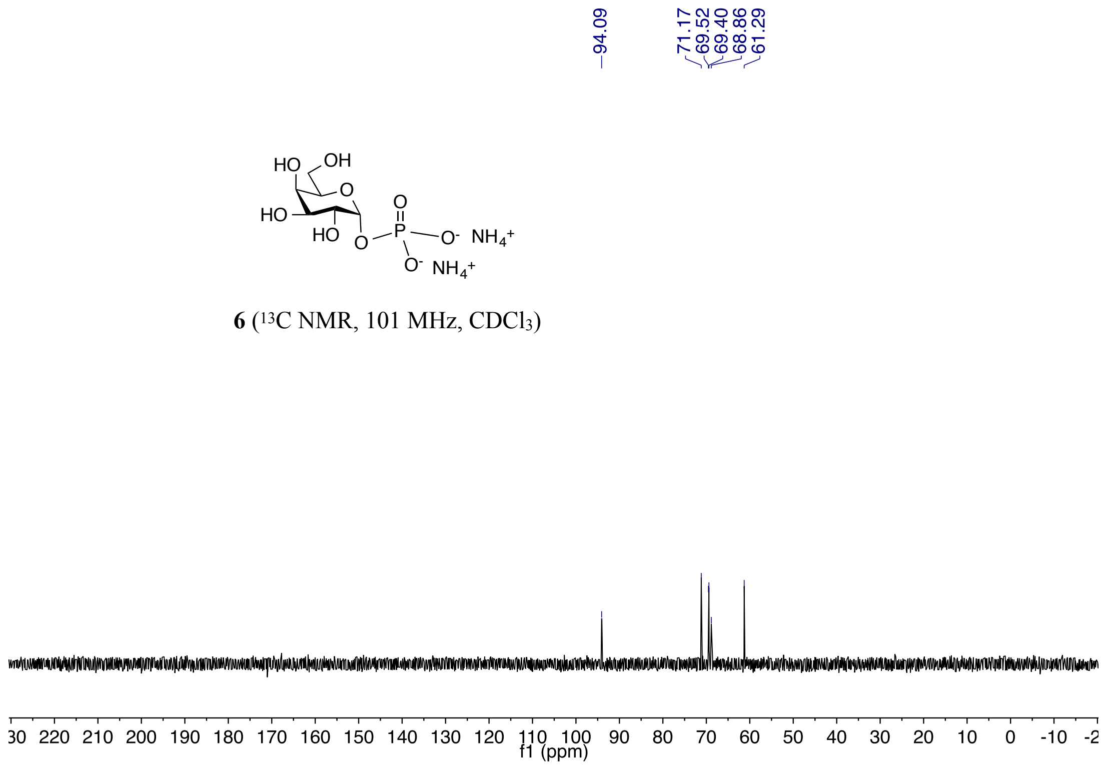

Supplementary Figure 261. ^{31}P NMR spectrum of compound **6**

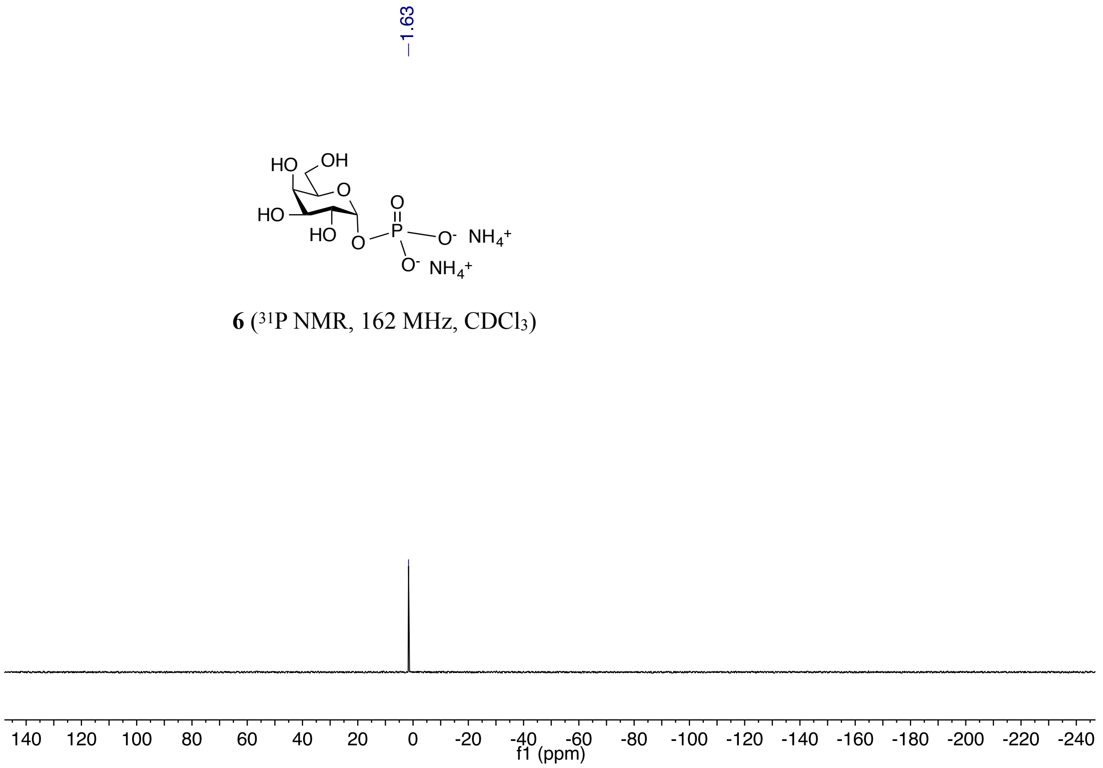

Supplementary Figure 262. ^{1}H NMR spectrum of compound **7**

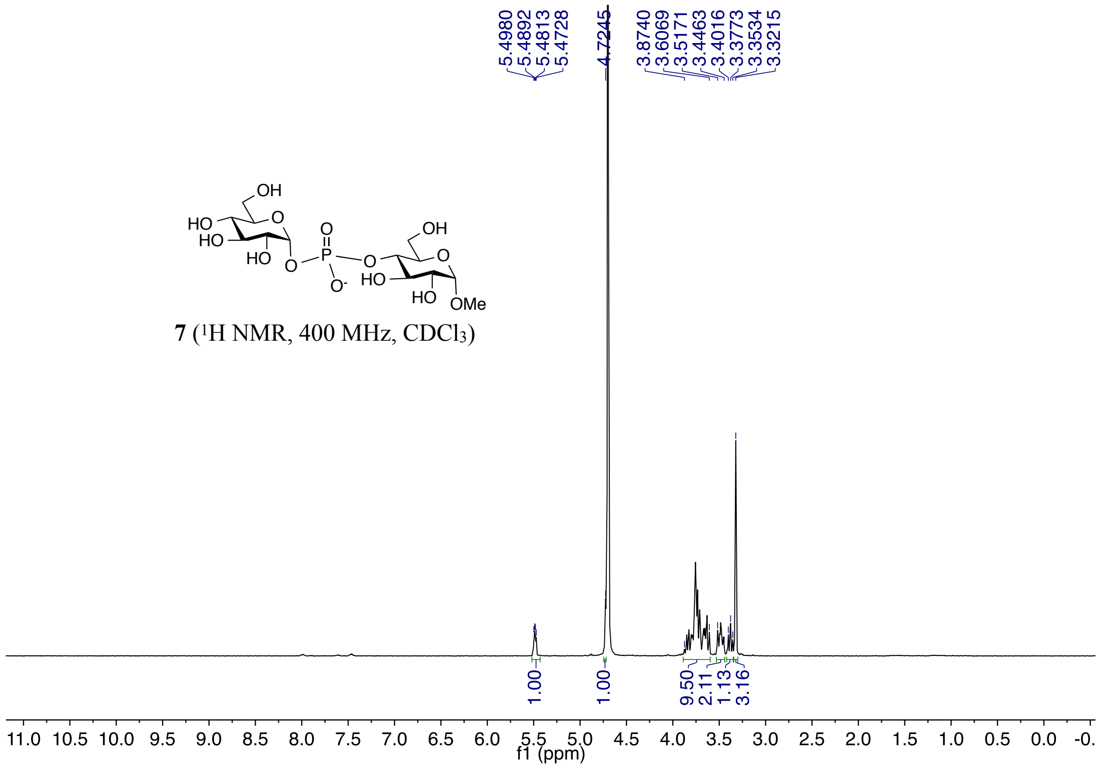

Supplementary Figure 263. ^{13}C NMR spectrum of compound **7**

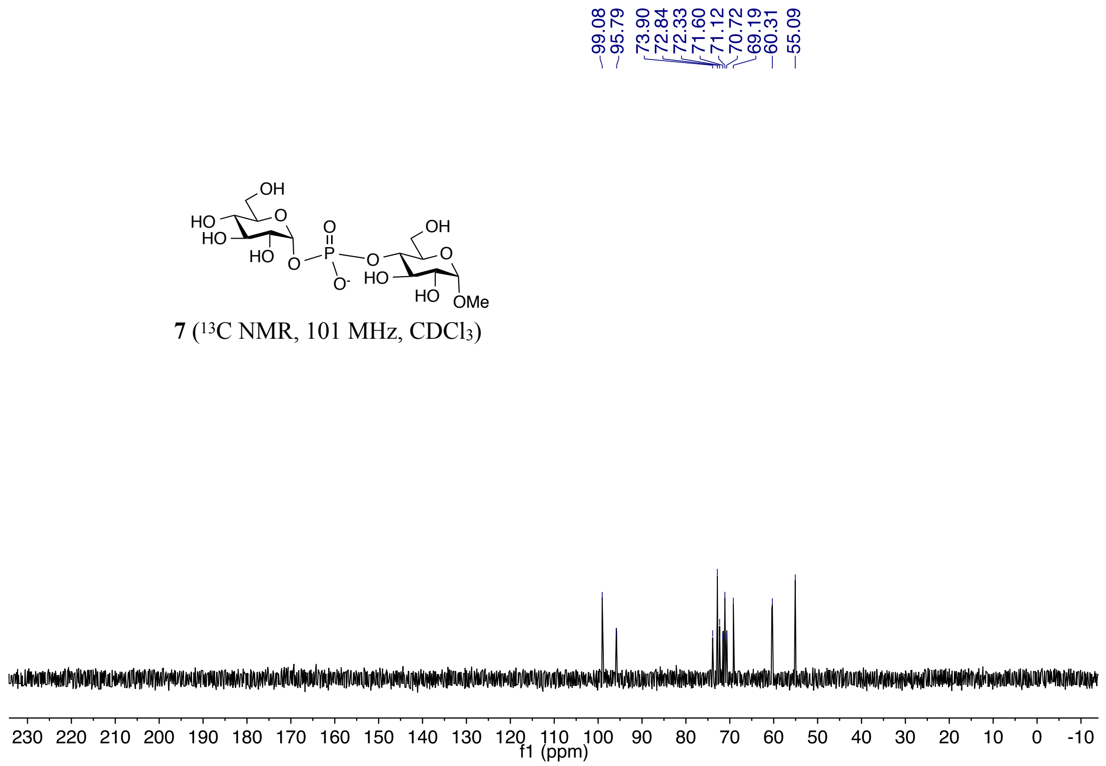

Supplementary Figure 264. ^{31}P NMR spectrum of compound **7**

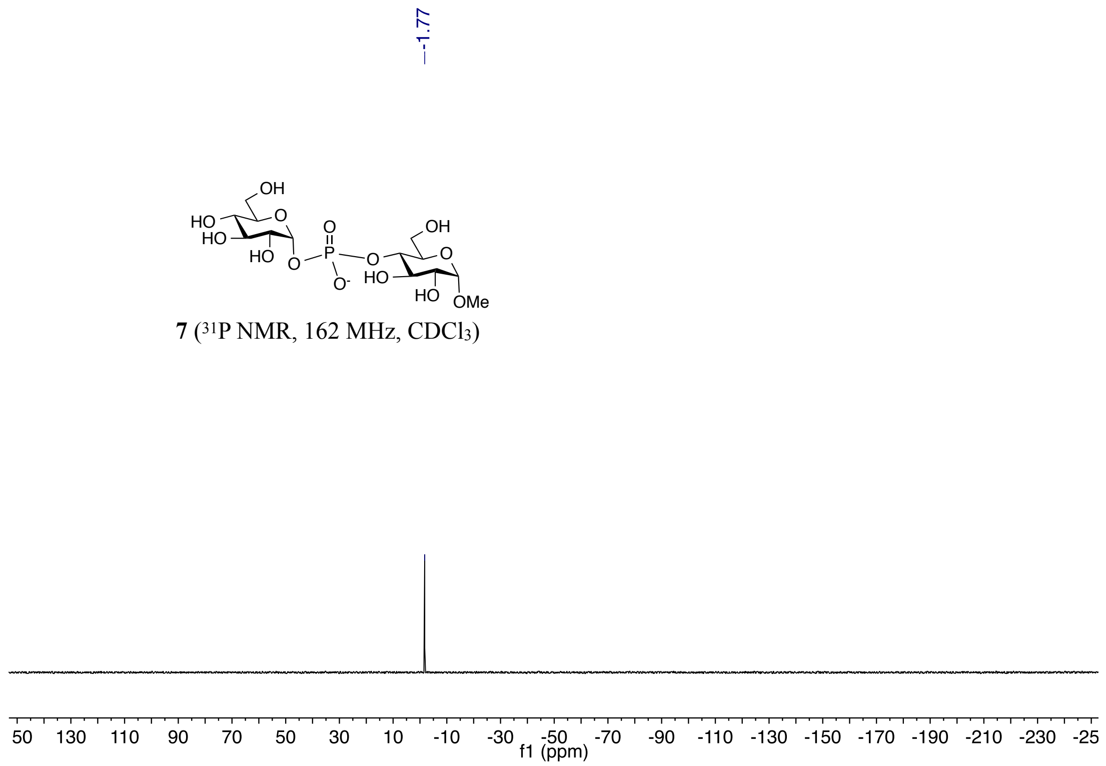

Supplementary Figure 265. ^{1}H NMR spectrum of compound **8**

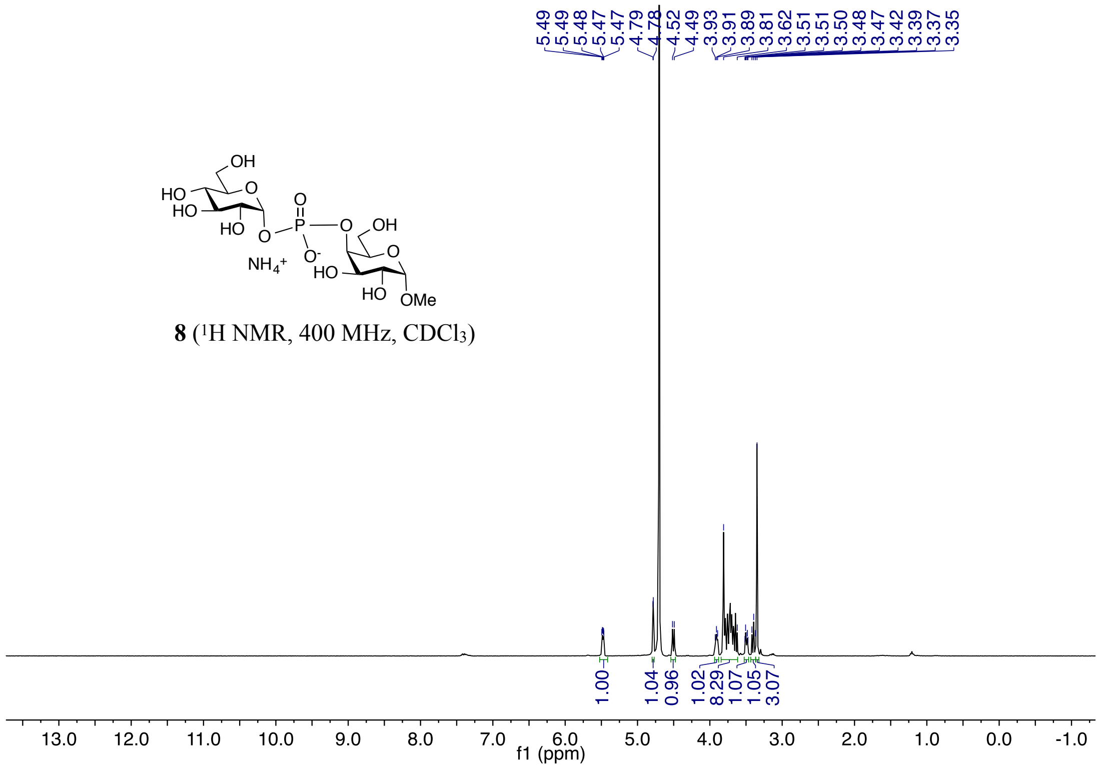

Supplementary Figure 266. ^{13}C NMR spectrum of compound **8**

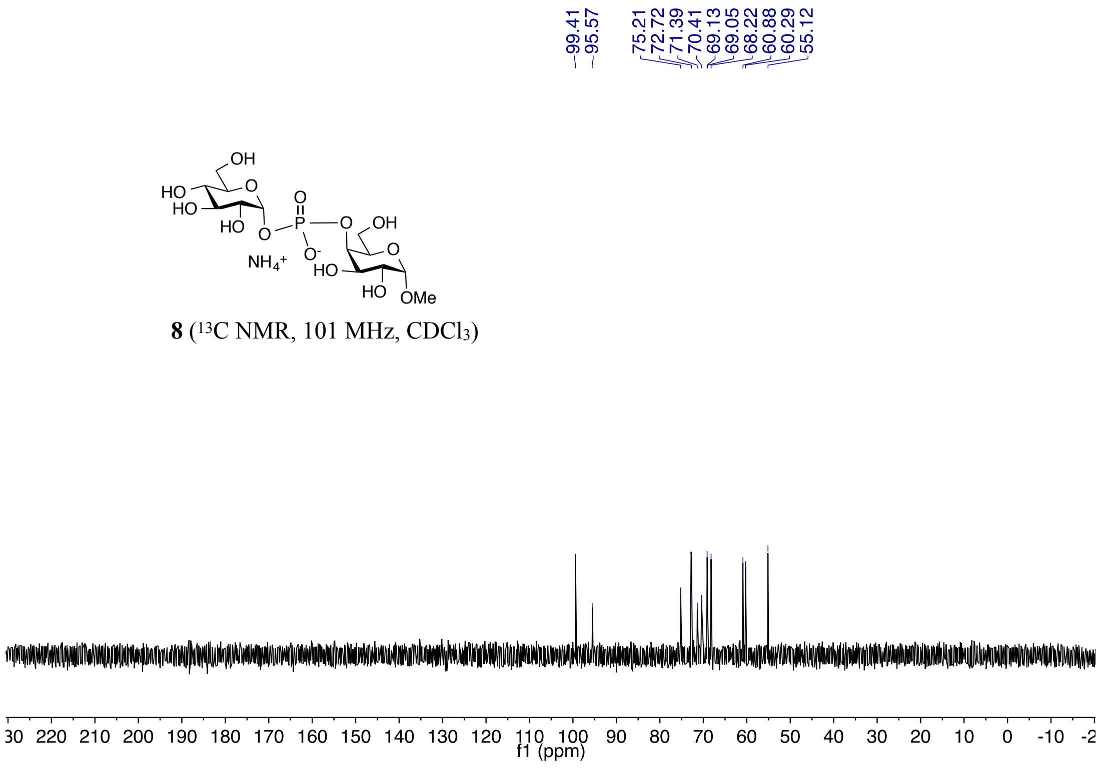

Supplementary Figure 267. ^{31}P NMR spectrum of compound **8**

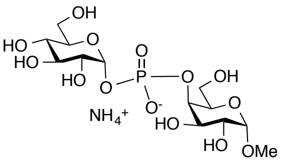

8 (^{31}P NMR, 162 MHz, $CDCl_3$)

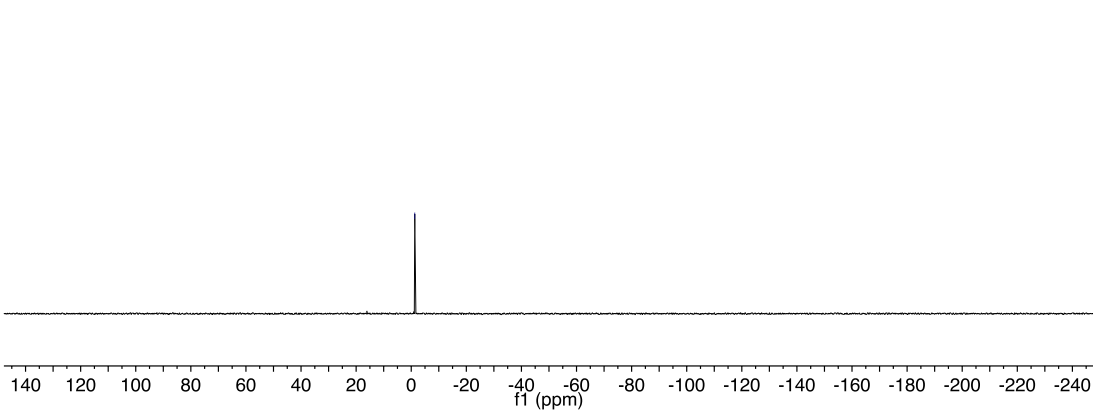

Supplementary Figure 268. ^{1}H NMR spectrum of compound **S12**

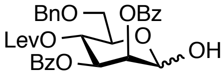

S12 (^{1}H NMR, 400 MHz, $CDCl_3$)

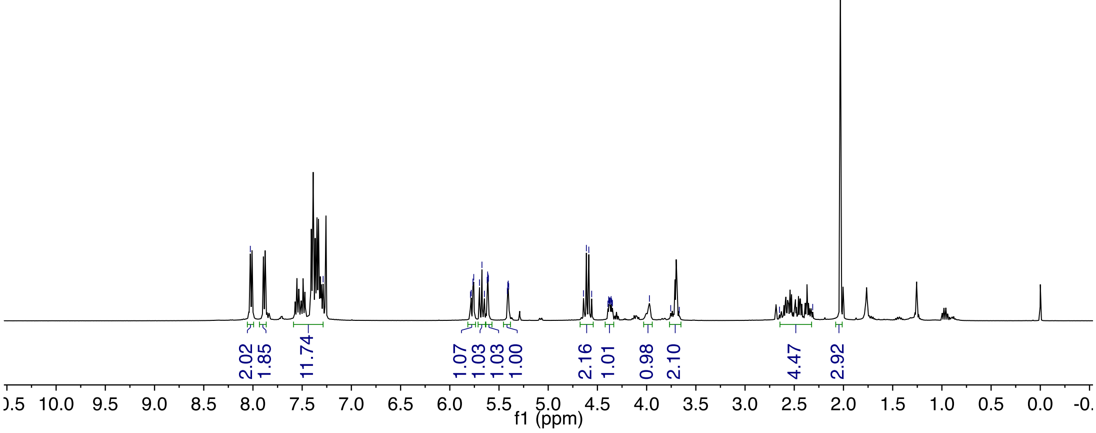

Supplementary Figure 269. ^{13}C NMR spectrum of compound **S12**

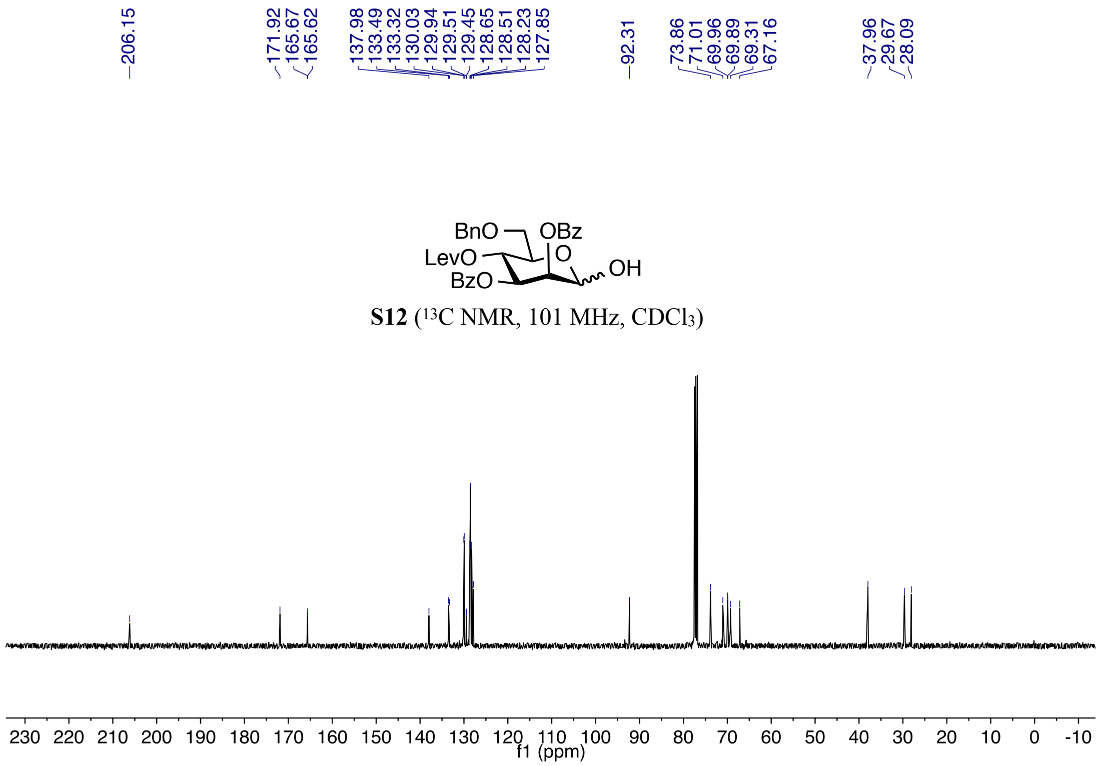

Supplementary Figure 270. ^{1}H NMR spectrum of compound **1p**

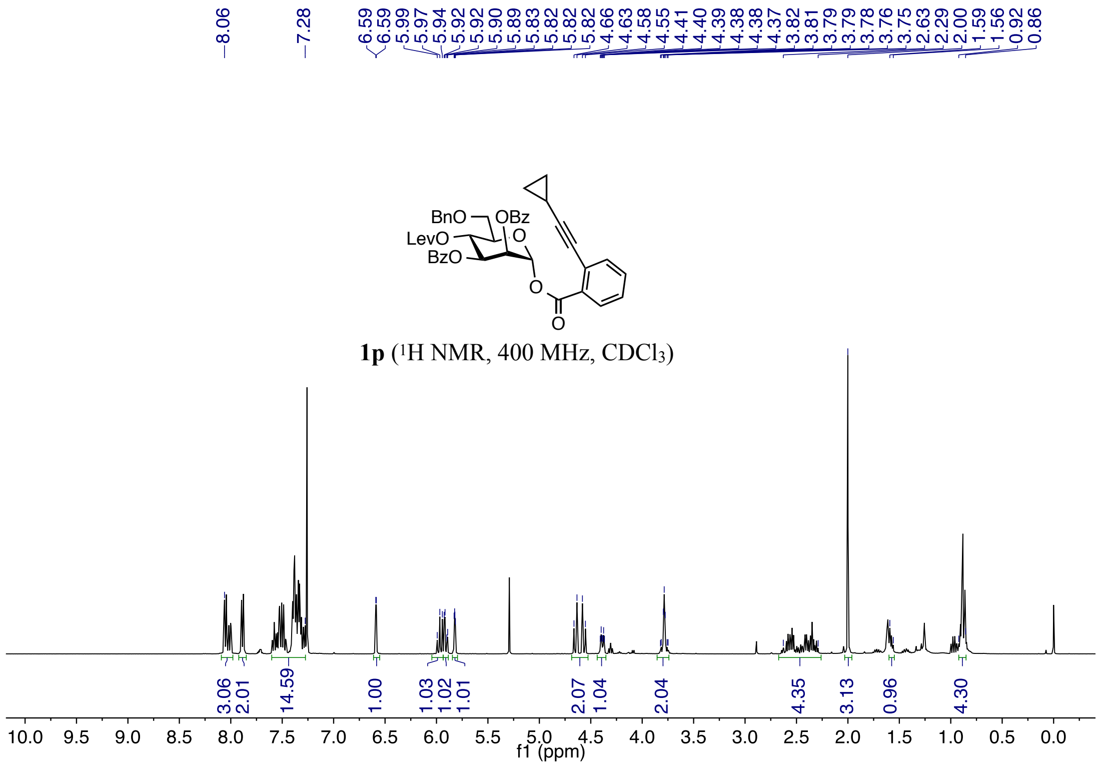

Supplementary Figure 271. ^{13}C NMR spectrum of compound **1p**

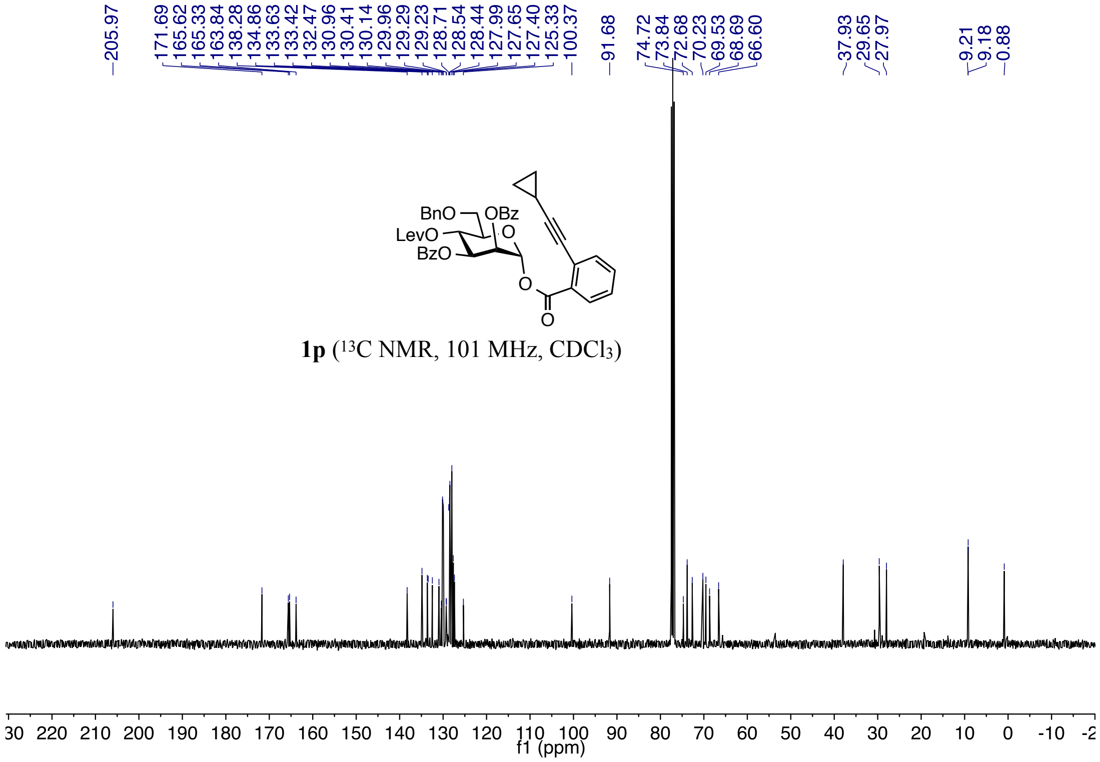

Supplementary Figure 272. ^{1}H NMR spectrum of compound **S13**

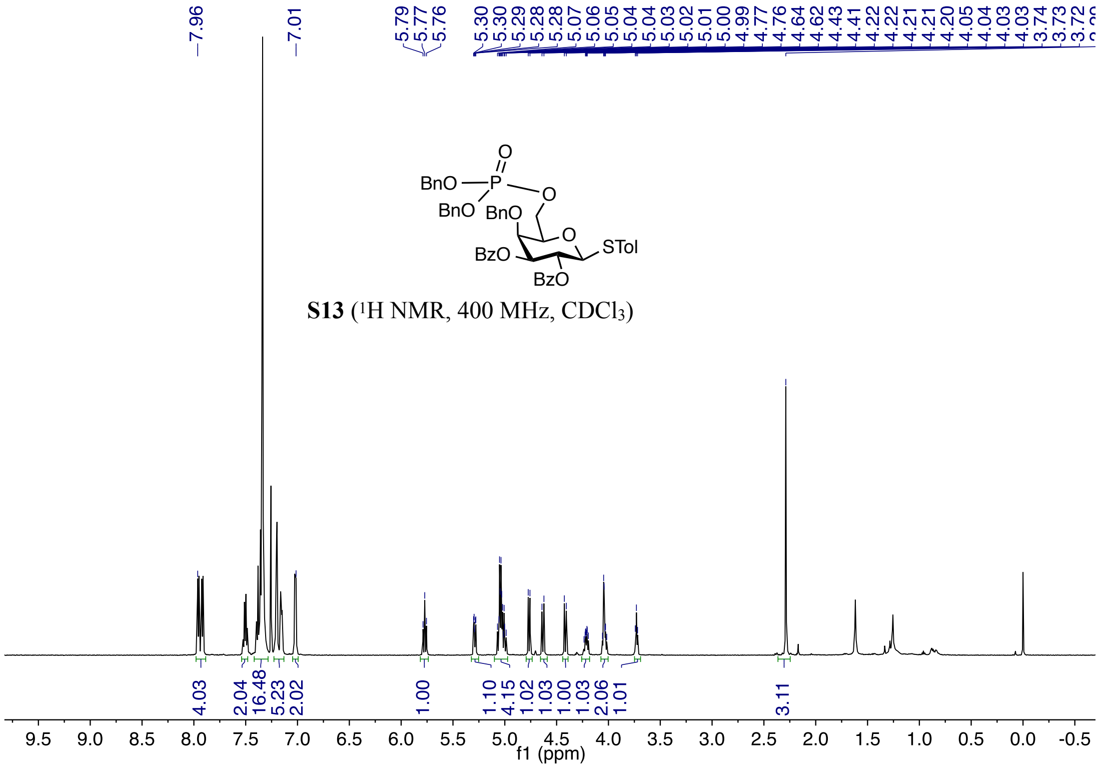

Supplementary Figure 273. ^{13}C NMR spectrum of compound **S13**

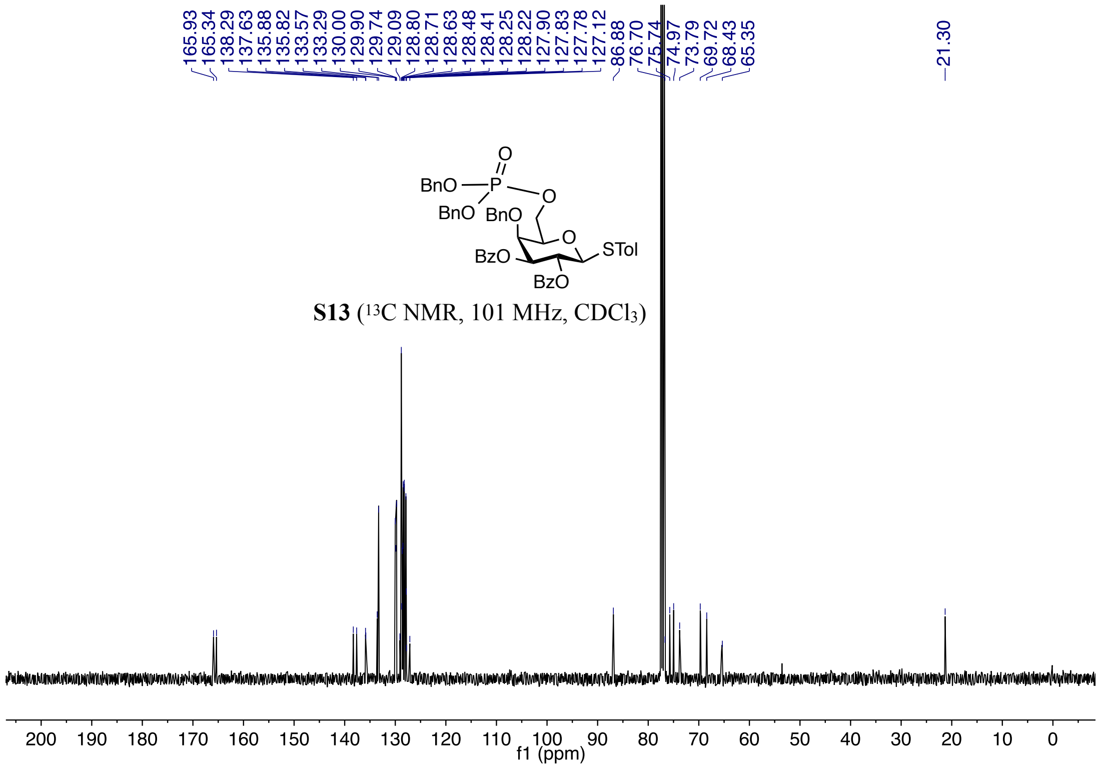

Supplementary Figure 274. ^{31}P NMR spectrum of compound **S13**

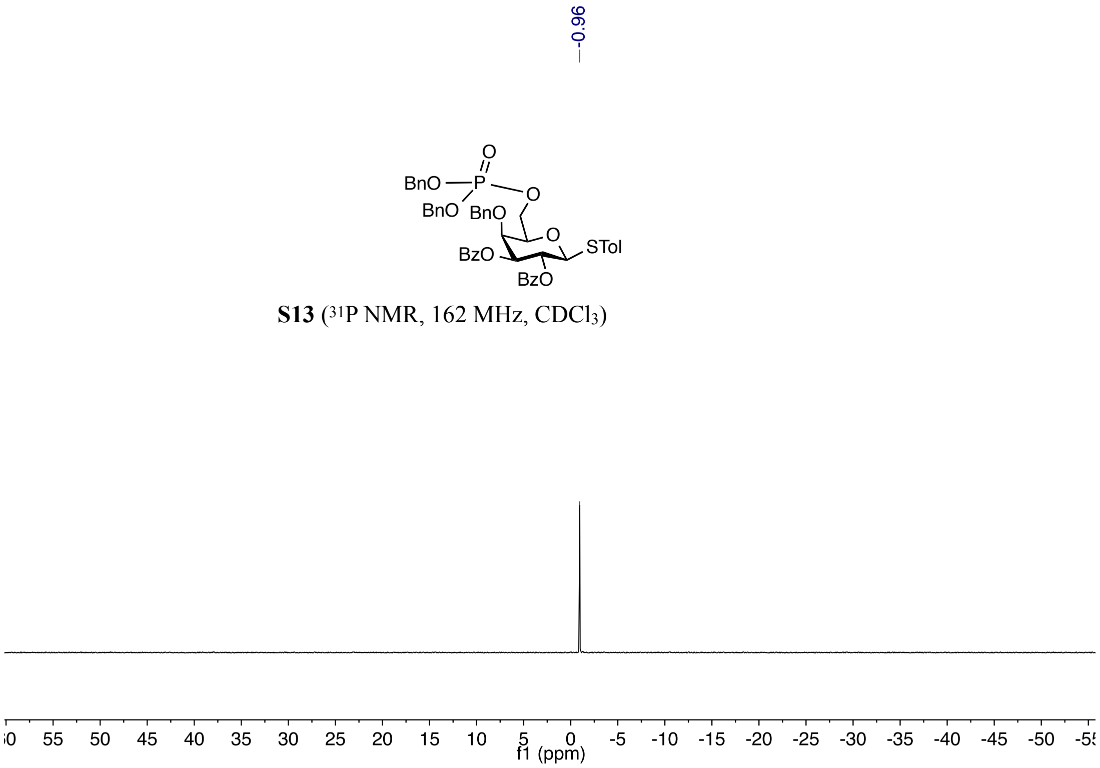

Supplementary Figure 275. ^{1}H NMR spectrum of compound **2h**

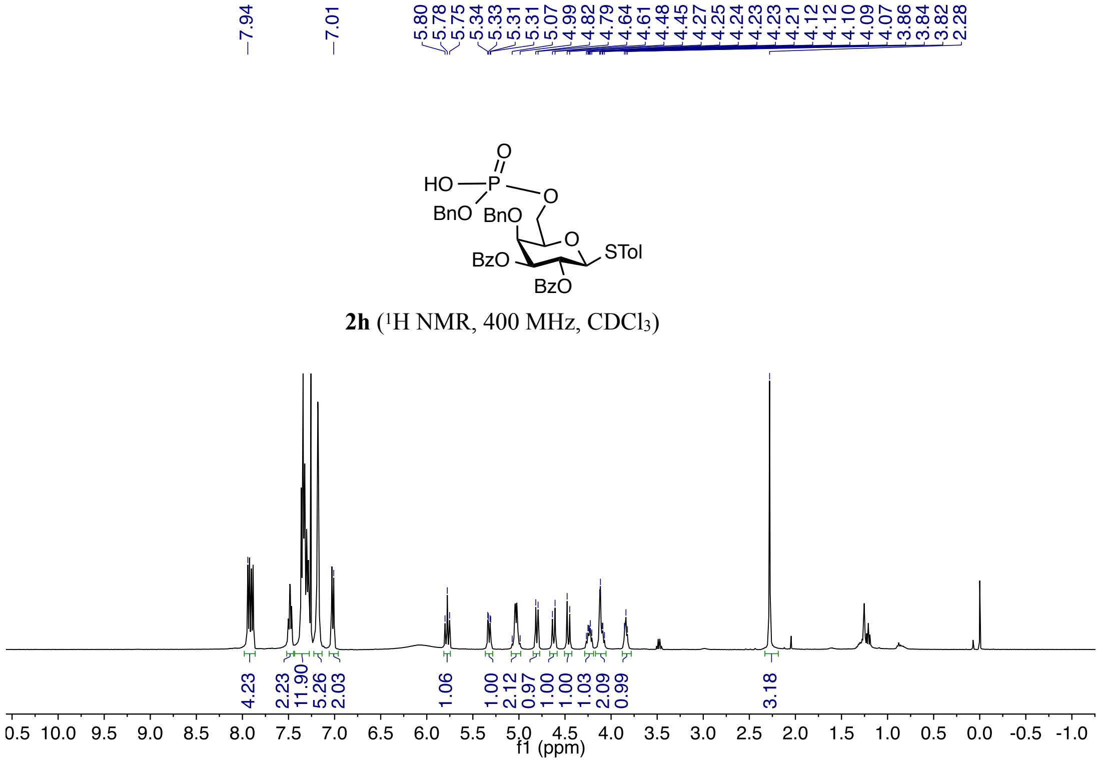

Supplementary Figure 276. ^{13}C NMR spectrum of compound **2h**

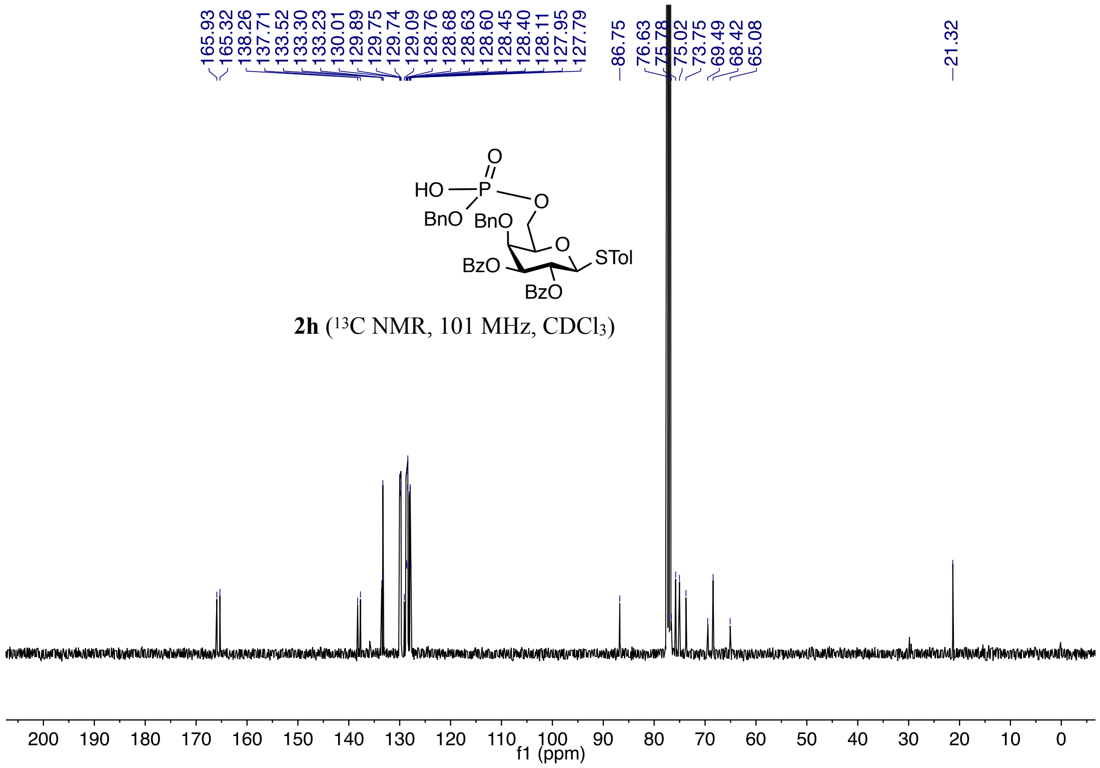

Supplementary Figure 277. ^{31}P NMR spectrum of compound **2h**

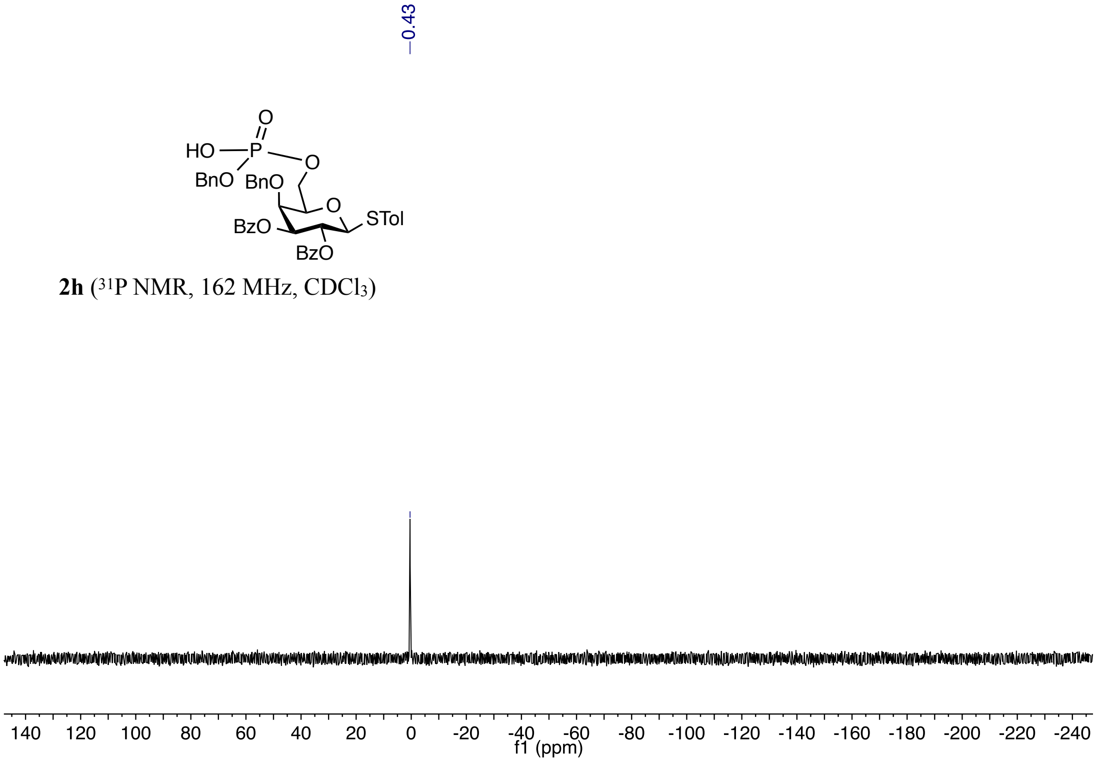

Supplementary Figure 278. ^{1}H NMR spectrum of compound **9**

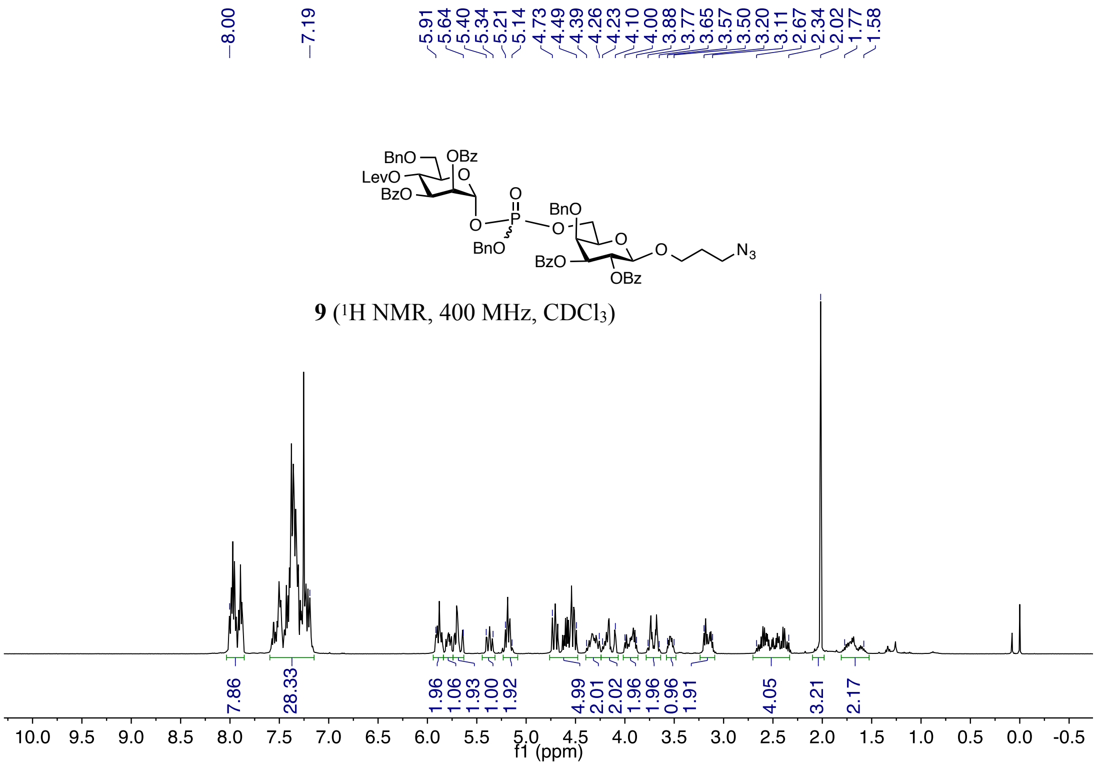

Supplementary Figure 279. ^{13}C NMR spectrum of compound **9**

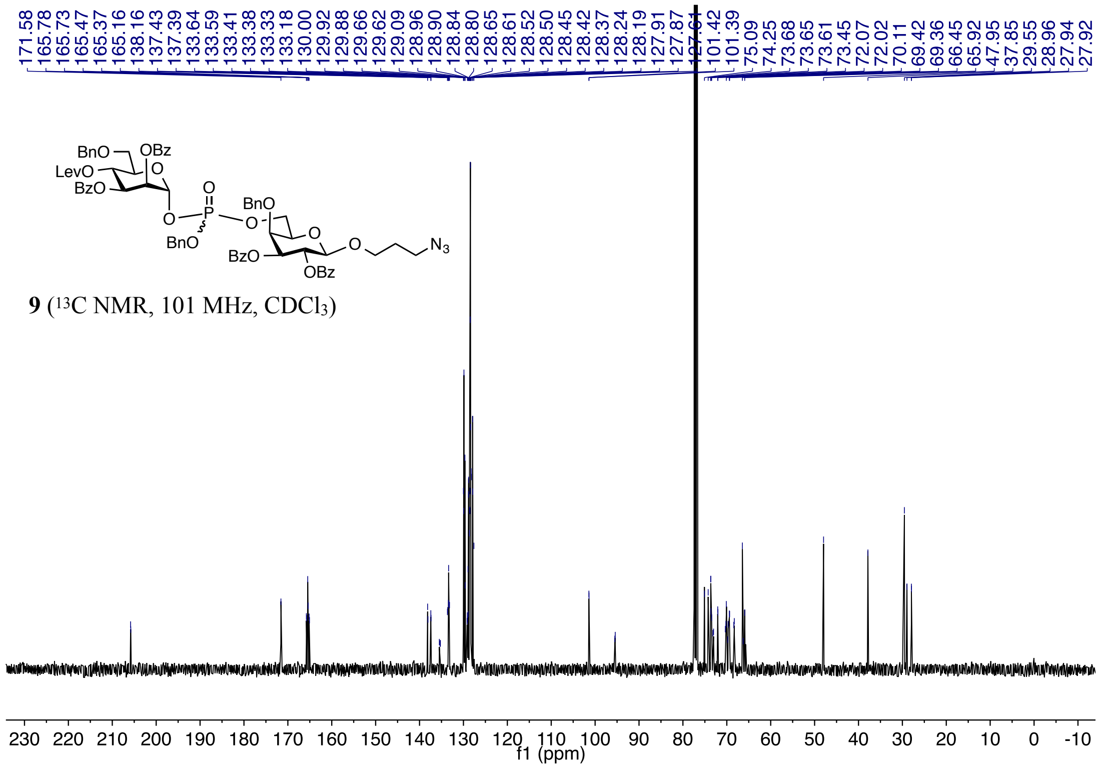

Supplementary Figure 280. ^{31}P NMR spectrum of compound **9**

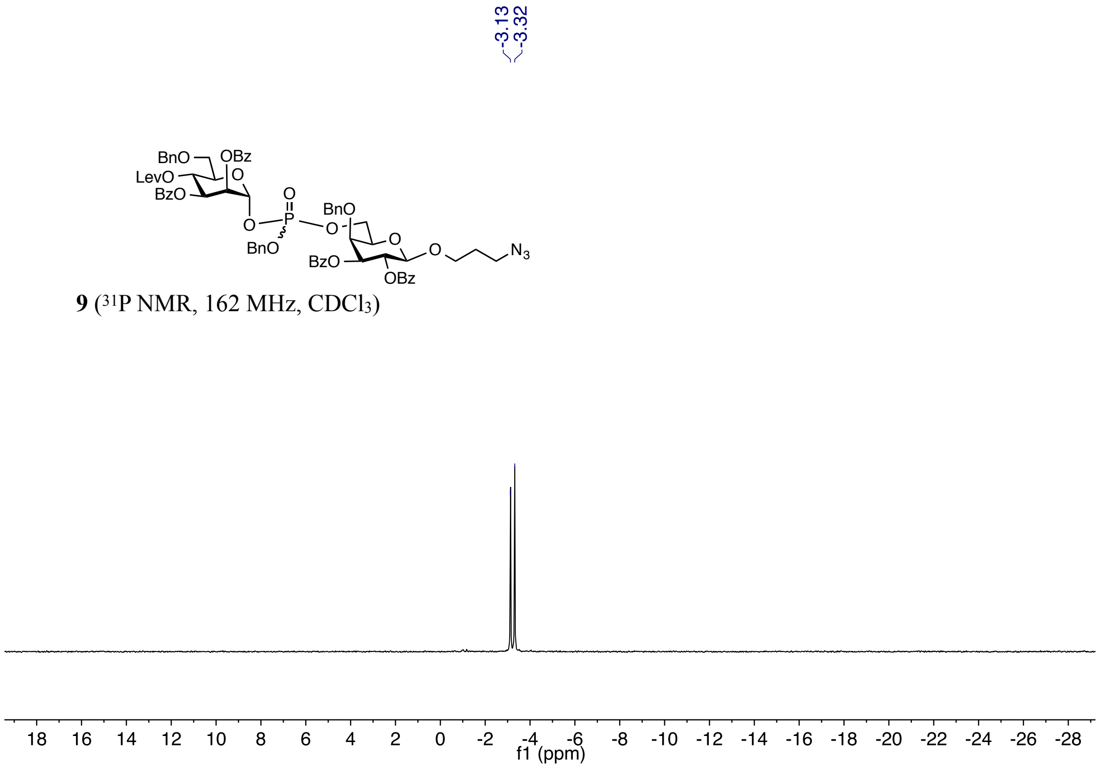

Supplementary Figure 281. ^{1}H NMR spectrum of compound **10**

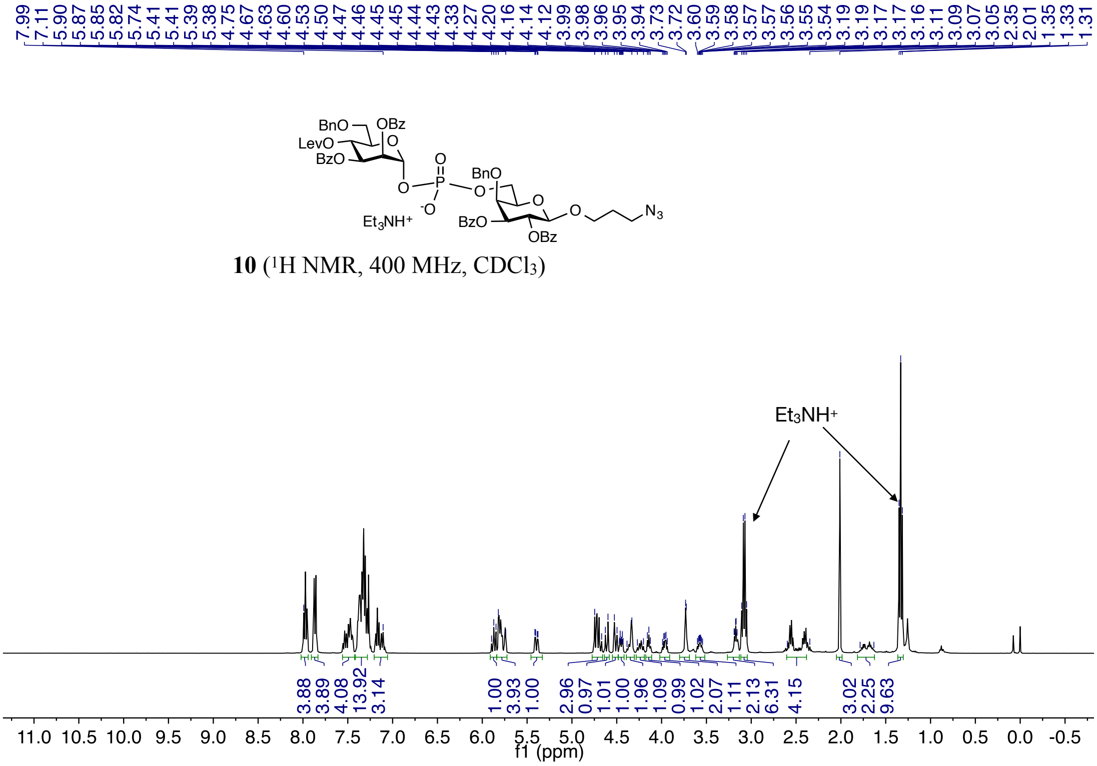

Supplementary Figure 282. ^{13}C NMR spectrum of compound **10**

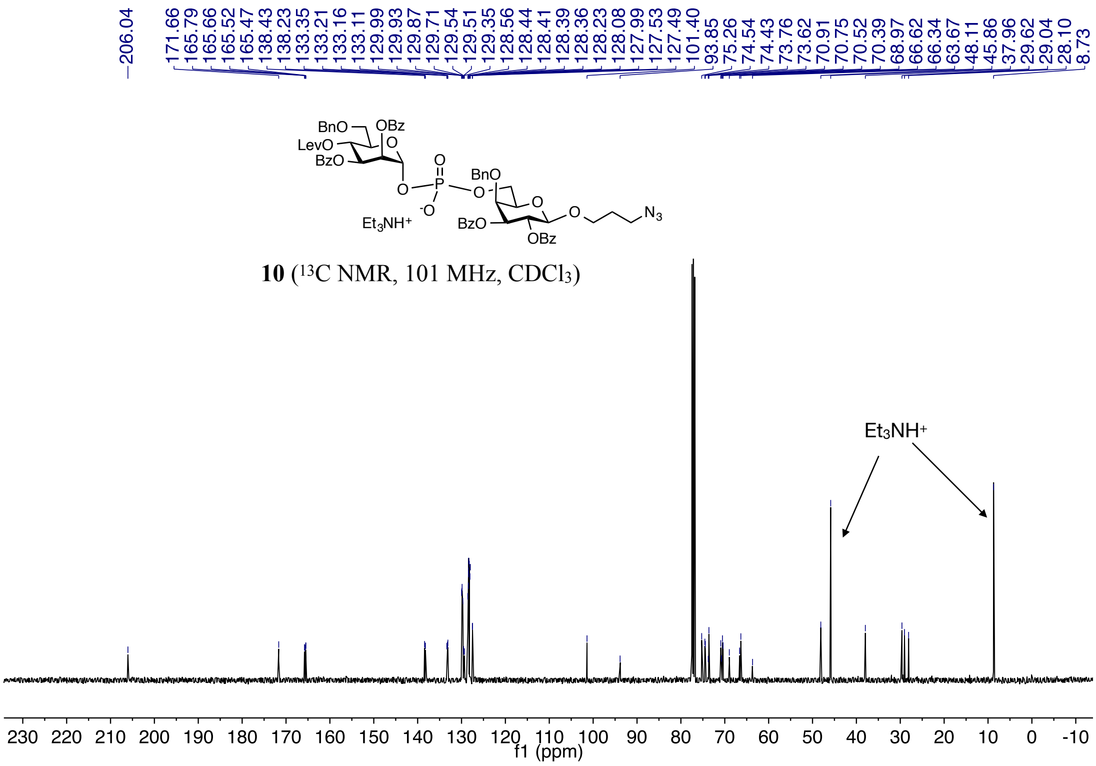

Supplementary Figure 283 ^{31}P NMR spectrum of compound **10**

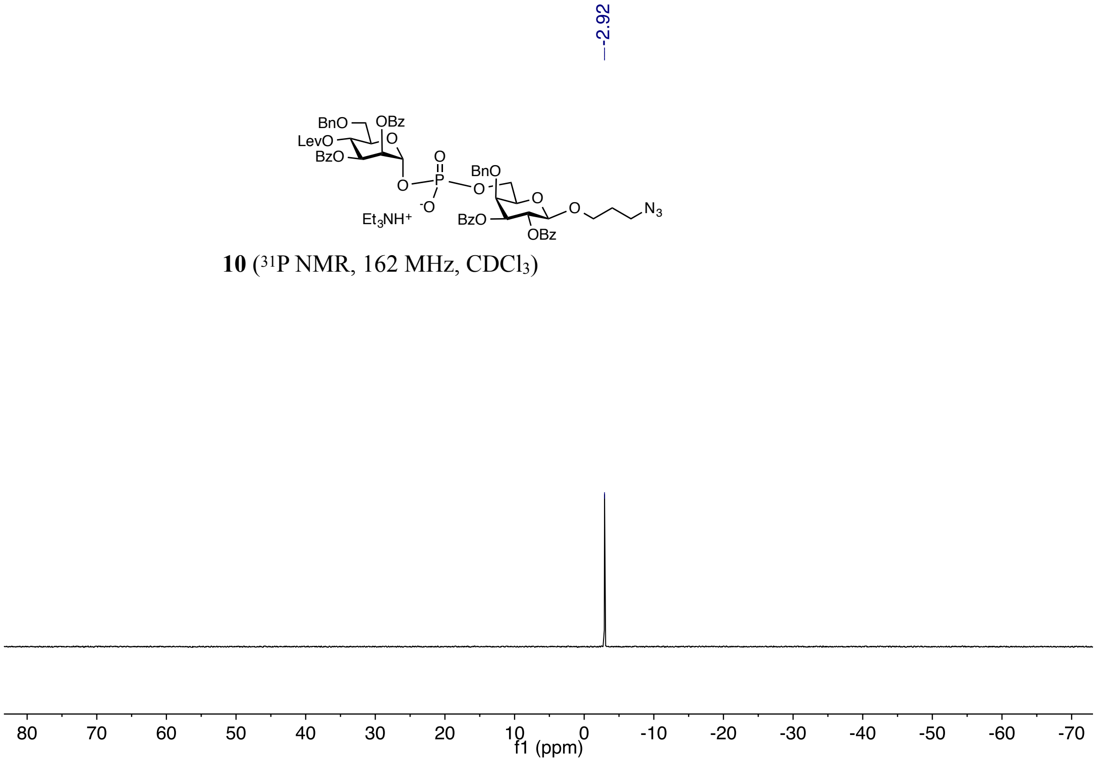

10 (^{31}P NMR, 162 MHz, $CDCl_3$)

Supplementary Figure 284. ^{1}H NMR spectrum of compound **1q**

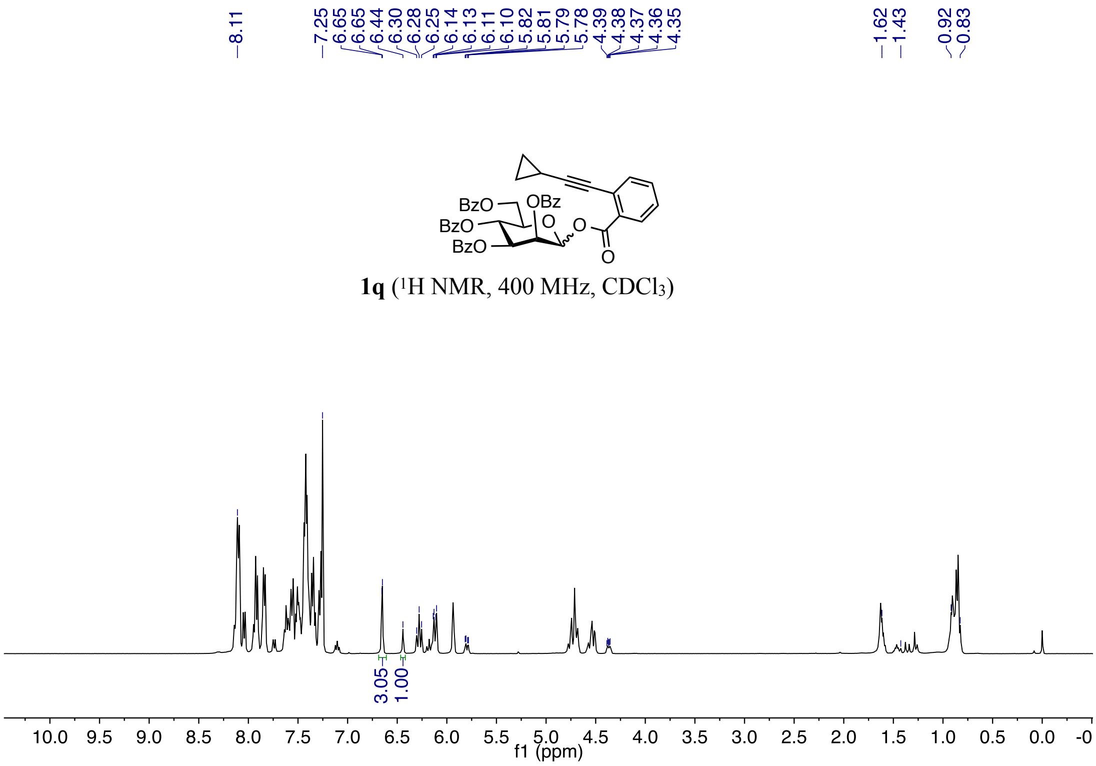

1q (^{1}H NMR, 400 MHz, $CDCl_3$)

Supplementary Figure 285. ^{13}C NMR spectrum of compound **1q**

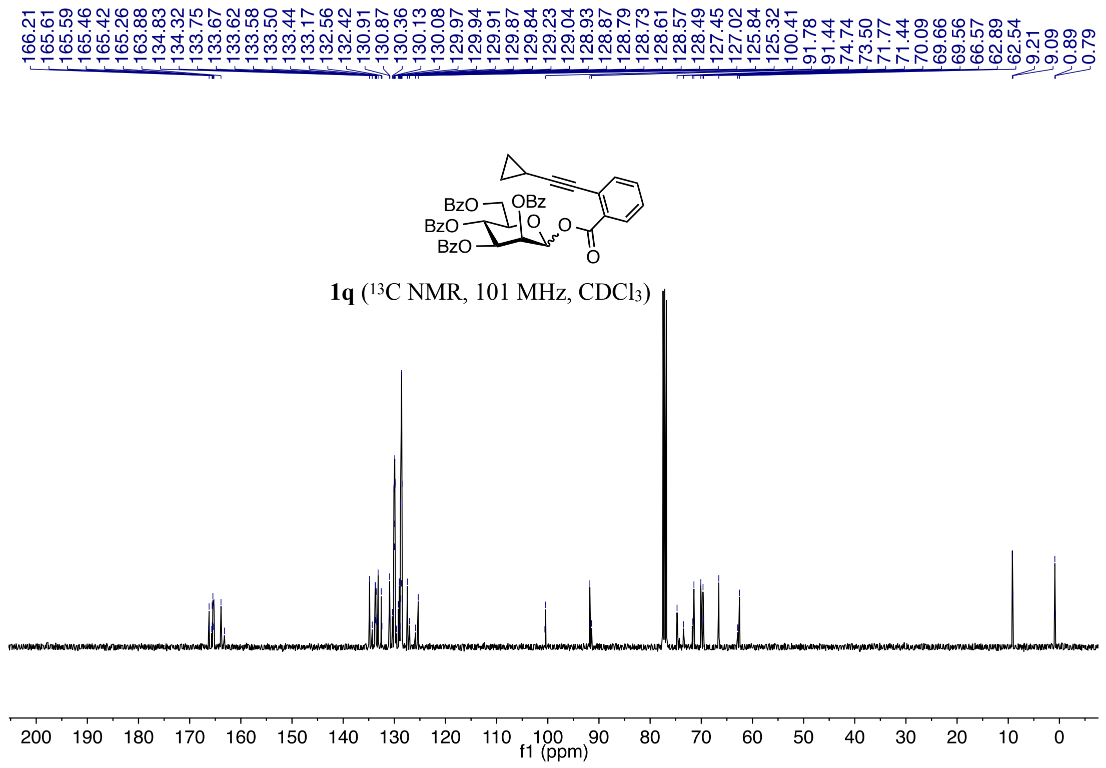

Supplementary Figure 286. ^{1}H NMR spectrum of compound **S14**

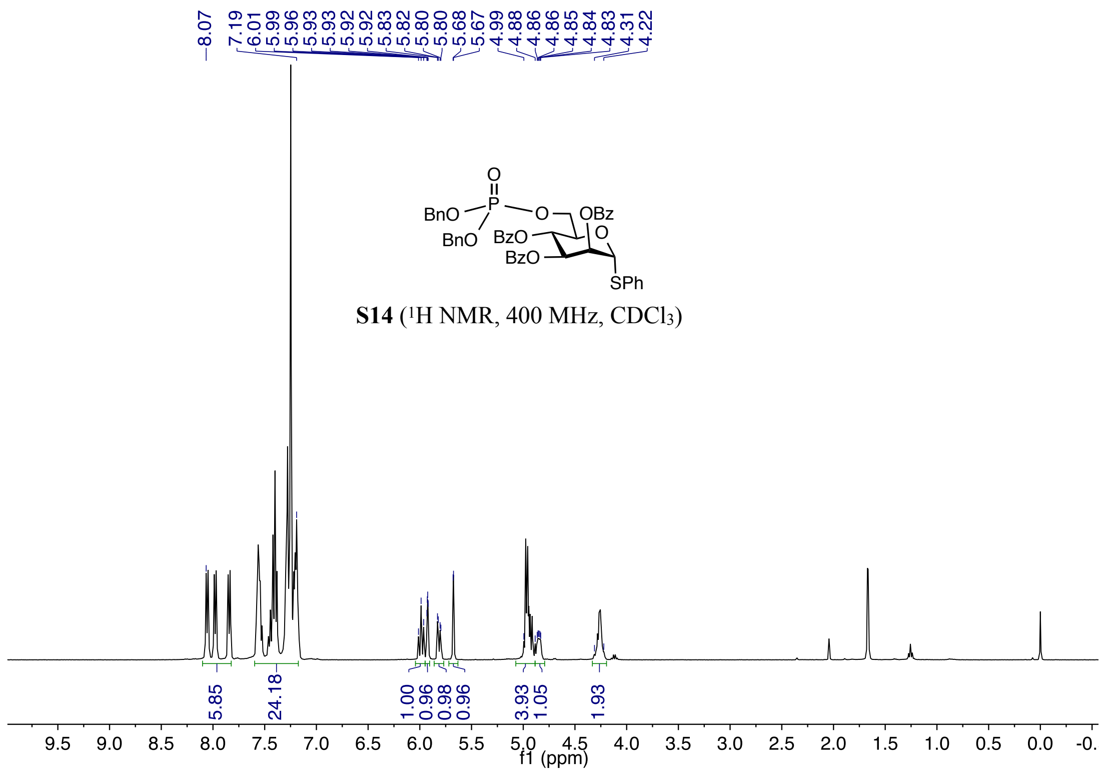

Supplementary Figure 287. ^{13}C NMR spectrum of compound **S14**

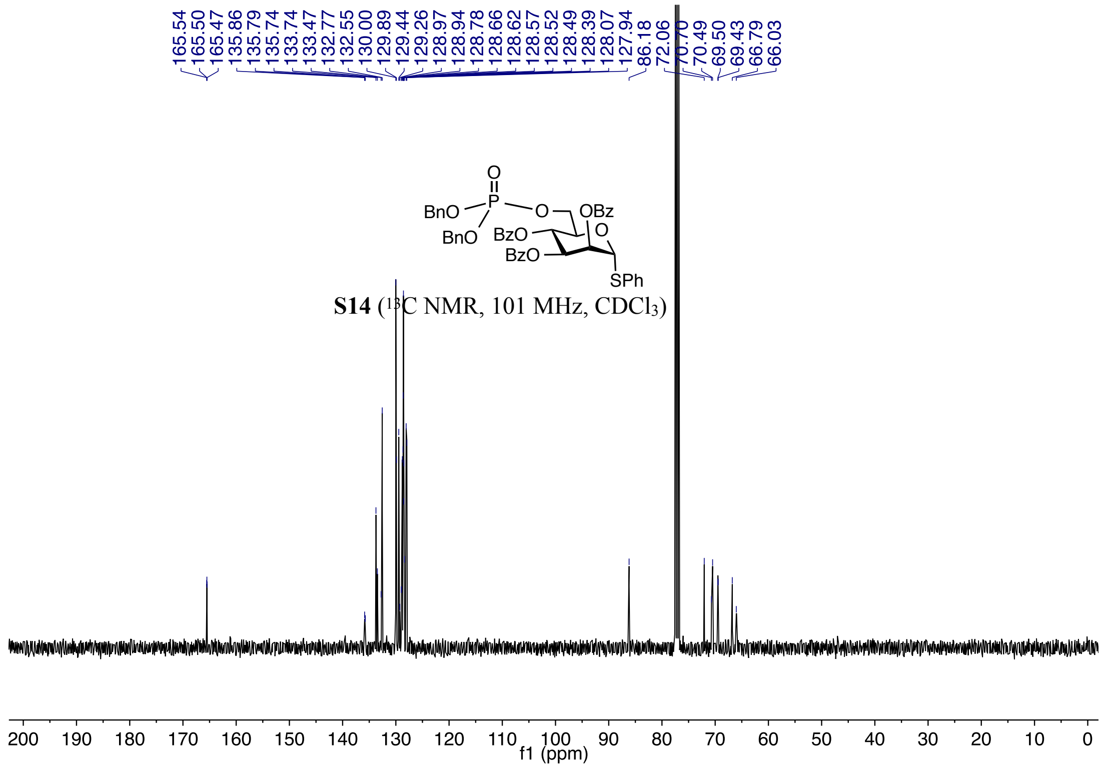

Supplementary Figure 288. ^{31}P NMR spectrum of compound S**14**

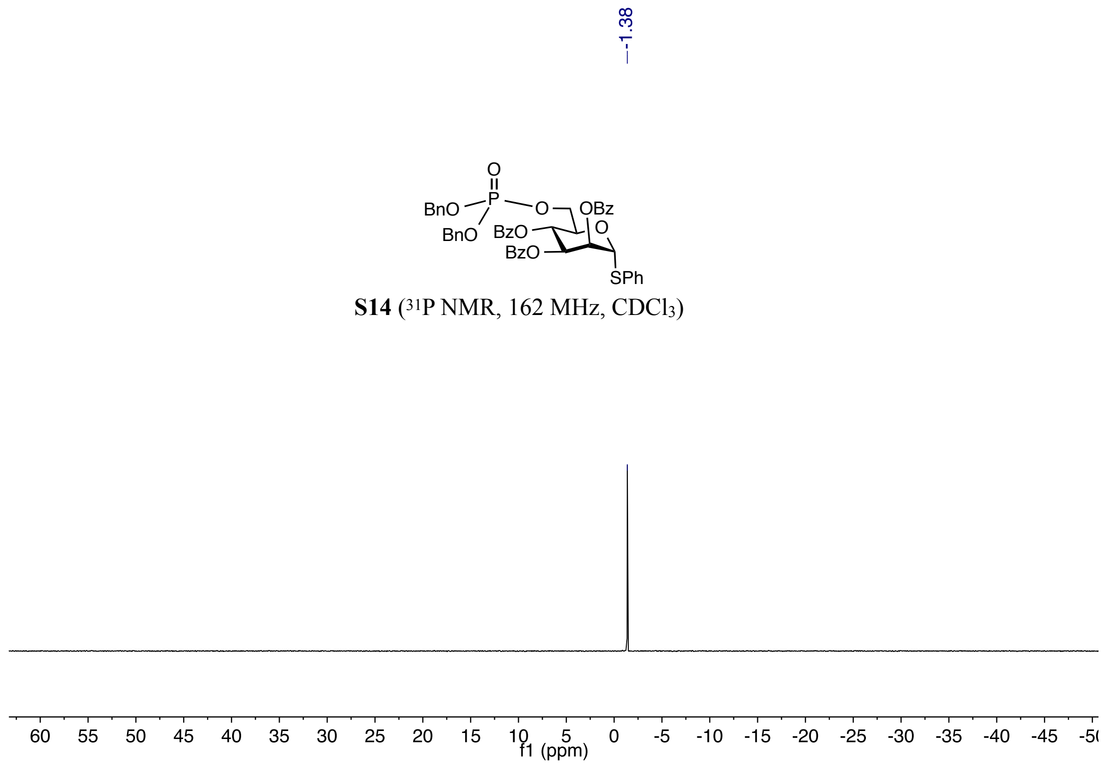

Supplementary Figure 289. ^{1}H NMR spectrum of compound **2i**

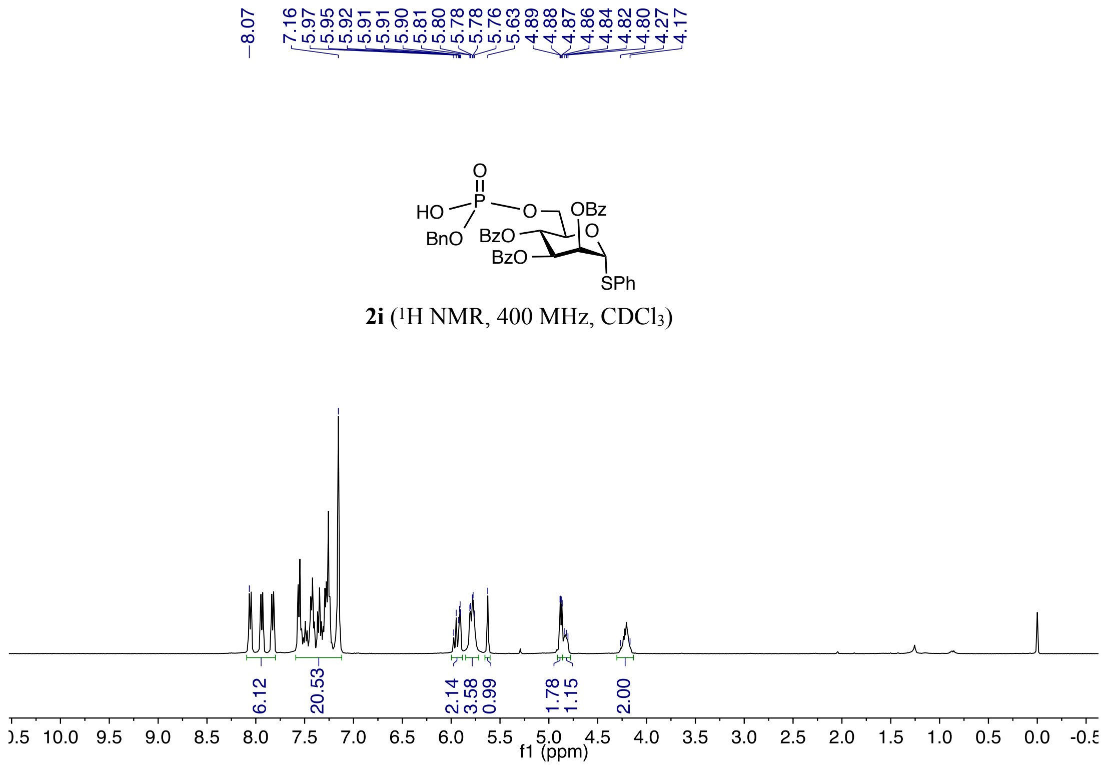

Supplementary Figure 290. ^{13}C NMR spectrum of compound **2i**

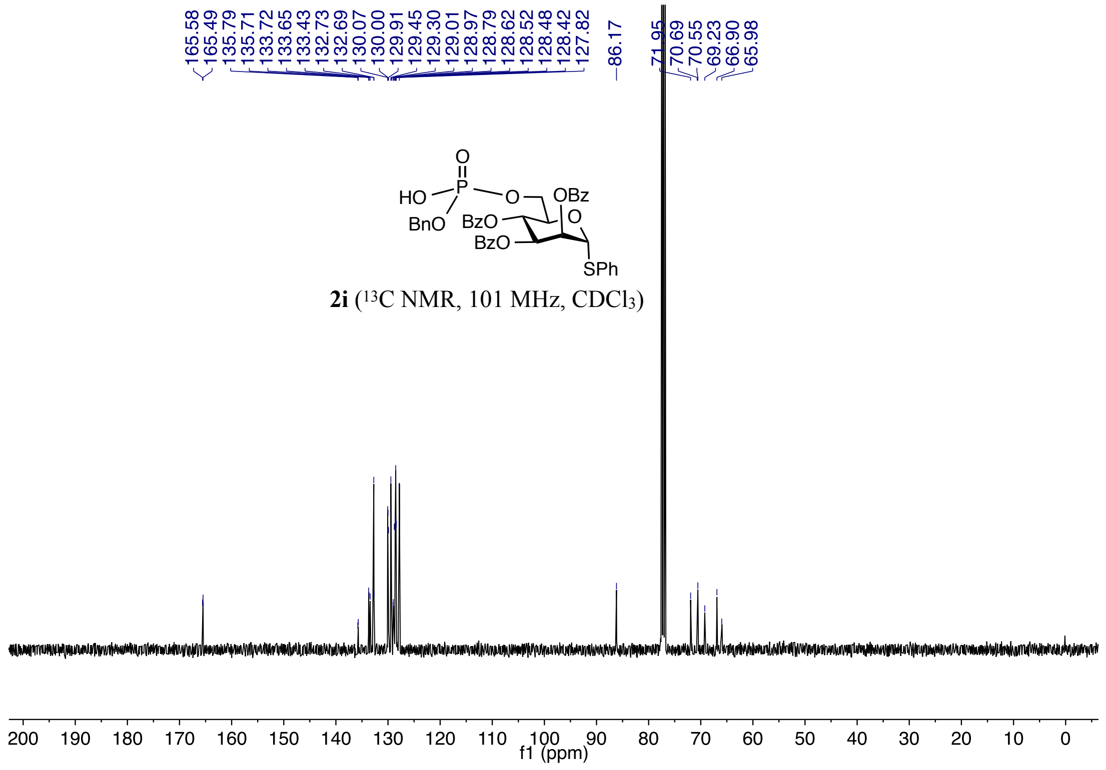

Supplementary Figure 291. ^{31}P NMR spectrum of compound **2i**

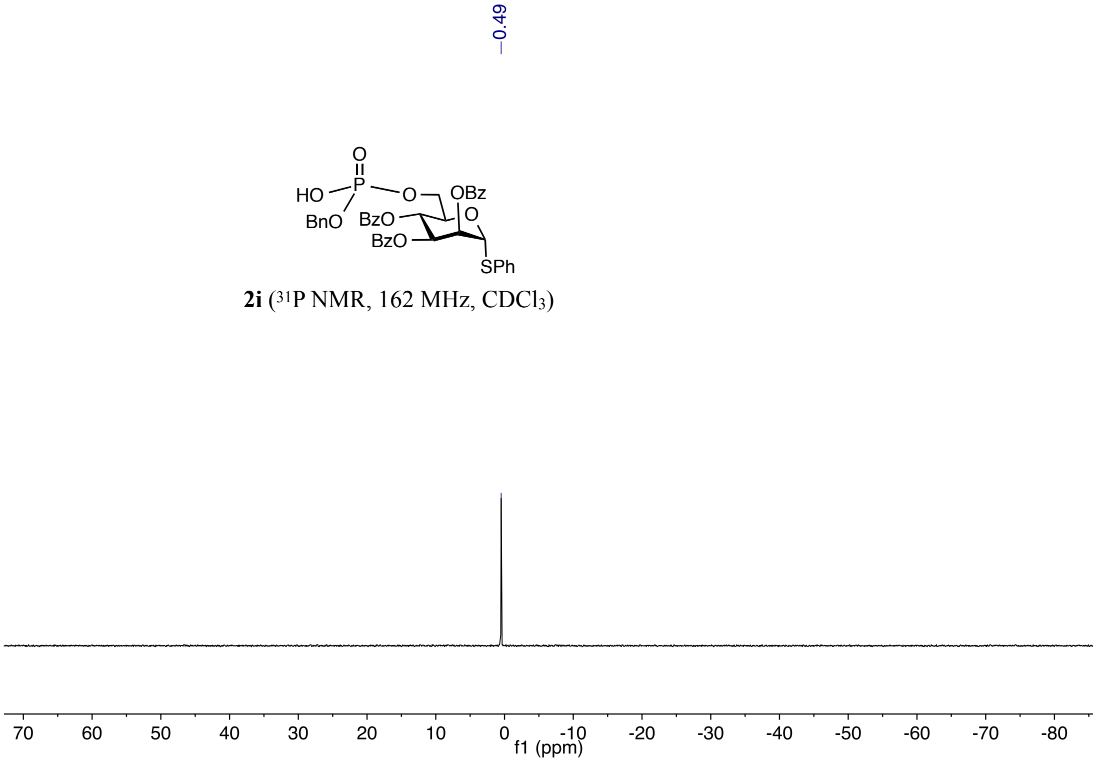

Supplementary Figure 292. ^{1}H NMR spectrum of compound **4zh-H2**

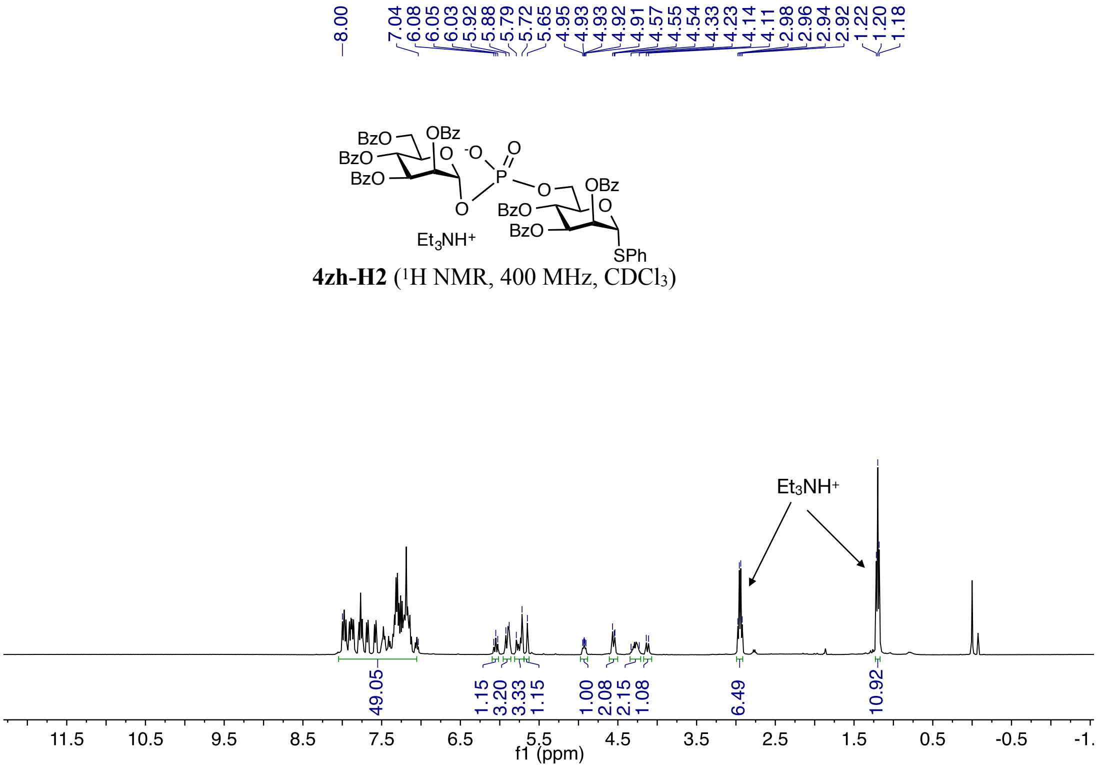

Supplementary Figure 293. ^{13}C NMR spectrum of compound **4zh-H2**

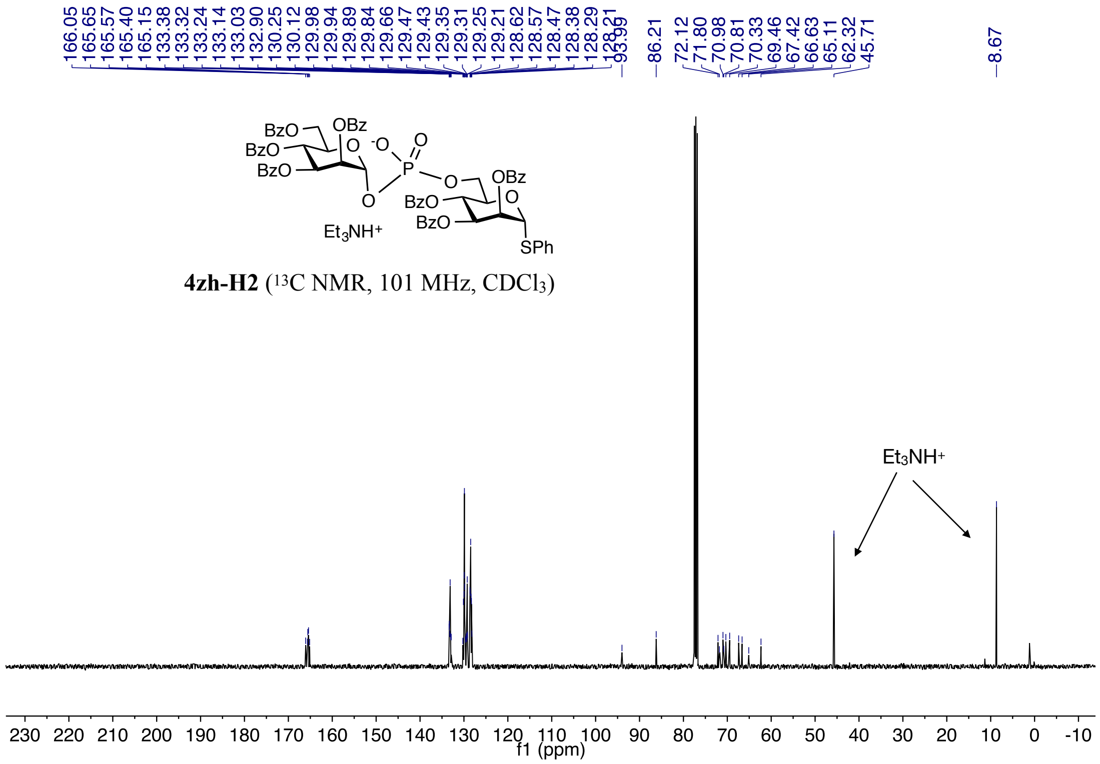

Supplementary Figure 294. ^{31}P NMR spectrum of compound **4zh-H2**

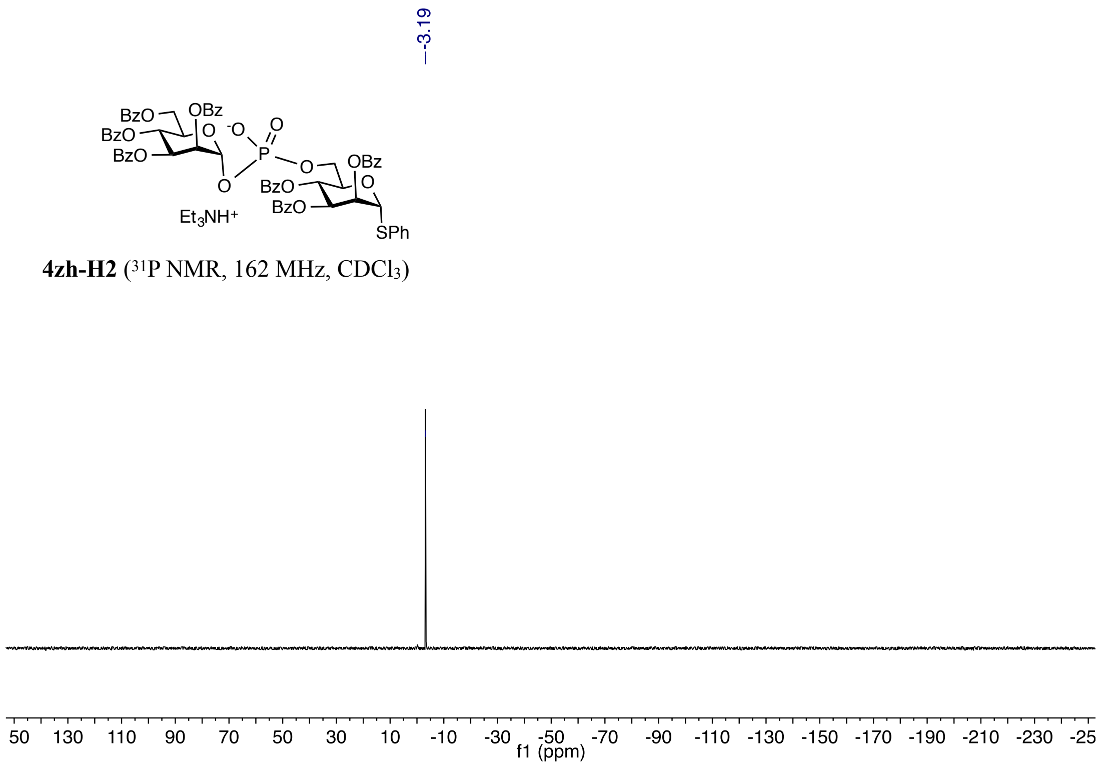

Supplementary Figure 295. ^{1}H NMR spectrum of compound **11**

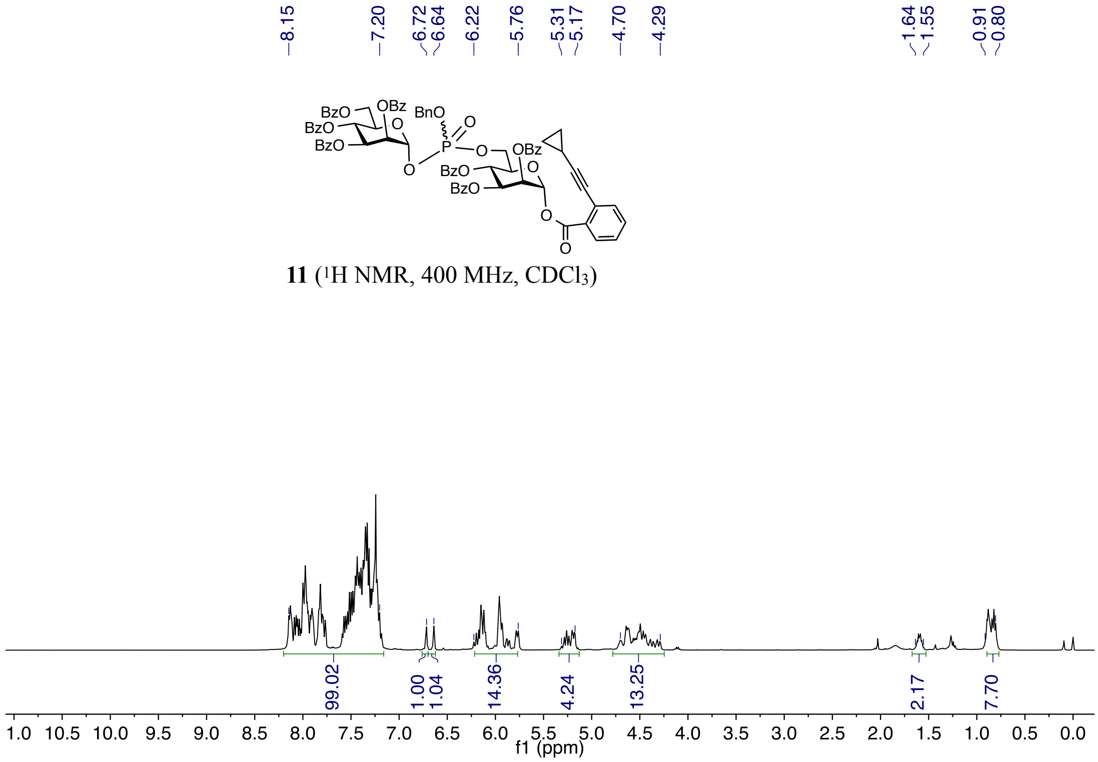

Supplementary Figure 296. ^{13}C NMR spectrum of compound **11**

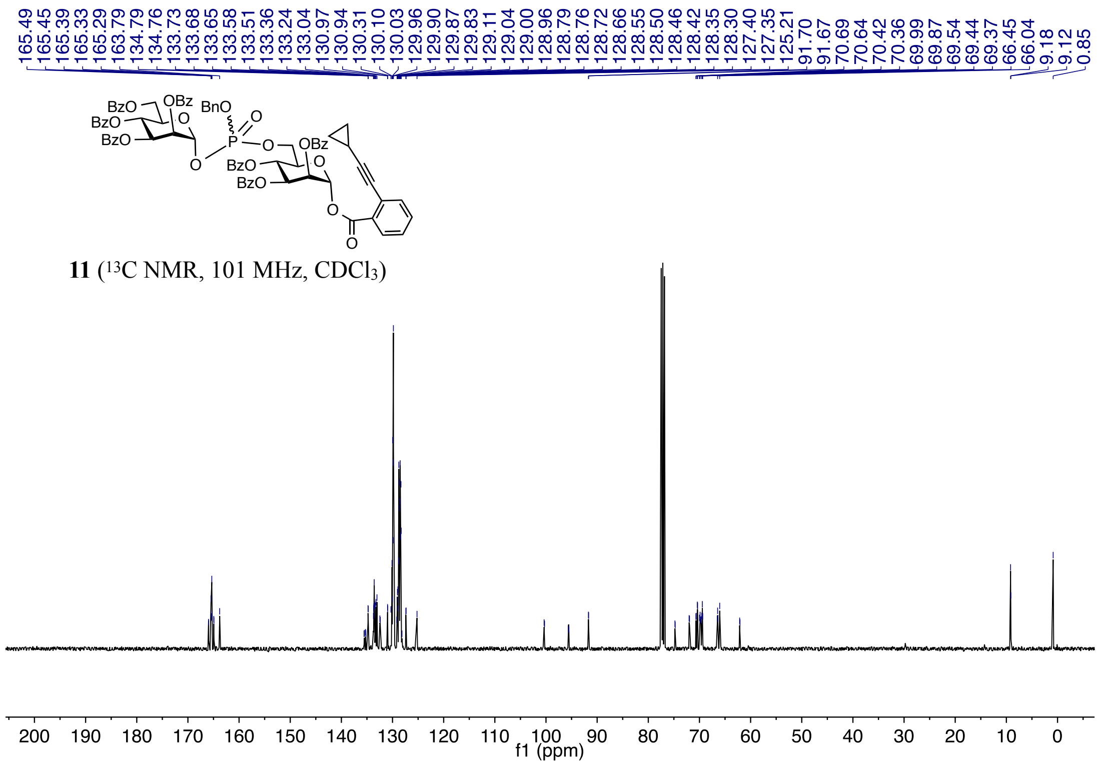

Supplementary Figure 297. ^{31}P NMR spectrum of compound **11**

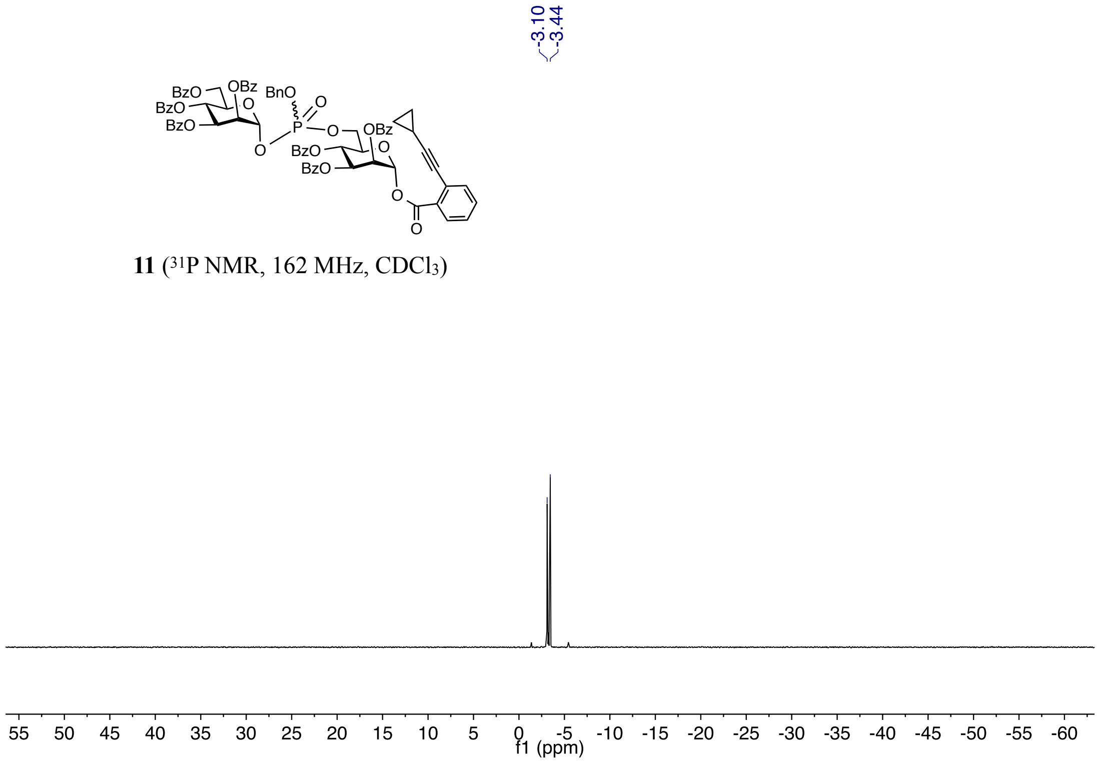

11 (^{31}P NMR, 162 MHz, $CDCl_3$)

Supplementary Figure 298. ^{1}H NMR spectrum of compound **12-H2**

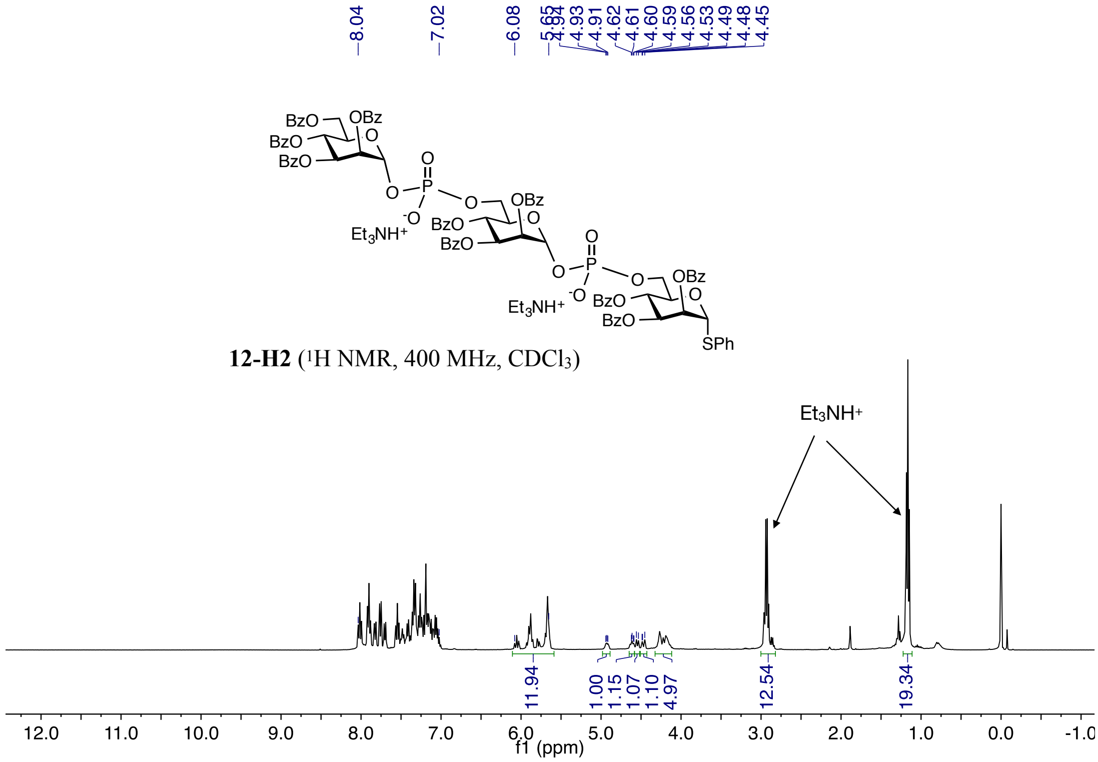

12-H2 (^{1}H NMR, 400 MHz, $CDCl_3$)

Supplementary Figure 299. ^{13}C NMR spectrum of compound **12-H2**

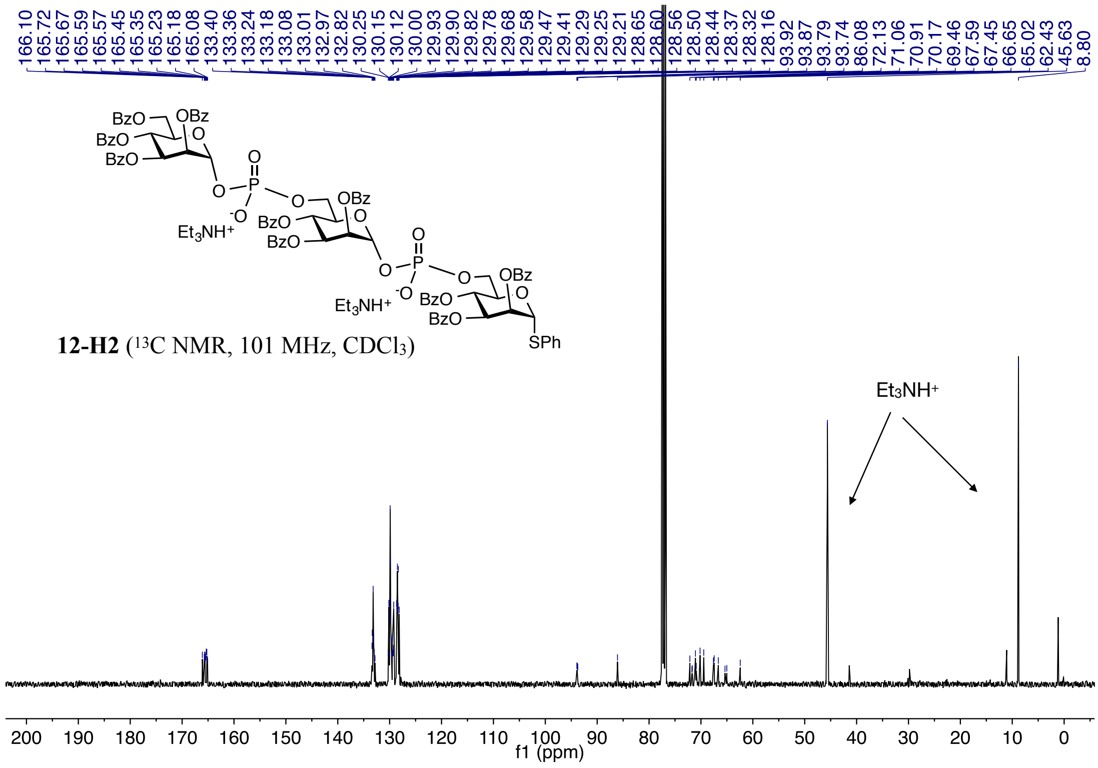

Supplementary Figure 300. ^{31}P NMR spectrum of compound **12-H2**

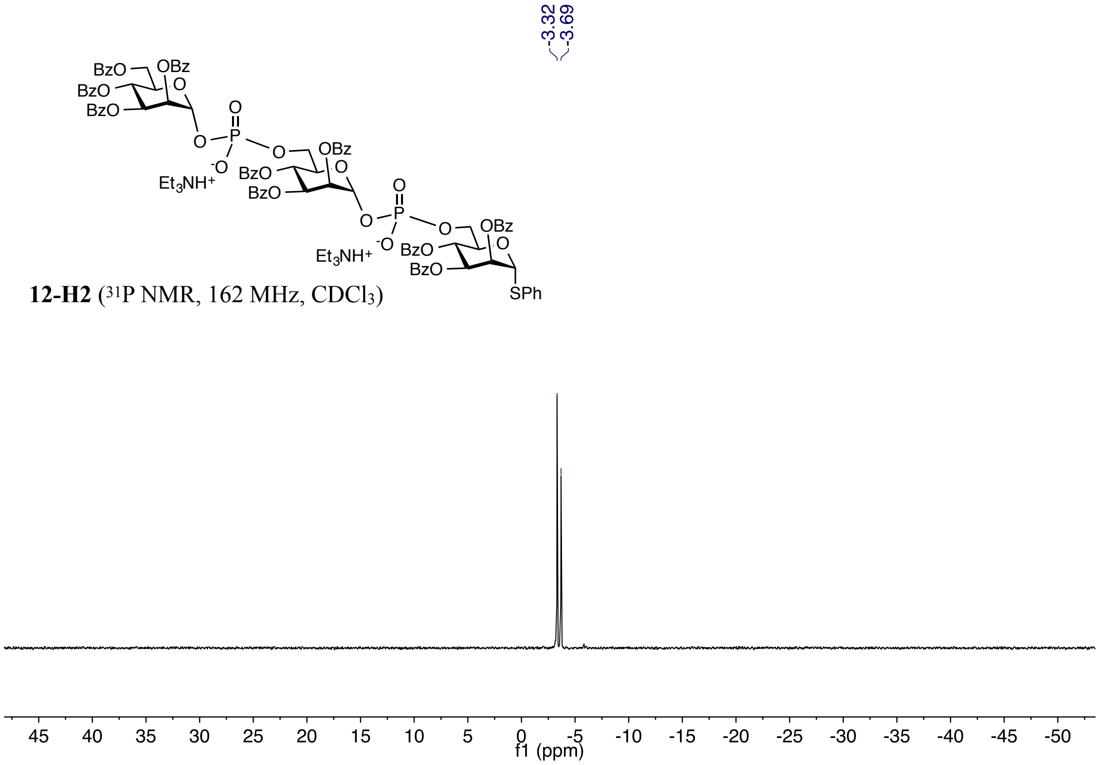

Supplementary Figure 301. ^{1}H NMR spectrum of compound **13**

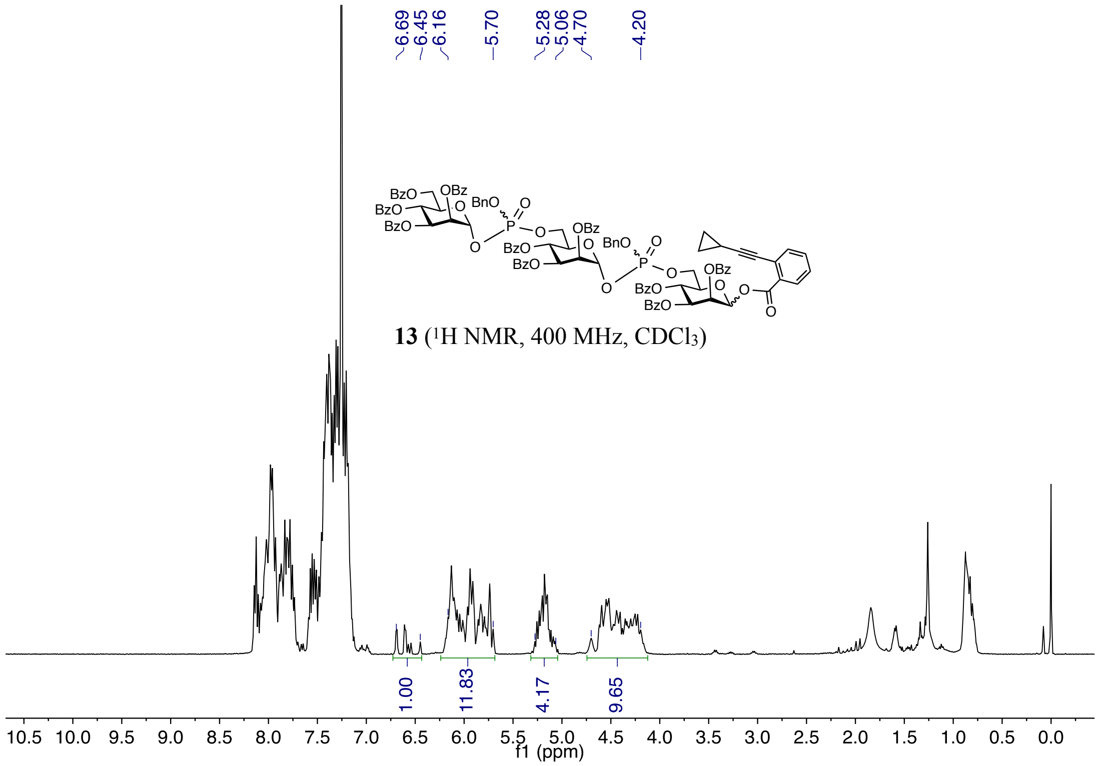

Supplementary Figure 302. ^{13}C NMR spectrum of compound **13**

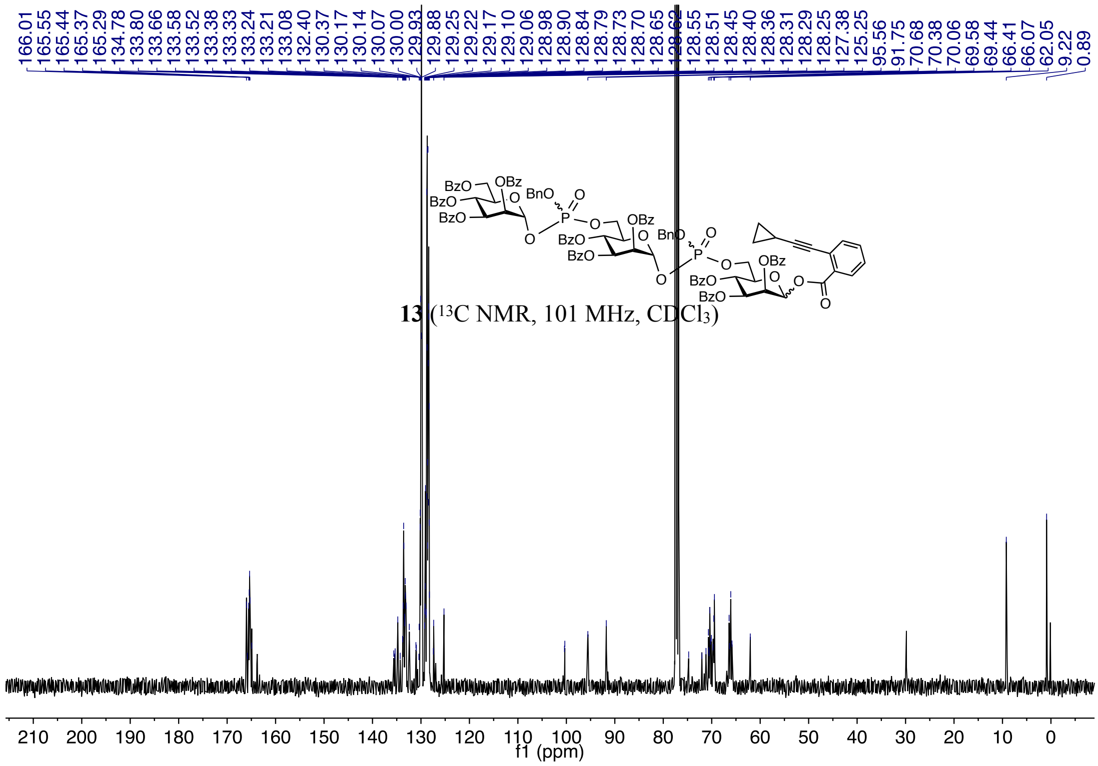

Supplementary Figure 303. ^{31}P NMR spectrum of compound **13**

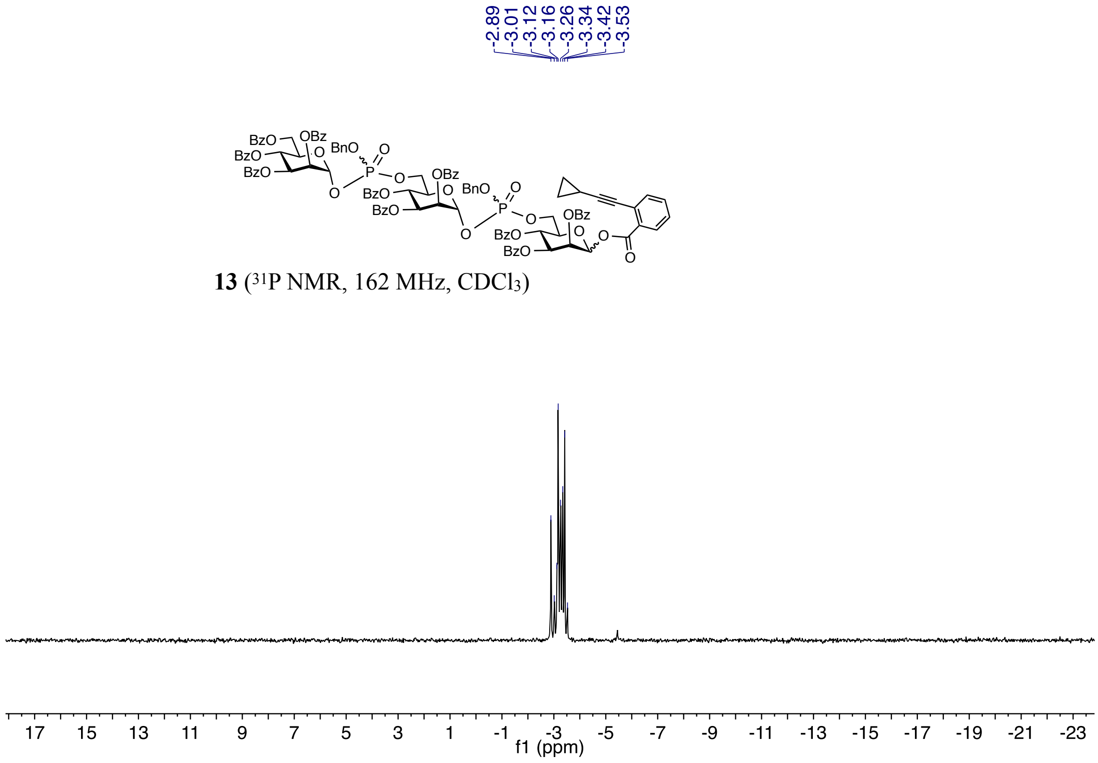

Supplementary Figure 304. ^{1}H NMR spectrum of compound **14**

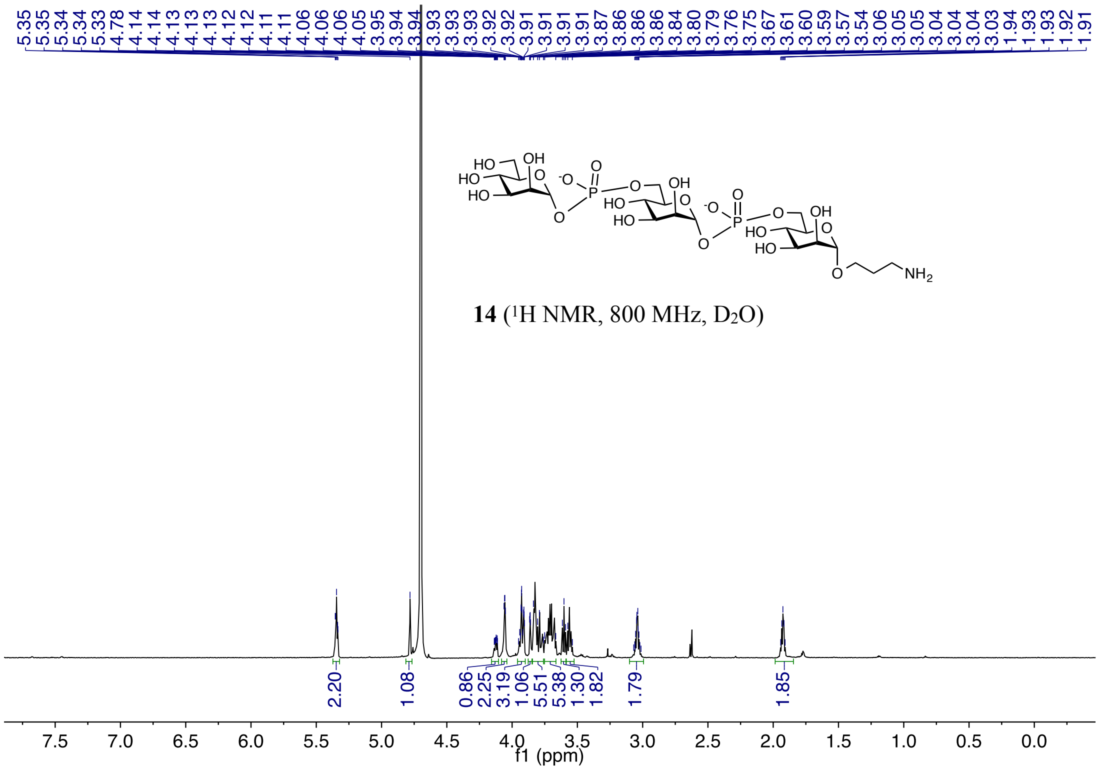

Supplementary Figure 305. ^{13}C NMR spectrum of compound **14**

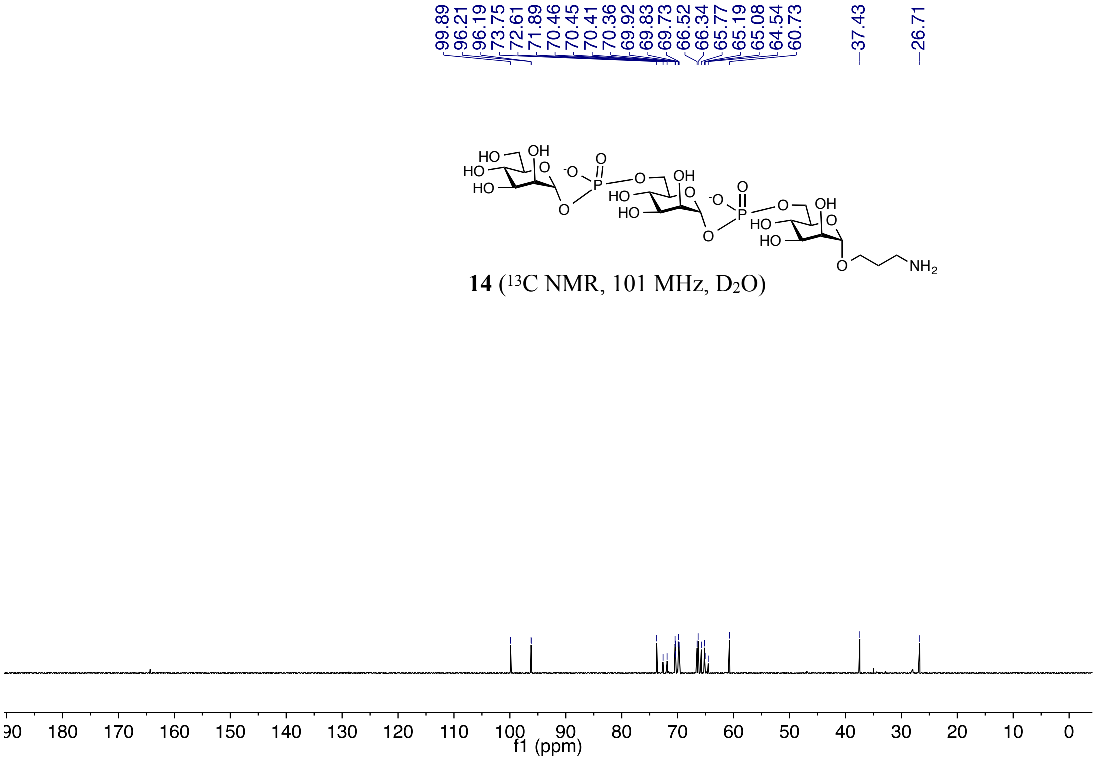

Supplementary Figure 306. ^{31}P NMR spectrum of compound **14**

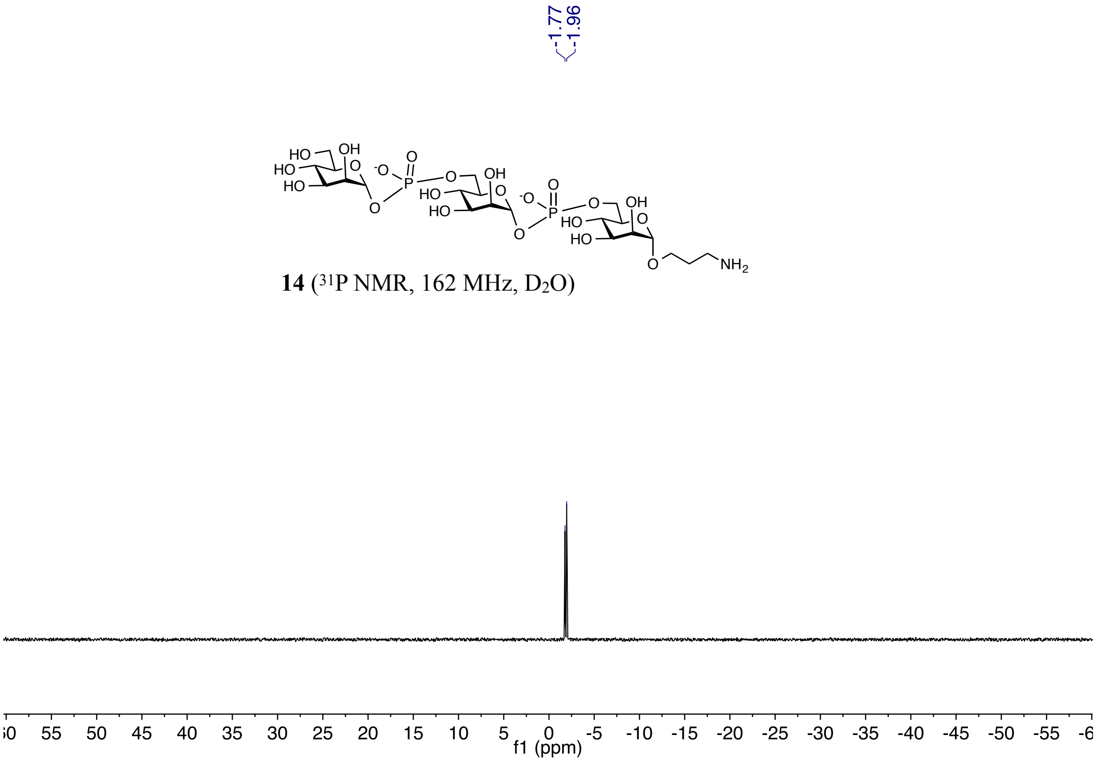

2. Supplementary References

The known compounds below were prepared according the corresponding references.

1. Yu, Biao et al. *J. Am. Chem. Soc.* **135**, 18396-18405, (2013)

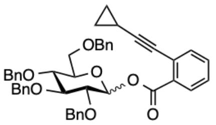

2. Hotha, Srinivas et al. *Chem. Commun.* **18**, 2505-2507, (2009)

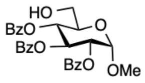

3. Townsend, Steven D. et al. *J. Am. Chem. Soc.* **141**, 12939-12945, (2019)

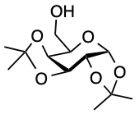

4. Dong, Hai et al. *Green Chemistry* **22**, 1139-1144, (2020)

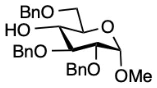

5. Hoff, Bard Helge et al. *Eur. J. Org. Chem.* 1592-1597, (2009)

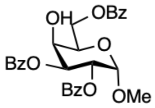

6. Qin, Yong et al. *J. Org. Chem.* **81**, 162-184, (2016)

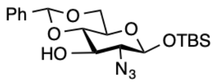

7. Allbritton, Nancy L. et al. *Angew. Chem. Int.Ed.* **55**, 13095-13098, (2016)

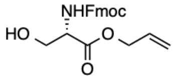

8. Gilmour, Ryan et al. *Angew. Chem. Int. Ed.* **49**, 8724-8728, (2010)

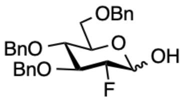

9. Gilmour, Ryan et al. *Angew. Chem. Int. Ed.* **49**, 8724-8728, (2010)

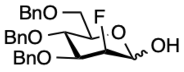

10. Gilmour, Ryan et al. *Chem. Eur. J.* **18**, 8208-8215, (2012)

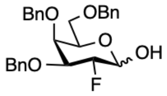

11. Moravcova, Jitka et al. *Carbohydr. Res.* **360**, 31-39, (2012)

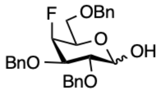

12. Lowary, Todd L. et al. *J. Org. Chem.* **83**, 7659-7671, (2018)

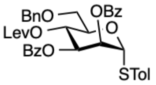

13. Wong, Chi-Huey et al. *J. Am. Chem. Soc.* **141**, 6484-6488, (2019)

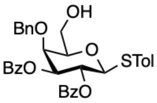

14. Van Boom et al. *Tetrahedron*, **49,** 6501-6514, (1993)

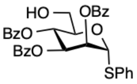

15. Yu, B. et al. *J. Am. Chem. Soc.* **135**, 18396-18405, (2013)

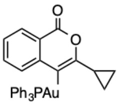

16. Lipták, A. et al. *Tetrahedron*, **58**, 5723-5732, (2002)

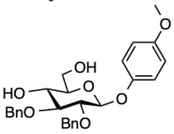

17. Yamasaki, R. et al. *Carbohydrate Research*, **337**, 11-20, (2002)

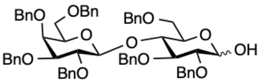

Supplement: Supplementary file 1 — supplementary information [file 41467_2022_28025_MOESM1_ESM.pdf]
